# Development of Electronic Health Record–Based Prediction Models for 30-Day Readmission Risk Among Patients Hospitalized for Acute Myocardial Infarction

**DOI:** 10.1001/jamanetworkopen.2020.35782

**Published:** 2021-01-29

**Authors:** Michael E. Matheny, Iben Ricket, Christine A. Goodrich, Rashmee U. Shah, Meagan E. Stabler, Amy M. Perkins, Chad Dorn, Jason Denton, Bruce E. Bray, Ram Gouripeddi, John Higgins, Wendy W. Chapman, Todd A. MacKenzie, Jeremiah R. Brown

**Affiliations:** 1 Department of Biomedical Informatics, Vanderbilt University Medical Center, Nashville, Tennessee; 2 Deparment of Biostatistics, Vanderbilt University Medical Center, Nashville, Tennessee; 3 Division of General Internal Medicine, Vanderbilt University Medical Center, Nashville, Tennessee; 4 Geriatric Research Education and Clinical Care Center, Tennessee Valley Healthcare System VA, Nashville; 5 Departments of Epidemiology and Biomedical Data Science, Dartmouth Geisel School of Medicine, Hanover, New Hampshire; 6 Division of Cardiovascular Medicine, University of Utah School of Medicine, Salt Lake City; 7 Department of Biomedical Informatics, University of Utah School of Medicine, Salt Lake City; 8 Centre for Clinical and Public Health Informatics, University of Melbourne, Melbourne, Victoria, Australia

## Abstract

**Importance:**

In the US, more than 600 000 adults will experience an acute myocardial infarction (AMI) each year, and up to 20% of the patients will be rehospitalized within 30 days. This study highlights the need for consideration of calibration in these risk models.

**Objective:**

To compare multiple machine learning risk prediction models using an electronic health record (EHR)–derived data set standardized to a common data model.

**Design, Setting, and Participants:**

This was a retrospective cohort study that developed risk prediction models for 30-day readmission among all inpatients discharged from Vanderbilt University Medical Center between January 1, 2007, and December 31, 2016, with a primary diagnosis of AMI who were not transferred from another facility. The model was externally validated at Dartmouth-Hitchcock Medical Center from April 2, 2011, to December 31, 2016. Data analysis occurred between January 4, 2019, and November 15, 2020.

**Exposures:**

Acute myocardial infarction that required hospital admission.

**Main Outcomes and Measures:**

The main outcome was thirty-day hospital readmission. A total of 141 candidate variables were considered from administrative codes, medication orders, and laboratory tests. Multiple risk prediction models were developed using parametric models (elastic net, least absolute shrinkage and selection operator, and ridge regression) and nonparametric models (random forest and gradient boosting). The models were assessed using holdout data with area under the receiver operating characteristic curve (AUROC), percentage of calibration, and calibration curve belts.

**Results:**

The final Vanderbilt University Medical Center cohort included 6163 unique patients, among whom the mean (SD) age was 67 (13) years, 4137 were male (67.1%), 1019 (16.5%) were Black or other race, and 933 (15.1%) were rehospitalized within 30 days. The final Dartmouth-Hitchcock Medical Center cohort included 4024 unique patients, with mean (SD) age of 68 (12) years; 2584 (64.2%) were male, 412 (10.2%) were rehospitalized within 30 days, and most of the cohort were non-Hispanic and White. The final test set AUROC performance was between 0.686 to 0.695 for the parametric models and 0.686 to 0.704 for the nonparametric models. In the validation cohort, AUROC performance was between 0.558 to 0.655 for parametric models and 0.606 to 0.608 for nonparametric models.

**Conclusions and Relevance:**

In this study, 5 machine learning models were developed and externally validated to predict 30-day readmission AMI hospitalization. These models can be deployed within an EHR using routinely collected data.

## Key Points

**Question:**

Can machine learning deployed in electronic health records be used to improve readmission risk estimation for patients following acute myocardial infarction?

**Findings:**

In this cohort study examining externally validated machine learning risk models for 30-day readmission of 10 187 patients following hospitalization for acute myocardial infarction, good discrimination performance was noted at the development site, but the best discrimination did not result in the best calibration. External validation yielded significant declines in discrimination and calibration.

**Meaning:**

The findings of this study highlight that robust calibration assessments are a necessary complement to discrimination when machine learning models are used to predict post–acute myocardial infarction readmission; challenges with data availability across sites, even in the presence of a common data model, limit external validation performance.

## Teaser

This cohort study compares multiple externally validated machine learning models using electronic health record data to predict 30-day readmission among patients hospitalized for acute myocardial infarction.

## Introduction

Coronary heart disease leads to approximately 14% of deaths in the US; an acute myocardial infarction (AMI) occurs every 34 seconds and an AMI-related death occurs every 84 seconds.^1^ Approximately 635 000 individuals within the US have their first AMI each year, and almost half experience a recurrent AMI the same year.^1^ One in 5 patients with AMI is rehospitalized within 30 days of discharge.^1,2,3^

The 2010 Patient Protection and Affordable Care Act identified hospital readmission as a target for revising fee-for-service hospital reimbursement and reducing health care spending.^4^ Unplanned readmissions account for 17% of Medicare hospital reimbursement, costing approximately $17.4 billion annually.^5^ Approximately 20% of Medicare beneficiaries who experience an AMI are re-hospitalized within 30 days after discharge.^6^ Although quality improvement initiatives and financial incentives have led to a decline in 30-day readmissions, emergency department visits and observation stays after index admission have increased.^7,8,9^ The success of readmission reduction programs is therefore uncertain, highlighting the need to identify patients who may benefit from additional health care resources to reduce the risk of readmission.

Risk prediction models are important to promote efficient resource allocation for high-risk patients and readmission prevention.^4,5,10^ There is increasing opportunity to embed predictive models into electronic health records (EHRs) to support automated readmission prediction for use by clinical teams, yet challenges persist.^11,12,13^ Portability of predictive models between different EHRs is often limited^10,11,12^ owing to systematic differences in the patient case-mix between institutions, differences in EHR data storage methods,^14^ and changes in clinical and data practices within a health care system over time.^15^ Strategies to address portability include a systematic approach to data management (eg, common data models and exchange formats) and model maintenance.^16,17^

Current models developed for 30-day readmission after AMI focus on risk factors derived from claims data or restricted patient populations, which limit performance and deployment.^18^ Many studies have had limited discrimination in predicting 30-day readmissions using either Medicare claims (*C* statistic, 0.63) or state registries with only structured data (*C* statistic, 0.64-0.67).^18,19,20,21,22^ Machine learning models can address some of these limitations by accounting for variable interactions and nonlinearity; however, there is limited transparency of model outputs.^23^ However, the ability to incorporate such models into EHRs, offload manual calculation burdens, and carefully select methods that are understandable have increased clinical usability.^24,25^

In this study, we sought to improve 30-day readmission risk prediction among hospitalized patients with AMI by analyzing a broad set of data collected from EHRs standardized within a common data model and comparing a robust set of machine learning methods.

## Methods

We conducted this study at Vanderbilt University Medical Center (VUMC), a large, tertiary care academic hospital system in Nashville, Tennessee, with a catchment area that includes a 9-state surrounding region. Dartmouth-Hitchcock Medical Center (DHMC), a tertiary care facility serving New Hampshire along with 3 neighboring states, was used as the external validation site. The initial inclusion cohort comprised all 10 731 patients hospitalized with primary *International Classification of Diseases, Ninth Revision, Clinical Modification* (*ICD-9-CM*) or *International Statistical Classification of Diseases, Tenth Revision, Clinical Modification (ICD-10-CM) *diagnosis codes of AMI between January 1, 2007, and December 31, 2016, and an aligned cohort of hospitalized patients discharged between April 2, 2011, and December 31, 2016, at DHMC. Data analysis was performed between January 4, 2019, and November 15, 2020.

We then excluded all patient hospitalizations that were not the index AMI hospitalization (VUMC, 4241 and DHMC, 2617) to ensure 1 hospitalization per patient, and excluded patients who died before discharge (VUMC, 327 and DHMC, 244). The final cohort was 6163 VUMC and 4024 DHMC unique patients. To supplement ascertainment of 30-day readmissions following hospitalization, the cohorts were linked to Medicare Provider Analysis and Review inpatient claims.^26^

All data were collected within the VUMC EHR, StarPanel^27,28^ and DHMC local EHR aggregated to a clinical data warehouse. Source EHR data were transformed at each institution into the Observational Medical Outcome Partnership (OMOP) Common Data Model. At VUMC, this was done by VICTR and supported by institutional and National Institutes of Health Clinical and Translational Science Award funding. At DHMC, this was done by the research team. The OMOP supports normalization of data variables encoded using different terminologies and data structures and is well known for its usefulness for clinical data.^17,29^ All structured data fields in this study were extracted from each site’s OMOP instance.

We followed the Transparent Reporting of a Multivariable Prediction Model for Individual Prognosis or Diagnosis (TRIPOD) reporting guideline for cohort studies.^30^ This study was approved by the VUMC and Dartmouth College institutional review boards under expedited review with a waiver of informed consent. Informed consent was waived because the work could not feasibly be done with direct informed consent, and the study represented minimal risk to the participants as determined by the institutional review boards.

### Candidate Predictors

Main effect predictors and definitions are listed in eTable 1 in the Supplement, and these included 4 demographic variables, 9 medication orders, 86 administration variables, 9 composite score variables, and 33 laboratory tests. Variables were defined using *ICD-9-CM*, *ICD-10-CM*, Current Procedural Terminology, and Healthcare Common Procedure Coding System codes, and translated to SNOMED-CT using the Unified Medical Language System crosswalk, where the crosswalks existed, to query the OMOP tables. To more fairly compare the parametric methods, which cannot automatically evaluate interaction terms and nonlinear variable representations in the way that the nonparametric methods can, we evaluated first-order interaction terms using forward and backward step logistic regression, with α = .10 as a threshold for retention of the interaction term variable. A full list of variable candidate predictors is available in eTable 8 in the Supplement. All candidate predictors generated at VUMC were replicated at DHMC.

### Outcome

The main outcome of interest was 30-day hospital readmission. Using the Centers for Medicare & Medicaid Services definition, readmission was defined to be a subsequent stay in the hospital for observation or an acute inpatient stay within 30 days from the index AMI discharge, and excluding rehabilitation admissions, nursing home admissions, or scheduled admissions for surgeries or procedures. The dates and causes for readmission were derived from each hospital’s administrative databases, including the admitting hospital’s state and surrounding state inpatient data sets, and Medicare claims, ensuring complete ascertainment of 30-day readmissions. Outcome derivation was the same at VUMC and DHMC.

### Missing Values

The final analytic file contained 37 variables with missing values at VUMC and 30 at DHMC. We addressed these issues through a combination of assumptions and imputation techniques. Imputation, when data are missing at random and isolated to the predictor variables, is necessary, and multiple imputation provides robust results.^31^ Except for laboratory test data, clinical information was assumed to be negative or not present when null in the EHR data. For laboratory variables, SAS, version 9.4 (SAS Institute Inc) was used to create 20 imputed data sets using Markov-chain Monte Carlo methods, assuming all imputed variables have a multivariate normal distribution.^32^ Missing data were derived by drawing from a conditional multivariate normal distribution. With sufficiently large samples, this method often leads to reliable estimates, even if the assumption of normality is not fully met.^32^

The final files at VUMC and DHMC contained a total of 241 variables, including 141 main effects, 99 second-order terms, and 1 outcome. Each site contained 20 imputed data files, with 123 260 observations at VUMC and 80 480 observations at DHMC. Having 20 data files allows for enough uncertainty about the missing values to be confident about the variables’ influence on outcome. The final analytic data files were imported into R, version 3.6.0 (R Foundation) for machine learning development and execution.

### Statistical Analysis

#### Model Development

Five machine learning models were developed and included both parametric models (elastic net [EN], least absolute shrinkage and selection operator [LASSO], and ridge regression [RR]) and nonparametric models (random forest [RF] and gradient boosting [GB]). Final models were selected from the best-performing models in each method by evaluating both the default hyperparameter settings for each model method in R, as well as a grid search on the key hyperparameter features of each nonparametric model, using the caret package in R with 5-fold cross validation to determine hyperparameters. The trainControl algorithm in the caret package builds a grid of possible parameters to optimize the hyperparameters for use in the final model. All numbers listed initially with each hyperparameter are the default values.

Hyperparameters for the parametric models included α and λ. The α hyperparameters are, by definition, fixed to 1 for LASSO and 0 for RR. Following a grid search using the caret package, α was set to 0.55 for EN. Each parametric model was assessed using both λ minimum and 1 SE from λ minimum.

Hyperparameters for RF were the number of drawn candidate variables in each split (11.87), the sample size of observations (n), whether observations are drawn with replacement (true), node size (1), number of trees (500), and splitting rule (Gini impurity, *P* value, random).^33^ A grid search resulted in changing the number of trees to 1000 and the number of drawn candidate variables in each split to 7; default values were retained for the remaining hyperparameters.

Hyperparameters for GB were the number of trees (100), interaction depth/maximum node per tree (4), minimum number of samples in tree terminal nodes (10), the fraction of training observations randomly selected for the subsequent tree (0.5), the train fraction (1), and learning rate (0.01). The grid search optimized the number of trees to 7, interaction depth/maximum node per tree (1), and shrinkage parameter (0.537).^34^

Before deploying each machine learning model, the final analytic data file was randomly split into two-thirds training set and one-third testing set in each of the 20 imputed data sets. Parametric models were developed in R, using the glmnet and caret packages.^35^ Nonparametric models used RandomForest and gbm libraries.^34,36^

#### Model Assessment and Scoring

Each model was trained using 10-fold cross validation on the full training set with 5 repeats. Model performance was determined using the full holdout test set. The area under the receiver operating characteristic curve (AUROC), 95% CIs, and SE were calculated from the test set for each imputed data set (1-20) for each model. The AUROC, SE, and 95% CIs were pooled across all imputed files using Rubin indexes to generate a single metric for each distinct machine learning model. Various thresholds for predicted probabilities were investigated. Calibration was assessed using calibration curve belts and percentage of calibration (proportion of predictions in which the calibration belt crossed the observed/expected 1 line) for each model on the training and testing data sets.^37^ We report these calibration results for model comparisons. The pooled Brier score was then assessed, which is a global metric that combines discrimination and calibration performance.^38^ The best-performing model was defined as the model with the most observations falling within the calibration band.^39^

After deployment of each model, scoring was performed using the DHMC data. The models were scored on the full DHMC data set, using models with default and optimized hyperparameters. Model discrimination was assessed with pooled AUROCs and calibration was evaluated with calibration curve belts and percentage of calibration, following the methods described with the Model Assessment section.

## Results

Among 6163 patients at VUMC, 933 (15.1%) were readmitted within 30 days, 2026 (32.9%) were female, 4137 (67.1%) were male, 1019 (16.5%) were Black or other race, and most were Hispanic. Mean (SD) age was 67 (13) years. Among 4024 patients at DHMC, 412 (10.2%) were readmitted within 30 days, 1440 (35.8%) were female, 2584 (64.2%) were male, and most were non-Hispanic and White. Mean (SD) age was 68 (12) years. Low cell thresholds limited specific release of race and ethnicity data. Table 1 and eTable 2 in the Supplement present additional patient characteristics at the sites. The list of demographic and clinical variables derived from the OMOP-transformed variables can be found in eTable 1 in the Supplement and a full variable list is provided in eTable 8 in the Supplement.

**Table 1.** Characteristics for 6194 Patients Hospitalized at Vanderbilt University Medical Center With a Primary Diagnosis of AMI Cohort

Characteristic	No. (%)	
	Readmission (n = 933)	Nonreadmission (n = 5230)
Sex		
Male	592 (63.5)	3545 (67.8)
Female	341 (36.5)	1685 (32.2)
Race		
White	781 (83.7)	4363 (83.4)
Black	101 (10.8)	491 (9.4)
Other	51 (5.5)	376 (7.2)
Comorbidities		
Arrhythmia	196 (21.0)	665 (12.7)
Anemia	159 (17.0)	429 (8.2)
Hypertension	357 (38.3)	1579 (30.2)
COPD	42 (4.5)	150 (2.9)
CKD	148 (15.9)	353 (6.7)
Tobacco use	58 (6.2)	246 (4.7)
Depression	64 (6.9)	217 (4.1)
CAD	96 (10.3)	527 (10.1)
CHF	197 (21.1)	598 (11.4)
Dementia	24 (2.6)	101 (1.9)
Cardiac arrest	53 (5.7)	269 (5.1)
STEMI	450 (48.2)	2651 (50.7)
During hospitalization		
Heart failure	496 (53.2)	1870 (35.8)
Ischemia	159 (17.0)	600 (11.5)
History		
AMI	224 (24.0)	1122 (21.5)
Peripheral vascular disease	198 (21.2)	647 (12.4)
Angina	142 (15.2)	574 (11.0)
Unstable angina	228 (24.4)	1042 (19.9)
Hypertension	476 (51.0)	2240 (42.8)
Depression	120 (12.9)	535 (10.2)
Discharge location		
Home	729 (78.1)	4670 (89.3)
Health facility	204 (21.9)	560 (10.7)
Continuous scores, mean (SD)		
Age, y	67.78 (13.05)	63.22 (12.99)
LACE score^a^	5.71 (2.36)	4.67 (2.00)
GRACE score^b^	141.06 (33.3)	129.55 (33.19)
HOSPITAL score^c^	3.41 (1.64)	2.63 (1.58)
Charlson Deyo score	1.19 (1.86)	0.75 (1.86)
Length of stay, d	7.47 (5.64)	5.67 (5.06)

Abbreviations: AMI, acute myocardial infarction; CAD, coronary artery disease; CHF, congestive heart failure; CKD, chronic kidney disease; COPD, chronic obstructive pulmonary disease; STEMI, ST-elevation myocardial infarction.

^a^ LACE indicates length of stay, acuity of the admission, comorbidity of the patient (measured with the Charlson comorbidity index score), and emergency department use (measured as the number of visits in the 6 months before admission). Possible score range is 1 to 19.

^b^ GRACE indicates Global Registry of Acute Coronary Events; possible score is 1 to 372 points.

^c^ HOSPITAL indicates hemoglobin level at discharge, discharge from an oncology service, sodium level at discharge, procedure during the index admission, index type of admission, number of admissions during the past 12 months, and length of stay. Possible score range is 0 to 13.

The final features of the parametric (EN, LASSO, RR) models are shown as pooled coefficients in eTable 3, eTable 4, and eTable 5 in the Supplement, respectively. Although parameters cannot be directly represented in the same way for the nonparametric models (RF and GB), we included the commonly reported features from the pooled RF and GB models (eTable 6 and eTable 7 in the Supplement).

For the parametric models EN, LASSO, and RR, the testing sets’ AUROC level was between 0.686 and 0.695, and for the nonparametric models, the testing sets’ AUROC level was from 0.686 for RF to 0.704 for GB (Table 2). The best-performing EN, LASSO, RR, and GB models occurred with default hyperparameters, and the best-performing RF models occurred with optimized hyperparameters. Among the external validation cohort, the best-performing parametric and nonparametric models occurred with optimized hyperparameters. The AUROC for parametric models was between 0.558 to 0.655 and, for the nonparametric models, the AUROC was between 0.606 and 0.608 (Table 2).

**Table 2.** Pooled AUROC for Train/Test on VUMC and Scored DHMC Machine Learning Models With Pooled 95% CIs

Model name	VUMC		DHMC, validation
	Train	Test	
Elastic net	0.732 (0.709-0.755)	0.695 (0.646-0.745)	0.655 (0.555-0.760)
LASSO	0.731 (0.708-0.755)	0.695 (0.645-0.744)	0.595 (0.452-0.738)
Ridge regression	0.735 (0.708-0.719)	0.686 (0.713-0.757)	0.558 (0.462-0.654)
Random forest	0.695 (0.671-0.719)	0.686 (0.632-0.741)	0.608 (0.569-0.648)
Gradient boosting	0.731 (0.710 0.753)	0.704 (0.650-0.759)	0.606 (0.540-0.671)

Abbreviations: AUROC, area under the receiver operating characteristic curve; DHMC, Dartmouth-Hitchcock Medical Center; LASSO, least absolute shrinkage and selection operator; VUMC, Vanderbilt University Medical Center.

**Figure 1.**
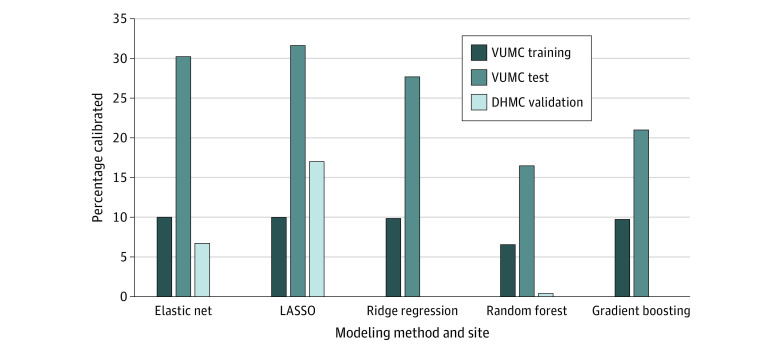
Percentage Calibrated for Train/Test on Vanderbilt University Medical Center (VUMC) and Scored on Dartmouth-Hitchcock Medical Center (DHMC) Machine Learning Models Bars represent the percentage of aligned risk predictions. LASSO indicates least absolute shrinkage and selection operator.

**Figure 2.**
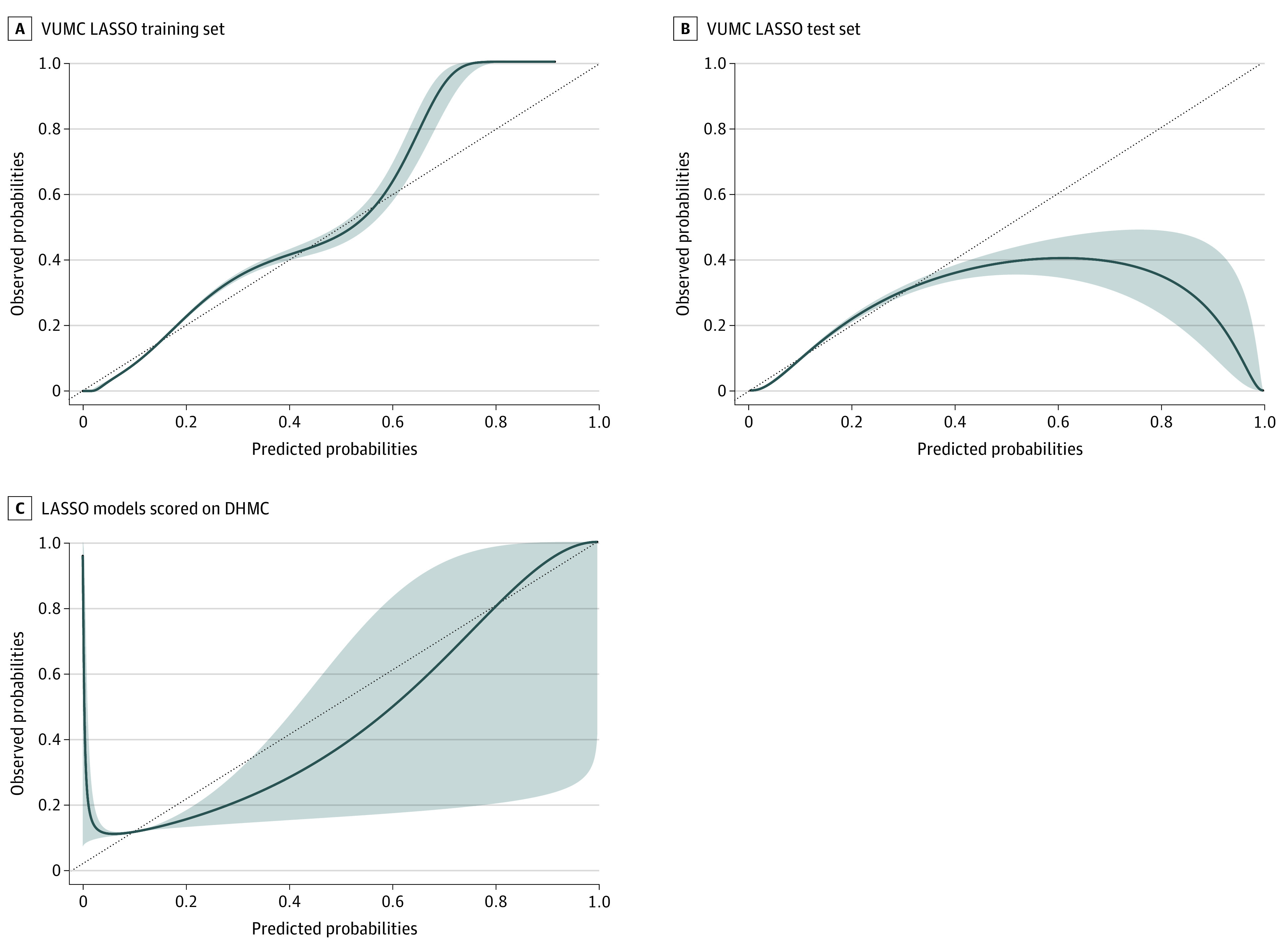
Calibration Curve Belts Fitted From Fitted and Predicted Values for the Final Least Absolute Shrinkage and Selection Operator (LASSO) Model LASSO training (A) and test (B) sets for Vanderbilt University Medical Center (VUMC), with models scored at Dartmouth-Hitchcock Medical Center (DHMC) (C). The diagonal line represents an observed/expected (O/E) slope of 1.0. The shaded area is the area within the CI of the fitted calibration curve for the O/E ratio across the range of predicted probabilities.

For calibration assessments on VUMC testing data, the best-performing model was LASSO, which had the highest percentage of calibrated observations (31.64%), followed closely by EN (30.24%), with both using default hyperparameters (Figure 1). The model with the highest percentage of calibration among the external validation cohort was LASSO (17.0%) using optimized hyperparameters. Figure 2 illustrates the calibration curves for the best-performing LASSO models within both cohorts. Additional calibration curves for the other 4 models can be found in eFigure 1 and eFigure 2 in the Supplement. For VUMC, multiple thresholds were tested for sensitivity, specificity, positive predictive value, negative predictive value, and the F1 score for the best-performing LASSO model (Table 3). Predictors that were repeatedly among the strongest in the models were discharge location, age, hospital score, hemoglobin level, and troponin level.

**Table 3.** Performance Metrics (Thresholds 0.1-0.5) on the Testing Data for Final VUMC LASSO Model

Threshold	Sensitivity	Specificity	PPV	NPV	F1
0.1	0.675	0.338	0.154	0.853	0.250
0.15	0.410	0.625	0.163	0.856	0.223
0.2	0.252	0.784	0.171	0.855	0.204
0.25	0.156	0.875	0.180	0.854	0.166
0.3	0.089	0.930	0.182	0.852	0.119
0.35	0.049	0.962	0.186	0.851	0.078
0.4	0.029	0.980	0.199	0.850	0.050
0.45	0.017	0.989	0.199	0.850	0.035
0.5	0.010	0.994	0.232	0.850	0.026

Abbreviations: LASSO, least absolute shrinkage and selection operator; VUMC, Vanderbilt University Medical Center.

## Discussion

In this study, we developed EHR-derived machine learning risk prediction models that performed better than previously published models in the derivation site while retaining good calibration.^22,40,41,42^ Moreover, we externally validated the machine learning risk models at an independent site, highlighting challenges in retaining adequate calibration at nonderivation sites. We chose LASSO as the model with the best fit owing to several factors, because the AUROCs were not statistically significantly different, and we primarily targeted calibration plots’ proximity to the diagonal fit line. Among the options, our selection of LASSO as the optimal model may seem counterintuitive because it did not offer the highest AUROC, but it appears to represent a balanced prioritization between discrimination and calibration performance. Our hope is that robust calibration assessment becomes the norm for risk model development.^39^ Discrimination metrics among the external validation cohort were poor for LASSO and RR models; however, EN, RF, and GB models experienced less discrimination decline. However, calibration was very poor for RR, RF, and GB models, which suggests overfitting to the derivation data.

Previous studies have highlighted the need for ongoing surveillance and updating of these models during their use, as there are systematic data collection differences between sites and clinical practice drift over time.^15,43^ Although external validation is an important metric for risk models, this study highlights that even when developed using a common data model to align data definitions and, in the same time period, a model derivation, portability to other sites can be compromised. It is likely that almost all models will require adaptation to local environments and continued updating over time to achieve clinical utility and safety.

Several publications have assessed all-cause readmissions after cardiovascular events,^40,44,45^ but few are specific to AMI^41,46,47,48,49^ and, to our knowledge, none of the AMI literature has compared machine learning models derived from automated EHR-mapped risk factors. Dodson and colleagues^48^ recently published SILVER-AMI, an ensemble model trained and tested in 3006 patients with AMI with a *C* statistic of 0.63 in the validation population. In a systematic review, Smith et al^41^ identified 11 studies covering 16 different readmission models (median *C* statistic, 0.65; range, 0.53-0.79). Of these 16 models, half used validation in the form of a split cohort or bootstrapping, and only 1 model was validated more broadly on a separate cohort. The *C* statistic in the study by Meddings et al^40^ was 0.79, but the model derivation sample size was small (n = 833). In READMITS, Nguyen et al^46^ studied 30-day readmission for 876 patients using backward-step parameter selection and 5-fold cross validation; the resulting model discrimination was 0.75. Hebert and colleagues^45^ developed an AMI-specific model initially reporting an AUROC value of 0.76. When validated with a historical cohort for 2 years of retrospective data, however, the validation dropped to 0.66. A test of an augmented Centers for Medicare & Medicaid Services model to predict events after AMI from the TRACE-CORE cohort reported *C* statistics from 0.62 to 0.65 but was poorly calibrated.^45^

Concurrent comparison of calibration with discrimination is necessary. Previous literature establishes that discrimination is relatively stable and reliable during model development and validation. However, calibration drifts quickly over time,^50^ and model calibration varies highly by feature availability and sample size even in initial model derivation, within ranges that appear insensitive to AUC performance.^15^ For this reason, it is hard to compare earlier developed models that do not include calibration metrics.

We have extended earlier studies in this domain by comparing a variety of risk models and candidate variable pools.^41,46,47^ The underlying assumptions differ among models and vary between parametric and nonparametric. This type of robust comparison has seen recent use in predictive modeling but is not yet widely practiced.^15^

### Limitations

There are several limitations to this study. There were data quality limitations at the external validation site, such that candidate predictor variables that were available at VUMC could not be populated at DHMC. This factor limited the number of available candidate predictors for the VUMC models, which affected their performance and thus the quality of available variables at DHMC, thereby also affecting the model scoring performance. Despite the use of a common data model, standardized variable definitions, and code sharing, nuances in local EHR mappings limited the availability of data at the external validation site. Focus on methods to establish enhanced data interoperability across sites may be warranted in future studies.

In addition, there are limitations to deploying this model in clinical practice. In the absence of significant data quality differences across sites, the use of rigorous EHR-mapped variables using OMOP Common Data Model can incorporate EHR-structured variables into an automated risk prediction toolkit. Multiple imputation was used for missing variables, and this feature may be unavailable in a real-time production environment; thus, an alternative strategy of simple imputation might be needed. In addition, implementation of any model in clinical practice would require surveillance and potential recalibration to the local environment.

## Conclusions

In this study, we developed and externally validated an EHR-derived readmission risk prediction model for use among patients hospitalized for AMI. We developed the models within a framework for comparison of candidate modeling methods and selected the method that maintained a balance between calibration and discrimination among the candidates. This model development framework can assist in selecting a model for deployment within an EHR environment to support prioritization of limited resources for reducing the likelihood of readmission among these patients.

## Notes

Supplement. **eTable 1.** Possible ML Variables **eTable 2.** Patient Characteristics for 4024 Patients Hospitalized at Dartmouth-Hitchcock Medical Center With a Primary Diagnosis of Acute Myocardial Infarction AMI Cohort **eFigure 1.** Calibration Curves for Final VUMC Models **eFigure 2.** Calibration Curves for Final DHMC Models **eTable 3.** Final Elastic Net Model Parameter Coefficients for VUMC **eTable 4.** Final LASSO Model Parameter Coefficients for VUMC **eTable 5.** Final Ridge Regression Model Parameter Coefficients for VUMC **eTable 6.** Pooled Variable Importance Results of Final Random Forest Model for VUMC **eTable 7.** Pooled Relative Influence Values from Final Gradient Boosting Model for VUMC **eTable 8.** Full Variable List Value Set Definitions

## References

[1] Mozaffarian D, Benjamin EJ, Go AS, ; American Heart Association Statistics Committee and Stroke Statistics Subcommittee Heart disease and stroke statistics—2015 update: a report from the American Heart Association. Circulation. 2015;131(4):e29-e322. doi:10.1161/CIR.0000000000000152 25520374

[2] Medicare Payment Advisory Commission Report to the Congress: Medicare and the health care delivery system. Published June 2013 Accessed December 18, 2020. http://medpac.gov/docs/default-source/reports/jun13_entirereport.pdf

[3] Benjamin EJ, Muntner P, Alonso A, ; American Heart Association Council on Epidemiology and Prevention Statistics Committee and Stroke Statistics Subcommittee Heart disease and stroke statistics—2019 update: a report from the American Heart Association. Circulation. 2019;139(10):e56-e528. doi:10.1161/CIR.0000000000000659 30700139

[4] Donzé J, Aujesky D, Williams D, Schnipper JL Potentially avoidable 30-day hospital readmissions in medical patients: derivation and validation of a prediction model. JAMA Intern Med. 2013;173(8):632-638. doi:10.1001/jamainternmed.2013.3023 23529115

[5] Rana S, Tran T, Luo W, Phung D, Kennedy RL, Venkatesh S Predicting unplanned readmission after myocardial infarction from routinely collected administrative hospital data. Aust Health Rev. 2014;38(4):377-382. doi:10.1071/AH14059 25001433

[6] Jencks SF, Williams MV, Coleman EA Rehospitalizations among patients in the Medicare fee-for-service program. N Engl J Med. 2009;360(14):1418-1428. doi:10.1056/NEJMsa0803563 19339721

[7] Centers for Medicare & Medicaid Services Hospital Readmissions Reduction Program (HRRP). Published 2013 Accessed December 18, 2020. http://www.cms.gov/Medicare/Medicare-Fee-for-Service-Payment/AcuteInpatientPPS/Readmissions-Reduction-Program.html

[8] Angelelli J, Gifford D, Intrator O, Gozalo P, Laliberte L, Mor V Access to postacute nursing home care before and after the BBA. Health Aff (Millwood). 2002;21(5):254-264. doi:10.1377/hlthaff.21.5.254 12224890

[9] Gerhardt G, Yemane A, Apostle K, Oelschlaeger A, Rollins E, Brennan N Evaluating whether changes in utilization of hospital outpatient services contributed to lower Medicare readmission rate. Medicare Medicaid Res Rev. 2014;4(1):mmrr2014.004.01.b03. doi:10.5600/mmrr.004.01.b03 25009762 PMC4086854

[10] Au AG, McAlister FA, Bakal JA, Ezekowitz J, Kaul P, van Walraven C Predicting the risk of unplanned readmission or death within 30 days of discharge after a heart failure hospitalization. Am Heart J. 2012;164(3):365-372. doi:10.1016/j.ahj.2012.06.010 22980303

[11] Choudhry SA, Li J, Davis D, Erdmann C, Sikka R, Sutariya B A public-private partnership develops and externally validates a 30-day hospital readmission risk prediction model. Online J Public Health Inform. 2013;5(2):219. doi:10.5210/ojphi.v5i2.4726 24224068 PMC3812998

[12] Amarasingham R, Audet AM, Bates DW, Consensus statement on electronic health predictive analytics: a guiding framework to address challenges. EGEMS (Wash DC). 2016;4(1):1163. doi:10.13063/2327-9214.1163 27141516 PMC4837887

[13] Cholleti S, Post A, Gao J, Leveraging derived data elements in data analytic models for understanding and predicting hospital readmissions. AMIA Annu Symp Proc. 2012;2012:103-111. 23304278 PMC3540449

[14] Weiskopf NG, Weng C Methods and dimensions of electronic health record data quality assessment: enabling reuse for clinical research. J Am Med Inform Assoc. 2013;20(1):144-151. doi:10.1136/amiajnl-2011-000681 22733976 PMC3555312

[15] Davis SE, Lasko TA, Chen G, Siew ED, Matheny ME Calibration drift in regression and machine learning models for acute kidney injury. J Am Med Inform Assoc. 2017;24(6):1052-1061. doi:10.1093/jamia/ocx030 28379439 PMC6080675

[16] Rosenbloom ST, Carroll RJ, Warner JL, Matheny ME, Denny JC Representing knowledge consistently across health systems. Yearb Med Inform. 2017;26(1):139-147. doi:10.15265/IY-2017-018 29063555 PMC6239235

[17] FitzHenry F, Resnic FS, Robbins SL, Creating a common data model for comparative effectiveness with the observational medical outcomes partnership. Appl Clin Inform. 2015;6(3):536-547. doi:10.4338/ACI-2014-12-CR-0121 26448797 PMC4586341

[18] Keenan PS, Normand SL, Lin Z, An administrative claims measure suitable for profiling hospital performance on the basis of 30-day all-cause readmission rates among patients with heart failure. Circ Cardiovasc Qual Outcomes. 2008;1(1):29-37. doi:10.1161/CIRCOUTCOMES.108.802686 20031785

[19] Hannan EL, Zhong Y, Krumholz H, 30-Day readmission for patients undergoing percutaneous coronary interventions in New York state. JACC Cardiovasc Interv. 2011;4(12):1335-1342. doi:10.1016/j.jcin.2011.08.013 22192374

[20] Yeh RW, Rosenfield K, Zelevinsky K, Sources of hospital variation in short-term readmission rates after percutaneous coronary intervention. Circ Cardiovasc Interv. 2012;5(2):227-236. doi:10.1161/CIRCINTERVENTIONS.111.967638 22438431

[21] Khawaja FJ, Shah ND, Lennon RJ, Factors associated with 30-day readmission rates after percutaneous coronary intervention. Arch Intern Med. 2012;172(2):112-117. doi:10.1001/archinternmed.2011.569 22123752 PMC3688066

[22] Krumholz HM, Lin Z, Drye EE, An administrative claims measure suitable for profiling hospital performance based on 30-day all-cause readmission rates among patients with acute myocardial infarction. Circ Cardiovasc Qual Outcomes. 2011;4(2):243-252. doi:10.1161/CIRCOUTCOMES.110.957498 21406673 PMC3350811

[23] Shmueli G, Koppius OR Predictive analytics in information systems research. Manage Inf Syst Q. 2011;35(3):553-572. doi:10.2307/23042796

[24] Cronin PR, Greenwald JL, Crevensten GC, Chueh HC, Zai AH Development and implementation of a real-time 30-day readmission predictive model. AMIA Annu Symp Proc. 2014;2014:424-431. 25954346 PMC4419988

[25] Watson AJ, O’Rourke J, Jethwani K, Linking electronic health record-extracted psychosocial data in real-time to risk of readmission for heart failure. Psychosomatics. 2011;52(4):319-327. doi:10.1016/j.psym.2011.02.007 21777714 PMC4452280

[26] Centers for Medicare & Medicaid Services MEDPAR Limited Data Set (LDS). Published 2019 Accessed December 18, 2020. https://www.cms.gov/Research-Statistics-Data-and-Systems/Files-for-Order/LimitedDataSets/MEDPARLDSHospitalNational.html

[27] Giuse DA Supporting communication in an integrated patient record system. AMIA Annu Symp Proc. 2003;2003:1065. 14728568 PMC1480157

[28] Giuse NB, Williams AM, Giuse DA Integrating best evidence into patient care: a process facilitated by a seamless integration with informatics tools. J Med Libr Assoc. 2010;98(3):220-222. doi:10.3163/1536-5050.98.3.009 20648255 PMC2901005

[29] Garza M, Del Fiol G, Tenenbaum J, Walden A, Zozus MN Evaluating common data models for use with a longitudinal community registry. J Biomed Inform. 2016;64:333-341. doi:10.1016/j.jbi.2016.10.016 27989817 PMC6810649

[30] Collins GS, Reitsma JB, Altman DG, Moons KG Transparent reporting of a multivariable prediction model for individual prognosis or diagnosis (TRIPOD): the TRIPOD statement. BMJ. 2015;350:g7594. doi:10.1136/bmj.g7594 25569120

[31] Pedersen AB, Mikkelsen EM, Cronin-Fenton D, Missing data and multiple imputation in clinical epidemiological research. Clin Epidemiol. 2017;9:157-166. doi:10.2147/CLEP.S129785 28352203 PMC5358992

[32] Yuan YC Multiple imputation for missing data: concepts and new development. Published 2000 Accessed December 18, 2020. https://connect.ssri.duke.edu/sites/connect.ssri.duke.edu/files/upload/help-resource/multipleimputation%20missing%20data%20-%20sas.pdf

[33] Probst P, Wright MN, Boulesteix AL Hyperparameters and tuning strategies for random forest. WIRES Data: Data Mining and Knowledge Discovery. 2019;9(3):e1301. doi:10.1002/widm.1301

[34] Ridgeway G Generalized boosted models: a guide to the gbm package. Accessed December 18, 2020. https://cran.r-project.org/web/packages/gbm/vignettes/gbm.pdf

[35] Kuhn M Building predictive models in R using the caret package. J Stat Softw. 2008;28(5):1-26. doi:10.18637/jss.v028.i05 27774042

[36] Liaw A, Wiener M Classification and regression by RandomForest. R News. Published November 2001 Accessed December 20, 2020. https://cogns.northwestern.edu/cbmg/LiawAndWiener2002.pdf

[37] Nattino G, Finazzi S, Bertolini G A new calibration test and a reappraisal of the calibration belt for the assessment of prediction models based on dichotomous outcomes. Stat Med. 2014;33(14):2390-2407. doi:10.1002/sim.6100 24497413

[38] Gerds TA, van de Wiel MA Confidence scores for prediction models. Biom J. 2011;53(2):259-274. doi:10.1002/bimj.201000157 21328604

[39] Steyerberg EW, Vickers AJ, Cook NR, Assessing the performance of prediction models: a framework for traditional and novel measures. Epidemiology. 2010;21(1):128-138. doi:10.1097/EDE.0b013e3181c30fb2 20010215 PMC3575184

[40] Meddings J, Reichert H, Smith SN, The impact of disability and social determinants of health on condition-specific readmissions beyond Medicare risk adjustments: a cohort study. J Gen Intern Med. 2017;32(1):71-80. doi:10.1007/s11606-016-3869-x 27848189 PMC5215164

[41] Smith LN, Makam AN, Darden D, Acute myocardial infarction readmission risk prediction models: a systematic review of model performance. Circ Cardiovasc Qual Outcomes. 2018;11(1):e003885. doi:10.1161/CIRCOUTCOMES.117.003885 29321135 PMC5858710

[42] McManus DD, Saczynski JS, Lessard D, ; TRACE-CORE Investigators TRACE-CORE Investigators. Reliability of predicting early hospital readmission after discharge for an acute coronary syndrome using claims-based data. Am J Cardiol. 2016;117(4):501-507. doi:10.1016/j.amjcard.2015.11.034 26718235 PMC4768305

[43] Davis SE, Greevy RA, Fonnesbeck C, Lasko TA, Walsh CG, Matheny ME A nonparametric updating method to correct clinical prediction model drift. J Am Med Inform Assoc. 2019;26(12):1448-1457. doi:10.1093/jamia/ocz127 31397478 PMC6857513

[44] Artetxe A, Beristain A, Graña M Predictive models for hospital readmission risk: a systematic review of methods. Comput Methods Programs Biomed. 2018;164:49-64. doi:10.1016/j.cmpb.2018.06.006 30195431

[45] Hebert C, Shivade C, Foraker R, Diagnosis-specific readmission risk prediction using electronic health data: a retrospective cohort study. BMC Med Inform Decis Mak. 2014;14:65. Published online August 4, 2014. doi:10.1186/1472-6947-14-65 25091637 PMC4136398

[46] Nguyen OK, Makam AN, Clark C, Zhang S, Das SR, Halm EA Predicting 30-day hospital readmissions in acute myocardial infarction: the AMI “READMITS” (renal function, elevated brain natriuretic peptide, age, diabetes mellitus, nonmale sex, intervention with timely percutaneous coronary intervention, and low systolic blood pressure) score. J Am Heart Assoc. 2018;7(8):e008882. doi:10.1161/JAHA.118.008882 29666065 PMC6015397

[47] Burke RE, Schnipper JL, Williams MV, The HOSPITAL score predicts potentially preventable 30-day readmissions in conditions targeted by the hospital readmissions reduction program. Med Care. 2017;55(3):285-290. doi:10.1097/MLR.0000000000000665 27755392 PMC5309170

[48] Dodson JA, Hajduk AM, Murphy TE, Thirty-day readmission risk model for older adults hospitalized with acute myocardial infarction. Circ Cardiovasc Qual Outcomes. 2019;12(5):e005320. doi:10.1161/CIRCOUTCOMES.118.005320 31010300 PMC6481309

[49] Wasfy JH, Singal G, O’Brien C, Enhancing the prediction of 30-day readmission after percutaneous coronary intervention using data extracted by querying of the electronic health record. Circ Cardiovasc Qual Outcomes. 2015;8(5):477-485. doi:10.1161/CIRCOUTCOMES.115.001855 26286871

[50] Minne L, Eslami S, de Keizer N, de Jonge E, de Rooij SE, Abu-Hanna A Effect of changes over time in the performance of a customized SAPS-II model on the quality of care assessment. Intensive Care Med. 2012;38(1):40-46. doi:10.1007/s00134-011-2390-2 22042520 PMC3233667

