# Omission in Funding

**DOI:** 10.1001/jamanetworkopen.2022.4247

**Published:** 2022-03-07

In the Original Investigation titled “Development of Electronic Health Record–Based Prediction Models for 30-Day Readmission Risk Among Patients Hospitalized for Acute Myocardial Infarction,”^1^ published January 29, 2021, there was an omission in the funding statement end matter. The Funding/Support statement should have included the following sentence: “Ms Ricket was supported in part by BD2K T-32 Training Grant (T32 LM012204) from the National Institutes of Health.” This addition does not affect the Role of the Funder/Sponsor information. This article has been corrected.^1^
