# Supplementary material for: Development of Electronic Health Record–Based Prediction Models for 30-Day Readmission Risk Among Patients Hospitalized for Acute Myocardial Infarction
Source: JAMA Netw Open. 2021 Jan 29;4(1):e2035782. doi: 10.1001/jamanetworkopen.2020.35782 (PMC7846941; doi:10.1001/jamanetworkopen.2020.35782)
Supplement: Supplement. — eTable 1. Possible ML Variables eTable 2. Patient Characteristics for 4024 Patients Hospitalized at Dartmouth-Hitchcock Medical Center With a Primary Diagnosis of Acute Myocardial Infarction AMI Cohort eFigure 1. Calibration Curves for Final VUMC Models eFigure 2. Calibration Curves for Final DHMC Models eTable 3. Final Elastic Net Model Parameter Coefficients for VUMC eTable 4. Final LASSO Model Parameter Coefficients for VUMC eTable 5. Final Ridge Regression Model Parameter Coefficients for VUMC eTable 6. Pooled Variable Importance Results of Final Random Forest Model for VUMC eTable 7. Pooled Relative Influence Values from Final Gradient Boosting Model for VUMC eTable 8. Full Variable List Value Set Definitions [file jamanetwopen-e2035782-s001.pdf]

Supplementary Online Content

Matheny ME, Ricket I, Goodrich CA, et al. Development of electronic health record–based prediction models for 30-day readmission risk among patients hospitalized for acute myocardial infarction. *JAMA Netw Open*. 2021;4(1):e2035782. doi:10.1001/jamanetworkopen.2020.35782

eTable 1. Possible ML Variables

eTable 2. Patient Characteristics for 4024 Patients Hospitalized at Dartmouth-Hitchcock Medical Center With a Primary Diagnosis of Acute Myocardial Infarction AMI Cohort

eFigure 1. Calibration Curves for Final VUMC Models

eFigure 2. Calibration Curves for Final DHMC Models

eTable 3. Final Elastic Net Model Parameter Coefficients for VUMC

eTable 4. Final LASSO Model Parameter Coefficients for VUMC

eTable 5. Final Ridge Regression Model Parameter Coefficients for VUMC

eTable 6. Pooled Variable Importance Results of Final Random Forest Model for VUMC

eTable 7. Pooled Relative Influence Values from Final Gradient Boosting Model for VUMC

eTable 8. Full Variable List Value Set Definitions

This supplementary material has been provided by the authors to give readers additional information about their work.

© 2021 Matheny ME et al. *JAMA Network Open.*

eTable 1. Possible ML Variables

**Demographics**	Race, age, gender, ethnicity
**Comorbidities**	Charlson Deyo Comorbidity Score; Comorbid conditions that include age>80, arrhythmia, anemia, hypertension, chronic obstructive pulmonary disease, chronic kidney disease, cerebrovascular accident/stroke, tobacco use, depression status, hypercholesterolemia, coronary artery disease, diabetes, congestive heart failure, prior myocardial infarction, peripheral vascular disease, cerebrovascular disease, dementia, chronic pulmonary disease, rheumatologic disease, peptic ulcer, mild liver disease, hemiplegia or paraplegia, renal disease, AIDS
**Laboratories**	Sodium (<136 mEq/L), calcium (<8.6mg/dL), troponin, hemoglobin, blood urea nitrogen and creatinine
**Discharge Information**	Anti-depressant on discharge; aspirin, beta blocker, ACE or ARB inhibitors combined at discharge; unstable angina, NSTEMI or STEMI; transfer to other hospital at discharge; LVEF diagnosis for index admission
**Presentation/ Disease**	Transfer patient, chest pain, cardiac arrest, AMI location
**Administrative Data**	Length of index hospitalization stay, number of other admissions within 30 days prior to the index admission, number of other admissions within 1-year prior to index admission
**Patient History**	History of: chest pain, myocardial infarction, CABG, PCI, peripheral vascular disease, angina, unstable angina, hypertension, depression, number of major depressive episodes within the prior year
**Prior Month Diagnosis**	Sepsis, hyperkalemia, hypokalemia, acute kidney failure
**Prior 3 Month Diagnosis**	Sepsis, disorders of magnesium metabolism, hypokalemia, acute kidney failure
**In-Hospital Outcomes**	In-hospital new heart failure, recurrent ischemia in hospital; cardiac surgery; post-MI CABG
**Acute Kidney Injury**	AKI Stage, AKI_Flag
**HOSPITAL Score Modified**	Overall HOSPITAL score; low hemoglobin level at discharge (<12 g/dL), low sodium level at discharge (<135 mEq/L), number of hospital admissions during the previous year, admission type: non-elective, length of stay > 5 days

© 2021 Matheny ME et al. *JAMA Network Open.*

**GRACE Score**<br><br>**Modified**	Overall GRACE score; in-hospital PCI, troponin, age, initial serum creatinine (mg/dL), cardiac arrest, STEMI
**LACE Score**<br><br>**Modified**	Overall LACE score; length of stay, Charlson Deyo score (comorbidities that include previous MI, cerebrovascular disease, peripheral vascular disease, diabetes with and without complications, CHF, chronic pulmonary disease, liver or renal disease, tumor including lymphoma/leukemia, dementia, connective tissue damage, AIDS, liver/renal disease, metastatic solid tumor)

© 2021 Matheny ME et al. *JAMA Network Open.*

eTable 2. Patient characteristics for 4024 patients hospitalized at Dartmouth-Hitchcock Medical Center with a primary diagnosis of acute myocardial infarction AMI cohort

	**Readmission (%) (N=412)**	**Non-readmission (%) (N=3612)**
**Gender**		
Male	60.2 (N=248)	64.7 (N=2336)
Female	39.8 (N=164)	35.13 (N=1276)
**Co-morbidities**		
STEMI	27.7 (N=114)	36.7 (N=1327)
Heart Failure during hospitalization	19.7 (N=81)	9.7 (N=350)
Ischemia during hospitalization	3.2 (N=13)	2.3 (N= 83)
**Discharge Location**		
Home*	74.3 (N=306)	84.5 (N=3052)
Health facility*	25.7 (N=106)	15.5 (N=560)
**Mean Continuous Scores**		
Age (years)	74.0 (SD=11.8)	66.4 (SD=13.2)
LACE score	4.19 (SD=1.43)	3.75 (SD=1.33)
GRACE score	82.25 (SD=22.79)	72.44 (SD=24.33)
HOSPITAL score	0.78 (SD=1.04)	1.30 (SD=1.26)
Length of stay	5.42 (SD=6.18)	6.71 (SD=5.55)

© 2021 Matheny ME et al. *JAMA Network Open.*

eFigure 1: Calibration Curves for Final VUMC Models

Training Set	Test Set

Elastic Net

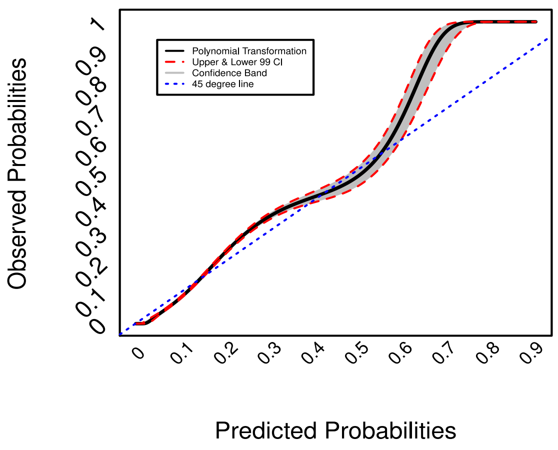

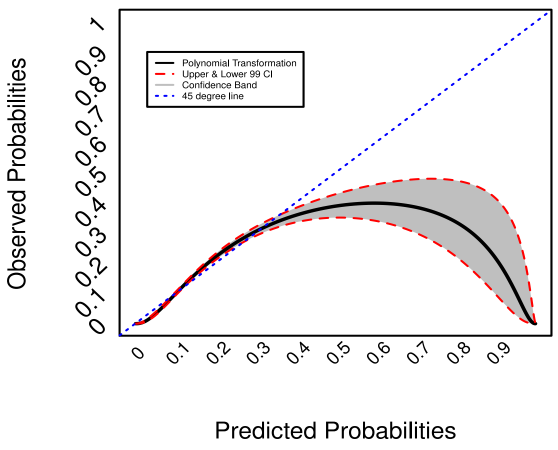

Gradient Boosting

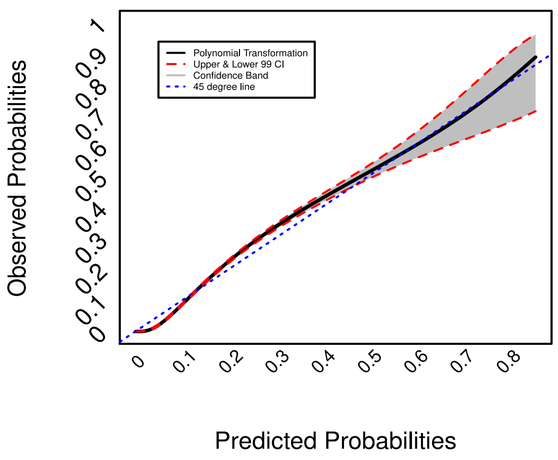

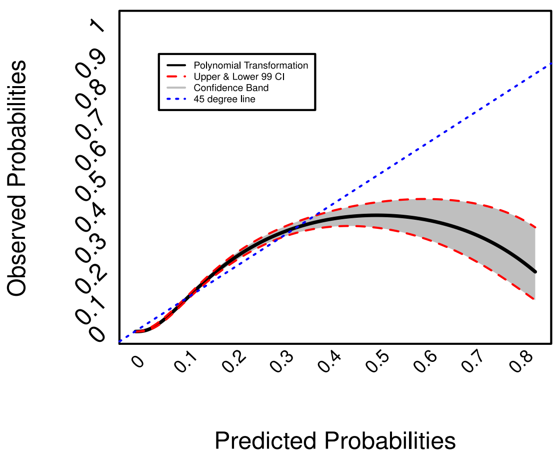

LASSO

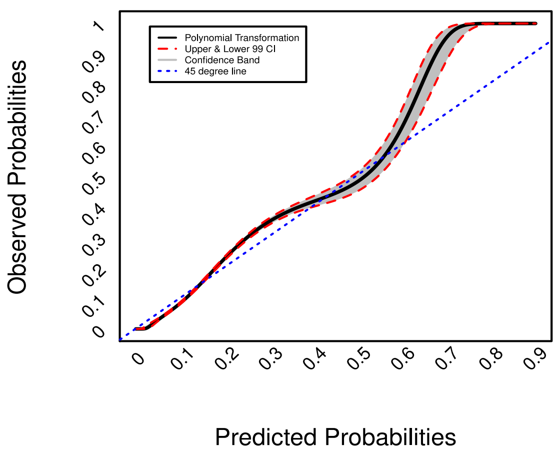

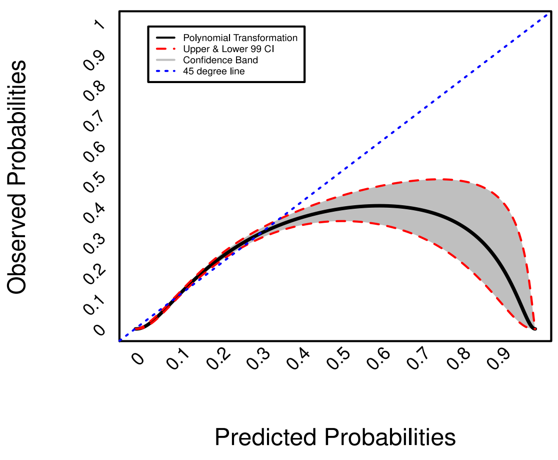

© 2021 Matheny ME et al. *JAMA Network Open.*

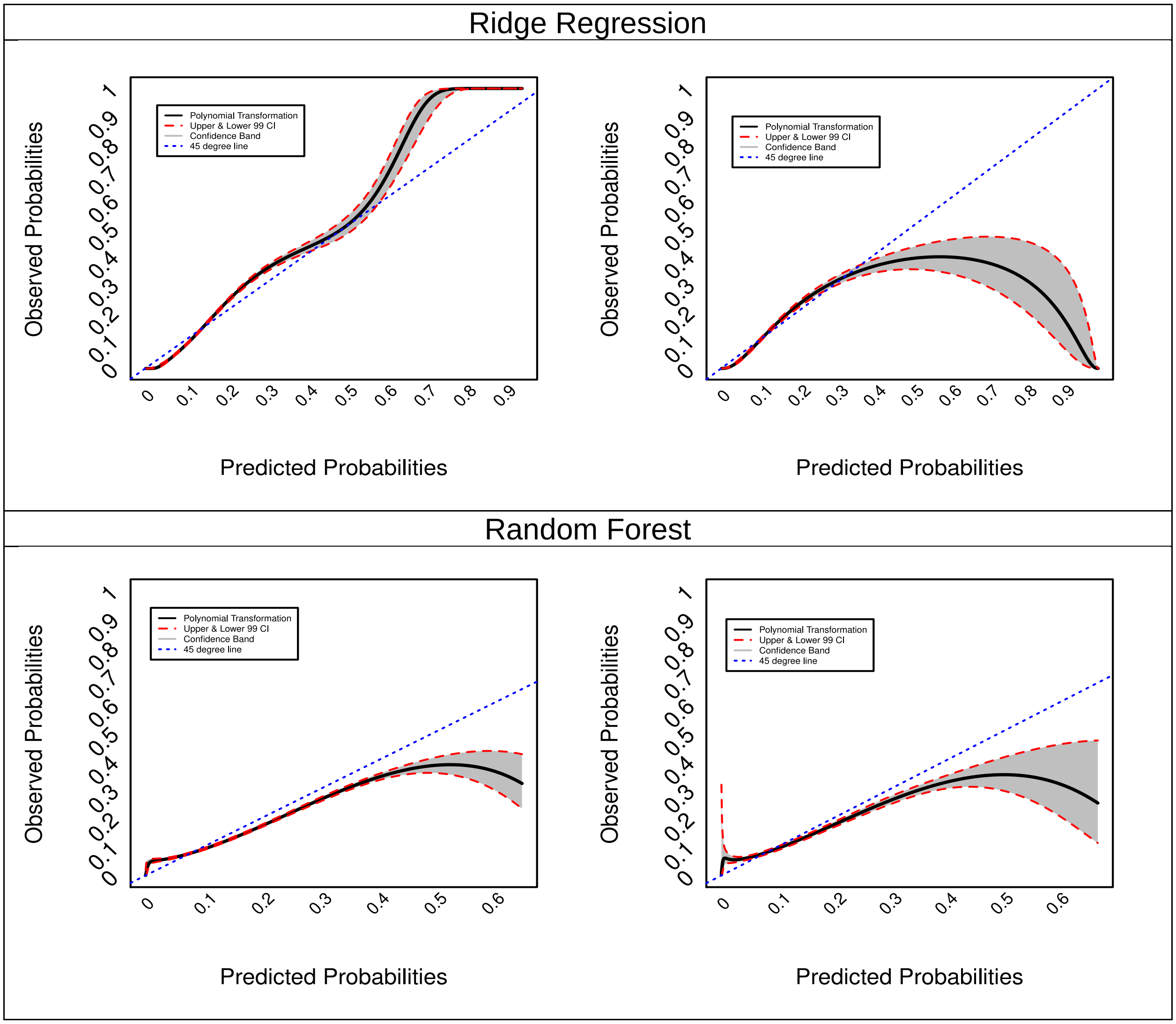

© 2021 Matheny ME et al. *JAMA Network Open*.

eFigure 2: Calibration Curves for Final Scored DHMC Models

Elastic Net

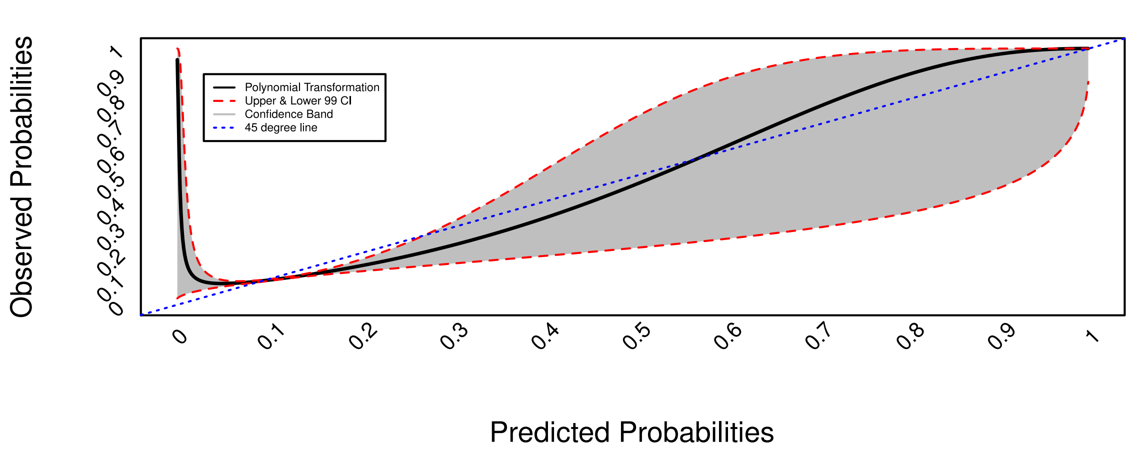

Gradient Boosting

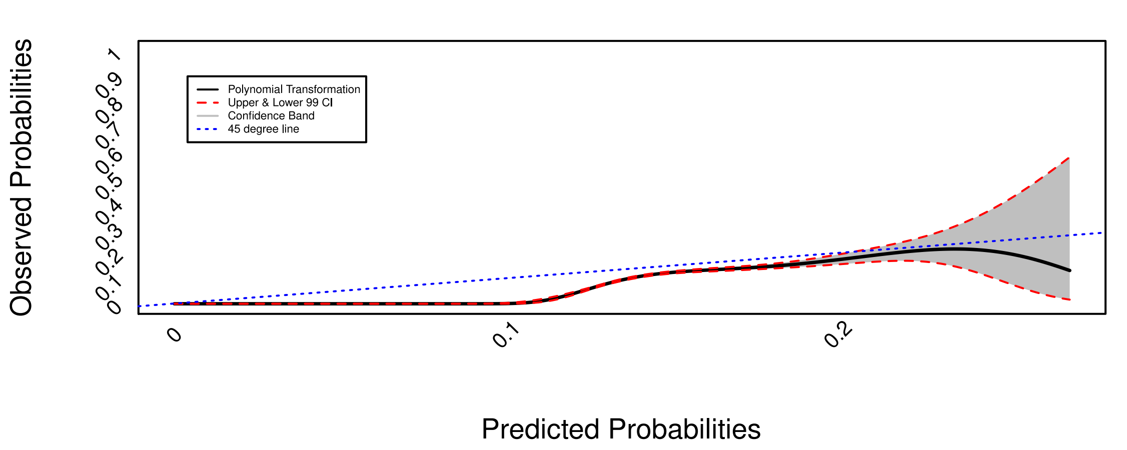

LASSO

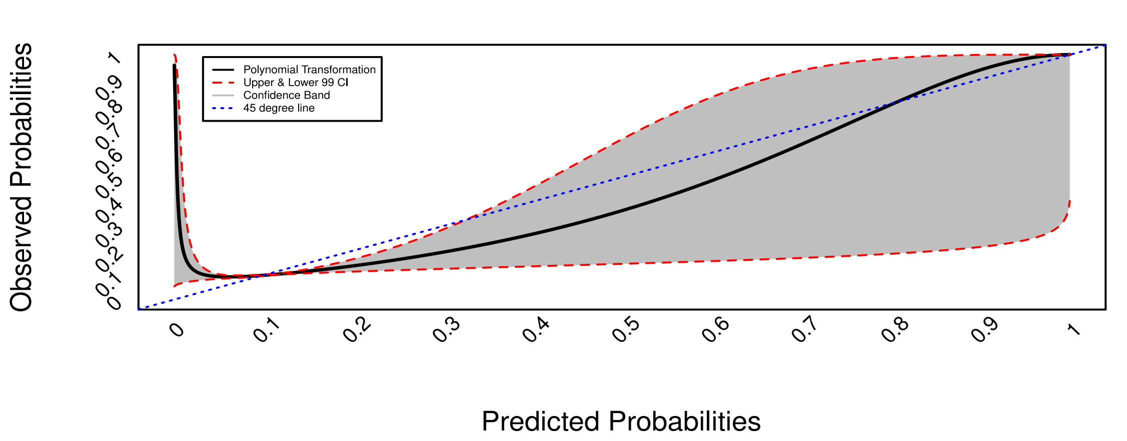

Ridge Regression

© 2021 Matheny ME et al. *JAMA Network Open.*

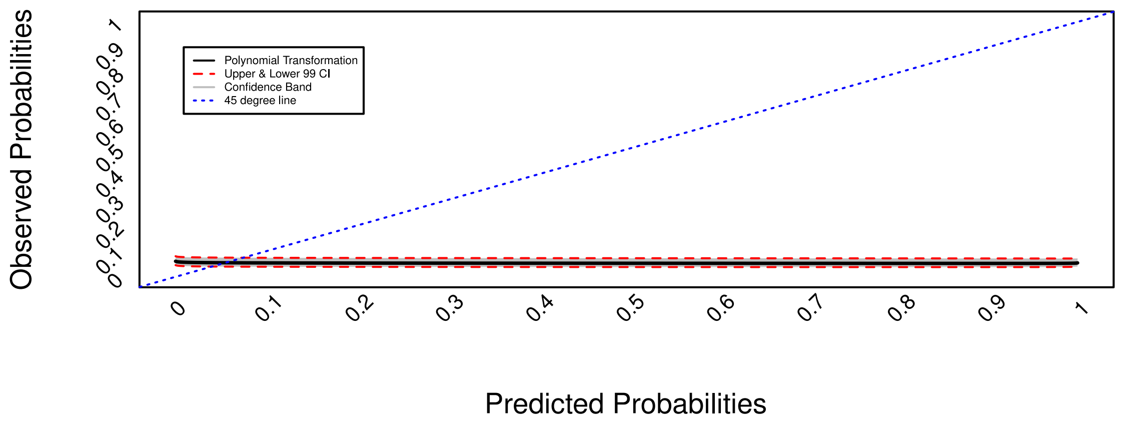

Random Forest

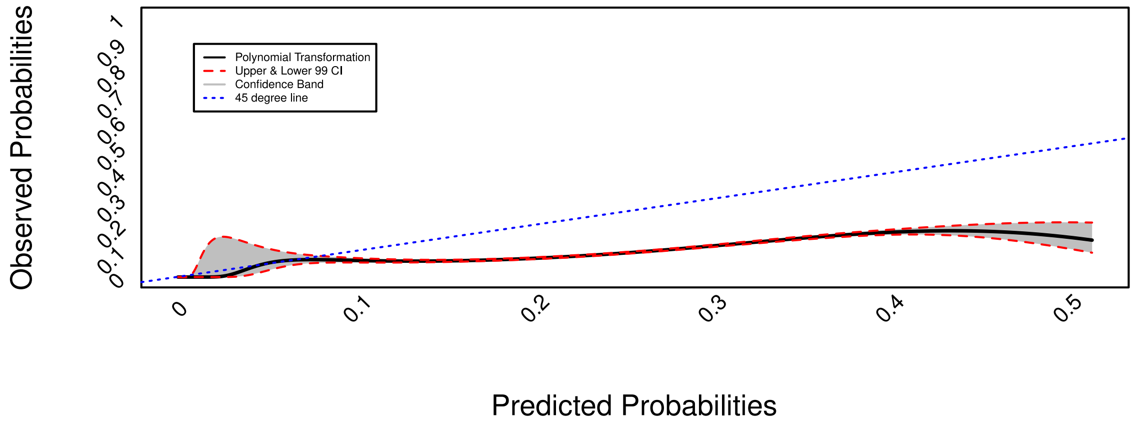

© 2021 Matheny ME et al. *JAMA Network Open.*

eTable 3: Final Elastic Net Model Parameter Coefficients for VUMC

Variable Name	Pooled Coefficient
(Intercept)	-1.794
genderMALE	-0.002
raceAsian	-0.005
raceNo matching concept	-0.012
ethnicityNo matching concept	-0.055
ethnicityNot Hispanic or Latino	0.041
age_at_admit	0.003
prim_diag410.11	0.002
prim_diag410.41	-0.002
prim_diag410.51	0.033
prim_diag410.61	-0.003
prim_diag410.71	0.008
prim_diag410.81	0.003
prim_diag410.91	-0.006
prim_diagI21.09	0.003
prim_diagI21.11	-0.022
prim_diagI21.29	-0.002
prim_diagI21.3	0.025
prim_diagI21.4	-0.053
prior_sepsis_30d	-0.005
prior_hyperkalemia_30d	0.118
prior_hypokalemia_30d	0.007
prior_hypervolemia_30d	-0.020
prior_sepsis_90d	0.001
prior_dis_magn_metab_90d	0.106
prior_hypokalemia_90d	0.041
prior_akf_90d	0.055
los5_flag	0.244
prior_year_admissions_count	0.059
oncology_flag	0.018
hemoglobin_level_last_12_flag	0.006
hospital_score	0.006
chest_pain_flag	0.023
ami_locationAnterior wall	0.001
ami_locationOther inferior wall	-0.009
ami_locationOther lateral wall	0.024
ami_locationUnspecified site	-0.001

© 2021 Matheny ME et al. *JAMA Network Open.*

admission_prior_30_days_count	0.008
unstable_angina_flag	-0.093
nstemi_flag	0.007
discharge_locationFederally Qualified Health Center	-0.341
discharge_locationHome	-0.209
discharge_locationHOME OR SELF CARE W/ PLANNED READMISSION	3.369
discharge_locationHospice	-0.915
discharge_locationInpatient Long-term Care	-0.004
discharge_locationInpatient Psychiatric Facility	0.123
discharge_locationINTERMED.CARE FACILITY	0.075
discharge_locationOther Place of Service	0.057
discharge_locationPrison/Correctional Facility	0.188
discharge_locationSkilled Nursing Facility	0.010
discharge_locationSWING BED	0.754
discharge_locationTRANSFER OTHER HOSPITAL	-0.005
history_chest_pain_flag	0.001
history_ami_flag	-0.004
history_pvd_flag	0.019
history_angina_flag	0.070
history_unstable_angina_flag	0.004
major_depression_count	0.009
in_hospital_hf_flag	0.125
age_80_flag	0.095
comorbid_arrhythmia_flag	0.047
comorbid_anemia_flag	0.065
comorbid_copd_flag	0.002
comorbid_stroke_flag	-0.003
comorbid_tobacco_use_flag	0.001
comorbid_depression_flag	0.003
comorbid_hypercholesterolemia_f	-0.002
comorbid_cad_flag	-0.082
comorbid_diabetes_cc_flag	-0.013
comorbid_chf_flag	0.035
comorbid_mi_flag	-0.011
comorbid_peripheral_vascular_di	0.048
comorbid_cerebrovascular_diseas	-0.004
comorbid_chronic_pulmonary_dise	0.011
comorbid_peptic_ulcer_disease_f	0.004
comorbid_mild_liver_disease_fla	0.060

© 2021 Matheny ME et al. *JAMA Network Open.*

comorbid_hemiplegia_or_parapleg	-0.121
comorbid_moderate_or_severe_liv	0.010
comorbid_aids_flag	0.084
comorbid_diabetes_cc_flag_score	-0.004
comorbid_chf_flag_score	0.031
comorbid_mi_flag_score	-0.009
comorbid_peripheral_vascular_disease_flag_score	0.043
comorbid_cerebrovascular_disease_flag_score	-0.003
comorbid_chronic_pulmonary_disease_flag_score	0.010
comorbid_peptic_ulcer_disease_flag_score	0.002
comorbid_mild_liver_disease_flag_score	0.045
comorbid_hemiplegia_or_paraplegia_flag_score	-0.046
comorbid_moderate_or_severe_liver_disease_flag_score	0.003
comorbid_aids_flag_score	0.012
lace_los_score	0.052
lace_score	0.029
lvef_flag	-0.025
post_mi_cabg_flag	0.200
history_stroke_flag	0.015
grace_score_age	0.006
grace_score_creatinine_level_fi	0.012
disch_med_bb_method	-0.088
disch_med_antidep_method	-0.004
disch_med_ace_arb_flag	-0.070
disch_med_ace_arb_method	-0.066
disch_med_aspirin_method	-0.006
disch_med_statin_flag	-0.013
disch_med_statin_method	-0.033
aki_flag	0.211
aki_stage	0.034
aki_stage_2_flag	0.017
aki_stage_3_flag	0.004
sodium_level_avg	-0.002
sodium_level_first	-0.002
sodium_level_min	-0.001
calcium_level_avg	0.039
calcium_level_max	-0.033
calcium_level_first	-0.007
calcium_level_last	0.000

© 2021 Matheny ME et al. *JAMA Network Open.*

creatinine_level_avg	0.004
creatinine_level_min	0.017
creatinine_level_max	0.001
creatinine_level_first	0.009
creatinine_level_last	0.005
hemoglobin_level_avg	-0.029
hemoglobin_level_max	-0.003
hemoglobin_level_first	-0.012
hemoglobin_level_last	-0.003
bnp_first_cat	0.003
i_bnp_ckd	0.016
i_bnp_dementia	-0.031
i_bnp_stroke	-0.037
i_bnp_med_ace	-0.085
i_bnp_hospital	0.004
i_bnp_inhosp_ischemia	0.001
i_bnp_lace	0.002
i_bnp_dis_metab_90d	0.064
i_bnp_prior_yr_count	0.007
i_cardiac_a_inhosp_ischemia	-0.073
i_cardiac_a_lace	-0.005
i_cardiac_a_oncology	0.146
i_ckd_dementia	0.086
i_ckd_lace	0.004
i_ckd_prior_yr_count	0.022
i_dementia_med_ace	1.910
i_dementia_pvd	-0.004
i_dementia_hospital	-0.007
i_dementia_dis_metab_90d	-0.436
i_dementia_prior_yr_count	-0.053
i_med_ace_med_antidep	0.342
i_med_ace_hospital	-0.020
i_med_ace_inhosp_ischemia	-0.005
i_med_ace_oncology	0.074
i_med_ace_prior_yr_count	0.110
i_med_antidep_hospital	-0.004
i_pvd_lace	0.001
i_inhosp_ischemia_oncology	0.126
i_inhosp_ischemia_prior_yr_count	-0.011

© 2021 Matheny ME et al. *JAMA Network Open.*

i_lace_oncology	0.021
i_lace_dis_metab_90d	0.007
i_dis_metab90d_prior_yr_count	0.006

If pooled coefficients were 0 when rounded to 3 decimals, it was removed from the list

© 2021 Matheny ME et al. *JAMA Network Open.*

eTable 4: Final LASSO Model Parameter Coefficients for VUMC

Variable Name	Pooled Coefficient
(Intercept)	-1.714
genderMALE	-0.001
raceAsian	-0.003
raceNo matching concept	-0.011
ethnicityNo matching concept	-0.056
ethnicityNot Hispanic or Latino	0.039
age_at_admit	0.001
prim_diag410.11	0.002
prim_diag410.51	0.045
prim_diag410.71	0.006
prim_diag410.81	0.003
prim_diag410.91	-0.005
prim_diagI21.09	0.000
prim_diagI21.11	-0.017
prim_diagI21.3	0.019
prim_diagI21.4	-0.055
prior_sepsis_30d	-0.004
prior_hyperkalemia_30d	0.113
prior_hypokalemia_30d	0.005
prior_hypervolemia_30d	-0.015
prior_sepsis_90d	0.001
prior_dis_magn_metab_90d	0.106
prior_hypokalemia_90d	0.035
prior_akf_90d	0.052
los5_flag	0.260
prior_year_admissions_count	0.065
oncology_flag	0.019
hemoglobin_level_last_12_flag	0.004
hospital_score	0.003
calcium_level_avg_86_flag	0.001
chest_pain_flag	0.022
ami_locationOther inferior wall	-0.009
ami_locationOther lateral wall	0.002
ami_locationOther specified sites	0.002
admission_prior_30_days_count	0.008

© 2021 Matheny ME et al. *JAMA Network Open.*

unstable_angina_flag	-0.094
stemi_flag	0.000
nstemi_flag	0.005
discharge_locationFederally Qualified Health Center	-0.337
discharge_locationHome	-0.211
discharge_locationHOME OR SELF CARE W/ PLANNED READMISSION	3.427
discharge_locationHospice	-0.939
discharge_locationInpatient Long-term Care	-0.002
discharge_locationInpatient Psychiatric Facility	0.130
discharge_locationINTERMED.CARE FACILITY	0.068
discharge_locationOther Place of Service	0.039
discharge_locationPrison/Correctional Facility	0.182
discharge_locationSkilled Nursing Facility	0.008
discharge_locationSWING BED	0.727
discharge_locationTRANSFER OTHER HOSPITAL	-0.002
history_chest_pain_flag	0.001
history_ami_flag	-0.003
history_pvd_flag	0.018
history_angina_flag	0.067
history_unstable_angina_flag	0.004
major_depression_count	0.008
in_hospital_hf_flag	0.126
age_80_flag	0.083
comorbid_arrhythmia_flag	0.044
comorbid_anemia_flag	0.063
comorbid_hypertension_flag	0.000
comorbid_copd_flag	0.002
comorbid_tobacco_use_flag	0.001
comorbid_depression_flag	0.003
comorbid_hypercholesterolemia_f	-0.002
comorbid_cad_flag	-0.083
comorbid_diabetes_cc_flag	-0.019
comorbid_chf_flag	0.060
comorbid_mi_flag	-0.014
comorbid_peripheral_vascular_di	0.085
comorbid_cerebrovascular_diseas	-0.007
comorbid_chronic_pulmonary_dise	0.016
comorbid_rheumatologic_disease_	0.000
comorbid_peptic_ulcer_disease_f	0.006

© 2021 Matheny ME et al. *JAMA Network Open.*

comorbid_mild_liver_disease_fla	0.095
comorbid_hemiplegia_or_parapleg	-0.211
comorbid_moderate_or_severe_liv	0.018
comorbid_aids_flag	0.142
comorbid_diabetes_cc_flag_score	-0.001
comorbid_chf_flag_score	0.003
comorbid_mi_flag_score	-0.005
comorbid_peripheral_vascular_disease_flag_score	0.004
comorbid_peptic_ulcer_disease_flag_score	0.000
comorbid_mild_liver_disease_flag_score	0.006
comorbid_hemiplegia_or_paraplegia_flag_score	-0.001
lace_los_score	0.049
lace_charlson_score	0.000
lace_score	0.031
lvef_flag	-0.024
post_mi_cabg_flag	0.199
history_stroke_flag	0.014
grace_score_age	0.008
grace_score_creatinine_level_fi	0.013
disch_med_bb_method	-0.091
disch_med_antidep_method	-0.005
disch_med_ace_arb_flag	-0.057
disch_med_ace_arb_method	-0.067
disch_med_aspirin_method	-0.005
disch_med_statin_flag	-0.012
disch_med_statin_method	-0.032
aki_flag	0.224
aki_stage	0.029
aki_stage_2_flag	0.014
aki_stage_3_flag	0.006
sodium_level_avg	-0.002
sodium_level_first	-0.002
sodium_level_min	-0.001
calcium_level_avg	0.041
calcium_level_min	0.000
calcium_level_max	-0.034
calcium_level_first	-0.007
creatinine_level_avg	0.003
creatinine_level_min	0.017

© 2021 Matheny ME et al. *JAMA Network Open.*

creatinine_level_max	0.000
creatinine_level_first	0.009
creatinine_level_last	0.004
hemoglobin_level_avg	-0.032
hemoglobin_level_max	-0.004
hemoglobin_level_first	-0.011
hemoglobin_level_last	-0.002
bnp_first_cat	0.003
i_bnp_ckd	0.016
i_bnp_dementia	-0.033
i_bnp_stroke	-0.037
i_bnp_med_ace	-0.095
i_bnp_hospital	0.004
i_bnp_inhosp_ischemia	0.001
i_bnp_lace	0.002
i_bnp_dis_metab_90d	0.061
i_bnp_prior_yr_count	0.007
i_cardiac_a_inhosp_ischemia	-0.071
i_cardiac_a_lace	-0.004
i_cardiac_a_oncology	0.145
i_ckd_dementia	0.081
i_ckd_lace	0.003
i_ckd_prior_yr_count	0.020
i_dementia_med_ace	1.862
i_dementia_pvd	-0.003
i_dementia_hospital	-0.005
i_dementia_dis_metab_90d	-0.428
i_dementia_prior_yr_count	-0.054
i_med_ace_med_antidep	0.309
i_med_ace_hospital	-0.017
i_med_ace_inhosp_ischemia	-0.004
i_med_ace_oncology	0.065
i_med_ace_prior_yr_count	0.119
i_med_antidep_hospital	-0.004
i_pvd_lace	0.001
i_inhosp_ischemia_oncology	0.122
i_inhosp_ischemia_prior_yr_count	-0.012
i_lace_oncology	0.021
i_lace_dis_metab_90d	0.006

© 2021 Matheny ME et al. *JAMA Network Open.*

i_dis_metab90d_prior_yr_count	0.004

If pooled coefficients were 0 when rounded to 3 decimals, it was removed from the list

© 2021 Matheny ME et al. *JAMA Network Open.*

eTable 5: Final Ridge Regression Model Parameter Coefficients for VUMC

Variable Name	Pooled Coefficient
(Intercept)	-1.809
genderMALE	-0.016
genderNo matching concept	-0.191
raceAsian	-0.092
raceBlack	0.012
raceNo matching concept	-0.036
raceWhite	0.012
ethnicityNo matching concept	-0.064
ethnicityNot Hispanic or Latino	0.061
age_at_admit	0.003
prim_diag410.1	-0.434
prim_diag410.11	0.015
prim_diag410.21	-0.005
prim_diag410.31	0.018
prim_diag410.32	-0.262
prim_diag410.4	-0.331
prim_diag410.41	-0.025
prim_diag410.51	0.130
prim_diag410.61	-0.027
prim_diag410.7	-0.344
prim_diag410.71	0.024
prim_diag410.72	-0.310
prim_diag410.81	0.061
prim_diag410.9	-0.207
prim_diag410.91	-0.065
prim_diagI21.09	0.077
prim_diagI21.11	-0.322
prim_diagI21.19	0.013
prim_diagI21.29	-0.073
prim_diagI21.3	0.336
prim_diagI21.4	-0.052
prior_sepsis_30d	-0.072
prior_hyperkalemia_30d	0.182
prior_hypokalemia_30d	0.058
prior_hypervolemia_30d	-0.166
prior_akf_30d	-0.049

© 2021 Matheny ME et al. *JAMA Network Open.*

prior_sepsis_90d	0.037
prior_dis_magn_metab_90d	0.150
prior_hypokalemia_90d	0.080
prior_akf_90d	0.109
los	0.002
los5_flag	0.091
prior_year_admissions_count	0.023
oncology_flag	0.053
hemoglobin_level_last_12_flag	0.031
sodium_level_last_135_flag	-0.021
hospital_score	0.015
calcium_level_avg_86_flag	0.030
chest_pain_flag	0.037
cardiac_arrest_flag	0.005
ami_locationAnterior wall	0.015
ami_locationAnterolateral wall	0.012
ami_locationInferolateral wall	0.002
ami_locationInferoposterior wall	-0.016
ami_locationOther anterior wall	0.058
ami_locationOther inferior wall	-0.025
ami_locationOther lateral wall	0.130
ami_locationOther specified sites	0.028
ami_locationSubendocardial infarction	0.003
ami_locationTrue posterior wall infarction initial episode of care	-0.027
ami_locationUnspecified site	-0.047
index_los	0.002
admission_prior_30_days_count	0.056
unstable_angina_flag	-0.070
stemi_flag	-0.019
nstemi_flag	0.032
discharge_locationFederally Qualified Health Center	-0.359
discharge_locationHome	-0.123
discharge_locationHOME OR SELF CARE W/ PLANNED READMISSION	2.183
discharge_locationHOME-HEALTH CARE SVC W/ PLANNED READMISSION	-0.459
discharge_locationHospice	-0.468
discharge_locationInpatient Long-term Care	-0.096
discharge_locationInpatient Psychiatric Facility	0.382
discharge_locationINTERMED.CARE FACILITY	0.368
discharge_locationOther Place of Service	0.362

© 2021 Matheny ME et al. *JAMA Network Open.*

discharge_locationPatient self-discharge against medical advice	-0.017
discharge_locationPrison/Correctional Facility	0.429
discharge_locationSkilled Nursing Facility	0.083
discharge_locationSWING BED	0.912
discharge_locationTRANSFER OTHER HOSPITAL	-0.005
rehab_flag	0.000
history_chest_pain_flag	0.014
history_ami_flag	-0.028
history_pvd_flag	0.046
history_angina_flag	0.065
history_unstable_angina_flag	0.028
history_hypertension_flag	0.009
history_depression_flag	-0.019
major_depression_count	0.015
in_hospital_hf_flag	0.064
in_hospital_ischemia_flag	0.028
age_80_flag	0.107
comorbid_arrhythmia_flag	0.065
comorbid_anemia_flag	0.066
comorbid_hypertension_flag	0.002
comorbid_copd_flag	0.013
comorbid_ckd_flag	0.039
comorbid_stroke_flag	-0.043
comorbid_tobacco_use_flag	0.019
comorbid_depression_flag	0.037
comorbid_hypercholesterolemia_f	-0.023
comorbid_cad_flag	-0.093
comorbid_diabetes_cc_flag	-0.046
comorbid_diabetes_flag	-0.024
comorbid_chf_flag	0.048
comorbid_mi_flag	-0.034
comorbid_peripheral_vascular_di	0.060
comorbid_cerebrovascular_diseas	-0.023
comorbid_dementia_flag	0.000
comorbid_chronic_pulmonary_dise	0.031
comorbid_rheumatologic_disease_	-0.027
comorbid_peptic_ulcer_disease_f	0.003
comorbid_mild_liver_disease_fla	0.121
comorbid_hemiplegia_or_parapleg	-0.218

© 2021 Matheny ME et al. *JAMA Network Open.*

comorbid_moderate_or_severe_liv	-0.015
comorbid_aids_flag	0.152
comorbid_diabetes_cc_flag_score	-0.023
comorbid_diabetes_flag_score	-0.024
comorbid_chf_flag_score	0.048
comorbid_mi_flag_score	-0.034
comorbid_peripheral_vascular_disease_flag_score	0.060
comorbid_cerebrovascular_disease_flag_score	-0.023
comorbid_dementia_flag_score	-0.001
comorbid_chronic_pulmonary_disease_flag_score	0.031
comorbid_rheumatologic_disease_flag_score	-0.027
comorbid_peptic_ulcer_disease_flag_score	0.003
comorbid_mild_liver_disease_flag_score	0.121
comorbid_hemiplegia_or_paraplegia_flag_score	-0.109
comorbid_moderate_or_severe_liver_disease_flag_score	-0.005
comorbid_aids_flag_score	0.025
lace_los_score	0.028
lace_charlson_score	0.001
lace_score	0.014
lvef_flag	-0.120
post_mi_cabg_flag	0.164
chf_flag	0.049
history_stroke_flag	0.076
grace_score_age	0.002
grace_score_creatinine_level_fi	0.005
grace_score_cardiac_arrest	0.000
grace_score_stemi	-0.001
grace_score	0.001
disch_med_bb_flag	-0.042
disch_med_bb_method	-0.046
disch_med_antidep_flag	-0.067
disch_med_antidep_method	-0.081
disch_med_ace_arb_flag	-0.104
disch_med_ace_arb_method	-0.052
disch_med_aspirin_flag	0.007
disch_med_aspirin_method	-0.027
disch_med_statin_flag	-0.076
disch_med_statin_method	-0.032
aki_flag	0.107

© 2021 Matheny ME et al. *JAMA Network Open.*

aki_stage	0.048
aki_stage_1_flag	0.110
aki_stage_2_flag	0.100
aki_stage_3_flag	0.101
sodium_level_avg	-0.002
sodium_level_first	-0.001
sodium_level_min	-0.001
sodium_level_max	0.001
sodium_level_last	-0.001
calcium_level_avg	0.022
calcium_level_min	0.005
calcium_level_max	-0.025
calcium_level_first	-0.013
calcium_level_last	-0.005
creatinine_level_avg	0.012
creatinine_level_min	0.016
creatinine_level_max	0.004
creatinine_level_first	0.007
creatinine_level_last	0.011
hemoglobin_level_min	-0.002
hemoglobin_level_avg	-0.012
hemoglobin_level_max	-0.005
hemoglobin_level_first	-0.010
hemoglobin_level_last	-0.008
bnp_level_avg	0.000
quad_hospital_score	0.001
quad_lace_score	0.001
i_bnp_cardiac_a	-0.006
i_bnp_ckd	0.017
i_bnp_dementia	-0.039
i_bnp_stroke	-0.039
i_bnp_med_ace	-0.053
i_bnp_med_antidep	0.018
i_bnp_pvd	-0.001
i_bnp_hospital	0.004
i_bnp_inhosp_ischemia	0.008
i_bnp_lace	0.003
i_bnp_oncology	0.002
i_bnp_dis_metab_90d	0.064

© 2021 Matheny ME et al. *JAMA Network Open.*

i_bnp_prior_yr_count	0.008
i_cardiac_a_med_ace	-0.212
i_cardiac_a_med_antidep	-0.378
i_cardiac_a_hospital	-0.001
i_cardiac_a_inhosp_ischemia	-0.203
i_cardiac_a_lace	-0.011
i_cardiac_a_oncology	0.280
i_cardiac_a_quad_grace	0.000
i_ckd_dementia	0.279
i_ckd_pvd	0.005
i_ckd_hospital	-0.006
i_ckd_lace	0.006
i_ckd_prior_yr_count	0.026
i_dementia_med_ace	1.763
i_dementia_pvd	-0.068
i_dementia_hospital	-0.021
i_dementia_lace	0.001
i_dementia_dis_metab_90d	-1.058
i_dementia_prior_yr_count	-0.071
i_stroke_pvd	0.002
i_stroke_lace	0.000
i_stroke_prior_yr_count	-0.007
i_stroke_quad_grace	0.000
i_med_ace_med_antidep	0.673
i_med_ace_hospital	-0.037
i_med_ace_inhosp_ischemia	-0.276
i_med_ace_lace	-0.014
i_med_ace_oncology	0.410
i_med_ace_prior_yr_count	0.217
i_med_antidep_hospital	-0.005
i_med_antidep_lace	-0.009
i_pvd_hospital	-0.003
i_pvd_lace	0.004
i_pvd_prior_yr_count	0.009
i_hospital_ischemia	-0.002
i_hospital_lace	0.001
i_hospital_oncology	0.006
i_hospital_dis_metab_90d	0.007
i_hospital_prior_yr_count	0.001

© 2021 Matheny ME et al. *JAMA Network Open.*

i_inhosp_ischemia_lace	0.001
i_inhosp_ischemia_oncology	0.190
i_inhosp_ischemia_prior_yr_count	-0.040
i_lace_oncology	0.011
i_lace_dis_metab_90d	0.018
i_lace_prior_yr_count	0.002
i_oncology_prior_yr_count	-0.029
i_dis_metab90d_prior_yr_count	0.034

If pooled coefficients were 0 when rounded to 3 decimals, it was removed from the list

© 2021 Matheny ME et al. *JAMA Network Open.*

eTable 6: Pooled Variable Importance Results of Final Random Forest Model for VUMC

Variable	Pooled Feature Importance
gender	5.722
race	7.226
ethnicity	3.501
age_at_admit	35.210
prim_diag	23.674
prior_sepsis_30d	1.855
prior_hyperkalemia_30d	0.836
prior_hypokalemia_30d	0.691
prior_hypervolemia_30d	0.510
prior_akf_30d	0.990
prior_sepsis_90d	2.664
prior_dis_magn_metab_90d	0.773
prior_hypokalemia_90d	1.155
prior_akf_90d	1.977
los	21.366
los5_flag	5.312
prior_year_admissions_count	7.969
oncology_flag	3.235
hemoglobin_level_last_12_flag	4.580
sodium_level_last_135_flag	3.740
hospital_score	15.973
calcium_level_avg_86_flag	4.418
chest_pain_flag	6.054
cardiac_arrest_flag	2.425
ami_location	16.564
index_los	21.362
admission_prior_30_days_count	2.689
unstable_angina_flag	6.465
stemi_flag	5.317
nstemi_flag	4.824
discharge_location	19.131
history_chest_pain_flag	5.430
history_ami_flag	4.212
history_pvd_flag	4.445
history_angina_flag	4.325
history_unstable_angina_flag	4.729
history_hypertension_flag	5.482

© 2021 Matheny ME et al. *JAMA Network Open.*

history_depression_flag	3.444
major_depression_count	7.715
in_hospital_hf_flag	6.499
in_hospital_ischemia_flag	5.116
age_80_flag	5.257
comorbid_arrhythmia_flag	4.894
comorbid_anemia_flag	4.056
comorbid_hypertension_flag	4.884
comorbid_copd_flag	1.765
comorbid_ckd_flag	4.051
comorbid_stroke_flag	1.030
comorbid_tobacco_use_flag	2.309
comorbid_depression_flag	2.236
comorbid_hypercholesterolemia_f	2.340
comorbid_cad_flag	2.804
comorbid_diabetes_cc_flag	1.946
comorbid_diabetes_flag	3.029
comorbid_chf_flag	4.261
comorbid_mi_flag	2.979
comorbid_peripheral_vascular_di	3.078
comorbid_cerebrovascular_diseas	2.345
comorbid_dementia_flag	1.240
comorbid_chronic_pulmonary_dise	2.795
comorbid_rheumatologic_disease_	1.108
comorbid_peptic_ulcer_disease_f	0.783
comorbid_mild_liver_disease_fla	1.018
comorbid_hemiplegia_or_parapleg	0.198
comorbid_moderate_or_severe_liv	0.842
comorbid_aids_flag	0.618
comorbid_diabetes_cc_flag_score	1.933
comorbid_diabetes_flag_score	3.030
comorbid_chf_flag_score	4.229
comorbid_mi_flag_score	3.008
comorbid_peripheral_vascular_disease_flag_score	3.048
comorbid_cerebrovascular_disease_flag_score	2.358
comorbid_dementia_flag_score	1.259
comorbid_chronic_pulmonary_disease_flag_score	2.771
comorbid_rheumatologic_disease_flag_score	1.068
comorbid_peptic_ulcer_disease_flag_score	0.786

© 2021 Matheny ME et al. *JAMA Network Open.*

comorbid_mild_liver_disease_flag_score	1.009
comorbid_hemiplegia_or_paraplegia_flag_score	0.210
comorbid_moderate_or_severe_liver_disease_flag_score	0.846
comorbid_aids_flag_score	0.636
charlson_deyo_score	10.284
lace_los_score	10.915
lace_charlson_score	8.519
lace_score	18.470
lvef_flag	0.678
post_mi_cabg_flag	3.758
chf_flag	6.069
history_stroke_flag	2.840
grace_score_age	18.348
grace_score_creatinine_level_fi	9.428
grace_score_cardiac_arrest	2.399
grace_score_stemi	5.338
grace_score	33.824
disch_med_bb_flag	2.758
disch_med_bb_method	3.176
disch_med_antidep_flag	0.408
disch_med_antidep_method	0.414
disch_med_ace_arb_flag	0.809
disch_med_ace_arb_method	0.885
disch_med_aspirin_flag	3.197
disch_med_aspirin_method	3.740
disch_med_statin_flag	1.352
disch_med_statin_method	1.433
aki_flag	4.286
aki_stage	5.595
aki_stage_1_flag	1.498
aki_stage_2_flag	2.667
aki_stage_3_flag	1.178
sodium_level_avg	32.985
sodium_level_first	25.236
sodium_level_min	23.662
sodium_level_max	23.850
sodium_level_last	24.002
calcium_level_avg	27.979
calcium_level_min	16.905

© 2021 Matheny ME et al. *JAMA Network Open.*

calcium_level_max	12.939
calcium_level_first	15.141
calcium_level_last	13.277
creatinine_level_avg	24.552
creatinine_level_min	9.507
creatinine_level_max	13.342
creatinine_level_first	11.412
creatinine_level_last	11.718
hemoglobin_level_min	20.288
hemoglobin_level_avg	33.587
hemoglobin_level_max	21.034
hemoglobin_level_first	23.293
hemoglobin_level_last	19.378
bnp_level_avg	33.446
bnp_level_min	33.324
bnp_level_max	34.016
bnp_level_first	33.325
bnp_level_last	34.281
troponin_i_avg	30.850
bnp_first_cat	7.570
creatinine_level_diff	10.437
hemoglobin_level_diff	20.109
bnp_level_diff	29.533
ck_level_max2	32.155

If pooled feature importance was 0 when rounded to 3 decimals, it was removed from the list

© 2021 Matheny ME et al. *JAMA Network Open.*

eTable 7: Pooled Relative Influence Values from Final Gradient Boosting Model for VUMC

Variable Name	Pooled Relative Influence
hospital_score	15.099
age_at_admit	10.248
hemoglobin_level_avg	8.889
discharge_location	8.090
creatinine_level_avg	6.517
lace_score	5.333
prior_year_admissions_count	4.221
creatinine_level_max	3.676
bnp_level_last	3.138
grace_score	2.844
prim_diag	2.602
troponin_i_avg	2.389
hemoglobin_level_first	2.141
comorbid_chf_flag	1.979
comorbid_ckd_flag	1.881
calcium_level_avg	1.746
ck_level_max2	1.642
sodium_level_avg	1.526
prior_hypokalemia_90d	1.400
aki_stage	1.324
bnp_level_first	1.244
bnp_level_diff	1.157
history_pvd_flag	1.074
hemoglobin_level_max	0.996
calcium_level_last	0.927
bnp_level_avg	0.869
comorbid_mild_liver_disease_fla	0.794
sodium_level_min	0.738
major_depression_count	0.700
creatinine_level_min	0.666
bnp_level_max	0.621
grace_score_age	0.574
comorbid_peripheral_vascular_di	0.525
sodium_level_first	0.458
aki_flag	0.418
bnp_level_min	0.359

© 2021 Matheny ME et al. *JAMA Network Open.*

disch_med_bb_flag	0.296
history_angina_flag	0.238
disch_med_bb_method	0.197
gender	0.157
race	0.128
ethnicity	0.069
prior_sepsis_30d	0.067
prior_hyperkalemia_30d	0.024
prior_hypokalemia_30d	0.022

If pooled relative influence was 0 when rounded to 3 decimals, it was removed from the list

© 2021 Matheny ME et al. *JAMA Network Open.*

eTable 8: Full Variable List Value Set Definitions

Element	Notes
GENDER	Uses CONCEPT_NAME from the CONCEPT table.
RACE	Uses CONCEPT_NAME from the CONCEPT table.
ETHNICITY	Uses CONCEPT_NAME from the CONCEPT table.
PRIM_DIAG	When both ICD-9 and ICD-10 are present, uses ICD-9.
**Table 1: Prior Month Diagnosis**	The code for this component of Table 1 looks for an instance of the specified condition during the 30 day period prior to the admission. If the condition was present during that period, a value of one is given to that variable. Otherwise, a value of zero is given to that variable. Please see below to find a list of the conditions and the diagnosis codes used in this query.
PRIOR_SEPSIS_30D	Codes: 995.91, A41.9
PRIOR_HYPERKALEMIA_30D	Codes: 276.7, E87.5
PRIOR_HYPOKALEMIA_30D	Codes: 276.8, E87.6
PRIOR_HYPERVOLEMIA_30D	Codes: 276.61, E87.71
PRIOR_AKF_30D	Codes: 584, N17, N17.0, N17.1, N17.2, N17.8, N17.9
**Table 1: Prior 3 Month Diagnosis**	The code for this component of Table 1 looks for an instance of the specified condition during the 90 day period prior to the admission. If the condition was present during that period, a value of one is given to that variable. Otherwise, a value of zero is given to that variable. Please see below to find a list of the conditions and the diagnosis codes used in this query.
PRIOR_SEPSIS_90D	Codes: 995.91, A41.9
PRIOR_DIS_MAGN_METAB_90D	Codes: 275.2, E83.40, E83.41, E83.42, E83.49
PRIOR_HYPOKALEMIA_90D	Codes: 276.8, E87.6
PRIOR_AKF_90D	Codes: 584, N17, N17.0, N17.1, N17.2, N17.8, N17.9

**Table 1: HOSPITAL Score**	
LOS	Length of stay (LOS) is defined as (Discharge Date minus Admit Date) + 1.
LOS5_FLAG	If the length of stay >=5, then this value is set to one. Otherwise, this value is set to zero.
PRIOR_YEAR_ADMISSIONS_COUNT	Count of the number of admissions with a discharge date within 365 days before the admission date of the index admission.
ONCOLOGY_SERVICE_FLAG	If a condition ID from the condition events table for this visit is equal to 10 or 419, then this value is set to one. Otherwise, zero. Please see appendices.
HEMOGLOBIN_LEVEL_LAST_12_FLAG	Of the hemoglobin values associated with the admission, the last one is selected. If that value is < 12 g/DL, then this variable is set to 1. Otherwise, this variable is set to zero.
SODIUM_LEVEL_LAST_135_FLAG	Of the sodium values associated with the admission, the last one is selected. If that value is < 135 mEq/L, then this variable is set to 1. Otherwise, this variable is set to zero.
HOSPITAL_SCORE	This value is composed of a sum of values associated the flags listed above based on the following:<br>--If LOS5_FLAG = 1, then add 2 to HOSPITAL_SCORE.<br>--If PROCEDURE_FLAG = 1, then add 1 to HOSPITAL_SCORE.<br>--If PRIOR_YEAR_ADMISSIONS_COUNT = 2 to 5, then add 2 to HOSPITAL_SCORE.<br>--If PRIOR_YEAR_ADMISSIONS_COUNT > 5, then add 5 to HOSPITAL_SCORE.

© 2021 Matheny ME et al. *JAMA Network Open.*

	--If NONELECTIVE_ADMISSION_FLAG = 1, then add 1 to HOSPITAL_SCORE.<br>--If ONCOLOGY_SERVICE_FLAG = 1, then add 2 to HOSPITAL_SCORE.<br>--If HEMOGLOBIN_LEVEL_LAST_12_FLAG = 1, then add 1 to HOSPITAL_SCORE.<br>--If SODIUM_LEVEL_LAST_135_FLAG = 1, then add 1 to HOSPITAL_SCORE.

**Table 1: Laboratories**	
SODIUM_LEVEL_AVG	Of the sodium values associated with the admission, the average is calculated. (mEq/L)
SODIUM_LEVEL_MIN	Of the sodium values associated with the admission, the minimum value is selected. (mEq/L)
SODIUM_LEVEL_MAX	Of the sodium values associated with the admission, the maximum value is selected. (mEq/L)
SODIUM_LEVEL_FIRST	Of the sodium values associated with the admission, the first value is selected. (mEq/L)
SODIUM_LEVEL_LAST	Of the sodium values associated with the admission, the last value is selected. (mEq/L)
SODIUM_LEVEL_AVG_136_FLAG	Of the sodium values associated with the admission, the average is calculated. If that value is < 136 mEq/L, then this variable is set to 1. Otherwise, this variable is set to zero.
CALCIUM_LEVEL_AVG	Of the calcium values associated with the admission, the average is calculated. (mg/dL)
CALCIUM_LEVEL_MIN	Of the calcium values associated with the admission, the minimum value is selected. (mg/dL)
CALCIUM_LEVEL_MAX	Of the calcium values associated with the admission, the maximum value is selected. (mg/dL)
CALCIUM_LEVEL_FIRST	Of the calcium values associated with the admission, the first value is selected. (mg/dL)
CALCIUM_LEVEL_LAST	Of the calcium values associated with the admission, the last value is selected. (mg/dL)
CALCIUM_LEVEL_AVG_86_FLAG	Of the calcium values associated with the admission, the average is calculated. If that value is < 8.6 mg/dL, then this variable is set to 1. Otherwise, this variable is set to zero.
CREATININE_LEVEL_AVG	Of the creatinine values associated with the admission, the average is calculated. (mg/dL)
CREATININE_LEVEL_MIN	Of the creatinine values associated with the admission, the minimum value is selected. (mg/dL)
CREATININE_LEVEL_MAX	Of the creatinine values associated with the admission, the maximum value is selected. (mg/dL)
CREATININE_LEVEL_FIRST	Of the creatinine values associated with the admission, the first value is selected. (mg/dL)
CREATININE_LEVEL_LAST	Of the creatinine values associated with the admission, the last value is selected. (mg/dL)
HEMOGLOBIN_LEVEL_AVG	Of the hemoglobin values associated with the admission, the average is calculated. (g/dL)
HEMOGLOBIN_LEVEL_MIN	Of the hemoglobin values associated with the admission, the minimum value is selected. (g/dL)
HEMOGLOBIN_LEVEL_MAX	Of the hemoglobin values associated with the admission, the maximum value is selected. (g/dL)
HEMOGLOBIN_LEVEL_FIRST	Of the hemoglobin values associated with the admission, the first value is selected. (g/dL)
HEMOGLOBIN_LEVEL_LAST	Of the hemoglobin values associated with the admission, the last value is selected. (g/dL)
BNP_LEVEL_AVG	Of the brain 33atriuretic peptide values associated with the admission, the average is calculated. (pg/mL)

© 2021 Matheny ME et al. *JAMA Network Open.*

BNP_LEVEL_MIN	Of the brain 34atriuretic peptide values associated with the admission, the minimum value is selected. (pg/mL)
BNP_LEVEL_MAX	Of the brain 34atriuretic peptide values associated with the admission, the maximum value is selected. (pg/mL)
BNP_LEVEL_FIRST	Of the brain 34atriuretic peptide values associated with the admission, the first value is selected. (pg/mL)
BNP_LEVEL_LAST	Of the brain 34atriuretic peptide values associated with the admission, the last value is selected. (pg/mL)

**Table 1: Presentation/Disease**	
AMI_LOCATION	If the primary diagnosis code for the visit is in the set of AMI codes, then the value is assigned as follows:<br>('410.00', '410.01') then 'Anterolateral wall'<br>('410.10', 'I21.09') then 'Other anterior wall'<br>('410.11', 'I21.0', 'I21.02') then 'Anterior wall'<br>('410.20','410.21', 'I21.19') then 'Inferolateral wall'<br>('410.30', '410.31', 'I21.11') then 'Inferoposterior wall'<br>('410.40', '410.41', 'I21.19') then 'Other inferior wall'<br>('410.50','410.51') then 'Other lateral wall'<br>('410.60', '410.61') then 'True posterior wall infarction initial episode of care'<br>('410.70', '410.71', 'I21.4') then 'Subendocardial infarction'<br>('410.80', '410.81', 'I21.29', 'I21.2') then 'Other specified sites'<br>('410.90', '410.91', 'I21.3') then 'Unspecified site'<br>Otherwise, 'NA'.

**Table 1: Administrative Data**	
Index_LOS	Defined as (Discharge Date minus Admit Date) + 1 for the Index admission.
Admission_Prior_30_Days_Count	Total number of inpatient admissions during the 30 days prior to admission date.

**Table 1: Discharge Information**	
STEMI_Flag	If a diagnosis code for the visit is categorized as STEMI, then this value is set to one. Otherwise, this value is set to zero. Please see appendices.
NSTEMI_Flag	If a diagnosis code for the visit is categorized as NSTEMI, then this value is set to one. Otherwise, this value is set to zero. Please see appendices.
DISCH_MED_BB_FLAG	Beta blocker on discharge
DISCH_MED_ANTIDEP_FLAG	Antidepressant on discharge
DISCH_MED_ACE_ARB_FLAG	ACE or ARB inhibitor on discharge
DISCH_MED_ASPIRIN_FLAG	Aspirin or aspirin alternative on discharge
DISCH_MED_BB_METHOD<br>DISCH_MED_ANTIDEP_METHOD<br>DISCH_MED_ACE_ARB_METHOD<br>DISCH_MED_ASPIRIN_METHOD	Logic for each method listed below (If flag = 0, then method = 0):<br>1) (Considered to be the most reliable for indication of a discharge medication.)<br>• Drug exposure type includes the following:<br>-- Prescription written<br>-- Prescription dispensed in pharmacy<br>• Date criteria: The drug exposure date occurred on the day before, day of, or day after discharge.<br>2)<br>• Drug exposure type includes the following:<br>-- Physician administered drug<br>-- Inpatient administration<br>-- CPT Codes in Drug

	• Date criteria: The drug exposure date-time occurred up to 24 hours before the discharge date-time.<br><br>3) (Considered to be the least reliable for indication of a discharge medication.)<br>• Drug exposure type includes the following:<br>-- Medication list entry<br>-- Patient Self-Reported Medication<br>• Date criteria: The drug exposure date occurred on the discharge date.

**Table 1: Demographics and Additions**	
Age_at_Admit	Difference between date of birth and admit date in years.
Discharge_Location	The value of the Discharge_To_Concept_ID for the current visit in the Visit_Occurrence table.
Rehab_Flag	If a diagnosis code for the visit is categorized as "Rehabilitation", then this value is set to one. Otherwise, this value is set to zero. Please see appendices.

**Table 1: Patient History**	
History_Chest_Pain_Flag	If a diagnosis code for a prior visit is categorized as "Chest Pain", then this value is set to one. Otherwise, this value is set to zero. Please see appendices.
History_AMI_Flag	If a condition ID from the condition events table for a prior visit for this patient is equal to 13 or 380, then this value is set to one. Otherwise, zero. Please see appendices.
History_PVD_Flag	If a condition ID from the condition events table for a prior visit for this patient is equal to 12 or 282, then this value is set to one. Otherwise, zero Please see appendices.
History_Angina_Flag	If a condition ID from the condition events table for a prior visit for this patient is equal to 203, then this value is set to one. Otherwise, zero. Please see appendices.
History_Unstable_Angina_Flag	If a condition ID from the condition events table for a prior visit for this patient is equal to 53, then this value is set to one. Otherwise, zero. Please see appendices.
History_Hypertension_Flag	If a condition ID from the condition events table for a prior visit for this patient is equal to 6, 406, or 407, then this value is set to one. Otherwise, zero. Please see appendices.
History_Depression_Flag	If a condition ID from the condition events table for a prior visit for this patient is equal to 431, then this value is set to one. Otherwise, zero. Please see appendices.
Major_Depression_Count	If a condition ID from the condition events table for a prior visit for this patient is equal to 431, then the number of such visits within the prior year is assigned here. Please see appendices.

**Table 1: In-hospital Outcomes**	
IN_HOSPITAL_HF_FLAG	If a diagnosis code for this visit is categorized as "HF", then this value is set to one. Otherwise, this value is set to zero. Please see appendices.
IN_HOSPITAL_ISCHEMIA_FLAG	If a diagnosis code for this visit is categorized as "Ischemic Stroke", then this value is set to one. Otherwise, this value is set to zero Please see appendices.

**Table 1: Comorbidities**	
AGE_80_FLAG	If the number of years between the patient date of birth and the admission date >= 80, then this value is set to one. Otherwise, zero.

© 2021 Matheny ME et al. *JAMA Network Open.*

COMORBID_ARRHYTHMIA_FLAG	If a condition ID from the condition events table for a prior visit during the preceding year for this patient is equal to 35 or 402, then this value is set to one. Otherwise, zero. Please see appendices.
COMORBID_ANEMIA_FLAG	If a condition ID from the condition events table for a prior visit during the preceding year for this patient is equal to 40, 426, 427, or 642, then this value is set to one. Otherwise, zero. Please see appendices.
COMORBID_HYPERTENSION_FLAG	If a condition ID from the condition events table for a prior visit during the preceding year for this patient is equal to 6, 76, 320, 406, or 407, then this value is set to one. Otherwise, zero. Please see appendices.
COMORBID_COPD_FLAG	If a condition ID from the condition events table for a prior visit during the preceding year for this patient is equal to 45 or 98, then this value is set to one. Otherwise, zero Please see appendices.
COMORBID_CKD_FLAG	If a condition ID from the condition events table for a prior visit during the preceding year for this patient is equal to 2, then this value is set to one. Otherwise, zero. Please see appendices.
COMORBID_STROKE_FLAG	If a condition ID from the condition events table for a prior visit during the preceding year for this patient is equal to 80, then this value is set to one. Otherwise, zero Please see appendices.
COMORBID_TOBACCO_USE_FLAG	If a condition ID from the condition events table for a prior visit during the preceding year for this patient is equal to 49, then this value is set to one. Otherwise, zero. Please see appendices.
COMORBID_DEPRESSION_FLAG	If a condition ID from the condition events table for a prior visit during the preceding year for this patient is equal to 431 or 654, then this value is set to one. Otherwise, zero. Please see appendices.
COMORBID_HYPERCHOLESTEROLEMIA_FLAG	If a diagnosis code for a prior visit during the preceding year is categorized as "Hypercholesterolemia", then this value is set to one. Otherwise, this value is set to zero. Please see appendices.
COMORBID_CAD_FLAG	If a diagnosis code for a prior visit during the preceding year is categorized as "Coronary Artery Disease", then this value is set to one. Otherwise, this value is set to zero. Please see appendices.
COMORBID_DIABETES_CC_FLAG	If a condition ID from the condition events table for a prior visit during the preceding year for this patient is equal to 290, then this value is set to one. Otherwise, zero Please see appendices.
COMORBID_DIABETES_FLAG	If a condition ID from the condition events table for a prior visit during the preceding year for this patient is equal to 3, 289, 290, 411, or 412, then this value is set to one. Otherwise, zero. Please see appendices.
COMORBID_CHF_FLAG	If a condition ID from the condition events table for a prior visit during the preceding year for this patient is equal to 79 or 678, then this value is set to one. Otherwise, zero. Please see appendices.
COMORBID_MI_FLAG	If a condition ID from the condition events table for a prior visit during the preceding year for this patient is equal to 13 or 280, then this value is set to one. Otherwise, zero. Please see appendices.
COMORBID_PERIPHERAL_VASCULAR_DISEASE_FLAG	If a condition ID from the condition events table for a prior visit during the preceding year for this patient is equal to 12, 282, or 405, then this value is set to one. Otherwise, zero. Please see appendices.

© 2021 Matheny ME et al. *JAMA Network Open.*

COMORBID_CEREBROVASCULAR_DISEASE_FLAG	If a condition ID from the condition events table for a prior visit during the preceding year for this patient is equal to 5 or 283, then this value is set to one. Otherwise, zero. Please see appendices.
COMORBID_DEMENTIA_FLAG	If a condition ID from the condition events table for a prior visit during the preceding year for this patient is equal to 284, 61, 644, or 645, then this value is set to one. Otherwise, zero. Please see appendices.
COMORBID_CHRONIC_PULMONARY_DISEASE_FLAG	If a condition ID from the condition events table for a prior visit during the preceding year for this patient is equal to 285 or 410, then this value is set to one. Otherwise, zero. Please see appendices.
COMORBID_RHEUMATOLOGIC_DISEASE_FLAG	If a condition ID from the condition events table for a prior visit during the preceding year for this patient is equal to 151 or 286, then this value is set to one. Otherwise, zero. Please see appendices.
COMORBID_PEPTIC_ULCER_DISEASE_FLAG	If a condition ID from the condition events table for a prior visit during the preceding year for this patient is equal to 287, 416, or 152, then this value is set to one. Otherwise, zero. Please see appendices.
COMORBID_MILD_LIVER_DISEASE_FLAG	If a condition ID from the condition events table for a prior visit during the preceding year for this patient is equal to 288, then this value is set to one. Otherwise, zero. Please see appendices.
COMORBID_HEMIPLEGIA_OR_PARAPLEGIA_FLAG	If a condition ID from the condition events table for a prior visit during the preceding year for this patient is equal to 153 or 291, then this value is set to one. Otherwise, zero. Please see appendices.
COMORBID_RENAL_DISEASE_FLAG	If a condition ID from the condition events table for a prior visit during the preceding year for this patient is equal to 292, then this value is set to one. Otherwise, zero. Please see appendices.
COMORBID_MODERATE_OR_SEVERE_LIVER_DISEASE_FLAG	If a condition ID from the condition events table for a prior visit during the preceding year for this patient is equal to 294, then this value is set to one. Otherwise, zero. Please see appendices.
COMORBID_AIDS_FLAG	If a condition ID from the condition events table for a prior visit during the preceding year for this patient is equal to 296 or 417, then this value is set to one. Otherwise, zero. Please see appendices.
COMORBID_DIABETES_CC_FLAG_SCORE	If COMORBID_DIABETES_CC_FLAG = 1, then this score is set = 2. Otherwise, zero.
COMORBID_DIABETES_FLAG_SCORE	If COMORBID_DIABETES_FLAG = 1, then this score is set = 1. Otherwise, zero.
COMORBID_CHF_FLAG_SCORE	If COMORBID_CHF_FLAG = 1, then this score is set = 1. Otherwise, zero.
COMORBID_MI_FLAG_SCORE	If COMORBID_MI_FLAG = 1, then this score is set = 1. Otherwise, zero.
COMORBID_PERIPHERAL_VASCULAR_DISEASE_FLAG_SCORE	If COMORBID_PERIPHERAL_VASCULAR_DISEASE_FLAG = 1, then this score is set = 1. Otherwise, zero.
COMORBID_CEREBROVASCULAR_DISEASE_FLAG_SCORE	If COMORBID_CEREBROVASCULAR_DISEASE_FLAG = 1, then this score is set = 1. Otherwise, zero.
COMORBID_DEMENTIA_FLAG_SCORE	If COMORBID_DEMENTIA_FLAG = 1, then this score is set = 1. Otherwise, zero.
COMORBID_CHRONIC_PULMONARY_DISEASE_FLAG_SCORE	If COMORBID_CHRONIC_PULMONARY_FLAG = 1, then this score is set = 1. Otherwise, zero.

© 2021 Matheny ME et al. *JAMA Network Open.*

COMORBID_RHEUMATOLOGIC_DISEASE_FLAG_SCORE	If COMORBID_RHEUMATOLOGIC_DISEASE_FLAG = 1, then this score is set = 1. Otherwise, zero.
COMORBID_PEPTIC_ULCER_DISEASE_FLAG_SCORE	If COMORBID_PEPTIC_ULCER_DISEASE_FLAG = 1, then this score is set = 1. Otherwise, zero.
COMORBID_MILD_LIVER_DISEASE_FLAG_SCORE	If COMORBID_MILD_LIVER_DISEASE_FLAG = 1, then this score is set = 1. Otherwise, zero.
COMORBID_HEMIPLEGIA_OR_PARAPLEGIA_FLAG_SCORE	If COMORBID_HEMIPLEGIA_OR_PARAPLEGIA_FLAG = 1, then this score is set = 2. Otherwise, zero.
COMORBID_RENAL_DISEASE_FLAG_SCORE	If COMORBID_RENAL_DISEASE_FLAG = 1, then this score is set = 2. Otherwise, zero.
COMORBID_MODERATE_OR_SEVERE_LIVER_DISEASE_FLAG_SCORE	If COMORBID_ MODERATE_OR_SEVERE_LIVER _DISEASE_FLAG = 1, then this score is set = 3. Otherwise, zero.
COMORBID_AIDS_FLAG_SCORE	If COMORBID_AIDS_FLAG = 1, then this score is set = 6. Otherwise, zero.
CHARLSON_DEYO_SCORE	Comorbid_Diabetes_CC_Flag_Score<br>+ Comorbid_Diabetes_Flag_Score<br>+ Comorbid_CHF_Flag_Score<br>+ Comorbid_MI_Flag_Score<br>+ Comorbid_Peripheral_Vascular_Disease_Flag_Score<br>+ Comorbid_Cerebrovascular_Disease_Flag_Score<br>+ Comorbid_Dementia_Flag_Score<br>+ Comorbid_Chronic_Pulmonary_Disease_Flag_Score<br>+ Comorbid_Rheumatologic_Disease_Flag_Score<br>+ Comorbid_Peptic_Ulcer_Disease_Flag_Score<br>+ Comorbid_Mild_Liver_Disease_Flag_Score<br>+ Comorbid_Hemiplegia_or_Paraplegia_Flag_Score<br>+ Comorbid_Renal_Disease_Flag_Score<br>+ Comorbid_Moderate_or_Severe_Liver_Disease_Flag_Score<br>+ Comorbid_AIDS_Flag_Score

**Table 1: LACE Score**	
LACE_LOS_SCORE	When LOS between 0 and 1 then 1.<br>When LOS = 2 then 2.<br>When LOS = 3 then 3.<br>When LOS between 4 and 6 then 4.<br>When LOS between 7 and 13 then 5.<br>When LOS >= 14 then 7.
LACE_CHARLSON_SCORE	When Charlson_Deyo_Score = 0 then 0.<br>When Charlson_Deyo_Score = 1 then 1.<br>When Charlson_Deyo_Score = 2 then 2.<br>When Charlson_Deyo_Score = 3 then 3.<br>When Charlson_Deyo_Score >= 4 then 5.
LACE_SCORE	LACE_Acuity_Score<br>+ LACE_LOS_Score<br>+ LACE_Charlson_Score<br>+ LACE_ED_Score

**Table 1: ENRICHD Score**	
LVEF_FLAG	If a diagnosis code for this visit is categorized as "LVEF", then this value is set to one. Otherwise, this value is set to zero. Please see appendices.
POST_MI_CABG_FLAG	If a CABG (condition code 48) occurs after an MI (condition code 13 or 280), then this value is set to one, Otherwise, zero.

© 2021 Matheny ME et al. *JAMA Network Open.*

CHF_FLAG	If a condition ID from the condition events table for a prior visit for this patient is equal to 79 then this value is set to one. Otherwise, zero. Please see appendices.
HISTORY_STROKE_FLAG	If a condition ID from the condition events table for a prior visit for this patient is equal to 80 then this value is set to one. Otherwise, zero. Please see appendices.

**Table 1: GRACE Score**	
TROPONIN_AVG	The average of Tropinin I. readings for this visit in ng/mL.
CARDIAC_MARKER_ELEVATION_FLAG	When the average Troponin I. reading for this visit >.04, then this value is set to one. Otherwise, zero.
GRACE_SCORE_AGE	When AGE_AT_ADMIT < 30 then 0.<br>When AGE_AT_ADMIT between 30 and 39 then 8.<br>When AGE_AT_ADMIT between 40 and 49 then 25.<br>When hen AGE_AT_ADMIT between 50 and 59 then 41.<br>When AGE_AT_ADMIT between 60 and 69 then 58.<br>When AGE_AT_ADMIT between 70 and 79 then 75.<br>When AGE_AT_ADMIT between 80 and 89 then 91.<br>When AGE_AT_ADMIT >= 90 then 100.
GRACE_SCORE_CREATININE_LEVEL_FIRST	When CREATININE_LEVEL_FIRST < 0.4 then 1.<br>When CREATININE_LEVEL_FIRST between 0.4 and 0.79 then 4.<br>When CREATININE_LEVEL_FIRST between 0.8 and 1.19 then 7.<br>When CREATININE_LEVEL_FIRST between 1.2 and 1.59 then 10.<br>When CREATININE_LEVEL_FIRST between 1.6 and 1.99 then 13.<br>When CREATININE_LEVEL_FIRST between 0.2 and 3.99 then 21.<br>When CREATININE_LEVEL_FIRST >= 4 then 28.
GRACE_SCORE_CARDIAC_ARREST	When Cardiac_Arrest_Flag = 1 then 39. Otherwise, 0.
GRACE_SCORE_STEMI	When STEMI_FLAG = 1 then 28. Otherwise, 0.
GRACE_SCORE	Grace_Score_Age<br>+ Grace_Score_CREATININE_LEVEL_FIRST<br>+ Grace_Score_Cardiac_Arrest<br>+ Grace_Score_STEMI

**AKI Stage Variables**	AKI stage uses creatinine values during the admission to compare to creatinine values up to two years before the admission. Each creatinine value during the admission is compared to a baseline creatinine value calculated from creatinine values obtained prior to the admission. AKI stage is calculated based on the level of increase from baseline creatinine to the creatinine values during the admission.<br><br>Calculation for AKI Stage:<br>If Anchor_Creatinine/Baseline_Creatinine >= 1.5<br>OR (Anchor_Creatinine - Baseline_Creatinine) >= 0.3<br>THEN AKI Stage = 1<br><br>If Anchor_Creatinine/Baseline_Creatinine >= 2.0<br>THEN AKI Stage = 2<br><br>If Anchor_Creatinine/Baseline_Creatinine >= 3.0<br>THEN AKI Stage = 3
AKI_Flag	Equal to 1 if AKI stage > 0 for any creatinine measure during the admission.

© 2021 Matheny ME et al. *JAMA Network Open.*

1	Dialysis,Procedure	46177005	End-stage renal disease	193782
1	Dialysis,Procedure	73257006	Peritoneal dialysis catheter maintenance	4247794
1	Dialysis,Procedure	108241001	Dialysis procedure	4032243
1	Dialysis,Procedure	251859005	Dialysis finding	4090651
2	Chronic Kidney Disease,Condition	193003	Benign hypertensive renal disease	193493
2	Chronic Kidney Disease,Condition	38481006	Hypertensive renal disease	201313
2	Chronic Kidney Disease,Condition	46177005	End-stage renal disease	193782
2	Chronic Kidney Disease,Condition	65443008	Malignant hypertensive renal disease	442766
2	Chronic Kidney Disease,Condition	66052004	Benign hypertensive heart AND renal disease	442626
2	Chronic Kidney Disease,Condition	66610008	Malignant hypertensive heart AND renal disease	442603
2	Chronic Kidney Disease,Condition	86234004	Hypertensive heart AND renal disease	195556
2	Chronic Kidney Disease,Condition	194774006	Hypertensive renal disease with renal failure	439697
2	Chronic Kidney Disease,Condition	194779001	Hypertensive heart and renal disease with (congestive) heart failure	439696
2	Chronic Kidney Disease,Condition	194780003	Hypertensive heart and renal disease with renal failure	439695
2	Chronic Kidney Disease,Condition	194781004	Hypertensive heart and renal disease with both (congestive) heart failure and renal failure	439694
2	Chronic Kidney Disease,Condition	431855005	Chronic kidney disease stage 1	443614

© 2021 Matheny ME et al. *JAMA Network Open.*

2	Chronic Kidney Disease,Condition	431856006	Chronic kidney disease stage 2	443601
2	Chronic Kidney Disease,Condition	431857002	Chronic kidney disease stage 4	443612
2	Chronic Kidney Disease,Condition	433144002	Chronic kidney disease stage 3	443597
2	Chronic Kidney Disease,Condition	433146000	Chronic kidney disease stage 5	443611
2	Chronic Kidney Disease,Condition	709044004	Chronic kidney disease	46271022
2	Chronic Kidney Disease,Condition	8501000119104	Hypertensive heart and chronic kidney disease	44784621
2	Chronic Kidney Disease,Condition	285831000119108	Malignant hypertensive chronic kidney disease	43021852
2	Chronic Kidney Disease,Condition	285841000119104	Malignant hypertensive end stage renal disease	43020455

© 2021 Matheny ME et al. *JAMA Network Open.*

Mapping of Condition IDs to SNOMED Codes

CONDITIONID	CONDITION_DESCRIPTION	SNOMED_CODE	DESCRIPTION
1	Dialysis,Procedure	46177005	End-stage renal disease
1	Dialysis,Procedure	73257006	Peritoneal dialysis catheter maintenance
1	Dialysis,Procedure	108241001	Dialysis procedure
1	Dialysis,Procedure	251859005	Dialysis finding
2	Chronic Kidney Disease,Condition	193003	Benign hypertensive renal disease
2	Chronic Kidney Disease,Condition	38481006	Hypertensive renal disease
2	Chronic Kidney Disease,Condition	46177005	End-stage renal disease
2	Chronic Kidney Disease,Condition	65443008	Malignant hypertensive renal disease
2	Chronic Kidney Disease,Condition	66052004	Benign hypertensive heart AND renal disease
2	Chronic Kidney Disease,Condition	66610008	Malignant hypertensive heart AND renal disease
2	Chronic Kidney Disease,Condition	86234004	Hypertensive heart AND renal disease
2	Chronic Kidney Disease,Condition	194774006	Hypertensive renal disease with renal failure
2	Chronic Kidney Disease,Condition	194779001	Hypertensive heart and renal disease with (congestive) heart failure
2	Chronic Kidney Disease,Condition	194780003	Hypertensive heart and renal disease with renal failure
2	Chronic Kidney Disease,Condition	194781004	Hypertensive heart and renal disease with both (congestive) heart

© 2021 Matheny ME et al. *JAMA Network Open.*

CONDITIONID	CONDITION_DESCRIPTION	SNOMED_CODE	DESCRIPTION
			failure and renal failure
2	Chronic Kidney Disease,Condition	431855005	Chronic kidney disease stage 1
2	Chronic Kidney Disease,Condition	431856006	Chronic kidney disease stage 2
2	Chronic Kidney Disease,Condition	431857002	Chronic kidney disease stage 4
2	Chronic Kidney Disease,Condition	433144002	Chronic kidney disease stage 3
2	Chronic Kidney Disease,Condition	433146000	Chronic kidney disease stage 5
2	Chronic Kidney Disease,Condition	709044004	Chronic kidney disease
2	Chronic Kidney Disease,Condition	8501000119104	Hypertensive heart and chronic kidney disease
2	Chronic Kidney Disease,Condition	285831000119108	Malignant hypertensive chronic kidney disease
2	Chronic Kidney Disease,Condition	285841000119104	Malignant hypertensive end stage renal disease
3	Diabetes,Condition	4855003	Retinopathy due to diabetes mellitus
3	Diabetes,Condition	8801005	Secondary diabetes mellitus
3	Diabetes,Condition	20313009	Hyperosmolality
3	Diabetes,Condition	25093002	Disorder of eye due to diabetes mellitus
3	Diabetes,Condition	43959009	Cataract due to diabetes mellitus
3	Diabetes,Condition	44054006	Type 2 diabetes mellitus
3	Diabetes,Condition	46635009	Type 1 diabetes mellitus
3	Diabetes,Condition	49455004	Diabetic polyneuropathy

© 2021 Matheny ME et al. *JAMA Network Open.*

CONDITIONID	CONDITION_DESCRIPTION	SNOMED_CODE	DESCRIPTION
3	Diabetes,Condition	59276001	Proliferative retinopathy with diabetes mellitus
3	Diabetes,Condition	73211009	Diabetes mellitus
3	Diabetes,Condition	74627003	Diabetic complication
3	Diabetes,Condition	127013003	Kidney disorder due to diabetes mellitus
3	Diabetes,Condition	170747006	Diabetic on insulin
3	Diabetes,Condition	190329007	Diabetes mellitus with hyperosmolar coma
3	Diabetes,Condition	190330002	Type 1 diabetes mellitus with hyperosmolar coma
3	Diabetes,Condition	190331003	Type 2 diabetes mellitus with hyperosmolar coma
3	Diabetes,Condition	267384006	Hypoglycemic coma
3	Diabetes,Condition	312903003	Mild nonproliferative diabetic retinopathy
3	Diabetes,Condition	312904009	Moderate nonproliferative diabetic retinopathy
3	Diabetes,Condition	312905005	Severe nonproliferative diabetic retinopathy
3	Diabetes,Condition	312912001	Macular edema due to diabetes mellitus
3	Diabetes,Condition	360546002	Hypoglycemic shock

© 2021 Matheny ME et al. *JAMA Network Open.*

CONDITIONID	CONDITION_DESCRIPTION	SNOMED_CODE	DESCRIPTION
3	Diabetes,Condition	390834004	Nonproliferative diabetic retinopathy
3	Diabetes,Condition	420270002	Ketoacidosis in type 1 diabetes mellitus
3	Diabetes,Condition	420279001	Renal disorder due to type 2 diabetes mellitus
3	Diabetes,Condition	420422005	Diabetic ketoacidosis
3	Diabetes,Condition	420662003	Coma associated with diabetes mellitus
3	Diabetes,Condition	420868002	Disorder due to type 1 diabetes mellitus
3	Diabetes,Condition	421165007	Diabetic oculopathy associated with type 1 diabetes mellitus
3	Diabetes,Condition	421326000	Neurological disorder with type 2 diabetes mellitus
3	Diabetes,Condition	421365002	Peripheral circulatory disorder associated with type 1 diabetes mellitus
3	Diabetes,Condition	421437000	Hypoglycemic coma in type 1 diabetes mellitus
3	Diabetes,Condition	421468001	Neurological disorder with type 1 diabetes mellitus
3	Diabetes,Condition	421750000	Ketoacidosis in type 2 diabetes mellitus
3	Diabetes,Condition	421893009	Renal disorder associated with

© 2021 Matheny ME et al. *JAMA Network Open.*

CONDITIONID	CONDITION_DESCRIPTION	SNOMED_CODE	DESCRIPTION
			type 1 diabetes mellitus
3	Diabetes,Condition	421895002	Peripheral vascular disorder due to diabetes mellitus
3	Diabetes,Condition	422014003	Disorder due to type 2 diabetes mellitus
3	Diabetes,Condition	422088007	Nervous system disorder due to diabetes mellitus
3	Diabetes,Condition	422099009	Disorder of eye with type 2 diabetes mellitus
3	Diabetes,Condition	422166005	Peripheral circulatory disorder associated with type 2 diabetes mellitus
3	Diabetes,Condition	428896009	Hyperosmolality due to uncontrolled type 1 diabetes mellitus
3	Diabetes,Condition	443694000	Type II diabetes mellitus uncontrolled
3	Diabetes,Condition	444073006	Type 1 diabetes mellitus uncontrolled
5	Cerebrovascular Disease,Condition	1386000	Intracranial hemorrhage
5	Cerebrovascular Disease,Condition	2929001	Occlusion of artery
5	Cerebrovascular Disease,Condition	15258001	Subclavian steal syndrome
5	Cerebrovascular Disease,Condition	20059004	Cerebral artery occlusion
5	Cerebrovascular Disease,Condition	20262006	Ataxia
5	Cerebrovascular Disease,Condition	21454007	Subarachnoid hemorrhage
5	Cerebrovascular Disease,Condition	28366008	Cerebral arteritis

© 2021 Matheny ME et al. *JAMA Network Open.*

CONDITIONID	CONDITION_DESCRIPTION	SNOMED_CODE	DESCRIPTION
5	Cerebrovascular Disease,Condition	34781003	Vertebral artery syndrome
5	Cerebrovascular Disease,Condition	35486000	Subdural hemorrhage
5	Cerebrovascular Disease,Condition	42970005	Nonpyogenic thrombosis of intracranial venous sinus
5	Cerebrovascular Disease,Condition	42994005	Nonruptured cerebral aneurysm
5	Cerebrovascular Disease,Condition	43658003	Vertebral artery obstruction
5	Cerebrovascular Disease,Condition	50490005	Hypertensive encephalopathy
5	Cerebrovascular Disease,Condition	55382008	Cerebral atherosclerosis
5	Cerebrovascular Disease,Condition	62914000	Cerebrovascular disease
5	Cerebrovascular Disease,Condition	64009001	Basilar artery syndrome
5	Cerebrovascular Disease,Condition	69116000	Moyamoya disease
5	Cerebrovascular Disease,Condition	69798007	Carotid artery obstruction
5	Cerebrovascular Disease,Condition	71444005	Cerebral thrombosis
5	Cerebrovascular Disease,Condition	75543006	Cerebral embolism
5	Cerebrovascular Disease,Condition	87224000	Transient arterial retinal occlusion
5	Cerebrovascular Disease,Condition	95666008	Weakness of face muscles
5	Cerebrovascular Disease,Condition	195180004	Basilar artery occlusion
5	Cerebrovascular Disease,Condition	195183002	Multiple and bilateral precerebral arterial occlusion
5	Cerebrovascular Disease,Condition	195189003	Cerebral infarction due to thrombosis of cerebral arteries

© 2021 Matheny ME et al. *JAMA Network Open.*

CONDITIONID	CONDITION_DESCRIPTION	SNOMED_CODE	DESCRIPTION
5	Cerebrovascular Disease,Condition	195190007	Cerebral infarction due to embolism of cerebral arteries
5	Cerebrovascular Disease,Condition	195199008	Vertebrobasilar artery syndrome
5	Cerebrovascular Disease,Condition	195239002	Late effects of cerebrovascular disease
5	Cerebrovascular Disease,Condition	229621000	Disorder of fluency
5	Cerebrovascular Disease,Condition	230692004	Infarction - precerebral
5	Cerebrovascular Disease,Condition	230736007	Transient global amnesia
5	Cerebrovascular Disease,Condition	266253001	Precerebral arterial occlusion
5	Cerebrovascular Disease,Condition	266257000	Transient cerebral ischemia
5	Cerebrovascular Disease,Condition	274100004	Cerebral hemorrhage
5	Cerebrovascular Disease,Condition	287731003	Cerebral ischemia
5	Cerebrovascular Disease,Condition	288723005	Acute ill-defined cerebrovascular disease
5	Cerebrovascular Disease,Condition	397809001	Nontraumatic extradural hemorrhage
5	Cerebrovascular Disease,Condition	425642008	Monoplegia of dominant lower limb as a late effect of cerebrovascular accident
5	Cerebrovascular Disease,Condition	425882004	Paralytic syndrome as late effect of stroke
5	Cerebrovascular Disease,Condition	426033005	Dysphagia as a late effect of cerebrovascular accident
5	Cerebrovascular Disease,Condition	426788002	Vertigo as late effect of stroke

© 2021 Matheny ME et al. *JAMA Network Open.*

CONDITIONID	CONDITION_DESCRIPTION	SNOMED_CODE	DESCRIPTION
5	Cerebrovascular Disease,Condition	427065003	Monoplegia of dominant upper limb as a late effect of cerebrovascular accident
5	Cerebrovascular Disease,Condition	428668000	Apraxia due to cerebrovascular accident
5	Cerebrovascular Disease,Condition	430947007	Paralytic syndrome of nondominant side as late effect of stroke
5	Cerebrovascular Disease,Condition	430959006	Paralytic syndrome of dominant side as late effect of stroke
5	Cerebrovascular Disease,Condition	441529001	Dysphasia as late effect of cerebrovascular disease
5	Cerebrovascular Disease,Condition	441630004	Aphasia as late effect of cerebrovascular disease
5	Cerebrovascular Disease,Condition	441735003	Sensory disorder as a late effect of cerebrovascular disease
5	Cerebrovascular Disease,Condition	441887006	Monoplegia of lower limb as late effect of cerebrovascular disease
5	Cerebrovascular Disease,Condition	441894009	Monoplegia of nondominant lower limb as a late effect of cerebrovascular accident
5	Cerebrovascular Disease,Condition	441960006	Speech and language deficit as late effect of

© 2021 Matheny ME et al. *JAMA Network Open.*

CONDITIONID	CONDITION_DESCRIPTION	SNOMED_CODE	DESCRIPTION
			cerebrovascular accident
5	Cerebrovascular Disease,Condition	442024001	Hemiplegia as late effect of cerebrovascular disease
5	Cerebrovascular Disease,Condition	442097001	Monoplegia of upper limb as late effect of cerebrovascular disease
5	Cerebrovascular Disease,Condition	442181008	Monoplegia of nondominant upper limb as a late effect of cerebrovascular accident
5	Cerebrovascular Disease,Condition	442212003	Residual cognitive deficit as late effect of cerebrovascular accident
5	Cerebrovascular Disease,Condition	442668000	Hemiplegia of nondominant side as late effect of cerebrovascular disease
5	Cerebrovascular Disease,Condition	442676003	Hemiplegia of dominant side as late effect of cerebrovascular disease
5	Cerebrovascular Disease,Condition	87551000119101	Visual disturbance as sequela of cerebrovascular disease
5	Cerebrovascular Disease,Condition	133981000119106	Dysarthria as late effects of cerebrovascular disease

© 2021 Matheny ME et al. *JAMA Network Open.*

CONDITIONID	CONDITION_DESCRIPTION	SNOMED_CODE	DESCRIPTION
6	Hypertension,Condition	193003	Benign hypertensive renal disease
6	Hypertension,Condition	1201005	Benign essential hypertension
6	Hypertension,Condition	5148006	Hypertensive heart disease with congestive heart failure
6	Hypertension,Condition	31992008	Secondary hypertension
6	Hypertension,Condition	36221001	Benign hypertensive heart disease
6	Hypertension,Condition	36315003	Malignant hypertensive heart disease without congestive heart failure
6	Hypertension,Condition	38481006	Hypertensive renal disease
6	Hypertension,Condition	50490005	Hypertensive encephalopathy
6	Hypertension,Condition	54225002	Malignant hypertensive heart disease
6	Hypertension,Condition	59621000	Essential hypertension
6	Hypertension,Condition	60899001	Hypertensive heart disease without congestive heart failure
6	Hypertension,Condition	64715009	Hypertensive heart disease
6	Hypertension,Condition	65443008	Malignant hypertensive renal disease
6	Hypertension,Condition	66052004	Benign hypertensive heart AND renal disease

© 2021 Matheny ME et al. *JAMA Network Open.*

CONDITIONID	CONDITION_DESCRIPTION	SNOMED_CODE	DESCRIPTION
6	Hypertension,Condition	66610008	Malignant hypertensive heart AND renal disease
6	Hypertension,Condition	73410007	Benign secondary renovascular hypertension
6	Hypertension,Condition	77737007	Benign hypertensive heart disease with congestive heart failure
6	Hypertension,Condition	77970009	Benign hypertensive heart disease without congestive heart failure
6	Hypertension,Condition	78975002	Malignant essential hypertension
6	Hypertension,Condition	83105008	Malignant hypertensive heart disease with congestive heart failure
6	Hypertension,Condition	86234004	Hypertensive heart AND renal disease
6	Hypertension,Condition	89242004	Malignant secondary hypertension
6	Hypertension,Condition	123799005	Renovascular hypertension
6	Hypertension,Condition	194774006	Hypertensive renal disease with renal failure
6	Hypertension,Condition	194779001	Hypertensive heart and renal disease with (congestive) heart failure
6	Hypertension,Condition	194780003	Hypertensive heart and renal disease with renal failure

© 2021 Matheny ME et al. *JAMA Network Open.*

CONDITIONID	CONDITION_DESCRIPTION	SNOMED_CODE	DESCRIPTION
6	Hypertension,Condition	194781004	Hypertensive heart and renal disease with both (congestive) heart failure and renal failure
6	Hypertension,Condition	194783001	Malignant secondary renovascular hypertension
6	Hypertension,Condition	194785008	Benign secondary hypertension
6	Hypertension,Condition	8501000119104	Hypertensive heart and chronic kidney disease
6	Hypertension,Condition	285831000119108	Malignant hypertensive chronic kidney disease
6	Hypertension,Condition	285841000119104	Malignant hypertensive end stage renal disease
7	Renal Transplant,Procedure	58797008	Disorder of transplanted kidney
7	Renal Transplant,Procedure	161665007	History of renal transplant
9	Congestive Heart Failure,Condition	5148006	Hypertensive heart disease with congestive heart failure
9	Congestive Heart Failure,Condition	42343007	Congestive heart failure
9	Congestive Heart Failure,Condition	45227007	Hypertrophic obstructive cardiomyopathy
9	Congestive Heart Failure,Condition	77737007	Benign hypertensive heart disease with congestive heart failure

© 2021 Matheny ME et al. *JAMA Network Open.*

CONDITIONID	CONDITION_DESCRIPTION	SNOMED_CODE	DESCRIPTION
9	Congestive Heart Failure,Condition	82523003	Congestive rheumatic heart failure
9	Congestive Heart Failure,Condition	83105008	Malignant hypertensive heart disease with congestive heart failure
9	Congestive Heart Failure,Condition	83521008	Dilated cardiomyopathy secondary to alcohol
9	Congestive Heart Failure,Condition	84114007	Heart failure
9	Congestive Heart Failure,Condition	85232009	Left heart failure
9	Congestive Heart Failure,Condition	85898001	Cardiomyopathy
9	Congestive Heart Failure,Condition	194779001	Hypertensive heart and renal disease with (congestive) heart failure
9	Congestive Heart Failure,Condition	194781004	Hypertensive heart and renal disease with both (congestive) heart failure and renal failure
9	Congestive Heart Failure,Condition	195023001	Nutritional and metabolic cardiomyopathies
9	Congestive Heart Failure,Condition	195029002	Cardiomyopathy associated with another disorder
9	Congestive Heart Failure,Condition	233873004	Hypertrophic cardiomyopathy
9	Congestive Heart Failure,Condition	417996009	Systolic heart failure
9	Congestive Heart Failure,Condition	418304008	Diastolic heart failure
9	Congestive Heart Failure,Condition	441481004	Chronic systolic heart failure
9	Congestive Heart Failure,Condition	441530006	Chronic diastolic heart failure

© 2021 Matheny ME et al. *JAMA Network Open.*

CONDITIONID	CONDITION_DESCRIPTION	SNOMED_CODE	DESCRIPTION
9	Congestive Heart Failure,Condition	442304009	Combined systolic and diastolic dysfunction
9	Congestive Heart Failure,Condition	443253003	Acute on chronic systolic heart failure
9	Congestive Heart Failure,Condition	443254009	Acute systolic heart failure
9	Congestive Heart Failure,Condition	443343001	Acute diastolic heart failure
9	Congestive Heart Failure,Condition	443344007	Acute on chronic diastolic heart failure
9	Congestive Heart Failure,Condition	153931000119109	Acute combined systolic and diastolic heart failure
9	Congestive Heart Failure,Condition	153941000119100	Chronic combined systolic and diastolic heart failure
9	Congestive Heart Failure,Condition	153951000119103	Acute on chronic combined systolic and diastolic heart failure
10	Cancer,Condition	65399007	Langerhans cell histiocytosis
10	Cancer,Condition	91854005	Acute leukemia in remission
10	Cancer,Condition	91855006	Acute leukemia
10	Cancer,Condition	91856007	Acute lymphoid leukemia in remission
10	Cancer,Condition	91857003	Acute lymphoid leukemia
10	Cancer,Condition	91858008	Acute monocytic leukemia in remission
10	Cancer,Condition	91860005	Acute myeloid leukemia in remission

© 2021 Matheny ME et al. *JAMA Network Open.*

CONDITIONID	CONDITION_DESCRIPTION	SNOMED_CODE	DESCRIPTION
10	Cancer,Condition	91861009	Acute myeloid leukemia, disease
10	Cancer,Condition	92508006	Burkitt's tumor of intra-abdominal lymph nodes
10	Cancer,Condition	92510008	Burkitt's tumor of intrathoracic lymph nodes
10	Cancer,Condition	92511007	Burkitt's tumor of lymph nodes of axilla AND/OR upper limb
10	Cancer,Condition	92512000	Burkitt's tumor of lymph nodes of head, face AND/OR neck
10	Cancer,Condition	92515003	Burkitt's tumor of spleen
10	Cancer,Condition	92811003	Chronic leukemia in remission
10	Cancer,Condition	92812005	Chronic leukemia
10	Cancer,Condition	92813000	Chronic lymphoid leukemia in remission
10	Cancer,Condition	92814006	Chronic lymphoid leukemia, disease
10	Cancer,Condition	92817004	Chronic myeloid leukemia in remission
10	Cancer,Condition	92818009	Chronic myeloid leukemia
10	Cancer,Condition	93133006	Letterer-Siwe disease of intra-abdominal lymph nodes
10	Cancer,Condition	93134000	Letterer-Siwe disease of intrapelvic lymph nodes
10	Cancer,Condition	93135004	Letterer-Siwe disease of

© 2021 Matheny ME et al. *JAMA Network Open.*

CONDITIONID	CONDITION_DESCRIPTION	SNOMED_CODE	DESCRIPTION
			intrathoracic lymph nodes
10	Cancer,Condition	93136003	Letterer-Siwe disease of lymph nodes of axilla AND/OR upper limb
10	Cancer,Condition	93137007	Letterer-Siwe disease of lymph nodes of head, face AND/OR neck
10	Cancer,Condition	93138002	Letterer-Siwe disease of lymph nodes of inguinal region AND/OR lower limb
10	Cancer,Condition	93139005	Letterer-Siwe disease of lymph nodes of multiple sites
10	Cancer,Condition	93140007	Letterer-Siwe disease of spleen
10	Cancer,Condition	93142004	Leukemia in remission
10	Cancer,Condition	93143009	Leukemia
10	Cancer,Condition	93144003	Leukemic reticuloendotheliosis of intra-abdominal lymph nodes
10	Cancer,Condition	93145002	Leukemic reticuloendotheliosis of intrapelvic lymph nodes
10	Cancer,Condition	93146001	Leukemic reticuloendotheliosis of intrathoracic lymph nodes
10	Cancer,Condition	93147005	Leukemic reticuloendotheliosis of lymph nodes

© 2021 Matheny ME et al. *JAMA Network Open.*

CONDITIONID	CONDITION_DESCRIPTION	SNOMED_CODE	DESCRIPTION
			of axilla AND/OR upper limb
10	Cancer,Condition	93148000	Leukemic reticuloendotheliosis of lymph nodes of head, face AND/OR neck
10	Cancer,Condition	93149008	Leukemic reticuloendotheliosis of lymph nodes of inguinal region AND/OR lower limb
10	Cancer,Condition	93150008	Leukemic reticuloendotheliosis of lymph nodes of multiple sites
10	Cancer,Condition	93151007	Hairy cell leukemia of spleen
10	Cancer,Condition	93152000	Leukemic reticuloendotheliosis of extranodal AND/OR solid organ site
10	Cancer,Condition	93169003	Lymphoid leukemia in remission
10	Cancer,Condition	93182006	Malignant histiocytosis of intra-abdominal lymph nodes
10	Cancer,Condition	93183001	Malignant histiocytosis of intrapelvic lymph nodes
10	Cancer,Condition	93184007	Malignant histiocytosis of intrathoracic lymph nodes
10	Cancer,Condition	93185008	Malignant histiocytosis of lymph nodes of

© 2021 Matheny ME et al. *JAMA Network Open.*

CONDITIONID	CONDITION_DESCRIPTION	SNOMED_CODE	DESCRIPTION
			axilla AND/OR upper limb
10	Cancer,Condition	93186009	Malignant histiocytosis of lymph nodes of head, face AND/OR neck
10	Cancer,Condition	93187000	Malignant histiocytosis of lymph nodes of inguinal region AND/OR lower limb
10	Cancer,Condition	93188005	Malignant histiocytosis of lymph nodes of multiple sites
10	Cancer,Condition	93189002	Malignant histiocytosis of spleen
10	Cancer,Condition	93191005	Malignant lymphoma of intra-abdominal lymph nodes
10	Cancer,Condition	93192003	Malignant lymphoma of intrapelvic lymph nodes
10	Cancer,Condition	93193008	Malignant lymphoma of intrathoracic lymph nodes
10	Cancer,Condition	93194002	Malignant lymphoma of lymph nodes of axilla AND/OR upper limb
10	Cancer,Condition	93195001	Malignant lymphoma of lymph nodes of

© 2021 Matheny ME et al. *JAMA Network Open.*

CONDITIONID	CONDITION_DESCRIPTION	SNOMED_CODE	DESCRIPTION
			head, face AND/OR neck
10	Cancer,Condition	93196000	Malignant lymphoma of lymph nodes of inguinal region AND/OR lower limb
10	Cancer,Condition	93197009	Malignant lymphoma of lymph nodes of multiple sites
10	Cancer,Condition	93198004	Malignant lymphoma of spleen
10	Cancer,Condition	93201009	Malignant mast cell tumor of intrapelvic lymph nodes
10	Cancer,Condition	93202002	Malignant mast cell tumor of intrathoracic lymph nodes
10	Cancer,Condition	93203007	Malignant mast cell tumor of lymph nodes of axilla AND/OR upper limb
10	Cancer,Condition	93204001	Malignant mast cell tumor of lymph nodes of head, face AND/OR neck
10	Cancer,Condition	93205000	Malignant mast cell tumor of lymph nodes of inguinal region AND/OR lower limb
10	Cancer,Condition	93206004	Malignant mast cell tumor of lymph nodes of multiple sites

© 2021 Matheny ME et al. *JAMA Network Open.*

CONDITIONID	CONDITION_DESCRIPTION	SNOMED_CODE	DESCRIPTION
10	Cancer,Condition	93207008	Malignant mast cell tumor of spleen
10	Cancer,Condition	93224002	Malignant melanoma of skin of eyelid
10	Cancer,Condition	93225001	Malignant melanoma of skin of face
10	Cancer,Condition	93450001	Erythroleukemia in remission
10	Cancer,Condition	93451002	Erythroleukemia, FAB M6
10	Cancer,Condition	93487009	Hodgkin's disease, lymphocytic depletion of lymph nodes of axilla AND/OR upper limb
10	Cancer,Condition	93488004	Hodgkin's disease, lymphocytic depletion of lymph nodes of head, face AND/OR neck
10	Cancer,Condition	93489007	Hodgkin's disease, lymphocytic depletion of lymph nodes of inguinal region AND/OR lower limb
10	Cancer,Condition	93493001	Hodgkin's disease, lymphocytic-histiocytic predominance of intra-abdominal lymph nodes
10	Cancer,Condition	93494007	Hodgkin's disease, lymphocytic-histiocytic predominance of intrapelvic lymph nodes

© 2021 Matheny ME et al. *JAMA Network Open.*

CONDITIONID	CONDITION_DESCRIPTION	SNOMED_CODE	DESCRIPTION
10	Cancer,Condition	93495008	Hodgkin's disease, lymphocytic-histiocytic predominance of intrathoracic lymph nodes
10	Cancer,Condition	93496009	Hodgkin's disease, lymphocytic-histiocytic predominance of lymph nodes of axilla AND/OR upper limb
10	Cancer,Condition	93497000	Hodgkin's disease, lymphocytic-histiocytic predominance of lymph nodes of head, face AND/OR neck
10	Cancer,Condition	93498005	Hodgkin's disease, lymphocytic-histiocytic predominance of lymph nodes of inguinal region AND/OR lower limb
10	Cancer,Condition	93500006	Hodgkin's disease, lymphocytic-histiocytic predominance of spleen
10	Cancer,Condition	93505001	Hodgkin's disease, mixed cellularity of lymph nodes of axilla AND/OR upper limb
10	Cancer,Condition	93506000	Hodgkin's disease, mixed cellularity of lymph nodes of

© 2021 Matheny ME et al. *JAMA Network Open.*

CONDITIONID	CONDITION_DESCRIPTION	SNOMED_CODE	DESCRIPTION
			head, face AND/OR neck
10	Cancer,Condition	93507009	Hodgkin's disease, mixed cellularity of lymph nodes of inguinal region AND/OR lower limb
10	Cancer,Condition	93509007	Hodgkin's disease, mixed cellularity of spleen
10	Cancer,Condition	93514006	Hodgkin's disease, nodular sclerosis of lymph nodes of axilla AND/OR upper limb
10	Cancer,Condition	93515007	Hodgkin's disease, nodular sclerosis of lymph nodes of head, face AND/OR neck
10	Cancer,Condition	93516008	Hodgkin's disease, nodular sclerosis of lymph nodes of inguinal region AND/OR lower limb
10	Cancer,Condition	93518009	Hodgkin's disease, nodular sclerosis of spleen
10	Cancer,Condition	93520007	Hodgkin's disease of intra-abdominal lymph nodes
10	Cancer,Condition	93521006	Hodgkin's disease of intrapelvic lymph nodes
10	Cancer,Condition	93522004	Hodgkin's disease of intrathoracic lymph nodes
10	Cancer,Condition	93523009	Hodgkin's disease of lymph nodes of

© 2021 Matheny ME et al. *JAMA Network Open.*

CONDITIONID	CONDITION_DESCRIPTION	SNOMED_CODE	DESCRIPTION
			axilla AND/OR upper limb
10	Cancer,Condition	93524003	Hodgkin's disease of lymph nodes of head, face AND/OR neck
10	Cancer,Condition	93525002	Hodgkin's disease of lymph nodes of inguinal region AND/OR lower limb
10	Cancer,Condition	93526001	Hodgkin's disease of lymph nodes of multiple sites
10	Cancer,Condition	93527005	Hodgkin's disease of spleen
10	Cancer,Condition	93530003	Hodgkin's granuloma of intrapelvic lymph nodes
10	Cancer,Condition	93531004	Hodgkin's granuloma of intrathoracic lymph nodes
10	Cancer,Condition	93532006	Hodgkin's granuloma of lymph nodes of axilla AND/OR upper limb
10	Cancer,Condition	93533001	Hodgkin's granuloma of lymph nodes of head, face AND/OR neck
10	Cancer,Condition	93534007	Hodgkin's granuloma of lymph nodes of inguinal region AND/OR lower limb

© 2021 Matheny ME et al. *JAMA Network Open.*

CONDITIONID	CONDITION_DESCRIPTION	SNOMED_CODE	DESCRIPTION
10	Cancer,Condition	93536009	Hodgkin's granuloma of spleen
10	Cancer,Condition	93541001	Hodgkin's paragranuloma of lymph nodes of axilla AND/OR upper limb
10	Cancer,Condition	93542008	Hodgkin's paragranuloma of lymph nodes of head, face AND/OR neck
10	Cancer,Condition	93543003	Hodgkin's paragranuloma of lymph nodes of inguinal region AND/OR lower limb
10	Cancer,Condition	93545005	Hodgkin's paragranuloma of spleen
10	Cancer,Condition	93547002	Hodgkin's sarcoma of intra-abdominal lymph nodes
10	Cancer,Condition	93548007	Hodgkin's sarcoma of intrapelvic lymph nodes
10	Cancer,Condition	93549004	Hodgkin's sarcoma of intrathoracic lymph nodes
10	Cancer,Condition	93550004	Hodgkin's sarcoma of lymph nodes of axilla AND/OR upper limb
10	Cancer,Condition	93551000	Hodgkin's sarcoma of lymph nodes of head, face AND/OR neck
10	Cancer,Condition	93552007	Hodgkin's sarcoma of lymph nodes of

© 2021 Matheny ME et al. *JAMA Network Open.*

CONDITIONID	CONDITION_DESCRIPTION	SNOMED_CODE	DESCRIPTION
			inguinal region AND/OR lower limb
10	Cancer,Condition	93554008	Hodgkin's sarcoma of spleen
10	Cancer,Condition	93640008	Malignant melanoma of skin of lip
10	Cancer,Condition	93641007	Malignant melanoma of skin of lower limb
10	Cancer,Condition	93651008	Malignant melanoma of skin of trunk
10	Cancer,Condition	93653006	Malignant melanoma of skin of upper limb
10	Cancer,Condition	93655004	Malignant melanoma of skin
10	Cancer,Condition	93659005	Primary malignant neoplasm of accessory sinus
10	Cancer,Condition	93665005	Primary malignant neoplasm of adrenal gland
10	Cancer,Condition	93669004	Primary malignant neoplasm of anal canal
10	Cancer,Condition	93670003	Primary malignant neoplasm of anterior aspect of epiglottis
10	Cancer,Condition	93676009	Primary malignant neoplasm of anus
10	Cancer,Condition	93679002	Primary malignant neoplasm of appendix
10	Cancer,Condition	93687001	Primary malignant neoplasm of base of tongue

© 2021 Matheny ME et al. *JAMA Network Open.*

CONDITIONID	CONDITION_DESCRIPTION	SNOMED_CODE	DESCRIPTION
10	Cancer,Condition	93720005	Primary malignant neoplasm of bone marrow
10	Cancer,Condition	93726004	Primary malignant neoplasm of brain stem
10	Cancer,Condition	93738008	Primary malignant neoplasm of cardia of stomach
10	Cancer,Condition	93745008	Primary malignant neoplasm of central portion of female breast
10	Cancer,Condition	93746009	Primary malignant neoplasm of cerebellum
10	Cancer,Condition	93748005	Primary malignant neoplasm of cerebral ventricle
10	Cancer,Condition	93755007	Primary malignant neoplasm of choroid
10	Cancer,Condition	93761005	Primary malignant neoplasm of colon
10	Cancer,Condition	93764002	Primary malignant neoplasm of conjunctiva
10	Cancer,Condition	93766000	Primary malignant neoplasm of cornea
10	Cancer,Condition	93767009	Primary malignant neoplasm of cranial nerve
10	Cancer,Condition	93771007	Primary malignant neoplasm of descending colon
10	Cancer,Condition	93773005	Primary malignant neoplasm of dorsal surface of tongue

© 2021 Matheny ME et al. *JAMA Network Open.*

CONDITIONID	CONDITION_DESCRIPTION	SNOMED_CODE	DESCRIPTION
10	Cancer,Condition	93775003	Primary malignant neoplasm of duodenum
10	Cancer,Condition	93783009	Primary malignant neoplasm of epididymis
10	Cancer,Condition	93787005	Primary malignant neoplasm of ethmoidal sinus
10	Cancer,Condition	93796005	Primary malignant neoplasm of female breast
10	Cancer,Condition	93797001	Primary malignant neoplasm of female genital organ
10	Cancer,Condition	93802007	Primary malignant neoplasm of floor of mouth
10	Cancer,Condition	93808006	Primary malignant neoplasm of frontal sinus
10	Cancer,Condition	93818001	Primary malignant neoplasm of greater curvature of stomach
10	Cancer,Condition	93825008	Primary malignant neoplasm of heart
10	Cancer,Condition	93826009	Primary malignant neoplasm of hepatic flexure of colon
10	Cancer,Condition	93831006	Primary malignant neoplasm of hypopharynx
10	Cancer,Condition	93835002	Primary malignant neoplasm of inner aspect of lip
10	Cancer,Condition	93839008	Primary malignant neoplasm of intra-abdominal organs

© 2021 Matheny ME et al. *JAMA Network Open.*

CONDITIONID	CONDITION_DESCRIPTION	SNOMED_CODE	DESCRIPTION
10	Cancer,Condition	93841009	Primary malignant neoplasm of intrathoracic organs
10	Cancer,Condition	93844001	Primary malignant neoplasm of isthmus of uterus
10	Cancer,Condition	93846004	Primary malignant neoplasm of jejunum
10	Cancer,Condition	93848003	Primary malignant neoplasm of junctional zone of tongue
10	Cancer,Condition	93849006	Primary malignant neoplasm of kidney
10	Cancer,Condition	93861003	Primary malignant neoplasm of lateral wall of nasopharynx
10	Cancer,Condition	93867004	Primary malignant neoplasm of lesser curvature of stomach
10	Cancer,Condition	93868009	Primary malignant neoplasm of lingual tonsil
10	Cancer,Condition	93870000	Malignant neoplasm of liver
10	Cancer,Condition	93871001	Primary malignant neoplasm of long bone of lower limb
10	Cancer,Condition	93875005	Primary malignant neoplasm of lower limb
10	Cancer,Condition	93884005	Primary malignant neoplasm of male breast
10	Cancer,Condition	93885006	Primary malignant neoplasm of male genital organ

© 2021 Matheny ME et al. *JAMA Network Open.*

CONDITIONID	CONDITION_DESCRIPTION	SNOMED_CODE	DESCRIPTION
10	Cancer,Condition	93889000	Primary malignant neoplasm of maxillary sinus
10	Cancer,Condition	93891008	Primary malignant neoplasm of mediastinum
10	Cancer,Condition	93923002	Primary malignant neoplasm of nervous system
10	Cancer,Condition	93932000	Primary malignant neoplasm of orbit
10	Cancer,Condition	93933005	Primary malignant neoplasm of oropharynx
10	Cancer,Condition	93934004	Primary malignant neoplasm of ovary
10	Cancer,Condition	93941005	Primary malignant neoplasm of paraganglion
10	Cancer,Condition	93943008	Primary malignant neoplasm of parathyroid gland
10	Cancer,Condition	93944002	Primary malignant neoplasm of paraurethral glands
10	Cancer,Condition	93953009	Primary malignant neoplasm of pelvis
10	Cancer,Condition	93961004	Primary malignant neoplasm of pharynx
10	Cancer,Condition	93962006	Primary malignant neoplasm of pineal gland
10	Cancer,Condition	93966009	Primary malignant neoplasm of pleura
10	Cancer,Condition	93967000	Primary malignant neoplasm of postcricoid region
10	Cancer,Condition	93968005	Primary malignant neoplasm of posterior

© 2021 Matheny ME et al. *JAMA Network Open.*

CONDITIONID	CONDITION_DESCRIPTION	SNOMED_CODE	DESCRIPTION
			hypopharyngeal wall
10	Cancer,Condition	93974005	Primary malignant neoplasm of prostate
10	Cancer,Condition	93976007	Primary malignant neoplasm of pyloric antrum
10	Cancer,Condition	93977003	Primary malignant neoplasm of pylorus
10	Cancer,Condition	93978008	Primary malignant neoplasm of pyriform sinus
10	Cancer,Condition	93980002	Primary malignant neoplasm of rectosigmoid junction
10	Cancer,Condition	93984006	Primary malignant neoplasm of rectum
10	Cancer,Condition	93985007	Primary malignant neoplasm of renal pelvis
10	Cancer,Condition	93986008	Primary malignant neoplasm of respiratory tract
10	Cancer,Condition	93987004	Primary malignant neoplasm of retina
10	Cancer,Condition	93989001	Primary malignant neoplasm of retromolar area
10	Cancer,Condition	93994001	Primary malignant neoplasm of round ligament of uterus
10	Cancer,Condition	94003005	Primary malignant neoplasm of short bone of lower limb
10	Cancer,Condition	94004004	Primary malignant neoplasm of short bone of upper limb

© 2021 Matheny ME et al. *JAMA Network Open.*

CONDITIONID	CONDITION_DESCRIPTION	SNOMED_CODE	DESCRIPTION
10	Cancer,Condition	94006002	Primary malignant neoplasm of sigmoid colon
10	Cancer,Condition	94048009	Primary malignant neoplasm of small intestine
10	Cancer,Condition	94049001	Primary malignant neoplasm of soft palate
10	Cancer,Condition	94050001	Primary malignant neoplasm of soft tissues of abdomen
10	Cancer,Condition	94062002	Primary malignant neoplasm of soft tissues of thorax
10	Cancer,Condition	94063007	Primary malignant neoplasm of soft tissues of trunk
10	Cancer,Condition	94067008	Primary malignant neoplasm of sphenoidal sinus
10	Cancer,Condition	94068003	Primary malignant neoplasm of spinal cord
10	Cancer,Condition	94071006	Primary malignant neoplasm of spleen
10	Cancer,Condition	94072004	Primary malignant neoplasm of splenic flexure of colon
10	Cancer,Condition	94075002	Primary malignant neoplasm of subglottis
10	Cancer,Condition	94076001	Primary malignant neoplasm of sublingual gland
10	Cancer,Condition	94078000	Primary malignant neoplasm of superior wall of nasopharynx

© 2021 Matheny ME et al. *JAMA Network Open.*

CONDITIONID	CONDITION_DESCRIPTION	SNOMED_CODE	DESCRIPTION
10	Cancer,Condition	94087009	Primary malignant neoplasm of testis
10	Cancer,Condition	94096009	Primary malignant neoplasm of thymus
10	Cancer,Condition	94101009	Primary malignant neoplasm of tongue
10	Cancer,Condition	94102002	Primary malignant neoplasm of tonsillar fossa
10	Cancer,Condition	94103007	Primary malignant neoplasm of tonsillar pillar
10	Cancer,Condition	94104001	Primary malignant neoplasm of trachea
10	Cancer,Condition	94105000	Primary malignant neoplasm of transverse colon
10	Cancer,Condition	94116007	Primary malignant neoplasm of upper limb
10	Cancer,Condition	94118008	Primary malignant neoplasm of upper respiratory tract
10	Cancer,Condition	94120006	Primary malignant neoplasm of urachus
10	Cancer,Condition	94121005	Primary malignant neoplasm of ureter
10	Cancer,Condition	94123008	Primary malignant neoplasm of urethra
10	Cancer,Condition	94125001	Primary malignant neoplasm of urinary system
10	Cancer,Condition	94126000	Primary malignant neoplasm of uterine adnexa

© 2021 Matheny ME et al. *JAMA Network Open.*

CONDITIONID	CONDITION_DESCRIPTION	SNOMED_CODE	DESCRIPTION
10	Cancer,Condition	94129007	Primary malignant neoplasm of uvula
10	Cancer,Condition	94132005	Primary malignant neoplasm of vallecula
10	Cancer,Condition	94134006	Primary malignant neoplasm of ventral surface of tongue
10	Cancer,Condition	94135007	Primary malignant neoplasm of vermilion border of lip
10	Cancer,Condition	94143002	Primary malignant neoplasm of vulva
10	Cancer,Condition	94148006	Megakaryocytic leukemia in remission
10	Cancer,Condition	94704006	Multiple myeloma in remission
10	Cancer,Condition	94707004	Mycosis fungoides of intra-abdominal lymph nodes
10	Cancer,Condition	94708009	Mycosis fungoides of intrapelvic lymph nodes
10	Cancer,Condition	94709001	Mycosis fungoides of intrathoracic lymph nodes
10	Cancer,Condition	94710006	Mycosis fungoides of lymph nodes of axilla AND/OR upper limb
10	Cancer,Condition	94711005	Mycosis fungoides of lymph nodes of head, face AND/OR neck
10	Cancer,Condition	94712003	Mycosis fungoides of lymph nodes of inguinal region

© 2021 Matheny ME et al. *JAMA Network Open.*

CONDITIONID	CONDITION_DESCRIPTION	SNOMED_CODE	DESCRIPTION
			AND/OR lower limb
10	Cancer,Condition	94714002	Mycosis fungoides of spleen
10	Cancer,Condition	94715001	Mycosis fungoides of extranodal AND/OR solid organ site
10	Cancer,Condition	94716000	Myeloid leukemia in remission
10	Cancer,Condition	94718004	Myeloid sarcoma in remission
10	Cancer,Condition	94719007	Myeloid sarcoma
10	Cancer,Condition	95186006	Nodular lymphoma of intra-abdominal lymph nodes
10	Cancer,Condition	95187002	Nodular lymphoma of intrapelvic lymph nodes
10	Cancer,Condition	95188007	Nodular lymphoma of intrathoracic lymph nodes
10	Cancer,Condition	95192000	Nodular lymphoma of lymph nodes of multiple sites
10	Cancer,Condition	95193005	Nodular lymphoma of spleen
10	Cancer,Condition	95209008	Plasma cell leukemia in remission
10	Cancer,Condition	95210003	Plasma cell leukemia
10	Cancer,Condition	95224004	Reticulosarcoma of intra-abdominal lymph nodes
10	Cancer,Condition	95225003	Reticulosarcoma of intrapelvic lymph nodes
10	Cancer,Condition	95226002	Reticulosarcoma of intrathoracic lymph nodes

© 2021 Matheny ME et al. *JAMA Network Open.*

CONDITIONID	CONDITION_DESCRIPTION	SNOMED_CODE	DESCRIPTION
10	Cancer,Condition	95230004	Reticulosarcoma of lymph nodes of multiple sites
10	Cancer,Condition	95231000	Reticulosarcoma of spleen
10	Cancer,Condition	95260009	SÃ©zary's disease of lymph nodes of head, face AND/OR neck
10	Cancer,Condition	95261008	SÃ©zary's disease of lymph nodes of inguinal region AND/OR lower limb
10	Cancer,Condition	95263006	SÃ©zary's disease of spleen
10	Cancer,Condition	109267002	Overlapping malignant melanoma of skin
10	Cancer,Condition	109349007	Overlapping malignant neoplasm of soft tissues
10	Cancer,Condition	109357005	Primary malignant neoplasm of ill-defined site
10	Cancer,Condition	109366009	Overlapping malignant neoplasm of accessory sinuses
10	Cancer,Condition	109367000	Overlapping malignant neoplasm of nasopharynx
10	Cancer,Condition	109368005	Overlapping malignant neoplasm of hypopharynx
10	Cancer,Condition	109369002	Overlapping malignant neoplasm of larynx

© 2021 Matheny ME et al. *JAMA Network Open.*

CONDITIONID	CONDITION_DESCRIPTION	SNOMED_CODE	DESCRIPTION
10	Cancer,Condition	109370001	Primary malignant neoplasm of laryngeal cartilage
10	Cancer,Condition	109371002	Overlapping malignant neoplasm of bronchus and lung
10	Cancer,Condition	109374005	Overlapping malignant neoplasm of mediastinum and pleura
10	Cancer,Condition	109384006	Overlapping malignant neoplasm of heart, mediastinum and pleura
10	Cancer,Condition	109385007	Kaposi's sarcoma
10	Cancer,Condition	109386008	Kaposi's sarcoma of skin
10	Cancer,Condition	109388009	Kaposi's sarcoma of palate
10	Cancer,Condition	109389001	Kaposi's sarcoma of gastrointestinal tract
10	Cancer,Condition	109390005	Kaposi's sarcoma of lung
10	Cancer,Condition	109391009	Kaposi's sarcoma of lymph nodes
10	Cancer,Condition	109822001	Overlapping malignant neoplasm of lip
10	Cancer,Condition	109823006	Overlapping malignant neoplasm of tongue
10	Cancer,Condition	109824000	Overlapping malignant neoplasm of major salivary gland

© 2021 Matheny ME et al. *JAMA Network Open.*

CONDITIONID	CONDITION_DESCRIPTION	SNOMED_CODE	DESCRIPTION
10	Cancer,Condition	109828002	Primary malignant neoplasm of salivary gland duct
10	Cancer,Condition	109830000	Overlapping malignant neoplasm of floor of mouth
10	Cancer,Condition	109832008	Overlapping malignant neoplasm of oropharynx
10	Cancer,Condition	109835005	Overlapping malignant neoplasm of esophagus
10	Cancer,Condition	109836006	Overlapping malignant neoplasm of stomach
10	Cancer,Condition	109837002	Overlapping malignant neoplasm of small intestine
10	Cancer,Condition	109838007	Overlapping malignant neoplasm of colon
10	Cancer,Condition	109839004	Overlapping malignant neoplasm of rectum, anus and anal canal
10	Cancer,Condition	109847004	Overlapping malignant neoplasm of biliary tract
10	Cancer,Condition	109848009	Overlapping malignant neoplasm of pancreas
10	Cancer,Condition	109870007	Overlapping malignant

© 2021 Matheny ME et al. *JAMA Network Open.*

CONDITIONID	CONDITION_DESCRIPTION	SNOMED_CODE	DESCRIPTION
			neoplasm of urinary system
10	Cancer,Condition	109874003	Overlapping malignant neoplasm of male genital organs
10	Cancer,Condition	109878000	Overlapping malignant neoplasm of female genital organs
10	Cancer,Condition	109879008	Overlapping malignant neoplasm of body of uterus
10	Cancer,Condition	109886000	Overlapping malignant neoplasm of female breast
10	Cancer,Condition	109911004	Overlapping malignant neoplasm of brain and other parts of the central nervous system
10	Cancer,Condition	109948008	Overlapping malignant neoplasm of eye and adnexa
10	Cancer,Condition	109969005	Diffuse non-Hodgkin's lymphoma, large cell
10	Cancer,Condition	109977009	Peripheral T-cell lymphoma
10	Cancer,Condition	109979007	B-cell lymphoma
10	Cancer,Condition	109989006	Multiple myeloma
10	Cancer,Condition	109992005	Polycythemia vera
10	Cancer,Condition	118599009	Hodgkin's disease
10	Cancer,Condition	118600007	Malignant lymphoma

© 2021 Matheny ME et al. *JAMA Network Open.*

CONDITIONID	CONDITION_DESCRIPTION	SNOMED_CODE	DESCRIPTION
10	Cancer,Condition	118602004	Hodgkin's granuloma
10	Cancer,Condition	118605002	Hodgkin lymphoma, nodular lymphocyte predominance
10	Cancer,Condition	118606001	Hodgkin's sarcoma
10	Cancer,Condition	118607005	Lymphocyte-rich classical Hodgkin lymphoma
10	Cancer,Condition	118608000	Hodgkin's disease, nodular sclerosis
10	Cancer,Condition	118609008	Hodgkin's disease, mixed cellularity
10	Cancer,Condition	118610003	Hodgkin's disease, lymphocytic depletion
10	Cancer,Condition	118611004	SÃ©zary's disease
10	Cancer,Condition	118612006	Malignant histiocytosis
10	Cancer,Condition	118615008	Malignant mast cell tumor
10	Cancer,Condition	118617000	Burkitt's lymphoma
10	Cancer,Condition	118618005	Mycosis fungoides
10	Cancer,Condition	126667002	Neoplasm of respiratory tract
10	Cancer,Condition	126879004	Neoplasm of urinary system
10	Cancer,Condition	187606005	Malignant tumor of upper labial mucosa
10	Cancer,Condition	187637005	Malignant neoplasm of tongue, tip and lateral border
10	Cancer,Condition	187652003	Malignant tumor of anterior floor of mouth

© 2021 Matheny ME et al. *JAMA Network Open.*

CONDITIONID	CONDITION_DESCRIPTION	SNOMED_CODE	DESCRIPTION
10	Cancer,Condition	187653008	Malignant tumor of lateral floor of mouth
10	Cancer,Condition	187658004	Malignant tumor of vestibule of mouth
10	Cancer,Condition	187688008	Malignant tumor of posterior wall of oropharynx
10	Cancer,Condition	187692001	Malignant tumor of nasopharynx
10	Cancer,Condition	187693006	Malignant tumor of posterior wall of nasopharynx
10	Cancer,Condition	187700006	Malignant tumor of anterior wall of nasopharynx
10	Cancer,Condition	187708004	Malignant tumor aryepiglottic fold - hypopharyngeal aspect
10	Cancer,Condition	187716008	Malignant tumor of Waldeyer's ring
10	Cancer,Condition	187722004	Malignant tumor of cervical part of esophagus
10	Cancer,Condition	187723009	Malignant tumor of thoracic part of esophagus
10	Cancer,Condition	187724003	Malignant tumor of abdominal part of esophagus
10	Cancer,Condition	187725002	Malignant tumor of upper third of esophagus
10	Cancer,Condition	187726001	Malignant tumor of middle third of esophagus
10	Cancer,Condition	187727005	Malignant tumor of lower third of esophagus

© 2021 Matheny ME et al. *JAMA Network Open.*

CONDITIONID	CONDITION_DESCRIPTION	SNOMED_CODE	DESCRIPTION
10	Cancer,Condition	187741001	Malignant tumor of fundus of stomach
10	Cancer,Condition	187742008	Malignant tumor of body of stomach
10	Cancer,Condition	187752007	Malignant tumor of Meckel's diverticulum
10	Cancer,Condition	187767006	Malignant neoplasm of liver and intrahepatic bile ducts
10	Cancer,Condition	187791002	Malignant tumor of body of pancreas
10	Cancer,Condition	187792009	Malignant tumor of tail of pancreas
10	Cancer,Condition	187793004	Malignant tumor of pancreatic duct
10	Cancer,Condition	187794005	Malignant tumor of Islets of Langerhans
10	Cancer,Condition	187801002	Malignant tumor of peritoneum and retroperitoneum
10	Cancer,Condition	187808008	Malignant neoplasm of specified parts of peritoneum
10	Cancer,Condition	187828007	Malignant neoplasm of nasal cavities, middle ear and accessory sinuses
10	Cancer,Condition	187833006	Malignant neoplasm of auditory tube, middle ear and mastoid air cells
10	Cancer,Condition	187841006	Malignant tumor of glottis
10	Cancer,Condition	187842004	Malignant tumor of supraglottis

© 2021 Matheny ME et al. *JAMA Network Open.*

CONDITIONID	CONDITION_DESCRIPTION	SNOMED_CODE	DESCRIPTION
10	Cancer,Condition	187864008	Malignant neoplasm of middle lobe, bronchus or lung
10	Cancer,Condition	187868006	Malignant neoplasm of lower lobe, bronchus or lung
10	Cancer,Condition	187900002	Malignant neoplasm of bones of skull and face
10	Cancer,Condition	188019007	Malignant neoplasm of connective and soft tissue of pelvis
10	Cancer,Condition	188029000	Kaposi's sarcoma of soft tissue
10	Cancer,Condition	188032002	Malignant melanoma of ear and/or external auditory canal
10	Cancer,Condition	188044004	Malignant melanoma of scalp and/or neck
10	Cancer,Condition	188147009	Malignant neoplasm of nipple and areola of female breast
10	Cancer,Condition	188152004	Malignant neoplasm of upper-inner quadrant of female breast
10	Cancer,Condition	188153009	Malignant neoplasm of lower-inner quadrant of female breast
10	Cancer,Condition	188154003	Malignant neoplasm of upper-outer

© 2021 Matheny ME et al. *JAMA Network Open.*

CONDITIONID	CONDITION_DESCRIPTION	SNOMED_CODE	DESCRIPTION
			quadrant of female breast
10	Cancer,Condition	188155002	Malignant neoplasm of lower-outer quadrant of female breast
10	Cancer,Condition	188156001	Malignant neoplasm of axillary tail of female breast
10	Cancer,Condition	188163001	Malignant neoplasm of nipple and areola of male breast
10	Cancer,Condition	188189001	Malignant neoplasm of corpus uteri, excluding isthmus
10	Cancer,Condition	188219004	Malignant tumor of undescended testis
10	Cancer,Condition	188230001	Malignant tumor of body of penis
10	Cancer,Condition	188239000	Malignant tumor of trigone of urinary bladder
10	Cancer,Condition	188240003	Malignant tumor of vault of bladder
10	Cancer,Condition	188241004	Malignant neoplasm of lateral wall of urinary bladder
10	Cancer,Condition	188242006	Malignant neoplasm of anterior wall of urinary bladder
10	Cancer,Condition	188243001	Malignant neoplasm of posterior wall of urinary bladder
10	Cancer,Condition	188244007	Malignant tumor of bladder neck

© 2021 Matheny ME et al. *JAMA Network Open.*

CONDITIONID	CONDITION_DESCRIPTION	SNOMED_CODE	DESCRIPTION
10	Cancer,Condition	188245008	Malignant tumor of ureteric orifice
10	Cancer,Condition	188261005	Malignant neoplasm of eyeball excluding conjunctiva, cornea, retina and choroid
10	Cancer,Condition	188272000	Malignant tumor of lacrimal gland
10	Cancer,Condition	188274004	Malignant neoplasm of nasolacrimal duct
10	Cancer,Condition	188280007	Malignant neoplasm of cerebrum
10	Cancer,Condition	188339002	Malignant neoplasm of pituitary gland and craniopharyngeal duct
10	Cancer,Condition	188353002	Malignant neoplasm of head, neck and face
10	Cancer,Condition	188361007	Malignant neoplasm of thorax
10	Cancer,Condition	188366002	Malignant neoplasm of abdomen
10	Cancer,Condition	188487008	Lymphosarcoma and reticulosarcoma
10	Cancer,Condition	188489006	Reticulosarcoma of lymph nodes of head, face and neck
10	Cancer,Condition	188492005	Reticulosarcoma of lymph nodes of axilla and upper limb

© 2021 Matheny ME et al. *JAMA Network Open.*

CONDITIONID	CONDITION_DESCRIPTION	SNOMED_CODE	DESCRIPTION
10	Cancer,Condition	188493000	Reticulosarcoma of lymph nodes of inguinal region and lower limb
10	Cancer,Condition	188498009	Lymphosarcoma
10	Cancer,Condition	188500005	Lymphosarcoma of lymph nodes of head, face and neck
10	Cancer,Condition	188501009	Lymphosarcoma of intrathoracic lymph nodes
10	Cancer,Condition	188502002	Lymphosarcoma of intra-abdominal lymph nodes
10	Cancer,Condition	188503007	Lymphosarcoma of lymph nodes of axilla and upper limb
10	Cancer,Condition	188504001	Lymphosarcoma of lymph nodes of inguinal region and lower limb
10	Cancer,Condition	188505000	Lymphosarcoma of intrapelvic lymph nodes
10	Cancer,Condition	188506004	Lymphosarcoma of spleen
10	Cancer,Condition	188507008	Lymphosarcoma of lymph nodes of multiple sites
10	Cancer,Condition	188514005	Burkitt's lymphoma of lymph nodes of inguinal region and lower limb
10	Cancer,Condition	188515006	Burkitt's lymphoma of intrapelvic lymph nodes
10	Cancer,Condition	188517003	Burkitt's lymphoma of lymph nodes of multiple sites

© 2021 Matheny ME et al. *JAMA Network Open.*

CONDITIONID	CONDITION_DESCRIPTION	SNOMED_CODE	DESCRIPTION
10	Cancer,Condition	188524002	Hodgkin's paragranuloma of intrathoracic lymph nodes
10	Cancer,Condition	188526000	Hodgkin's paragranuloma of intra-abdominal lymph nodes
10	Cancer,Condition	188529007	Hodgkin's paragranuloma of intrapelvic lymph nodes
10	Cancer,Condition	188531003	Hodgkin's paragranuloma of lymph nodes of multiple sites
10	Cancer,Condition	188536008	Hodgkin's granuloma of intra-abdominal lymph nodes
10	Cancer,Condition	188541000	Hodgkin's granuloma of lymph nodes of multiple sites
10	Cancer,Condition	188551004	Hodgkin's sarcoma of lymph nodes of multiple sites
10	Cancer,Condition	188562004	Hodgkin's disease, lymphocytic-histiocytic predominance of lymph nodes of multiple sites
10	Cancer,Condition	188566001	Hodgkin's disease, nodular sclerosis of intrathoracic lymph nodes
10	Cancer,Condition	188567005	Hodgkin's disease, nodular sclerosis of intra-abdominal lymph nodes

© 2021 Matheny ME et al. *JAMA Network Open.*

CONDITIONID	CONDITION_DESCRIPTION	SNOMED_CODE	DESCRIPTION
10	Cancer,Condition	188570009	Hodgkin's disease, nodular sclerosis of intrapelvic lymph nodes
10	Cancer,Condition	188572001	Hodgkin's disease, nodular sclerosis of lymph nodes of multiple sites
10	Cancer,Condition	188576003	Hodgkin's disease, mixed cellularity of intrathoracic lymph nodes
10	Cancer,Condition	188577007	Hodgkin's disease, mixed cellularity of intra-abdominal lymph nodes
10	Cancer,Condition	188580008	Hodgkin's disease, mixed cellularity of intrapelvic lymph nodes
10	Cancer,Condition	188582000	Hodgkin's disease, mixed cellularity of lymph nodes of multiple sites
10	Cancer,Condition	188586002	Hodgkin's disease, lymphocytic depletion of intrathoracic lymph nodes
10	Cancer,Condition	188587006	Hodgkin's disease, lymphocytic depletion of intra-abdominal lymph nodes
10	Cancer,Condition	188591001	Hodgkin's disease, lymphocytic depletion of intrapelvic lymph nodes

© 2021 Matheny ME et al. *JAMA Network Open.*

CONDITIONID	CONDITION_DESCRIPTION	SNOMED_CODE	DESCRIPTION
10	Cancer,Condition	188592008	Hodgkin's disease, lymphocytic depletion of spleen
10	Cancer,Condition	188593003	Hodgkin's disease, lymphocytic depletion of lymph nodes of multiple sites
10	Cancer,Condition	188609000	Nodular lymphoma of lymph nodes of head, face and neck
10	Cancer,Condition	188612002	Nodular lymphoma of lymph nodes of axilla and upper limb
10	Cancer,Condition	188613007	Nodular lymphoma of lymph nodes of inguinal region and lower limb
10	Cancer,Condition	188627002	Mycosis fungoides of lymph nodes of multiple sites
10	Cancer,Condition	188631008	SÃ©zary's disease of intrathoracic lymph nodes
10	Cancer,Condition	188632001	SÃ©zary's disease of intra-abdominal lymph nodes
10	Cancer,Condition	188633006	SÃ©zary's disease of lymph nodes of axilla AND/OR upper limb
10	Cancer,Condition	188635004	SÃ©zary's disease of intrapelvic lymph nodes
10	Cancer,Condition	188637007	SÃ©zary's disease of lymph nodes of multiple sites
10	Cancer,Condition	188660004	Malignant mast cell tumors

© 2021 Matheny ME et al. *JAMA Network Open.*

CONDITIONID	CONDITION_DESCRIPTION	SNOMED_CODE	DESCRIPTION
10	Cancer,Condition	188664008	Mast cell malignancy of intra-abdominal lymph nodes
10	Cancer,Condition	188725004	Lymphoid leukemia
10	Cancer,Condition	188726003	Subacute lymphoid leukemia
10	Cancer,Condition	188732008	Myeloid leukemia
10	Cancer,Condition	188736006	Subacute myeloid leukemia
10	Cancer,Condition	188744006	Monocytic leukemia
10	Cancer,Condition	188745007	Chronic monocytic leukemia
10	Cancer,Condition	188746008	Subacute monocytic leukemia
10	Cancer,Condition	188754005	Megakaryocytic leukemia
10	Cancer,Condition	269464000	Malignant neoplasm of upper lobe, bronchus or lung
10	Cancer,Condition	269469005	Malignant neoplasm of soft tissue
10	Cancer,Condition	269476000	Nodular lymphoma
10	Cancer,Condition	271323007	Malignant neoplasm of lip, oral cavity and pharynx
10	Cancer,Condition	271568003	Malignant tumor of lower labial mucosa
10	Cancer,Condition	274905008	Malignant lymphoma - lymphocytic, intermediate differentiation
10	Cancer,Condition	275524009	Immunoproliferative neoplasm

© 2021 Matheny ME et al. *JAMA Network Open.*

CONDITIONID	CONDITION_DESCRIPTION	SNOMED_CODE	DESCRIPTION
10	Cancer,Condition	276819005	Carcinoid tumor of large intestine
10	Cancer,Condition	277626001	Diffuse high grade B-cell lymphoma
10	Cancer,Condition	277637000	Large cell anaplastic lymphoma
10	Cancer,Condition	302855005	Subacute leukemia
10	Cancer,Condition	302856006	Aleukemic leukemia
10	Cancer,Condition	307649006	Primary central nervous system lymphoma
10	Cancer,Condition	363350007	Malignant tumor of cecum
10	Cancer,Condition	363354003	Malignant tumor of cervix
10	Cancer,Condition	363372009	Malignant tumor of vermilion border of upper lip
10	Cancer,Condition	363373004	Malignant tumor of vermilion border of lower lip
10	Cancer,Condition	363378008	Malignant tumor of major salivary gland
10	Cancer,Condition	363380002	Malignant tumor of submandibular gland
10	Cancer,Condition	363385007	Malignant tumor of floor of mouth
10	Cancer,Condition	363402007	Malignant tumor of esophagus
10	Cancer,Condition	363405009	Malignant tumor of ileum
10	Cancer,Condition	363412000	Malignant tumor of ascending colon
10	Cancer,Condition	363420003	Malignant retroperitoneal tumor

© 2021 Matheny ME et al. *JAMA Network Open.*

CONDITIONID	CONDITION_DESCRIPTION	SNOMED_CODE	DESCRIPTION
10	Cancer,Condition	363422006	Malignant tumor of nasal cavity
10	Cancer,Condition	363443007	Malignant tumor of ovary
10	Cancer,Condition	363446004	Malignant neoplasm of labia majora
10	Cancer,Condition	363447008	Malignant neoplasm of labia minora
10	Cancer,Condition	363467004	Malignant neoplasm of frontal lobe
10	Cancer,Condition	363468009	Malignant neoplasm of temporal lobe
10	Cancer,Condition	363469001	Malignant neoplasm of parietal lobe
10	Cancer,Condition	363470000	Malignant neoplasm of occipital lobe
10	Cancer,Condition	363474009	Malignant neoplasm of cerebral meninges
10	Cancer,Condition	363476006	Malignant neoplasm of spinal meninges
10	Cancer,Condition	363505006	Malignant tumor of oral cavity
10	Cancer,Condition	363514001	Malignant tumor of female genital organ
10	Cancer,Condition	371967001	Primary malignant neoplasm of ampulla of Vater
10	Cancer,Condition	371968006	Primary malignant neoplasm of anterior two-thirds of tongue

© 2021 Matheny ME et al. *JAMA Network Open.*

CONDITIONID	CONDITION_DESCRIPTION	SNOMED_CODE	DESCRIPTION
10	Cancer,Condition	371970002	Primary malignant neoplasm of biliary tract
10	Cancer,Condition	371972005	Malignant neoplasm of body of uterus
10	Cancer,Condition	371973000	Malignant neoplasm of uterus
10	Cancer,Condition	371976008	Primary malignant neoplasm of buccal mucosa
10	Cancer,Condition	371979001	Malignant neoplasm of clitoris
10	Cancer,Condition	371981004	Primary malignant neoplasm of commissure of lip
10	Cancer,Condition	371983001	Primary malignant neoplasm of endocrine gland
10	Cancer,Condition	371984007	Primary malignant neoplasm of esophagus
10	Cancer,Condition	371986009	Primary malignant neoplasm of eye
10	Cancer,Condition	371987000	Primary malignant neoplasm of fallopian tube
10	Cancer,Condition	371989002	Primary malignant neoplasm of glans penis
10	Cancer,Condition	371990006	Primary malignant neoplasm of gum
10	Cancer,Condition	371991005	Primary malignant neoplasm of hard palate
10	Cancer,Condition	371992003	Primary malignant neoplasm of intestinal tract
10	Cancer,Condition	371995001	Primary malignant neoplasm of larynx

© 2021 Matheny ME et al. *JAMA Network Open.*

CONDITIONID	CONDITION_DESCRIPTION	SNOMED_CODE	DESCRIPTION
10	Cancer,Condition	371996000	Primary malignant neoplasm of lip
10	Cancer,Condition	371997009	Primary malignant neoplasm of lower gum
10	Cancer,Condition	372002009	Primary malignant neoplasm of palate
10	Cancer,Condition	372003004	Primary malignant neoplasm of pancreas
10	Cancer,Condition	372004005	Primary malignant neoplasm of parotid gland
10	Cancer,Condition	372005006	Primary malignant neoplasm of penis
10	Cancer,Condition	372006007	Primary malignant neoplasm of prepuce
10	Cancer,Condition	372009000	Primary malignant neoplasm of scrotum
10	Cancer,Condition	372010005	Primary malignant neoplasm of soft tissues
10	Cancer,Condition	372012002	Primary malignant neoplasm of soft tissues of upper limb
10	Cancer,Condition	372013007	Primary malignant neoplasm of spermatic cord
10	Cancer,Condition	372014001	Primary malignant neoplasm of stomach
10	Cancer,Condition	372016004	Primary malignant neoplasm of the peritoneum
10	Cancer,Condition	372020000	Primary malignant neoplasm of tonsil

© 2021 Matheny ME et al. *JAMA Network Open.*

CONDITIONID	CONDITION_DESCRIPTION	SNOMED_CODE	DESCRIPTION
10	Cancer,Condition	372022008	Primary malignant neoplasm of upper gum
10	Cancer,Condition	372024009	Primary malignant neoplasm of uterine cervix
10	Cancer,Condition	372025005	Primary malignant neoplasm of vagina
10	Cancer,Condition	372028007	Primary malignant neoplasm of vertebral column
10	Cancer,Condition	372065009	Malignant neoplasm of main bronchus
10	Cancer,Condition	372097009	Malignant neoplasm of endocervix
10	Cancer,Condition	372099007	Malignant neoplasm of exocervix
10	Cancer,Condition	372107001	Primary malignant neoplasm of ribs and/or sternum and/or clavicle
10	Cancer,Condition	372115003	Primary malignant neoplasm of pelvic bones, sacrum and coccyx
10	Cancer,Condition	372119009	Primary malignant neoplasm of head of pancreas
10	Cancer,Condition	372133009	Primary malignant neoplasm of upper limb bones and scapula
10	Cancer,Condition	372139008	Primary malignant neoplasm of gallbladder
10	Cancer,Condition	373168002	Reticulosarcoma
10	Cancer,Condition	399326009	Malignant tumor of urinary bladder

© 2021 Matheny ME et al. *JAMA Network Open.*

CONDITIONID	CONDITION_DESCRIPTION	SNOMED_CODE	DESCRIPTION
10	Cancer,Condition	413441006	Acute monocytic leukemia
10	Cancer,Condition	415111003	Plasma cell neoplasm
10	Cancer,Condition	415287001	Relapsing chronic myeloid leukemia
10	Cancer,Condition	426217000	Aleukemic leukemia in remission
10	Cancer,Condition	427056005	Subacute leukemia in remission
10	Cancer,Condition	428061005	Malignant neoplasm of brain
10	Cancer,Condition	428281000	Malignant neoplasm of bone
10	Cancer,Condition	428322007	Malignant neoplasm of uterine adnexa
10	Cancer,Condition	441559006	Mantle cell lymphoma of spleen
10	Cancer,Condition	441962003	Large cell lymphoma of intrapelvic lymph nodes
10	Cancer,Condition	443487006	Mantle cell lymphoma
10	Cancer,Condition	443488001	Malignant neoplasm of anorectum
10	Cancer,Condition	445269007	Extranodal marginal zone B-cell lymphoma of mucosa-associated lymphoid tissue
10	Cancer,Condition	446189008	Primary malignant neoplasm of extrahepatic bile duct
10	Cancer,Condition	447100004	Marginal zone lymphoma

© 2021 Matheny ME et al. *JAMA Network Open.*

CONDITIONID	CONDITION_DESCRIPTION	SNOMED_CODE	DESCRIPTION
10	Cancer,Condition	447109003	Primary malignant neoplasm of intrahepatic bile duct
10	Cancer,Condition	447883002	Malignant neoplasm of carotid body
10	Cancer,Condition	448668007	Malignant neoplasm of mandible
10	Cancer,Condition	448670003	Malignant neoplasm of posterior mediastinum
10	Cancer,Condition	448674007	Malignant neoplasm of parametrium
10	Cancer,Condition	448868009	Malignant neoplasm of lateral wall of oropharynx
10	Cancer,Condition	449067008	Malignant neoplasm of parietal pleura
10	Cancer,Condition	449224009	Malignant neoplasm of anterior mediastinum
10	Cancer,Condition	449259009	Malignant neoplasm of broad ligament of uterus
10	Cancer,Condition	449308006	Malignant neoplasm of visceral pleura
10	Cancer,Condition	609519004	Gestational trophoblastic neoplasia
11	Liver,Condition	1761006	Biliary cirrhosis
11	Liver,Condition	14223005	Esophageal varices without bleeding
11	Liver,Condition	17709002	Bleeding esophageal varices

© 2021 Matheny ME et al. *JAMA Network Open.*

CONDITIONID	CONDITION_DESCRIPTION	SNOMED_CODE	DESCRIPTION
11	Liver,Condition	34742003	Portal hypertension
11	Liver,Condition	41309000	Alcoholic liver damage
11	Liver,Condition	41889008	Chronic persistent hepatitis
11	Liver,Condition	50325005	Alcoholic fatty liver
11	Liver,Condition	76783007	Chronic hepatitis
11	Liver,Condition	79720007	Chronic nonalcoholic liver disease
11	Liver,Condition	128302006	Chronic hepatitis C
11	Liver,Condition	161671001	H/O: liver recipient
11	Liver,Condition	186624004	Acute hepatitis B with delta agent (coinfection) with hepatic coma
11	Liver,Condition	186628001	Viral hepatitis C with coma
11	Liver,Condition	195475003	Esophageal varices with bleeding, associated with another disorder
11	Liver,Condition	195476002	Esophageal varices without bleeding, associated with another disorder
11	Liver,Condition	235856003	Disease of liver
11	Liver,Condition	235869004	Chronic viral hepatitis B with hepatitis D
11	Liver,Condition	266468003	Cirrhosis - non-alcoholic
11	Liver,Condition	371067004	Hepatopulmonary syndrome
11	Liver,Condition	408335007	Autoimmune hepatitis
11	Liver,Condition	420054005	Alcoholic cirrhosis
11	Liver,Condition	424340000	Hepatic coma due to chronic hepatitis B

© 2021 Matheny ME et al. *JAMA Network Open.*

CONDITIONID	CONDITION_DESCRIPTION	SNOMED_CODE	DESCRIPTION
12	Peripheral Vascular Disease,Condition	3827008	Aneurysm of artery of neck
12	Peripheral Vascular Disease,Condition	13290008	Aneurysm of iliac artery
12	Peripheral Vascular Disease,Condition	14336007	Ruptured abdominal aortic aneurysm
12	Peripheral Vascular Disease,Condition	20981007	Aneurysm of artery of lower extremity
12	Peripheral Vascular Disease,Condition	29495008	Aneurysm of artery of upper extremity
12	Peripheral Vascular Disease,Condition	31529002	Thrombosis of arteries of upper extremity
12	Peripheral Vascular Disease,Condition	35239001	Aneurysm of visceral artery
12	Peripheral Vascular Disease,Condition	36184004	Aneurysm of renal artery
12	Peripheral Vascular Disease,Condition	39823006	Generalized atherosclerosis
12	Peripheral Vascular Disease,Condition	40136003	Aneurysm of subclavian artery
12	Peripheral Vascular Disease,Condition	45281005	Atherosclerosis of renal artery
12	Peripheral Vascular Disease,Condition	67362008	Aortic aneurysm
12	Peripheral Vascular Disease,Condition	70405009	Aneurysm of splenic artery
12	Peripheral Vascular Disease,Condition	72092001	Arteriosclerotic vascular disease
12	Peripheral Vascular Disease,Condition	73067008	Ruptured aortic aneurysm
12	Peripheral Vascular Disease,Condition	75878002	Abdominal aortic aneurysm without rupture
12	Peripheral Vascular Disease,Condition	81817003	Atherosclerosis of aorta
12	Peripheral Vascular Disease,Condition	90958004	Thrombosis of arteries of lower extremity
12	Peripheral Vascular Disease,Condition	161678007	H/O: artificial blood vessel

© 2021 Matheny ME et al. *JAMA Network Open.*

CONDITIONID	CONDITION_DESCRIPTION	SNOMED_CODE	DESCRIPTION
12	Peripheral Vascular Disease,Condition	195258006	Thoracic aortic aneurysm which has ruptured
12	Peripheral Vascular Disease,Condition	195265003	Thoracoabdominal aortic aneurysm, ruptured
12	Peripheral Vascular Disease,Condition	195318006	Embolism and thrombosis of an arm or leg artery
12	Peripheral Vascular Disease,Condition	233981004	Arterial aneurysm
12	Peripheral Vascular Disease,Condition	233984007	Thoracoabdominal aortic aneurysm
12	Peripheral Vascular Disease,Condition	233994002	Dissection of thoracic aorta
12	Peripheral Vascular Disease,Condition	308546005	Dissection of aorta
12	Peripheral Vascular Disease,Condition	408666009	Dissection of abdominal aorta
12	Peripheral Vascular Disease,Condition	433068007	Aneurysm of thoracic aorta
12	Peripheral Vascular Disease,Condition	441574008	Atherosclerosis of artery
12	Peripheral Vascular Disease,Condition	442439008	Atherosclerosis of bypass graft of limb
12	Peripheral Vascular Disease,Condition	443971004	Arteriosclerosis of artery of extremity
12	Peripheral Vascular Disease,Condition	713029000	Dissection of thoracoabdominal aorta
12	Peripheral Vascular Disease,Condition	145891000119104	Atherosclerosis of native arteries of the extremities
13	Myocardial Infarction,Condition	54329005	Acute myocardial infarction of anterior wall
13	Myocardial Infarction,Condition	57054005	Acute myocardial infarction
13	Myocardial Infarction,Condition	58612006	Acute myocardial infarction of lateral wall

© 2021 Matheny ME et al. *JAMA Network Open.*

CONDITIONID	CONDITION_DESCRIPTION	SNOMED_CODE	DESCRIPTION
13	Myocardial Infarction,Condition	65547006	Acute myocardial infarction of inferolateral wall
13	Myocardial Infarction,Condition	70211005	Acute myocardial infarction of anterolateral wall
13	Myocardial Infarction,Condition	70422006	Acute subendocardial infarction
13	Myocardial Infarction,Condition	73795002	Acute myocardial infarction of inferior wall
13	Myocardial Infarction,Condition	76593002	Acute myocardial infarction of inferoposterior wall
13	Myocardial Infarction,Condition	194802003	True posterior myocardial infarction
13	Myocardial Infarction,Condition	194809007	Acute myocardial infarction of atrium
14	Rhabdomyolysis,Condition	240131006	Rhabdomyolysis
15	Schistosomiasis,Condition	750009	Schistosoma mansoni infection
15	Schistosomiasis,Condition	10087007	Infection by Schistosoma
15	Schistosomiasis,Condition	60979006	Schistosoma haematobium infection
15	Schistosomiasis,Condition	187115002	Cutaneous schistosomiasis
15	Schistosomiasis,Condition	268058007	Schistosoma japonicum infection
16	Aspergillosis,Condition	37981002	Allergic bronchopulmonary aspergillosis
16	Aspergillosis,Condition	65553006	Aspergillosis
16	Aspergillosis,Condition	111900000	Pneumonia in aspergillosis
17	Oliguria,Condition	271845002	Oliguria and anuria

© 2021 Matheny ME et al. *JAMA Network Open.*

CONDITIONID	CONDITION_DESCRIPTION	SNOMED_CODE	DESCRIPTION
18	Pancreatitis,Condition	197456007	Acute pancreatitis
18	Pancreatitis,Condition	235494005	Chronic pancreatitis
19	Hypercalcemia,Condition	66931009	Hypercalcemia
20	Candidiasis,Condition	3487004	Candidiasis of lung
20	Candidiasis,Condition	16681000	Candidal otitis externa
20	Candidiasis,Condition	20639004	Candidiasis of the esophagus
20	Candidiasis,Condition	45021001	Candidal meningitis
20	Candidiasis,Condition	63553008	Candidal endocarditis
20	Candidiasis,Condition	70572005	Invasive candidiasis
20	Candidiasis,Condition	78048006	Candidiasis
20	Candidiasis,Condition	79740000	Candidiasis of mouth
20	Candidiasis,Condition	111904009	Candidiasis of urogenital site
20	Candidiasis,Condition	187014000	Candidiasis of skin and nails
20	Candidiasis,Condition	240706001	Candida infection of genital region
20	Candidiasis,Condition	426507006	Enteritis due to Candida
22	Burns,Condition	818005	Full thickness burn of multiple sites of lower limb
22	Burns,Condition	890002	Deep third degree burn of elbow
22	Burns,Condition	1203008	Deep third degree burn of forehead AND/OR cheek with loss of body part
22	Burns,Condition	1989002	Burn of vagina AND/OR uterus
22	Burns,Condition	2420007	Full thickness burn of multiple sites of upper limb

© 2021 Matheny ME et al. *JAMA Network Open.*

CONDITIONID	CONDITION_DESCRIPTION	SNOMED_CODE	DESCRIPTION
22	Burns,Condition	3461004	Deep third degree burn of thumb
22	Burns,Condition	3469002	Partial thickness burn of thumb
22	Burns,Condition	3480002	Burn of wrist
22	Burns,Condition	3975002	Deep third degree burn of lower limb
22	Burns,Condition	4300009	Deep third degree burn of forearm
22	Burns,Condition	4310000	Third degree burn of wrist AND/OR hand
22	Burns,Condition	5083008	Partial thickness burn of back
22	Burns,Condition	5414003	Burn any degree involving 80-89 percent of body surface
22	Burns,Condition	6055000	Burn of upper limb
22	Burns,Condition	6132001	Burn of thigh
22	Burns,Condition	6280008	Full thickness burn of wrist
22	Burns,Condition	6500006	Partial thickness burn of trunk
22	Burns,Condition	6772000	Burn with deep necrosis of underlying tissues with loss of body part
22	Burns,Condition	6821004	Partial thickness burn of neck
22	Burns,Condition	7765006	Epidermal burn of scalp
22	Burns,Condition	9063003	Epidermal burn of back
22	Burns,Condition	9269007	Epidermal burn of ear
22	Burns,Condition	9590000	Full thickness burn of hand
22	Burns,Condition	10132008	Burns of multiple sites

© 2021 Matheny ME et al. *JAMA Network Open.*

CONDITIONID	CONDITION_DESCRIPTION	SNOMED_CODE	DESCRIPTION
22	Burns,Condition	10137002	Epidermal burn of chin
22	Burns,Condition	10416004	Full thickness burn of lower leg
22	Burns,Condition	10815006	Full thickness burn of axilla
22	Burns,Condition	11406003	Partial thickness burn of nose
22	Burns,Condition	11868005	Burn of multiple sites of trunk
22	Burns,Condition	11980003	Burn of foot
22	Burns,Condition	13140001	Burn of gastrointestinal tract
22	Burns,Condition	13212004	Partial thickness burn of multiple sites
22	Burns,Condition	14020004	Partial thickness burn of multiple sites of upper arm
22	Burns,Condition	14261008	Burn of upper arm
22	Burns,Condition	14276000	Burn any degree involving 10-19 percent of body surface
22	Burns,Condition	14664006	Epidermal burn of hand
22	Burns,Condition	14893008	Burn of hand
22	Burns,Condition	15271003	Late effect of burn of wrist AND/OR hand
22	Burns,Condition	16096002	Chemical burn of eyelid AND/OR periocular area
22	Burns,Condition	16223008	Burn of scapular region
22	Burns,Condition	17543009	Burn of internal organ
22	Burns,Condition	17610001	Deep third degree burn of toe

© 2021 Matheny ME et al. *JAMA Network Open.*

CONDITIONID	CONDITION_DESCRIPTION	SNOMED_CODE	DESCRIPTION
22	Burns,Condition	17771000	Burn of face AND/OR head
22	Burns,Condition	18084001	Burn of elbow
22	Burns,Condition	19684007	Burn of knee
22	Burns,Condition	19856004	Late effect of burns of eye, face, head AND/OR neck
22	Burns,Condition	20490007	Epidermal burn of lower leg
22	Burns,Condition	20945009	Burn erythema of face AND/OR head
22	Burns,Condition	21835004	Disorder due to and following burn
22	Burns,Condition	22045003	Partial thickness burn of scalp
22	Burns,Condition	22233004	Burn of nose
22	Burns,Condition	22428001	Burn any degree involving 20-29 percent of body surface
22	Burns,Condition	23252003	Deep third degree burn of trunk with loss of body part
22	Burns,Condition	24379005	Burn of forehead AND/OR cheek
22	Burns,Condition	25515000	Second degree burn of face, head AND/OR neck
22	Burns,Condition	26729007	Full thickness burn of upper limb
22	Burns,Condition	26876007	Second degree burn of two OR more fingers not including thumb
22	Burns,Condition	27246003	Deep third degree burn of multiple sites of upper limb with loss of body part
22	Burns,Condition	27346001	Epidermal burn of ankle

© 2021 Matheny ME et al. *JAMA Network Open.*

CONDITIONID	CONDITION_DESCRIPTION	SNOMED_CODE	DESCRIPTION
22	Burns,Condition	28066004	Deep third degree burn of abdominal wall
22	Burns,Condition	28571002	Partial thickness burn of toe
22	Burns,Condition	28976004	Full thickness burn of scapular region
22	Burns,Condition	29418006	Epidermal burn of palm
22	Burns,Condition	29673001	Second degree burn of single finger, not thumb
22	Burns,Condition	29734005	Third degree burn of abdominal wall
22	Burns,Condition	30044002	Full thickness burn of foot
22	Burns,Condition	30264000	Full thickness burn of knee
22	Burns,Condition	31355000	Deep third degree burn of wrist
22	Burns,Condition	31577002	Partial thickness burn of palm
22	Burns,Condition	31946005	Burn of two OR more fingers not including thumb
22	Burns,Condition	33047005	Partial thickness burn of elbow
22	Burns,Condition	33824005	Burn erythema of face, head AND/OR neck
22	Burns,Condition	33826007	Epidermal burn of elbow
22	Burns,Condition	34036009	Deep third degree burn of palm
22	Burns,Condition	34384008	Second degree burn of chest wall
22	Burns,Condition	34554001	Epidermal burn of axilla
22	Burns,Condition	35501005	Epidermal burn of breast

© 2021 Matheny ME et al. *JAMA Network Open.*

CONDITIONID	CONDITION_DESCRIPTION	SNOMED_CODE	DESCRIPTION
22	Burns,Condition	35507009	Deep third degree burn of chest wall
22	Burns,Condition	35510002	Full thickness burn of back
22	Burns,Condition	35847008	Partial thickness burn of wrist
22	Burns,Condition	36121005	Deep third degree burn of axilla
22	Burns,Condition	36520004	Full thickness burn of ear
22	Burns,Condition	36830004	Third degree burn of breast
22	Burns,Condition	37022009	Deep third degree burn of hand with loss of body part
22	Burns,Condition	37645002	Burn of head AND/OR neck
22	Burns,Condition	37696000	Burn of lower leg
22	Burns,Condition	38916004	Alkaline chemical burn of cornea AND/OR conjunctival sac
22	Burns,Condition	38978009	Burn of scalp
22	Burns,Condition	39065001	Burn of ear
22	Burns,Condition	40261006	Full thickness burn of neck
22	Burns,Condition	40903003	Burn of palm
22	Burns,Condition	40904009	Burn any degree involving 30-39 percent of body surface
22	Burns,Condition	40986008	Epidermal burn of foot
22	Burns,Condition	40993007	Burn of ankle
22	Burns,Condition	41755001	Burn of two OR more fingers including thumb
22	Burns,Condition	42066004	Partial thickness burn of ear
22	Burns,Condition	42367004	Partial thickness burn of lower leg

© 2021 Matheny ME et al. *JAMA Network Open.*

CONDITIONID	CONDITION_DESCRIPTION	SNOMED_CODE	DESCRIPTION
22	Burns,Condition	42879007	Burn erythema of two OR more fingers including thumb
22	Burns,Condition	43138003	Second degree burn of wrist AND/OR hand
22	Burns,Condition	43635001	Epidermal burn of scapular region
22	Burns,Condition	44807006	Full thickness burn of back of hand
22	Burns,Condition	45267008	Deep third degree burn of back
22	Burns,Condition	45982008	Burn of esophagus
22	Burns,Condition	46000006	Burn any degree involving 70-79 percent of body surface
22	Burns,Condition	46156007	Burn erythema of chest wall
22	Burns,Condition	48260006	Deep third degree burn of scalp
22	Burns,Condition	49523001	Epidermal burn of multiple sites of upper limb
22	Burns,Condition	49615009	Burn erythema of abdominal wall
22	Burns,Condition	49862000	Full thickness burn of thigh
22	Burns,Condition	51041008	Burn any degree involving 60-69 percent of body surface
22	Burns,Condition	52405000	Burn of trunk
22	Burns,Condition	52899007	Partial thickness burn of lower limb
22	Burns,Condition	53080007	Partial thickness burn of upper limb
22	Burns,Condition	53106000	Burn of abdominal wall

© 2021 Matheny ME et al. *JAMA Network Open.*

CONDITIONID	CONDITION_DESCRIPTION	SNOMED_CODE	DESCRIPTION
22	Burns,Condition	53642007	Non-chemical burn of cornea AND/OR conjunctival sac
22	Burns,Condition	54296007	Partial thickness burn of hand
22	Burns,Condition	54674008	Burn erythema of forehead AND/OR cheek
22	Burns,Condition	54896004	Deep third degree burn of face AND/OR head with loss of body part
22	Burns,Condition	54975007	Second degree burn of face AND/OR head
22	Burns,Condition	55030004	Partial thickness burn of breast
22	Burns,Condition	55343006	Burn of back of hand
22	Burns,Condition	55473001	Full thickness burn of lower limb
22	Burns,Condition	56710004	Epidermal burn of wrist
22	Burns,Condition	56716005	Full thickness burn of scalp
22	Burns,Condition	57143002	Burn of shoulder
22	Burns,Condition	57266005	Second degree burn of two OR more fingers including thumb
22	Burns,Condition	57332004	Partial thickness burn of shoulder
22	Burns,Condition	57565008	Deep third degree burn of foot
22	Burns,Condition	58340008	Epidermal burn of nose
22	Burns,Condition	59110008	Deep third degree burn of toe with loss of body part
22	Burns,Condition	59448007	Third degree burn of two OR more

© 2021 Matheny ME et al. *JAMA Network Open.*

CONDITIONID	CONDITION_DESCRIPTION	SNOMED_CODE	DESCRIPTION
			fingers including thumb
22	Burns,Condition	59684006	Partial thickness burn of back of hand
22	Burns,Condition	60713008	Burn of neck
22	Burns,Condition	61455009	Third degree burn of face, head AND/OR neck
22	Burns,Condition	61984009	Deep third degree burn of hand
22	Burns,Condition	62011006	Deep third degree burn of two OR more fingers not including thumb
22	Burns,Condition	62242007	Epidermal burn of upper limb
22	Burns,Condition	62537000	Partial thickness burn of foot
22	Burns,Condition	62738008	Partial thickness burn of upper arm
22	Burns,Condition	62952004	Epidermal burn of knee
22	Burns,Condition	63053003	Deep third degree burn of knee
22	Burns,Condition	63420001	Deep third degree burn of two OR more fingers including thumb
22	Burns,Condition	63542002	Acid chemical burn of cornea AND/OR conjunctival sac
22	Burns,Condition	64090007	Burn of thumb
22	Burns,Condition	64359003	Full thickness burn of toe
22	Burns,Condition	64647005	Burn any degree involving 50-59 percent of body surface
22	Burns,Condition	65467000	Epidermal burn of forearm

© 2021 Matheny ME et al. *JAMA Network Open.*

CONDITIONID	CONDITION_DESCRIPTION	SNOMED_CODE	DESCRIPTION
22	Burns,Condition	66526003	Burn with resulting rupture AND/OR destruction of eyeball
22	Burns,Condition	66535005	Partial thickness burn of knee
22	Burns,Condition	67573002	Burn of multiple sites of upper limb
22	Burns,Condition	68140000	Partial thickness burn of thigh
22	Burns,Condition	68628004	Epidermal burn of toe
22	Burns,Condition	69025008	Epidermal burn of thumb
22	Burns,Condition	69429006	Burn erythema of two OR more fingers not including thumb
22	Burns,Condition	70038008	Deep third degree burn of finger, not thumb with loss of body part
22	Burns,Condition	70097001	Third degree burn of forehead AND/OR cheek
22	Burns,Condition	70250009	Epidermal burn of lower limb
22	Burns,Condition	70432004	Epidermal burn of upper arm
22	Burns,Condition	70505006	Partial thickness burn of forearm
22	Burns,Condition	71157005	Burn of eye AND/OR adnexa
22	Burns,Condition	71364004	Deep third degree burn of thigh
22	Burns,Condition	71637007	Burn of multiple sites of wrist AND/OR hand
22	Burns,Condition	72030007	Deep third degree burn of forehead AND/OR cheek

© 2021 Matheny ME et al. *JAMA Network Open.*

CONDITIONID	CONDITION_DESCRIPTION	SNOMED_CODE	DESCRIPTION
22	Burns,Condition	72759002	Deep third degree burn of shoulder
22	Burns,Condition	72882005	Epidermal burn of shoulder
22	Burns,Condition	72998004	Burn of back
22	Burns,Condition	73518002	Burn of lip
22	Burns,Condition	73545001	Full thickness burn of chin
22	Burns,Condition	73948007	Second degree burn of abdominal wall
22	Burns,Condition	74198004	Deep third degree burn of neck
22	Burns,Condition	74842002	Full thickness burn of trunk
22	Burns,Condition	75984003	Burn of chest wall
22	Burns,Condition	76867002	Full thickness burn of upper arm
22	Burns,Condition	76972007	Full thickness burn of thumb
22	Burns,Condition	77490007	Full thickness burn of shoulder
22	Burns,Condition	77830003	Epidermal burn of multiple sites
22	Burns,Condition	77890001	Late effect of burn of extremities, except wrist AND hand
22	Burns,Condition	78202006	Epidermal burn of lip
22	Burns,Condition	78506008	Third degree burn of two OR more fingers not including thumb
22	Burns,Condition	80027000	Second degree burn of forehead AND/OR cheek
22	Burns,Condition	80183002	Burn of multiple sites of lower limb
22	Burns,Condition	80210002	Burn with deep necrosis of

© 2021 Matheny ME et al. *JAMA Network Open.*

CONDITIONID	CONDITION_DESCRIPTION	SNOMED_CODE	DESCRIPTION
			underlying tissues without loss of body part
22	Burns,Condition	80271009	Full thickness burn of forearm
22	Burns,Condition	80433004	Burn of breast
22	Burns,Condition	80821000	Burn erythema of single finger, not thumb
22	Burns,Condition	80827001	Burn of forearm
22	Burns,Condition	81094005	Epidermal burn of thigh
22	Burns,Condition	81116005	Burn of chin
22	Burns,Condition	81152009	Partial thickness burn of scapular region
22	Burns,Condition	82057004	Burn of single finger, not thumb
22	Burns,Condition	83366000	Full thickness burns of multiple sites
22	Burns,Condition	84432000	Deep third degree burn of face, head AND/OR neck with loss of body part
22	Burns,Condition	84493007	Burn of axilla
22	Burns,Condition	84677008	Burn of lower limb
22	Burns,Condition	85052001	Full thickness burn of ankle
22	Burns,Condition	86051001	Deep third degree burn of multiple sites of lower limb with loss of body part
22	Burns,Condition	86089006	Partial thickness burn of axilla
22	Burns,Condition	86351009	Deep third degree burn of finger, not thumb
22	Burns,Condition	86660005	Burn any degree involving 40-49

© 2021 Matheny ME et al. *JAMA Network Open.*

CONDITIONID	CONDITION_DESCRIPTION	SNOMED_CODE	DESCRIPTION
			percent of body surface
22	Burns,Condition	87559001	Full thickness burn of elbow
22	Burns,Condition	88374002	Burn of toe
22	Burns,Condition	88756008	Partial thickness burn of chin
22	Burns,Condition	89266005	Partial thickness burn of ankle
22	Burns,Condition	89390009	Epidermal burn of multiple sites of lower limb
22	Burns,Condition	89614009	Third degree burn of nose
22	Burns,Condition	89661005	Full thickness burn of palm
22	Burns,Condition	89843004	Deep third degree burn of breast
22	Burns,Condition	90808005	Burn any degree involving 90 percent OR more of body surface
22	Burns,Condition	91146006	Epidermal burn of back of hand
22	Burns,Condition	111716006	Epidermal burn of trunk
22	Burns,Condition	111717002	Full thickness burn of chest wall
22	Burns,Condition	111718007	Burn erythema of multiple sites of wrist AND/OR hand
22	Burns,Condition	111719004	Deep third degree burn of wrist AND/OR hand
22	Burns,Condition	111720005	Partial thickness burn of multiple sites of lower limb
22	Burns,Condition	125666000	Burn
22	Burns,Condition	211718006	Full thickness burn of lip(s)

© 2021 Matheny ME et al. *JAMA Network Open.*

CONDITIONID	CONDITION_DESCRIPTION	SNOMED_CODE	DESCRIPTION
22	Burns,Condition	211720009	Full thickness burn of multiple sites of face, head or neck
22	Burns,Condition	211733003	Deep full thickness burn of multiple sites of face, head or neck without loss of body part
22	Burns,Condition	211737002	Deep full thickness burn of ear, with loss of body part
22	Burns,Condition	211740002	Deep full thickness burn of chin, with loss of body part
22	Burns,Condition	211742005	Deep full thickness burn of nose, with loss of body part
22	Burns,Condition	211769006	Epidermal burn of multiple sites of trunk
22	Burns,Condition	211787009	Partial thickness burn of multiple sites of trunk
22	Burns,Condition	211792006	Full thickness burn of genitalia
22	Burns,Condition	211810007	Deep full thickness burn of back, with loss of body part
22	Burns,Condition	211812004	Deep full thickness burn of the genitalia, with loss of body part
22	Burns,Condition	211853007	Deep full thickness burn of the arm without loss of body part
22	Burns,Condition	211857008	Deep full thickness burn of the upper arm without loss of body part

© 2021 Matheny ME et al. *JAMA Network Open.*

CONDITIONID	CONDITION_DESCRIPTION	SNOMED_CODE	DESCRIPTION
22	Burns,Condition	211861002	Deep full thickness burn of the scapular region without loss of body part
22	Burns,Condition	211862009	Deep full thickness burn of multiple sites of the arm without loss of body part
22	Burns,Condition	211864005	Deep full thickness burn of arm, with loss of body part
22	Burns,Condition	211866007	Deep full thickness burn of the forearm, with loss of body part
22	Burns,Condition	211867003	Deep full thickness burn of the elbow, with loss of body part
22	Burns,Condition	211868008	Deep full thickness burn of the upper arm, with loss of body part
22	Burns,Condition	211869000	Deep full thickness burn of the axilla, with loss of body part
22	Burns,Condition	211870004	Deep full thickness burn of the shoulder, with loss of body part
22	Burns,Condition	211871000	Deep full thickness burn of the scapular region, with loss of body part
22	Burns,Condition	211879003	Burn of wrist(s) and hand(s)

© 2021 Matheny ME et al. *JAMA Network Open.*

CONDITIONID	CONDITION_DESCRIPTION	SNOMED_CODE	DESCRIPTION
22	Burns,Condition	211919009	Full thickness burn of a single finger
22	Burns,Condition	211922006	Full thickness burn of multiple sites of wrist or hand
22	Burns,Condition	211931006	Deep full thickness burn of the back of hand without loss of body part
22	Burns,Condition	211933009	Deep full thickness burn of multiple sites of the wrist or hand without loss of body part
22	Burns,Condition	211938000	Deep full thickness burn of thumb, with loss of body part
22	Burns,Condition	211939008	Deep full thickness burn- of more than one finger with loss of body part
22	Burns,Condition	211940005	Deep full thickness burn of the thumb and finger(s) with loss of body part
22	Burns,Condition	211941009	Deep full thickness burn of the palm of hand with loss of body part
22	Burns,Condition	211942002	Deep full thickness burn of the back of hand with loss of body part
22	Burns,Condition	211943007	Deep full thickness burn of wrist, with loss of body part
22	Burns,Condition	211944001	Deep full thickness burn of multiple sites of the wrist or

© 2021 Matheny ME et al. *JAMA Network Open.*

CONDITIONID	CONDITION_DESCRIPTION	SNOMED_CODE	DESCRIPTION
			hand with loss of body part
22	Burns,Condition	211989000	Deep full thickness burn of the ankle without loss of body part
22	Burns,Condition	211990009	Deep full thickness burn of the lower leg without loss of body part
22	Burns,Condition	211993006	Deep full thickness burn of multiple sites of the leg without loss of body part
22	Burns,Condition	211995004	Deep full thickness burn of leg, with loss of body part
22	Burns,Condition	211998002	Deep full thickness burn of foot, with loss of body part
22	Burns,Condition	211999005	Deep full thickness burn of the ankle, with loss of body part
22	Burns,Condition	212000004	Deep full thickness burn of the lower leg, with loss of body part
22	Burns,Condition	212001000	Deep full thickness burn of knee, with loss of body part
22	Burns,Condition	212002007	Deep full thickness burn of the thigh, with loss of body part
22	Burns,Condition	212021004	Deep full thickness burn of multiple specified sites without loss of body part

© 2021 Matheny ME et al. *JAMA Network Open.*

CONDITIONID	CONDITION_DESCRIPTION	SNOMED_CODE	DESCRIPTION
22	Burns,Condition	212022006	Deep full thickness burn of multiple specified sites with loss of body part
22	Burns,Condition	212032004	Burn of mouth and pharynx
22	Burns,Condition	212037005	Burn of larynx, trachea and lung
22	Burns,Condition	212060002	Burn involving 20-29 percent of body surface, with 10-19 percent of body surface with full thickness burn
22	Burns,Condition	212061003	Burn involving 20-29 percent of body surface, with 20-29 percent of body surface with full thickness burn
22	Burns,Condition	212065007	Burn involving 30-39 percent of body surface, with 10-19 percent of body surface with full thickness burn
22	Burns,Condition	212066008	Burn involving 30-39 percent of body surface, with 20-29 percent of body surface with full thickness burn
22	Burns,Condition	212067004	Burn involving 30-39 percent of body surface, with 30-39 percent of body surface with full thickness burn
22	Burns,Condition	212071001	Burn involving 40-49 percent of body surface, with 10-19

© 2021 Matheny ME et al. *JAMA Network Open.*

CONDITIONID	CONDITION_DESCRIPTION	SNOMED_CODE	DESCRIPTION
			percent of body surface with full thickness burn
22	Burns,Condition	212072008	Burn involving 40-49 percent of body surface, with 20-29 percent of body surface with full thickness burn
22	Burns,Condition	212073003	Burn involving 40-49 percent of body surface, with 30-39 percent of body surface with full thickness burn
22	Burns,Condition	212074009	Burn involving 40-49 percent of body surface, with 40-49 percent of body surface with full thickness burn
22	Burns,Condition	212078007	Burn involving 50-59 percent of body surface, with 10-19 percent of body surface with full thickness burn
22	Burns,Condition	212079004	Burn involving 50-59 percent of body surface, with 20-29 percent of body surface with full thickness burn
22	Burns,Condition	212080001	Burn involving 50-59 percent of body surface, with 30-39 percent of body surface with full thickness burn

© 2021 Matheny ME et al. *JAMA Network Open.*

CONDITIONID	CONDITION_DESCRIPTION	SNOMED_CODE	DESCRIPTION
22	Burns,Condition	212081002	Burn involving 50-59 percent of body surface, with 40-49 percent of body surface with full thickness burn
22	Burns,Condition	212082009	Burn involving 50-59 percent of body surface, with 50-59 percent of body surface with full thickness burn
22	Burns,Condition	212086007	Burn involving 60-69 percent of body surface, with 10-19 percent of body surface with full thickness burn
22	Burns,Condition	212087003	Burn involving 60-69 percent of body surface, with 20-29 percent of body surface with full thickness burn
22	Burns,Condition	212088008	Burn involving 60-69 percent of body surface, with 30-39 percent of body surface with full thickness burn
22	Burns,Condition	212089000	Burn involving 60-69 percent of body surface, with 40-49 percent of body surface with full thickness burn
22	Burns,Condition	212090009	Burn involving 60-69 percent of body surface, with 50-59 percent of body

© 2021 Matheny ME et al. *JAMA Network Open.*

CONDITIONID	CONDITION_DESCRIPTION	SNOMED_CODE	DESCRIPTION
			surface with full thickness burn
22	Burns,Condition	212091008	Burn involving 60-69 percent of body surface, with 60-69 percent of body surface with full thickness burn
22	Burns,Condition	212095004	Burn involving 70-79 percent of body surface, with 10-19 percent of body surface with full thickness burn
22	Burns,Condition	212096003	Burn involving 70-79 percent of body surface, with 20-29 percent of body surface with full thickness burn
22	Burns,Condition	212097007	Burn involving 70-79 percent of body surface, with 30-39 percent of body surface with full thickness burn
22	Burns,Condition	212098002	Burn involving 70-79 percent of body surface, with 40-49 percent of body surface with full thickness burn
22	Burns,Condition	212099005	Burn involving 70-79 percent of body surface, with 50-59 percent of body surface with full thickness burn

© 2021 Matheny ME et al. *JAMA Network Open.*

CONDITIONID	CONDITION_DESCRIPTION	SNOMED_CODE	DESCRIPTION
22	Burns,Condition	212100002	Burn involving 70-79 percent of body surface, with 60-69 percent of body surface with full thickness burn
22	Burns,Condition	212101003	Burn involving 70-79 percent of body surface, with 70-79 percent of body surface with full thickness burn
22	Burns,Condition	212105007	Burn involving 80-89 percent of body surface, with 10-19 percent of body surface with full thickness burn
22	Burns,Condition	212106008	Burn involving 80-89 percent of body surface, with 20-29 percent of body surface with full thickness burn
22	Burns,Condition	212107004	Burn involving 80-89 percent of body surface, with 30-39 percent of body surface with full thickness burn
22	Burns,Condition	212108009	Burn involving 80-89 percent of body surface, with 40-49 percent of body surface with full thickness burn
22	Burns,Condition	212109001	Burn involving 80-89 percent of body surface, with 50-59 percent of body

© 2021 Matheny ME et al. *JAMA Network Open.*

CONDITIONID	CONDITION_DESCRIPTION	SNOMED_CODE	DESCRIPTION
			surface with full thickness burn
22	Burns,Condition	212110006	Burn involving 80-89 percent of body surface, with 60-69 percent of body surface with full thickness burn
22	Burns,Condition	212111005	Burn involving 80-89 percent of body surface, with 70-79 percent of body surface with full thickness burn
22	Burns,Condition	212112003	Burn involving 80-89 percent of body surface, with 80-89 percent of body surface with full thickness burn
22	Burns,Condition	212117009	Burn involving more than 90 percent of body surface, with 10-19 percent of body surface with full thickness burn
22	Burns,Condition	212118004	Burn involving more than 90 percent of body surface, with 20-29 percent of body surface with full thickness burn
22	Burns,Condition	212119007	Burn involving more than 90 percent of body surface, with 30-39 percent of body

© 2021 Matheny ME et al. *JAMA Network Open.*

CONDITIONID	CONDITION_DESCRIPTION	SNOMED_CODE	DESCRIPTION
			surface with full thickness burn
22	Burns,Condition	212120001	Burn involving more than 90 percent of body surface, with 40-49 percent of body surface with full thickness burn
22	Burns,Condition	212121002	Burn involving more than 90 percent of body surface, with 50-59 percent of body surface with full thickness burn
22	Burns,Condition	212122009	Burn involving more than 90 percent of body surface, with 60-69 percent of body surface with full thickness burn
22	Burns,Condition	212123004	Burn involving more than 90 percent of body surface, with 70-79 percent of body surface with full thickness burn
22	Burns,Condition	212124005	Burn involving more than 90 percent of body surface, with 80-89 percent of body surface with full thickness burn
22	Burns,Condition	212125006	Burn involving more than 90 percent of body

© 2021 Matheny ME et al. *JAMA Network Open.*

CONDITIONID	CONDITION_DESCRIPTION	SNOMED_CODE	DESCRIPTION
			surface, with more than 90 percent of body surface with full thickness burn
22	Burns,Condition	269232009	Burn confined to eye and adnexa
22	Burns,Condition	274205003	Burn of eye region
22	Burns,Condition	284205006	Partial thickness burn of lip
22	Burns,Condition	284210005	Multiple burns of head and neck
22	Burns,Condition	284220000	Burn of genitalia
22	Burns,Condition	403190006	Epidermal burn of skin
22	Burns,Condition	403191005	Partial thickness burn
22	Burns,Condition	403192003	Full thickness burn
23	Systemic Lupus Erythematosus,Condition	39367000	Inflammatory disease of the central nervous system
23	Systemic Lupus Erythematosus,Condition	52845002	Nephritis
23	Systemic Lupus Erythematosus,Condition	55464009	Systemic lupus erythematosus
23	Systemic Lupus Erythematosus,Condition	191287000	Hemorrhagic disorder due to circulating anticoagulants
23	Systemic Lupus Erythematosus,Condition	196131004	Lung involvement associated with another disorder
23	Systemic Lupus Erythematosus,Condition	197582001	Acute glomerulonephritis associated with another disorder
23	Systemic Lupus Erythematosus,Condition	197616000	Chronic glomerulonephritis associated with another disorder
23	Systemic Lupus Erythematosus,Condition	200936003	Lupus erythematosus

© 2021 Matheny ME et al. *JAMA Network Open.*

CONDITIONID	CONDITION_DESCRIPTION	SNOMED_CODE	DESCRIPTION
24	Sepsis,Condition	76571007	Septic shock
24	Sepsis,Condition	91302008	Sepsis
25	Leukemia,Condition	91854005	Acute leukemia in remission
25	Leukemia,Condition	91855006	Acute leukemia
25	Leukemia,Condition	91856007	Acute lymphoid leukemia in remission
25	Leukemia,Condition	91857003	Acute lymphoid leukemia
25	Leukemia,Condition	91858008	Acute monocytic leukemia in remission
25	Leukemia,Condition	91860005	Acute myeloid leukemia in remission
25	Leukemia,Condition	91861009	Acute myeloid leukemia, disease
25	Leukemia,Condition	92811003	Chronic leukemia in remission
25	Leukemia,Condition	92812005	Chronic leukemia
25	Leukemia,Condition	92813000	Chronic lymphoid leukemia in remission
25	Leukemia,Condition	92814006	Chronic lymphoid leukemia, disease
25	Leukemia,Condition	92817004	Chronic myeloid leukemia in remission
25	Leukemia,Condition	92818009	Chronic myeloid leukemia
25	Leukemia,Condition	93142004	Leukemia in remission
25	Leukemia,Condition	93143009	Leukemia
25	Leukemia,Condition	93144003	Leukemic reticuloendotheliosis of intra-abdominal lymph nodes
25	Leukemia,Condition	93145002	Leukemic reticuloendothelios

© 2021 Matheny ME et al. *JAMA Network Open.*

CONDITIONID	CONDITION_DESCRIPTION	SNOMED_CODE	DESCRIPTION
			is of intrapelvic lymph nodes
25	Leukemia,Condition	93146001	Leukemic reticuloendotheliosis of intrathoracic lymph nodes
25	Leukemia,Condition	93147005	Leukemic reticuloendotheliosis of lymph nodes of axilla AND/OR upper limb
25	Leukemia,Condition	93148000	Leukemic reticuloendotheliosis of lymph nodes of head, face AND/OR neck
25	Leukemia,Condition	93149008	Leukemic reticuloendotheliosis of lymph nodes of inguinal region AND/OR lower limb
25	Leukemia,Condition	93150008	Leukemic reticuloendotheliosis of lymph nodes of multiple sites
25	Leukemia,Condition	93151007	Hairy cell leukemia of spleen
25	Leukemia,Condition	93152000	Leukemic reticuloendotheliosis of extranodal AND/OR solid organ site
25	Leukemia,Condition	93169003	Lymphoid leukemia in remission
25	Leukemia,Condition	93450001	Erythroleukemia in remission
25	Leukemia,Condition	93451002	Erythroleukemia, FAB M6

© 2021 Matheny ME et al. *JAMA Network Open.*

CONDITIONID	CONDITION_DESCRIPTION	SNOMED_CODE	DESCRIPTION
25	Leukemia,Condition	94148006	Megakaryocytic leukemia in remission
25	Leukemia,Condition	94716000	Myeloid leukemia in remission
25	Leukemia,Condition	94718004	Myeloid sarcoma in remission
25	Leukemia,Condition	94719007	Myeloid sarcoma
25	Leukemia,Condition	95209008	Plasma cell leukemia in remission
25	Leukemia,Condition	95210003	Plasma cell leukemia
25	Leukemia,Condition	109992005	Polycythemia vera
25	Leukemia,Condition	188725004	Lymphoid leukemia
25	Leukemia,Condition	188726003	Subacute lymphoid leukemia
25	Leukemia,Condition	188732008	Myeloid leukemia
25	Leukemia,Condition	188736006	Subacute myeloid leukemia
25	Leukemia,Condition	188744006	Monocytic leukemia
25	Leukemia,Condition	188745007	Chronic monocytic leukemia
25	Leukemia,Condition	188746008	Subacute monocytic leukemia
25	Leukemia,Condition	188754005	Megakaryocytic leukemia
25	Leukemia,Condition	302855005	Subacute leukemia
25	Leukemia,Condition	302856006	Aleukemic leukemia
25	Leukemia,Condition	413441006	Acute monocytic leukemia
25	Leukemia,Condition	415287001	Relapsing chronic myeloid leukemia
25	Leukemia,Condition	417662000	History of clinical finding in subject
25	Leukemia,Condition	426217000	Aleukemic leukemia in remission

© 2021 Matheny ME et al. *JAMA Network Open.*

CONDITIONID	CONDITION_DESCRIPTION	SNOMED_CODE	DESCRIPTION
25	Leukemia,Condition	427056005	Subacute leukemia in remission
26	Multiple Myeloma,Condition	94704006	Multiple myeloma in remission
26	Multiple Myeloma,Condition	109989006	Multiple myeloma
27	Jaundice,Condition	17140000	Neonatal jaundice due to delayed conjugation
27	Jaundice,Condition	18165001	Jaundice
27	Jaundice,Condition	24911006	Perinatal jaundice from excessive hemolysis
27	Jaundice,Condition	56921004	Perinatal jaundice from hereditary hemolytic anemia
27	Jaundice,Condition	73749009	Neonatal jaundice associated with preterm delivery
27	Jaundice,Condition	206453006	Delayed conjugation causing neonatal jaundice associated with another disorder
27	Jaundice,Condition	206478005	Kernicterus not due to isoimmunization
27	Jaundice,Condition	387712008	Neonatal jaundice
28	Cardiogenic Shock,Condition	89138009	Cardiogenic shock
29	Diarrhea,Condition	19213003	Infectious diarrheal disease
29	Diarrhea,Condition	43240000	Diarrhea of presumed infectious origin
29	Diarrhea,Condition	47812002	Functional diarrhea
29	Diarrhea,Condition	62315008	Diarrhea
30	Intravascular Volume Disease,Condition	28560003	Hypovolemia
30	Intravascular Volume Disease,Condition	34095006	Dehydration
31	Tuburculosis,Condition	4445009	Tuberculosis of genitourinary system

© 2021 Matheny ME et al. *JAMA Network Open.*

CONDITIONID	CONDITION_DESCRIPTION	SNOMED_CODE	DESCRIPTION
31	Tuburculosis,Condition	10706006	Tuberculosis of central nervous system
31	Tuburculosis,Condition	14188007	Tuberculosis of hip
31	Tuburculosis,Condition	15284007	Tuberculosis of esophagus
31	Tuburculosis,Condition	17136009	Tuberculosis of knee
31	Tuburculosis,Condition	17653001	Tuberculosis of bones and/or joints
31	Tuburculosis,Condition	23022004	Tuberculous bronchiectasis
31	Tuburculosis,Condition	26935004	Tuberculosis of thyroid gland
31	Tuburculosis,Condition	28399005	Tuberculosis of spleen
31	Tuburculosis,Condition	29731002	Tuberculous pneumothorax
31	Tuburculosis,Condition	32268008	Tuberculosis of bladder
31	Tuburculosis,Condition	35786001	Tuberculoma of meninges
31	Tuburculosis,Condition	35984006	Tuberculosis of vertebral column
31	Tuburculosis,Condition	38279006	Tuberculosis of bone
31	Tuburculosis,Condition	44323002	Tuberculosis of kidney
31	Tuburculosis,Condition	44572005	Tuberculous peritonitis
31	Tuburculosis,Condition	47604008	Miliary tuberculosis
31	Tuburculosis,Condition	49107007	Tuberculosis of eye
31	Tuburculosis,Condition	56717001	Tuberculosis
31	Tuburculosis,Condition	58437007	Tuberculosis of meninges
31	Tuburculosis,Condition	63309002	Primary tuberculosis
31	Tuburculosis,Condition	70341005	Tuberculous laryngitis

© 2021 Matheny ME et al. *JAMA Network Open.*

CONDITIONID	CONDITION_DESCRIPTION	SNOMED_CODE	DESCRIPTION
31	Tuburculosis,Condition	71375000	Tuberculosis of mastoid process
31	Tuburculosis,Condition	74610006	Tuberculous erythema nodosum
31	Tuburculosis,Condition	77038006	Tuberculosis of peripheral lymph nodes
31	Tuburculosis,Condition	78436002	Tuberculosis of intrathoracic lymph nodes
31	Tuburculosis,Condition	80003002	Tuberculous pneumonia
31	Tuburculosis,Condition	80602006	Nodular tuberculosis of lung
31	Tuburculosis,Condition	81359005	Tuberculosis of ureter
31	Tuburculosis,Condition	83652003	Tuberculosis of epididymis
31	Tuburculosis,Condition	90117007	Tuberculous fibrosis of lung
31	Tuburculosis,Condition	111832004	Primary progressive tuberculosis
31	Tuburculosis,Condition	154283005	Pulmonary tuberculosis
31	Tuburculosis,Condition	185113005	Tuberculosis of adrenal glands
31	Tuburculosis,Condition	186172004	Tuberculous pleurisy in primary progressive tuberculosis
31	Tuburculosis,Condition	186175002	Infiltrative lung tuberculosis
31	Tuburculosis,Condition	186177005	Tuberculosis of lung with cavitation
31	Tuburculosis,Condition	186178000	Tuberculosis of bronchus

© 2021 Matheny ME et al. *JAMA Network Open.*

CONDITIONID	CONDITION_DESCRIPTION	SNOMED_CODE	DESCRIPTION
31	Tuburculosis,Condition	186182003	Tuberculosis of pleura
31	Tuburculosis,Condition	186193001	Tuberculosis of lung, confirmed by sputum microscopy with or without culture
31	Tuburculosis,Condition	186194007	Tuberculosis of lung, confirmed by culture only
31	Tuburculosis,Condition	186195008	Tuberculosis of lung, confirmed histologically
31	Tuburculosis,Condition	186202007	Respiratory tuberculosis, not confirmed bacteriologically or histologically
31	Tuburculosis,Condition	186204008	Tuberculosis of lung, bacteriological and histological examination not done
31	Tuburculosis,Condition	186217006	Tuberculous abscess of brain
31	Tuburculosis,Condition	186225008	Tuberculosis of intestines, peritoneum and mesenteric glands
31	Tuburculosis,Condition	186242002	Tuberculous oophoritis or salpingitis
31	Tuburculosis,Condition	186269001	Tuberculosis of ear
31	Tuburculosis,Condition	186276006	Acute miliary tuberculosis
31	Tuburculosis,Condition	187252008	Late effects of respiratory tuberculosis
31	Tuburculosis,Condition	187253003	Late effects of central nervous

© 2021 Matheny ME et al. *JAMA Network Open.*

CONDITIONID	CONDITION_DESCRIPTION	SNOMED_CODE	DESCRIPTION
			system tuberculosis
31	Tuburculosis,Condition	187254009	Late effects of genitourinary system tuberculosis
31	Tuburculosis,Condition	187255005	Late effects of tuberculosis of bones and/or joints
31	Tuburculosis,Condition	190710003	Tuberculous arthritis
31	Tuburculosis,Condition	198241002	Female tuberculous pelvic inflammatory disease
31	Tuburculosis,Condition	203267007	Tuberculosis of limb bones
31	Tuburculosis,Condition	236684001	Tuberculous urethritis
31	Tuburculosis,Condition	240379005	Tuberculosis of male genital organs
31	Tuburculosis,Condition	271423008	Tuberculosis of skin and subcutaneous tissue
31	Tuburculosis,Condition	416265003	Tuberculoma of brain
31	Tuburculosis,Condition	416903004	Tuberculoma of spinal cord
31	Tuburculosis,Condition	417484006	Tuberculous abscess of spinal cord
31	Tuburculosis,Condition	417662000	History of clinical finding in subject
31	Tuburculosis,Condition	700273003	Isolated tracheobronchial tuberculosis
32	Cirrhosis,Condition	266468003	Cirrhosis - non-alcoholic
32	Cirrhosis,Condition	420054005	Alcoholic cirrhosis
33	SIRS,Condition	91302008	Sepsis

© 2021 Matheny ME et al. *JAMA Network Open.*

CONDITIONID	CONDITION_DESCRIPTION	SNOMED_CODE	DESCRIPTION
33	SIRS,Condition	238149007	Systemic inflammatory response syndrome
33	SIRS,Condition	441596002	Systemic inflammatory response syndrome associated with organ dysfunction
34	Valvulopathy,Condition	7484005	Double outlet right ventricle
34	Valvulopathy,Condition	8722008	Aortic valve disorder
34	Valvulopathy,Condition	11851006	Mitral valve disorder
34	Valvulopathy,Condition	12023003	Rheumatic disease of aortic valve
34	Valvulopathy,Condition	13213009	Congenital heart disease
34	Valvulopathy,Condition	15459006	Endocardial cushion defect
34	Valvulopathy,Condition	17718000	Ostium primum defect
34	Valvulopathy,Condition	17759006	Rheumatic aortic stenosis with regurgitation
34	Valvulopathy,Condition	18546004	Congenital stenosis of aortic valve
34	Valvulopathy,Condition	18687009	Rheumatic disease of pulmonary valve
34	Valvulopathy,Condition	20721001	Tricuspid valve disorder
34	Valvulopathy,Condition	26146002	Complete transposition of great vessels
34	Valvulopathy,Condition	28656008	Congenital insufficiency of aortic valve

© 2021 Matheny ME et al. *JAMA Network Open.*

CONDITIONID	CONDITION_DESCRIPTION	SNOMED_CODE	DESCRIPTION
34	Valvulopathy,Condition	29928006	Congenital insufficiency of mitral valve
34	Valvulopathy,Condition	30288003	Ventricular septal defect
34	Valvulopathy,Condition	31085000	Rheumatic mitral regurgitation
34	Valvulopathy,Condition	36110001	Congenital anomaly of pulmonary artery
34	Valvulopathy,Condition	45503006	Common ventricle
34	Valvulopathy,Condition	48872007	Rheumatic endocarditis
34	Valvulopathy,Condition	56819008	Endocarditis
34	Valvulopathy,Condition	61959006	Common arterial trunk
34	Valvulopathy,Condition	62067003	Hypoplastic left heart syndrome
34	Valvulopathy,Condition	67278007	Congenital stenosis of pulmonary valve
34	Valvulopathy,Condition	72011007	Rheumatic aortic stenosis
34	Valvulopathy,Condition	73660006	Congenital subaortic stenosis
34	Valvulopathy,Condition	76267008	Pulmonary valve disorder
34	Valvulopathy,Condition	78031003	Rheumatic aortic regurgitation
34	Valvulopathy,Condition	79619009	Mitral valve stenosis
34	Valvulopathy,Condition	81990004	Cor biloculare
34	Valvulopathy,Condition	82458004	Congenital stenosis of mitral valve
34	Valvulopathy,Condition	83799000	Corrected transposition of great vessels
34	Valvulopathy,Condition	86299006	Tetralogy of Fallot
34	Valvulopathy,Condition	88610006	Heart murmur
34	Valvulopathy,Condition	123824001	Heart sounds abnormal

© 2021 Matheny ME et al. *JAMA Network Open.*

CONDITIONID	CONDITION_DESCRIPTION	SNOMED_CODE	DESCRIPTION
34	Valvulopathy,Condition	161667004	H/O: heart valve recipient
34	Valvulopathy,Condition	161677002	H/O: artificial heart valve
34	Valvulopathy,Condition	194726006	Mitral stenosis with insufficiency
34	Valvulopathy,Condition	194732001	Diseases of mitral and aortic valves
34	Valvulopathy,Condition	194733006	Mitral and aortic stenosis
34	Valvulopathy,Condition	194734000	Mitral stenosis and aortic insufficiency
34	Valvulopathy,Condition	194735004	Mitral insufficiency and aortic stenosis
34	Valvulopathy,Condition	194736003	Mitral and aortic incompetence
34	Valvulopathy,Condition	194737007	Multiple mitral and aortic valve involvement
34	Valvulopathy,Condition	194989009	Tricuspid valve disorder, non-rheumatic
34	Valvulopathy,Condition	195012000	Endocarditis associated with another disorder
34	Valvulopathy,Condition	204296002	Discordant ventriculoarterial connection
34	Valvulopathy,Condition	204315000	Ostium secundum type atrial septal defect
34	Valvulopathy,Condition	204339005	Congenital pulmonary valve abnormality
34	Valvulopathy,Condition	204342004	Congenital atresia of pulmonary valve
34	Valvulopathy,Condition	204354004	Congenital tricuspid atresia and stenosis
34	Valvulopathy,Condition	204357006	Ebstein's anomaly

© 2021 Matheny ME et al. *JAMA Network Open.*

CONDITIONID	CONDITION_DESCRIPTION	SNOMED_CODE	DESCRIPTION
34	Valvulopathy,Condition	204370002	Infundibular pulmonic stenosis
34	Valvulopathy,Condition	253273004	Cardiac septal defects
34	Valvulopathy,Condition	268174004	Bulbus cordis and cardiac septal closure anomalies
35	Arrhythmia,Condition	3424008	Tachycardia
35	Arrhythmia,Condition	4973001	Left bundle branch hemiblock
35	Arrhythmia,Condition	5370000	Atrial flutter
35	Arrhythmia,Condition	6374002	Bundle branch block
35	Arrhythmia,Condition	9651007	Long QT syndrome
35	Arrhythmia,Condition	12026006	Paroxysmal tachycardia
35	Arrhythmia,Condition	17869006	Anomalous atrioventricular excitation
35	Arrhythmia,Condition	20143001	Bilateral bundle branch block
35	Arrhythmia,Condition	27885002	Complete atrioventricular block
35	Arrhythmia,Condition	28189009	Mobitz type II atrioventricular block
35	Arrhythmia,Condition	29717002	Premature beats
35	Arrhythmia,Condition	30667004	Right bundle branch block AND left anterior fascicular block
35	Arrhythmia,Condition	34955007	Revision of automatic implantable cardioverter/defibrillator
35	Arrhythmia,Condition	44808001	Conduction disorder of the heart

© 2021 Matheny ME et al. *JAMA Network Open.*

CONDITIONID	CONDITION_DESCRIPTION	SNOMED_CODE	DESCRIPTION
35	Arrhythmia,Condition	46319007	Right bundle branch block AND left posterior fascicular block
35	Arrhythmia,Condition	46619002	Congenital heart block
35	Arrhythmia,Condition	49436004	Atrial fibrillation
35	Arrhythmia,Condition	55475008	Lown-Ganong-Levine syndrome
35	Arrhythmia,Condition	59118001	Right bundle branch block
35	Arrhythmia,Condition	60423000	Sinus node dysfunction
35	Arrhythmia,Condition	63467002	Left bundle branch block
35	Arrhythmia,Condition	63593006	Supraventricular premature beats
35	Arrhythmia,Condition	66657009	Paroxysmal ventricular tachycardia
35	Arrhythmia,Condition	67198005	Paroxysmal supraventricular tachycardia
35	Arrhythmia,Condition	71908006	Ventricular fibrillation
35	Arrhythmia,Condition	80313002	Palpitations
35	Arrhythmia,Condition	86014007	Trifascicular block
35	Arrhythmia,Condition	111288001	Ventricular flutter
35	Arrhythmia,Condition	161692001	H/O: cardiac pacemaker in situ
35	Arrhythmia,Condition	195042002	Second degree atrioventricular block
35	Arrhythmia,Condition	195080001	Atrial fibrillation and flutter
35	Arrhythmia,Condition	195083004	Ventricular fibrillation and flutter
35	Arrhythmia,Condition	233174007	Cardiac pacemaker procedure

© 2021 Matheny ME et al. *JAMA Network Open.*

CONDITIONID	CONDITION_DESCRIPTION	SNOMED_CODE	DESCRIPTION
35	Arrhythmia,Condition	233184008	Maintenance procedure for cardiac pacemaker system
35	Arrhythmia,Condition	233916004	Heart block
35	Arrhythmia,Condition	233917008	Atrioventricular block
35	Arrhythmia,Condition	270492004	First degree atrioventricular block
35	Arrhythmia,Condition	302116007	Finding of cardiovascular device
35	Arrhythmia,Condition	410429000	Cardiac arrest
35	Arrhythmia,Condition	413341007	Neonatal bradycardia
35	Arrhythmia,Condition	418341009	Atrioventricular conduction disorder
35	Arrhythmia,Condition	441509002	Cardiac pacemaker in situ
35	Arrhythmia,Condition	443325000	Automatic implantable cardiac defibrillator in situ
35	Arrhythmia,Condition	703398004	Cardiac implant in situ
36	Hepatitis,Condition	3738000	Viral hepatitis
36	Hepatitis,Condition	9953008	Acute alcoholic liver disease
36	Hepatitis,Condition	16060001	Hepatic coma due to viral hepatitis A
36	Hepatitis,Condition	17681007	Hepatitis due to acquired toxoplasmosis
36	Hepatitis,Condition	26206000	Hepatic coma due to viral hepatitis B
36	Hepatitis,Condition	40946000	Hepatic coma due to viral hepatitis
36	Hepatitis,Condition	41889008	Chronic persistent hepatitis

© 2021 Matheny ME et al. *JAMA Network Open.*

CONDITIONID	CONDITION_DESCRIPTION	SNOMED_CODE	DESCRIPTION
36	Hepatitis,Condition	50711007	Viral hepatitis C
36	Hepatitis,Condition	72836002	Hepatic coma
36	Hepatitis,Condition	76783007	Chronic hepatitis
36	Hepatitis,Condition	89231008	Mumps hepatitis
36	Hepatitis,Condition	111879004	Viral hepatitis A without hepatic coma
36	Hepatitis,Condition	111891008	Viral hepatitis B without hepatic coma
36	Hepatitis,Condition	111896003	Viral hepatitis without hepatic coma
36	Hepatitis,Condition	128241005	Inflammatory disease of liver
36	Hepatitis,Condition	128302006	Chronic hepatitis C
36	Hepatitis,Condition	170489004	Viral hepatitis carrier
36	Hepatitis,Condition	170536002	Vaccination required
36	Hepatitis,Condition	186624004	Acute hepatitis B with delta agent (coinfection) with hepatic coma
36	Hepatitis,Condition	186626002	Acute hepatitis B with delta-agent (coinfection) without hepatic coma
36	Hepatitis,Condition	186628001	Viral hepatitis C with coma
36	Hepatitis,Condition	186639003	Chronic viral hepatitis B without delta-agent
36	Hepatitis,Condition	197348008	Hepatitis in secondary syphilis
36	Hepatitis,Condition	235862008	Hepatitis due to infection
36	Hepatitis,Condition	235865005	Hepatitis D superinfection of hepatitis B carrier

© 2021 Matheny ME et al. *JAMA Network Open.*

CONDITIONID	CONDITION_DESCRIPTION	SNOMED_CODE	DESCRIPTION
36	Hepatitis,Condition	235866006	Acute hepatitis C
36	Hepatitis,Condition	235867002	Acute hepatitis E
36	Hepatitis,Condition	235869004	Chronic viral hepatitis B with hepatitis D
36	Hepatitis,Condition	235871004	Hepatitis B carrier
36	Hepatitis,Condition	235872006	Hepatitis C carrier
36	Hepatitis,Condition	406584008	Non-A, non-B, non-C hepatitis
36	Hepatitis,Condition	408335007	Autoimmune hepatitis
36	Hepatitis,Condition	424340000	Hepatic coma due to chronic hepatitis B
37	Injury-Trauma,Condition	171008	Injury of ascending right colon without open wound into abdominal cavity
37	Injury-Trauma,Condition	188001	Injury of intercostal artery
37	Injury-Trauma,Condition	496002	Closed traumatic dislocation of third cervical vertebra
37	Injury-Trauma,Condition	658009	Injury of colon without open wound into abdominal cavity
37	Injury-Trauma,Condition	845006	Injury of inferior mesenteric artery
37	Injury-Trauma,Condition	1264004	Injury of descending left colon without open wound into abdominal cavity
37	Injury-Trauma,Condition	1351002	Injury of iliac artery
37	Injury-Trauma,Condition	1367008	Injury of superior mesenteric artery
37	Injury-Trauma,Condition	1430001	Intracranial hemorrhage following injury without open

© 2021 Matheny ME et al. *JAMA Network Open.*

CONDITIONID	CONDITION_DESCRIPTION	SNOMED_CODE	DESCRIPTION
			intracranial wound AND with prolonged loss of consciousness (more than 24 hours) without return to pre-existing level
37	Injury-Trauma,Condition	1544005	Open dislocation of knee
37	Injury-Trauma,Condition	1988005	Late effect of injury to nerve roots, spinal plexus AND/OR other nerves of trunk
37	Injury-Trauma,Condition	2053005	Late effect of injury to blood vessels of thorax, abdomen AND/OR pelvis
37	Injury-Trauma,Condition	2366001	Late effect of dislocation
37	Injury-Trauma,Condition	2591000	Crushing injury of shoulder region
37	Injury-Trauma,Condition	2651006	Closed traumatic dislocation of elbow joint
37	Injury-Trauma,Condition	2840008	Open fracture of vault of skull with cerebral laceration AND/OR contusion
37	Injury-Trauma,Condition	2973002	Pelvic organ injury without open wound into abdominal cavity
37	Injury-Trauma,Condition	3019000	Closed anterior dislocation of elbow
37	Injury-Trauma,Condition	3119002	Brain stem laceration with open intracranial

© 2021 Matheny ME et al. *JAMA Network Open.*

CONDITIONID	CONDITION_DESCRIPTION	SNOMED_CODE	DESCRIPTION
			wound AND loss of consciousness
37	Injury-Trauma,Condition	3217005	Open dislocation of sixth cervical vertebra
37	Injury-Trauma,Condition	3446000	Open fracture of T7-T12 level with spinal cord injury
37	Injury-Trauma,Condition	3759007	Injury of heart with open wound into thorax
37	Injury-Trauma,Condition	3903005	Traumatic pneumothorax without open wound into thorax
37	Injury-Trauma,Condition	3913002	Injury of gastrointestinal tract with open wound into abdominal cavity
37	Injury-Trauma,Condition	4260009	Sacral spinal cord injury without bone injury
37	Injury-Trauma,Condition	4273008	Closed posterior dislocation of elbow
37	Injury-Trauma,Condition	4332009	Subarachnoid hemorrhage following injury without open intracranial wound AND with concussion
37	Injury-Trauma,Condition	4975008	Crushing injury of trunk
37	Injury-Trauma,Condition	5073009	Brain stem laceration with open intracranial wound AND prolonged loss of consciousness

© 2021 Matheny ME et al. *JAMA Network Open.*

CONDITIONID	CONDITION_DESCRIPTION	SNOMED_CODE	DESCRIPTION
			(more than 24 hours) AND return to pre-existing conscious level
37	Injury-Trauma,Condition	5120006	Cortex laceration without open intracranial wound AND with concussion
37	Injury-Trauma,Condition	5137004	Recurrent dislocation of joint
37	Injury-Trauma,Condition	5166001	Injury of external jugular vein
37	Injury-Trauma,Condition	5202009	Brain injury with open intracranial wound AND concussion
37	Injury-Trauma,Condition	5251007	Subarachnoid hemorrhage following injury with open intracranial wound
37	Injury-Trauma,Condition	5256002	Injury of spleen with open wound into abdominal cavity
37	Injury-Trauma,Condition	5313005	Derangement of posterior horn of medial meniscus
37	Injury-Trauma,Condition	5450005	Crushing injury of elbow
37	Injury-Trauma,Condition	5753006	Injury of renal vein
37	Injury-Trauma,Condition	5886004	Cortex contusion without open intracranial wound AND with no loss of consciousness
37	Injury-Trauma,Condition	5949004	Open dislocation of tarsal joint

© 2021 Matheny ME et al. *JAMA Network Open.*

CONDITIONID	CONDITION_DESCRIPTION	SNOMED_CODE	DESCRIPTION
37	Injury-Trauma,Condition	6015002	Crushing injury of multiple sites of upper limb
37	Injury-Trauma,Condition	6147005	Brain stem laceration with open intracranial wound AND brief loss of consciousness
37	Injury-Trauma,Condition	6342009	Injury at C1-C4 level with central cord syndrome AND without bone injury
37	Injury-Trauma,Condition	6378004	Injury of multiple blood vessels of head AND/OR neck
37	Injury-Trauma,Condition	6666006	Subdural hemorrhage following injury without open intracranial wound AND with concussion
37	Injury-Trauma,Condition	6715005	Injury of external carotid artery
37	Injury-Trauma,Condition	6956001	Injury of carotid artery
37	Injury-Trauma,Condition	7346000	Injury of inferior mesenteric vein
37	Injury-Trauma,Condition	7369002	Injury of palmar artery
37	Injury-Trauma,Condition	7711006	Cortex laceration without open intracranial wound AND with prolonged loss of consciousness (more than 24 hours) without return to pre-

© 2021 Matheny ME et al. *JAMA Network Open.*

CONDITIONID	CONDITION_DESCRIPTION	SNOMED_CODE	DESCRIPTION
			existing conscious level
37	Injury-Trauma,Condition	7819003	Intracranial hemorrhage following injury with open intracranial wound AND no loss of consciousness
37	Injury-Trauma,Condition	7862002	Prolonged loss of consciousness
37	Injury-Trauma,Condition	8135006	Traumatic closed dislocation of temporomandibular joint
37	Injury-Trauma,Condition	8183009	Closed fracture of T7-T12 level with central cord syndrome
37	Injury-Trauma,Condition	8281003	Injury of uterine artery
37	Injury-Trauma,Condition	8328009	Late effect of injury to blood vessels of head, neck AND/OR extremities
37	Injury-Trauma,Condition	8840000	Closed fracture of C1-C4 level with spinal cord injury
37	Injury-Trauma,Condition	9051008	Articular cartilage disorder of upper arm
37	Injury-Trauma,Condition	9298006	Hematoma of kidney without rupture of capsule AND without open wound into abdominal cavity

© 2021 Matheny ME et al. *JAMA Network Open.*

CONDITIONID	CONDITION_DESCRIPTION	SNOMED_CODE	DESCRIPTION
37	Injury-Trauma,Condition	9337000	Recurrent dislocation of multiple sites
37	Injury-Trauma,Condition	9560007	Crushing injury of upper limb
37	Injury-Trauma,Condition	9771006	Derangement of anterior horn of medial meniscus
37	Injury-Trauma,Condition	9787009	Closed lateral dislocation of proximal end of tibia
37	Injury-Trauma,Condition	9978007	Cortex laceration without open intracranial wound AND with loss of consciousness
37	Injury-Trauma,Condition	10380004	Crushing injury of finger
37	Injury-Trauma,Condition	10392004	Injury of abdominal aorta
37	Injury-Trauma,Condition	10464008	Recurrent dislocation of hand
37	Injury-Trauma,Condition	10575009	Open fracture of vertebral column with spinal cord injury
37	Injury-Trauma,Condition	10771002	Closed traumatic dislocation of first cervical vertebra
37	Injury-Trauma,Condition	11008006	Closed traumatic dislocation of seventh cervical vertebra
37	Injury-Trauma,Condition	11229008	Laceration of heart without penetration of heart chambers
37	Injury-Trauma,Condition	11730002	Crushing injury of hip

© 2021 Matheny ME et al. *JAMA Network Open.*

CONDITIONID	CONDITION_DESCRIPTION	SNOMED_CODE	DESCRIPTION
37	Injury-Trauma,Condition	11807002	Injury at C5-C7 level with spinal cord injury AND without bone injury
37	Injury-Trauma,Condition	11920000	Open dislocation of elbow
37	Injury-Trauma,Condition	12227007	Capsular tear without major disruption of parenchyma of spleen AND without open wound into abdominal cavity
37	Injury-Trauma,Condition	12462000	Closed traumatic dislocation of vertebra
37	Injury-Trauma,Condition	12589008	Brain stem laceration with open intracranial wound
37	Injury-Trauma,Condition	12912004	Brain injury with open intracranial wound AND no loss of consciousness
37	Injury-Trauma,Condition	13019000	Open posterior dislocation of proximal end of tibia
37	Injury-Trauma,Condition	13194006	Closed fracture of T7-T12 level with anterior cord syndrome
37	Injury-Trauma,Condition	13289004	Intracranial hemorrhage following injury with open intracranial wound AND loss of consciousness

© 2021 Matheny ME et al. *JAMA Network Open.*

CONDITIONID	CONDITION_DESCRIPTION	SNOMED_CODE	DESCRIPTION
37	Injury-Trauma,Condition	13463001	Open dislocation of fifth cervical vertebra
37	Injury-Trauma,Condition	13752003	Brain injury with open intracranial wound AND loss of consciousness
37	Injury-Trauma,Condition	13891000	Major laceration of liver without open wound into abdominal cavity
37	Injury-Trauma,Condition	14180000	Injury of splenic vein
37	Injury-Trauma,Condition	14434006	Cerebellar laceration with open intracranial wound AND no loss of consciousness
37	Injury-Trauma,Condition	14493003	Closed fracture of T1-T6 level with anterior cord syndrome
37	Injury-Trauma,Condition	14584002	Closed fracture of sacrum AND/OR coccyx with spinal cord injury
37	Injury-Trauma,Condition	14741001	Closed posterior dislocation of proximal end of tibia
37	Injury-Trauma,Condition	14949009	Old disruption of ligament of knee
37	Injury-Trauma,Condition	15151004	Injury of liver with open wound into abdominal cavity
37	Injury-Trauma,Condition	15531007	Injury of popliteal artery
37	Injury-Trauma,Condition	15971009	Injury of anterior tibial vein
37	Injury-Trauma,Condition	16319002	Cortex contusion without open

© 2021 Matheny ME et al. *JAMA Network Open.*

CONDITIONID	CONDITION_DESCRIPTION	SNOMED_CODE	DESCRIPTION
			intracranial wound AND with concussion
37	Injury-Trauma,Condition	16695002	Open fracture of T1-T6 level with spinal cord injury
37	Injury-Trauma,Condition	16837005	Brain stem contusion with open intracranial wound AND prolonged loss of consciousness (more than 24 hours) AND return to pre-existing conscious level
37	Injury-Trauma,Condition	16907002	Extradural hemorrhage following injury without open intracranial wound AND with prolonged loss of consciousness (more than 24 hours) without return to pre-existing conscious level
37	Injury-Trauma,Condition	17290007	Open dislocation of sternum
37	Injury-Trauma,Condition	17414004	Contusion to heart
37	Injury-Trauma,Condition	17498002	Cortex contusion without open intracranial wound AND with loss of consciousness
37	Injury-Trauma,Condition	17567001	Injury of hepatic artery

© 2021 Matheny ME et al. *JAMA Network Open.*

CONDITIONID	CONDITION_DESCRIPTION	SNOMED_CODE	DESCRIPTION
37	Injury-Trauma,Condition	17667005	Intracranial hemorrhage following injury without open intracranial wound AND with brief loss of consciousness
37	Injury-Trauma,Condition	18147000	Injury of colon with open wound into abdominal cavity
37	Injury-Trauma,Condition	18296004	Injury of ovarian artery
37	Injury-Trauma,Condition	18485009	Intracranial hemorrhage following injury without open intracranial wound AND with loss of consciousness
37	Injury-Trauma,Condition	18605003	Intracranial hemorrhage following injury with open intracranial wound AND brief loss of consciousness
37	Injury-Trauma,Condition	18685001	Late effect of internal injury to intra-abdominal organs
37	Injury-Trauma,Condition	18796000	Injury of renal artery
37	Injury-Trauma,Condition	19090007	Injury at C1-C4 level with complete lesion of spinal cord AND without bone injury
37	Injury-Trauma,Condition	19180002	Crushing injury of upper arm

© 2021 Matheny ME et al. *JAMA Network Open.*

CONDITIONID	CONDITION_DESCRIPTION	SNOMED_CODE	DESCRIPTION
37	Injury-Trauma,Condition	19241004	Open dislocation of interphalangeal joint of hand
37	Injury-Trauma,Condition	19474002	Injury of common carotid artery
37	Injury-Trauma,Condition	19494006	Current tear of semilunar cartilage
37	Injury-Trauma,Condition	20026002	Open dislocation of proximal end of metacarpal bone of wrist
37	Injury-Trauma,Condition	20132009	Closed traumatic dislocation of fourth cervical vertebra
37	Injury-Trauma,Condition	20167009	Cortex laceration without open intracranial wound AND with prolonged loss of consciousness (more than 24 hours) AND return to pre-existing conscious level
37	Injury-Trauma,Condition	20213006	Injury of spleen without open wound into abdominal cavity
37	Injury-Trauma,Condition	20276007	Subarachnoid hemorrhage following injury without open intracranial wound AND with prolonged loss of consciousness (more than 24 hours) AND without return to

© 2021 Matheny ME et al. *JAMA Network Open.*

CONDITIONID	CONDITION_DESCRIPTION	SNOMED_CODE	DESCRIPTION
			pre-existing conscious level
37	Injury-Trauma,Condition	20341008	Injury of kidney without open wound into abdominal cavity
37	Injury-Trauma,Condition	20474007	Injury of multiple sites of pancreas without open wound into abdominal cavity
37	Injury-Trauma,Condition	20580001	Injury of bronchus with open wound into thoracic cavity
37	Injury-Trauma,Condition	20593006	Injury of superficial femoral artery
37	Injury-Trauma,Condition	20784007	Internal injury of abdominal organs with open wound into cavity
37	Injury-Trauma,Condition	20899000	Brain stem laceration without open intracranial wound
37	Injury-Trauma,Condition	21333004	Derangement of lateral meniscus
37	Injury-Trauma,Condition	21949009	Closed traumatic dislocation of interphalangeal joint of toe
37	Injury-Trauma,Condition	21978005	Open fracture of C1-C4 level with anterior cord syndrome
37	Injury-Trauma,Condition	22095002	Injury of adrenal gland without open wound into abdominal cavity

© 2021 Matheny ME et al. *JAMA Network Open.*

CONDITIONID	CONDITION_DESCRIPTION	SNOMED_CODE	DESCRIPTION
37	Injury-Trauma,Condition	22383006	Closed fracture of vault of skull with cerebral laceration AND/OR contusion
37	Injury-Trauma,Condition	22682006	Open lateral dislocation of proximal end of tibia
37	Injury-Trauma,Condition	22693008	Brain injury without open intracranial wound AND with brief loss of consciousness
37	Injury-Trauma,Condition	22897006	Traumatic pneumothorax with open wound into thorax
37	Injury-Trauma,Condition	22911007	Closed traumatic dislocation of joint of shoulder region
37	Injury-Trauma,Condition	22973003	Closed fracture of vertebral column with spinal cord injury
37	Injury-Trauma,Condition	23026001	Brain stem contusion with open intracranial wound AND moderate loss of consciousness
37	Injury-Trauma,Condition	23368007	Injury of uterus without open wound into abdominal cavity
37	Injury-Trauma,Condition	23589004	Injury of spleen
37	Injury-Trauma,Condition	23658007	Late effect of injury to peripheral nerve of shoulder girdle AND/OR upper limb

© 2021 Matheny ME et al. *JAMA Network Open.*

CONDITIONID	CONDITION_DESCRIPTION	SNOMED_CODE	DESCRIPTION
37	Injury-Trauma,Condition	24056002	Multiple closed fractures of skull AND/OR face with subarachnoid, subdural AND/OR extradural hemorrhage
37	Injury-Trauma,Condition	24179004	Hematoma AND contusion of liver without open wound into abdominal cavity
37	Injury-Trauma,Condition	24350003	Injury of lung without open wound into thorax
37	Injury-Trauma,Condition	24392008	Injury at C1-C4 level with spinal cord injury AND without bone injury
37	Injury-Trauma,Condition	25110002	Injury of multiple sites in colon AND/OR rectum with open wound into abdominal cavity
37	Injury-Trauma,Condition	25185004	Cerebellar contusion with open intracranial wound AND no loss of consciousness
37	Injury-Trauma,Condition	25331007	Injury at T7-T12 level with spinal cord injury AND without bone injury
37	Injury-Trauma,Condition	25420003	Injury of multiple sites of pancreas with open wound into abdominal cavity

© 2021 Matheny ME et al. *JAMA Network Open.*

CONDITIONID	CONDITION_DESCRIPTION	SNOMED_CODE	DESCRIPTION
37	Injury-Trauma,Condition	25554004	Major laceration of liver with open wound into abdominal cavity
37	Injury-Trauma,Condition	25689009	Cortex laceration without open intracranial wound AND with brief loss of consciousness
37	Injury-Trauma,Condition	26205001	Subdural hemorrhage following injury without open intracranial wound AND with brief loss of consciousness
37	Injury-Trauma,Condition	26738009	Spinal cord injury without spinal bone injury
37	Injury-Trauma,Condition	27817002	Internal injury of chest
37	Injury-Trauma,Condition	27923006	Brain injury with open intracranial wound AND prolonged loss of consciousness (more than 24 hours) AND return to pre-existing conscious level
37	Injury-Trauma,Condition	27937002	Open fracture of T7-T12 level with anterior cord syndrome
37	Injury-Trauma,Condition	28048009	Subarachnoid hemorrhage following injury without open intracranial wound
37	Injury-Trauma,Condition	28155008	Extradural hemorrhage

© 2021 Matheny ME et al. *JAMA Network Open.*

CONDITIONID	CONDITION_DESCRIPTION	SNOMED_CODE	DESCRIPTION
			following injury with open intracranial wound AND concussion
37	Injury-Trauma,Condition	28156009	Brain injury with open intracranial wound AND brief loss of consciousness
37	Injury-Trauma,Condition	28188001	Brain injury with open intracranial wound
37	Injury-Trauma,Condition	28545001	Injury of renal vessels
37	Injury-Trauma,Condition	29264003	Closed fracture of base of skull with subarachnoid, subdural AND/OR extradural hemorrhage
37	Injury-Trauma,Condition	29635000	Subdural hemorrhage following injury with open intracranial wound AND brief loss of consciousness
37	Injury-Trauma,Condition	29691006	Injury of multiple sites in colon AND/OR rectum without open wound into abdominal cavity
37	Injury-Trauma,Condition	29807001	Brain stem contusion without open intracranial wound AND with loss of consciousness
37	Injury-Trauma,Condition	29818001	Closed traumatic dislocation of

© 2021 Matheny ME et al. *JAMA Network Open.*

CONDITIONID	CONDITION_DESCRIPTION	SNOMED_CODE	DESCRIPTION
			metacarpophalangeal joint of finger
37	Injury-Trauma,Condition	29880001	Injury of rectum with open wound into abdominal cavity
37	Injury-Trauma,Condition	30371007	Open fracture of base of skull with cerebral laceration AND contusion
37	Injury-Trauma,Condition	30556007	Recurrent dislocation of shoulder region
37	Injury-Trauma,Condition	31110000	Injury of ovarian vein
37	Injury-Trauma,Condition	31235003	Closed fracture of C5-C7 level with anterior cord syndrome
37	Injury-Trauma,Condition	31882001	Closed fracture of T1-T6 level with spinal cord injury
37	Injury-Trauma,Condition	32106001	Cortex contusion without open intracranial wound
37	Injury-Trauma,Condition	32734003	Crushing injury of multiple sites of trunk
37	Injury-Trauma,Condition	32834005	Brief loss of consciousness
37	Injury-Trauma,Condition	33072005	Traumatic hemothorax with open wound into thorax
37	Injury-Trauma,Condition	33117006	Cerebellar contusion without open intracranial wound AND with prolonged loss of consciousness (more than 24

© 2021 Matheny ME et al. *JAMA Network Open.*

CONDITIONID	CONDITION_DESCRIPTION	SNOMED_CODE	DESCRIPTION
			hours) AND return to pre-existing conscious level
37	Injury-Trauma,Condition	33332005	Brain injury without open intracranial wound AND with concussion
37	Injury-Trauma,Condition	33358005	Closed traumatic dislocation of sternum
37	Injury-Trauma,Condition	33908005	Injury of peritoneum without open wound into abdominal cavity
37	Injury-Trauma,Condition	34047002	Injury of multiple intrathoracic organs without open wound into cavity
37	Injury-Trauma,Condition	34135005	Subdural hemorrhage following injury without open intracranial wound AND with loss of consciousness
37	Injury-Trauma,Condition	34501004	Intracranial hemorrhage following injury with intracranial wound AND moderate loss of consciousness
37	Injury-Trauma,Condition	34565006	Closed anterior dislocation of humerus
37	Injury-Trauma,Condition	34704009	Injury of portal vein

© 2021 Matheny ME et al. *JAMA Network Open.*

CONDITIONID	CONDITION_DESCRIPTION	SNOMED_CODE	DESCRIPTION
37	Injury-Trauma,Condition	34751006	Cerebellar laceration without open intracranial wound AND with brief loss of consciousness
37	Injury-Trauma,Condition	34798003	Injury of liver without open wound into abdominal cavity
37	Injury-Trauma,Condition	34873006	Open dislocation of wrist
37	Injury-Trauma,Condition	35106007	Closed traumatic dislocation of knee joint
37	Injury-Trauma,Condition	35191005	Injury of blood vessels of abdomen AND/OR pelvis
37	Injury-Trauma,Condition	35672006	Subarachnoid hemorrhage following injury without open intracranial wound AND with no loss of consciousness
37	Injury-Trauma,Condition	35790004	Injury of retroperitoneum without open wound into abdominal cavity
37	Injury-Trauma,Condition	36071006	Chondromalacia of patella
37	Injury-Trauma,Condition	36523002	Injury of posterior tibial artery
37	Injury-Trauma,Condition	36539009	Disorder due to and following injury of cranial nerve

© 2021 Matheny ME et al. *JAMA Network Open.*

CONDITIONID	CONDITION_DESCRIPTION	SNOMED_CODE	DESCRIPTION
37	Injury-Trauma,Condition	36822000	Cortex contusion without open intracranial wound AND with prolonged loss of consciousness (more than 24 hours) without return to pre-existing conscious level
37	Injury-Trauma,Condition	37134004	Intracranial hemorrhage following injury without open intracranial wound AND with no loss of consciousness
37	Injury-Trauma,Condition	37325002	Injury of saphenous vein
37	Injury-Trauma,Condition	37553001	Injury of axillary vein
37	Injury-Trauma,Condition	38128008	Crushing injury of knee
37	Injury-Trauma,Condition	38261007	Injury of superior vena cava
37	Injury-Trauma,Condition	38278003	Injury at C5-C7 level with central cord syndrome AND without bone injury
37	Injury-Trauma,Condition	38556006	Closed traumatic dislocation of joint of wrist
37	Injury-Trauma,Condition	38972005	Injury of sigmoid colon without open wound into abdominal cavity
37	Injury-Trauma,Condition	39131004	Injury of heart without open wound into thorax

© 2021 Matheny ME et al. *JAMA Network Open.*

CONDITIONID	CONDITION_DESCRIPTION	SNOMED_CODE	DESCRIPTION
37	Injury-Trauma,Condition	39400004	Injury of liver
37	Injury-Trauma,Condition	39595001	Crushing injury of lower limb
37	Injury-Trauma,Condition	39881005	Injury at C5-C7 level with complete lesion of spinal cord AND without bone injury
37	Injury-Trauma,Condition	40047007	Closed traumatic dislocation of sixth cervical vertebra
37	Injury-Trauma,Condition	40095003	Injury of kidney
37	Injury-Trauma,Condition	40135004	Subdural hemorrhage following injury without open intracranial wound AND with no loss of consciousness
37	Injury-Trauma,Condition	40286002	Cortex contusion without open intracranial wound AND with prolonged loss of consciousness (more than 24 hours) AND return to pre-existing conscious level
37	Injury-Trauma,Condition	40368002	Crushing injury of scapular region
37	Injury-Trauma,Condition	40521006	Contusion to heart with open wound into thorax
37	Injury-Trauma,Condition	40549004	Extradural hemorrhage following injury without open intracranial wound

© 2021 Matheny ME et al. *JAMA Network Open.*

CONDITIONID	CONDITION_DESCRIPTION	SNOMED_CODE	DESCRIPTION
			AND with brief loss of consciousness
37	Injury-Trauma,Condition	40795009	Injury at T1-T6 level with complete lesion of spinal cord AND without bone injury
37	Injury-Trauma,Condition	40863000	Moderate loss of consciousness
37	Injury-Trauma,Condition	40874009	Crushing injury of lower leg
37	Injury-Trauma,Condition	41061006	Closed traumatic dislocation of tarsometatarsal joint
37	Injury-Trauma,Condition	41222005	Brain stem laceration with open intracranial wound AND concussion
37	Injury-Trauma,Condition	41335004	Injury of blood vessels of lower extremity
37	Injury-Trauma,Condition	41359009	Closed anterior dislocation of proximal end of tibia
37	Injury-Trauma,Condition	41455002	Closed fracture of sacrum AND/OR coccyx with complete cauda equina lesion
37	Injury-Trauma,Condition	41547006	Injury of bronchus without open wound into thoracic cavity
37	Injury-Trauma,Condition	41870008	Cortex laceration with open intracranial wound

© 2021 Matheny ME et al. *JAMA Network Open.*

CONDITIONID	CONDITION_DESCRIPTION	SNOMED_CODE	DESCRIPTION
			AND loss of consciousness
37	Injury-Trauma,Condition	42019003	Contusion of lung with open wound into thorax
37	Injury-Trauma,Condition	42206007	Injury at T1-T6 level with anterior cord syndrome AND without bone injury
37	Injury-Trauma,Condition	42670008	Brain stem contusion with open intracranial wound AND prolonged loss of consciousness (more than 24 hours) without return to pre-existing conscious level
37	Injury-Trauma,Condition	43216008	Extradural hemorrhage following injury without open intracranial wound
37	Injury-Trauma,Condition	43262000	Subdural hemorrhage following injury with open intracranial wound AND concussion
37	Injury-Trauma,Condition	43405004	Open fracture of C5-C7 level with anterior cord syndrome
37	Injury-Trauma,Condition	43422002	Crushing injury of foot
37	Injury-Trauma,Condition	43841005	Moderate laceration of liver without open

© 2021 Matheny ME et al. *JAMA Network Open.*

CONDITIONID	CONDITION_DESCRIPTION	SNOMED_CODE	DESCRIPTION
			wound into abdominal cavity
37	Injury-Trauma,Condition	43842003	Injury of lung with open wound into thorax
37	Injury-Trauma,Condition	44237008	Open dislocation of coccyx
37	Injury-Trauma,Condition	44333005	Open medial dislocation of proximal end of tibia
37	Injury-Trauma,Condition	44341005	Cortex laceration without open intracranial wound
37	Injury-Trauma,Condition	44434003	Closed fracture of thoracic region with spinal cord injury
37	Injury-Trauma,Condition	44599007	Traumatic hemothorax without open wound into thorax
37	Injury-Trauma,Condition	45387006	Open dislocation of second cervical vertebra
37	Injury-Trauma,Condition	45634004	Closed traumatic dislocation of carpometacarpal joint of wrist
37	Injury-Trauma,Condition	45659008	Subdural hemorrhage following injury with open intracranial wound AND loss of consciousness
37	Injury-Trauma,Condition	46307004	Closed posterior dislocation of humerus
37	Injury-Trauma,Condition	46328008	Injury of ureter without open

© 2021 Matheny ME et al. *JAMA Network Open.*

CONDITIONID	CONDITION_DESCRIPTION	SNOMED_CODE	DESCRIPTION
			wound into abdominal cavity
37	Injury-Trauma,Condition	46549005	Multiple spinal cord injuries without spinal bone injury
37	Injury-Trauma,Condition	46758000	Open fracture of base of skull with subarachnoid, subdural AND/OR extradural hemorrhage
37	Injury-Trauma,Condition	46931002	Closed traumatic dislocation of second cervical vertebra
37	Injury-Trauma,Condition	46995009	Closed fracture of C5-C7 level with spinal cord injury
37	Injury-Trauma,Condition	47450003	Brain injury without open intracranial wound AND with moderate loss of consciousness
37	Injury-Trauma,Condition	47462004	Brain injury without open intracranial wound AND with prolonged loss of consciousness (more than 24 hours) without return to pre-existing conscious level
37	Injury-Trauma,Condition	47468000	Injury of gastric artery
37	Injury-Trauma,Condition	47533003	Injury of tail of pancreas without

© 2021 Matheny ME et al. *JAMA Network Open.*

CONDITIONID	CONDITION_DESCRIPTION	SNOMED_CODE	DESCRIPTION
			open wound into abdominal cavity
37	Injury-Trauma,Condition	47771009	Injury of gastrointestinal tract
37	Injury-Trauma,Condition	47814001	Crushing injury of wrist
37	Injury-Trauma,Condition	47979002	Cortex contusion without open intracranial wound AND with moderate loss of consciousness
37	Injury-Trauma,Condition	47983002	Cerebellar laceration without open intracranial wound AND with moderate loss of consciousness
37	Injury-Trauma,Condition	48424004	Contusion of lung without open wound into thorax
37	Injury-Trauma,Condition	48518008	Subarachnoid hemorrhage following injury with open intracranial wound AND concussion
37	Injury-Trauma,Condition	48653005	Injury of appendix without open wound into abdominal cavity
37	Injury-Trauma,Condition	48956000	Open fracture of lumbar vertebra with spinal cord injury
37	Injury-Trauma,Condition	49011004	Internal injury of abdominal organs
37	Injury-Trauma,Condition	49055005	Closed traumatic dislocation of metatarsal joint

© 2021 Matheny ME et al. *JAMA Network Open.*

CONDITIONID	CONDITION_DESCRIPTION	SNOMED_CODE	DESCRIPTION
37	Injury-Trauma,Condition	49280002	Injury of multiple intra-abdominal organs with open wound into abdominal cavity
37	Injury-Trauma,Condition	49497005	Open fracture of T1-T6 level with anterior cord syndrome
37	Injury-Trauma,Condition	49747005	Closed fracture of lumbar vertebra with spinal cord injury
37	Injury-Trauma,Condition	49880009	Late effect of crushing injury
37	Injury-Trauma,Condition	49891002	Closed traumatic dislocation of coccyx
37	Injury-Trauma,Condition	50001009	Crushing injury of neck
37	Injury-Trauma,Condition	50087006	Open dislocation of third cervical vertebra
37	Injury-Trauma,Condition	50088001	Hematoma of spleen without rupture of capsule AND without open wound into abdominal cavity
37	Injury-Trauma,Condition	50444002	Injury of diaphragm without open wound into cavity
37	Injury-Trauma,Condition	50793006	Crushing injury of hand
37	Injury-Trauma,Condition	50888000	Closed fracture of C1-C4 level with central cord syndrome

© 2021 Matheny ME et al. *JAMA Network Open.*

CONDITIONID	CONDITION_DESCRIPTION	SNOMED_CODE	DESCRIPTION
37	Injury-Trauma,Condition	50952009	Recurrent dislocation of ankle AND/OR foot
37	Injury-Trauma,Condition	51000003	Injury of multiple intra-abdominal organs without open wound into abdominal cavity
37	Injury-Trauma,Condition	51101002	Cerebellar contusion without open intracranial wound AND with brief loss of consciousness
37	Injury-Trauma,Condition	51536009	Injury of hypogastric artery
37	Injury-Trauma,Condition	51879004	Closed medial dislocation of proximal end of tibia
37	Injury-Trauma,Condition	52087004	Injury of rectum without open wound into abdominal cavity
37	Injury-Trauma,Condition	52289006	Closed fracture of T1-T6 level with central cord syndrome
37	Injury-Trauma,Condition	52576001	Injury of anterior tibial artery
37	Injury-Trauma,Condition	52888005	Brain stem contusion without open intracranial wound
37	Injury-Trauma,Condition	52902005	Subdural hemorrhage following injury with open intracranial wound AND prolonged loss of

© 2021 Matheny ME et al. *JAMA Network Open.*

CONDITIONID	CONDITION_DESCRIPTION	SNOMED_CODE	DESCRIPTION
			consciousness (more than 24 hours) AND return to pre-existing conscious level
37	Injury-Trauma,Condition	52984008	Injury of common femoral artery
37	Injury-Trauma,Condition	53417006	Articular cartilage disorder
37	Injury-Trauma,Condition	53730006	Cortex laceration without open intracranial wound AND with no loss of consciousness
37	Injury-Trauma,Condition	53810004	Injury at T1-T6 level with spinal cord injury AND without bone injury
37	Injury-Trauma,Condition	53868003	Closed fracture of C1-C4 level with anterior cord syndrome
37	Injury-Trauma,Condition	54317001	Open dislocation of lumbar vertebra
37	Injury-Trauma,Condition	54394007	Open dislocation of ankle
37	Injury-Trauma,Condition	54420005	Open dislocation of finger
37	Injury-Trauma,Condition	55126004	Closed traumatic dislocation of tarsal joint
37	Injury-Trauma,Condition	55600008	Injury at T7-T12 level with anterior cord syndrome AND without bone injury
37	Injury-Trauma,Condition	55885004	Brain injury without open intracranial wound AND with

© 2021 Matheny ME et al. *JAMA Network Open.*

CONDITIONID	CONDITION_DESCRIPTION	SNOMED_CODE	DESCRIPTION
			prolonged loss of consciousness (more than 24 hours) with return to pre-existing conscious level
37	Injury-Trauma,Condition	55983005	Injury at T7-T12 level with complete lesion of spinal cord AND without bone injury
37	Injury-Trauma,Condition	56515006	Injury of inferior vena cava
37	Injury-Trauma,Condition	57012007	Brain stem contusion with open intracranial wound AND brief loss of consciousness
37	Injury-Trauma,Condition	57467003	Closed traumatic dislocation of distal radioulnar joint of wrist
37	Injury-Trauma,Condition	57662003	Injury of blood vessel
37	Injury-Trauma,Condition	57998008	Closed fracture of vault of skull with subarachnoid, subdural AND/OR extradural hemorrhage
37	Injury-Trauma,Condition	58020007	Closed skull fracture with subarachnoid, subdural AND/OR extradural hemorrhage
37	Injury-Trauma,Condition	58070005	Cortex laceration without open intracranial wound

© 2021 Matheny ME et al. *JAMA Network Open.*

CONDITIONID	CONDITION_DESCRIPTION	SNOMED_CODE	DESCRIPTION
			AND with moderate loss of consciousness
37	Injury-Trauma,Condition	58188004	Traumatic arthropathy
37	Injury-Trauma,Condition	58320001	Traumatic dislocation of knee joint
37	Injury-Trauma,Condition	58361001	Injury of posterior tibial vein
37	Injury-Trauma,Condition	58565006	Injury of head of pancreas without open wound into cavity
37	Injury-Trauma,Condition	58935006	Injury of stomach without open wound into abdominal cavity
37	Injury-Trauma,Condition	59029004	Cortex contusion without open intracranial wound AND with brief loss of consciousness
37	Injury-Trauma,Condition	59038002	Crushing injury of back
37	Injury-Trauma,Condition	59190009	Traumatic pneumohemothorax without open wound into thorax
37	Injury-Trauma,Condition	59568004	Injury of thoracic aorta
37	Injury-Trauma,Condition	59633005	Subarachnoid hemorrhage following injury with open intracranial wound AND brief loss of consciousness
37	Injury-Trauma,Condition	59648004	Extradural hemorrhage following injury

© 2021 Matheny ME et al. *JAMA Network Open.*

CONDITIONID	CONDITION_DESCRIPTION	SNOMED_CODE	DESCRIPTION
			with open intracranial wound AND moderate loss of consciousness
37	Injury-Trauma,Condition	59748008	Cortex laceration with open intracranial wound
37	Injury-Trauma,Condition	60190000	Injury of blood vessels of thorax
37	Injury-Trauma,Condition	60366008	Injury of pulmonary vein
37	Injury-Trauma,Condition	61014001	Laceration extending into parenchyma of spleen without open wound into abdominal cavity
37	Injury-Trauma,Condition	61386002	Open fracture of C1-C4 level with central cord syndrome
37	Injury-Trauma,Condition	61474001	Injury of kidney with open wound into abdominal cavity
37	Injury-Trauma,Condition	61642003	Multiple open fractures of skull AND/OR face with subarachnoid, subdural AND/OR extradural hemorrhage
37	Injury-Trauma,Condition	61812003	Injury of multiple blood vessels of abdomen AND/OR pelvis
37	Injury-Trauma,Condition	62106007	Concussion with no loss of consciousness

© 2021 Matheny ME et al. *JAMA Network Open.*

CONDITIONID	CONDITION_DESCRIPTION	SNOMED_CODE	DESCRIPTION
37	Injury-Trauma,Condition	62564004	Concussion with loss of consciousness
37	Injury-Trauma,Condition	62973004	Extradural hemorrhage following injury without open intracranial wound AND with moderate loss of consciousness
37	Injury-Trauma,Condition	63023005	Brain stem contusion without open intracranial wound AND with prolonged loss of consciousness (more than 24 hours) without return to pre-existing conscious level
37	Injury-Trauma,Condition	63141004	Closed traumatic dislocation of joint of foot
37	Injury-Trauma,Condition	63323000	Subdural hemorrhage following injury without open intracranial wound AND with moderate loss of consciousness
37	Injury-Trauma,Condition	63456003	Open anterior dislocation of proximal end of tibia
37	Injury-Trauma,Condition	63643000	Derangement of knee
37	Injury-Trauma,Condition	63819001	Open fracture of T1-T6 level with

© 2021 Matheny ME et al. *JAMA Network Open.*

CONDITIONID	CONDITION_DESCRIPTION	SNOMED_CODE	DESCRIPTION
			central cord syndrome
37	Injury-Trauma,Condition	63975004	Closed posterior dislocation of hip
37	Injury-Trauma,Condition	64042000	Injury of uterine vein
37	Injury-Trauma,Condition	64323009	Complete disruption of kidney parenchyma without open wound into cavity
37	Injury-Trauma,Condition	64413001	Brain stem laceration with open intracranial wound AND prolonged loss of consciousness (more than 24 hours) without return to pre-existing conscious level
37	Injury-Trauma,Condition	64438006	Closed traumatic dislocation of metatarsophalangeal joint
37	Injury-Trauma,Condition	64617008	Injury at T7-T12 level with central cord syndrome AND without bone injury
37	Injury-Trauma,Condition	64638002	Injury of hepatic vein
37	Injury-Trauma,Condition	64672005	Injury of innominate artery
37	Injury-Trauma,Condition	64684007	Injury at T1-T6 level with central cord syndrome AND without bone injury

© 2021 Matheny ME et al. *JAMA Network Open.*

CONDITIONID	CONDITION_DESCRIPTION	SNOMED_CODE	DESCRIPTION
37	Injury-Trauma,Condition	64789007	Internal injury of abdominal organs without open wound into cavity
37	Injury-Trauma,Condition	65071008	Injury of gastrointestinal tract without open wound into abdominal cavity
37	Injury-Trauma,Condition	65189006	Extradural hemorrhage following injury with open intracranial wound
37	Injury-Trauma,Condition	65324009	Minor laceration of liver without open wound into abdominal cavity
37	Injury-Trauma,Condition	65491009	Open fracture of sacrum AND/OR coccyx with spinal cord injury
37	Injury-Trauma,Condition	65535000	Cerebellar laceration without open intracranial wound AND with prolonged loss of consciousness (more than 24 hours) without return to pre-existing conscious level
37	Injury-Trauma,Condition	65785006	Injury of superior mesenteric vein AND/OR primary subdivisions
37	Injury-Trauma,Condition	65896005	Crushing injury of ankle
37	Injury-Trauma,Condition	66393002	Brain stem contusion with

© 2021 Matheny ME et al. *JAMA Network Open.*

CONDITIONID	CONDITION_DESCRIPTION	SNOMED_CODE	DESCRIPTION
			open intracranial wound AND loss of consciousness
37	Injury-Trauma,Condition	66829004	Injury of splenic artery
37	Injury-Trauma,Condition	66976009	Intracranial hemorrhage following injury without intracranial wound AND with moderate loss of consciousness
37	Injury-Trauma,Condition	67447005	Injury of body of pancreas without open wound into abdominal cavity
37	Injury-Trauma,Condition	67843002	Injury of axillary artery
37	Injury-Trauma,Condition	67861006	Injury at C1-C4 level with anterior cord syndrome AND without bone injury
37	Injury-Trauma,Condition	68708005	Injury of celiac AND/OR mesenteric arteries
37	Injury-Trauma,Condition	68734002	Injury of small intestine without open wound into abdominal cavity
37	Injury-Trauma,Condition	69178005	Subarachnoid hemorrhage following injury with open intracranial wound AND prolonged loss of consciousness (more than 24 hours) without

© 2021 Matheny ME et al. *JAMA Network Open.*

CONDITIONID	CONDITION_DESCRIPTION	SNOMED_CODE	DESCRIPTION
			return to pre-existing conscious level
37	Injury-Trauma,Condition	69458009	Subarachnoid hemorrhage following injury without open intracranial wound AND with loss of consciousness
37	Injury-Trauma,Condition	69593000	Open dislocation of fourth cervical vertebra
37	Injury-Trauma,Condition	69875006	Cortex laceration with open intracranial wound AND concussion
37	Injury-Trauma,Condition	69913007	Late effect of internal injury to chest
37	Injury-Trauma,Condition	69976008	Open dislocation of metatarsal joint
37	Injury-Trauma,Condition	70000009	Crushing injury of multiple sites of lower limb
37	Injury-Trauma,Condition	70307002	Injury at C5-C7 level with anterior cord syndrome AND without bone injury
37	Injury-Trauma,Condition	70861009	Subdural hemorrhage following injury without open intracranial wound AND with prolonged loss of consciousness (more than 24 hours) without

© 2021 Matheny ME et al. *JAMA Network Open.*

CONDITIONID	CONDITION_DESCRIPTION	SNOMED_CODE	DESCRIPTION
			return to pre-existing conscious level
37	Injury-Trauma,Condition	71074007	Open skull fracture with subarachnoid, subdural AND/OR extradural hemorrhage
37	Injury-Trauma,Condition	71266000	Open fracture of vault of skull with subarachnoid, subdural AND/OR extradural hemorrhage
37	Injury-Trauma,Condition	71458007	Crushing injury of external genitalia
37	Injury-Trauma,Condition	72513001	Closed fracture of T7-T12 level with spinal cord injury
37	Injury-Trauma,Condition	73143002	Multiple closed fractures of skull AND/OR face with cerebral laceration AND/OR contusion
37	Injury-Trauma,Condition	73308006	Extradural hemorrhage following injury with open intracranial wound AND brief loss of consciousness
37	Injury-Trauma,Condition	73387003	Closed traumatic dislocation of proximal end of metacarpal bone of wrist
37	Injury-Trauma,Condition	73413009	Cortex contusion with open intracranial wound AND concussion

© 2021 Matheny ME et al. *JAMA Network Open.*

CONDITIONID	CONDITION_DESCRIPTION	SNOMED_CODE	DESCRIPTION
37	Injury-Trauma,Condition	73439007	Subarachnoid hemorrhage following injury with open intracranial wound AND prolonged loss of consciousness (more than 24 hours) AND return to pre-existing conscious level
37	Injury-Trauma,Condition	73477001	Traumatic pneumohemothorax with open wound into thorax
37	Injury-Trauma,Condition	73557005	Injury of internal mammary vein
37	Injury-Trauma,Condition	73825003	Crushing injury of buttock
37	Injury-Trauma,Condition	74270009	Crushing injury of thigh
37	Injury-Trauma,Condition	74324004	Laceration of kidney without open wound into abdominal cavity
37	Injury-Trauma,Condition	74360009	Open fracture of C5-C7 level with central cord syndrome
37	Injury-Trauma,Condition	74472004	Late effect of intracranial injury without skull fracture
37	Injury-Trauma,Condition	75137002	Closed traumatic dislocation of joint of finger
37	Injury-Trauma,Condition	75507000	Subarachnoid hemorrhage following injury with open

© 2021 Matheny ME et al. *JAMA Network Open.*

CONDITIONID	CONDITION_DESCRIPTION	SNOMED_CODE	DESCRIPTION
			intracranial wound AND loss of consciousness
37	Injury-Trauma,Condition	75824002	Closed lateral dislocation of elbow
37	Injury-Trauma,Condition	76019006	Injury of internal jugular vein
37	Injury-Trauma,Condition	76211000	Injury of iliac vein
37	Injury-Trauma,Condition	76276001	Injury of popliteal vessels
37	Injury-Trauma,Condition	76435008	Open fracture of C1-C4 level with spinal cord injury
37	Injury-Trauma,Condition	76675006	Cerebellar laceration without open intracranial wound AND with concussion
37	Injury-Trauma,Condition	76877000	Injury of diaphragm with open wound into cavity
37	Injury-Trauma,Condition	77165001	Injury of bladder
37	Injury-Trauma,Condition	77498000	Extradural hemorrhage following injury without open intracranial wound AND with concussion
37	Injury-Trauma,Condition	77768006	Intracranial hemorrhage following injury with open intracranial wound
37	Injury-Trauma,Condition	77860008	Derangement of posterior horn of lateral meniscus
37	Injury-Trauma,Condition	77940004	Hematoma of spleen without

© 2021 Matheny ME et al. *JAMA Network Open.*

CONDITIONID	CONDITION_DESCRIPTION	SNOMED_CODE	DESCRIPTION
			rupture of capsule AND with open wound into abdominal cavity
37	Injury-Trauma,Condition	78157002	Closed fracture of C5-C7 level with central cord syndrome
37	Injury-Trauma,Condition	78211006	Open fracture of thoracic spine with spinal cord injury
37	Injury-Trauma,Condition	78396000	Open dislocation of seventh cervical vertebra
37	Injury-Trauma,Condition	78477003	Intracranial hemorrhage following injury without open intracranial wound AND with prolonged loss of consciousness (more than 24 hours) AND return to pre-existing conscious level
37	Injury-Trauma,Condition	78757009	Intracranial hemorrhage following injury without open intracranial wound AND with concussion
37	Injury-Trauma,Condition	78879009	Intracranial hemorrhage following injury with open intracranial wound AND concussion
37	Injury-Trauma,Condition	78968003	Brain stem contusion with

© 2021 Matheny ME et al. *JAMA Network Open.*

CONDITIONID	CONDITION_DESCRIPTION	SNOMED_CODE	DESCRIPTION
			open intracranial wound
37	Injury-Trauma,Condition	79125007	Open dislocation of first cervical vertebra
37	Injury-Trauma,Condition	79220008	Brain stem contusion without open intracranial wound AND with concussion
37	Injury-Trauma,Condition	80090009	Open dislocation of metacarpophalangeal joint of finger
37	Injury-Trauma,Condition	80192004	Massive parenchymal disruption of spleen with open wound into abdominal cavity
37	Injury-Trauma,Condition	80820004	Injury of internal carotid artery
37	Injury-Trauma,Condition	80980003	Open fracture of C5-C7 level with spinal cord injury
37	Injury-Trauma,Condition	81193001	Pelvic organ injury with open wound into abdominal cavity
37	Injury-Trauma,Condition	81268009	Cerebellar contusion without open intracranial wound AND with moderate loss of consciousness
37	Injury-Trauma,Condition	81412002	Cortex contusion with open intracranial wound AND loss of consciousness
37	Injury-Trauma,Condition	81428007	Crushing injury of axillary region

© 2021 Matheny ME et al. *JAMA Network Open.*

CONDITIONID	CONDITION_DESCRIPTION	SNOMED_CODE	DESCRIPTION
37	Injury-Trauma,Condition	81442004	Closed fracture of cervical region with spinal cord injury
37	Injury-Trauma,Condition	81512004	Loose body in knee
37	Injury-Trauma,Condition	81520002	Extradural hemorrhage following injury with open intracranial wound AND prolonged loss of consciousness (more than 24 hours) AND return to pre-existing conscious level
37	Injury-Trauma,Condition	81629009	Traumatic dislocation of temporomandibular joint
37	Injury-Trauma,Condition	81642009	Late effect of spinal cord injury
37	Injury-Trauma,Condition	81813004	Closed medial dislocation of elbow
37	Injury-Trauma,Condition	82632005	Derangement of anterior horn of lateral meniscus
37	Injury-Trauma,Condition	82637004	Cerebellar contusion without open intracranial wound AND with no loss of consciousness
37	Injury-Trauma,Condition	83209008	Injury of transverse colon without open wound into abdominal cavity
37	Injury-Trauma,Condition	83276000	Injury of uterus with open wound

© 2021 Matheny ME et al. *JAMA Network Open.*

CONDITIONID	CONDITION_DESCRIPTION	SNOMED_CODE	DESCRIPTION
			into abdominal cavity
37	Injury-Trauma,Condition	83401009	Open dislocation of interphalangeal joint of foot
37	Injury-Trauma,Condition	84097002	Articular cartilage disorder of multiple sites
37	Injury-Trauma,Condition	84338002	Laceration of heart with penetration of heart chambers
37	Injury-Trauma,Condition	84621006	Injury of esophagus with open wound into thoracic cavity
37	Injury-Trauma,Condition	84792001	Extradural hemorrhage following injury without open intracranial wound AND with prolonged loss of consciousness (more than 24 hours) AND return to pre-existing conscious level
37	Injury-Trauma,Condition	84803006	Cerebellar laceration with open intracranial wound AND moderate loss of consciousness
37	Injury-Trauma,Condition	84920009	Open dislocation of hip
37	Injury-Trauma,Condition	84970007	Articular cartilage disorder of ankle AND/OR foot
37	Injury-Trauma,Condition	85314006	Cerebellar laceration without open intracranial wound AND with

© 2021 Matheny ME et al. *JAMA Network Open.*

CONDITIONID	CONDITION_DESCRIPTION	SNOMED_CODE	DESCRIPTION
			no loss of consciousness
37	Injury-Trauma,Condition	85341001	Closed inferior dislocation of humerus
37	Injury-Trauma,Condition	85453003	Open dislocation of metatarsophalangeal joint
37	Injury-Trauma,Condition	85564003	Injury of blood vessels of head AND/OR neck
37	Injury-Trauma,Condition	85719003	Injury of subclavian vein
37	Injury-Trauma,Condition	86006001	Massive parenchymal disruption of spleen without open wound into abdominal cavity
37	Injury-Trauma,Condition	86010003	Brain injury with open intracranial wound AND moderate loss of consciousness
37	Injury-Trauma,Condition	86125000	Cerebellar contusion with open intracranial wound AND concussion
37	Injury-Trauma,Condition	86182004	Extradural hemorrhage following injury with open intracranial wound AND prolonged loss of consciousness (more than 24 hours) without return to pre-

© 2021 Matheny ME et al. *JAMA Network Open.*

CONDITIONID	CONDITION_DESCRIPTION	SNOMED_CODE	DESCRIPTION
			existing conscious level
37	Injury-Trauma,Condition	86378006	Old bucket handle tear of medial meniscus
37	Injury-Trauma,Condition	86488006	Brain injury with open intracranial wound AND prolonged loss of consciousness (more than 24 hours) without return to pre-existing conscious level
37	Injury-Trauma,Condition	87020000	Subarachnoid hemorrhage following injury without open intracranial wound AND with brief loss of consciousness
37	Injury-Trauma,Condition	87253004	Subarachnoid hemorrhage following injury with open intracranial wound AND moderate loss of consciousness
37	Injury-Trauma,Condition	87345009	Subdural hemorrhage following injury without open intracranial wound AND with prolonged loss of consciousness (more than 24 hours) AND return

© 2021 Matheny ME et al. *JAMA Network Open.*

CONDITIONID	CONDITION_DESCRIPTION	SNOMED_CODE	DESCRIPTION
			to pre-existing conscious level
37	Injury-Trauma,Condition	87438005	Injury of multiple blood vessels of thorax
37	Injury-Trauma,Condition	87888006	Cortex contusion with open intracranial wound
37	Injury-Trauma,Condition	88008005	Multiple open fractures of skull AND/OR face with cerebral laceration AND/OR contusion
37	Injury-Trauma,Condition	88405003	Open fracture of T7-T12 level with central cord syndrome
37	Injury-Trauma,Condition	88533001	Late effect of injury to peripheral nerve of pelvic girdle AND/OR lower limb
37	Injury-Trauma,Condition	88651008	Closed skull fracture with cerebral laceration AND/OR contusion
37	Injury-Trauma,Condition	88743001	Open dislocation of vertebra
37	Injury-Trauma,Condition	88744007	Injury of blood vessels of upper extremity
37	Injury-Trauma,Condition	88747000	Subarachnoid hemorrhage following injury without open intracranial wound AND with prolonged loss of consciousness

© 2021 Matheny ME et al. *JAMA Network Open.*

CONDITIONID	CONDITION_DESCRIPTION	SNOMED_CODE	DESCRIPTION
			(more than 24 hours) AND return to pre-existing conscious level
37	Injury-Trauma,Condition	88837005	Injury of pulmonary artery
37	Injury-Trauma,Condition	88898002	Injury of primary branch of superior mesenteric artery
37	Injury-Trauma,Condition	89130002	Closed traumatic dislocation of fifth cervical vertebra
37	Injury-Trauma,Condition	89581003	Open dislocation of patella
37	Injury-Trauma,Condition	90068007	Articular cartilage disorder of hand
37	Injury-Trauma,Condition	90070003	Traumatic pneumothorax
37	Injury-Trauma,Condition	90165008	Subdural hemorrhage following injury with open intracranial wound AND moderate loss of consciousness
37	Injury-Trauma,Condition	90178008	Extradural hemorrhage following injury without open intracranial wound AND with loss of consciousness
37	Injury-Trauma,Condition	90289008	Closed obturator dislocation of hip
37	Injury-Trauma,Condition	90429009	Cerebellar laceration without open intracranial wound AND with loss of consciousness

© 2021 Matheny ME et al. *JAMA Network Open.*

CONDITIONID	CONDITION_DESCRIPTION	SNOMED_CODE	DESCRIPTION
37	Injury-Trauma,Condition	90898001	Open skull fracture with cerebral laceration AND/OR contusion
37	Injury-Trauma,Condition	91168001	Cerebellar laceration without open intracranial wound AND with prolonged loss of consciousness (more than 24 hours) AND return to pre-existing conscious level
37	Injury-Trauma,Condition	91216000	Intracranial hemorrhage following injury with open intracranial wound AND prolonged loss of consciousness (more than 24 hours) AND return to pre-existing conscious level
37	Injury-Trauma,Condition	91589002	Closed fracture of base of skull with cerebral laceration AND/OR contusion
37	Injury-Trauma,Condition	95854004	Pulled elbow
37	Injury-Trauma,Condition	105612003	Injury of internal organ
37	Injury-Trauma,Condition	105615001	Open wound of trunk
37	Injury-Trauma,Condition	108367008	Dislocation of joint
37	Injury-Trauma,Condition	110030002	Concussion injury of brain
37	Injury-Trauma,Condition	111222003	Derangement of medial meniscus

© 2021 Matheny ME et al. *JAMA Network Open.*

CONDITIONID	CONDITION_DESCRIPTION	SNOMED_CODE	DESCRIPTION
37	Injury-Trauma,Condition	111224002	Derangement of meniscus
37	Injury-Trauma,Condition	111593004	Closed fracture of vault of skull with intracranial hemorrhage
37	Injury-Trauma,Condition	111597003	Open fracture of vault of skull with intracranial hemorrhage
37	Injury-Trauma,Condition	111601003	Closed fracture of base of skull with intracranial hemorrhage
37	Injury-Trauma,Condition	111603000	Closed fracture of base of skull with intracranial injury
37	Injury-Trauma,Condition	111605007	Open fracture of base of skull with intracranial hemorrhage
37	Injury-Trauma,Condition	111607004	Open fracture of base of skull with intracranial injury
37	Injury-Trauma,Condition	111611005	Closed skull fracture with intracranial hemorrhage
37	Injury-Trauma,Condition	111613008	Closed skull fracture with intracranial injury
37	Injury-Trauma,Condition	111615001	Open skull fracture with intracranial hemorrhage
37	Injury-Trauma,Condition	111617009	Open skull fracture with intracranial injury
37	Injury-Trauma,Condition	111633007	Open fracture of sacrum AND/OR coccyx with

© 2021 Matheny ME et al. *JAMA Network Open.*

CONDITIONID	CONDITION_DESCRIPTION	SNOMED_CODE	DESCRIPTION
			complete cauda equina lesion
37	Injury-Trauma,Condition	111648001	Closed traumatic dislocation of cervical vertebra
37	Injury-Trauma,Condition	111649009	Closed anterior dislocation of hip
37	Injury-Trauma,Condition	111671004	Intracranial hemorrhage following injury with open intracranial wound and prolonged loss of consciousness (more than 24 hours) without return to pre-existing level
37	Injury-Trauma,Condition	111673001	Subarachnoid hemorrhage following injury without open intracranial wound AND with moderate loss of consciousness
37	Injury-Trauma,Condition	111677000	Subdural hemorrhage following injury with open intracranial wound and prolonged loss of consciousness (more than 24 hours) and without return to pre-existing conscious level
37	Injury-Trauma,Condition	111679002	Extradural hemorrhage following injury

© 2021 Matheny ME et al. *JAMA Network Open.*

CONDITIONID	CONDITION_DESCRIPTION	SNOMED_CODE	DESCRIPTION
			without open intracranial wound AND with no loss of consciousness
37	Injury-Trauma,Condition	111681000	Extradural hemorrhage following injury with open intracranial wound AND loss of consciousness
37	Injury-Trauma,Condition	111683002	Injury of duodenum without open wound into abdominal cavity
37	Injury-Trauma,Condition	111691006	Crushing injury of multiple sites
37	Injury-Trauma,Condition	111693009	Crushing injury of forearm
37	Injury-Trauma,Condition	111694003	Injury of branch of celiac axis
37	Injury-Trauma,Condition	111696001	Injury of femoral vein
37	Injury-Trauma,Condition	111697005	Injury of popliteal vein
37	Injury-Trauma,Condition	111729006	Disorder due to and following injury to nerve
37	Injury-Trauma,Condition	111731002	Late effect of injury to internal organ
37	Injury-Trauma,Condition	123534001	Closed fracture of sacrum AND/OR coccyx with cauda equina injury
37	Injury-Trauma,Condition	123535000	Open fracture of sacrum AND/OR coccyx with cauda equina injury
37	Injury-Trauma,Condition	125609005	Open fracture of cervical region with spinal cord injury

© 2021 Matheny ME et al. *JAMA Network Open.*

CONDITIONID	CONDITION_DESCRIPTION	SNOMED_CODE	DESCRIPTION
37	Injury-Trauma,Condition	125615005	Traumatic dislocation of shoulder region
37	Injury-Trauma,Condition	125617002	Traumatic dislocation of elbow joint
37	Injury-Trauma,Condition	125618007	Traumatic dislocation of joint of wrist
37	Injury-Trauma,Condition	125619004	Traumatic dislocation of joint of finger
37	Injury-Trauma,Condition	125621009	Traumatic dislocation of hip joint
37	Injury-Trauma,Condition	125622002	Traumatic dislocation of ankle joint
37	Injury-Trauma,Condition	125623007	Traumatic dislocation of joint of foot
37	Injury-Trauma,Condition	125643001	Open wound
37	Injury-Trauma,Condition	125665001	Crushing injury
37	Injury-Trauma,Condition	125802004	Closed traumatic dislocation of interphalangeal joint of finger
37	Injury-Trauma,Condition	127296001	Intracranial injury
37	Injury-Trauma,Condition	127297005	Intracranial injury with loss of consciousness
37	Injury-Trauma,Condition	127308007	Intracranial hemorrhage following injury with loss of consciousness
37	Injury-Trauma,Condition	127310009	Intracranial hemorrhage following injury with moderate loss of consciousness

© 2021 Matheny ME et al. *JAMA Network Open.*

CONDITIONID	CONDITION_DESCRIPTION	SNOMED_CODE	DESCRIPTION
37	Injury-Trauma,Condition	127311008	Intracranial hemorrhage following injury with prolonged loss of consciousness AND return to pre-existing conscious level
37	Injury-Trauma,Condition	127312001	Intracranial hemorrhage following injury with prolonged loss of consciousness without return to pre-existing conscious level
37	Injury-Trauma,Condition	127314000	Open wound of chest wall
37	Injury-Trauma,Condition	201932009	Traumatic arthropathy of the shoulder region
37	Injury-Trauma,Condition	201935006	Traumatic arthropathy of the hand
37	Injury-Trauma,Condition	201936007	Traumatic arthropathy of the pelvic region and thigh
37	Injury-Trauma,Condition	201938008	Traumatic arthropathy of the ankle and/or foot
37	Injury-Trauma,Condition	201940003	Traumatic arthropathy of multiple sites
37	Injury-Trauma,Condition	201944007	Traumatic arthropathy-elbow
37	Injury-Trauma,Condition	201952005	Traumatic arthropathy-knee

© 2021 Matheny ME et al. *JAMA Network Open.*

CONDITIONID	CONDITION_DESCRIPTION	SNOMED_CODE	DESCRIPTION
37	Injury-Trauma,Condition	202108004	Old lateral collateral ligament disruption
37	Injury-Trauma,Condition	202109007	Old medial collateral ligament disruption
37	Injury-Trauma,Condition	202110002	Old anterior cruciate ligament disruption
37	Injury-Trauma,Condition	202111003	Old posterior cruciate ligament disruption
37	Injury-Trauma,Condition	202133001	Articular cartilage disorder of shoulder region
37	Injury-Trauma,Condition	202137000	Articular cartilage disorder of the pelvic region and thigh
37	Injury-Trauma,Condition	202222009	Recurrent dislocation of elbow
37	Injury-Trauma,Condition	202230005	Recurrent dislocation of wrist
37	Injury-Trauma,Condition	202245003	Recurrent dislocation of knee
37	Injury-Trauma,Condition	207687004	Closed fracture vault of skull with intracranial injury
37	Injury-Trauma,Condition	207689001	Closed fracture of vault of skull with intracranial injury, with no loss of consciousness
37	Injury-Trauma,Condition	207690005	Closed fracture of vault of skull with intracranial injury, with less than 1 hour loss of consciousness

© 2021 Matheny ME et al. *JAMA Network Open.*

CONDITIONID	CONDITION_DESCRIPTION	SNOMED_CODE	DESCRIPTION
37	Injury-Trauma,Condition	207691009	Closed fracture of vault of skull with intracranial injury, with 1-24 hours loss of consciousness
37	Injury-Trauma,Condition	207692002	Closed fracture of vault of skull with intracranial injury, with more than 24 hours loss of consciousness and return to pre-existing conscious level
37	Injury-Trauma,Condition	207693007	Closed fracture of vault of skull with intracranial injury, with more than 24 hours loss of consciousness without return to pre-existing conscious level
37	Injury-Trauma,Condition	207705002	Open fracture vault of skull with intracranial injury
37	Injury-Trauma,Condition	207707005	Open fracture of vault of skull with intracranial injury, with no loss of consciousness
37	Injury-Trauma,Condition	207708000	Open fracture of vault of skull with intracranial injury, with less than 1 hour loss of consciousness
37	Injury-Trauma,Condition	207709008	Open fracture of vault of skull with intracranial injury,

© 2021 Matheny ME et al. *JAMA Network Open.*

CONDITIONID	CONDITION_DESCRIPTION	SNOMED_CODE	DESCRIPTION
			with 1-24 hours loss of consciousness
37	Injury-Trauma,Condition	207710003	Open fracture of vault of skull with intracranial injury, with more than 24 hours loss of consciousness and return to pre-existing conscious level
37	Injury-Trauma,Condition	207711004	Open fracture of vault of skull with intracranial injury, with more than 24 hours loss of consciousness without return to pre-existing conscious level
37	Injury-Trauma,Condition	207727006	Closed fracture of base of skull with intracranial injury, with no loss of consciousness
37	Injury-Trauma,Condition	207728001	Closed fracture of base of skull with intracranial injury, with less than 1 hour loss of consciousness
37	Injury-Trauma,Condition	207729009	Closed fracture of base of skull with intracranial injury, with 1-24 hours loss of consciousness

© 2021 Matheny ME et al. *JAMA Network Open.*

CONDITIONID	CONDITION_DESCRIPTION	SNOMED_CODE	DESCRIPTION
37	Injury-Trauma,Condition	207730004	Closed fracture of base of skull with intracranial injury, with more than 24 hours loss of consciousness and return to pre-existing conscious level
37	Injury-Trauma,Condition	207731000	Closed fracture of base of skull with intracranial injury, with more than 24 hours loss of consciousness without return to pre-existing conscious level
37	Injury-Trauma,Condition	207745006	Open fracture of base of skull with intracranial injury, with no loss of consciousness
37	Injury-Trauma,Condition	207746007	Open fracture of base of skull with intracranial injury, with less than 1 hour loss of consciousness
37	Injury-Trauma,Condition	207747003	Open fracture of base of skull with intracranial injury, with 1-24 hours loss of consciousness
37	Injury-Trauma,Condition	207748008	Open fracture of base of skull with intracranial injury, with more than 24 hours loss of consciousness and

© 2021 Matheny ME et al. *JAMA Network Open.*

CONDITIONID	CONDITION_DESCRIPTION	SNOMED_CODE	DESCRIPTION
			return to pre-existing conscious level
37	Injury-Trauma,Condition	207749000	Open fracture of base of skull with intracranial injury, with more than 24 hours loss of consciousness without return to pre-existing conscious level
37	Injury-Trauma,Condition	207881000	Multiple fractures involving skull and facial bones
37	Injury-Trauma,Condition	208000003	Closed spinal fracture with complete cervical cord lesion, C1-4
37	Injury-Trauma,Condition	208006009	Closed spinal fracture with complete cervical cord lesion, C5-7
37	Injury-Trauma,Condition	208014003	Open spinal fracture with complete cervical cord lesion, C1-4
37	Injury-Trauma,Condition	208020002	Open spinal fracture with complete cervical cord lesion, C5-7
37	Injury-Trauma,Condition	208028009	Closed spinal fracture with complete thoracic cord lesion, T1-6
37	Injury-Trauma,Condition	208034002	Closed spinal fracture with complete thoracic cord lesion,T7-12

© 2021 Matheny ME et al. *JAMA Network Open.*

CONDITIONID	CONDITION_DESCRIPTION	SNOMED_CODE	DESCRIPTION
37	Injury-Trauma,Condition	208042001	Open spinal fracture with complete thoracic cord lesion, T1-6
37	Injury-Trauma,Condition	208048002	Open spinal fracture with complete thoracic cord lesion, T7-12
37	Injury-Trauma,Condition	208748005	Open dislocation of jaw
37	Injury-Trauma,Condition	208759001	Closed traumatic dislocation acromioclavicular joint
37	Injury-Trauma,Condition	208766000	Open traumatic dislocation of glenohumeral joint, anterior
37	Injury-Trauma,Condition	208768004	Open traumatic dislocation of glenohumeral joint, inferior
37	Injury-Trauma,Condition	208769007	Open traumatic dislocation acromioclavicular joint
37	Injury-Trauma,Condition	208795009	Open traumatic dislocation elbow joint, anterior
37	Injury-Trauma,Condition	208796005	Open traumatic dislocation elbow joint, posterior
37	Injury-Trauma,Condition	208797001	Open traumatic dislocation elbow joint, medial
37	Injury-Trauma,Condition	208798006	Open traumatic dislocation elbow joint, lateral
37	Injury-Trauma,Condition	208826009	Open traumatic dislocation distal radioulnar joint

© 2021 Matheny ME et al. *JAMA Network Open.*

CONDITIONID	CONDITION_DESCRIPTION	SNOMED_CODE	DESCRIPTION
37	Injury-Trauma,Condition	208827000	Open traumatic dislocation radiocarpal joint
37	Injury-Trauma,Condition	208828005	Open traumatic dislocation midcarpal joint
37	Injury-Trauma,Condition	208829002	Open traumatic dislocation carpometacarpal joint
37	Injury-Trauma,Condition	208892001	Closed traumatic dislocation of hip
37	Injury-Trauma,Condition	208900000	Open traumatic dislocation hip joint, posterior
37	Injury-Trauma,Condition	208901001	Open traumatic obturator dislocation of hip
37	Injury-Trauma,Condition	208902008	Open traumatic dislocation hip joint, anterior
37	Injury-Trauma,Condition	208929003	Closed traumatic dislocation of patellofemoral joint
37	Injury-Trauma,Condition	208981003	Closed traumatic dislocation ankle joint
37	Injury-Trauma,Condition	208990005	Closed traumatic dislocation, midtarsal joint
37	Injury-Trauma,Condition	209000008	Open traumatic dislocation of foot
37	Injury-Trauma,Condition	209003005	Open traumatic dislocation, midtarsal joint
37	Injury-Trauma,Condition	209004004	Open traumatic dislocation of tarsometatarsal joint

© 2021 Matheny ME et al. *JAMA Network Open.*

CONDITIONID	CONDITION_DESCRIPTION	SNOMED_CODE	DESCRIPTION
37	Injury-Trauma,Condition	209052009	Closed dislocation of multiple cervical vertebrae
37	Injury-Trauma,Condition	209054005	Open dislocation of cervical spine
37	Injury-Trauma,Condition	209071006	Open dislocation of multiple cervical vertebrae
37	Injury-Trauma,Condition	209073009	Closed dislocation of thoracic and/or lumbar spine
37	Injury-Trauma,Condition	209074003	Closed dislocation lumbar spine
37	Injury-Trauma,Condition	209075002	Closed dislocation thoracic spine
37	Injury-Trauma,Condition	209088004	Open dislocation of thoracic and/or lumbar spine
37	Injury-Trauma,Condition	209090003	Open dislocation thoracic spine
37	Injury-Trauma,Condition	209112009	Open dislocation of sacrum
37	Injury-Trauma,Condition	209827006	Concussion with less than 1 hour loss of consciousness
37	Injury-Trauma,Condition	209828001	Concussion with 1-24 hours loss of consciousness
37	Injury-Trauma,Condition	209829009	Concussion with more than 24 hours loss of consciousness and return to pre-existing conscious level
37	Injury-Trauma,Condition	209830004	Concussion with more than 24 hours loss of consciousness without return to

© 2021 Matheny ME et al. *JAMA Network Open.*

CONDITIONID	CONDITION_DESCRIPTION	SNOMED_CODE	DESCRIPTION
			pre-existing conscious level
37	Injury-Trauma,Condition	209845006	Cortex contusion with open intracranial wound, with no loss of consciousness
37	Injury-Trauma,Condition	209847003	Cortex contusion with open intracranial wound, with less than 1 hour loss of consciousness
37	Injury-Trauma,Condition	209848008	Cortex contusion with open intracranial wound, with 1-24 hours loss of consciousness
37	Injury-Trauma,Condition	209849000	Cortex contusion with open intracranial wound, with more than 24 hours loss of consciousness and return to pre-existing conscious level
37	Injury-Trauma,Condition	209850000	Cortex contusion with open intracranial wound, with more than 24 hours loss of consciousness without return to pre-existing conscious level
37	Injury-Trauma,Condition	209864000	Cortex laceration with open intracranial wound,

© 2021 Matheny ME et al. *JAMA Network Open.*

CONDITIONID	CONDITION_DESCRIPTION	SNOMED_CODE	DESCRIPTION
			with no loss of consciousness
37	Injury-Trauma,Condition	209865004	Cortex laceration with open intracranial wound, with less than 1 hour loss of consciousness
37	Injury-Trauma,Condition	209866003	Cortex laceration with open intracranial wound, with 1-24 hours loss of consciousness
37	Injury-Trauma,Condition	209867007	Cortex laceration with open intracranial wound, with more than 24 hours loss of consciousness and return to pre-existing conscious level
37	Injury-Trauma,Condition	209868002	Cortex laceration with open intracranial wound, with more than 24 hours loss of consciousness without return to pre-existing conscious level
37	Injury-Trauma,Condition	209940000	Subarachnoid hemorrhage following injury with open intracranial wound, with no loss of consciousness

© 2021 Matheny ME et al. *JAMA Network Open.*

CONDITIONID	CONDITION_DESCRIPTION	SNOMED_CODE	DESCRIPTION
37	Injury-Trauma,Condition	209947002	Closed traumatic subdural hemorrhage
37	Injury-Trauma,Condition	209956005	Subdural hemorrhage following open wound of head
37	Injury-Trauma,Condition	209958006	Subdural hemorrhage following injury with open intracranial wound, with no loss of consciousness
37	Injury-Trauma,Condition	209978003	Extradural hemorrhage following injury with open intracranial wound, with no loss of consciousness
37	Injury-Trauma,Condition	210063008	Heart laceration with open wound into thorax, without penetration of heart chambers
37	Injury-Trauma,Condition	210064002	Heart laceration with open wound into thorax, with penetration of heart chambers
37	Injury-Trauma,Condition	210076002	Lung laceration with open wound into thorax
37	Injury-Trauma,Condition	210106000	Multiple intrathoracic organ injury with open wound into cavity

© 2021 Matheny ME et al. *JAMA Network Open.*

CONDITIONID	CONDITION_DESCRIPTION	SNOMED_CODE	DESCRIPTION
37	Injury-Trauma,Condition	210109007	Stomach injury with open wound into cavity
37	Injury-Trauma,Condition	210114006	Injury of small intestine with open wound into abdominal cavity
37	Injury-Trauma,Condition	210116008	Injury of duodenum with open wound into abdominal cavity
37	Injury-Trauma,Condition	210128000	Colon or rectum injury with open wound into cavity
37	Injury-Trauma,Condition	210130003	Injury of ascending right colon with open wound into abdominal cavity
37	Injury-Trauma,Condition	210131004	Injury of transverse colon with open wound into abdominal cavity
37	Injury-Trauma,Condition	210132006	Injury of descending left colon with open wound into abdominal cavity
37	Injury-Trauma,Condition	210133001	Sigmoid colon injury with open wound into cavity
37	Injury-Trauma,Condition	210155009	Injury of head of pancreas with open wound into abdominal cavity
37	Injury-Trauma,Condition	210156005	Pancreas body injury with open wound into cavity
37	Injury-Trauma,Condition	210157001	Injury of tail of pancreas with open wound into abdominal cavity

© 2021 Matheny ME et al. *JAMA Network Open.*

CONDITIONID	CONDITION_DESCRIPTION	SNOMED_CODE	DESCRIPTION
37	Injury-Trauma,Condition	210159003	Injury of appendix with open wound into abdominal cavity
37	Injury-Trauma,Condition	210173007	Liver hematoma and contusion with open wound into cavity
37	Injury-Trauma,Condition	210174001	Liver minor laceration with open wound into cavity
37	Injury-Trauma,Condition	210175000	Liver moderate laceration with open wound into cavity
37	Injury-Trauma,Condition	210191002	Spleen capsular tear without major disruption of parenchyma, with open wound into cavity
37	Injury-Trauma,Condition	210192009	Spleen laceration extending into parenchyma with open wound into cavity
37	Injury-Trauma,Condition	210205007	Kidney hematoma without rupture of capsule, with open wound into cavity
37	Injury-Trauma,Condition	210206008	Kidney laceration with open wound into cavity
37	Injury-Trauma,Condition	210207004	Complete disruption of kidney parenchyma with open wound into cavity
37	Injury-Trauma,Condition	210215001	Bladder and urethra injury with

© 2021 Matheny ME et al. *JAMA Network Open.*

CONDITIONID	CONDITION_DESCRIPTION	SNOMED_CODE	DESCRIPTION
			open wound into cavity
37	Injury-Trauma,Condition	210220001	Injury of ureter with open wound into abdominal cavity
37	Injury-Trauma,Condition	210260000	Injury of adrenal gland with open wound into abdominal cavity
37	Injury-Trauma,Condition	210262008	Peritoneum injury with open wound into cavity
37	Injury-Trauma,Condition	210263003	Retroperitoneum injury with open wound into cavity
37	Injury-Trauma,Condition	210790008	Pulmonary blood vessel injury
37	Injury-Trauma,Condition	210808001	Portal system vein injury
37	Injury-Trauma,Condition	210814008	Iliac blood vessel injury
37	Injury-Trauma,Condition	210816005	Injury of internal iliac vein
37	Injury-Trauma,Condition	210824000	Axillary blood vessel injury
37	Injury-Trauma,Condition	210835005	Injury of radial blood vessel
37	Injury-Trauma,Condition	210839004	Ulnar blood vessel injury
37	Injury-Trauma,Condition	210848009	Digital blood vessel injury
37	Injury-Trauma,Condition	210883008	Injury of tibial blood vessel
37	Injury-Trauma,Condition	210892006	Injury of deep plantar blood vessel
37	Injury-Trauma,Condition	211561006	Crush injury, upper arm, multiple sites
37	Injury-Trauma,Condition	211563009	Crush injury elbow or forearm

© 2021 Matheny ME et al. *JAMA Network Open.*

CONDITIONID	CONDITION_DESCRIPTION	SNOMED_CODE	DESCRIPTION
37	Injury-Trauma,Condition	211589009	Crush injury of hip and thigh
37	Injury-Trauma,Condition	211609000	Crush injury, toe(s)
37	Injury-Trauma,Condition	212183002	Lumbar cord injury without spinal bone injury
37	Injury-Trauma,Condition	239723003	Deficiency of ligaments of knee joint
37	Injury-Trauma,Condition	248222000	Consciousness unimpaired
37	Injury-Trauma,Condition	262791003	Contusion of esophagus
37	Injury-Trauma,Condition	262802005	Laceration of liver
37	Injury-Trauma,Condition	262812003	Bile duct injury without open wound into cavity
37	Injury-Trauma,Condition	262937009	Injury to blood vessel of neck
37	Injury-Trauma,Condition	263013004	Dislocation of joint of spine
37	Injury-Trauma,Condition	263025001	Dislocation of midcarpal joint
37	Injury-Trauma,Condition	269129003	Closed dislocation of sacrum
37	Injury-Trauma,Condition	269144002	Cerebral laceration and contusion
37	Injury-Trauma,Condition	269159008	Injury of pelvic organs
37	Injury-Trauma,Condition	269162006	Bile duct/gallbladder injury with open wound into cavity
37	Injury-Trauma,Condition	269224001	Crush injury, face, scalp and neck
37	Injury-Trauma,Condition	274170000	Open wound of abdominal wall
37	Injury-Trauma,Condition	275326005	Bucket handle tear of lateral meniscus of knee

© 2021 Matheny ME et al. *JAMA Network Open.*

CONDITIONID	CONDITION_DESCRIPTION	SNOMED_CODE	DESCRIPTION
37	Injury-Trauma,Condition	284006002	Injury of thoracic cavity
37	Injury-Trauma,Condition	287096003	Multiple dislocations
37	Injury-Trauma,Condition	307945003	Current tear of medial cartilage AND/OR meniscus of knee
37	Injury-Trauma,Condition	308849005	Current tear of lateral cartilage AND/OR meniscus of knee
37	Injury-Trauma,Condition	373410009	Open traumatic dislocation of joint
37	Injury-Trauma,Condition	398042001	Accidental dural puncture
37	Injury-Trauma,Condition	405753002	Cauda equina injury without spinal bone injury
37	Injury-Trauma,Condition	405759003	Cervical spinal cord injury without spinal bone injury
37	Injury-Trauma,Condition	410047003	Open dislocation of shoulder region
37	Injury-Trauma,Condition	410048008	Open traumatic dislocation of glenohumeral joint, posterior
37	Injury-Trauma,Condition	417662000	History of clinical finding in subject
37	Injury-Trauma,Condition	418764009	Fracture of bone of head
37	Injury-Trauma,Condition	419045004	Loss of consciousness
37	Injury-Trauma,Condition	429052003	Articular cartilage disorder of wrist
37	Injury-Trauma,Condition	429190007	Recurrent dislocation of hip
37	Injury-Trauma,Condition	429630006	Closed dislocation of radiocarpal joint

© 2021 Matheny ME et al. *JAMA Network Open.*

CONDITIONID	CONDITION_DESCRIPTION	SNOMED_CODE	DESCRIPTION
37	Injury-Trauma,Condition	445852002	Closed traumatic dislocation of joint
37	Injury-Trauma,Condition	450410005	Intracranial hemorrhage following injury
37	Injury-Trauma,Condition	450551009	Traumatic brain injury with prolonged loss of consciousness (more than 24 hours) and return to pre-existing conscious level
37	Injury-Trauma,Condition	450552002	Traumatic brain injury with prolonged loss of consciousness (more than 24 hours) without return to pre-existing conscious level
38	Vomiting,Condition	8765009	Hematemesis
38	Vomiting,Condition	14094001	Hyperemesis gravidarum
38	Vomiting,Condition	16932000	Nausea and vomiting
38	Vomiting,Condition	19569008	Mild hyperemesis gravidarum
38	Vomiting,Condition	37224001	Psychogenic vomiting
38	Vomiting,Condition	38331001	Late vomiting of pregnancy
38	Vomiting,Condition	74621002	Epidemic vomiting syndrome
38	Vomiting,Condition	90325002	Vomiting of pregnancy
38	Vomiting,Condition	196746003	Persistent vomiting
38	Vomiting,Condition	197130009	Vomiting after gastrointestinal tract surgery

© 2021 Matheny ME et al. *JAMA Network Open.*

CONDITIONID	CONDITION_DESCRIPTION	SNOMED_CODE	DESCRIPTION
38	Vomiting,Condition	199022003	Mild hyperemesis-delivered
38	Vomiting,Condition	199023008	Mild hyperemesis-not delivered
38	Vomiting,Condition	199025001	Hyperemesis gravidarum with metabolic disturbance
38	Vomiting,Condition	199027009	Hyperemesis gravidarum with metabolic disturbance - delivered
38	Vomiting,Condition	199028004	Hyperemesis gravidarum with metabolic disturbance - not delivered
38	Vomiting,Condition	199032005	Late pregnancy vomiting - delivered
38	Vomiting,Condition	199033000	Late pregnancy vomiting - not delivered
38	Vomiting,Condition	300366003	Vomit contains feces
39	Alcohol Use,Condition	281004	Dementia associated with alcoholism
39	Alcohol Use,Condition	2043009	Alcoholic gastritis
39	Alcohol Use,Condition	7052005	Alcohol hallucinosis
39	Alcohol Use,Condition	7916009	Alcoholic polyneuropathy
39	Alcohol Use,Condition	8635005	Alcohol withdrawal delirium
39	Alcohol Use,Condition	9953008	Acute alcoholic liver disease
39	Alcohol Use,Condition	15167005	Alcohol abuse
39	Alcohol Use,Condition	21000000	Idiosyncratic intoxication

© 2021 Matheny ME et al. *JAMA Network Open.*

CONDITIONID	CONDITION_DESCRIPTION	SNOMED_CODE	DESCRIPTION
39	Alcohol Use,Condition	25702006	Alcohol intoxication
39	Alcohol Use,Condition	27134008	Poisoning by alcohol deterrent
39	Alcohol Use,Condition	29212009	Alcohol-induced organic mental disorder
39	Alcohol Use,Condition	36558000	Fetal or neonatal effect of alcohol transmitted via placenta and/or breast milk
39	Alcohol Use,Condition	41083005	Alcohol-induced sleep disorder
39	Alcohol Use,Condition	41309000	Alcoholic liver damage
39	Alcohol Use,Condition	50325005	Alcoholic fatty liver
39	Alcohol Use,Condition	53936005	Alcohol-induced mood disorder
39	Alcohol Use,Condition	61144001	Alcohol-induced psychotic disorder with delusions
39	Alcohol Use,Condition	61401005	Gastric hemorrhage
39	Alcohol Use,Condition	66590003	Alcohol dependence
39	Alcohol Use,Condition	67426006	Toxic effect of alcohol
39	Alcohol Use,Condition	73097000	Alcohol amnestic disorder
39	Alcohol Use,Condition	82782008	Toxic effect of ethyl alcohol
39	Alcohol Use,Condition	83521008	Dilated cardiomyopathy secondary to alcohol
39	Alcohol Use,Condition	191480000	Alcohol withdrawal syndrome
39	Alcohol Use,Condition	191802004	Acute alcoholic intoxication in alcoholism

© 2021 Matheny ME et al. *JAMA Network Open.*

CONDITIONID	CONDITION_DESCRIPTION	SNOMED_CODE	DESCRIPTION
39	Alcohol Use,Condition	191804003	Continuous acute alcoholic intoxication in alcoholism
39	Alcohol Use,Condition	191805002	Episodic acute alcoholic intoxication in alcoholism
39	Alcohol Use,Condition	191806001	Acute alcoholic intoxication in remission, in alcoholism
39	Alcohol Use,Condition	191811004	Continuous chronic alcoholism
39	Alcohol Use,Condition	191812006	Episodic chronic alcoholism
39	Alcohol Use,Condition	191813001	Chronic alcoholism in remission
39	Alcohol Use,Condition	191882002	Nondependent alcohol abuse, continuous
39	Alcohol Use,Condition	191883007	Nondependent alcohol abuse, episodic
39	Alcohol Use,Condition	191884001	Nondependent alcohol abuse in remission
39	Alcohol Use,Condition	212809004	Methyl alcohol causing toxic effect
39	Alcohol Use,Condition	212813006	Toxic effect of isopropyl alcohol
39	Alcohol Use,Condition	216633005	Accidental poisoning by alcoholic beverage
39	Alcohol Use,Condition	216640006	Accidental poisoning by methyl alcohol
39	Alcohol Use,Condition	216645001	Accidental poisoning by isopropyl alcohol

© 2021 Matheny ME et al. *JAMA Network Open.*

CONDITIONID	CONDITION_DESCRIPTION	SNOMED_CODE	DESCRIPTION
39	Alcohol Use,Condition	269765000	Accidental poisoning by alcohol
39	Alcohol Use,Condition	287166006	Accidental poisoning with ethyl alcohol
39	Alcohol Use,Condition	417662000	History of clinical finding in subject
39	Alcohol Use,Condition	420054005	Alcoholic cirrhosis
39	Alcohol Use,Condition	429775004	Alcohol intake exceeds recommended daily limit
40	Anemia,Condition	4854004	Acquired hemolytic anemia
40	Anemia,Condition	5876000	Acquired pancytopenia
40	Anemia,Condition	16360009	delta beta Thalassemia
40	Anemia,Condition	19442009	Heterozygous thalassemia
40	Anemia,Condition	27342004	Anemia of pregnancy
40	Anemia,Condition	28975000	Constitutional aplastic anemia
40	Anemia,Condition	38911009	Hereditary hemolytic anemia
40	Anemia,Condition	40108008	Thalassemia
40	Anemia,Condition	41841004	Sideroblastic anemia
40	Anemia,Condition	45828008	Anemia in mother complicating pregnancy, childbirth AND/OR puerperium
40	Anemia,Condition	47739002	Myelophthisis
40	Anemia,Condition	49472006	Megaloblastic anemia due to vitamin B>12< deficiency

© 2021 Matheny ME et al. *JAMA Network Open.*

CONDITIONID	CONDITION_DESCRIPTION	SNOMED_CODE	DESCRIPTION
40	Anemia,Condition	49708008	Anemia of chronic renal failure
40	Anemia,Condition	50715003	Pure red cell aplasia
40	Anemia,Condition	52565000	Non megaloblastic anemia associated with nutritional deficiency
40	Anemia,Condition	53165003	Megaloblastic anemia
40	Anemia,Condition	63565007	Congenital anemia
40	Anemia,Condition	65959000	beta Thalassemia
40	Anemia,Condition	66612000	Nutritional anemia
40	Anemia,Condition	68913001	alpha Thalassemia
40	Anemia,Condition	84027009	Pernicious anemia
40	Anemia,Condition	85649008	Megaloblastic anemia due to folate deficiency
40	Anemia,Condition	87522002	Iron deficiency anemia
40	Anemia,Condition	111407006	Hemolytic uremic syndrome
40	Anemia,Condition	127034005	Pancytopenia
40	Anemia,Condition	191156009	Protein-deficiency anemia
40	Anemia,Condition	191170009	Hemolytic anemia due to glutathione metabolism disorder
40	Anemia,Condition	191216004	Non-autoimmune hemolytic anemia
40	Anemia,Condition	191265009	Anemia in neoplastic disease
40	Anemia,Condition	199244000	Anemia during pregnancy - baby delivered
40	Anemia,Condition	199246003	Anemia during pregnancy - baby not yet delivered
40	Anemia,Condition	234347009	Anemia of chronic disease

© 2021 Matheny ME et al. *JAMA Network Open.*

CONDITIONID	CONDITION_DESCRIPTION	SNOMED_CODE	DESCRIPTION
40	Anemia,Condition	234376007	Acquired red cell aplasia
40	Anemia,Condition	234392002	Hemoglobin E/beta thalassemia disease
40	Anemia,Condition	234401000	Erythrocyte enzyme deficiency
40	Anemia,Condition	267513007	Deficiency anemias
40	Anemia,Condition	267530009	Acute posthemorrhagic anemia
40	Anemia,Condition	271737000	Anemia
40	Anemia,Condition	306058006	Aplastic anemia
40	Anemia,Condition	371315009	Iron deficiency anemia secondary to inadequate dietary iron intake
40	Anemia,Condition	413533008	Anemia due to chronic blood loss
40	Anemia,Condition	413603009	Autoimmune hemolytic anemia
40	Anemia,Condition	416826005	Sickle cell-thalassemia disease with crisis
40	Anemia,Condition	417048006	Sickle cell-thalassemia disease without crisis
40	Anemia,Condition	429564000	Anemia due to chemotherapy
41	GI Bleeding,Condition	2367005	Acute hemorrhagic gastritis
41	GI Bleeding,Condition	4556007	Gastritis
41	GI Bleeding,Condition	8765009	Hematemesis
41	GI Bleeding,Condition	15238002	Esophageal bleeding
41	GI Bleeding,Condition	17067009	Acute gastric ulcer with hemorrhage AND with perforation but

© 2021 Matheny ME et al. *JAMA Network Open.*

CONDITIONID	CONDITION_DESCRIPTION	SNOMED_CODE	DESCRIPTION
			without obstruction
41	GI Bleeding,Condition	22157005	Acute peptic ulcer with hemorrhage but without obstruction
41	GI Bleeding,Condition	37372002	Upper gastrointestinal bleeding
41	GI Bleeding,Condition	46523000	Chronic gastrojejunal ulcer with hemorrhage AND with perforation but without obstruction
41	GI Bleeding,Condition	47064007	Acute peptic ulcer with hemorrhage AND with perforation but without obstruction
41	GI Bleeding,Condition	51847008	Acute duodenal ulcer with hemorrhage AND with perforation but without obstruction
41	GI Bleeding,Condition	55746001	Chronic peptic ulcer with hemorrhage AND with perforation but without obstruction
41	GI Bleeding,Condition	59515005	Acute gastrojejunal ulcer with hemorrhage but without obstruction
41	GI Bleeding,Condition	61401005	Gastric hemorrhage

© 2021 Matheny ME et al. *JAMA Network Open.*

CONDITIONID	CONDITION_DESCRIPTION	SNOMED_CODE	DESCRIPTION
41	GI Bleeding,Condition	62341002	Chronic duodenal ulcer with hemorrhage but without obstruction
41	GI Bleeding,Condition	62838000	Chronic gastrojejunal ulcer with hemorrhage
41	GI Bleeding,Condition	66673003	Acute gastrojejunal ulcer with hemorrhage AND with perforation but without obstruction
41	GI Bleeding,Condition	66767006	Acute duodenal ulcer with hemorrhage but without obstruction
41	GI Bleeding,Condition	70418001	Acute gastric ulcer with hemorrhage but without obstruction
41	GI Bleeding,Condition	74341002	Chronic gastric ulcer with hemorrhage AND with perforation but without obstruction
41	GI Bleeding,Condition	74474003	Gastrointestinal hemorrhage
41	GI Bleeding,Condition	76078009	Chronic gastric ulcer with hemorrhage but without obstruction
41	GI Bleeding,Condition	81142005	Chronic duodenal ulcer with hemorrhage AND with perforation

© 2021 Matheny ME et al. *JAMA Network Open.*

CONDITIONID	CONDITION_DESCRIPTION	SNOMED_CODE	DESCRIPTION
			but without obstruction
41	GI Bleeding,Condition	81518000	Chronic peptic ulcer with hemorrhage but without obstruction
41	GI Bleeding,Condition	405729008	Hematochezia
42	Cardiovascular Disease,Condition	1755008	Old myocardial infarction
42	Cardiovascular Disease,Condition	4557003	Preinfarction syndrome
42	Cardiovascular Disease,Condition	15258001	Subclavian steal syndrome
42	Cardiovascular Disease,Condition	20059004	Cerebral artery occlusion
42	Cardiovascular Disease,Condition	21454007	Subarachnoid hemorrhage
42	Cardiovascular Disease,Condition	34781003	Vertebral artery syndrome
42	Cardiovascular Disease,Condition	43658003	Vertebral artery obstruction
42	Cardiovascular Disease,Condition	54329005	Acute myocardial infarction of anterior wall
42	Cardiovascular Disease,Condition	57054005	Acute myocardial infarction
42	Cardiovascular Disease,Condition	58612006	Acute myocardial infarction of lateral wall
42	Cardiovascular Disease,Condition	59021001	Angina decubitus
42	Cardiovascular Disease,Condition	63739005	Coronary occlusion
42	Cardiovascular Disease,Condition	64009001	Basilar artery syndrome
42	Cardiovascular Disease,Condition	65547006	Acute myocardial infarction of inferolateral wall
42	Cardiovascular Disease,Condition	66189004	Postmyocardial infarction syndrome

© 2021 Matheny ME et al. *JAMA Network Open.*

CONDITIONID	CONDITION_DESCRIPTION	SNOMED_CODE	DESCRIPTION
42	Cardiovascular Disease,Condition	69798007	Carotid artery obstruction
42	Cardiovascular Disease,Condition	70211005	Acute myocardial infarction of anterolateral wall
42	Cardiovascular Disease,Condition	70422006	Acute subendocardial infarction
42	Cardiovascular Disease,Condition	71444005	Cerebral thrombosis
42	Cardiovascular Disease,Condition	73795002	Acute myocardial infarction of inferior wall
42	Cardiovascular Disease,Condition	75543006	Cerebral embolism
42	Cardiovascular Disease,Condition	76593002	Acute myocardial infarction of inferoposterior wall
42	Cardiovascular Disease,Condition	87343002	Prinzmetal angina
42	Cardiovascular Disease,Condition	194802003	True posterior myocardial infarction
42	Cardiovascular Disease,Condition	194809007	Acute myocardial infarction of atrium
42	Cardiovascular Disease,Condition	194828000	Angina pectoris
42	Cardiovascular Disease,Condition	194861007	Certain current complications following acute myocardial infarction
42	Cardiovascular Disease,Condition	194863005	Atrial septal defect due to and following acute myocardial infarction
42	Cardiovascular Disease,Condition	195180004	Basilar artery occlusion
42	Cardiovascular Disease,Condition	195183002	Multiple and bilateral precerebral arterial occlusion

© 2021 Matheny ME et al. *JAMA Network Open.*

CONDITIONID	CONDITION_DESCRIPTION	SNOMED_CODE	DESCRIPTION
42	Cardiovascular Disease,Condition	195189003	Cerebral infarction due to thrombosis of cerebral arteries
42	Cardiovascular Disease,Condition	195190007	Cerebral infarction due to embolism of cerebral arteries
42	Cardiovascular Disease,Condition	195199008	Vertebrobasilar artery syndrome
42	Cardiovascular Disease,Condition	230692004	Infarction - precerebral
42	Cardiovascular Disease,Condition	266253001	Precerebral arterial occlusion
42	Cardiovascular Disease,Condition	266257000	Transient cerebral ischemia
42	Cardiovascular Disease,Condition	274100004	Cerebral hemorrhage
42	Cardiovascular Disease,Condition	288723005	Acute ill-defined cerebrovascular disease
42	Cardiovascular Disease,Condition	414545008	Ischemic heart disease
43	Organ Transplant,Procedure	161665007	History of renal transplant
43	Organ Transplant,Procedure	161666008	H/O: heart recipient
43	Organ Transplant,Procedure	161671001	H/O: liver recipient
43	Organ Transplant,Procedure	161672008	H/O: lung recipient
43	Organ Transplant,Procedure	416940007	Past history of procedure
44	HIV,Condition	79019005	Human immunodeficiency virus II infection
44	HIV,Condition	86406008	Human immunodeficiency virus infection
44	HIV,Condition	91947003	Asymptomatic human immunodeficiency virus infection

© 2021 Matheny ME et al. *JAMA Network Open.*

CONDITIONID	CONDITION_DESCRIPTION	SNOMED_CODE	DESCRIPTION
45	COPD/Asthma,Condition	12428000	Intrinsic asthma without status asthmaticus
45	COPD/Asthma,Condition	31387002	Exercise-induced asthma
45	COPD/Asthma,Condition	57686001	Emphysematous bleb of lung
45	COPD/Asthma,Condition	63480004	Chronic bronchitis
45	COPD/Asthma,Condition	74417001	Mucopurulent chronic bronchitis
45	COPD/Asthma,Condition	87433001	Pulmonary emphysema
45	COPD/Asthma,Condition	89549007	Catarrhal bronchitis
45	COPD/Asthma,Condition	171230000	Chronic obstructive pulmonary disease screening
45	COPD/Asthma,Condition	185086009	Emphysematous bronchitis
45	COPD/Asthma,Condition	195949008	Chronic asthmatic bronchitis
45	COPD/Asthma,Condition	195951007	Acute exacerbation of chronic obstructive airways disease
45	COPD/Asthma,Condition	195967001	Asthma
45	COPD/Asthma,Condition	266361008	Intrinsic asthma
45	COPD/Asthma,Condition	281239006	Exacerbation of asthma
45	COPD/Asthma,Condition	285381006	Acute infective exacerbation of chronic obstructive airways disease
45	COPD/Asthma,Condition	409663006	Cough variant asthma
45	COPD/Asthma,Condition	416471007	Family history of clinical finding
45	COPD/Asthma,Condition	424643009	IgE-mediated allergic asthma
45	COPD/Asthma,Condition	442025000	Acute exacerbation of chronic

© 2021 Matheny ME et al. *JAMA Network Open.*

CONDITIONID	CONDITION_DESCRIPTION	SNOMED_CODE	DESCRIPTION
			asthmatic bronchitis
45	COPD/Asthma,Condition	708090002	Acute severe exacerbation of asthma
45	COPD/Asthma,Condition	708093000	Acute exacerbation of allergic asthma
45	COPD/Asthma,Condition	708094006	Acute exacerbation of intrinsic asthma
45	COPD/Asthma,Condition	708095007	Acute severe exacerbation of immunoglobin E-mediated allergic asthma
45	COPD/Asthma,Condition	708096008	Acute severe exacerbation of intrinsic asthma
46	Hypotension,Condition	28651003	Orthostatic hypotension
46	Hypotension,Condition	45007003	Low blood pressure
46	Hypotension,Condition	77545000	Chronic hypotension
46	Hypotension,Condition	234171009	Drug-induced hypotension
46	Hypotension,Condition	408667000	Hemodialysis-associated hypotension
46	Hypotension,Condition	408668005	Iatrogenic hypotension
47	Dyslipidemia,Condition	10741005	Lipid storage disease
47	Dyslipidemia,Condition	55822004	Hyperlipidemia
47	Dyslipidemia,Condition	71325002	Lipodystrophy
47	Dyslipidemia,Condition	267431006	Disorder of lipid metabolism
47	Dyslipidemia,Condition	267432004	Pure hypercholesterolemia
47	Dyslipidemia,Condition	267433009	Pure hyperglyceridemia

© 2021 Matheny ME et al. *JAMA Network Open.*

CONDITIONID	CONDITION_DESCRIPTION	SNOMED_CODE	DESCRIPTION
47	Dyslipidemia,Condition	267434003	Mixed hyperlipidemia
47	Dyslipidemia,Condition	267435002	Familial hyperchylomicronemia
47	Dyslipidemia,Condition	267436001	Lipoprotein deficiency disorder
48	CABG,Procedure	251019006	Coronary bypass graft finding
48	CABG,Procedure	442421004	Arteriosclerosis of arterial coronary artery bypass graft
49	Tobacco Use,Condition	89765005	Tobacco dependence syndrome
50	Mitral Regurgitation,Procedure	11851006	Mitral valve disorder
50	Mitral Regurgitation,Procedure	29928006	Congenital insufficiency of mitral valve
50	Mitral Regurgitation,Procedure	194736003	Mitral and aortic incompetence
51	STEMI, Condition	54329005	Acute myocardial infarction of anterior wall
51	STEMI, Condition	57054005	Acute myocardial infarction
51	STEMI, Condition	58612006	Acute myocardial infarction of lateral wall
51	STEMI, Condition	65547006	Acute myocardial infarction of inferolateral wall
51	STEMI, Condition	70211005	Acute myocardial infarction of anterolateral wall
51	STEMI, Condition	73795002	Acute myocardial infarction of inferior wall
51	STEMI, Condition	76593002	Acute myocardial infarction of

© 2021 Matheny ME et al. *JAMA Network Open.*

CONDITIONID	CONDITION_DESCRIPTION	SNOMED_CODE	DESCRIPTION
			inferoposterior wall
51	STEMI, Condition	194802003	True posterior myocardial infarction
51	STEMI, Condition	194809007	Acute myocardial infarction of atrium
52	NSTEMI, Condition	70422006	Acute subendocardial infarction
53	Unstable Angina, Condition	4557003	Preinfarction syndrome
53	Unstable Angina, Condition	59021001	Angina decubitus
53	Unstable Angina, Condition	87343002	Prinzmetal angina
53	Unstable Angina, Condition	194828000	Angina pectoris
55	PCI, Procedure	371822007	Patient post percutaneous transluminal coronary angioplasty
56	Sickle Cell Disease, Condition	65959000	beta Thalassemia
56	Sickle Cell Disease, Condition	68913001	alpha Thalassemia
56	Sickle Cell Disease, Condition	127040003	Sickle cell-hemoglobin SS disease
56	Sickle Cell Disease, Condition	416180004	Hemoglobin SS disease without crisis
56	Sickle Cell Disease, Condition	416826005	Sickle cell-thalassemia disease with crisis
56	Sickle Cell Disease, Condition	417048006	Sickle cell-thalassemia disease without crisis
56	Sickle Cell Disease, Condition	417425009	Hemoglobin SS disease with crisis
56	Sickle Cell Disease, Condition	417517009	Sickle cell-hemoglobin C disease with crisis

© 2021 Matheny ME et al. *JAMA Network Open.*

CONDITIONID	CONDITION_DESCRIPTION	SNOMED_CODE	DESCRIPTION
56	Sickle Cell Disease, Condition	417683006	Sickle cell-hemoglobin C disease without crisis
57	Diabetic Ketoacidosis, Condition	8801005	Secondary diabetes mellitus
57	Diabetic Ketoacidosis, Condition	420270002	Ketoacidosis in type 1 diabetes mellitus
57	Diabetic Ketoacidosis, Condition	420422005	Diabetic ketoacidosis
57	Diabetic Ketoacidosis, Condition	421750000	Ketoacidosis in type 2 diabetes mellitus
58	Diabetic w/ Hyperosmolarity, Condition	8801005	Secondary diabetes mellitus
58	Diabetic w/ Hyperosmolarity, Condition	20313009	Hyperosmolality
58	Diabetic w/ Hyperosmolarity, Condition	190329007	Diabetes mellitus with hyperosmolar coma
58	Diabetic w/ Hyperosmolarity, Condition	190330002	Type 1 diabetes mellitus with hyperosmolar coma
58	Diabetic w/ Hyperosmolarity, Condition	190331003	Type 2 diabetes mellitus with hyperosmolar coma
58	Diabetic w/ Hyperosmolarity, Condition	428896009	Hyperosmolality due to uncontrolled type 1 diabetes mellitus
59	Bone Marrow Transplant, Procedure	416940007	Past history of procedure
59	Bone Marrow Transplant, Procedure	417089009	Limbal stem cell deficiency
59	Bone Marrow Transplant, Procedure	428103008	Disorder of transplanted bone marrow
60	Acute GLOMERULONEPHRITIS, Condition	19351000	Acute glomerulonephritis

© 2021 Matheny ME et al. *JAMA Network Open.*

CONDITIONID	CONDITION_DESCRIPTION	SNOMED_CODE	DESCRIPTION
60	Acute GLOMERULONEPHRITIS, Condition	197579006	Acute proliferative glomerulonephritis
60	Acute GLOMERULONEPHRITIS, Condition	197580009	Acute nephritis with lesions of necrotizing glomerulitis
60	Acute GLOMERULONEPHRITIS, Condition	197582001	Acute glomerulonephritis associated with another disorder
61	Dementia w/o Delirium, Condition	12348006	Presenile dementia
61	Dementia w/o Delirium, Condition	13092008	Pick's disease
61	Dementia w/o Delirium, Condition	26929004	Alzheimer's disease
61	Dementia w/o Delirium, Condition	45864009	Senile degeneration of brain
61	Dementia w/o Delirium, Condition	52448006	Dementia
61	Dementia w/o Delirium, Condition	191449005	Uncomplicated senile dementia
61	Dementia w/o Delirium, Condition	191451009	Uncomplicated presenile dementia
61	Dementia w/o Delirium, Condition	191452002	Presenile dementia with delirium
61	Dementia w/o Delirium, Condition	191455000	Presenile dementia with depression
61	Dementia w/o Delirium, Condition	191457008	Senile dementia with depressive or paranoid features
61	Dementia w/o Delirium, Condition	191459006	Senile dementia with depression
61	Dementia w/o Delirium, Condition	191461002	Senile dementia with delirium
61	Dementia w/o Delirium, Condition	191463004	Uncomplicated arteriosclerotic dementia
61	Dementia w/o Delirium, Condition	191464005	Arteriosclerotic dementia with delirium
61	Dementia w/o Delirium, Condition	191465006	Arteriosclerotic dementia with paranoia

© 2021 Matheny ME et al. *JAMA Network Open.*

CONDITIONID	CONDITION_DESCRIPTION	SNOMED_CODE	DESCRIPTION
61	Dementia w/o Delirium, Condition	191466007	Arteriosclerotic dementia with depression
61	Dementia w/o Delirium, Condition	191519005	Dementia associated with another disease
61	Dementia w/o Delirium, Condition	230270009	Frontotemporal dementia
61	Dementia w/o Delirium, Condition	231438001	Presbyophrenic psychosis
61	Dementia w/o Delirium, Condition	278857002	Dementia of frontal lobe type
61	Dementia w/o Delirium, Condition	371024007	Senile dementia with delusion
61	Dementia w/o Delirium, Condition	429998004	Vascular dementia
65	Acute Tubular Necrosis, Condition	35455006	Acute tubular necrosis
66	Decompensated Cirrhosis, Condition	1761006	Biliary cirrhosis
66	Decompensated Cirrhosis, Condition	9953008	Acute alcoholic liver disease
66	Decompensated Cirrhosis, Condition	13920009	Hepatic encephalopathy
66	Decompensated Cirrhosis, Condition	14223005	Esophageal varices without bleeding
66	Decompensated Cirrhosis, Condition	17709002	Bleeding esophageal varices
66	Decompensated Cirrhosis, Condition	34742003	Portal hypertension
66	Decompensated Cirrhosis, Condition	41309000	Alcoholic liver damage
66	Decompensated Cirrhosis, Condition	41889008	Chronic persistent hepatitis
66	Decompensated Cirrhosis, Condition	50325005	Alcoholic fatty liver
66	Decompensated Cirrhosis, Condition	51292008	Hepatorenal syndrome
66	Decompensated Cirrhosis, Condition	67656006	Portal pyemia
66	Decompensated Cirrhosis, Condition	76783007	Chronic hepatitis
66	Decompensated Cirrhosis, Condition	79720007	Chronic nonalcoholic liver disease

© 2021 Matheny ME et al. *JAMA Network Open.*

CONDITIONID	CONDITION_DESCRIPTION	SNOMED_CODE	DESCRIPTION
66	Decompensated Cirrhosis, Condition	195475003	Esophageal varices with bleeding, associated with another disorder
66	Decompensated Cirrhosis, Condition	235856003	Disease of liver
66	Decompensated Cirrhosis, Condition	266468003	Cirrhosis - non-alcoholic
66	Decompensated Cirrhosis, Condition	389026000	Ascites
66	Decompensated Cirrhosis, Condition	408335007	Autoimmune hepatitis
66	Decompensated Cirrhosis, Condition	420054005	Alcoholic cirrhosis
67	Urinary Obstruction, Condition	7163005	Urinary tract obstruction
67	Urinary Obstruction, Condition	20018005	Occlusion of ureter
67	Urinary Obstruction, Condition	20342001	Calculus in urethra
67	Urinary Obstruction, Condition	31054009	Ureteric stone
67	Urinary Obstruction, Condition	76618002	Urethral stricture
67	Urinary Obstruction, Condition	79509009	Calculus of lower urinary tract
67	Urinary Obstruction, Condition	86347007	Traumatic urethral stricture
67	Urinary Obstruction, Condition	111411000	Postoperative urethral stricture
67	Urinary Obstruction, Condition	204967008	Renal pelvis and ureter obstructive defects
67	Urinary Obstruction, Condition	204998005	Urethra and bladder neck atresia and stenosis
67	Urinary Obstruction, Condition	236646007	Benign prostatic hypertrophy with outflow obstruction
67	Urinary Obstruction, Condition	252025007	Incompetent urethral closure mechanism
67	Urinary Obstruction, Condition	265557005	Attention to nephrostomy tube
67	Urinary Obstruction, Condition	267064002	Retention of urine
67	Urinary Obstruction, Condition	302113004	Urostomy present

© 2021 Matheny ME et al. *JAMA Network Open.*

CONDITIONID	CONDITION_DESCRIPTION	SNOMED_CODE	DESCRIPTION
67	Urinary Obstruction, Condition	373584008	Congenital pelviureteric junction obstruction
67	Urinary Obstruction, Condition	373585009	Congenital ureterovesical obstruction
67	Urinary Obstruction, Condition	399072004	Bladder neck obstruction
70	Heart Transplant,Procedure	161666008	H/O: heart recipient
71	Liver Transplant,Procedure	161671001	H/O: liver recipient
72	Lung Transplant,Procedure	161672008	H/O: lung recipient
74	Nephritis Glomerular Not Specified,Condition	52845002	Nephritis
74	Nephritis Glomerular Not Specified,Condition	197582001	Acute glomerulonephritis associated with another disorder
75	Atrial Fibulation, Condition	5370000	Atrial flutter
75	Atrial Fibulation, Condition	49436004	Atrial fibrillation
76	Hypertension Emergency, Condition	54225002	Malignant hypertensive heart disease
76	Hypertension Emergency, Condition	65443008	Malignant hypertensive renal disease
76	Hypertension Emergency, Condition	66610008	Malignant hypertensive heart AND renal disease
76	Hypertension Emergency, Condition	78975002	Malignant essential hypertension
76	Hypertension Emergency, Condition	83105008	Malignant hypertensive heart disease with congestive heart failure
76	Hypertension Emergency, Condition	89242004	Malignant secondary hypertension

© 2021 Matheny ME et al. *JAMA Network Open.*

CONDITIONID	CONDITION_DESCRIPTION	SNOMED_CODE	DESCRIPTION
76	Hypertension Emergency, Condition	194779001	Hypertensive heart and renal disease with (congestive) heart failure
76	Hypertension Emergency, Condition	194780003	Hypertensive heart and renal disease with renal failure
76	Hypertension Emergency, Condition	194781004	Hypertensive heart and renal disease with both (congestive) heart failure and renal failure
76	Hypertension Emergency, Condition	194783001	Malignant secondary renovascular hypertension
76	Hypertension Emergency, Condition	285841000119104	Malignant hypertensive end stage renal disease
77	Glomerular Nephritis (Exclusion), Condition	19351000	Acute glomerulonephritis
77	Glomerular Nephritis (Exclusion), Condition	77182004	Membranous glomerulonephritis
77	Glomerular Nephritis (Exclusion), Condition	80321008	Mesangiocapillary glomerulonephritis
77	Glomerular Nephritis (Exclusion), Condition	197579006	Acute proliferative glomerulonephritis
77	Glomerular Nephritis (Exclusion), Condition	197580009	Acute nephritis with lesions of necrotizing glomerulitis
77	Glomerular Nephritis (Exclusion), Condition	197582001	Acute glomerulonephritis associated with another disorder
77	Glomerular Nephritis (Exclusion), Condition	197589005	Nephrotic syndrome with proliferative glomerulonephritis

© 2021 Matheny ME et al. *JAMA Network Open.*

CONDITIONID	CONDITION_DESCRIPTION	SNOMED_CODE	DESCRIPTION
77	Glomerular Nephritis (Exclusion), Condition	197590001	Nephrotic syndrome with membranous glomerulonephritis
77	Glomerular Nephritis (Exclusion), Condition	197591002	Nephrotic syndrome with membranoproliferative glomerulonephritis
77	Glomerular Nephritis (Exclusion), Condition	236392004	Rapidly progressive glomerulonephritis
77	Glomerular Nephritis (Exclusion), Condition	266549004	Nephrotic syndrome with minimal change glomerulonephritis
78	Acute Renal Failure, Condition	13010001	Acute renal failure following labor AND/OR delivery
78	Acute Renal Failure, Condition	14669001	Acute renal failure syndrome
78	Acute Renal Failure, Condition	35455006	Acute tubular necrosis
78	Acute Renal Failure, Condition	200117009	Post-delivery acute renal failure - delivered with postnatal problem
78	Acute Renal Failure, Condition	200118004	Post-delivery acute renal failure with postnatal problem
78	Acute Renal Failure, Condition	298015003	Acute renal papillary necrosis with renal failure
78	Acute Renal Failure, Condition	429224003	Acute renal failure due to acute cortical necrosis
79	CHF, Condition	5148006	Hypertensive heart disease with congestive heart failure
79	CHF, Condition	42343007	Congestive heart failure

© 2021 Matheny ME et al. *JAMA Network Open.*

CONDITIONID	CONDITION_DESCRIPTION	SNOMED_CODE	DESCRIPTION
79	CHF, Condition	45227007	Hypertrophic obstructive cardiomyopathy
79	CHF, Condition	77737007	Benign hypertensive heart disease with congestive heart failure
79	CHF, Condition	82523003	Congestive rheumatic heart failure
79	CHF, Condition	83105008	Malignant hypertensive heart disease with congestive heart failure
79	CHF, Condition	83521008	Dilated cardiomyopathy secondary to alcohol
79	CHF, Condition	84114007	Heart failure
79	CHF, Condition	85232009	Left heart failure
79	CHF, Condition	85898001	Cardiomyopathy
79	CHF, Condition	194779001	Hypertensive heart and renal disease with (congestive) heart failure
79	CHF, Condition	194781004	Hypertensive heart and renal disease with both (congestive) heart failure and renal failure
79	CHF, Condition	195023001	Nutritional and metabolic cardiomyopathies
79	CHF, Condition	195029002	Cardiomyopathy associated with another disorder
79	CHF, Condition	233873004	Hypertrophic cardiomyopathy

© 2021 Matheny ME et al. *JAMA Network Open.*

CONDITIONID	CONDITION_DESCRIPTION	SNOMED_CODE	DESCRIPTION
79	CHF, Condition	417996009	Systolic heart failure
79	CHF, Condition	418304008	Diastolic heart failure
79	CHF, Condition	441481004	Chronic systolic heart failure
79	CHF, Condition	441530006	Chronic diastolic heart failure
79	CHF, Condition	442304009	Combined systolic and diastolic dysfunction
79	CHF, Condition	443253003	Acute on chronic systolic heart failure
79	CHF, Condition	443254009	Acute systolic heart failure
79	CHF, Condition	443343001	Acute diastolic heart failure
79	CHF, Condition	443344007	Acute on chronic diastolic heart failure
79	CHF, Condition	153931000119109	Acute combined systolic and diastolic heart failure
79	CHF, Condition	153941000119100	Chronic combined systolic and diastolic heart failure
79	CHF, Condition	153951000119103	Acute on chronic combined systolic and diastolic heart failure
80	Stroke, Condition	4069002	Anoxic brain damage during AND/OR resulting from a procedure
80	Stroke, Condition	20059004	Cerebral artery occlusion
80	Stroke, Condition	21454007	Subarachnoid hemorrhage

© 2021 Matheny ME et al. *JAMA Network Open.*

CONDITIONID	CONDITION_DESCRIPTION	SNOMED_CODE	DESCRIPTION
80	Stroke, Condition	71444005	Cerebral thrombosis
80	Stroke, Condition	75543006	Cerebral embolism
80	Stroke, Condition	195189003	Cerebral infarction due to thrombosis of cerebral arteries
80	Stroke, Condition	195190007	Cerebral infarction due to embolism of cerebral arteries
80	Stroke, Condition	274100004	Cerebral hemorrhage
80	Stroke, Condition	288723005	Acute ill-defined cerebrovascular disease
81	TIA, Condition	266257000	Transient cerebral ischemia
82	Abdominal Aortic Aneurysm, Procedure	75878002	Abdominal aortic aneurysm without rupture
83	Carotid Disease, Condition	69798007	Carotid artery obstruction
91	Angina, Condition	4557003	Preinfarction syndrome
91	Angina, Condition	59021001	Angina decubitus
91	Angina, Condition	87343002	Prinzmetal angina
91	Angina, Condition	194828000	Angina pectoris
93	Advanced Liver Disease,Condition	1761006	Biliary cirrhosis
93	Advanced Liver Disease,Condition	13920009	Hepatic encephalopathy
93	Advanced Liver Disease,Condition	14223005	Esophageal varices without bleeding
93	Advanced Liver Disease,Condition	17709002	Bleeding esophageal varices
93	Advanced Liver Disease,Condition	34742003	Portal hypertension
93	Advanced Liver Disease,Condition	41309000	Alcoholic liver damage
93	Advanced Liver Disease,Condition	51292008	Hepatorenal syndrome
93	Advanced Liver Disease,Condition	186624004	Acute hepatitis B with delta agent

© 2021 Matheny ME et al. *JAMA Network Open.*

CONDITIONID	CONDITION_DESCRIPTION	SNOMED_CODE	DESCRIPTION
			(coinfection) with hepatic coma
93	Advanced Liver Disease,Condition	186628001	Viral hepatitis C with coma
93	Advanced Liver Disease,Condition	195475003	Esophageal varices with bleeding, associated with another disorder
93	Advanced Liver Disease,Condition	195476002	Esophageal varices without bleeding, associated with another disorder
93	Advanced Liver Disease,Condition	235856003	Disease of liver
93	Advanced Liver Disease,Condition	266468003	Cirrhosis - non-alcoholic
93	Advanced Liver Disease,Condition	420054005	Alcoholic cirrhosis
93	Advanced Liver Disease,Condition	424340000	Hepatic coma due to chronic hepatitis B
94	Pallative Care,Condition	103735009	Palliative care
95	Infection (sepsis sup),Condition	3419005	Faucial diphtheria
95	Infection (sepsis sup),Condition	4089001	Meningococcemia
95	Infection (sepsis sup),Condition	5758002	Bacteremia
95	Infection (sepsis sup),Condition	6365004	Cutaneous actinomycosis
95	Infection (sepsis sup),Condition	7587000	Acute osteomyelitis of shoulder region
95	Infection (sepsis sup),Condition	7773002	Conjunctival diphtheria
95	Infection (sepsis sup),Condition	8725005	Abscess of prostate
95	Infection (sepsis sup),Condition	8872002	Osteomyelitis of pelvic region
95	Infection (sepsis sup),Condition	9467007	Meningococcal arthropathy
95	Infection (sepsis sup),Condition	9713002	Prostatitis
95	Infection (sepsis sup),Condition	10188004	Acute osteomyelitis of hand
95	Infection (sepsis sup),Condition	11817007	Actinomycotic infection

© 2021 Matheny ME et al. *JAMA Network Open.*

CONDITIONID	CONDITION_DESCRIPTION	SNOMED_CODE	DESCRIPTION
95	Infection (sepsis sup),Condition	12295008	Bronchiectasis
95	Infection (sepsis sup),Condition	12890007	Periostitis of ankle AND/OR foot
95	Infection (sepsis sup),Condition	13325005	Chronic osteomyelitis of hand
95	Infection (sepsis sup),Condition	13596001	Diphtheritic peritonitis
95	Infection (sepsis sup),Condition	14386001	Indeterminate leprosy
95	Infection (sepsis sup),Condition	15682004	Anterior nasal diphtheria
95	Infection (sepsis sup),Condition	16594005	Periostitis of multiple sites
95	Infection (sepsis sup),Condition	17028003	Osteomyelitis of forearm
95	Infection (sepsis sup),Condition	18071005	Meningococcal encephalitis
95	Infection (sepsis sup),Condition	18901009	Cutaneous diphtheria
95	Infection (sepsis sup),Condition	19905009	Chronic prostatitis
95	Infection (sepsis sup),Condition	19956000	Acute osteomyelitis of forearm
95	Infection (sepsis sup),Condition	20358003	Pyogenic arthritis of ankle
95	Infection (sepsis sup),Condition	21120002	Osteomyelitis of lower leg
95	Infection (sepsis sup),Condition	21560005	Lepromatous leprosy
95	Infection (sepsis sup),Condition	21846001	Pulmonary actinomycosis
95	Infection (sepsis sup),Condition	23014006	Cervicofacial actinomycosis
95	Infection (sepsis sup),Condition	23301003	Infection following infusion, injection, transfusion AND/OR vaccination
95	Infection (sepsis sup),Condition	23511006	Meningococcal infectious disease

© 2021 Matheny ME et al. *JAMA Network Open.*

CONDITIONID	CONDITION_DESCRIPTION	SNOMED_CODE	DESCRIPTION
95	Infection (sepsis sup),Condition	24557004	Abscess of intestine
95	Infection (sepsis sup),Condition	25626000	Cutaneous infectious disease due to Mycobacteria
95	Infection (sepsis sup),Condition	26117009	Diphtheritic myocarditis
95	Infection (sepsis sup),Condition	27836007	Pertussis
95	Infection (sepsis sup),Condition	28769004	Osteomyelitis of ankle AND/OR foot
95	Infection (sepsis sup),Condition	30242009	Scarlet fever
95	Infection (sepsis sup),Condition	31871009	Infection AND/OR inflammatory reaction due to internal prosthetic device, implant AND/OR graft
95	Infection (sepsis sup),Condition	32636009	Periostitis of shoulder region
95	Infection (sepsis sup),Condition	33618002	Meningococcal carditis
95	Infection (sepsis sup),Condition	33910007	Postoperative infection
95	Infection (sepsis sup),Condition	34845009	Meningococcal endocarditis
95	Infection (sepsis sup),Condition	34934005	Pyogenic arthritis of multiple sites
95	Infection (sepsis sup),Condition	36102002	Waterhouse-Friderichsen syndrome
95	Infection (sepsis sup),Condition	36678001	Pyogenic arthritis of shoulder region
95	Infection (sepsis sup),Condition	40198004	Thrombophlebitis of deep veins of lower extremity
95	Infection (sepsis sup),Condition	40283005	Thrombophlebitis of superficial veins of lower extremity
95	Infection (sepsis sup),Condition	40970001	Chronic osteomyelitis

© 2021 Matheny ME et al. *JAMA Network Open.*

CONDITIONID	CONDITION_DESCRIPTION	SNOMED_CODE	DESCRIPTION
95	Infection (sepsis sup),Condition	43878008	Streptococcal sore throat
95	Infection (sepsis sup),Condition	43894001	Abdominal actinomycosis
95	Infection (sepsis sup),Condition	44653001	Erysipelas
95	Infection (sepsis sup),Condition	46253008	Thrombophlebitis of lower extremities
95	Infection (sepsis sup),Condition	48278001	Diphtheritic cystitis
95	Infection (sepsis sup),Condition	49736003	Periostosis without osteomyelitis
95	Infection (sepsis sup),Condition	50215002	Laryngeal diphtheria
95	Infection (sepsis sup),Condition	53453008	Osteomyelitis of shoulder region
95	Infection (sepsis sup),Condition	55083007	Periostitis of lower leg
95	Infection (sepsis sup),Condition	56905009	Perforation of intestine
95	Infection (sepsis sup),Condition	59867002	Acute osteomyelitis of multiple sites
95	Infection (sepsis sup),Condition	60168000	Osteomyelitis
95	Infection (sepsis sup),Condition	61112000	Pyogenic arthritis of hand
95	Infection (sepsis sup),Condition	61585002	Osteomyelitis of hand
95	Infection (sepsis sup),Condition	64156001	Thrombophlebitis
95	Infection (sepsis sup),Condition	65275009	Acute cholecystitis
95	Infection (sepsis sup),Condition	66634003	Chronic osteomyelitis of multiple sites
95	Infection (sepsis sup),Condition	66704002	Meningococcal pericarditis
95	Infection (sepsis sup),Condition	67685000	Prostatocystitis
95	Infection (sepsis sup),Condition	68566005	Urinary tract infectious disease
95	Infection (sepsis sup),Condition	69698001	Infection as complication of medical care

© 2021 Matheny ME et al. *JAMA Network Open.*

CONDITIONID	CONDITION_DESCRIPTION	SNOMED_CODE	DESCRIPTION
95	Infection (sepsis sup),Condition	70143003	Tuberculoid leprosy
95	Infection (sepsis sup),Condition	73431005	Meningococcal optic neuritis
95	Infection (sepsis sup),Condition	75286007	Osteomyelitis of upper arm
95	Infection (sepsis sup),Condition	75589004	Nasopharyngeal diphtheria
95	Infection (sepsis sup),Condition	76902006	Tetanus
95	Infection (sepsis sup),Condition	77116006	Infection due to Bordetella parapertussis
95	Infection (sepsis sup),Condition	79411002	Acute prostatitis
95	Infection (sepsis sup),Condition	81004002	Leprosy
95	Infection (sepsis sup),Condition	82670009	Whooping cough due to organism other than Bordetella pertussis
95	Infection (sepsis sup),Condition	85769006	Streptococcal infectious disease
95	Infection (sepsis sup),Condition	87073000	Periostitis of upper arm
95	Infection (sepsis sup),Condition	88415009	Mycobacteriosis
95	Infection (sepsis sup),Condition	91302008	Sepsis
95	Infection (sepsis sup),Condition	91538002	Osteomyelitis of multiple sites
95	Infection (sepsis sup),Condition	95450003	Thrombophlebitis of upper extremities
95	Infection (sepsis sup),Condition	95451004	Thrombophlebitis of superficial veins of upper extremities
95	Infection (sepsis sup),Condition	95452006	Thrombophlebitis of deep veins of upper extremities
95	Infection (sepsis sup),Condition	95881004	Mycetoma of foot
95	Infection (sepsis sup),Condition	111252006	Infection of bone associated with another disease

© 2021 Matheny ME et al. *JAMA Network Open.*

CONDITIONID	CONDITION_DESCRIPTION	SNOMED_CODE	DESCRIPTION
95	Infection (sepsis sup),Condition	111253001	Infection of bone
95	Infection (sepsis sup),Condition	192644005	Meningococcal meningitis
95	Infection (sepsis sup),Condition	194950003	Acute myocarditis - meningococcal
95	Infection (sepsis sup),Condition	195394007	Phlebitis and thrombophlebitis
95	Infection (sepsis sup),Condition	195410000	Thrombophlebitis of the femoral vein
95	Infection (sepsis sup),Condition	195951007	Acute exacerbation of chronic obstructive airways disease
95	Infection (sepsis sup),Condition	203153007	Acute osteomyelitis of lower leg
95	Infection (sepsis sup),Condition	203164005	Acute osteomyelitis of humerus
95	Infection (sepsis sup),Condition	203184009	Chronic osteomyelitis of shoulder
95	Infection (sepsis sup),Condition	203185005	Chronic osteomyelitis of upper arm
95	Infection (sepsis sup),Condition	203186006	Chronic osteomyelitis of forearm
95	Infection (sepsis sup),Condition	203189004	Chronic osteomyelitis of lower leg
95	Infection (sepsis sup),Condition	203224004	Infection of humerus
95	Infection (sepsis sup),Condition	203240001	Infection of multiple bones
95	Infection (sepsis sup),Condition	203245006	Periostitis without osteomyelitis
95	Infection (sepsis sup),Condition	203249000	Periostitis without osteomyelitis, of the forearm

© 2021 Matheny ME et al. *JAMA Network Open.*

CONDITIONID	CONDITION_DESCRIPTION	SNOMED_CODE	DESCRIPTION
95	Infection (sepsis sup),Condition	203250000	Periostitis without osteomyelitis, of the hand
95	Infection (sepsis sup),Condition	203251001	Periostitis without osteomyelitis, of the pelvic region and/or thigh
95	Infection (sepsis sup),Condition	203271005	Tuberculosis of the pelvic and/or thigh bones
95	Infection (sepsis sup),Condition	203284001	Osteopathy from poliomyelitis
95	Infection (sepsis sup),Condition	203286004	Poliomyelitis osteopathy of the shoulder region
95	Infection (sepsis sup),Condition	203287008	Poliomyelitis osteopathy of the upper arm
95	Infection (sepsis sup),Condition	203288003	Poliomyelitis osteopathy of the forearm
95	Infection (sepsis sup),Condition	203289006	Poliomyelitis osteopathy of the hand
95	Infection (sepsis sup),Condition	203290002	Poliomyelitis osteopathy of the pelvic region and/or thigh
95	Infection (sepsis sup),Condition	203291003	Poliomyelitis osteopathy of the lower leg
95	Infection (sepsis sup),Condition	203292005	Poliomyelitis osteopathy of the ankle and/or foot
95	Infection (sepsis sup),Condition	203294006	Poliomyelitis osteopathy of multiple sites
95	Infection (sepsis sup),Condition	239778009	Wrist pyogenic arthritis
95	Infection (sepsis sup),Condition	239779001	Elbow pyogenic arthritis

© 2021 Matheny ME et al. *JAMA Network Open.*

CONDITIONID	CONDITION_DESCRIPTION	SNOMED_CODE	DESCRIPTION
95	Infection (sepsis sup),Condition	240413000	Disseminated atypical mycobacterial infection
95	Infection (sepsis sup),Condition	266267005	Deep vein phlebitis and thrombophlebitis of the leg
95	Infection (sepsis sup),Condition	267880006	Pyogenic arthritis of the pelvic region and thigh
95	Infection (sepsis sup),Condition	267882003	Pyogenic arthritis of the ankle and/or foot
95	Infection (sepsis sup),Condition	268016009	Acute osteomyelitis of pelvic region and/or thigh
95	Infection (sepsis sup),Condition	268017000	Acute osteomyelitis of ankle and/or foot
95	Infection (sepsis sup),Condition	268018005	Chronic osteomyelitis of pelvic region and/or thigh
95	Infection (sepsis sup),Condition	268019002	Chronic osteomyelitis of ankle and/or foot
95	Infection (sepsis sup),Condition	276494008	Phlebitis and/or thrombophlebitis of iliac vein
95	Infection (sepsis sup),Condition	277869007	Non-tuberculous mycobacterial pneumonia
95	Infection (sepsis sup),Condition	312154004	Musculoskeletal infective disorder
95	Infection (sepsis sup),Condition	372939007	Suppurative arthritis
95	Infection (sepsis sup),Condition	397428000	Diphtheria
95	Infection (sepsis sup),Condition	400008009	Borderline leprosy

© 2021 Matheny ME et al. *JAMA Network Open.*

CONDITIONID	CONDITION_DESCRIPTION	SNOMED_CODE	DESCRIPTION
95	Infection (sepsis sup),Condition	408866006	Infection of central venous catheter
95	Infection (sepsis sup),Condition	409780002	Acute osteomyelitis
95	Infection (sepsis sup),Condition	441795000	Infected seroma after surgical procedure
95	Infection (sepsis sup),Condition	442449006	Infection of bone of ankle and/or foot
95	Infection (sepsis sup),Condition	444785006	Infection of bone of shoulder girdle
95	Infection (sepsis sup),Condition	444786007	Infection of bone of radius and/or ulna
95	Infection (sepsis sup),Condition	445012003	Infection of bone of hand
95	Infection (sepsis sup),Condition	445013008	Infection of bone of pelvic region and/or femur
95	Infection (sepsis sup),Condition	445215005	Infection of bone of tibia and/or fibula
95	Infection (sepsis sup),Condition	445378003	Acute exacerbation of bronchiectasis
95	Infection (sepsis sup),Condition	447685007	Sepsis due to Haemophilus influenzae
95	Infection (sepsis sup),Condition	447843005	Sepsis due to anaerobic bacteria
95	Infection (sepsis sup),Condition	447894003	Sepsis due to Staphylococcus
95	Infection (sepsis sup),Condition	447899008	Sepsis due to Escherichia coli
95	Infection (sepsis sup),Condition	448417001	Sepsis due to Staphylococcus aureus
95	Infection (sepsis sup),Condition	448418006	Sepsis due to Streptococcus

© 2021 Matheny ME et al. *JAMA Network Open.*

CONDITIONID	CONDITION_DESCRIPTION	SNOMED_CODE	DESCRIPTION
95	Infection (sepsis sup),Condition	448421008	Sepsis due to Streptococcus pneumoniae
95	Infection (sepsis sup),Condition	448812000	Sepsis due to methicillin resistant Staphylococcus aureus
95	Infection (sepsis sup),Condition	448813005	Sepsis due to Pseudomonas
95	Infection (sepsis sup),Condition	449082003	Sepsis due to Gram negative bacteria
95	Infection (sepsis sup),Condition	449084002	Sepsis due to Serratia
96	Organ Failure (sepsis sup),Condition	2776000	Delirium
96	Organ Failure (sepsis sup),Condition	5510009	Organic delusional disorder
96	Organ Failure (sepsis sup),Condition	14669001	Acute renal failure syndrome
96	Organ Failure (sepsis sup),Condition	17496003	Organic anxiety disorder
96	Organ Failure (sepsis sup),Condition	17890003	Hepatic infarction
96	Organ Failure (sepsis sup),Condition	27942005	Shock
96	Organ Failure (sepsis sup),Condition	28651003	Orthostatic hypotension
96	Organ Failure (sepsis sup),Condition	35455006	Acute tubular necrosis
96	Organ Failure (sepsis sup),Condition	45007003	Low blood pressure
96	Organ Failure (sepsis sup),Condition	45912004	Organic hallucinosis
96	Organ Failure (sepsis sup),Condition	46206005	Mood disorder
96	Organ Failure (sepsis sup),Condition	50122000	Metabolic encephalopathy
96	Organ Failure (sepsis sup),Condition	64779008	Blood coagulation disorder
96	Organ Failure (sepsis sup),Condition	67406007	Disseminated intravascular coagulation
96	Organ Failure (sepsis sup),Condition	73162004	Posttransfusion purpura

© 2021 Matheny ME et al. *JAMA Network Open.*

CONDITIONID	CONDITION_DESCRIPTION	SNOMED_CODE	DESCRIPTION
96	Organ Failure (sepsis sup),Condition	74576004	Acquired thrombocytopenia
96	Organ Failure (sepsis sup),Condition	76571007	Septic shock
96	Organ Failure (sepsis sup),Condition	77545000	Chronic hypotension
96	Organ Failure (sepsis sup),Condition	81308009	Disorder of brain
96	Organ Failure (sepsis sup),Condition	89138009	Cardiogenic shock
96	Organ Failure (sepsis sup),Condition	191507002	Subacute delirium
96	Organ Failure (sepsis sup),Condition	197268000	Acute and subacute liver necrosis
96	Organ Failure (sepsis sup),Condition	231442003	Organic catatonic disorder
96	Organ Failure (sepsis sup),Condition	234171009	Drug-induced hypotension
96	Organ Failure (sepsis sup),Condition	298015003	Acute renal papillary necrosis with renal failure
96	Organ Failure (sepsis sup),Condition	302215000	Thrombocytopenic disorder
96	Organ Failure (sepsis sup),Condition	389098007	Anoxic encephalopathy
96	Organ Failure (sepsis sup),Condition	408667000	Hemodialysis-associated hypotension
96	Organ Failure (sepsis sup),Condition	408668005	Iatrogenic hypotension
96	Organ Failure (sepsis sup),Condition	428703001	Transient organic mental disorder
96	Organ Failure (sepsis sup),Condition	429224003	Acute renal failure due to acute cortical necrosis
98	COPD, Condition	195949008	Chronic asthmatic bronchitis
98	COPD, Condition	442025000	Acute exacerbation of chronic asthmatic bronchitis
99	Acute Coronary Syndrome,Condition	4557003	Preinfarction syndrome

© 2021 Matheny ME et al. *JAMA Network Open.*

CONDITIONID	CONDITION_DESCRIPTION	SNOMED_CODE	DESCRIPTION
99	Acute Coronary Syndrome,Condition	54329005	Acute myocardial infarction of anterior wall
99	Acute Coronary Syndrome,Condition	57054005	Acute myocardial infarction
99	Acute Coronary Syndrome,Condition	58612006	Acute myocardial infarction of lateral wall
99	Acute Coronary Syndrome,Condition	63739005	Coronary occlusion
99	Acute Coronary Syndrome,Condition	65547006	Acute myocardial infarction of inferolateral wall
99	Acute Coronary Syndrome,Condition	66189004	Postmyocardial infarction syndrome
99	Acute Coronary Syndrome,Condition	70211005	Acute myocardial infarction of anterolateral wall
99	Acute Coronary Syndrome,Condition	70422006	Acute subendocardial infarction
99	Acute Coronary Syndrome,Condition	73795002	Acute myocardial infarction of inferior wall
99	Acute Coronary Syndrome,Condition	76593002	Acute myocardial infarction of inferoposterior wall
99	Acute Coronary Syndrome,Condition	194802003	True posterior myocardial infarction
99	Acute Coronary Syndrome,Condition	194809007	Acute myocardial infarction of atrium
99	Acute Coronary Syndrome,Condition	414545008	Ischemic heart disease
100	Pancreas Transplant,Procedure	416940007	Past history of procedure
101	Pregnancy,Condition	223003	Tumor of body of uterus affecting pregnancy

© 2021 Matheny ME et al. *JAMA Network Open.*

CONDITIONID	CONDITION_DESCRIPTION	SNOMED_CODE	DESCRIPTION
101	Pregnancy,Condition	1538006	Central nervous system malformation in fetus affecting obstetrical care
101	Pregnancy,Condition	1639007	Abnormality of organs AND/OR soft tissues of pelvis affecting pregnancy
101	Pregnancy,Condition	2781009	Miscarriage complicated by delayed and/or excessive hemorrhage
101	Pregnancy,Condition	2858002	Puerperal sepsis
101	Pregnancy,Condition	2989004	Complication following molar AND/OR ectopic pregnancy
101	Pregnancy,Condition	3033000	Bone AND/OR joint disorder of pelvis in mother complicating pregnancy, childbirth AND/OR puerperium
101	Pregnancy,Condition	3634007	Legal termination of pregnancy complicated by metabolic disorder
101	Pregnancy,Condition	4576001	Legal termination of pregnancy complicated by renal failure
101	Pregnancy,Condition	5626004	Varicose veins of legs complicating pregnancy AND/OR puerperium
101	Pregnancy,Condition	5740008	Pelvic hematoma during delivery

© 2021 Matheny ME et al. *JAMA Network Open.*

CONDITIONID	CONDITION_DESCRIPTION	SNOMED_CODE	DESCRIPTION
101	Pregnancy,Condition	5939002	Failed attempted termination of pregnancy complicated by metabolic disorder
101	Pregnancy,Condition	6096002	Breech presentation
101	Pregnancy,Condition	6234006	Second degree perineal laceration
101	Pregnancy,Condition	6594005	Cerebrovascular disorder in the puerperium
101	Pregnancy,Condition	6995000	Failure of lactation
101	Pregnancy,Condition	7792000	Placenta previa without hemorrhage
101	Pregnancy,Condition	7802000	Illegal termination of pregnancy without complication
101	Pregnancy,Condition	7822001	Failed attempted termination of pregnancy complicated by damage to pelvic organ and/or tissues
101	Pregnancy,Condition	7870007	Vascular lesion of cord
101	Pregnancy,Condition	8996006	Illegal termination of pregnancy complicated by renal failure
101	Pregnancy,Condition	9780006	Presentation of prolapsed arm of fetus
101	Pregnancy,Condition	9899009	Ovarian pregnancy
101	Pregnancy,Condition	10217006	Third degree perineal laceration
101	Pregnancy,Condition	10573002	Infection of amniotic cavity

© 2021 Matheny ME et al. *JAMA Network Open.*

CONDITIONID	CONDITION_DESCRIPTION	SNOMED_CODE	DESCRIPTION
101	Pregnancy,Condition	10697004	Miscarriage complicated by renal failure
101	Pregnancy,Condition	11209007	Cord entanglement without compression
101	Pregnancy,Condition	11914001	Transverse OR oblique presentation of fetus
101	Pregnancy,Condition	12729009	Prolonged rupture of membranes
101	Pregnancy,Condition	12867002	Fetal distress affecting management of mother
101	Pregnancy,Condition	13010001	Acute renal failure following labor AND/OR delivery
101	Pregnancy,Condition	13384007	Miscarriage complicated by metabolic disorder
101	Pregnancy,Condition	13943000	Failed attempted termination of pregnancy complicated by embolism
101	Pregnancy,Condition	14022007	Fetal death, affecting management of mother
101	Pregnancy,Condition	14094001	Hyperemesis gravidarum
101	Pregnancy,Condition	14918004	Disruption of perineal wound in the puerperium
101	Pregnancy,Condition	15230009	Liver disorder in pregnancy
101	Pregnancy,Condition	15400003	Obstetrical air embolism
101	Pregnancy,Condition	15938005	Eclampsia

© 2021 Matheny ME et al. *JAMA Network Open.*

CONDITIONID	CONDITION_DESCRIPTION	SNOMED_CODE	DESCRIPTION
101	Pregnancy,Condition	16083003	Damage to pelvic organs AND/OR tissues following molar AND/OR ectopic pregnancy
101	Pregnancy,Condition	16356006	Multiple pregnancy
101	Pregnancy,Condition	16607004	Missed miscarriage
101	Pregnancy,Condition	17263003	Amniotic fluid embolism
101	Pregnancy,Condition	17369002	Miscarriage
101	Pregnancy,Condition	17382005	Cervical incompetence
101	Pregnancy,Condition	17532001	Breech malpresentation successfully converted to cephalic presentation
101	Pregnancy,Condition	17787002	Peripheral neuritis in pregnancy
101	Pregnancy,Condition	18656007	Grand multipara
101	Pregnancy,Condition	18684002	Illegal termination of pregnancy complicated by metabolic disorder
101	Pregnancy,Condition	19569008	Mild hyperemesis gravidarum
101	Pregnancy,Condition	19773009	Infection of the breast AND/OR nipple associated with childbirth
101	Pregnancy,Condition	22173004	Excessive fetal growth affecting management of mother
101	Pregnancy,Condition	22399000	Puerperal endometritis
101	Pregnancy,Condition	22753004	Fetal AND/OR placental disorder affecting

© 2021 Matheny ME et al. *JAMA Network Open.*

CONDITIONID	CONDITION_DESCRIPTION	SNOMED_CODE	DESCRIPTION
			management of mother
101	Pregnancy,Condition	23171006	Delayed AND/OR secondary postpartum hemorrhage
101	Pregnancy,Condition	23332002	Failed trial of labor
101	Pregnancy,Condition	23793007	Miscarriage without complication
101	Pregnancy,Condition	24258008	Damage to pelvic joints AND/OR ligaments during delivery
101	Pregnancy,Condition	25053000	Obstetrical central nervous system complication of anesthesia AND/OR sedation
101	Pregnancy,Condition	25825004	Hemorrhage in early pregnancy
101	Pregnancy,Condition	26158002	Uterine inertia
101	Pregnancy,Condition	26224003	Failed attempted termination of pregnancy complicated by genital-pelvic infection
101	Pregnancy,Condition	26623000	Failed attempted termination of pregnancy complicated by delayed and/or excessive hemorrhage
101	Pregnancy,Condition	26743002	Illegal termination of pregnancy complicated by embolism
101	Pregnancy,Condition	26850007	Phlebitis AND/OR thrombosis

© 2021 Matheny ME et al. *JAMA Network Open.*

CONDITIONID	CONDITION_DESCRIPTION	SNOMED_CODE	DESCRIPTION
			complicating pregnancy AND/OR puerperium
101	Pregnancy,Condition	27075004	Venereal disease in mother complicating pregnancy, childbirth AND/OR puerperium
101	Pregnancy,Condition	27169005	Legal termination of pregnancy complicated by shock
101	Pregnancy,Condition	27214003	Atonic postpartum hemorrhage
101	Pregnancy,Condition	27215002	Uterine inversion
101	Pregnancy,Condition	27342004	Anemia of pregnancy
101	Pregnancy,Condition	29399001	Elderly primigravida
101	Pregnancy,Condition	29421008	Failed attempted termination of pregnancy complicated by renal failure
101	Pregnancy,Condition	29847008	Obstetrical complication of anesthesia AND/OR sedation
101	Pregnancy,Condition	30506002	Suppressed lactation
101	Pregnancy,Condition	30850008	Hemorrhage in early pregnancy, antepartum
101	Pregnancy,Condition	31407004	Pre-existing hypertension complicating AND/OR reason for care during puerperium

© 2021 Matheny ME et al. *JAMA Network Open.*

CONDITIONID	CONDITION_DESCRIPTION	SNOMED_CODE	DESCRIPTION
101	Pregnancy,Condition	31516002	Viral disease in mother complicating pregnancy, childbirth AND/OR puerperium
101	Pregnancy,Condition	31563000	Asymptomatic bacteriuria in pregnancy
101	Pregnancy,Condition	31805001	Fetal disproportion
101	Pregnancy,Condition	32999002	Complication of obstetrical surgical wound
101	Pregnancy,Condition	33627001	Prolonged first stage of labor
101	Pregnancy,Condition	34150001	Drug dependence in mother complicating pregnancy, childbirth AND/OR puerperium
101	Pregnancy,Condition	34242002	Syphilis in mother complicating pregnancy, childbirth AND/OR puerperium
101	Pregnancy,Condition	34270000	Miscarriage complicated by shock
101	Pregnancy,Condition	34500003	Legal termination of pregnancy without complication
101	Pregnancy,Condition	34614007	Miscarriage with complication
101	Pregnancy,Condition	34694006	Pre-existing hypertension complicating AND/OR reason for care during childbirth

© 2021 Matheny ME et al. *JAMA Network Open.*

CONDITIONID	CONDITION_DESCRIPTION	SNOMED_CODE	DESCRIPTION
101	Pregnancy,Condition	34801009	Ectopic pregnancy
101	Pregnancy,Condition	34842007	Antepartum hemorrhage
101	Pregnancy,Condition	35046003	Disorder of lactation
101	Pregnancy,Condition	35208003	Metabolic disorder following molar AND/OR ectopic pregnancy
101	Pregnancy,Condition	35347003	Delayed delivery after artificial rupture of membranes
101	Pregnancy,Condition	35882009	Abnormality of forces of labor
101	Pregnancy,Condition	35999006	Blighted ovum
101	Pregnancy,Condition	36854009	Inlet contraction of pelvis
101	Pregnancy,Condition	37141005	Maternal pyrexia during labor
101	Pregnancy,Condition	37762002	Face OR brow presentation of fetus
101	Pregnancy,Condition	38331001	Late vomiting of pregnancy
101	Pregnancy,Condition	38784004	Illegal termination of pregnancy complicated by damage to pelvic organs and/or tissues
101	Pregnancy,Condition	39406005	Legal termination of pregnancy
101	Pregnancy,Condition	39763004	Abnormal glucose tolerance in mother complicating pregnancy, childbirth AND/OR puerperium

© 2021 Matheny ME et al. *JAMA Network Open.*

CONDITIONID	CONDITION_DESCRIPTION	SNOMED_CODE	DESCRIPTION
101	Pregnancy,Condition	39804004	Abnormal products of conception
101	Pregnancy,Condition	40125005	Major puerperal infection
101	Pregnancy,Condition	40609001	Infectious disease in mother complicating pregnancy, childbirth AND/OR puerperium
101	Pregnancy,Condition	41114007	Mild pre-eclampsia
101	Pregnancy,Condition	41215002	Congenital abnormality of uterus, affecting pregnancy
101	Pregnancy,Condition	41337007	Conjoined twins
101	Pregnancy,Condition	42070007	Embolism following molar AND/OR ectopic pregnancy
101	Pregnancy,Condition	42686001	Chromosomal abnormality in fetus affecting obstetrical care
101	Pregnancy,Condition	43306002	Miscarriage complicated by embolism
101	Pregnancy,Condition	43629001	Renal failure following molar AND/OR ectopic pregnancy
101	Pregnancy,Condition	43715006	Secondary uterine inertia
101	Pregnancy,Condition	44223004	Premature rupture of membranes
101	Pregnancy,Condition	44772007	Maternal obesity syndrome
101	Pregnancy,Condition	44795003	Rhesus isoimmunization affecting pregnancy

© 2021 Matheny ME et al. *JAMA Network Open.*

CONDITIONID	CONDITION_DESCRIPTION	SNOMED_CODE	DESCRIPTION
101	Pregnancy,Condition	45828008	Anemia in mother complicating pregnancy, childbirth AND/OR puerperium
101	Pregnancy,Condition	46764007	Severe pre-eclampsia
101	Pregnancy,Condition	47236005	Third stage hemorrhage
101	Pregnancy,Condition	47236005	Third stage hemorrhage
101	Pregnancy,Condition	47821001	Postpartum hemorrhage
101	Pregnancy,Condition	48200009	Bone AND/OR joint disorder of lower extremities in mother complicating pregnancy, childbirth AND/OR puerperium
101	Pregnancy,Condition	48287005	Umbilical cord complication
101	Pregnancy,Condition	48433002	Legal termination of pregnancy with complication
101	Pregnancy,Condition	48782003	Delivery normal
101	Pregnancy,Condition	49416000	Failed attempted termination of pregnancy
101	Pregnancy,Condition	49550006	Premature pregnancy delivered
101	Pregnancy,Condition	49561003	Rupture of uterus before onset of labor
101	Pregnancy,Condition	49632008	Illegal termination of pregnancy
101	Pregnancy,Condition	49956009	Antepartum deep vein thrombosis

© 2021 Matheny ME et al. *JAMA Network Open.*

CONDITIONID	CONDITION_DESCRIPTION	SNOMED_CODE	DESCRIPTION
101	Pregnancy,Condition	51154004	Obstetrical pulmonary complication of anesthesia AND/OR sedation
101	Pregnancy,Condition	51195001	Placental polyp
101	Pregnancy,Condition	51920004	Precipitate labor
101	Pregnancy,Condition	53443007	Prolonged labor
101	Pregnancy,Condition	54048003	Threatened miscarriage
101	Pregnancy,Condition	55613002	Engorgement of breasts associated with childbirth
101	Pregnancy,Condition	56272000	Postpartum deep phlebothrombosis
101	Pregnancy,Condition	57734001	Legal termination of pregnancy complicated by embolism
101	Pregnancy,Condition	57759005	First degree perineal laceration
101	Pregnancy,Condition	58071009	Legal termination of pregnancy complicated by delayed and/or excessive hemorrhage
101	Pregnancy,Condition	58990004	Miscarriage complicated by damage to pelvic organs and/or tissues
101	Pregnancy,Condition	59566000	Oligohydramnios
101	Pregnancy,Condition	59795007	Short cord
101	Pregnancy,Condition	59919008	Failed attempted termination of pregnancy with complication
101	Pregnancy,Condition	60810003	Quadruplet pregnancy

© 2021 Matheny ME et al. *JAMA Network Open.*

CONDITIONID	CONDITION_DESCRIPTION	SNOMED_CODE	DESCRIPTION
101	Pregnancy,Condition	61752008	Illegal termination of pregnancy complicated by shock
101	Pregnancy,Condition	62377009	Postpartum cardiomyopathy
101	Pregnancy,Condition	64171002	Obstetrical cardiac complication of anesthesia AND/OR sedation
101	Pregnancy,Condition	64229006	Traumatic lesion during delivery
101	Pregnancy,Condition	64254006	Triplet pregnancy
101	Pregnancy,Condition	64646001	Failed forceps delivery
101	Pregnancy,Condition	64779008	Blood coagulation disorder
101	Pregnancy,Condition	64779008	Blood coagulation disorder
101	Pregnancy,Condition	65147003	Twin pregnancy
101	Pregnancy,Condition	65402008	Pre-existing hypertension complicating AND/OR reason for care during pregnancy
101	Pregnancy,Condition	65727000	Intrauterine pregnancy
101	Pregnancy,Condition	66844003	Generalized infection during labor
101	Pregnancy,Condition	66958002	Isoimmunization from non-ABO, non-Rh blood-group incompatibility affecting pregnancy
101	Pregnancy,Condition	67042008	Failed attempted termination of pregnancy

© 2021 Matheny ME et al. *JAMA Network Open.*

CONDITIONID	CONDITION_DESCRIPTION	SNOMED_CODE	DESCRIPTION
			complicated by shock
101	Pregnancy,Condition	67480003	Indication for care AND/OR intervention in labor AND/OR delivery
101	Pregnancy,Condition	69217004	Outlet contraction of pelvis
101	Pregnancy,Condition	69270005	Rupture of uterus during AND/OR after labor
101	Pregnancy,Condition	69344006	Shock following molar AND/OR ectopic pregnancy
101	Pregnancy,Condition	69777007	Interlocked twins
101	Pregnancy,Condition	70068004	Persistent occipitoposterior position
101	Pregnancy,Condition	70591005	Fetal disorder
101	Pregnancy,Condition	71028008	Fetal-maternal hemorrhage
101	Pregnancy,Condition	71639005	Galactorrhea associated with childbirth
101	Pregnancy,Condition	71901000	Congenital contracted pelvis
101	Pregnancy,Condition	72860003	Disorder of amniotic cavity AND/OR membrane
101	Pregnancy,Condition	74978008	Illegal termination of pregnancy with complication
101	Pregnancy,Condition	75150001	Renal disease in pregnancy AND/OR puerperium without hypertension
101	Pregnancy,Condition	76751001	Diabetes mellitus in mother

© 2021 Matheny ME et al. *JAMA Network Open.*

CONDITIONID	CONDITION_DESCRIPTION	SNOMED_CODE	DESCRIPTION
			complicating pregnancy, childbirth AND/OR puerperium
101	Pregnancy,Condition	77206006	Puerperal pelvic sepsis
101	Pregnancy,Condition	77259008	Prolonged second stage of labor
101	Pregnancy,Condition	77278008	Velamentous insertion of umbilical cord
101	Pregnancy,Condition	77386006	Pregnant
101	Pregnancy,Condition	77854008	Failed medical induction of labor
101	Pregnancy,Condition	78381004	Heart disease in mother complicating pregnancy, childbirth AND/OR puerperium
101	Pregnancy,Condition	78408007	Complication of obstetrical surgery AND/OR procedure
101	Pregnancy,Condition	78697003	Nonpurulent mastitis associated with childbirth
101	Pregnancy,Condition	79133008	Delayed AND/OR excessive hemorrhage following molar AND/OR ectopic pregnancy
101	Pregnancy,Condition	79586000	Tubal pregnancy
101	Pregnancy,Condition	79668009	Vasa previa
101	Pregnancy,Condition	80002007	Malpresentation of fetus
101	Pregnancy,Condition	80113008	Complication of the puerperium
101	Pregnancy,Condition	80224003	Multiple gestation with one OR more

© 2021 Matheny ME et al. *JAMA Network Open.*

CONDITIONID	CONDITION_DESCRIPTION	SNOMED_CODE	DESCRIPTION
			fetal malpresentations
101	Pregnancy,Condition	80256005	Intervillous thrombosis
101	Pregnancy,Condition	81448000	Hemorrhage in early pregnancy, delivered
101	Pregnancy,Condition	82661006	Abdominal pregnancy
101	Pregnancy,Condition	84007008	Shock during AND/OR following labor AND/OR delivery
101	Pregnancy,Condition	84457005	Spontaneous onset of labor
101	Pregnancy,Condition	84757009	Epilepsy
101	Pregnancy,Condition	84939004	Rubella in mother complicating pregnancy, childbirth AND/OR puerperium
101	Pregnancy,Condition	86196005	Disorder of breast associated with childbirth
101	Pregnancy,Condition	86203003	Polyhydramnios
101	Pregnancy,Condition	86356004	Unstable lie
101	Pregnancy,Condition	86891002	Illegal termination of pregnancy complicated by delayed and/or excessive hemorrhage
101	Pregnancy,Condition	87383005	Maternal distress
101	Pregnancy,Condition	88887003	Maternal hypotension syndrome
101	Pregnancy,Condition	89700002	Shoulder girdle dystocia
101	Pregnancy,Condition	90127001	Fetus papyraceous
101	Pregnancy,Condition	90188009	Failed mechanical induction

© 2021 Matheny ME et al. *JAMA Network Open.*

CONDITIONID	CONDITION_DESCRIPTION	SNOMED_CODE	DESCRIPTION
101	Pregnancy,Condition	90325002	Vomiting of pregnancy
101	Pregnancy,Condition	90645002	Failed attempted termination of pregnancy without complication
101	Pregnancy,Condition	90968009	Post-term pregnancy
101	Pregnancy,Condition	102878001	Recurrent miscarriage
101	Pregnancy,Condition	106005003	Hypertension AND/OR vomiting complicating pregnancy childbirth AND/OR puerperium
101	Pregnancy,Condition	106007006	Maternal AND/OR fetal condition affecting labor AND/OR delivery
101	Pregnancy,Condition	106009009	Fetal condition affecting obstetrical care of mother
101	Pregnancy,Condition	111425004	Genital tract AND/OR pelvic infection following molar AND/OR ectopic pregnancy
101	Pregnancy,Condition	111431001	Legal termination of pregnancy complicated by damage to pelvic organ and/or tissues
101	Pregnancy,Condition	111443000	Congenital OR acquired abnormality of cervix affecting pregnancy

© 2021 Matheny ME et al. *JAMA Network Open.*

CONDITIONID	CONDITION_DESCRIPTION	SNOMED_CODE	DESCRIPTION
101	Pregnancy,Condition	111444006	Congenital OR acquired abnormality of vagina affecting pregnancy
101	Pregnancy,Condition	111445007	Congenital OR acquired abnormality of vulva affecting pregnancy
101	Pregnancy,Condition	111447004	Placental condition affecting management of mother
101	Pregnancy,Condition	111451002	Obstetrical injury to pelvic organ
101	Pregnancy,Condition	111453004	Retained placenta, without hemorrhage
101	Pregnancy,Condition	111454005	Retained portions of placenta AND/OR membranes without hemorrhage
101	Pregnancy,Condition	111459000	Infection of nipple, associated with childbirth
101	Pregnancy,Condition	118864003	Procedure on uterus
101	Pregnancy,Condition	124736009	Compound presentation
101	Pregnancy,Condition	127364007	Primigravida
101	Pregnancy,Condition	128076000	Bone AND/OR joint disorder in mother complicating pregnancy, childbirth AND/OR puerperium
101	Pregnancy,Condition	133874006	Selective fetal reduction

© 2021 Matheny ME et al. *JAMA Network Open.*

CONDITIONID	CONDITION_DESCRIPTION	SNOMED_CODE	DESCRIPTION
101	Pregnancy,Condition	134435003	Routine antenatal care
101	Pregnancy,Condition	156072005	Incomplete miscarriage
101	Pregnancy,Condition	156073000	Complete miscarriage
101	Pregnancy,Condition	169940006	Maternal tobacco abuse
101	Pregnancy,Condition	198609003	Complication of pregnancy, childbirth and/or the puerperium
101	Pregnancy,Condition	198609003	Complication of pregnancy, childbirth and/or the puerperium
101	Pregnancy,Condition	198644001	Incomplete miscarriage with genital tract or pelvic infection
101	Pregnancy,Condition	198645000	Incomplete miscarriage with delayed or excessive hemorrhage
101	Pregnancy,Condition	198646004	Incomplete miscarriage with damage to pelvic organs or tissues
101	Pregnancy,Condition	198647008	Incomplete miscarriage with renal failure
101	Pregnancy,Condition	198648003	Incomplete miscarriage with metabolic disorder
101	Pregnancy,Condition	198649006	Incomplete miscarriage with shock
101	Pregnancy,Condition	198650006	Incomplete miscarriage with embolism

© 2021 Matheny ME et al. *JAMA Network Open.*

CONDITIONID	CONDITION_DESCRIPTION	SNOMED_CODE	DESCRIPTION
101	Pregnancy,Condition	198655001	Complete miscarriage with genital tract or pelvic infection
101	Pregnancy,Condition	198656000	Complete miscarriage with delayed or excessive hemorrhage
101	Pregnancy,Condition	198657009	Complete miscarriage with damage to pelvic organs or tissues
101	Pregnancy,Condition	198659007	Complete miscarriage with renal failure
101	Pregnancy,Condition	198660002	Complete miscarriage with metabolic disorder
101	Pregnancy,Condition	198661003	Complete miscarriage with shock
101	Pregnancy,Condition	198663000	Complete miscarriage with embolism
101	Pregnancy,Condition	198705001	Incomplete legal termination of pregnancy with genital tract or pelvic infection
101	Pregnancy,Condition	198706000	Incomplete legal termination of pregnancy with delayed or excessive hemorrhage
101	Pregnancy,Condition	198707009	Incomplete legal termination of pregnancy with damage to pelvic organs or tissues

© 2021 Matheny ME et al. *JAMA Network Open.*

CONDITIONID	CONDITION_DESCRIPTION	SNOMED_CODE	DESCRIPTION
101	Pregnancy,Condition	198708004	Incomplete legal termination of pregnancy with renal failure
101	Pregnancy,Condition	198709007	Incomplete legal termination of pregnancy with metabolic disorder
101	Pregnancy,Condition	198710002	Incomplete legal termination of pregnancy with shock
101	Pregnancy,Condition	198711003	Incomplete legal termination of pregnancy with embolism
101	Pregnancy,Condition	198718009	Complete legal termination of pregnancy with genital tract or pelvic infection
101	Pregnancy,Condition	198719001	Complete legal termination of pregnancy with delayed or excessive hemorrhage
101	Pregnancy,Condition	198720007	Complete legal termination of pregnancy with damage to pelvic organs or tissues
101	Pregnancy,Condition	198721006	Complete legal termination of pregnancy with renal failure
101	Pregnancy,Condition	198722004	Complete legal termination of pregnancy with metabolic disorder

© 2021 Matheny ME et al. *JAMA Network Open.*

CONDITIONID	CONDITION_DESCRIPTION	SNOMED_CODE	DESCRIPTION
101	Pregnancy,Condition	198723009	Complete legal termination of pregnancy with shock
101	Pregnancy,Condition	198724003	Complete legal termination of pregnancy with embolism
101	Pregnancy,Condition	198744009	Incomplete illegal termination of pregnancy with genital tract or pelvic infection
101	Pregnancy,Condition	198745005	Incomplete illegal termination of pregnancy with delayed or excessive hemorrhage
101	Pregnancy,Condition	198746006	Incomplete illegal termination of pregnancy with damage to pelvic organs or tissues
101	Pregnancy,Condition	198747002	Incomplete illegal termination of pregnancy with renal failure
101	Pregnancy,Condition	198748007	Incomplete illegal termination of pregnancy with metabolic disorder
101	Pregnancy,Condition	198749004	Incomplete illegal termination of pregnancy with shock
101	Pregnancy,Condition	198750004	Incomplete illegal termination of pregnancy with embolism

© 2021 Matheny ME et al. *JAMA Network Open.*

CONDITIONID	CONDITION_DESCRIPTION	SNOMED_CODE	DESCRIPTION
101	Pregnancy,Condition	198755009	Illegal termination of pregnancy, complete
101	Pregnancy,Condition	198756005	Complete illegal termination of pregnancy with genital tract or pelvic infection
101	Pregnancy,Condition	198757001	Complete illegal termination of pregnancy with delayed or excessive hemorrhage
101	Pregnancy,Condition	198758006	Complete illegal termination of pregnancy with damage to pelvic organs or tissues
101	Pregnancy,Condition	198759003	Complete illegal termination of pregnancy with renal failure
101	Pregnancy,Condition	198760008	Complete illegal termination of pregnancy with metabolic disorder
101	Pregnancy,Condition	198761007	Complete illegal termination of pregnancy with shock
101	Pregnancy,Condition	198762000	Complete illegal termination of pregnancy with embolism
101	Pregnancy,Condition	198899007	Placenta previa without hemorrhage - delivered
101	Pregnancy,Condition	198900002	Placenta previa without

© 2021 Matheny ME et al. *JAMA Network Open.*

CONDITIONID	CONDITION_DESCRIPTION	SNOMED_CODE	DESCRIPTION
			hemorrhage - not delivered
101	Pregnancy,Condition	198903000	Placenta previa with hemorrhage
101	Pregnancy,Condition	198905007	Placenta previa with hemorrhage - delivered
101	Pregnancy,Condition	198906008	Placenta previa with hemorrhage - not delivered
101	Pregnancy,Condition	198910006	Placental abruption - delivered
101	Pregnancy,Condition	198911005	Placental abruption - not delivered
101	Pregnancy,Condition	198917009	Antepartum hemorrhage with coagulation defect - delivered
101	Pregnancy,Condition	198941007	Hypertension complicating pregnancy, childbirth and the puerperium
101	Pregnancy,Condition	198942000	Benign essential hypertension complicating pregnancy, childbirth and the puerperium
101	Pregnancy,Condition	198944004	Benign essential hypertension complicating pregnancy, childbirth and the puerperium - delivered
101	Pregnancy,Condition	198945003	Benign essential hypertension complicating pregnancy, childbirth and the

© 2021 Matheny ME et al. *JAMA Network Open.*

CONDITIONID	CONDITION_DESCRIPTION	SNOMED_CODE	DESCRIPTION
			puerperium - delivered with postnatal complication
101	Pregnancy,Condition	198946002	Benign essential hypertension complicating pregnancy, childbirth and the puerperium - not delivered
101	Pregnancy,Condition	198947006	Benign essential hypertension complicating pregnancy, childbirth and the puerperium with postnatal complication
101	Pregnancy,Condition	198949009	Renal hypertension complicating pregnancy, childbirth and the puerperium
101	Pregnancy,Condition	198951008	Renal hypertension complicating pregnancy, childbirth and the puerperium - delivered
101	Pregnancy,Condition	198952001	Renal hypertension complicating pregnancy, childbirth and the puerperium - delivered with postnatal complication
101	Pregnancy,Condition	198953006	Renal hypertension complicating pregnancy,

© 2021 Matheny ME et al. *JAMA Network Open.*

CONDITIONID	CONDITION_DESCRIPTION	SNOMED_CODE	DESCRIPTION
			childbirth and the puerperium - not delivered
101	Pregnancy,Condition	198954000	Renal hypertension complicating pregnancy, childbirth and the puerperium with postnatal complication
101	Pregnancy,Condition	198965005	Transient hypertension of pregnancy - delivered
101	Pregnancy,Condition	198966006	Transient hypertension of pregnancy - delivered with postnatal complication
101	Pregnancy,Condition	198967002	Transient hypertension of pregnancy - not delivered
101	Pregnancy,Condition	198983002	Severe pre-eclampsia - delivered
101	Pregnancy,Condition	198984008	Severe pre-eclampsia - delivered with postnatal complication
101	Pregnancy,Condition	198985009	Severe pre-eclampsia - not delivered
101	Pregnancy,Condition	198986005	Severe pre-eclampsia with postnatal complication
101	Pregnancy,Condition	198990007	Eclampsia - delivered

© 2021 Matheny ME et al. *JAMA Network Open.*

CONDITIONID	CONDITION_DESCRIPTION	SNOMED_CODE	DESCRIPTION
101	Pregnancy,Condition	198991006	Eclampsia - delivered with postnatal complication
101	Pregnancy,Condition	198992004	Eclampsia in pregnancy
101	Pregnancy,Condition	198997005	Pre-eclampsia or eclampsia with pre-existing hypertension
101	Pregnancy,Condition	198999008	Pre-eclampsia or eclampsia with pre-existing hypertension - delivered
101	Pregnancy,Condition	199000005	Pre-eclampsia or eclampsia with pre-existing hypertension - delivered with postnatal complication
101	Pregnancy,Condition	199002002	Pre-eclampsia or eclampsia with pre-existing hypertension - not delivered
101	Pregnancy,Condition	199005000	Pre-existing hypertension complicating pregnancy, childbirth and puerperium
101	Pregnancy,Condition	199022003	Mild hyperemesis-delivered
101	Pregnancy,Condition	199023008	Mild hyperemesis-not delivered
101	Pregnancy,Condition	199025001	Hyperemesis gravidarum with metabolic disturbance

© 2021 Matheny ME et al. *JAMA Network Open.*

CONDITIONID	CONDITION_DESCRIPTION	SNOMED_CODE	DESCRIPTION
101	Pregnancy,Condition	199027009	Hyperemesis gravidarum with metabolic disturbance - delivered
101	Pregnancy,Condition	199028004	Hyperemesis gravidarum with metabolic disturbance - not delivered
101	Pregnancy,Condition	199032005	Late pregnancy vomiting - delivered
101	Pregnancy,Condition	199033000	Late pregnancy vomiting - not delivered
101	Pregnancy,Condition	199049003	Threatened premature labor - not delivered
101	Pregnancy,Condition	199063009	Post-term pregnancy - delivered
101	Pregnancy,Condition	199069008	Papyraceous fetus - delivered
101	Pregnancy,Condition	199070009	Papyraceous fetus - not delivered
101	Pregnancy,Condition	199087006	History of recurrent miscarriage - delivered
101	Pregnancy,Condition	199088001	History of recurrent miscarriage - not delivered
101	Pregnancy,Condition	199093003	Peripheral neuritis in pregnancy - delivered
101	Pregnancy,Condition	199099004	Asymptomatic bacteriuria in pregnancy - delivered

© 2021 Matheny ME et al. *JAMA Network Open.*

CONDITIONID	CONDITION_DESCRIPTION	SNOMED_CODE	DESCRIPTION
101	Pregnancy,Condition	199100007	Asymptomatic bacteriuria in pregnancy - delivered with postnatal complication
101	Pregnancy,Condition	199102004	Asymptomatic bacteriuria in pregnancy with postnatal complication
101	Pregnancy,Condition	199106001	Genitourinary tract infection in pregnancy - delivered
101	Pregnancy,Condition	199107005	Genitourinary tract infection in pregnancy - delivered with postnatal complication
101	Pregnancy,Condition	199108000	Genitourinary tract infection in pregnancy - not delivered
101	Pregnancy,Condition	199117000	Liver disorder in pregnancy - delivered
101	Pregnancy,Condition	199118005	Liver disorder in pregnancy - not delivered
101	Pregnancy,Condition	199153003	Infective/parasitic disease in preg/childbirth/puerperium
101	Pregnancy,Condition	199156006	Maternal syphilis during pregnancy - baby delivered
101	Pregnancy,Condition	199157002	Maternal syphilis in the puerperium - baby delivered

© 2021 Matheny ME et al. *JAMA Network Open.*

CONDITIONID	CONDITION_DESCRIPTION	SNOMED_CODE	DESCRIPTION
			during current episode of care
101	Pregnancy,Condition	199158007	Maternal syphilis during pregnancy - baby not yet delivered
101	Pregnancy,Condition	199161008	Maternal gonorrhea during pregnancy, childbirth and the puerperium
101	Pregnancy,Condition	199163006	Maternal gonorrhea during pregnancy - baby delivered
101	Pregnancy,Condition	199175001	Maternal tuberculosis during pregnancy, childbirth and the puerperium
101	Pregnancy,Condition	199178004	Tuberculosis in pregnancy, childbirth and the puerperium - delivered
101	Pregnancy,Condition	199180005	Maternal tuberculosis during pregnancy - baby not yet delivered
101	Pregnancy,Condition	199181009	Maternal tuberculosis in the puerperium - baby delivered during previous episode of care
101	Pregnancy,Condition	199183007	Maternal malaria during pregnancy, childbirth and the puerperium

© 2021 Matheny ME et al. *JAMA Network Open.*

CONDITIONID	CONDITION_DESCRIPTION	SNOMED_CODE	DESCRIPTION
101	Pregnancy,Condition	199185000	Maternal malaria during pregnancy - baby delivered
101	Pregnancy,Condition	199187008	Maternal malaria during pregnancy - baby not yet delivered
101	Pregnancy,Condition	199188003	Maternal malaria in the puerperium - baby delivered during previous episode of care
101	Pregnancy,Condition	199192005	Maternal rubella during pregnancy - baby delivered
101	Pregnancy,Condition	199193000	Maternal rubella in the puerperium - baby delivered during current episode of care
101	Pregnancy,Condition	199195007	Maternal rubella in the puerperium - baby delivered during previous episode of care
101	Pregnancy,Condition	199225007	Diabetes mellitus during pregnancy - baby delivered
101	Pregnancy,Condition	199226008	Diabetes mellitus in the puerperium - baby delivered during current episode of care
101	Pregnancy,Condition	199227004	Diabetes mellitus during pregnancy - baby not yet delivered
101	Pregnancy,Condition	199228009	Diabetes mellitus in the puerperium - baby delivered

© 2021 Matheny ME et al. *JAMA Network Open.*

CONDITIONID	CONDITION_DESCRIPTION	SNOMED_CODE	DESCRIPTION
			during previous episode of care
101	Pregnancy,Condition	199235001	Thyroid dysfunction during pregnancy, childbirth and the puerperium
101	Pregnancy,Condition	199237009	Thyroid dysfunction during pregnancy - baby delivered
101	Pregnancy,Condition	199238004	Thyroid dysfunction in the puerperium - baby delivered during current episode of care
101	Pregnancy,Condition	199239007	Thyroid dysfunction during pregnancy - baby not yet delivered
101	Pregnancy,Condition	199240009	Thyroid dysfunction in the puerperium - baby delivered during previous episode of care
101	Pregnancy,Condition	199244000	Anemia during pregnancy - baby delivered
101	Pregnancy,Condition	199246003	Anemia during pregnancy - baby not yet delivered
101	Pregnancy,Condition	199252002	Drug dependence during pregnancy - baby delivered
101	Pregnancy,Condition	199253007	Drug dependence in the puerperium - baby delivered
101	Pregnancy,Condition	199257008	Mental disorders during pregnancy,

© 2021 Matheny ME et al. *JAMA Network Open.*

CONDITIONID	CONDITION_DESCRIPTION	SNOMED_CODE	DESCRIPTION
			childbirth and the puerperium
101	Pregnancy,Condition	199259006	Mental disorder during pregnancy - baby delivered
101	Pregnancy,Condition	199260001	Mental disorder in the puerperium - baby delivered
101	Pregnancy,Condition	199266007	Congenital cardiovascular disorder during pregnancy - baby delivered
101	Pregnancy,Condition	199268008	Congenital cardiovascular disorder during pregnancy - baby not yet delivered
101	Pregnancy,Condition	199307003	Continuing pregnancy after intrauterine death of one or more fetuses
101	Pregnancy,Condition	199317008	Twin pregnancy - delivered
101	Pregnancy,Condition	199318003	Twin pregnancy with antenatal problem
101	Pregnancy,Condition	199321001	Triplet pregnancy - delivered
101	Pregnancy,Condition	199322008	Triplet pregnancy with antenatal problem
101	Pregnancy,Condition	199325005	Quadruplet pregnancy - delivered
101	Pregnancy,Condition	199326006	Quadruplet pregnancy with antenatal problem
101	Pregnancy,Condition	199344003	Unstable lie - delivered

© 2021 Matheny ME et al. *JAMA Network Open.*

CONDITIONID	CONDITION_DESCRIPTION	SNOMED_CODE	DESCRIPTION
101	Pregnancy,Condition	199345002	Unstable lie with antenatal problem
101	Pregnancy,Condition	199354004	Breech presentation - delivered
101	Pregnancy,Condition	199355003	Breech presentation with antenatal problem
101	Pregnancy,Condition	199362007	Transverse lie - delivered
101	Pregnancy,Condition	199373000	High head at term
101	Pregnancy,Condition	199375007	High head at term - delivered
101	Pregnancy,Condition	199376008	High head at term with antenatal problem
101	Pregnancy,Condition	199380003	Multiple pregnancy with malpresentation - delivered
101	Pregnancy,Condition	199381004	Multiple pregnancy with malpresentation with antenatal problem
101	Pregnancy,Condition	199384007	Prolapsed arm - delivered
101	Pregnancy,Condition	199385008	Prolapsed arm with antenatal problem
101	Pregnancy,Condition	199398004	Disproportion - major pelvic abnormality
101	Pregnancy,Condition	199400000	Disproportion - major pelvic abnormality - delivered
101	Pregnancy,Condition	199402008	Disproportion - major pelvic abnormality with antenatal problem

© 2021 Matheny ME et al. *JAMA Network Open.*

CONDITIONID	CONDITION_DESCRIPTION	SNOMED_CODE	DESCRIPTION
101	Pregnancy,Condition	199405005	Generally contracted pelvis - delivered
101	Pregnancy,Condition	199406006	Generally contracted pelvis with antenatal problem
101	Pregnancy,Condition	199409004	Inlet pelvic contraction - delivered
101	Pregnancy,Condition	199410009	Inlet pelvic contraction with antenatal problem
101	Pregnancy,Condition	199413006	Outlet pelvic contraction - delivered
101	Pregnancy,Condition	199414000	Outlet pelvic contraction with antenatal problem
101	Pregnancy,Condition	199416003	Mixed feto-pelvic disproportion
101	Pregnancy,Condition	199418002	Mixed feto-pelvic disproportion - delivered
101	Pregnancy,Condition	199419005	Mixed feto-pelvic disproportion with antenatal problem
101	Pregnancy,Condition	199422007	Large fetus causing disproportion - delivered
101	Pregnancy,Condition	199423002	Large fetus causing disproportion with antenatal problem
101	Pregnancy,Condition	199425009	Hydrocephalic disproportion
101	Pregnancy,Condition	199427001	Hydrocephalic disproportion - delivered
101	Pregnancy,Condition	199428006	Hydrocephalic disproportion with antenatal problem

© 2021 Matheny ME et al. *JAMA Network Open.*

CONDITIONID	CONDITION_DESCRIPTION	SNOMED_CODE	DESCRIPTION
101	Pregnancy,Condition	199446000	Pelvic soft tissue abnormality in pregnancy, childbirth and the puerperium
101	Pregnancy,Condition	199461004	Uterine scar from previous surgery in pregnancy, childbirth and the puerperium
101	Pregnancy,Condition	199463001	Uterine scar from previous surgery in pregnancy, childbirth and the puerperium - delivered
101	Pregnancy,Condition	199464007	Uterine scar from previous surgery in pregnancy, childbirth and the puerperium with antenatal problem
101	Pregnancy,Condition	199466009	Retroverted incarcerated gravid uterus
101	Pregnancy,Condition	199468005	Retroverted incarcerated gravid uterus - delivered
101	Pregnancy,Condition	199469002	Retroverted incarcerated gravid uterus - delivered with postnatal complication
101	Pregnancy,Condition	199470001	Retroverted incarcerated gravid uterus with antenatal problem
101	Pregnancy,Condition	199482005	Cervical incompetence - delivered

© 2021 Matheny ME et al. *JAMA Network Open.*

CONDITIONID	CONDITION_DESCRIPTION	SNOMED_CODE	DESCRIPTION
101	Pregnancy,Condition	199483000	Cervical incompetence - delivered with postnatal complication
101	Pregnancy,Condition	199484006	Cervical incompetence with antenatal problem
101	Pregnancy,Condition	199485007	Cervical incompetence with postnatal complication
101	Pregnancy,Condition	199512003	Pelvic soft tissue abnormality in pregnancy, childbirth and the puerperium - delivered with postnatal complication
101	Pregnancy,Condition	199513008	Pelvic soft tissue abnormality in pregnancy, childbirth and the puerperium with antenatal problem
101	Pregnancy,Condition	199526007	Fetus with chromosomal abnormality - delivered
101	Pregnancy,Condition	199527003	Fetus with chromosomal abnormality with antenatal problem
101	Pregnancy,Condition	199531009	Fetus with hereditary disease
101	Pregnancy,Condition	199533007	Fetus with hereditary disease - delivered
101	Pregnancy,Condition	199534001	Fetus with hereditary disease

© 2021 Matheny ME et al. *JAMA Network Open.*

CONDITIONID	CONDITION_DESCRIPTION	SNOMED_CODE	DESCRIPTION
			with antenatal problem
101	Pregnancy,Condition	199538003	Fetus with viral damage via mother - delivered
101	Pregnancy,Condition	199539006	Fetus with viral damage via mother with antenatal problem
101	Pregnancy,Condition	199547006	Fetus with drug damage
101	Pregnancy,Condition	199549009	Fetus with drug damage - delivered
101	Pregnancy,Condition	199550009	Fetus with drug damage with antenatal problem
101	Pregnancy,Condition	199553006	Fetus with radiation damage
101	Pregnancy,Condition	199555004	Fetus with radiation damage - delivered
101	Pregnancy,Condition	199556003	Fetus with radiation damage with antenatal problem
101	Pregnancy,Condition	199558002	Fetus with damage due to intrauterine contraceptive device
101	Pregnancy,Condition	199577000	Fetal-maternal hemorrhage - delivered
101	Pregnancy,Condition	199578005	Fetal-maternal hemorrhage with antenatal problem
101	Pregnancy,Condition	199582007	Rhesus isoimmunization - delivered
101	Pregnancy,Condition	199583002	Rhesus isoimmunization

© 2021 Matheny ME et al. *JAMA Network Open.*

CONDITIONID	CONDITION_DESCRIPTION	SNOMED_CODE	DESCRIPTION
			with antenatal problem
101	Pregnancy,Condition	199607009	Intrauterine death - delivered
101	Pregnancy,Condition	199608004	Intrauterine death with antenatal problem
101	Pregnancy,Condition	199646006	Polyhydramnios - delivered
101	Pregnancy,Condition	199647002	Polyhydramnios with antenatal problem
101	Pregnancy,Condition	199653002	Oligohydramnios - delivered
101	Pregnancy,Condition	199654008	Oligohydramnios with antenatal problem
101	Pregnancy,Condition	199658006	Premature rupture of membranes - delivered
101	Pregnancy,Condition	199659003	Premature rupture of membranes with antenatal problem
101	Pregnancy,Condition	199677008	Amniotic cavity infection - delivered
101	Pregnancy,Condition	199678003	Amniotic cavity infection with antenatal problem
101	Pregnancy,Condition	199694005	Failed mechanical induction - delivered
101	Pregnancy,Condition	199695006	Failed mechanical induction with antenatal problem
101	Pregnancy,Condition	199714004	Grand multiparity - delivered
101	Pregnancy,Condition	199715003	Grand multiparity with antenatal problem

© 2021 Matheny ME et al. *JAMA Network Open.*

CONDITIONID	CONDITION_DESCRIPTION	SNOMED_CODE	DESCRIPTION
101	Pregnancy,Condition	199718001	Elderly primigravida - delivered
101	Pregnancy,Condition	199719009	Elderly primigravida with antenatal problem
101	Pregnancy,Condition	199745000	Complication occurring during labor and delivery
101	Pregnancy,Condition	199746004	Obstructed labor
101	Pregnancy,Condition	199747008	Obstructed labor due to fetal malposition
101	Pregnancy,Condition	199749006	Obstructed labor due to fetal malposition - delivered
101	Pregnancy,Condition	199750006	Obstructed labor due to fetal malposition with antenatal problem
101	Pregnancy,Condition	199757009	Obstructed labor caused by bony pelvis
101	Pregnancy,Condition	199759007	Obstructed labor caused by bony pelvis - delivered
101	Pregnancy,Condition	199760002	Obstructed labor caused by bony pelvis with antenatal problem
101	Pregnancy,Condition	199767004	Obstructed labor caused by pelvic soft tissues
101	Pregnancy,Condition	199769001	Obstructed labor caused by pelvic soft tissues - delivered
101	Pregnancy,Condition	199770000	Obstructed labor caused by pelvic

© 2021 Matheny ME et al. *JAMA Network Open.*

CONDITIONID	CONDITION_DESCRIPTION	SNOMED_CODE	DESCRIPTION
			soft tissues with antenatal problem
101	Pregnancy,Condition	199774009	Deep transverse arrest - delivered
101	Pregnancy,Condition	199775005	Deep transverse arrest with antenatal problem
101	Pregnancy,Condition	199783004	Shoulder dystocia - delivered
101	Pregnancy,Condition	199784005	Shoulder dystocia with antenatal problem
101	Pregnancy,Condition	199787003	Locked twins - delivered
101	Pregnancy,Condition	199788008	Locked twins with antenatal problem
101	Pregnancy,Condition	199819004	Primary uterine inertia - delivered
101	Pregnancy,Condition	199821009	Primary uterine inertia with antenatal problem
101	Pregnancy,Condition	199824001	Secondary uterine inertia - delivered
101	Pregnancy,Condition	199825000	Secondary uterine inertia with antenatal problem
101	Pregnancy,Condition	199833004	Precipitate labor - delivered
101	Pregnancy,Condition	199834005	Precipitate labor with antenatal problem
101	Pregnancy,Condition	199847000	Prolonged first stage - delivered
101	Pregnancy,Condition	199848005	Prolonged first stage with antenatal problem
101	Pregnancy,Condition	199857004	Prolonged second stage - delivered
101	Pregnancy,Condition	199858009	Prolonged second stage with antenatal problem

© 2021 Matheny ME et al. *JAMA Network Open.*

CONDITIONID	CONDITION_DESCRIPTION	SNOMED_CODE	DESCRIPTION
101	Pregnancy,Condition	199860006	Delayed delivery of second twin, triplet etc
101	Pregnancy,Condition	199862003	Delayed delivery second twin - delivered
101	Pregnancy,Condition	199863008	Delayed delivery second twin with antenatal problem
101	Pregnancy,Condition	199869007	Prolapse of cord - delivered
101	Pregnancy,Condition	199870008	Prolapse of cord with antenatal problem
101	Pregnancy,Condition	199874004	Umbilical cord tight around neck - delivered
101	Pregnancy,Condition	199875003	Umbilical cord tight around neck with antenatal problem
101	Pregnancy,Condition	199880007	Cord tangled with compression - delivered
101	Pregnancy,Condition	199881006	Cord tangled with compression with antenatal problem
101	Pregnancy,Condition	199889008	Short cord - delivered
101	Pregnancy,Condition	199890004	Short cord with antenatal problem
101	Pregnancy,Condition	199895009	Vasa previa - delivered
101	Pregnancy,Condition	199896005	Vasa previa with antenatal problem
101	Pregnancy,Condition	199900008	Vascular lesions of cord - delivered
101	Pregnancy,Condition	199901007	Vascular lesions of cord with antenatal problem
101	Pregnancy,Condition	199916005	First degree perineal tear

© 2021 Matheny ME et al. *JAMA Network Open.*

CONDITIONID	CONDITION_DESCRIPTION	SNOMED_CODE	DESCRIPTION
			during delivery - delivered
101	Pregnancy,Condition	199925004	Second degree perineal tear during delivery - delivered
101	Pregnancy,Condition	199926003	Second degree perineal tear during delivery with postnatal problem
101	Pregnancy,Condition	199930000	Third degree perineal tear during delivery - delivered
101	Pregnancy,Condition	199931001	Third degree perineal tear during delivery with postnatal problem
101	Pregnancy,Condition	199934009	Fourth degree perineal tear during delivery - delivered
101	Pregnancy,Condition	199935005	Fourth degree perineal tear during delivery with postnatal problem
101	Pregnancy,Condition	199944006	Vulval and/or perineal hematoma during delivery - delivered
101	Pregnancy,Condition	199945007	Vulval and/or perineal hematoma during delivery with postnatal problem
101	Pregnancy,Condition	199960005	Rupture of uterus before labor - delivered

© 2021 Matheny ME et al. *JAMA Network Open.*

CONDITIONID	CONDITION_DESCRIPTION	SNOMED_CODE	DESCRIPTION
101	Pregnancy,Condition	199961009	Rupture of uterus before labor with antenatal problem
101	Pregnancy,Condition	199964001	Rupture of uterus during and after labor - delivered
101	Pregnancy,Condition	199969006	Obstetric inversion of uterus - delivered with postnatal problem
101	Pregnancy,Condition	199970007	Obstetric inversion of uterus with postnatal problem
101	Pregnancy,Condition	199972004	Laceration of cervix - obstetric
101	Pregnancy,Condition	199974003	Obstetric laceration of cervix - delivered
101	Pregnancy,Condition	199975002	Obstetric laceration of cervix with postnatal problem
101	Pregnancy,Condition	199977005	Obstetric high vaginal laceration
101	Pregnancy,Condition	199979008	Obstetric high vaginal laceration - delivered
101	Pregnancy,Condition	199980006	Obstetric high vaginal laceration with postnatal problem
101	Pregnancy,Condition	199991004	Obstetric damage to pelvic joints and ligaments with postnatal problem
101	Pregnancy,Condition	199996009	Obstetric pelvic hematoma - delivered with postnatal problem
101	Pregnancy,Condition	200025008	Secondary postpartum

© 2021 Matheny ME et al. *JAMA Network Open.*

CONDITIONID	CONDITION_DESCRIPTION	SNOMED_CODE	DESCRIPTION
			hemorrhage - delivered with postnatal problem
101	Pregnancy,Condition	200030007	Postpartum coagulation defects - delivered with postnatal problem
101	Pregnancy,Condition	200031006	Postpartum coagulation defects with postnatal problem
101	Pregnancy,Condition	200038000	Retained placenta with no hemorrhage with postnatal problem
101	Pregnancy,Condition	200099005	Maternal distress - delivered
101	Pregnancy,Condition	200100002	Maternal distress - delivered with postnatal problem
101	Pregnancy,Condition	200101003	Maternal distress with antenatal problem
101	Pregnancy,Condition	200102005	Maternal distress with postnatal problem
101	Pregnancy,Condition	200105007	Obstetric shock - delivered
101	Pregnancy,Condition	200106008	Obstetric shock - delivered with postnatal problem
101	Pregnancy,Condition	200107004	Obstetric shock with antenatal problem
101	Pregnancy,Condition	200108009	Obstetric shock with postnatal problem
101	Pregnancy,Condition	200111005	Maternal hypotension syndrome - delivered

© 2021 Matheny ME et al. *JAMA Network Open.*

CONDITIONID	CONDITION_DESCRIPTION	SNOMED_CODE	DESCRIPTION
101	Pregnancy,Condition	200112003	Maternal hypotension syndrome - delivered with postnatal problem
101	Pregnancy,Condition	200113008	Maternal hypotension syndrome with antenatal problem
101	Pregnancy,Condition	200117009	Post-delivery acute renal failure - delivered with postnatal problem
101	Pregnancy,Condition	200118004	Post-delivery acute renal failure with postnatal problem
101	Pregnancy,Condition	200130005	Forceps delivery - delivered
101	Pregnancy,Condition	200142000	Breech extraction - delivered
101	Pregnancy,Condition	200144004	Deliveries by cesarean
101	Pregnancy,Condition	200146002	Cesarean delivery - delivered
101	Pregnancy,Condition	200181000	Puerperal endometritis - delivered with postnatal complication
101	Pregnancy,Condition	200182007	Puerperal endometritis with postnatal complication
101	Pregnancy,Condition	200196001	Puerperal sepsis with postnatal complication
101	Pregnancy,Condition	200204002	Varicose veins of legs in pregnancy and the puerperium

© 2021 Matheny ME et al. *JAMA Network Open.*

CONDITIONID	CONDITION_DESCRIPTION	SNOMED_CODE	DESCRIPTION
101	Pregnancy,Condition	200206000	Varicose veins of legs in pregnancy and the puerperium - delivered
101	Pregnancy,Condition	200207009	Varicose veins of legs in pregnancy and the puerperium - delivered with postnatal complication
101	Pregnancy,Condition	200208004	Varicose veins of legs in pregnancy and the puerperium with antenatal complication
101	Pregnancy,Condition	200215007	Varicose veins of perineum and vulva in pregnancy and the puerperium - delivered
101	Pregnancy,Condition	200216008	Varicose veins of perineum and vulva in pregnancy and the puerperium - delivered with postnatal complication
101	Pregnancy,Condition	200217004	Varicose veins of perineum and vulva in pregnancy and the puerperium with antenatal complication
101	Pregnancy,Condition	200222004	Superficial thrombophlebitis

© 2021 Matheny ME et al. *JAMA Network Open.*

CONDITIONID	CONDITION_DESCRIPTION	SNOMED_CODE	DESCRIPTION
			in pregnancy and the puerperium
101	Pregnancy,Condition	200224003	Superficial thrombophlebitis in pregnancy and the puerperium - delivered
101	Pregnancy,Condition	200226001	Superficial thrombophlebitis in pregnancy and the puerperium with antenatal complication
101	Pregnancy,Condition	200232006	Antenatal deep vein thrombosis - delivered
101	Pregnancy,Condition	200237000	Postnatal deep vein thrombosis - delivered with postnatal complication
101	Pregnancy,Condition	200238005	Postnatal deep vein thrombosis with postnatal complication
101	Pregnancy,Condition	200277008	Puerperal pyrexia of unknown origin
101	Pregnancy,Condition	200280009	Puerperal pyrexia of unknown origin - delivered with postnatal complication
101	Pregnancy,Condition	200281008	Puerperal pyrexia of unknown origin with postnatal complication
101	Pregnancy,Condition	200284000	Obstetric pulmonary embolism

© 2021 Matheny ME et al. *JAMA Network Open.*

CONDITIONID	CONDITION_DESCRIPTION	SNOMED_CODE	DESCRIPTION
101	Pregnancy,Condition	200286003	Obstetric air pulmonary embolism
101	Pregnancy,Condition	200288002	Obstetric air pulmonary embolism - delivered
101	Pregnancy,Condition	200289005	Obstetric air pulmonary embolism - delivered with postnatal complication
101	Pregnancy,Condition	200290001	Obstetric air pulmonary embolism with antenatal complication
101	Pregnancy,Condition	200291002	Obstetric air pulmonary embolism with postnatal complication
101	Pregnancy,Condition	200294005	Amniotic fluid pulmonary embolism - delivered
101	Pregnancy,Condition	200295006	Amniotic fluid pulmonary embolism - delivered with postnatal complication
101	Pregnancy,Condition	200296007	Amniotic fluid pulmonary embolism with antenatal complication
101	Pregnancy,Condition	200297003	Amniotic fluid pulmonary embolism with

© 2021 Matheny ME et al. *JAMA Network Open.*

CONDITIONID	CONDITION_DESCRIPTION	SNOMED_CODE	DESCRIPTION
			postnatal complication
101	Pregnancy,Condition	200299000	Obstetric blood-clot pulmonary embolism
101	Pregnancy,Condition	200301007	Obstetric blood-clot pulmonary embolism - delivered
101	Pregnancy,Condition	200302000	Obstetric blood-clot pulmonary embolism - delivered with postnatal complication
101	Pregnancy,Condition	200303005	Obstetric blood-clot pulmonary embolism with antenatal complication
101	Pregnancy,Condition	200304004	Obstetric blood-clot pulmonary embolism with postnatal complication
101	Pregnancy,Condition	200308001	Obstetric pyemic and septic pulmonary embolism - delivered
101	Pregnancy,Condition	200309009	Obstetric pyemic and septic pulmonary embolism - delivered with postnatal complication
101	Pregnancy,Condition	200310004	Obstetric pyemic and septic pulmonary embolism with

© 2021 Matheny ME et al. *JAMA Network Open.*

CONDITIONID	CONDITION_DESCRIPTION	SNOMED_CODE	DESCRIPTION
			antenatal complication
101	Pregnancy,Condition	200330000	Puerperal cerebrovascular disorder - delivered
101	Pregnancy,Condition	200331001	Puerperal cerebrovascular disorder - delivered with postnatal complication
101	Pregnancy,Condition	200332008	Puerperal cerebrovascular disorder with antenatal complication
101	Pregnancy,Condition	200333003	Puerperal cerebrovascular disorder with postnatal complication
101	Pregnancy,Condition	200337002	Cesarean wound disruption - delivered with postnatal complication
101	Pregnancy,Condition	200342005	Obstetric perineal wound disruption - delivered with postnatal complication
101	Pregnancy,Condition	200351002	Placental polyp - delivered with postnatal complication
101	Pregnancy,Condition	200352009	Placental polyp with postnatal complication
101	Pregnancy,Condition	200368003	Obstetric nipple infection - delivered with

© 2021 Matheny ME et al. *JAMA Network Open.*

CONDITIONID	CONDITION_DESCRIPTION	SNOMED_CODE	DESCRIPTION
			postnatal complication
101	Pregnancy,Condition	200369006	Obstetric nipple infection with antenatal complication
101	Pregnancy,Condition	200370007	Obstetric nipple infection with postnatal complication
101	Pregnancy,Condition	200374003	Obstetric breast abscess - delivered
101	Pregnancy,Condition	200375002	Obstetric breast abscess - delivered with postnatal complication
101	Pregnancy,Condition	200376001	Obstetric breast abscess with antenatal complication
101	Pregnancy,Condition	200377005	Obstetric breast abscess with postnatal complication
101	Pregnancy,Condition	200382003	Obstetric non-purulent mastitis - delivered with postnatal complication
101	Pregnancy,Condition	200383008	Obstetric non-purulent mastitis with antenatal complication
101	Pregnancy,Condition	200402001	Retracted nipple in pregnancy, the puerperium or lactation
101	Pregnancy,Condition	200405004	Retracted nipple in pregnancy, the puerperium or lactation -

© 2021 Matheny ME et al. *JAMA Network Open.*

CONDITIONID	CONDITION_DESCRIPTION	SNOMED_CODE	DESCRIPTION
			delivered with postnatal complication
101	Pregnancy,Condition	200406003	Retracted nipple in pregnancy, the puerperium or lactation with antenatal complication
101	Pregnancy,Condition	200407007	Retracted nipple in pregnancy, the puerperium or lactation with postnatal complication
101	Pregnancy,Condition	200411001	Cracked nipple in pregnancy, the puerperium or lactation - delivered
101	Pregnancy,Condition	200412008	Cracked nipple in pregnancy, the puerperium or lactation - delivered with postnatal complication
101	Pregnancy,Condition	200413003	Cracked nipple in pregnancy, the puerperium or lactation with antenatal complication
101	Pregnancy,Condition	200414009	Cracked nipple in pregnancy, the puerperium or lactation with postnatal complication
101	Pregnancy,Condition	200416006	Breast engorgement in

© 2021 Matheny ME et al. *JAMA Network Open.*

CONDITIONID	CONDITION_DESCRIPTION	SNOMED_CODE	DESCRIPTION
			pregnancy, the puerperium or lactation
101	Pregnancy,Condition	200418007	Breast engorgement in pregnancy, the puerperium or lactation - delivered
101	Pregnancy,Condition	200419004	Breast engorgement in pregnancy, the puerperium or lactation - delivered with postnatal complication
101	Pregnancy,Condition	200420005	Breast engorgement in pregnancy, the puerperium or lactation with antenatal complication
101	Pregnancy,Condition	200433004	Failure of lactation - delivered
101	Pregnancy,Condition	200434005	Failure of lactation - delivered with postnatal complication
101	Pregnancy,Condition	200435006	Failure of lactation with antenatal complication
101	Pregnancy,Condition	200436007	Failure of lactation with postnatal complication
101	Pregnancy,Condition	200439000	Suppressed lactation - delivered
101	Pregnancy,Condition	200440003	Suppressed lactation -

© 2021 Matheny ME et al. *JAMA Network Open.*

CONDITIONID	CONDITION_DESCRIPTION	SNOMED_CODE	DESCRIPTION
			delivered with postnatal complication
101	Pregnancy,Condition	200441004	Suppressed lactation with antenatal complication
101	Pregnancy,Condition	200442006	Suppressed lactation with postnatal complication
101	Pregnancy,Condition	200446009	Galactorrhea in pregnancy and the puerperium - delivered
101	Pregnancy,Condition	200447000	Galactorrhea in pregnancy and the puerperium - delivered with postnatal complication
101	Pregnancy,Condition	200448005	Galactorrhea in pregnancy and the puerperium with antenatal complication
101	Pregnancy,Condition	200449002	Galactorrhea in pregnancy and the puerperium with postnatal complication
101	Pregnancy,Condition	200473005	Maternal care for diminished fetal movements
101	Pregnancy,Condition	237149005	Fetus with suspected rubella damage via mother
101	Pregnancy,Condition	237243004	Biochemical pregnancy

© 2021 Matheny ME et al. *JAMA Network Open.*

CONDITIONID	CONDITION_DESCRIPTION	SNOMED_CODE	DESCRIPTION
101	Pregnancy,Condition	237256006	Disorder of pelvic size and disproportion
101	Pregnancy,Condition	237279007	Transient hypertension of pregnancy
101	Pregnancy,Condition	249020006	Cervical observation during pregnancy and labor
101	Pregnancy,Condition	267193004	Incomplete legal termination of pregnancy
101	Pregnancy,Condition	267194005	Complete legal termination of pregnancy
101	Pregnancy,Condition	267197003	Antepartum hemorrhage, abruptio placentae and placenta previa
101	Pregnancy,Condition	267199000	Antepartum hemorrhage associated with coagulation defect
101	Pregnancy,Condition	267204006	Genitourinary tract infection in pregnancy
101	Pregnancy,Condition	267207004	Congenital cardiovascular disorders during pregnancy, childbirth and the puerperium
101	Pregnancy,Condition	267214002	Congenital abnormality of uterus - baby delivered
101	Pregnancy,Condition	267215001	Congenital abnormality of uterus - baby

© 2021 Matheny ME et al. *JAMA Network Open.*

CONDITIONID	CONDITION_DESCRIPTION	SNOMED_CODE	DESCRIPTION
			delivered with postpartum complication
101	Pregnancy,Condition	267216000	Congenital abnormality of uterus complicating antenatal care, baby not yet delivered
101	Pregnancy,Condition	267221002	Tumor of uterine body - baby delivered
101	Pregnancy,Condition	267222009	Tumor of uterine body - baby delivered with postpartum complication
101	Pregnancy,Condition	267223004	Tumor of uterine body complicating antenatal care, baby not yet delivered
101	Pregnancy,Condition	267240008	Vaginal abnormality - baby delivered
101	Pregnancy,Condition	267241007	Vaginal abnormality - baby delivered with postpartum complication
101	Pregnancy,Condition	267247006	Vulval abnormality - baby delivered
101	Pregnancy,Condition	267248001	Congenital or acquired abnormality of vulva - baby delivered with postpartum complication

© 2021 Matheny ME et al. *JAMA Network Open.*

CONDITIONID	CONDITION_DESCRIPTION	SNOMED_CODE	DESCRIPTION
101	Pregnancy,Condition	267249009	Congenital or acquired abnormality of vulva complicating antenatal care - baby not yet delivered
101	Pregnancy,Condition	267252001	Fetus with central nervous system malformation
101	Pregnancy,Condition	267254000	Fetus with viral damage via mother
101	Pregnancy,Condition	267266006	Cord tangled or knotted with compression
101	Pregnancy,Condition	267268007	Trauma to perineum and/or vulva during delivery
101	Pregnancy,Condition	267269004	Vulval and perineal hematoma during delivery
101	Pregnancy,Condition	267271004	Obstetric trauma damaging pelvic joints and ligaments
101	Pregnancy,Condition	267272006	Postpartum coagulation defects
101	Pregnancy,Condition	267280004	Venous complication of pregnancy and/or the puerperium
101	Pregnancy,Condition	267282007	Varicose veins of perineum and vulva in pregnancy and the puerperium
101	Pregnancy,Condition	267283002	Superficial thrombophlebitis in pregnancy and the puerperium -

© 2021 Matheny ME et al. *JAMA Network Open.*

CONDITIONID	CONDITION_DESCRIPTION	SNOMED_CODE	DESCRIPTION
			delivered with postnatal complication
101	Pregnancy,Condition	267284008	Obstetric pyemic and septic pulmonary embolism
101	Pregnancy,Condition	267291006	Cracked nipple in pregnancy, the puerperium or lactation
101	Pregnancy,Condition	270498000	Malposition and malpresentation of fetus
101	Pregnancy,Condition	270500004	Prolapse of cord
101	Pregnancy,Condition	270502007	Obstetric breast abscess
101	Pregnancy,Condition	271370008	Deliveries by breech extraction
101	Pregnancy,Condition	271372000	Obstetric non-purulent mastitis
101	Pregnancy,Condition	274119009	Rubella in pregnancy
101	Pregnancy,Condition	275431006	Anal sphincter tear
101	Pregnancy,Condition	282020008	Premature delivery
101	Pregnancy,Condition	284075002	Spotting per vagina in pregnancy
101	Pregnancy,Condition	287979001	Premature/false labor
101	Pregnancy,Condition	289311005	Cord around neck with compression
101	Pregnancy,Condition	303063000	Eclampsia in puerperium
101	Pregnancy,Condition	307734007	Complete inevitable miscarriage complicated by genital tract and pelvic infection
101	Pregnancy,Condition	307735008	Complete inevitable

© 2021 Matheny ME et al. *JAMA Network Open.*

CONDITIONID	CONDITION_DESCRIPTION	SNOMED_CODE	DESCRIPTION
			miscarriage complicated by delayed or excessive hemorrhage
101	Pregnancy,Condition	307746006	Incomplete inevitable miscarriage complicated by embolism
101	Pregnancy,Condition	307749004	Incomplete inevitable miscarriage complicated by genital tract and pelvic infection
101	Pregnancy,Condition	307750004	Complete inevitable miscarriage complicated by embolism
101	Pregnancy,Condition	307752007	Incomplete inevitable miscarriage complicated by delayed or excessive hemorrhage
101	Pregnancy,Condition	308134004	Varicose veins of legs in the puerperium
101	Pregnancy,Condition	308137006	Superficial thrombophlebitis in the puerperium
101	Pregnancy,Condition	312668007	Abnormal fetal heart rate
101	Pregnancy,Condition	361095003	Dehiscence AND/OR disruption of uterine wound in the puerperium

© 2021 Matheny ME et al. *JAMA Network Open.*

CONDITIONID	CONDITION_DESCRIPTION	SNOMED_CODE	DESCRIPTION
101	Pregnancy,Condition	363681007	Pregnancy with abortive outcome
101	Pregnancy,Condition	372046001	Abnormal glucose tolerance test during pregnancy, childbirth and the puerperium
101	Pregnancy,Condition	373896005	Miscarriage complicated by genital-pelvic infection
101	Pregnancy,Condition	373901007	Legal termination of pregnancy complicated by genital-pelvic infection
101	Pregnancy,Condition	373902000	Illegal termination of pregnancy complicated by genital-pelvic infection
101	Pregnancy,Condition	387699008	Primary uterine inertia
101	Pregnancy,Condition	396544001	Cesarean wound disruption
101	Pregnancy,Condition	397752008	Obstetric perineal wound disruption
101	Pregnancy,Condition	397949005	Poor fetal growth affecting management
101	Pregnancy,Condition	398019008	Perineal laceration during delivery
101	Pregnancy,Condition	398254007	Pre-eclampsia
101	Pregnancy,Condition	399031001	Fourth degree perineal laceration
101	Pregnancy,Condition	413338003	Incomplete miscarriage with complication
101	Pregnancy,Condition	413339006	Failed trial of labor - delivered
101	Pregnancy,Condition	415105001	Placental abruption

© 2021 Matheny ME et al. *JAMA Network Open.*

CONDITIONID	CONDITION_DESCRIPTION	SNOMED_CODE	DESCRIPTION
101	Pregnancy,Condition	416413003	Advanced maternal age gravida
101	Pregnancy,Condition	417044008	Hydatidiform mole, benign
101	Pregnancy,Condition	429187001	Continuing pregnancy after intrauterine death of twin fetus
101	Pregnancy,Condition	430883002	Failed instrumental delivery
101	Pregnancy,Condition	430933008	Uterine size for dates discrepancy
101	Pregnancy,Condition	439731006	Septic thrombophlebitis
101	Pregnancy,Condition	442478007	Combined tubal and intrauterine pregnancy
101	Pregnancy,Condition	462166006	Fetal anemia
101	Pregnancy,Condition	472321009	Continuing pregnancy after intrauterine death of one twin with intrauterine retention of dead twin
101	Pregnancy,Condition	609133009	Short cervical length in pregnancy
101	Pregnancy,Condition	609420007	Suspected fetal damage from disease in the mother
101	Pregnancy,Condition	609422004	Suspected fetal damage from radiation
101	Pregnancy,Condition	609446006	Termination of pregnancy with complication
101	Pregnancy,Condition	609447002	Induced termination of pregnancy

© 2021 Matheny ME et al. *JAMA Network Open.*

CONDITIONID	CONDITION_DESCRIPTION	SNOMED_CODE	DESCRIPTION
			complicated by damage to pelvic organs and/or tissues
101	Pregnancy,Condition	609448007	Induced termination of pregnancy complicated by delayed and/or excessive hemorrhage
101	Pregnancy,Condition	609449004	Induced termination of pregnancy complicated by embolism
101	Pregnancy,Condition	609450004	Induced termination of pregnancy complicated by genital-pelvic infection
101	Pregnancy,Condition	609451000	Induced termination of pregnancy complicated by metabolic disorder
101	Pregnancy,Condition	609452007	Induced termination of pregnancy complicated by renal failure
101	Pregnancy,Condition	609453002	Induced termination of pregnancy complicated by shock
101	Pregnancy,Condition	609492009	Termination of pregnancy without complication

© 2021 Matheny ME et al. *JAMA Network Open.*

CONDITIONID	CONDITION_DESCRIPTION	SNOMED_CODE	DESCRIPTION
101	Pregnancy,Condition	609496007	Complication occurring during pregnancy
101	Pregnancy,Condition	171000119107	Maternal obesity complicating pregnancy, childbirth and the puerperium, antepartum
101	Pregnancy,Condition	100941000119100	Epilepsy in mother complicating pregnancy
101	Pregnancy,Condition	10742471000119100	Previous bariatric surgery in mother complicating childbirth
103	Asthma, Condition	12428000	Intrinsic asthma without status asthmaticus
103	Asthma, Condition	31387002	Exercise-induced asthma
103	Asthma, Condition	195949008	Chronic asthmatic bronchitis
103	Asthma, Condition	195967001	Asthma
103	Asthma, Condition	266361008	Intrinsic asthma
103	Asthma, Condition	281239006	Exacerbation of asthma
103	Asthma, Condition	409663006	Cough variant asthma
103	Asthma, Condition	424643009	IgE-mediated allergic asthma
103	Asthma, Condition	442025000	Acute exacerbation of chronic asthmatic bronchitis
103	Asthma, Condition	708090002	Acute severe exacerbation of asthma
103	Asthma, Condition	708093000	Acute exacerbation of allergic asthma

© 2021 Matheny ME et al. *JAMA Network Open.*

CONDITIONID	CONDITION_DESCRIPTION	SNOMED_CODE	DESCRIPTION
103	Asthma, Condition	708094006	Acute exacerbation of intrinsic asthma
103	Asthma, Condition	708095007	Acute severe exacerbation of immunoglobin E-mediated allergic asthma
103	Asthma, Condition	708096008	Acute severe exacerbation of intrinsic asthma
105	Nausea,Condition	16932000	Nausea and vomiting
105	Nausea,Condition	422587007	Nausea
106	Acute Dialysis,Procedure	108241001	Dialysis procedure
106	Acute Dialysis,Procedure	251859005	Dialysis finding
107	Hydronephrosis	43064006	Hydronephrosis
151	Rheumatic Disease (Charlson Comborbidity Definition)	31384009	Polymyositis
151	Rheumatic Disease (Charlson Comborbidity Definition)	55464009	Systemic lupus erythematosus
151	Rheumatic Disease (Charlson Comborbidity Definition)	57160007	Felty's syndrome
151	Rheumatic Disease (Charlson Comborbidity Definition)	69896004	Rheumatoid arthritis
151	Rheumatic Disease (Charlson Comborbidity Definition)	83901003	SjÃ¶gren's syndrome
151	Rheumatic Disease (Charlson Comborbidity Definition)	89155008	Systemic sclerosis
151	Rheumatic Disease (Charlson Comborbidity Definition)	239793008	Rheumatoid arthritis with organ / system involvement
151	Rheumatic Disease (Charlson Comborbidity Definition)	396230008	Dermatomyositis
151	Rheumatic Disease (Charlson Comborbidity Definition)	398726004	Rheumatoid lung disease
151	Rheumatic Disease (Charlson Comborbidity Definition)	414341000	Giant cell arteritis
151	Rheumatic Disease (Charlson Comborbidity Definition)	417373000	Inflammatory polyarthropathy

© 2021 Matheny ME et al. *JAMA Network Open.*

CONDITIONID	CONDITION_DESCRIPTION	SNOMED_CODE	DESCRIPTION
152	Peptic Ulcer Disease (Charlson Comborbidity Definition)	1567007	Chronic gastric ulcer without hemorrhage, without perforation AND without obstruction
152	Peptic Ulcer Disease (Charlson Comborbidity Definition)	3023008	Acute peptic ulcer without hemorrhage, without perforation AND without obstruction
152	Peptic Ulcer Disease (Charlson Comborbidity Definition)	3483000	Chronic peptic ulcer with perforation
152	Peptic Ulcer Disease (Charlson Comborbidity Definition)	4269005	Chronic gastrojejunal ulcer without hemorrhage AND without perforation
152	Peptic Ulcer Disease (Charlson Comborbidity Definition)	10389003	Acute gastrojejunal ulcer without hemorrhage AND without perforation but with obstruction
152	Peptic Ulcer Disease (Charlson Comborbidity Definition)	10897002	Chronic gastrojejunal ulcer with perforation AND with obstruction
152	Peptic Ulcer Disease (Charlson Comborbidity Definition)	12274003	Acute peptic ulcer with hemorrhage
152	Peptic Ulcer Disease (Charlson Comborbidity Definition)	12384004	Chronic peptic ulcer without hemorrhage AND without

© 2021 Matheny ME et al. *JAMA Network Open.*

CONDITIONID	CONDITION_DESCRIPTION	SNOMED_CODE	DESCRIPTION
			perforation but with obstruction
152	Peptic Ulcer Disease (Charlson Comborbidity Definition)	12847006	Acute duodenal ulcer with hemorrhage
152	Peptic Ulcer Disease (Charlson Comborbidity Definition)	17067009	Acute gastric ulcer with hemorrhage AND with perforation but without obstruction
152	Peptic Ulcer Disease (Charlson Comborbidity Definition)	18169007	Duodenal ulcer without hemorrhage AND without perforation but with obstruction
152	Peptic Ulcer Disease (Charlson Comborbidity Definition)	19850005	Acute gastric ulcer with perforation
152	Peptic Ulcer Disease (Charlson Comborbidity Definition)	22157005	Acute peptic ulcer with hemorrhage but without obstruction
152	Peptic Ulcer Disease (Charlson Comborbidity Definition)	22511002	Acute duodenal ulcer with perforation but without obstruction
152	Peptic Ulcer Disease (Charlson Comborbidity Definition)	23693000	Acute duodenal ulcer without hemorrhage, without perforation AND without obstruction
152	Peptic Ulcer Disease (Charlson Comborbidity Definition)	23812009	Duodenal ulcer with hemorrhage AND perforation
152	Peptic Ulcer Disease (Charlson Comborbidity Definition)	24001002	Chronic gastrojejunal ulcer with hemorrhage,

© 2021 Matheny ME et al. *JAMA Network Open.*

CONDITIONID	CONDITION_DESCRIPTION	SNOMED_CODE	DESCRIPTION
			with perforation AND with obstruction
152	Peptic Ulcer Disease (Charlson Comborbidity Definition)	28082003	Chronic duodenal ulcer without hemorrhage AND without perforation but with obstruction
152	Peptic Ulcer Disease (Charlson Comborbidity Definition)	28945005	Acute peptic ulcer with hemorrhage, with perforation AND with obstruction
152	Peptic Ulcer Disease (Charlson Comborbidity Definition)	30514008	Acute gastrojejunal ulcer without hemorrhage AND without perforation
152	Peptic Ulcer Disease (Charlson Comborbidity Definition)	31301004	Chronic gastric ulcer with perforation
152	Peptic Ulcer Disease (Charlson Comborbidity Definition)	31452001	Gastric ulcer without hemorrhage AND without perforation but with obstruction
152	Peptic Ulcer Disease (Charlson Comborbidity Definition)	34021006	Chronic duodenal ulcer with hemorrhage AND obstruction
152	Peptic Ulcer Disease (Charlson Comborbidity Definition)	34580000	Duodenal ulcer without hemorrhage, without perforation AND without obstruction
152	Peptic Ulcer Disease (Charlson Comborbidity Definition)	34602004	Chronic duodenal ulcer with

© 2021 Matheny ME et al. *JAMA Network Open.*

CONDITIONID	CONDITION_DESCRIPTION	SNOMED_CODE	DESCRIPTION
			perforation but without obstruction
152	Peptic Ulcer Disease (Charlson Comborbidity Definition)	34921009	Acute peptic ulcer with perforation but without obstruction
152	Peptic Ulcer Disease (Charlson Comborbidity Definition)	35517004	Gastrojejunal ulcer without hemorrhage, without perforation AND without obstruction
152	Peptic Ulcer Disease (Charlson Comborbidity Definition)	35681000	Acute peptic ulcer with perforation AND obstruction
152	Peptic Ulcer Disease (Charlson Comborbidity Definition)	36246001	Chronic gastric ulcer with perforation but without obstruction
152	Peptic Ulcer Disease (Charlson Comborbidity Definition)	37442009	Peptic ulcer without hemorrhage AND without perforation
152	Peptic Ulcer Disease (Charlson Comborbidity Definition)	38365000	Peptic ulcer without hemorrhage, without perforation AND without obstruction
152	Peptic Ulcer Disease (Charlson Comborbidity Definition)	40214005	Chronic duodenal ulcer without hemorrhage AND without perforation
152	Peptic Ulcer Disease (Charlson Comborbidity Definition)	41626001	Chronic gastrojejunal ulcer

© 2021 Matheny ME et al. *JAMA Network Open.*

CONDITIONID	CONDITION_DESCRIPTION	SNOMED_CODE	DESCRIPTION
			without hemorrhage, without perforation AND without obstruction
152	Peptic Ulcer Disease (Charlson Comborbidity Definition)	41986000	Acute duodenal ulcer with hemorrhage, with perforation AND with obstruction
152	Peptic Ulcer Disease (Charlson Comborbidity Definition)	43406003	Acute peptic ulcer with hemorrhage AND obstruction
152	Peptic Ulcer Disease (Charlson Comborbidity Definition)	43694004	Acute gastric ulcer with perforation AND obstruction
152	Peptic Ulcer Disease (Charlson Comborbidity Definition)	45485004	Acute peptic ulcer without hemorrhage AND without perforation
152	Peptic Ulcer Disease (Charlson Comborbidity Definition)	46523000	Chronic gastrojejunal ulcer with hemorrhage AND with perforation but without obstruction
152	Peptic Ulcer Disease (Charlson Comborbidity Definition)	46708007	Acute gastric ulcer with hemorrhage AND obstruction
152	Peptic Ulcer Disease (Charlson Comborbidity Definition)	47064007	Acute peptic ulcer with hemorrhage AND with perforation but without obstruction
152	Peptic Ulcer Disease (Charlson Comborbidity Definition)	47152002	Gastrojejunal ulcer without hemorrhage AND

© 2021 Matheny ME et al. *JAMA Network Open.*

CONDITIONID	CONDITION_DESCRIPTION	SNOMED_CODE	DESCRIPTION
			without perforation but with obstruction
152	Peptic Ulcer Disease (Charlson Comborbidity Definition)	48974009	Acute gastric ulcer with hemorrhage AND perforation
152	Peptic Ulcer Disease (Charlson Comborbidity Definition)	49232000	Chronic peptic ulcer with hemorrhage
152	Peptic Ulcer Disease (Charlson Comborbidity Definition)	49916007	Chronic duodenal ulcer with perforation
152	Peptic Ulcer Disease (Charlson Comborbidity Definition)	51847008	Acute duodenal ulcer with hemorrhage AND with perforation but without obstruction
152	Peptic Ulcer Disease (Charlson Comborbidity Definition)	53337006	Acute gastric ulcer with hemorrhage, with perforation AND with obstruction
152	Peptic Ulcer Disease (Charlson Comborbidity Definition)	54053008	Acute gastric ulcer without hemorrhage, without perforation AND without obstruction
152	Peptic Ulcer Disease (Charlson Comborbidity Definition)	54157007	Peptic ulcer without hemorrhage AND without perforation but with obstruction
152	Peptic Ulcer Disease (Charlson Comborbidity Definition)	55746001	Chronic peptic ulcer with hemorrhage AND with perforation

© 2021 Matheny ME et al. *JAMA Network Open.*

CONDITIONID	CONDITION_DESCRIPTION	SNOMED_CODE	DESCRIPTION
			but without obstruction
152	Peptic Ulcer Disease (Charlson Comborbidity Definition)	56461008	Chronic peptic ulcer with hemorrhage AND obstruction
152	Peptic Ulcer Disease (Charlson Comborbidity Definition)	56579005	Chronic gastrojejunal ulcer without hemorrhage AND without perforation but with obstruction
152	Peptic Ulcer Disease (Charlson Comborbidity Definition)	56776001	Duodenal ulcer without hemorrhage AND without perforation
152	Peptic Ulcer Disease (Charlson Comborbidity Definition)	57246001	Chronic gastric ulcer with hemorrhage
152	Peptic Ulcer Disease (Charlson Comborbidity Definition)	57871005	Chronic peptic ulcer with perforation AND obstruction
152	Peptic Ulcer Disease (Charlson Comborbidity Definition)	57940000	Chronic duodenal ulcer without hemorrhage, without perforation AND without obstruction
152	Peptic Ulcer Disease (Charlson Comborbidity Definition)	58085004	Acute peptic ulcer without hemorrhage AND without perforation but with obstruction
152	Peptic Ulcer Disease (Charlson Comborbidity Definition)	58711008	Acute gastrojejunal ulcer with hemorrhage, with

© 2021 Matheny ME et al. *JAMA Network Open.*

CONDITIONID	CONDITION_DESCRIPTION	SNOMED_CODE	DESCRIPTION
			perforation AND with obstruction
152	Peptic Ulcer Disease (Charlson Comborbidity Definition)	59515005	Acute gastrojejunal ulcer with hemorrhage but without obstruction
152	Peptic Ulcer Disease (Charlson Comborbidity Definition)	59913009	Gastric ulcer without hemorrhage, without perforation AND without obstruction
152	Peptic Ulcer Disease (Charlson Comborbidity Definition)	60400003	Chronic peptic ulcer without hemorrhage, without perforation AND without obstruction
152	Peptic Ulcer Disease (Charlson Comborbidity Definition)	60531007	Chronic gastric ulcer without hemorrhage AND without perforation but with obstruction
152	Peptic Ulcer Disease (Charlson Comborbidity Definition)	60551006	Chronic duodenal ulcer with perforation AND obstruction
152	Peptic Ulcer Disease (Charlson Comborbidity Definition)	61300005	Chronic peptic ulcer with hemorrhage AND perforation
152	Peptic Ulcer Disease (Charlson Comborbidity Definition)	61347001	Acute duodenal ulcer with perforation
152	Peptic Ulcer Disease (Charlson Comborbidity Definition)	62341002	Chronic duodenal ulcer with hemorrhage but

© 2021 Matheny ME et al. *JAMA Network Open.*

CONDITIONID	CONDITION_DESCRIPTION	SNOMED_CODE	DESCRIPTION
			without obstruction
152	Peptic Ulcer Disease (Charlson Comborbidity Definition)	62477005	Chronic gastrojejunal ulcer with perforation but without obstruction
152	Peptic Ulcer Disease (Charlson Comborbidity Definition)	62838000	Chronic gastrojejunal ulcer with hemorrhage
152	Peptic Ulcer Disease (Charlson Comborbidity Definition)	62936002	Acute duodenal ulcer with perforation AND obstruction
152	Peptic Ulcer Disease (Charlson Comborbidity Definition)	63954007	Acute gastrojejunal ulcer with hemorrhage
152	Peptic Ulcer Disease (Charlson Comborbidity Definition)	66636001	Acute gastrojejunal ulcer with perforation
152	Peptic Ulcer Disease (Charlson Comborbidity Definition)	66673003	Acute gastrojejunal ulcer with hemorrhage AND with perforation but without obstruction
152	Peptic Ulcer Disease (Charlson Comborbidity Definition)	66767006	Acute duodenal ulcer with hemorrhage but without obstruction
152	Peptic Ulcer Disease (Charlson Comborbidity Definition)	67964002	Acute gastric ulcer without hemorrhage AND without perforation
152	Peptic Ulcer Disease (Charlson Comborbidity Definition)	70418001	Acute gastric ulcer with hemorrhage but without obstruction

© 2021 Matheny ME et al. *JAMA Network Open.*

CONDITIONID	CONDITION_DESCRIPTION	SNOMED_CODE	DESCRIPTION
152	Peptic Ulcer Disease (Charlson Comborbidity Definition)	72219001	Acute gastrojejunal ulcer with perforation AND obstruction
152	Peptic Ulcer Disease (Charlson Comborbidity Definition)	72395008	Acute gastrojejunal ulcer with perforation but without obstruction
152	Peptic Ulcer Disease (Charlson Comborbidity Definition)	72408002	Acute gastrojejunal ulcer with hemorrhage AND obstruction
152	Peptic Ulcer Disease (Charlson Comborbidity Definition)	72486001	Gastric ulcer with perforation AND obstruction
152	Peptic Ulcer Disease (Charlson Comborbidity Definition)	74341002	Chronic gastric ulcer with hemorrhage AND with perforation but without obstruction
152	Peptic Ulcer Disease (Charlson Comborbidity Definition)	75342000	Acute duodenal ulcer without hemorrhage AND without perforation but with obstruction
152	Peptic Ulcer Disease (Charlson Comborbidity Definition)	76078009	Chronic gastric ulcer with hemorrhage but without obstruction
152	Peptic Ulcer Disease (Charlson Comborbidity Definition)	76181002	Chronic gastric ulcer with hemorrhage AND with perforation
152	Peptic Ulcer Disease (Charlson Comborbidity Definition)	76796008	Chronic gastric ulcer without hemorrhage AND

© 2021 Matheny ME et al. *JAMA Network Open.*

CONDITIONID	CONDITION_DESCRIPTION	SNOMED_CODE	DESCRIPTION
			without perforation
152	Peptic Ulcer Disease (Charlson Comborbidity Definition)	77661009	Chronic peptic ulcer with hemorrhage, with perforation AND with obstruction
152	Peptic Ulcer Disease (Charlson Comborbidity Definition)	77987006	Acute gastrojejunal ulcer without hemorrhage, without perforation AND without obstruction
152	Peptic Ulcer Disease (Charlson Comborbidity Definition)	79118000	Acute peptic ulcer with perforation
152	Peptic Ulcer Disease (Charlson Comborbidity Definition)	80953005	Chronic peptic ulcer with perforation but without obstruction
152	Peptic Ulcer Disease (Charlson Comborbidity Definition)	81142005	Chronic duodenal ulcer with hemorrhage AND with perforation but without obstruction
152	Peptic Ulcer Disease (Charlson Comborbidity Definition)	81225008	Acute gastric ulcer without hemorrhage AND without perforation but with obstruction
152	Peptic Ulcer Disease (Charlson Comborbidity Definition)	81387001	Acute gastrojejunal ulcer with hemorrhage AND perforation
152	Peptic Ulcer Disease (Charlson Comborbidity Definition)	81518000	Chronic peptic ulcer with hemorrhage but

© 2021 Matheny ME et al. *JAMA Network Open.*

CONDITIONID	CONDITION_DESCRIPTION	SNOMED_CODE	DESCRIPTION
			without obstruction
152	Peptic Ulcer Disease (Charlson Comborbidity Definition)	85787009	Chronic gastric ulcer with hemorrhage, with perforation AND with obstruction
152	Peptic Ulcer Disease (Charlson Comborbidity Definition)	85859006	Chronic gastric ulcer with hemorrhage AND with obstruction
152	Peptic Ulcer Disease (Charlson Comborbidity Definition)	86258000	Chronic duodenal ulcer with hemorrhage, with perforation AND with obstruction
152	Peptic Ulcer Disease (Charlson Comborbidity Definition)	86895006	Acute duodenal ulcer with hemorrhage AND perforation
152	Peptic Ulcer Disease (Charlson Comborbidity Definition)	87756006	Acute duodenal ulcer with hemorrhage AND obstruction
152	Peptic Ulcer Disease (Charlson Comborbidity Definition)	89469000	Chronic duodenal ulcer with hemorrhage
152	Peptic Ulcer Disease (Charlson Comborbidity Definition)	89748001	Acute gastric ulcer with hemorrhage
152	Peptic Ulcer Disease (Charlson Comborbidity Definition)	90257004	Chronic gastrojejunal ulcer with hemorrhage AND obstruction
152	Peptic Ulcer Disease (Charlson Comborbidity Definition)	90628007	Acute gastric ulcer with perforation but without obstruction
152	Peptic Ulcer Disease (Charlson Comborbidity Definition)	111353003	Acute peptic ulcer with hemorrhage and perforation

© 2021 Matheny ME et al. *JAMA Network Open.*

CONDITIONID	CONDITION_DESCRIPTION	SNOMED_CODE	DESCRIPTION
152	Peptic Ulcer Disease (Charlson Comborbidity Definition)	128287004	Chronic peptic ulcer
152	Peptic Ulcer Disease (Charlson Comborbidity Definition)	397825006	Gastric ulcer
153	Hemiplegia or Paraplegia (Charlson Comborbidity Definition)	1593000	Infantile hemiplegia
153	Hemiplegia or Paraplegia (Charlson Comborbidity Definition)	8663007	Cauda equina syndrome without neurogenic bladder
153	Hemiplegia or Paraplegia (Charlson Comborbidity Definition)	11538006	Tetraplegia
153	Hemiplegia or Paraplegia (Charlson Comborbidity Definition)	12454008	Cauda equina syndrome with neurogenic bladder
153	Hemiplegia or Paraplegia (Charlson Comborbidity Definition)	39912006	Hereditary spastic paraplegia
153	Hemiplegia or Paraplegia (Charlson Comborbidity Definition)	41764006	Monoplegia of upper limb
153	Hemiplegia or Paraplegia (Charlson Comborbidity Definition)	43486001	Hemiplegic cerebral palsy
153	Hemiplegia or Paraplegia (Charlson Comborbidity Definition)	44695005	Paralysis
153	Hemiplegia or Paraplegia (Charlson Comborbidity Definition)	50582007	Hemiplegia
153	Hemiplegia or Paraplegia (Charlson Comborbidity Definition)	54099005	Diplegia of upper limbs
153	Hemiplegia or Paraplegia (Charlson Comborbidity Definition)	56409008	Monoplegic cerebral palsy
153	Hemiplegia or Paraplegia (Charlson Comborbidity Definition)	58193001	Diplegic cerebral palsy
153	Hemiplegia or Paraplegia (Charlson Comborbidity Definition)	60389000	Paraplegia
153	Hemiplegia or Paraplegia (Charlson Comborbidity Definition)	79633009	Spastic hemiplegia
153	Hemiplegia or Paraplegia (Charlson Comborbidity Definition)	80420005	Monoplegia of lower limb
153	Hemiplegia or Paraplegia (Charlson Comborbidity Definition)	80935004	Flaccid hemiplegia
153	Hemiplegia or Paraplegia (Charlson Comborbidity Definition)	86022000	Monoplegia

© 2021 Matheny ME et al. *JAMA Network Open.*

CONDITIONID	CONDITION_DESCRIPTION	SNOMED_CODE	DESCRIPTION
153	Hemiplegia or Paraplegia (Charlson Comborbidity Definition)	128188000	Cerebral palsy
153	Hemiplegia or Paraplegia (Charlson Comborbidity Definition)	192970008	Cauda equina syndrome
153	Hemiplegia or Paraplegia (Charlson Comborbidity Definition)	275468009	Congenital quadriplegia
153	Hemiplegia or Paraplegia (Charlson Comborbidity Definition)	426167003	Monoplegia of lower limb affecting dominant side
153	Hemiplegia or Paraplegia (Charlson Comborbidity Definition)	426536006	Monoplegia of upper limb affecting non-dominant side
153	Hemiplegia or Paraplegia (Charlson Comborbidity Definition)	426934001	Monoplegia of lower limb affecting non-dominant side
153	Hemiplegia or Paraplegia (Charlson Comborbidity Definition)	441688003	Incomplete tetraplegia due to spinal cord lesion at C1-C4 level
153	Hemiplegia or Paraplegia (Charlson Comborbidity Definition)	441705005	Complete tetraplegia due to lesion at C1-C4 level
153	Hemiplegia or Paraplegia (Charlson Comborbidity Definition)	441717007	Hemiplegia of nondominant side
153	Hemiplegia or Paraplegia (Charlson Comborbidity Definition)	441722007	Spastic hemiplegia of nondominant side
153	Hemiplegia or Paraplegia (Charlson Comborbidity Definition)	441794001	Incomplete tetraplegia due to lesion at C5-C7 level
153	Hemiplegia or Paraplegia (Charlson Comborbidity Definition)	441892008	Spastic hemiplegia of dominant side
153	Hemiplegia or Paraplegia (Charlson Comborbidity Definition)	441951003	Monoplegia of upper limb of dominant side

© 2021 Matheny ME et al. *JAMA Network Open.*

CONDITIONID	CONDITION_DESCRIPTION	SNOMED_CODE	DESCRIPTION
153	Hemiplegia or Paraplegia (Charlson Comborbidity Definition)	441980007	Complete tetraplegia due to lesion at C5-C7 level
153	Hemiplegia or Paraplegia (Charlson Comborbidity Definition)	442020005	Flaccid hemiplegia of dominant side
153	Hemiplegia or Paraplegia (Charlson Comborbidity Definition)	442077006	Flaccid hemiplegia of nondominant side
153	Hemiplegia or Paraplegia (Charlson Comborbidity Definition)	442155009	Hemiplegia of dominant side
200	Constipation, Condition	14760008	Constipation
200	Constipation, Condition	33995003	Megacolon, not Hirschsprung's
200	Constipation, Condition	35298007	Slow transit constipation
200	Constipation, Condition	44635007	Fecal impaction
200	Constipation, Condition	62851005	Impaction of intestine
200	Constipation, Condition	81060008	Intestinal obstruction
200	Constipation, Condition	85920003	Constipation by outlet obstruction
200	Constipation, Condition	308925008	Digestive symptom
202	Cardiac Arrest, Diagnosis	71908006	Ventricular fibrillation
202	Cardiac Arrest, Diagnosis	111288001	Ventricular flutter
202	Cardiac Arrest, Diagnosis	410429000	Cardiac arrest
203	Stable Angina, Diagnosis	59021001	Angina decubitus
203	Stable Angina, Diagnosis	87343002	Prinzmetal angina
203	Stable Angina, Diagnosis	194828000	Angina pectoris
204	Diabetic Neuropathy, Diagnosis	49455004	Diabetic polyneuropathy
207	Anal Fissures or Fistula, Diagnosis	72779005	Anorectal fistula
208	Hemorrhoids, Diagnosis	23913003	External hemorrhoids
208	Hemorrhoids, Diagnosis	26373009	Thrombosed external hemorrhoids

© 2021 Matheny ME et al. *JAMA Network Open.*

CONDITIONID	CONDITION_DESCRIPTION	SNOMED_CODE	DESCRIPTION
208	Hemorrhoids, Diagnosis	31704005	Residual hemorrhoidal skin tags
208	Hemorrhoids, Diagnosis	38214006	Internal hemorrhoids without complication
208	Hemorrhoids, Diagnosis	38996000	External hemorrhoids without complication
208	Hemorrhoids, Diagnosis	52931009	Thrombosed internal hemorrhoids
208	Hemorrhoids, Diagnosis	70153002	Hemorrhoids
208	Hemorrhoids, Diagnosis	75955007	Thrombosed hemorrhoids
208	Hemorrhoids, Diagnosis	90458007	Internal hemorrhoids
209	Impaction, Diagnosis	44635007	Fecal impaction
210	Gastroparesis, Diagnosis	235675006	Gastroparesis syndrome
211	BowelPerforation, Diagnosis	56905009	Perforation of intestine
215	Irritable Bowel Syndrome, Diagnosis	10743008	Irritable bowel syndrome
216	Rectal prolapse, Diagnosis	57773001	Rectal prolapse
217	Rectocele, Diagnosis	447072005	Herniation of rectum into vagina
218	Proctalgia Fugax, Diagnosis	17440005	Anal spasm
219	Megacolon, Diagnosis	33995003	Megacolon, not Hirschsprung's
221	GI Perforation, Diagnosis	56905009	Perforation of intestine
222	Chronic Fatigue, Diagnosis	52702003	Chronic fatigue syndrome
224	Rheumatoid Arthritis, Diagnosis	69896004	Rheumatoid arthritis
225	Osteoarthritis, Diagnosis	417662000	History of clinical finding in subject
227	Migraine & Headache, Diagnosis	25064002	Headache

© 2021 Matheny ME et al. *JAMA Network Open.*

CONDITIONID	CONDITION_DESCRIPTION	SNOMED_CODE	DESCRIPTION
228	Fibromyalgia, Diagnosis	68962001	Muscle pain
229	Myopathies, Diagnosis	85898001	Cardiomyopathy
229	Myopathies, Diagnosis	129565002	Disorder of muscle
229	Myopathies, Diagnosis	443819006	Critical illness myopathy
231	Amyloidosis, Diagnosis	12579009	Familial Mediterranean fever
231	Amyloidosis, Diagnosis	17602002	Amyloidosis
232	Dermatomyositis, Diagnosis	396230008	Dermatomyositis
234	Scleroderma, Diagnosis	201048007	Localized scleroderma
235	Hyperkalemia, Diagnosis	14140009	Hyperkalemia
236	Hyperparathyroidism, Diagnosis	36348003	Primary hyperparathyroidism
236	Hyperparathyroidism, Diagnosis	66999008	Hyperparathyroidism
236	Hyperparathyroidism, Diagnosis	91478007	Secondary hyperparathyroidism
239	Porphyria, Diagnosis	29094004	Disorder of porphyrin metabolism
240	Multiple Endocrine Neoplasia, Diagnosis	30664006	Multiple endocrine neoplasia, type 1
240	Multiple Endocrine Neoplasia, Diagnosis	46724008	Polyglandular activity in multiple endocrine adenomatosis
240	Multiple Endocrine Neoplasia, Diagnosis	61530001	Multiple endocrine neoplasia, type 3
240	Multiple Endocrine Neoplasia, Diagnosis	61808009	Multiple endocrine neoplasia, type 2
241	Autonomic Neuropathy, Diagnosis	15241006	Disorder of autonomic nervous system
243	ParkinsonÃ¯Â¿Â½s Disease, Diagnosis	49049000	Parkinson's disease
244	Spinal Cord Injury / Tumors, Diagnosis	81642009	Late effect of spinal cord injury

© 2021 Matheny ME et al. *JAMA Network Open.*

CONDITIONID	CONDITION_DESCRIPTION	SNOMED_CODE	DESCRIPTION
244	Spinal Cord Injury / Tumors, Diagnosis	398042001	Accidental dural puncture
280	Myocardial Infarction, Diagnosis	1755008	Old myocardial infarction
280	Myocardial Infarction, Diagnosis	54329005	Acute myocardial infarction of anterior wall
280	Myocardial Infarction, Diagnosis	57054005	Acute myocardial infarction
280	Myocardial Infarction, Diagnosis	58612006	Acute myocardial infarction of lateral wall
280	Myocardial Infarction, Diagnosis	65547006	Acute myocardial infarction of inferolateral wall
280	Myocardial Infarction, Diagnosis	70211005	Acute myocardial infarction of anterolateral wall
280	Myocardial Infarction, Diagnosis	70422006	Acute subendocardial infarction
280	Myocardial Infarction, Diagnosis	73795002	Acute myocardial infarction of inferior wall
280	Myocardial Infarction, Diagnosis	76593002	Acute myocardial infarction of inferoposterior wall
280	Myocardial Infarction, Diagnosis	194802003	True posterior myocardial infarction
280	Myocardial Infarction, Diagnosis	194809007	Acute myocardial infarction of atrium
281	Congestive Heart Failure, Diagnosis	42343007	Congestive heart failure
281	Congestive Heart Failure, Diagnosis	84114007	Heart failure
281	Congestive Heart Failure, Diagnosis	85232009	Left heart failure
281	Congestive Heart Failure, Diagnosis	417996009	Systolic heart failure
281	Congestive Heart Failure, Diagnosis	418304008	Diastolic heart failure

© 2021 Matheny ME et al. *JAMA Network Open.*

CONDITIONID	CONDITION_DESCRIPTION	SNOMED_CODE	DESCRIPTION
281	Congestive Heart Failure, Diagnosis	441481004	Chronic systolic heart failure
281	Congestive Heart Failure, Diagnosis	441530006	Chronic diastolic heart failure
281	Congestive Heart Failure, Diagnosis	442304009	Combined systolic and diastolic dysfunction
281	Congestive Heart Failure, Diagnosis	443253003	Acute on chronic systolic heart failure
281	Congestive Heart Failure, Diagnosis	443254009	Acute systolic heart failure
281	Congestive Heart Failure, Diagnosis	443343001	Acute diastolic heart failure
281	Congestive Heart Failure, Diagnosis	443344007	Acute on chronic diastolic heart failure
281	Congestive Heart Failure, Diagnosis	153931000119109	Acute combined systolic and diastolic heart failure
281	Congestive Heart Failure, Diagnosis	153941000119100	Chronic combined systolic and diastolic heart failure
281	Congestive Heart Failure, Diagnosis	153951000119103	Acute on chronic combined systolic and diastolic heart failure
282	Peripheral Vascular Disease	14336007	Ruptured abdominal aortic aneurysm
282	Peripheral Vascular Disease	67362008	Aortic aneurysm
282	Peripheral Vascular Disease	73067008	Ruptured aortic aneurysm
282	Peripheral Vascular Disease	75878002	Abdominal aortic aneurysm without rupture
282	Peripheral Vascular Disease	161678007	H/O: artificial blood vessel

© 2021 Matheny ME et al. *JAMA Network Open.*

CONDITIONID	CONDITION_DESCRIPTION	SNOMED_CODE	DESCRIPTION
282	Peripheral Vascular Disease	195258006	Thoracic aortic aneurysm which has ruptured
282	Peripheral Vascular Disease	195265003	Thoracoabdominal aortic aneurysm, ruptured
282	Peripheral Vascular Disease	233984007	Thoracoabdominal aortic aneurysm
282	Peripheral Vascular Disease	233994002	Dissection of thoracic aorta
282	Peripheral Vascular Disease	308546005	Dissection of aorta
282	Peripheral Vascular Disease	372070002	Gangrenous disorder
282	Peripheral Vascular Disease	400047006	Peripheral vascular disease
282	Peripheral Vascular Disease	408666009	Dissection of abdominal aorta
282	Peripheral Vascular Disease	433068007	Aneurysm of thoracic aorta
282	Peripheral Vascular Disease	713029000	Dissection of thoracoabdominal aorta
283	Cerebrovascular Disease, Diagnosis	1386000	Intracranial hemorrhage
283	Cerebrovascular Disease, Diagnosis	2929001	Occlusion of artery
283	Cerebrovascular Disease, Diagnosis	15258001	Subclavian steal syndrome
283	Cerebrovascular Disease, Diagnosis	20059004	Cerebral artery occlusion
283	Cerebrovascular Disease, Diagnosis	20262006	Ataxia
283	Cerebrovascular Disease, Diagnosis	21454007	Subarachnoid hemorrhage
283	Cerebrovascular Disease, Diagnosis	28366008	Cerebral arteritis
283	Cerebrovascular Disease, Diagnosis	34781003	Vertebral artery syndrome
283	Cerebrovascular Disease, Diagnosis	35486000	Subdural hemorrhage
283	Cerebrovascular Disease, Diagnosis	42970005	Nonpyogenic thrombosis of intracranial venous sinus

© 2021 Matheny ME et al. *JAMA Network Open.*

CONDITIONID	CONDITION_DESCRIPTION	SNOMED_CODE	DESCRIPTION
283	Cerebrovascular Disease, Diagnosis	42994005	Nonruptured cerebral aneurysm
283	Cerebrovascular Disease, Diagnosis	43658003	Vertebral artery obstruction
283	Cerebrovascular Disease, Diagnosis	50490005	Hypertensive encephalopathy
283	Cerebrovascular Disease, Diagnosis	55382008	Cerebral atherosclerosis
283	Cerebrovascular Disease, Diagnosis	62914000	Cerebrovascular disease
283	Cerebrovascular Disease, Diagnosis	64009001	Basilar artery syndrome
283	Cerebrovascular Disease, Diagnosis	69116000	Moyamoya disease
283	Cerebrovascular Disease, Diagnosis	69798007	Carotid artery obstruction
283	Cerebrovascular Disease, Diagnosis	71444005	Cerebral thrombosis
283	Cerebrovascular Disease, Diagnosis	75543006	Cerebral embolism
283	Cerebrovascular Disease, Diagnosis	95666008	Weakness of face muscles
283	Cerebrovascular Disease, Diagnosis	195180004	Basilar artery occlusion
283	Cerebrovascular Disease, Diagnosis	195183002	Multiple and bilateral precerebral arterial occlusion
283	Cerebrovascular Disease, Diagnosis	195189003	Cerebral infarction due to thrombosis of cerebral arteries
283	Cerebrovascular Disease, Diagnosis	195190007	Cerebral infarction due to embolism of cerebral arteries
283	Cerebrovascular Disease, Diagnosis	195199008	Vertebrobasilar artery syndrome
283	Cerebrovascular Disease, Diagnosis	195239002	Late effects of cerebrovascular disease
283	Cerebrovascular Disease, Diagnosis	229621000	Disorder of fluency
283	Cerebrovascular Disease, Diagnosis	230692004	Infarction - precerebral

© 2021 Matheny ME et al. *JAMA Network Open.*

CONDITIONID	CONDITION_DESCRIPTION	SNOMED_CODE	DESCRIPTION
283	Cerebrovascular Disease, Diagnosis	230736007	Transient global amnesia
283	Cerebrovascular Disease, Diagnosis	266253001	Precerebral arterial occlusion
283	Cerebrovascular Disease, Diagnosis	266257000	Transient cerebral ischemia
283	Cerebrovascular Disease, Diagnosis	274100004	Cerebral hemorrhage
283	Cerebrovascular Disease, Diagnosis	287731003	Cerebral ischemia
283	Cerebrovascular Disease, Diagnosis	288723005	Acute ill-defined cerebrovascular disease
283	Cerebrovascular Disease, Diagnosis	397809001	Nontraumatic extradural hemorrhage
283	Cerebrovascular Disease, Diagnosis	425642008	Monoplegia of dominant lower limb as a late effect of cerebrovascular accident
283	Cerebrovascular Disease, Diagnosis	425882004	Paralytic syndrome as late effect of stroke
283	Cerebrovascular Disease, Diagnosis	426033005	Dysphagia as a late effect of cerebrovascular accident
283	Cerebrovascular Disease, Diagnosis	426788002	Vertigo as late effect of stroke
283	Cerebrovascular Disease, Diagnosis	427065003	Monoplegia of dominant upper limb as a late effect of cerebrovascular accident
283	Cerebrovascular Disease, Diagnosis	428668000	Apraxia due to cerebrovascular accident
283	Cerebrovascular Disease, Diagnosis	430947007	Paralytic syndrome of nondominant side as late effect of stroke

© 2021 Matheny ME et al. *JAMA Network Open.*

CONDITIONID	CONDITION_DESCRIPTION	SNOMED_CODE	DESCRIPTION
283	Cerebrovascular Disease, Diagnosis	430959006	Paralytic syndrome of dominant side as late effect of stroke
283	Cerebrovascular Disease, Diagnosis	441529001	Dysphasia as late effect of cerebrovascular disease
283	Cerebrovascular Disease, Diagnosis	441630004	Aphasia as late effect of cerebrovascular disease
283	Cerebrovascular Disease, Diagnosis	441735003	Sensory disorder as a late effect of cerebrovascular disease
283	Cerebrovascular Disease, Diagnosis	441887006	Monoplegia of lower limb as late effect of cerebrovascular disease
283	Cerebrovascular Disease, Diagnosis	441894009	Monoplegia of nondominant lower limb as a late effect of cerebrovascular accident
283	Cerebrovascular Disease, Diagnosis	441960006	Speech and language deficit as late effect of cerebrovascular accident
283	Cerebrovascular Disease, Diagnosis	442024001	Hemiplegia as late effect of cerebrovascular disease
283	Cerebrovascular Disease, Diagnosis	442097001	Monoplegia of upper limb as late effect of cerebrovascular disease

© 2021 Matheny ME et al. *JAMA Network Open.*

CONDITIONID	CONDITION_DESCRIPTION	SNOMED_CODE	DESCRIPTION
283	Cerebrovascular Disease, Diagnosis	442181008	Monoplegia of nondominant upper limb as a late effect of cerebrovascular accident
283	Cerebrovascular Disease, Diagnosis	442212003	Residual cognitive deficit as late effect of cerebrovascular accident
283	Cerebrovascular Disease, Diagnosis	442668000	Hemiplegia of nondominant side as late effect of cerebrovascular disease
283	Cerebrovascular Disease, Diagnosis	442676003	Hemiplegia of dominant side as late effect of cerebrovascular disease
283	Cerebrovascular Disease, Diagnosis	87551000119101	Visual disturbance as sequela of cerebrovascular disease
283	Cerebrovascular Disease, Diagnosis	133981000119106	Dysarthria as late effects of cerebrovascular disease
284	Dementia, Diagnosis	12348006	Presenile dementia
284	Dementia, Diagnosis	52448006	Dementia
284	Dementia, Diagnosis	191449005	Uncomplicated senile dementia
284	Dementia, Diagnosis	191451009	Uncomplicated presenile dementia
284	Dementia, Diagnosis	191452002	Presenile dementia with delirium
284	Dementia, Diagnosis	191455000	Presenile dementia with depression

© 2021 Matheny ME et al. *JAMA Network Open.*

CONDITIONID	CONDITION_DESCRIPTION	SNOMED_CODE	DESCRIPTION
284	Dementia, Diagnosis	191457008	Senile dementia with depressive or paranoid features
284	Dementia, Diagnosis	191459006	Senile dementia with depression
284	Dementia, Diagnosis	191461002	Senile dementia with delirium
284	Dementia, Diagnosis	191463004	Uncomplicated arteriosclerotic dementia
284	Dementia, Diagnosis	191464005	Arteriosclerotic dementia with delirium
284	Dementia, Diagnosis	191465006	Arteriosclerotic dementia with paranoia
284	Dementia, Diagnosis	191466007	Arteriosclerotic dementia with depression
284	Dementia, Diagnosis	231438001	Presbyophrenic psychosis
284	Dementia, Diagnosis	371024007	Senile dementia with delusion
284	Dementia, Diagnosis	429998004	Vascular dementia
285	Chronic Pulmonary Disease, Diagnosis	12295008	Bronchiectasis
285	Chronic Pulmonary Disease, Diagnosis	13394002	Suberosis
285	Chronic Pulmonary Disease, Diagnosis	13645005	Chronic obstructive lung disease
285	Chronic Pulmonary Disease, Diagnosis	17996008	Pneumoconiosis due to inorganic dust
285	Chronic Pulmonary Disease, Diagnosis	18690003	Farmers' lung
285	Chronic Pulmonary Disease, Diagnosis	22607003	Asbestosis
285	Chronic Pulmonary Disease, Diagnosis	25897000	Malt-workers' lung
285	Chronic Pulmonary Disease, Diagnosis	29422001	Coal workers' pneumoconiosis
285	Chronic Pulmonary Disease, Diagnosis	31387002	Exercise-induced asthma
285	Chronic Pulmonary Disease, Diagnosis	32398004	Bronchitis
285	Chronic Pulmonary Disease, Diagnosis	37471005	Extrinsic allergic alveolitis

© 2021 Matheny ME et al. *JAMA Network Open.*

CONDITIONID	CONDITION_DESCRIPTION	SNOMED_CODE	DESCRIPTION
285	Chronic Pulmonary Disease, Diagnosis	40122008	Pneumoconiosis
285	Chronic Pulmonary Disease, Diagnosis	48347002	Humidifier lung
285	Chronic Pulmonary Disease, Diagnosis	52333004	Mushroom workers' lung
285	Chronic Pulmonary Disease, Diagnosis	57686001	Emphysematous bleb of lung
285	Chronic Pulmonary Disease, Diagnosis	63480004	Chronic bronchitis
285	Chronic Pulmonary Disease, Diagnosis	67242002	Bagassosis
285	Chronic Pulmonary Disease, Diagnosis	69339004	Bird-fanciers' lung
285	Chronic Pulmonary Disease, Diagnosis	69454006	Chronic respiratory condition due to fumes AND/OR vapors
285	Chronic Pulmonary Disease, Diagnosis	74015002	Pneumonopathy due to inhalation of dust
285	Chronic Pulmonary Disease, Diagnosis	74417001	Mucopurulent chronic bronchitis
285	Chronic Pulmonary Disease, Diagnosis	86638007	Maple-bark strippers' lung
285	Chronic Pulmonary Disease, Diagnosis	87433001	Pulmonary emphysema
285	Chronic Pulmonary Disease, Diagnosis	89549007	Catarrhal bronchitis
285	Chronic Pulmonary Disease, Diagnosis	185086009	Emphysematous bronchitis
285	Chronic Pulmonary Disease, Diagnosis	195949008	Chronic asthmatic bronchitis
285	Chronic Pulmonary Disease, Diagnosis	195951007	Acute exacerbation of chronic obstructive airways disease
285	Chronic Pulmonary Disease, Diagnosis	195967001	Asthma
285	Chronic Pulmonary Disease, Diagnosis	285381006	Acute infective exacerbation of chronic obstructive airways disease
285	Chronic Pulmonary Disease, Diagnosis	409663006	Cough variant asthma
285	Chronic Pulmonary Disease, Diagnosis	426853005	Pneumoconiosis due to silicate

© 2021 Matheny ME et al. *JAMA Network Open.*

CONDITIONID	CONDITION_DESCRIPTION	SNOMED_CODE	DESCRIPTION
285	Chronic Pulmonary Disease, Diagnosis	442025000	Acute exacerbation of chronic asthmatic bronchitis
285	Chronic Pulmonary Disease, Diagnosis	445378003	Acute exacerbation of bronchiectasis
286	Rheumatic Disease, Diagnosis	31384009	Polymyositis
286	Rheumatic Disease, Diagnosis	55464009	Systemic lupus erythematosus
286	Rheumatic Disease, Diagnosis	57160007	Felty's syndrome
286	Rheumatic Disease, Diagnosis	65323003	Polymyalgia rheumatica
286	Rheumatic Disease, Diagnosis	69896004	Rheumatoid arthritis
286	Rheumatic Disease, Diagnosis	89155008	Systemic sclerosis
286	Rheumatic Disease, Diagnosis	239793008	Rheumatoid arthritis with organ / system involvement
286	Rheumatic Disease, Diagnosis	398726004	Rheumatoid lung disease
288	Mild Liver Disease, Diagnosis	1761006	Biliary cirrhosis
288	Mild Liver Disease, Diagnosis	41889008	Chronic persistent hepatitis
288	Mild Liver Disease, Diagnosis	76783007	Chronic hepatitis
288	Mild Liver Disease, Diagnosis	266468003	Cirrhosis - non-alcoholic
288	Mild Liver Disease, Diagnosis	408335007	Autoimmune hepatitis
288	Mild Liver Disease, Diagnosis	420054005	Alcoholic cirrhosis
289	Diabetes without chronic complication, Diagnosis	44054006	Type 2 diabetes mellitus
289	Diabetes without chronic complication, Diagnosis	46635009	Type 1 diabetes mellitus
289	Diabetes without chronic complication, Diagnosis	73211009	Diabetes mellitus
289	Diabetes without chronic complication, Diagnosis	190329007	Diabetes mellitus with hyperosmolar coma
289	Diabetes without chronic complication, Diagnosis	190330002	Type 1 diabetes mellitus with

© 2021 Matheny ME et al. *JAMA Network Open.*

CONDITIONID	CONDITION_DESCRIPTION	SNOMED_CODE	DESCRIPTION
			hyperosmolar coma
289	Diabetes without chronic complication, Diagnosis	190331003	Type 2 diabetes mellitus with hyperosmolar coma
289	Diabetes without chronic complication, Diagnosis	420270002	Ketoacidosis in type 1 diabetes mellitus
289	Diabetes without chronic complication, Diagnosis	420422005	Diabetic ketoacidosis
289	Diabetes without chronic complication, Diagnosis	420662003	Coma associated with diabetes mellitus
289	Diabetes without chronic complication, Diagnosis	421365002	Peripheral circulatory disorder associated with type 1 diabetes mellitus
289	Diabetes without chronic complication, Diagnosis	421437000	Hypoglycemic coma in type 1 diabetes mellitus
289	Diabetes without chronic complication, Diagnosis	421750000	Ketoacidosis in type 2 diabetes mellitus
289	Diabetes without chronic complication, Diagnosis	421895002	Peripheral vascular disorder due to diabetes mellitus
289	Diabetes without chronic complication, Diagnosis	422166005	Peripheral circulatory disorder associated with type 2 diabetes mellitus
289	Diabetes without chronic complication, Diagnosis	428896009	Hyperosmolality due to uncontrolled type 1 diabetes mellitus
289	Diabetes without chronic complication, Diagnosis	443694000	Type II diabetes mellitus uncontrolled

© 2021 Matheny ME et al. *JAMA Network Open.*

CONDITIONID	CONDITION_DESCRIPTION	SNOMED_CODE	DESCRIPTION
289	Diabetes without chronic complication, Diagnosis	444073006	Type 1 diabetes mellitus uncontrolled
290	Diabetes with chronic complication, Diagnosis	25093002	Disorder of eye due to diabetes mellitus
290	Diabetes with chronic complication, Diagnosis	127013003	Kidney disorder due to diabetes mellitus
290	Diabetes with chronic complication, Diagnosis	420279001	Renal disorder due to type 2 diabetes mellitus
290	Diabetes with chronic complication, Diagnosis	421165007	Diabetic oculopathy associated with type 1 diabetes mellitus
290	Diabetes with chronic complication, Diagnosis	421326000	Neurological disorder with type 2 diabetes mellitus
290	Diabetes with chronic complication, Diagnosis	421468001	Neurological disorder with type 1 diabetes mellitus
290	Diabetes with chronic complication, Diagnosis	421893009	Renal disorder associated with type 1 diabetes mellitus
290	Diabetes with chronic complication, Diagnosis	422088007	Nervous system disorder due to diabetes mellitus
290	Diabetes with chronic complication, Diagnosis	422099009	Disorder of eye with type 2 diabetes mellitus
291	Hemiplegia or Paraplegia, Diagnosis	50582007	Hemiplegia
291	Hemiplegia or Paraplegia, Diagnosis	60389000	Paraplegia
291	Hemiplegia or Paraplegia, Diagnosis	79633009	Spastic hemiplegia
291	Hemiplegia or Paraplegia, Diagnosis	80935004	Flaccid hemiplegia
291	Hemiplegia or Paraplegia, Diagnosis	441717007	Hemiplegia of nondominant side

© 2021 Matheny ME et al. *JAMA Network Open.*

CONDITIONID	CONDITION_DESCRIPTION	SNOMED_CODE	DESCRIPTION
291	Hemiplegia or Paraplegia, Diagnosis	441722007	Spastic hemiplegia of nondominant side
291	Hemiplegia or Paraplegia, Diagnosis	441892008	Spastic hemiplegia of dominant side
291	Hemiplegia or Paraplegia, Diagnosis	442020005	Flaccid hemiplegia of dominant side
291	Hemiplegia or Paraplegia, Diagnosis	442077006	Flaccid hemiplegia of nondominant side
291	Hemiplegia or Paraplegia, Diagnosis	442155009	Hemiplegia of dominant side
294	Moderate or Severe Liver Disease, Diagnosis	13920009	Hepatic encephalopathy
294	Moderate or Severe Liver Disease, Diagnosis	14223005	Esophageal varices without bleeding
294	Moderate or Severe Liver Disease, Diagnosis	17709002	Bleeding esophageal varices
294	Moderate or Severe Liver Disease, Diagnosis	34742003	Portal hypertension
294	Moderate or Severe Liver Disease, Diagnosis	51292008	Hepatorenal syndrome
294	Moderate or Severe Liver Disease, Diagnosis	195475003	Esophageal varices with bleeding, associated with another disorder
294	Moderate or Severe Liver Disease, Diagnosis	195476002	Esophageal varices without bleeding, associated with another disorder
294	Moderate or Severe Liver Disease, Diagnosis	235856003	Disease of liver
295	Metastatic Solid Tumor, Diagnosis	55342001	Neoplastic disease
295	Metastatic Solid Tumor, Diagnosis	94161006	Secondary malignant neoplasm of adrenal gland
295	Metastatic Solid Tumor, Diagnosis	94222008	Secondary malignant neoplasm of bone

© 2021 Matheny ME et al. *JAMA Network Open.*

CONDITIONID	CONDITION_DESCRIPTION	SNOMED_CODE	DESCRIPTION
295	Metastatic Solid Tumor, Diagnosis	94297009	Secondary malignant neoplasm of female breast
295	Metastatic Solid Tumor, Diagnosis	94298004	Secondary malignant neoplasm of female genital organ
295	Metastatic Solid Tumor, Diagnosis	94347008	Secondary malignant neoplasm of intra-abdominal lymph nodes
295	Metastatic Solid Tumor, Diagnosis	94350006	Secondary malignant neoplasm of intrapelvic lymph nodes
295	Metastatic Solid Tumor, Diagnosis	94351005	Secondary malignant neoplasm of intrathoracic lymph nodes
295	Metastatic Solid Tumor, Diagnosis	94360002	Secondary malignant neoplasm of kidney
295	Metastatic Solid Tumor, Diagnosis	94381002	Secondary malignant neoplasm of liver
295	Metastatic Solid Tumor, Diagnosis	94391008	Secondary malignant neoplasm of lung
295	Metastatic Solid Tumor, Diagnosis	94392001	Secondary malignant neoplasm of lymph node
295	Metastatic Solid Tumor, Diagnosis	94395004	Secondary malignant neoplasm of lymph

© 2021 Matheny ME et al. *JAMA Network Open.*

CONDITIONID	CONDITION_DESCRIPTION	SNOMED_CODE	DESCRIPTION
			nodes of lower limb
295	Metastatic Solid Tumor, Diagnosis	94396003	Secondary malignant neoplasm of lymph nodes of multiple sites
295	Metastatic Solid Tumor, Diagnosis	94397007	Secondary malignant neoplasm of lymph nodes of neck
295	Metastatic Solid Tumor, Diagnosis	94398002	Secondary malignant neoplasm of lymph nodes of upper limb
295	Metastatic Solid Tumor, Diagnosis	94409002	Secondary malignant neoplasm of mediastinum
295	Metastatic Solid Tumor, Diagnosis	94442001	Secondary malignant neoplasm of nervous system
295	Metastatic Solid Tumor, Diagnosis	94455000	Secondary malignant neoplasm of ovary
295	Metastatic Solid Tumor, Diagnosis	94493005	Secondary malignant neoplasm of pleura
295	Metastatic Solid Tumor, Diagnosis	94515004	Secondary malignant neoplasm of respiratory tract
295	Metastatic Solid Tumor, Diagnosis	94579000	Secondary malignant neoplasm of skin
295	Metastatic Solid Tumor, Diagnosis	94580002	Secondary malignant neoplasm of small intestine

© 2021 Matheny ME et al. *JAMA Network Open.*

CONDITIONID	CONDITION_DESCRIPTION	SNOMED_CODE	DESCRIPTION
295	Metastatic Solid Tumor, Diagnosis	94603006	Secondary malignant neoplasm of spleen
295	Metastatic Solid Tumor, Diagnosis	94649002	Secondary malignant neoplasm of trunk
295	Metastatic Solid Tumor, Diagnosis	94663008	Secondary malignant neoplasm of urinary system
295	Metastatic Solid Tumor, Diagnosis	109356001	Primary malignant neoplasm of unspecified site
295	Metastatic Solid Tumor, Diagnosis	127232002	Neoplasm of lymph node
295	Metastatic Solid Tumor, Diagnosis	128462008	Secondary malignant neoplastic disease
295	Metastatic Solid Tumor, Diagnosis	188445006	Secondary malignant neoplasm of retroperitoneum and peritoneum
295	Metastatic Solid Tumor, Diagnosis	188462001	Secondary malignant neoplasm of brain and spinal cord
295	Metastatic Solid Tumor, Diagnosis	269473008	Secondary malignant neoplasm of respiratory and digestive systems
295	Metastatic Solid Tumor, Diagnosis	285645000	Disseminated malignancy of unknown primary
295	Metastatic Solid Tumor, Diagnosis	363346000	Malignant neoplastic disease
295	Metastatic Solid Tumor, Diagnosis	448922007	Secondary malignant neoplasm of large

© 2021 Matheny ME et al. *JAMA Network Open.*

CONDITIONID	CONDITION_DESCRIPTION	SNOMED_CODE	DESCRIPTION
			intestine and rectum
296	AIDS/HIV, Diagnosis	86406008	Human immunodeficiency virus infection
300	Obesity	44772007	Maternal obesity syndrome
300	Obesity	190966007	Extreme obesity with alveolar hypoventilation
300	Obesity	414916001	Obesity
302	Alcohol Abuse	281004	Dementia associated with alcoholism
302	Alcohol Abuse	7052005	Alcohol hallucinosis
302	Alcohol Abuse	8635005	Alcohol withdrawal delirium
302	Alcohol Abuse	15167005	Alcohol abuse
302	Alcohol Abuse	25702006	Alcohol intoxication
302	Alcohol Abuse	29212009	Alcohol-induced organic mental disorder
302	Alcohol Abuse	61144001	Alcohol-induced psychotic disorder with delusions
302	Alcohol Abuse	66590003	Alcohol dependence
302	Alcohol Abuse	73097000	Alcohol amnestic disorder
302	Alcohol Abuse	191802004	Acute alcoholic intoxication in alcoholism
302	Alcohol Abuse	191804003	Continuous acute alcoholic intoxication in alcoholism
302	Alcohol Abuse	191805002	Episodic acute alcoholic

© 2021 Matheny ME et al. *JAMA Network Open.*

CONDITIONID	CONDITION_DESCRIPTION	SNOMED_CODE	DESCRIPTION
			intoxication in alcoholism
302	Alcohol Abuse	191806001	Acute alcoholic intoxication in remission, in alcoholism
302	Alcohol Abuse	191811004	Continuous chronic alcoholism
302	Alcohol Abuse	191812006	Episodic chronic alcoholism
302	Alcohol Abuse	191813001	Chronic alcoholism in remission
302	Alcohol Abuse	191882002	Nondependent alcohol abuse, continuous
302	Alcohol Abuse	191883007	Nondependent alcohol abuse, episodic
302	Alcohol Abuse	191884001	Nondependent alcohol abuse in remission
304	Ascites, Condition or Procedure (paracentesis)	236005001	Malignant ascites
304	Ascites, Condition or Procedure (paracentesis)	389026000	Ascites
305	Cirrhosis Risk Cohort without NAFLD	1761006	Biliary cirrhosis
305	Cirrhosis Risk Cohort without NAFLD	15167005	Alcohol abuse
305	Cirrhosis Risk Cohort without NAFLD	25702006	Alcohol intoxication
305	Cirrhosis Risk Cohort without NAFLD	66590003	Alcohol dependence
305	Cirrhosis Risk Cohort without NAFLD	191802004	Acute alcoholic intoxication in alcoholism
305	Cirrhosis Risk Cohort without NAFLD	191804003	Continuous acute alcoholic intoxication in alcoholism
305	Cirrhosis Risk Cohort without NAFLD	191805002	Episodic acute alcoholic

© 2021 Matheny ME et al. *JAMA Network Open.*

CONDITIONID	CONDITION_DESCRIPTION	SNOMED_CODE	DESCRIPTION
			intoxication in alcoholism
305	Cirrhosis Risk Cohort without NAFLD	191806001	Acute alcoholic intoxication in remission, in alcoholism
305	Cirrhosis Risk Cohort without NAFLD	191811004	Continuous chronic alcoholism
305	Cirrhosis Risk Cohort without NAFLD	191812006	Episodic chronic alcoholism
305	Cirrhosis Risk Cohort without NAFLD	191813001	Chronic alcoholism in remission
305	Cirrhosis Risk Cohort without NAFLD	191882002	Nondependent alcohol abuse, continuous
305	Cirrhosis Risk Cohort without NAFLD	191883007	Nondependent alcohol abuse, episodic
305	Cirrhosis Risk Cohort without NAFLD	191884001	Nondependent alcohol abuse in remission
305	Cirrhosis Risk Cohort without NAFLD	408335007	Autoimmune hepatitis
306	Hepatorenal Syndrome	51292008	Hepatorenal syndrome
308	Varices, Condition or Procedure	195475003	Esophageal varices with bleeding, associated with another disorder
308	Varices, Condition or Procedure	195476002	Esophageal varices without bleeding, associated with another disorder
309	Hepatic Encephalopathy, (general) Condition	13920009	Hepatic encephalopathy
309	Hepatic Encephalopathy, (general) Condition	26206000	Hepatic coma due to viral hepatitis B
309	Hepatic Encephalopathy, (general) Condition	40946000	Hepatic coma due to viral hepatitis
309	Hepatic Encephalopathy, (general) Condition	186624004	Acute hepatitis B with delta agent

© 2021 Matheny ME et al. *JAMA Network Open.*

CONDITIONID	CONDITION_DESCRIPTION	SNOMED_CODE	DESCRIPTION
			(coinfection) with hepatic coma
309	Hepatic Encephalopathy, (general) Condition	186628001	Viral hepatitis C with coma
309	Hepatic Encephalopathy, (general) Condition	424340000	Hepatic coma due to chronic hepatitis B
310	Spontaneous Bacterial Peritonitis - extra general, Condition	11836002	Primary bacterial peritonitis
310	Spontaneous Bacterial Peritonitis - extra general, Condition	48661000	Peritonitis
310	Spontaneous Bacterial Peritonitis - extra general, Condition	52890006	Acute generalized peritonitis
310	Spontaneous Bacterial Peritonitis - extra general, Condition	129129003	Infectious peritonitis
310	Spontaneous Bacterial Peritonitis - extra general, Condition	235983003	Purulent peritonitis
311	Hepatocellular Carcinoma, Condition	93870000	Malignant neoplasm of liver
312	Biliary Cirrhosis	1761006	Biliary cirrhosis
313	Non-alcoholic fatty liver disease	79720007	Chronic nonalcoholic liver disease
316	Cirrhosis Risk Cohort without NAFLD	9953008	Acute alcoholic liver disease
316	Cirrhosis Risk Cohort without NAFLD	41309000	Alcoholic liver damage
316	Cirrhosis Risk Cohort without NAFLD	50325005	Alcoholic fatty liver
318	Cirrhosis Risk Cohort without NAFLD	35400008	Hereditary hemochromatosis
318	Cirrhosis Risk Cohort without NAFLD	399187006	Hemochromatosis
319	Cirrhosis Risk Cohort without NAFLD	408335007	Autoimmune hepatitis
320	Portal Hypertension	34742003	Portal hypertension
322	Spontaneous Bacterial Peritonitis - specific, Condition	11836002	Primary bacterial peritonitis
323	Hepatic Encephalopathy (specific), Condition	13920009	Hepatic encephalopathy
401	Congestive Heart Failure, Diagnosis	42343007	Congestive heart failure

© 2021 Matheny ME et al. *JAMA Network Open.*

CONDITIONID	CONDITION_DESCRIPTION	SNOMED_CODE	DESCRIPTION
401	Congestive Heart Failure, Diagnosis	82523003	Congestive rheumatic heart failure
401	Congestive Heart Failure, Diagnosis	84114007	Heart failure
401	Congestive Heart Failure, Diagnosis	85232009	Left heart failure
401	Congestive Heart Failure, Diagnosis	417996009	Systolic heart failure
401	Congestive Heart Failure, Diagnosis	418304008	Diastolic heart failure
401	Congestive Heart Failure, Diagnosis	441481004	Chronic systolic heart failure
401	Congestive Heart Failure, Diagnosis	441530006	Chronic diastolic heart failure
401	Congestive Heart Failure, Diagnosis	442304009	Combined systolic and diastolic dysfunction
401	Congestive Heart Failure, Diagnosis	443253003	Acute on chronic systolic heart failure
401	Congestive Heart Failure, Diagnosis	443254009	Acute systolic heart failure
401	Congestive Heart Failure, Diagnosis	443343001	Acute diastolic heart failure
401	Congestive Heart Failure, Diagnosis	443344007	Acute on chronic diastolic heart failure
401	Congestive Heart Failure, Diagnosis	153931000119109	Acute combined systolic and diastolic heart failure
401	Congestive Heart Failure, Diagnosis	153941000119100	Chronic combined systolic and diastolic heart failure
401	Congestive Heart Failure, Diagnosis	153951000119103	Acute on chronic combined systolic and diastolic heart failure
402	Cardiac Arrhythmias, Diagnosis	3424008	Tachycardia
402	Cardiac Arrhythmias, Diagnosis	233184008	Maintenance procedure for

© 2021 Matheny ME et al. *JAMA Network Open.*

CONDITIONID	CONDITION_DESCRIPTION	SNOMED_CODE	DESCRIPTION
			cardiac pacemaker system
402	Cardiac Arrhythmias, Diagnosis	703398004	Cardiac implant in situ
403	Valvular Disease, Diagnosis	8722008	Aortic valve disorder
403	Valvular Disease, Diagnosis	11851006	Mitral valve disorder
403	Valvular Disease, Diagnosis	56819008	Endocarditis
403	Valvular Disease, Diagnosis	67391006	Syphilitic endocarditis
403	Valvular Disease, Diagnosis	76267008	Pulmonary valve disorder
403	Valvular Disease, Diagnosis	161667004	H/O: heart valve recipient
403	Valvular Disease, Diagnosis	161677002	H/O: artificial heart valve
403	Valvular Disease, Diagnosis	194989009	Tricuspid valve disorder, non-rheumatic
403	Valvular Disease, Diagnosis	195012000	Endocarditis associated with another disorder
404	Pulmonary Circulation Disorders, Diagnosis	45650007	Kyphoscoliotic heart disease
404	Pulmonary Circulation Disorders, Diagnosis	59282003	Pulmonary embolism
404	Pulmonary Circulation Disorders, Diagnosis	87837008	Chronic pulmonary heart disease
404	Pulmonary Circulation Disorders, Diagnosis	697897003	Heritable pulmonary arterial hypertension
405	Peripheral Vascular Disorders, Diagnosis	12232008	Syphilitic aneurysm of aorta
405	Peripheral Vascular Disorders, Diagnosis	42994005	Nonruptured cerebral aneurysm
405	Peripheral Vascular Disorders, Diagnosis	68109007	Stricture of artery
405	Peripheral Vascular Disorders, Diagnosis	161678007	H/O: artificial blood vessel
406	Hypertension, uncomplicated, Diagnosis	1201005	Benign essential hypertension

© 2021 Matheny ME et al. *JAMA Network Open.*

CONDITIONID	CONDITION_DESCRIPTION	SNOMED_CODE	DESCRIPTION
406	Hypertension, uncomplicated, Diagnosis	59621000	Essential hypertension
406	Hypertension, uncomplicated, Diagnosis	78975002	Malignant essential hypertension
407	Hypertension, complicated, Diagnosis	193003	Benign hypertensive renal disease
407	Hypertension, complicated, Diagnosis	5148006	Hypertensive heart disease with congestive heart failure
407	Hypertension, complicated, Diagnosis	31992008	Secondary hypertension
407	Hypertension, complicated, Diagnosis	36221001	Benign hypertensive heart disease
407	Hypertension, complicated, Diagnosis	36315003	Malignant hypertensive heart disease without congestive heart failure
407	Hypertension, complicated, Diagnosis	38481006	Hypertensive renal disease
407	Hypertension, complicated, Diagnosis	54225002	Malignant hypertensive heart disease
407	Hypertension, complicated, Diagnosis	60899001	Hypertensive heart disease without congestive heart failure
407	Hypertension, complicated, Diagnosis	64715009	Hypertensive heart disease
407	Hypertension, complicated, Diagnosis	65443008	Malignant hypertensive renal disease
407	Hypertension, complicated, Diagnosis	66052004	Benign hypertensive heart AND renal disease
407	Hypertension, complicated, Diagnosis	66610008	Malignant hypertensive heart AND renal disease

© 2021 Matheny ME et al. *JAMA Network Open.*

CONDITIONID	CONDITION_DESCRIPTION	SNOMED_CODE	DESCRIPTION
407	Hypertension, complicated, Diagnosis	73410007	Benign secondary renovascular hypertension
407	Hypertension, complicated, Diagnosis	77737007	Benign hypertensive heart disease with congestive heart failure
407	Hypertension, complicated, Diagnosis	77970009	Benign hypertensive heart disease without congestive heart failure
407	Hypertension, complicated, Diagnosis	83105008	Malignant hypertensive heart disease with congestive heart failure
407	Hypertension, complicated, Diagnosis	86234004	Hypertensive heart AND renal disease
407	Hypertension, complicated, Diagnosis	89242004	Malignant secondary hypertension
407	Hypertension, complicated, Diagnosis	123799005	Renovascular hypertension
407	Hypertension, complicated, Diagnosis	194774006	Hypertensive renal disease with renal failure
407	Hypertension, complicated, Diagnosis	194779001	Hypertensive heart and renal disease with (congestive) heart failure
407	Hypertension, complicated, Diagnosis	194780003	Hypertensive heart and renal disease with renal failure
407	Hypertension, complicated, Diagnosis	194781004	Hypertensive heart and renal disease with both (congestive) heart failure and renal failure

© 2021 Matheny ME et al. *JAMA Network Open.*

CONDITIONID	CONDITION_DESCRIPTION	SNOMED_CODE	DESCRIPTION
407	Hypertension, complicated, Diagnosis	194783001	Malignant secondary renovascular hypertension
407	Hypertension, complicated, Diagnosis	194785008	Benign secondary hypertension
407	Hypertension, complicated, Diagnosis	285831000119108	Malignant hypertensive chronic kidney disease
407	Hypertension, complicated, Diagnosis	285841000119104	Malignant hypertensive end stage renal disease
408	Paralysis, Diagnosis	39912006	Hereditary spastic paraplegia
409	Other Neurological Disorders	15244003	Neuroleptic malignant syndrome
409	Other Neurological Disorders	52522001	Degenerative brain disorder
409	Other Neurological Disorders	60576007	Subacute combined degeneration of spinal cord
410	Chronic Pulmonary Disease	69454006	Chronic respiratory condition due to fumes AND/OR vapors
413	Hypothyroidism	3716002	Goiter
414	Renal Failure	16726004	Renal osteodystrophy
414	Renal Failure	73257006	Peritoneal dialysis catheter maintenance
414	Renal Failure	108241001	Dialysis procedure
414	Renal Failure	161665007	History of renal transplant
414	Renal Failure	251859005	Dialysis finding
414	Renal Failure	365399009	Adequacy of dialysis - finding
415	Liver Disease	161671001	H/O: liver recipient

© 2021 Matheny ME et al. *JAMA Network Open.*

CONDITIONID	CONDITION_DESCRIPTION	SNOMED_CODE	DESCRIPTION
418	Lymphoma	109989006	Multiple myeloma
418	Lymphoma	415111003	Plasma cell neoplasm
421	Rheumatoid Arthritis, Collagent vascular diseases	1961000	Chronic polyarticular juvenile rheumatoid arthritis
421	Rheumatoid Arthritis, Collagent vascular diseases	22125009	Panniculitis
421	Rheumatoid Arthritis, Collagent vascular diseases	50442003	Palindromic rheumatism
421	Rheumatoid Arthritis, Collagent vascular diseases	50581000	Goodpasture's syndrome
421	Rheumatoid Arthritis, Collagent vascular diseases	57160007	Felty's syndrome
421	Rheumatoid Arthritis, Collagent vascular diseases	58961005	Lethal midline granuloma
421	Rheumatoid Arthritis, Collagent vascular diseases	60555002	Hypersensitivity angiitis
421	Rheumatoid Arthritis, Collagent vascular diseases	62918002	Arthropathy in Behcet's syndrome
421	Rheumatoid Arthritis, Collagent vascular diseases	69896004	Rheumatoid arthritis
421	Rheumatoid Arthritis, Collagent vascular diseases	74391003	Pauciarticular juvenile rheumatoid arthritis
421	Rheumatoid Arthritis, Collagent vascular diseases	75053002	Acute febrile mucocutaneous lymph node syndrome
421	Rheumatoid Arthritis, Collagent vascular diseases	75822003	Acute polyarticular juvenile rheumatoid arthritis
421	Rheumatoid Arthritis, Collagent vascular diseases	78129009	Thrombotic thrombocytopenic purpura
421	Rheumatoid Arthritis, Collagent vascular diseases	84801008	Jaccoud's syndrome

© 2021 Matheny ME et al. *JAMA Network Open.*

CONDITIONID	CONDITION_DESCRIPTION	SNOMED_CODE	DESCRIPTION
421	Rheumatoid Arthritis, Collagent vascular diseases	85551004	Hypermobility syndrome
421	Rheumatoid Arthritis, Collagent vascular diseases	155441006	Polyarteritis nodosa
421	Rheumatoid Arthritis, Collagent vascular diseases	195353004	Granulomatosis with polyangiitis
421	Rheumatoid Arthritis, Collagent vascular diseases	201048007	Localized scleroderma
421	Rheumatoid Arthritis, Collagent vascular diseases	201799006	Monarticular juvenile rheumatoid arthritis
421	Rheumatoid Arthritis, Collagent vascular diseases	239793008	Rheumatoid arthritis with organ / system involvement
421	Rheumatoid Arthritis, Collagent vascular diseases	268006001	Muscle, ligament and fascia disorders
421	Rheumatoid Arthritis, Collagent vascular diseases	359789008	Takayasu's disease
421	Rheumatoid Arthritis, Collagent vascular diseases	398726004	Rheumatoid lung disease
421	Rheumatoid Arthritis, Collagent vascular diseases	414341000	Giant cell arteritis
421	Rheumatoid Arthritis, Collagent vascular diseases	417373000	Inflammatory polyarthropathy
422	Coagulopathy	25904003	Acquired coagulation factor deficiency
422	Coagulopathy	28293008	Hereditary factor VIII deficiency disease
422	Coagulopathy	41788008	Hereditary factor IX deficiency disease
422	Coagulopathy	49762007	Hereditary factor XI deficiency disease
422	Coagulopathy	64779008	Blood coagulation disorder

© 2021 Matheny ME et al. *JAMA Network Open.*

CONDITIONID	CONDITION_DESCRIPTION	SNOMED_CODE	DESCRIPTION
422	Coagulopathy	67406007	Disseminated intravascular coagulation
422	Coagulopathy	86075001	Coagulation factor deficiency syndrome
422	Coagulopathy	128105004	von Willebrand disorder
422	Coagulopathy	191287000	Hemorrhagic disorder due to circulating anticoagulants
423	Obesity	414916001	Obesity
424	Weigh Loss	238108007	Cachexia
424	Weigh Loss	267024001	Abnormal weight loss
425	Fluid and Electrolyte Disorder	14140009	Hyperkalemia
425	Fluid and Electrolyte Disorder	21420006	Alkalosis
425	Fluid and Electrolyte Disorder	21639008	Hypervolemia
425	Fluid and Electrolyte Disorder	28560003	Hypovolemia
425	Fluid and Electrolyte Disorder	34095006	Dehydration
425	Fluid and Electrolyte Disorder	35633007	Transfusion reaction due to excess volume
425	Fluid and Electrolyte Disorder	43339004	Hypokalemia
425	Fluid and Electrolyte Disorder	51387008	Acidosis
425	Fluid and Electrolyte Disorder	72442006	Disorder of posterior pituitary
425	Fluid and Electrolyte Disorder	76220009	Mixed acid-base balance disorder
425	Fluid and Electrolyte Disorder	76314005	Disorder of fluid AND/OR electrolyte
425	Fluid and Electrolyte Disorder	267446004	Hyperosmolality and or hypernatremia
425	Fluid and Electrolyte Disorder	267447008	Hypo-osmolality and or hyponatremia
426	Blood Loss Anemia	413533008	Anemia due to chronic blood loss

© 2021 Matheny ME et al. *JAMA Network Open.*

CONDITIONID	CONDITION_DESCRIPTION	SNOMED_CODE	DESCRIPTION
427	Deficiency Anemia	49472006	Megaloblastic anemia due to vitamin B>12< deficiency
427	Deficiency Anemia	52565000	Non megaloblastic anemia associated with nutritional deficiency
427	Deficiency Anemia	53165003	Megaloblastic anemia
427	Deficiency Anemia	84027009	Pernicious anemia
427	Deficiency Anemia	85649008	Megaloblastic anemia due to folate deficiency
427	Deficiency Anemia	191156009	Protein-deficiency anemia
427	Deficiency Anemia	267513007	Deficiency anemias
428	Alcohol Abuse	2043009	Alcoholic gastritis
428	Alcohol Abuse	7916009	Alcoholic polyneuropathy
428	Alcohol Abuse	15167005	Alcohol abuse
428	Alcohol Abuse	57346004	Toxic effect of fusel oil
428	Alcohol Abuse	67426006	Toxic effect of alcohol
428	Alcohol Abuse	82782008	Toxic effect of ethyl alcohol
428	Alcohol Abuse	83521008	Dilated cardiomyopathy secondary to alcohol
428	Alcohol Abuse	212809004	Methyl alcohol causing toxic effect
428	Alcohol Abuse	212813006	Toxic effect of isopropyl alcohol
428	Alcohol Abuse	417662000	History of clinical finding in subject
428	Alcohol Abuse	418186002	Pellagra
429	Drug Abuse	5002000	Inhalant dependence

© 2021 Matheny ME et al. *JAMA Network Open.*

CONDITIONID	CONDITION_DESCRIPTION	SNOMED_CODE	DESCRIPTION
429	Drug Abuse	25508008	Pathological drug intoxication
429	Drug Abuse	31956009	Cocaine dependence
429	Drug Abuse	38247002	Hallucinogen dependence
429	Drug Abuse	75544000	Opioid dependence
429	Drug Abuse	85005007	Cannabis dependence
429	Drug Abuse	191483003	Drug-induced psychosis
429	Drug Abuse	191486006	Drug-induced hallucinosis
429	Drug Abuse	191492000	Drug-induced delirium
429	Drug Abuse	191493005	Drug-induced dementia
429	Drug Abuse	191494004	Drug-induced amnestic syndrome
429	Drug Abuse	191496002	Drug-induced personality disorder
429	Drug Abuse	191816009	Drug dependence
429	Drug Abuse	191819002	Continuous opioid dependence
429	Drug Abuse	191820008	Episodic opioid dependence
429	Drug Abuse	191821007	Opioid dependence in remission
429	Drug Abuse	191825003	Hypnotic or anxiolytic dependence, continuous
429	Drug Abuse	191826002	Hypnotic or anxiolytic dependence, episodic
429	Drug Abuse	191827006	Hypnotic or anxiolytic

© 2021 Matheny ME et al. *JAMA Network Open.*

CONDITIONID	CONDITION_DESCRIPTION	SNOMED_CODE	DESCRIPTION
			dependence in remission
429	Drug Abuse	191831000	Cocaine dependence, continuous
429	Drug Abuse	191832007	Cocaine dependence, episodic
429	Drug Abuse	191833002	Cocaine dependence in remission
429	Drug Abuse	191837001	Cannabis dependence, continuous
429	Drug Abuse	191838006	Cannabis dependence, episodic
429	Drug Abuse	191839003	Cannabis dependence in remission
429	Drug Abuse	191843004	Amphetamine or psychostimulant dependence, continuous
429	Drug Abuse	191844005	Amphetamine or psychostimulant dependence, episodic
429	Drug Abuse	191845006	Amphetamine or psychostimulant dependence in remission
429	Drug Abuse	191849000	Hallucinogen dependence, continuous
429	Drug Abuse	191850000	Hallucinogen dependence, episodic
429	Drug Abuse	191851001	Hallucinogen dependence in remission

© 2021 Matheny ME et al. *JAMA Network Open.*

CONDITIONID	CONDITION_DESCRIPTION	SNOMED_CODE	DESCRIPTION
429	Drug Abuse	191865004	Combined opioid with other drug dependence
429	Drug Abuse	191867007	Combined opioid with other drug dependence, continuous
429	Drug Abuse	191868002	Combined opioid with other drug dependence, episodic
429	Drug Abuse	191869005	Combined opioid with other drug dependence in remission
429	Drug Abuse	191871005	Combined drug dependence, excluding opioids
429	Drug Abuse	191873008	Combined drug dependence, excluding opioid, continuous
429	Drug Abuse	191874002	Combined drug dependence, excluding opioid, episodic
429	Drug Abuse	191875001	Combined drug dependence, excluding opioid, in remission
429	Drug Abuse	268640002	Hypnotic or anxiolytic dependence
429	Drug Abuse	313071005	Substance abuse counseling
429	Drug Abuse	363101005	Drug withdrawal
429	Drug Abuse	418475009	Drug-induced sleep disorder
429	Drug Abuse	429672007	Drug-induced mood disorder

© 2021 Matheny ME et al. *JAMA Network Open.*

CONDITIONID	CONDITION_DESCRIPTION	SNOMED_CODE	DESCRIPTION
429	Drug Abuse	442351006	Mental disorder due to drug
430	Psychoses	4926007	Schizophrenia in remission
430	Psychoses	7025000	Subchronic undifferentiated schizophrenia with acute exacerbations
430	Psychoses	12939007	Chronic disorganized schizophrenia
430	Psychoses	14291003	Subchronic disorganized schizophrenia with acute exacerbations
430	Psychoses	16990005	Subchronic schizophrenia
430	Psychoses	26025008	Residual schizophrenia
430	Psychoses	27387000	Subchronic disorganized schizophrenia
430	Psychoses	29599000	Chronic undifferentiated schizophrenia
430	Psychoses	30336007	Chronic residual schizophrenia with acute exacerbations
430	Psychoses	31373002	Disorganized schizophrenia in remission
430	Psychoses	31658008	Chronic paranoid schizophrenia
430	Psychoses	35218008	Chronic disorganized schizophrenia with acute exacerbations

© 2021 Matheny ME et al. *JAMA Network Open.*

CONDITIONID	CONDITION_DESCRIPTION	SNOMED_CODE	DESCRIPTION
430	Psychoses	35252006	Disorganized schizophrenia
430	Psychoses	42868002	Subchronic catatonic schizophrenia
430	Psychoses	51133006	Residual schizophrenia in remission
430	Psychoses	58214004	Schizophrenia
430	Psychoses	63181006	Paranoid schizophrenia in remission
430	Psychoses	64905009	Paranoid schizophrenia
430	Psychoses	68890003	Schizoaffective disorder
430	Psychoses	68995007	Chronic catatonic schizophrenia
430	Psychoses	70814008	Subchronic residual schizophrenia with acute exacerbations
430	Psychoses	71103003	Chronic residual schizophrenia
430	Psychoses	76566000	Subchronic residual schizophrenia
430	Psychoses	79204003	Chronic undifferentiated schizophrenia with acute exacerbations
430	Psychoses	79866005	Subchronic paranoid schizophrenia
430	Psychoses	88975006	Schizophreniform disorder
430	Psychoses	111482003	Subchronic schizophrenia with acute exacerbations

© 2021 Matheny ME et al. *JAMA Network Open.*

CONDITIONID	CONDITION_DESCRIPTION	SNOMED_CODE	DESCRIPTION
430	Psychoses	111483008	Catatonic schizophrenia in remission
430	Psychoses	111484002	Undifferentiated schizophrenia
430	Psychoses	191527001	Simple schizophrenia
430	Psychoses	191530008	Acute exacerbation of subchronic schizophrenia
430	Psychoses	191531007	Acute exacerbation of chronic schizophrenia
430	Psychoses	191542003	Catatonic schizophrenia
430	Psychoses	191547009	Acute exacerbation of subchronic catatonic schizophrenia
430	Psychoses	191548004	Acute exacerbation of chronic catatonic schizophrenia
430	Psychoses	191554003	Acute exacerbation of subchronic paranoid schizophrenia
430	Psychoses	191555002	Acute exacerbation of chronic paranoid schizophrenia
430	Psychoses	191559008	Latent schizophrenia
430	Psychoses	191561004	Subchronic latent schizophrenia
430	Psychoses	191562006	Chronic latent schizophrenia
430	Psychoses	191563001	Acute exacerbation of subchronic latent schizophrenia

© 2021 Matheny ME et al. *JAMA Network Open.*

CONDITIONID	CONDITION_DESCRIPTION	SNOMED_CODE	DESCRIPTION
430	Psychoses	191564007	Acute exacerbation of chronic latent schizophrenia
430	Psychoses	191565008	Latent schizophrenia in remission
430	Psychoses	191567000	Schizoaffective schizophrenia
430	Psychoses	191569002	Subchronic schizoaffective schizophrenia
430	Psychoses	191570001	Chronic schizoaffective schizophrenia
430	Psychoses	191571002	Acute exacerbation of subchronic schizoaffective schizophrenia
430	Psychoses	191572009	Acute exacerbation of chronic schizoaffective schizophrenia
430	Psychoses	191574005	Schizoaffective schizophrenia in remission
430	Psychoses	231442003	Organic catatonic disorder
430	Psychoses	268617001	Acute schizophrenic episode
431	Depression	9674006	Adjustment disorder with withdrawal
431	Depression	17226007	Adjustment disorder
431	Depression	35489007	Depressive disorder
431	Depression	47372000	Adjustment disorder with anxious mood

© 2021 Matheny ME et al. *JAMA Network Open.*

CONDITIONID	CONDITION_DESCRIPTION	SNOMED_CODE	DESCRIPTION
431	Depression	47505003	Posttraumatic stress disorder
431	Depression	55668003	Adjustment disorder with mixed emotional features
431	Depression	57194009	Adjustment disorder with depressed mood
431	Depression	66381006	Adjustment disorder with mixed disturbance of emotions AND conduct
431	Depression	78667006	Dysthymia
431	Depression	84984002	Adjustment disorder with disturbance of conduct
431	Depression	126943008	Separation anxiety
431	Depression	192049004	Prolonged depressive adjustment reaction
431	Depression	192051000	Adolescent emancipation disorder
431	Depression	192063005	Adjustment reaction with physical symptoms
431	Depression	268658008	Specific academic or work inhibition
601	QN_AMI_METCA_ALL	55342001	Neoplastic disease
601	QN_AMI_METCA_ALL	91854005	Acute leukemia in remission
601	QN_AMI_METCA_ALL	91855006	Acute leukemia
601	QN_AMI_METCA_ALL	91856007	Acute lymphoid leukemia in remission
601	QN_AMI_METCA_ALL	91857003	Acute lymphoid leukemia

© 2021 Matheny ME et al. *JAMA Network Open.*

CONDITIONID	CONDITION_DESCRIPTION	SNOMED_CODE	DESCRIPTION
601	QN_AMI_METCA_ALL	91858008	Acute monocytic leukemia in remission
601	QN_AMI_METCA_ALL	91860005	Acute myeloid leukemia in remission
601	QN_AMI_METCA_ALL	91861009	Acute myeloid leukemia, disease
601	QN_AMI_METCA_ALL	93450001	Erythroleukemia in remission
601	QN_AMI_METCA_ALL	93451002	Erythroleukemia, FAB M6
601	QN_AMI_METCA_ALL	94161006	Secondary malignant neoplasm of adrenal gland
601	QN_AMI_METCA_ALL	94186002	Secondary malignant neoplasm of bladder
601	QN_AMI_METCA_ALL	94217008	Secondary malignant neoplasm of bone marrow
601	QN_AMI_METCA_ALL	94222008	Secondary malignant neoplasm of bone
601	QN_AMI_METCA_ALL	94225005	Secondary malignant neoplasm of brain
601	QN_AMI_METCA_ALL	94246001	Secondary malignant neoplasm of cerebral meninges
601	QN_AMI_METCA_ALL	94347008	Secondary malignant neoplasm of intra-abdominal lymph nodes
601	QN_AMI_METCA_ALL	94350006	Secondary malignant

© 2021 Matheny ME et al. *JAMA Network Open.*

CONDITIONID	CONDITION_DESCRIPTION	SNOMED_CODE	DESCRIPTION
			neoplasm of intrapelvic lymph nodes
601	QN_AMI_METCA_ALL	94351005	Secondary malignant neoplasm of intrathoracic lymph nodes
601	QN_AMI_METCA_ALL	94360002	Secondary malignant neoplasm of kidney
601	QN_AMI_METCA_ALL	94381002	Secondary malignant neoplasm of liver
601	QN_AMI_METCA_ALL	94391008	Secondary malignant neoplasm of lung
601	QN_AMI_METCA_ALL	94395004	Secondary malignant neoplasm of lymph nodes of lower limb
601	QN_AMI_METCA_ALL	94396003	Secondary malignant neoplasm of lymph nodes of multiple sites
601	QN_AMI_METCA_ALL	94409002	Secondary malignant neoplasm of mediastinum
601	QN_AMI_METCA_ALL	94442001	Secondary malignant neoplasm of nervous system
601	QN_AMI_METCA_ALL	94455000	Secondary malignant neoplasm of ovary
601	QN_AMI_METCA_ALL	94493005	Secondary malignant neoplasm of pleura

© 2021 Matheny ME et al. *JAMA Network Open.*

CONDITIONID	CONDITION_DESCRIPTION	SNOMED_CODE	DESCRIPTION
601	QN_AMI_METCA_ALL	94515004	Secondary malignant neoplasm of respiratory tract
601	QN_AMI_METCA_ALL	94580002	Secondary malignant neoplasm of small intestine
601	QN_AMI_METCA_ALL	94603006	Secondary malignant neoplasm of spleen
601	QN_AMI_METCA_ALL	94663008	Secondary malignant neoplasm of urinary system
601	QN_AMI_METCA_ALL	109991003	Acute panmyelosis with myelofibrosis
601	QN_AMI_METCA_ALL	110004001	Acute promyelocytic leukemia, FAB M3
601	QN_AMI_METCA_ALL	127232002	Neoplasm of lymph node
601	QN_AMI_METCA_ALL	128462008	Secondary malignant neoplastic disease
601	QN_AMI_METCA_ALL	188445006	Secondary malignant neoplasm of retroperitoneum and peritoneum
601	QN_AMI_METCA_ALL	188462001	Secondary malignant neoplasm of brain and spinal cord
601	QN_AMI_METCA_ALL	188725004	Lymphoid leukemia
601	QN_AMI_METCA_ALL	188732008	Myeloid leukemia
601	QN_AMI_METCA_ALL	188744006	Monocytic leukemia
601	QN_AMI_METCA_ALL	253001006	Merkel cell carcinoma

© 2021 Matheny ME et al. *JAMA Network Open.*

CONDITIONID	CONDITION_DESCRIPTION	SNOMED_CODE	DESCRIPTION
601	QN_AMI_METCA_ALL	255046005	Neuroendocrine tumor
601	QN_AMI_METCA_ALL	269473008	Secondary malignant neoplasm of respiratory and digestive systems
601	QN_AMI_METCA_ALL	274088005	Secondary malignant neoplasm of unknown site
601	QN_AMI_METCA_ALL	277602003	Acute megakaryoblastic leukemia
601	QN_AMI_METCA_ALL	285645000	Disseminated malignancy of unknown primary
601	QN_AMI_METCA_ALL	303194003	Metastasis to head and neck lymph node
601	QN_AMI_METCA_ALL	369523007	Secondary malignant neoplasm of left ovary
601	QN_AMI_METCA_ALL	369530001	Secondary malignant neoplasm of right ovary
601	QN_AMI_METCA_ALL	413441006	Acute monocytic leukemia
601	QN_AMI_METCA_ALL	413442004	Acute monocytic/monoblastic leukemia
601	QN_AMI_METCA_ALL	425869007	Acute promyelocytic leukemia, FAB M3, in remission
601	QN_AMI_METCA_ALL	443492008	Carcinoid tumor
601	QN_AMI_METCA_ALL	444911000	Acute myeloid leukemia with

© 2021 Matheny ME et al. *JAMA Network Open.*

CONDITIONID	CONDITION_DESCRIPTION	SNOMED_CODE	DESCRIPTION
			t(9:11)(p22;q23); MLLT3-MLL
601	QN_AMI_METCA_ALL	445448008	Acute myeloid leukemia with myelodysplasia-related changes
601	QN_AMI_METCA_ALL	448922007	Secondary malignant neoplasm of large intestine and rectum
601	QN_AMI_METCA_ALL	813671000000107	Secondary malignant neoplasm of liver and intrahepatic bile duct
602	QN_AMI_LUNG_OTHER_CA	92817004	Chronic myeloid leukemia in remission
602	QN_AMI_LUNG_OTHER_CA	92818009	Chronic myeloid leukemia
602	QN_AMI_LUNG_OTHER_CA	93142004	Leukemia in remission
602	QN_AMI_LUNG_OTHER_CA	93143009	Leukemia
602	QN_AMI_LUNG_OTHER_CA	93818001	Primary malignant neoplasm of greater curvature of stomach
602	QN_AMI_LUNG_OTHER_CA	93846004	Primary malignant neoplasm of jejunum
602	QN_AMI_LUNG_OTHER_CA	93867004	Primary malignant neoplasm of lesser curvature of stomach
602	QN_AMI_LUNG_OTHER_CA	93870000	Malignant neoplasm of liver
602	QN_AMI_LUNG_OTHER_CA	93966009	Primary malignant neoplasm of pleura

© 2021 Matheny ME et al. *JAMA Network Open.*

CONDITIONID	CONDITION_DESCRIPTION	SNOMED_CODE	DESCRIPTION
602	QN_AMI_LUNG_OTHER_CA	93976007	Primary malignant neoplasm of pyloric antrum
602	QN_AMI_LUNG_OTHER_CA	93977003	Primary malignant neoplasm of pylorus
602	QN_AMI_LUNG_OTHER_CA	93986008	Primary malignant neoplasm of respiratory tract
602	QN_AMI_LUNG_OTHER_CA	94048009	Primary malignant neoplasm of small intestine
602	QN_AMI_LUNG_OTHER_CA	94148006	Megakaryocytic leukemia in remission
602	QN_AMI_LUNG_OTHER_CA	94704006	Multiple myeloma in remission
602	QN_AMI_LUNG_OTHER_CA	94716000	Myeloid leukemia in remission
602	QN_AMI_LUNG_OTHER_CA	94718004	Myeloid sarcoma in remission
602	QN_AMI_LUNG_OTHER_CA	94719007	Myeloid sarcoma
602	QN_AMI_LUNG_OTHER_CA	95209008	Plasma cell leukemia in remission
602	QN_AMI_LUNG_OTHER_CA	95210003	Plasma cell leukemia
602	QN_AMI_LUNG_OTHER_CA	95214007	Primary malignant neoplasm of liver
602	QN_AMI_LUNG_OTHER_CA	109357005	Primary malignant neoplasm of ill-defined site
602	QN_AMI_LUNG_OTHER_CA	109371002	Overlapping malignant neoplasm of bronchus and lung
602	QN_AMI_LUNG_OTHER_CA	109374005	Overlapping malignant neoplasm of mediastinum and pleura

© 2021 Matheny ME et al. *JAMA Network Open.*

CONDITIONID	CONDITION_DESCRIPTION	SNOMED_CODE	DESCRIPTION
602	QN_AMI_LUNG_OTHER_CA	109378008	Mesothelioma
602	QN_AMI_LUNG_OTHER_CA	109383000	Malignant mesothelioma of pericardium
602	QN_AMI_LUNG_OTHER_CA	109835005	Overlapping malignant neoplasm of esophagus
602	QN_AMI_LUNG_OTHER_CA	109836006	Overlapping malignant neoplasm of stomach
602	QN_AMI_LUNG_OTHER_CA	109837002	Overlapping malignant neoplasm of small intestine
602	QN_AMI_LUNG_OTHER_CA	109841003	Liver cell carcinoma
602	QN_AMI_LUNG_OTHER_CA	109842005	Intrahepatic bile duct carcinoma
602	QN_AMI_LUNG_OTHER_CA	109843000	Hepatoblastoma
602	QN_AMI_LUNG_OTHER_CA	109844006	Angiosarcoma of liver
602	QN_AMI_LUNG_OTHER_CA	109847004	Overlapping malignant neoplasm of biliary tract
602	QN_AMI_LUNG_OTHER_CA	109848009	Overlapping malignant neoplasm of pancreas
602	QN_AMI_LUNG_OTHER_CA	109851002	Overlapping malignant neoplasm of retroperitoneum and peritoneum
602	QN_AMI_LUNG_OTHER_CA	109853004	Mesothelioma of peritoneum
602	QN_AMI_LUNG_OTHER_CA	109989006	Multiple myeloma
602	QN_AMI_LUNG_OTHER_CA	109992005	Polycythemia vera
602	QN_AMI_LUNG_OTHER_CA	110002002	Mast cell leukemia

© 2021 Matheny ME et al. *JAMA Network Open.*

CONDITIONID	CONDITION_DESCRIPTION	SNOMED_CODE	DESCRIPTION
602	QN_AMI_LUNG_OTHER_CA	126855001	Neoplasm of extrahepatic bile ducts
602	QN_AMI_LUNG_OTHER_CA	127225006	Chronic myelomonocytic leukemia
602	QN_AMI_LUNG_OTHER_CA	187723009	Malignant tumor of thoracic part of esophagus
602	QN_AMI_LUNG_OTHER_CA	187724003	Malignant tumor of abdominal part of esophagus
602	QN_AMI_LUNG_OTHER_CA	187725002	Malignant tumor of upper third of esophagus
602	QN_AMI_LUNG_OTHER_CA	187726001	Malignant tumor of middle third of esophagus
602	QN_AMI_LUNG_OTHER_CA	187727005	Malignant tumor of lower third of esophagus
602	QN_AMI_LUNG_OTHER_CA	187732006	Malignant tumor of cardia
602	QN_AMI_LUNG_OTHER_CA	187736009	Malignant tumor of pylorus
602	QN_AMI_LUNG_OTHER_CA	187740000	Malignant tumor of pyloric antrum
602	QN_AMI_LUNG_OTHER_CA	187741001	Malignant tumor of fundus of stomach
602	QN_AMI_LUNG_OTHER_CA	187742008	Malignant tumor of body of stomach
602	QN_AMI_LUNG_OTHER_CA	187752007	Malignant tumor of Meckel's diverticulum
602	QN_AMI_LUNG_OTHER_CA	187767006	Malignant neoplasm of liver and intrahepatic bile ducts
602	QN_AMI_LUNG_OTHER_CA	187791002	Malignant tumor of body of pancreas

© 2021 Matheny ME et al. *JAMA Network Open.*

CONDITIONID	CONDITION_DESCRIPTION	SNOMED_CODE	DESCRIPTION
602	QN_AMI_LUNG_OTHER_CA	187792009	Malignant tumor of tail of pancreas
602	QN_AMI_LUNG_OTHER_CA	187793004	Malignant tumor of pancreatic duct
602	QN_AMI_LUNG_OTHER_CA	187794005	Malignant tumor of Islets of Langerhans
602	QN_AMI_LUNG_OTHER_CA	187801002	Malignant tumor of peritoneum and retroperitoneum
602	QN_AMI_LUNG_OTHER_CA	187808008	Malignant neoplasm of specified parts of peritoneum
602	QN_AMI_LUNG_OTHER_CA	187864008	Malignant neoplasm of middle lobe, bronchus or lung
602	QN_AMI_LUNG_OTHER_CA	187868006	Malignant neoplasm of lower lobe, bronchus or lung
602	QN_AMI_LUNG_OTHER_CA	188718006	Extramedullary plasmacytoma
602	QN_AMI_LUNG_OTHER_CA	188732008	Myeloid leukemia
602	QN_AMI_LUNG_OTHER_CA	188736006	Subacute myeloid leukemia
602	QN_AMI_LUNG_OTHER_CA	188744006	Monocytic leukemia
602	QN_AMI_LUNG_OTHER_CA	188745007	Chronic monocytic leukemia
602	QN_AMI_LUNG_OTHER_CA	188746008	Subacute monocytic leukemia
602	QN_AMI_LUNG_OTHER_CA	188754005	Megakaryocytic leukemia
602	QN_AMI_LUNG_OTHER_CA	254601002	Sarcoma of liver
602	QN_AMI_LUNG_OTHER_CA	254611009	Malignant tumor of endocrine pancreas

© 2021 Matheny ME et al. *JAMA Network Open.*

CONDITIONID	CONDITION_DESCRIPTION	SNOMED_CODE	DESCRIPTION
602	QN_AMI_LUNG_OTHER_CA	254645002	Malignant mesothelioma of pleura
602	QN_AMI_LUNG_OTHER_CA	269459004	Malignant tumor of lesser curve of stomach
602	QN_AMI_LUNG_OTHER_CA	269460009	Malignant tumor of greater curve of stomach
602	QN_AMI_LUNG_OTHER_CA	269464000	Malignant neoplasm of upper lobe, bronchus or lung
602	QN_AMI_LUNG_OTHER_CA	275524009	Immunoproliferative neoplasm
602	QN_AMI_LUNG_OTHER_CA	277589003	Atypical chronic myeloid leukemia
602	QN_AMI_LUNG_OTHER_CA	363349007	Malignant tumor of stomach
602	QN_AMI_LUNG_OTHER_CA	363353009	Malignant tumor of gallbladder
602	QN_AMI_LUNG_OTHER_CA	363358000	Malignant tumor of lung
602	QN_AMI_LUNG_OTHER_CA	363402007	Malignant tumor of esophagus
602	QN_AMI_LUNG_OTHER_CA	363403002	Malignant tumor of duodenum
602	QN_AMI_LUNG_OTHER_CA	363404008	Malignant tumor of jejunum
602	QN_AMI_LUNG_OTHER_CA	363405009	Malignant tumor of ileum
602	QN_AMI_LUNG_OTHER_CA	363415003	Malignant tumor of biliary tract
602	QN_AMI_LUNG_OTHER_CA	363417006	Malignant tumor of ampulla of Vater
602	QN_AMI_LUNG_OTHER_CA	363418001	Malignant tumor of pancreas
602	QN_AMI_LUNG_OTHER_CA	363419009	Malignant tumor of head of pancreas

© 2021 Matheny ME et al. *JAMA Network Open.*

CONDITIONID	CONDITION_DESCRIPTION	SNOMED_CODE	DESCRIPTION
602	QN_AMI_LUNG_OTHER_CA	363420003	Malignant retroperitoneal tumor
602	QN_AMI_LUNG_OTHER_CA	363432004	Malignant tumor of trachea
602	QN_AMI_LUNG_OTHER_CA	363433009	Malignant tumor of pleura
602	QN_AMI_LUNG_OTHER_CA	363492001	Malignant tumor of peritoneum
602	QN_AMI_LUNG_OTHER_CA	363509000	Malignant tumor of small intestine
602	QN_AMI_LUNG_OTHER_CA	371967001	Primary malignant neoplasm of ampulla of Vater
602	QN_AMI_LUNG_OTHER_CA	371970002	Primary malignant neoplasm of biliary tract
602	QN_AMI_LUNG_OTHER_CA	371984007	Primary malignant neoplasm of esophagus
602	QN_AMI_LUNG_OTHER_CA	372003004	Primary malignant neoplasm of pancreas
602	QN_AMI_LUNG_OTHER_CA	372014001	Primary malignant neoplasm of stomach
602	QN_AMI_LUNG_OTHER_CA	372016004	Primary malignant neoplasm of the peritoneum
602	QN_AMI_LUNG_OTHER_CA	372065009	Malignant neoplasm of main bronchus
602	QN_AMI_LUNG_OTHER_CA	415111003	Plasma cell neoplasm
602	QN_AMI_LUNG_OTHER_CA	415287001	Relapsing chronic myeloid leukemia
602	QN_AMI_LUNG_OTHER_CA	445227008	Juvenile myelomonocytic leukemia
602	QN_AMI_LUNG_OTHER_CA	446189008	Primary malignant neoplasm of

© 2021 Matheny ME et al. *JAMA Network Open.*

CONDITIONID	CONDITION_DESCRIPTION	SNOMED_CODE	DESCRIPTION
			extrahepatic bile duct
602	QN_AMI_LUNG_OTHER_CA	447109003	Primary malignant neoplasm of intrahepatic bile duct
602	QN_AMI_LUNG_OTHER_CA	449308006	Malignant neoplasm of visceral pleura
610	QN_AMI_DM_W_ACUTE_COMP	5368009	Drug-induced diabetes mellitus
610	QN_AMI_DM_W_ACUTE_COMP	8801005	Secondary diabetes mellitus
610	QN_AMI_DM_W_ACUTE_COMP	20313009	Hyperosmolality
610	QN_AMI_DM_W_ACUTE_COMP	26298008	Diabetic coma with ketoacidosis
610	QN_AMI_DM_W_ACUTE_COMP	73211009	Diabetes mellitus
610	QN_AMI_DM_W_ACUTE_COMP	111556005	Diabetic ketoacidosis without coma
610	QN_AMI_DM_W_ACUTE_COMP	190329007	Diabetes mellitus with hyperosmolar coma
610	QN_AMI_DM_W_ACUTE_COMP	190330002	Type 1 diabetes mellitus with hyperosmolar coma
610	QN_AMI_DM_W_ACUTE_COMP	190331003	Type 2 diabetes mellitus with hyperosmolar coma
610	QN_AMI_DM_W_ACUTE_COMP	267384006	Hypoglycemic coma
610	QN_AMI_DM_W_ACUTE_COMP	302866003	Hypoglycemia
610	QN_AMI_DM_W_ACUTE_COMP	310505005	Diabetic hyperosmolar non-ketotic state
610	QN_AMI_DM_W_ACUTE_COMP	420270002	Ketoacidosis in type 1 diabetes mellitus

© 2021 Matheny ME et al. *JAMA Network Open.*

CONDITIONID	CONDITION_DESCRIPTION	SNOMED_CODE	DESCRIPTION
610	QN_AMI_DM_W_ACUTE_COMP	420422005	Diabetic ketoacidosis
610	QN_AMI_DM_W_ACUTE_COMP	420662003	Coma associated with diabetes mellitus
610	QN_AMI_DM_W_ACUTE_COMP	421075007	Ketoacidotic coma in type 1 diabetes mellitus
610	QN_AMI_DM_W_ACUTE_COMP	421437000	Hypoglycemic coma in type 1 diabetes mellitus
610	QN_AMI_DM_W_ACUTE_COMP	421725003	Hypoglycemic coma in diabetes mellitus
610	QN_AMI_DM_W_ACUTE_COMP	421750000	Ketoacidosis in type 2 diabetes mellitus
610	QN_AMI_DM_W_ACUTE_COMP	428896009	Hyperosmolality due to uncontrolled type 1 diabetes mellitus
610	QN_AMI_DM_W_ACUTE_COMP	719216001	Hypoglycemic coma co-occurrent and due to diabetes mellitus type II
611	QN_AMI_DM_W_CHR_COMP	2556008	Periodontal disease
611	QN_AMI_DM_W_CHR_COMP	4855003	Retinopathy due to diabetes mellitus
611	QN_AMI_DM_W_CHR_COMP	5368009	Drug-induced diabetes mellitus
611	QN_AMI_DM_W_CHR_COMP	8801005	Secondary diabetes mellitus
611	QN_AMI_DM_W_CHR_COMP	25093002	Disorder of eye due to diabetes mellitus
611	QN_AMI_DM_W_CHR_COMP	39058009	Diabetic lumbosacral radiculoplexus neuropathy

© 2021 Matheny ME et al. *JAMA Network Open.*

CONDITIONID	CONDITION_DESCRIPTION	SNOMED_CODE	DESCRIPTION
611	QN_AMI_DM_W_CHR_COMP	43959009	Cataract due to diabetes mellitus
611	QN_AMI_DM_W_CHR_COMP	44054006	Type 2 diabetes mellitus
611	QN_AMI_DM_W_CHR_COMP	46635009	Type 1 diabetes mellitus
611	QN_AMI_DM_W_CHR_COMP	49455004	Diabetic polyneuropathy
611	QN_AMI_DM_W_CHR_COMP	50620007	Diabetic autonomic neuropathy
611	QN_AMI_DM_W_CHR_COMP	59276001	Proliferative retinopathy with diabetes mellitus
611	QN_AMI_DM_W_CHR_COMP	73211009	Diabetes mellitus
611	QN_AMI_DM_W_CHR_COMP	74627003	Diabetic complication
611	QN_AMI_DM_W_CHR_COMP	80394007	Hyperglycemia
611	QN_AMI_DM_W_CHR_COMP	127013003	Kidney disorder due to diabetes mellitus
611	QN_AMI_DM_W_CHR_COMP	127014009	Peripheral angiopathy due to diabetes mellitus
611	QN_AMI_DM_W_CHR_COMP	190368000	Type 1 diabetes mellitus with ulcer
611	QN_AMI_DM_W_CHR_COMP	190389009	Type 2 diabetes mellitus with ulcer
611	QN_AMI_DM_W_CHR_COMP	190448007	Drug-induced hypoglycemia without coma
611	QN_AMI_DM_W_CHR_COMP	201724008	Neuropathic arthropathy due to diabetes mellitus
611	QN_AMI_DM_W_CHR_COMP	230572002	Diabetic neuropathy
611	QN_AMI_DM_W_CHR_COMP	230577008	Diabetic mononeuropathy
611	QN_AMI_DM_W_CHR_COMP	237633009	Hypoglycemic state due to diabetes mellitus
611	QN_AMI_DM_W_CHR_COMP	302866003	Hypoglycemia

© 2021 Matheny ME et al. *JAMA Network Open.*

CONDITIONID	CONDITION_DESCRIPTION	SNOMED_CODE	DESCRIPTION
611	QN_AMI_DM_W_CHR_COMP	312903003	Mild nonproliferative diabetic retinopathy
611	QN_AMI_DM_W_CHR_COMP	312904009	Moderate nonproliferative diabetic retinopathy
611	QN_AMI_DM_W_CHR_COMP	312905005	Severe nonproliferative diabetic retinopathy
611	QN_AMI_DM_W_CHR_COMP	312912001	Macular edema due to diabetes mellitus
611	QN_AMI_DM_W_CHR_COMP	314893005	Type 1 diabetes mellitus with arthropathy
611	QN_AMI_DM_W_CHR_COMP	314902007	Type 2 diabetes mellitus with peripheral angiopathy
611	QN_AMI_DM_W_CHR_COMP	314903002	Type 2 diabetes mellitus with arthropathy
611	QN_AMI_DM_W_CHR_COMP	314904008	Type 2 diabetes mellitus with neuropathic arthropathy
611	QN_AMI_DM_W_CHR_COMP	359611005	Diabetic neuropathy with neurologic complication
611	QN_AMI_DM_W_CHR_COMP	360546002	Hypoglycemic shock
611	QN_AMI_DM_W_CHR_COMP	371087003	Diabetic foot ulcer
611	QN_AMI_DM_W_CHR_COMP	372070002	Gangrenous disorder
611	QN_AMI_DM_W_CHR_COMP	390834004	Nonproliferative diabetic retinopathy

© 2021 Matheny ME et al. *JAMA Network Open.*

CONDITIONID	CONDITION_DESCRIPTION	SNOMED_CODE	DESCRIPTION
611	QN_AMI_DM_W_CHR_COMP	399269003	Arthropathy
611	QN_AMI_DM_W_CHR_COMP	399872003	Severe nonproliferative diabetic retinopathy with clinically significant macular edema
611	QN_AMI_DM_W_CHR_COMP	399873008	Severe nonproliferative diabetic retinopathy with no macular edema
611	QN_AMI_DM_W_CHR_COMP	420279001	Renal disorder due to type 2 diabetes mellitus
611	QN_AMI_DM_W_CHR_COMP	420436000	Mononeuropathy with type 2 diabetes mellitus
611	QN_AMI_DM_W_CHR_COMP	420756003	Cataract due to diabetes mellitus type 2
611	QN_AMI_DM_W_CHR_COMP	420789003	Retinopathy with type 1 diabetes mellitus
611	QN_AMI_DM_W_CHR_COMP	420868002	Disorder due to type 1 diabetes mellitus
611	QN_AMI_DM_W_CHR_COMP	420918009	Mononeuropathy with type 1 diabetes mellitus
611	QN_AMI_DM_W_CHR_COMP	421165007	Diabetic oculopathy associated with type 1 diabetes mellitus
611	QN_AMI_DM_W_CHR_COMP	421326000	Neurological disorder with type 2 diabetes mellitus
611	QN_AMI_DM_W_CHR_COMP	421365002	Peripheral circulatory disorder associated with

© 2021 Matheny ME et al. *JAMA Network Open.*

CONDITIONID	CONDITION_DESCRIPTION	SNOMED_CODE	DESCRIPTION
			type 1 diabetes mellitus
611	QN_AMI_DM_W_CHR_COMP	421468001	Neurological disorder with type 1 diabetes mellitus
611	QN_AMI_DM_W_CHR_COMP	421893009	Renal disorder associated with type 1 diabetes mellitus
611	QN_AMI_DM_W_CHR_COMP	421895002	Peripheral vascular disorder due to diabetes mellitus
611	QN_AMI_DM_W_CHR_COMP	421920002	Cataract due to diabetes mellitus type 1
611	QN_AMI_DM_W_CHR_COMP	422014003	Disorder due to type 2 diabetes mellitus
611	QN_AMI_DM_W_CHR_COMP	422088007	Nervous system disorder due to diabetes mellitus
611	QN_AMI_DM_W_CHR_COMP	422099009	Disorder of eye with type 2 diabetes mellitus
611	QN_AMI_DM_W_CHR_COMP	422166005	Peripheral circulatory disorder associated with type 2 diabetes mellitus
611	QN_AMI_DM_W_CHR_COMP	422183001	Diabetic skin ulcer
611	QN_AMI_DM_W_CHR_COMP	427027005	Lumbosacral radiculoplexus neuropathy with type 2 diabetes mellitus
611	QN_AMI_DM_W_CHR_COMP	427571000	Lumbosacral radiculoplexus neuropathy with type 1 diabetes mellitus

© 2021 Matheny ME et al. *JAMA Network Open.*

CONDITIONID	CONDITION_DESCRIPTION	SNOMED_CODE	DESCRIPTION
611	QN_AMI_DM_W_CHR_COMP	441690002	Drug-induced hyperglycemia
611	QN_AMI_DM_W_CHR_COMP	712882000	Autonomic neuropathy with type 1 diabetes mellitus
611	QN_AMI_DM_W_CHR_COMP	712883005	Autonomic neuropathy with type 2 diabetes mellitus
611	QN_AMI_DM_W_CHR_COMP	713705003	Polyneuropathy due to type 1 diabetes mellitus
611	QN_AMI_DM_W_CHR_COMP	713706002	Polyneuropathy due to type 2 diabetes mellitus
611	QN_AMI_DM_W_CHR_COMP	771000119108	Chronic kidney disease due to type 2 diabetes mellitus
611	QN_AMI_DM_W_CHR_COMP	60961000119107	Nonproliferative diabetic retinopathy due to type 1 diabetes mellitus
611	QN_AMI_DM_W_CHR_COMP	60971000119101	Proliferative retinopathy with type 1 diabetes mellitus
611	QN_AMI_DM_W_CHR_COMP	71771000119100	Neuropathic arthropathy due to type 1 diabetes mellitus
611	QN_AMI_DM_W_CHR_COMP	84371000119108	Hypoglycemia due to type 1 diabetes mellitus
611	QN_AMI_DM_W_CHR_COMP	97331000119101	Macular edema and retinopathy due to type 2 diabetes mellitus

© 2021 Matheny ME et al. *JAMA Network Open.*

CONDITIONID	CONDITION_DESCRIPTION	SNOMED_CODE	DESCRIPTION
611	QN_AMI_DM_W_CHR_COMP	120731000119103	Hypoglycemia due to type 2 diabetes mellitus
611	QN_AMI_DM_W_CHR_COMP	138911000119106	Mild nonproliferative retinopathy due to type 2 diabetes mellitus
611	QN_AMI_DM_W_CHR_COMP	138921000119104	Moderate nonproliferative retinopathy due to type 2 diabetes mellitus
612	QN_AMI_DM_WO_COMP	5368009	Drug-induced diabetes mellitus
612	QN_AMI_DM_WO_COMP	8801005	Secondary diabetes mellitus
612	QN_AMI_DM_WO_COMP	44054006	Type 2 diabetes mellitus
612	QN_AMI_DM_WO_COMP	46635009	Type 1 diabetes mellitus
612	QN_AMI_DM_WO_COMP	73211009	Diabetes mellitus
612	QN_AMI_DM_WO_COMP	111552007	Diabetes mellitus without complication
612	QN_AMI_DM_WO_COMP	170747006	Diabetic on insulin
612	QN_AMI_DM_WO_COMP	313435000	Type 1 diabetes mellitus without complication
612	QN_AMI_DM_WO_COMP	313436004	Type 2 diabetes mellitus without complication
612	QN_AMI_DM_WO_COMP	443694000	Type II diabetes mellitus uncontrolled
612	QN_AMI_DM_WO_COMP	444073006	Type 1 diabetes mellitus uncontrolled
612	QN_AMI_DM_WO_COMP	710815001	Long-term current use of insulin

© 2021 Matheny ME et al. *JAMA Network Open.*

CONDITIONID	CONDITION_DESCRIPTION	SNOMED_CODE	DESCRIPTION
614	QN_AMI_MALNUTR	29740003	Nutritional marasmus
614	QN_AMI_MALNUTR	58262005	Kwashiorkor
614	QN_AMI_MALNUTR	74257000	Arrested development following protein-calorie malnutrition
614	QN_AMI_MALNUTR	77702009	Malnutrition of mild degree
614	QN_AMI_MALNUTR	190602008	Moderate protein-calorie malnutrition
614	QN_AMI_MALNUTR	190603003	Mild protein-calorie malnutrition
614	QN_AMI_MALNUTR	190675006	Sequelae of protein-energy malnutrition
614	QN_AMI_MALNUTR	238107002	Deficiency of macronutrients
614	QN_AMI_MALNUTR	238108007	Cachexia
614	QN_AMI_MALNUTR	238108007	Cachexia
614	QN_AMI_MALNUTR	238109004	Marasmic kwashiorkor
614	QN_AMI_MALNUTR	272588001	Malnutrition
614	QN_AMI_MALNUTR	360549009	Severe protein-calorie malnutrition
615	QN_AMI_MOR_OBES	162864005	Body mass index 30+ - obesity
615	QN_AMI_MOR_OBES	190966007	Extreme obesity with alveolar hypoventilation
615	QN_AMI_MOR_OBES	238136002	Morbid obesity
615	QN_AMI_MOR_OBES	408512008	Body mass index 40+ - severely obese
616	QN_AMI_OTHER_ENDO_METAB	626004	Hypercortisolism due to nonpituitary tumor

© 2021 Matheny ME et al. *JAMA Network Open.*

CONDITIONID	CONDITION_DESCRIPTION	SNOMED_CODE	DESCRIPTION
616	QN_AMI_OTHER_ENDO_METAB	1074009	Glucocorticoid-responsive primary hyperaldosteronism
616	QN_AMI_OTHER_ENDO_METAB	5181007	Disorder of tryptophan metabolism
616	QN_AMI_OTHER_ENDO_METAB	6483008	Tyrosinase-negative oculocutaneous albinism
616	QN_AMI_OTHER_ENDO_METAB	7259005	Mucopolysaccharidosis, MPS-IV-A
616	QN_AMI_OTHER_ENDO_METAB	7265005	Glycogen storage disease, type I
616	QN_AMI_OTHER_ENDO_METAB	7573000	Classical phenylketonuria
616	QN_AMI_OTHER_ENDO_METAB	9105005	Muscle AMP deaminase deficiency
616	QN_AMI_OTHER_ENDO_METAB	9311003	Hermansky-Pudlak syndrome
616	QN_AMI_OTHER_ENDO_METAB	10406007	Lesch-Nyhan syndrome
616	QN_AMI_OTHER_ENDO_METAB	10649000	Hyperpituitarism
616	QN_AMI_OTHER_ENDO_METAB	10741005	Lipid storage disease
616	QN_AMI_OTHER_ENDO_METAB	11282001	Homocystinuria
616	QN_AMI_OTHER_ENDO_METAB	11380006	Mucopolysaccharidosis
616	QN_AMI_OTHER_ENDO_METAB	12579009	Familial Mediterranean fever
616	QN_AMI_OTHER_ENDO_METAB	15771004	Diabetes insipidus
616	QN_AMI_OTHER_ENDO_METAB	15890002	Albinism
616	QN_AMI_OTHER_ENDO_METAB	16652001	Fabry's disease
616	QN_AMI_OTHER_ENDO_METAB	16784003	Amino acid transport disorder
616	QN_AMI_OTHER_ENDO_METAB	17602002	Amyloidosis
616	QN_AMI_OTHER_ENDO_METAB	18927009	Niemann-Pick disease, type D

© 2021 Matheny ME et al. *JAMA Network Open.*

CONDITIONID	CONDITION_DESCRIPTION	SNOMED_CODE	DESCRIPTION
616	QN_AMI_OTHER_ENDO_METAB	19034001	Hyperparathyroidism due to renal insufficiency
616	QN_AMI_OTHER_ENDO_METAB	20673009	Disorder of thymus gland
616	QN_AMI_OTHER_ENDO_METAB	20957000	Disorder of carbohydrate metabolism
616	QN_AMI_OTHER_ENDO_METAB	21263006	Myxedema coma
616	QN_AMI_OTHER_ENDO_METAB	21764004	Renal carnitine transport defect
616	QN_AMI_OTHER_ENDO_METAB	21806007	Aciduria
616	QN_AMI_OTHER_ENDO_METAB	22886006	Glutaric aciduria, type 2
616	QN_AMI_OTHER_ENDO_METAB	22935002	Congenital erythropoietic porphyria
616	QN_AMI_OTHER_ENDO_METAB	23501004	Arginase deficiency
616	QN_AMI_OTHER_ENDO_METAB	24743004	Complement deficiency disease
616	QN_AMI_OTHER_ENDO_METAB	24867002	Severe adrenal insufficiency
616	QN_AMI_OTHER_ENDO_METAB	25792000	Kearns-Sayre syndrome
616	QN_AMI_OTHER_ENDO_METAB	26336006	Tyrosinase-positive oculocutaneous albinism
616	QN_AMI_OTHER_ENDO_METAB	26399002	Ocular albinism
616	QN_AMI_OTHER_ENDO_METAB	26745009	Mucopolysaccharidosis, MPS-I-H/S
616	QN_AMI_OTHER_ENDO_METAB	27718001	Maple syrup urine disease
616	QN_AMI_OTHER_ENDO_METAB	28882002	Disorder of sulfur-bearing amino acid metabolism
616	QN_AMI_OTHER_ENDO_METAB	29094004	Disorder of porphyrin metabolism
616	QN_AMI_OTHER_ENDO_METAB	29633007	Glycogen storage disease

© 2021 Matheny ME et al. *JAMA Network Open.*

CONDITIONID	CONDITION_DESCRIPTION	SNOMED_CODE	DESCRIPTION
616	QN_AMI_OTHER_ENDO_METAB	30171000	Disorder of adrenal gland
616	QN_AMI_OTHER_ENDO_METAB	30188007	Alpha-1-antitrypsin deficiency
616	QN_AMI_OTHER_ENDO_METAB	30635002	Abscess of thymus
616	QN_AMI_OTHER_ENDO_METAB	30664006	Multiple endocrine neoplasia, type 1
616	QN_AMI_OTHER_ENDO_METAB	30911005	Cryoglobulinemia
616	QN_AMI_OTHER_ENDO_METAB	32390006	Panhypopituitarism
616	QN_AMI_OTHER_ENDO_METAB	35400008	Hereditary hemochromatosis
616	QN_AMI_OTHER_ENDO_METAB	36102002	Waterhouse-Friderichsen syndrome
616	QN_AMI_OTHER_ENDO_METAB	36348003	Primary hyperparathyroidism
616	QN_AMI_OTHER_ENDO_METAB	36444000	Disorder of the urea cycle metabolism
616	QN_AMI_OTHER_ENDO_METAB	36976004	Hypoparathyroidism
616	QN_AMI_OTHER_ENDO_METAB	37200009	Disorder of tyrosine metabolism
616	QN_AMI_OTHER_ENDO_METAB	38795005	Sialidosis
616	QN_AMI_OTHER_ENDO_METAB	39390005	Niemann-Pick disease, type B
616	QN_AMI_OTHER_ENDO_METAB	39925003	Juvenile myopathy, encephalopathy, lactic acidosis AND stroke
616	QN_AMI_OTHER_ENDO_METAB	39929009	Disorder of fatty acid metabolism
616	QN_AMI_OTHER_ENDO_METAB	41299009	Iatrogenic Cushing's disease
616	QN_AMI_OTHER_ENDO_METAB	41797007	5,10-Methylenetetrahydrofolate reductase deficiency

© 2021 Matheny ME et al. *JAMA Network Open.*

CONDITIONID	CONDITION_DESCRIPTION	SNOMED_CODE	DESCRIPTION
616	QN_AMI_OTHER_ENDO_METAB	41864002	Autoimmune polyendocrinopathy
616	QN_AMI_OTHER_ENDO_METAB	42295001	Familial amyloid polyneuropathy
616	QN_AMI_OTHER_ENDO_METAB	42393006	Methylmalonic acidemia
616	QN_AMI_OTHER_ENDO_METAB	43019009	Nelson syndrome
616	QN_AMI_OTHER_ENDO_METAB	44176004	Disorder of histidine metabolism
616	QN_AMI_OTHER_ENDO_METAB	44779003	Disorder of amino acid metabolism
616	QN_AMI_OTHER_ENDO_METAB	46011003	Ruvalcaba-Myhre syndrome
616	QN_AMI_OTHER_ENDO_METAB	47270006	Hypercortisolism
616	QN_AMI_OTHER_ENDO_METAB	51742006	Disorder of anterior pituitary
616	QN_AMI_OTHER_ENDO_METAB	52165006	Niemann-Pick disease, type A
616	QN_AMI_OTHER_ENDO_METAB	54905006	Disorder of carbohydrate transport
616	QN_AMI_OTHER_ENDO_METAB	55004003	Syndrome of inappropriate vasopressin secretion
616	QN_AMI_OTHER_ENDO_METAB	55912009	Glycogen storage disease, type V
616	QN_AMI_OTHER_ENDO_METAB	56692003	Rhizomelic chondrodysplasia punctata syndrome
616	QN_AMI_OTHER_ENDO_METAB	56871000	Localized amyloidosis
616	QN_AMI_OTHER_ENDO_METAB	58459009	Sphingomyelin/cholesterol lipidosis
616	QN_AMI_OTHER_ENDO_METAB	60086000	Aldosterone deficiency
616	QN_AMI_OTHER_ENDO_METAB	61530001	Multiple endocrine neoplasia, type 3

© 2021 Matheny ME et al. *JAMA Network Open.*

CONDITIONID	CONDITION_DESCRIPTION	SNOMED_CODE	DESCRIPTION
616	QN_AMI_OTHER_ENDO_METAB	61808009	Multiple endocrine neoplasia, type 2
616	QN_AMI_OTHER_ENDO_METAB	61860000	Porphyria cutanea tarda
616	QN_AMI_OTHER_ENDO_METAB	63844009	Oculocutaneous albinism
616	QN_AMI_OTHER_ENDO_METAB	65327002	Mucopolysaccharidosis, MPS-I-H
616	QN_AMI_OTHER_ENDO_METAB	65389002	Adrenoleukodystrophy
616	QN_AMI_OTHER_ENDO_METAB	66751000	Niemann-Pick disease, type C
616	QN_AMI_OTHER_ENDO_METAB	66937008	Glycogen storage disease type III
616	QN_AMI_OTHER_ENDO_METAB	66999008	Hyperparathyroidism
616	QN_AMI_OTHER_ENDO_METAB	67805000	Secondary hyperaldosteronism
616	QN_AMI_OTHER_ENDO_METAB	68448003	Myoclonus epilepsy AND ragged red fibers
616	QN_AMI_OTHER_ENDO_METAB	68528007	Hyperphenylalaninemia
616	QN_AMI_OTHER_ENDO_METAB	69080001	Propionic acidemia
616	QN_AMI_OTHER_ENDO_METAB	70737009	Mucopolysaccharidosis, MPS-II
616	QN_AMI_OTHER_ENDO_METAB	72442006	Disorder of posterior pituitary
616	QN_AMI_OTHER_ENDO_METAB	73123008	Mucopolysaccharidosis, MPS-I-S
616	QN_AMI_OTHER_ENDO_METAB	73132005	Disorder of parathyroid gland
616	QN_AMI_OTHER_ENDO_METAB	74728003	Hypopituitarism
616	QN_AMI_OTHER_ENDO_METAB	75934005	Metabolic disease
616	QN_AMI_OTHER_ENDO_METAB	78548001	Enzymopathy
616	QN_AMI_OTHER_ENDO_METAB	78642008	Ocular albinism, type I
616	QN_AMI_OTHER_ENDO_METAB	78921008	Autosomal recessive ocular albinism

© 2021 Matheny ME et al. *JAMA Network Open.*

CONDITIONID	CONDITION_DESCRIPTION	SNOMED_CODE	DESCRIPTION
616	QN_AMI_OTHER_ENDO_METAB	79385002	Lowe syndrome
616	QN_AMI_OTHER_ENDO_METAB	80902009	Neutral 1 amino acid transport defect
616	QN_AMI_OTHER_ENDO_METAB	83076007	Disorder of glycine metabolism
616	QN_AMI_OTHER_ENDO_METAB	84618009	Disorder of propionate AND/OR methylmalonate metabolism
616	QN_AMI_OTHER_ENDO_METAB	85020001	Cystinuria
616	QN_AMI_OTHER_ENDO_METAB	86842008	Iatrogenic pituitary disorder
616	QN_AMI_OTHER_ENDO_METAB	87827003	Isovaleryl-CoA dehydrogenase deficiency
616	QN_AMI_OTHER_ENDO_METAB	88213004	Hyperaldosteronism
616	QN_AMI_OTHER_ENDO_METAB	88469006	Zellweger syndrome
616	QN_AMI_OTHER_ENDO_METAB	90500005	Carnitine palmitoyltransferase deficiency
616	QN_AMI_OTHER_ENDO_METAB	91478007	Secondary hyperparathyroidism
616	QN_AMI_OTHER_ENDO_METAB	111395007	Nephrogenic diabetes insipidus
616	QN_AMI_OTHER_ENDO_METAB	111396008	ChÃ©diak-Higashi syndrome
616	QN_AMI_OTHER_ENDO_METAB	111544009	Combination of endocrine dysfunction
616	QN_AMI_OTHER_ENDO_METAB	111546006	Polyglandular dysfunction
616	QN_AMI_OTHER_ENDO_METAB	111548007	Syndrome of diencephalo-hypophyseal origin
616	QN_AMI_OTHER_ENDO_METAB	111563005	Adrenal hypofunction

© 2021 Matheny ME et al. *JAMA Network Open.*

CONDITIONID	CONDITION_DESCRIPTION	SNOMED_CODE	DESCRIPTION
616	QN_AMI_OTHER_ENDO_METAB	111565003	Medulloadrenal hyperfunction
616	QN_AMI_OTHER_ENDO_METAB	116020001	Disorder of branched-chain amino acid metabolism
616	QN_AMI_OTHER_ENDO_METAB	124166007	Deficiency of butyryl-CoA dehydrogenase
616	QN_AMI_OTHER_ENDO_METAB	124202004	Deficiency of catalase
616	QN_AMI_OTHER_ENDO_METAB	128596003	Medium-chain acyl-coenzyme A dehydrogenase deficiency
616	QN_AMI_OTHER_ENDO_METAB	190502001	Pituitary dependent hypercortisolism
616	QN_AMI_OTHER_ENDO_METAB	190507007	Primary aldosteronism
616	QN_AMI_OTHER_ENDO_METAB	190524001	Drug-induced adrenocortical insufficiency
616	QN_AMI_OTHER_ENDO_METAB	190525000	Postprocedural adrenocortical(-medullary) hypofunction
616	QN_AMI_OTHER_ENDO_METAB	190681003	Cystinosis
616	QN_AMI_OTHER_ENDO_METAB	190694001	Tyrosinemia
616	QN_AMI_OTHER_ENDO_METAB	190745006	Galactosemia
616	QN_AMI_OTHER_ENDO_METAB	190760009	Disorders of pyruvate metabolism and gluconeogenesis
616	QN_AMI_OTHER_ENDO_METAB	190794006	Gaucher's disease
616	QN_AMI_OTHER_ENDO_METAB	190817009	Macroglobulinemia
616	QN_AMI_OTHER_ENDO_METAB	190818004	WaldenstrÃ¶m macroglobulinemia
616	QN_AMI_OTHER_ENDO_METAB	190948002	Defect in post-translational

© 2021 Matheny ME et al. *JAMA Network Open.*

CONDITIONID	CONDITION_DESCRIPTION	SNOMED_CODE	DESCRIPTION
			modification of lysosomal enzymes
616	QN_AMI_OTHER_ENDO_METAB	230796005	Non-diabetic hypoglycemic coma
616	QN_AMI_OTHER_ENDO_METAB	234422006	Acute intermittent porphyria
616	QN_AMI_OTHER_ENDO_METAB	237654002	Idiopathic hypoparathyroidism
616	QN_AMI_OTHER_ENDO_METAB	237662005	Hyperprolactinemia
616	QN_AMI_OTHER_ENDO_METAB	237738005	Alcohol-induced pseudo-Cushing's syndrome
616	QN_AMI_OTHER_ENDO_METAB	237751000	Congenital adrenal hyperplasia
616	QN_AMI_OTHER_ENDO_METAB	237781008	Adrenomedullary hyperplasia
616	QN_AMI_OTHER_ENDO_METAB	237827002	Polyglandular hyperfunction
616	QN_AMI_OTHER_ENDO_METAB	237835004	Constitutional tall stature
616	QN_AMI_OTHER_ENDO_METAB	237868006	Familial non-neuropathic amyloidosis
616	QN_AMI_OTHER_ENDO_METAB	237911005	Disorder of amino acid and organic acid metabolism
616	QN_AMI_OTHER_ENDO_METAB	237928008	Disorder of ornithine metabolism
616	QN_AMI_OTHER_ENDO_METAB	237929000	Disorder of lysine and hydroxylysine metabolism
616	QN_AMI_OTHER_ENDO_METAB	237939006	Non-ketotic hyperglycinemia
616	QN_AMI_OTHER_ENDO_METAB	237950009	3-Methylglutaconic aciduria
616	QN_AMI_OTHER_ENDO_METAB	237959005	Trimethylaminuria

© 2021 Matheny ME et al. *JAMA Network Open.*

CONDITIONID	CONDITION_DESCRIPTION	SNOMED_CODE	DESCRIPTION
616	QN_AMI_OTHER_ENDO_METAB	237963003	Disorder of galactose metabolism
616	QN_AMI_OTHER_ENDO_METAB	237997005	Very long chain acyl-CoA dehydrogenase deficiency
616	QN_AMI_OTHER_ENDO_METAB	238006008	Disorder of purine and pyrimidine metabolism
616	QN_AMI_OTHER_ENDO_METAB	238028008	Sphingolipidosis
616	QN_AMI_OTHER_ENDO_METAB	238043005	Disorder of glycosaminoglycan metabolism
616	QN_AMI_OTHER_ENDO_METAB	238044004	Mucopolysaccharidosis, MPS-IV-B
616	QN_AMI_OTHER_ENDO_METAB	238045003	Disorder of glycoprotein metabolism
616	QN_AMI_OTHER_ENDO_METAB	238059005	Disorder of peroxisomal function
616	QN_AMI_OTHER_ENDO_METAB	238061001	Neonatal adrenoleucodystrophy
616	QN_AMI_OTHER_ENDO_METAB	238064009	Zellweger's-like syndrome
616	QN_AMI_OTHER_ENDO_METAB	238112001	Carnitine nutritional deficiency
616	QN_AMI_OTHER_ENDO_METAB	240096000	Mitochondrial cytopathy
616	QN_AMI_OTHER_ENDO_METAB	267386008	Gigantism and acromegaly
616	QN_AMI_OTHER_ENDO_METAB	267395000	Adrenogenital disorder
616	QN_AMI_OTHER_ENDO_METAB	267430007	Renal glycosuria
616	QN_AMI_OTHER_ENDO_METAB	267431006	Disorder of lipid metabolism

© 2021 Matheny ME et al. *JAMA Network Open.*

CONDITIONID	CONDITION_DESCRIPTION	SNOMED_CODE	DESCRIPTION
616	QN_AMI_OTHER_ENDO_METAB	274864009	Glycogen storage disease due to acid maltase deficiency
616	QN_AMI_OTHER_ENDO_METAB	275437005	Adrenocortical hyperfunction
616	QN_AMI_OTHER_ENDO_METAB	281034005	Secondary amyloidosis
616	QN_AMI_OTHER_ENDO_METAB	302865004	Non-diabetic disorder of endocrine pancreas
616	QN_AMI_OTHER_ENDO_METAB	361201009	Persistent hyperplasia of thymus
616	QN_AMI_OTHER_ENDO_METAB	367460001	Pituitary dwarfism
616	QN_AMI_OTHER_ENDO_METAB	373662000	Primary adrenocortical insufficiency
616	QN_AMI_OTHER_ENDO_METAB	386584007	Adrenal cortical hypofunction
616	QN_AMI_OTHER_ENDO_METAB	398680004	Citrullinemia
616	QN_AMI_OTHER_ENDO_METAB	399100005	Disorder of hypothalamus
616	QN_AMI_OTHER_ENDO_METAB	399244003	Disorder of pituitary gland
616	QN_AMI_OTHER_ENDO_METAB	408670001	Iatrogenic carnitine deficiency
616	QN_AMI_OTHER_ENDO_METAB	410058007	Histidinemia
616	QN_AMI_OTHER_ENDO_METAB	414031008	Immunosecretory disorder
616	QN_AMI_OTHER_ENDO_METAB	418470004	Porphyria
616	QN_AMI_OTHER_ENDO_METAB	421784001	Carnitine deficiency
616	QN_AMI_OTHER_ENDO_METAB	426655001	Disorder of aromatic amino acid metabolism
616	QN_AMI_OTHER_ENDO_METAB	699346009	Hereditary cancer-predisposing syndrome
616	QN_AMI_OTHER_ENDO_METAB	707742001	Bartter syndrome

© 2021 Matheny ME et al. *JAMA Network Open.*

CONDITIONID	CONDITION_DESCRIPTION	SNOMED_CODE	DESCRIPTION
616	QN_AMI_OTHER_ENDO_METAB	55341000119107	Carnitine deficiency due to inborn error of metabolism
616	QN_AMI_OTHER_ENDO_METAB	83201000119108	Secondary carnitine deficiency
616	QN_AMI_OTHER_ENDO_METAB	133791000119107	Mitochondrial metabolism defect
616	QN_AMI_OTHER_ENDO_METAB	366951000119109	Adolescent X-linked adrenoleukodystrophy
616	QN_AMI_OTHER_ENDO_METAB	367041000119108	Childhood cerebral X-linked adrenoleukodystrophy
616	QN_AMI_OTHER_ENDO_METAB	367621000119107	Hyperoxaluria
616	QN_AMI_OTHER_ENDO_METAB	367681000119106	Disorder of ketone metabolism
618	QN_AMI_DO_LIPID_METAB	10741005	Lipid storage disease
618	QN_AMI_DO_LIPID_METAB	39929009	Disorder of fatty acid metabolism
618	QN_AMI_DO_LIPID_METAB	55822004	Hyperlipidemia
618	QN_AMI_DO_LIPID_METAB	84241008	Lipoid dermatoarthritis
618	QN_AMI_DO_LIPID_METAB	123963007	Disorder of cholesterol metabolism
618	QN_AMI_DO_LIPID_METAB	267431006	Disorder of lipid metabolism
618	QN_AMI_DO_LIPID_METAB	267432004	Pure hypercholesterolemia
618	QN_AMI_DO_LIPID_METAB	267433009	Pure hyperglyceridemia
618	QN_AMI_DO_LIPID_METAB	267434003	Mixed hyperlipidemia

© 2021 Matheny ME et al. *JAMA Network Open.*

CONDITIONID	CONDITION_DESCRIPTION	SNOMED_CODE	DESCRIPTION
618	QN_AMI_DO_LIPID_METAB	267435002	Familial hyperchylomicronemia
618	QN_AMI_DO_LIPID_METAB	267436001	Lipoprotein deficiency disorder
618	QN_AMI_DO_LIPID_METAB	402693001	Lipomatosis
619	QN_AMI_OTHER_ENDO_METAB_NUTR_DO	547009	Hypersecretion of calcitonin
619	QN_AMI_OTHER_ENDO_METAB_NUTR_DO	3716002	Goiter
619	QN_AMI_OTHER_ENDO_METAB_NUTR_DO	4184009	Congenital anomaly of endocrine gland
619	QN_AMI_OTHER_ENDO_METAB_NUTR_DO	4715007	Vitamin A deficiency with xerophthalmic scars of cornea
619	QN_AMI_OTHER_ENDO_METAB_NUTR_DO	5291005	Hypocalcemia
619	QN_AMI_OTHER_ENDO_METAB_NUTR_DO	5388008	Congenital lactase deficiency
619	QN_AMI_OTHER_ENDO_METAB_NUTR_DO	8659000	Ectopic production of endocrine substance
619	QN_AMI_OTHER_ENDO_METAB_NUTR_DO	8659000	Ectopic production of endocrine substance
619	QN_AMI_OTHER_ENDO_METAB_NUTR_DO	8808004	Biotinidase deficiency
619	QN_AMI_OTHER_ENDO_METAB_NUTR_DO	12313004	Androgen resistance syndrome
619	QN_AMI_OTHER_ENDO_METAB_NUTR_DO	14304000	Disorder of thyroid gland
619	QN_AMI_OTHER_ENDO_METAB_NUTR_DO	15509004	Vitamin A deficiency with night blindness
619	QN_AMI_OTHER_ENDO_METAB_NUTR_DO	19577007	Hypocupremia
619	QN_AMI_OTHER_ENDO_METAB_NUTR_DO	20052008	Hereditary fructosuria

© 2021 Matheny ME et al. *JAMA Network Open.*

CONDITIONID	CONDITION_DESCRIPTION	SNOMED_CODE	DESCRIPTION
619	QN_AMI_OTHER_ENDO_METAB_NUTR_DO	20307000	Ariboflavinosis
619	QN_AMI_OTHER_ENDO_METAB_NUTR_DO	21007002	Wernicke's disease
619	QN_AMI_OTHER_ENDO_METAB_NUTR_DO	21983002	Hashimoto thyroiditis
619	QN_AMI_OTHER_ENDO_METAB_NUTR_DO	22169002	Intestinal disaccharidase deficiency
619	QN_AMI_OTHER_ENDO_METAB_NUTR_DO	26389007	Toxic multinodular goiter
619	QN_AMI_OTHER_ENDO_METAB_NUTR_DO	27503000	Gilbert's syndrome
619	QN_AMI_OTHER_ENDO_METAB_NUTR_DO	27712000	Hypervitaminosis D
619	QN_AMI_OTHER_ENDO_METAB_NUTR_DO	27796003	Vitamin A deficiency with conjunctival xerosis
619	QN_AMI_OTHER_ENDO_METAB_NUTR_DO	28259009	Crigler-Najjar syndrome
619	QN_AMI_OTHER_ENDO_METAB_NUTR_DO	29028009	Thyrotoxic crisis
619	QN_AMI_OTHER_ENDO_METAB_NUTR_DO	29206004	Testicular hyperfunction
619	QN_AMI_OTHER_ENDO_METAB_NUTR_DO	30913008	Disorder of iron metabolism
619	QN_AMI_OTHER_ENDO_METAB_NUTR_DO	30985009	Toxic nodular goiter with thyrotoxic storm
619	QN_AMI_OTHER_ENDO_METAB_NUTR_DO	34713006	Vitamin D deficiency
619	QN_AMI_OTHER_ENDO_METAB_NUTR_DO	35240004	Iron deficiency
619	QN_AMI_OTHER_ENDO_METAB_NUTR_DO	35487009	Hypercarotinemia
619	QN_AMI_OTHER_ENDO_METAB_NUTR_DO	35516008	Vitamin A deficiency with corneal xerosis
619	QN_AMI_OTHER_ENDO_METAB_NUTR_DO	35758009	Impaired intestinal carbohydrate absorption

© 2021 Matheny ME et al. *JAMA Network Open.*

CONDITIONID	CONDITION_DESCRIPTION	SNOMED_CODE	DESCRIPTION
619	QN_AMI_OTHER_ENDO_METAB_NUTR_DO	36241006	Non-toxic multinodular goiter
619	QN_AMI_OTHER_ENDO_METAB_NUTR_DO	37102008	Disorder of endocrine ovary
619	QN_AMI_OTHER_ENDO_METAB_NUTR_DO	37295009	Hyperestrogenism
619	QN_AMI_OTHER_ENDO_METAB_NUTR_DO	38727009	Subacute thyroiditis
619	QN_AMI_OTHER_ENDO_METAB_NUTR_DO	39452003	Fructose metabolism disorder
619	QN_AMI_OTHER_ENDO_METAB_NUTR_DO	40278002	Essential benign fructosuria
619	QN_AMI_OTHER_ENDO_METAB_NUTR_DO	40930008	Hypothyroidism
619	QN_AMI_OTHER_ENDO_METAB_NUTR_DO	42639000	Vitamin A deficiency with keratomalacia
619	QN_AMI_OTHER_ENDO_METAB_NUTR_DO	43595007	Chromium deficiency
619	QN_AMI_OTHER_ENDO_METAB_NUTR_DO	45053005	Chronic thyroiditis
619	QN_AMI_OTHER_ENDO_METAB_NUTR_DO	45744005	Disorder of mineral metabolism
619	QN_AMI_OTHER_ENDO_METAB_NUTR_DO	47421005	Manganese deficiency
619	QN_AMI_OTHER_ENDO_METAB_NUTR_DO	47903000	Vitamin B deficiency
619	QN_AMI_OTHER_ENDO_METAB_NUTR_DO	49340009	Vitamin A deficiency with Bitot's spot AND conjunctival xerosis
619	QN_AMI_OTHER_ENDO_METAB_NUTR_DO	49607006	Biotin deficiency disease
619	QN_AMI_OTHER_ENDO_METAB_NUTR_DO	52675005	Vitamin K deficiency
619	QN_AMI_OTHER_ENDO_METAB_NUTR_DO	54137008	Vitamin E deficiency
619	QN_AMI_OTHER_ENDO_METAB_NUTR_DO	57777000	Toxic nodular goiter

© 2021 Matheny ME et al. *JAMA Network Open.*

CONDITIONID	CONDITION_DESCRIPTION	SNOMED_CODE	DESCRIPTION
619	QN_AMI_OTHER_ENDO_METAB_NUTR_DO	58976002	Pseudohypoparathyroidism
619	QN_AMI_OTHER_ENDO_METAB_NUTR_DO	60216004	Toxic diffuse goiter with thyrotoxic crisis
619	QN_AMI_OTHER_ENDO_METAB_NUTR_DO	60414003	Acquired lactase deficiency
619	QN_AMI_OTHER_ENDO_METAB_NUTR_DO	60637003	Congenital anomaly of adrenal gland
619	QN_AMI_OTHER_ENDO_METAB_NUTR_DO	60853003	Disorder of magnesium metabolism
619	QN_AMI_OTHER_ENDO_METAB_NUTR_DO	61556008	Iatrogenic thyroiditis
619	QN_AMI_OTHER_ENDO_METAB_NUTR_DO	62278002	Toxic multinodular goiter with thyrotoxic crisis
619	QN_AMI_OTHER_ENDO_METAB_NUTR_DO	64559002	Hypervitaminosis A
619	QN_AMI_OTHER_ENDO_METAB_NUTR_DO	65846009	Primary ovarian failure
619	QN_AMI_OTHER_ENDO_METAB_NUTR_DO	66931009	Hypercalcemia
619	QN_AMI_OTHER_ENDO_METAB_NUTR_DO	66944004	Autoimmune thyroiditis
619	QN_AMI_OTHER_ENDO_METAB_NUTR_DO	66978005	Hypermagnesemia
619	QN_AMI_OTHER_ENDO_METAB_NUTR_DO	67360000	Wet beriberi
619	QN_AMI_OTHER_ENDO_METAB_NUTR_DO	67528009	Incomplete testicular feminization syndrome
619	QN_AMI_OTHER_ENDO_METAB_NUTR_DO	68295002	Vitamin D-dependent rickets
619	QN_AMI_OTHER_ENDO_METAB_NUTR_DO	69329005	Toxic uninodular goiter with thyrotoxic crisis
619	QN_AMI_OTHER_ENDO_METAB_NUTR_DO	69878008	Polycystic ovaries

© 2021 Matheny ME et al. *JAMA Network Open.*

CONDITIONID	CONDITION_DESCRIPTION	SNOMED_CODE	DESCRIPTION
619	QN_AMI_OTHER_ENDO_METAB_NUTR_DO	70241007	Nutritional deficiency disorder
619	QN_AMI_OTHER_ENDO_METAB_NUTR_DO	71021002	Dry beriberi
619	QN_AMI_OTHER_ENDO_METAB_NUTR_DO	71325002	Lipodystrophy
619	QN_AMI_OTHER_ENDO_METAB_NUTR_DO	71638002	Disorder of calcium metabolism
619	QN_AMI_OTHER_ENDO_METAB_NUTR_DO	72000004	Vitamin A deficiency
619	QN_AMI_OTHER_ENDO_METAB_NUTR_DO	72325004	Cyst of thyroid
619	QN_AMI_OTHER_ENDO_METAB_NUTR_DO	73820008	Disorder of endocrine testis
619	QN_AMI_OTHER_ENDO_METAB_NUTR_DO	73869005	Toxic uninodular goiter
619	QN_AMI_OTHER_ENDO_METAB_NUTR_DO	75934005	Metabolic disease
619	QN_AMI_OTHER_ENDO_METAB_NUTR_DO	76169001	Ascorbic acid deficiency
619	QN_AMI_OTHER_ENDO_METAB_NUTR_DO	78373000	Sucrase-isomaltase deficiency
619	QN_AMI_OTHER_ENDO_METAB_NUTR_DO	79886009	Disorder of copper metabolism
619	QN_AMI_OTHER_ENDO_METAB_NUTR_DO	80006005	Disorder of bilirubin metabolism
619	QN_AMI_OTHER_ENDO_METAB_NUTR_DO	81891001	Hypersecretion of ovarian androgens
619	QN_AMI_OTHER_ENDO_METAB_NUTR_DO	82119001	Thyroiditis
619	QN_AMI_OTHER_ENDO_METAB_NUTR_DO	82236004	Familial x-linked hypophosphatemic vitamin D refractory rickets
619	QN_AMI_OTHER_ENDO_METAB_NUTR_DO	84362007	Fibrous autoimmune thyroiditis
619	QN_AMI_OTHER_ENDO_METAB_NUTR_DO	85670002	Vitamin deficiency

© 2021 Matheny ME et al. *JAMA Network Open.*

CONDITIONID	CONDITION_DESCRIPTION	SNOMED_CODE	DESCRIPTION
619	QN_AMI_OTHER_ENDO_METAB_NUTR_DO	87049008	Disorder of phosphorus metabolism
619	QN_AMI_OTHER_ENDO_METAB_NUTR_DO	87232008	Hyperthyroidism due to ectopic thyroid nodule
619	QN_AMI_OTHER_ENDO_METAB_NUTR_DO	88273006	Iatrogenic hypothyroidism
619	QN_AMI_OTHER_ENDO_METAB_NUTR_DO	88518009	Wilson's disease
619	QN_AMI_OTHER_ENDO_METAB_NUTR_DO	88740003	Thyrotoxicosis factitia with thyrotoxic crisis
619	QN_AMI_OTHER_ENDO_METAB_NUTR_DO	89719007	Thyrotoxicosis without goiter OR other cause
619	QN_AMI_OTHER_ENDO_METAB_NUTR_DO	90653005	Vitamin A deficiency with corneal ulceration AND xerosis
619	QN_AMI_OTHER_ENDO_METAB_NUTR_DO	90739004	Thyrotoxicosis
619	QN_AMI_OTHER_ENDO_METAB_NUTR_DO	109983007	Monoclonal gammopathy
619	QN_AMI_OTHER_ENDO_METAB_NUTR_DO	111001004	Gammopathy
619	QN_AMI_OTHER_ENDO_METAB_NUTR_DO	111379007	Mineral deficiency
619	QN_AMI_OTHER_ENDO_METAB_NUTR_DO	111400008	Vitamin A deficiency with ocular manifestation
619	QN_AMI_OTHER_ENDO_METAB_NUTR_DO	111549004	Ovarian hyperfunction
619	QN_AMI_OTHER_ENDO_METAB_NUTR_DO	111550004	Ovarian failure
619	QN_AMI_OTHER_ENDO_METAB_NUTR_DO	111551000	Testicular hypofunction
619	QN_AMI_OTHER_ENDO_METAB_NUTR_DO	111566002	Acquired hypothyroidism

© 2021 Matheny ME et al. *JAMA Network Open.*

CONDITIONID	CONDITION_DESCRIPTION	SNOMED_CODE	DESCRIPTION
619	QN_AMI_OTHER_ENDO_METAB_NUTR_DO	123527003	Precocious sexual development
619	QN_AMI_OTHER_ENDO_METAB_NUTR_DO	190236006	Non-toxic nodular goiter
619	QN_AMI_OTHER_ENDO_METAB_NUTR_DO	190237002	Non-toxic uninodular goiter
619	QN_AMI_OTHER_ENDO_METAB_NUTR_DO	190255006	Hyperthyroidism due to ectopic thyroid tissue
619	QN_AMI_OTHER_ENDO_METAB_NUTR_DO	190256007	Thyrotoxicosis from ectopic thyroid nodule with crisis
619	QN_AMI_OTHER_ENDO_METAB_NUTR_DO	190279008	Iodine hypothyroidism
619	QN_AMI_OTHER_ENDO_METAB_NUTR_DO	190293001	Acute thyroiditis
619	QN_AMI_OTHER_ENDO_METAB_NUTR_DO	190304001	Dyshormonogenic goiter
619	QN_AMI_OTHER_ENDO_METAB_NUTR_DO	190305000	Thyroid hemorrhage and infarction
619	QN_AMI_OTHER_ENDO_METAB_NUTR_DO	190437000	Post-surgical hypoinsulinemia
619	QN_AMI_OTHER_ENDO_METAB_NUTR_DO	190448007	Drug-induced hypoglycemia without coma
619	QN_AMI_OTHER_ENDO_METAB_NUTR_DO	190538008	Postablative ovarian failure
619	QN_AMI_OTHER_ENDO_METAB_NUTR_DO	190552005	Postablative testicular hypofunction
619	QN_AMI_OTHER_ENDO_METAB_NUTR_DO	190641005	Late effect of rickets
619	QN_AMI_OTHER_ENDO_METAB_NUTR_DO	190656001	Dietary calcium deficiency
619	QN_AMI_OTHER_ENDO_METAB_NUTR_DO	190660003	Dietary selenium deficiency
619	QN_AMI_OTHER_ENDO_METAB_NUTR_DO	190661004	Dietary zinc deficiency

© 2021 Matheny ME et al. *JAMA Network Open.*

CONDITIONID	CONDITION_DESCRIPTION	SNOMED_CODE	DESCRIPTION
619	QN_AMI_OTHER_ENDO_METAB_NUTR_DO	190669002	Deficiency of multiple nutrient elements
619	QN_AMI_OTHER_ENDO_METAB_NUTR_DO	190671002	Imbalance of constituents of food intake
619	QN_AMI_OTHER_ENDO_METAB_NUTR_DO	190674005	Sequelae of malnutrition and other nutritional deficiencies
619	QN_AMI_OTHER_ENDO_METAB_NUTR_DO	190676007	Sequelae of vitamin A deficiency
619	QN_AMI_OTHER_ENDO_METAB_NUTR_DO	190677003	Sequelae of vitamin C deficiency
619	QN_AMI_OTHER_ENDO_METAB_NUTR_DO	190808009	Polyclonal hypergammaglobulinemia
619	QN_AMI_OTHER_ENDO_METAB_NUTR_DO	190855004	Hypomagnesemia
619	QN_AMI_OTHER_ENDO_METAB_NUTR_DO	190932003	Disorders of bilirubin excretion
619	QN_AMI_OTHER_ENDO_METAB_NUTR_DO	190971000	Sequelae of hyperalimentation
619	QN_AMI_OTHER_ENDO_METAB_NUTR_DO	213201002	Hyperstimulation of ovaries
619	QN_AMI_OTHER_ENDO_METAB_NUTR_DO	217710005	Congenital iodine deficiency syndrome
619	QN_AMI_OTHER_ENDO_METAB_NUTR_DO	237494009	Diffuse goiter
619	QN_AMI_OTHER_ENDO_METAB_NUTR_DO	237515009	Congenital hypothyroidism without goiter
619	QN_AMI_OTHER_ENDO_METAB_NUTR_DO	237527007	Postablative hypothyroidism
619	QN_AMI_OTHER_ENDO_METAB_NUTR_DO	237528002	Post-infectious hypothyroidism

© 2021 Matheny ME et al. *JAMA Network Open.*

CONDITIONID	CONDITION_DESCRIPTION	SNOMED_CODE	DESCRIPTION
619	QN_AMI_OTHER_ENDO_METAB_NUTR_DO	237538007	Chronic thyroiditis with transient thyrotoxicosis
619	QN_AMI_OTHER_ENDO_METAB_NUTR_DO	237539004	Drug-induced thyroiditis
619	QN_AMI_OTHER_ENDO_METAB_NUTR_DO	237558008	Atrophy of thyroid - acquired
619	QN_AMI_OTHER_ENDO_METAB_NUTR_DO	237562002	Iodine deficiency syndrome
619	QN_AMI_OTHER_ENDO_METAB_NUTR_DO	237565000	Congenital iodine deficiency syndrome - mixed type
619	QN_AMI_OTHER_ENDO_METAB_NUTR_DO	237566004	Congenital iodine deficiency syndrome - neurological type
619	QN_AMI_OTHER_ENDO_METAB_NUTR_DO	237567008	Subclinical iodine deficiency hypothyroidism
619	QN_AMI_OTHER_ENDO_METAB_NUTR_DO	237571006	Iodine-deficiency-related multinodular endemic goiter
619	QN_AMI_OTHER_ENDO_METAB_NUTR_DO	237602007	Metabolic syndrome X
619	QN_AMI_OTHER_ENDO_METAB_NUTR_DO	237630007	Hypoglycemic disorder
619	QN_AMI_OTHER_ENDO_METAB_NUTR_DO	237812002	Disorder of puberty
619	QN_AMI_OTHER_ENDO_METAB_NUTR_DO	237836003	Short stature disorder
619	QN_AMI_OTHER_ENDO_METAB_NUTR_DO	237883001	Hungry bone syndrome
619	QN_AMI_OTHER_ENDO_METAB_NUTR_DO	238114000	Essential fatty acid deficiency
619	QN_AMI_OTHER_ENDO_METAB_NUTR_DO	238118002	Magnesium deficiency
619	QN_AMI_OTHER_ENDO_METAB_NUTR_DO	238122007	Molybdenum deficiency

© 2021 Matheny ME et al. *JAMA Network Open.*

CONDITIONID	CONDITION_DESCRIPTION	SNOMED_CODE	DESCRIPTION
619	QN_AMI_OTHER_ENDO_METAB_NUTR_DO	238123002	Vanadium deficiency
619	QN_AMI_OTHER_ENDO_METAB_NUTR_DO	238146000	Hypervitaminosis B6
619	QN_AMI_OTHER_ENDO_METAB_NUTR_DO	267374005	Toxic diffuse goiter
619	QN_AMI_OTHER_ENDO_METAB_NUTR_DO	267412000	Thiamine and niacin deficiency states
619	QN_AMI_OTHER_ENDO_METAB_NUTR_DO	267425008	Lactose intolerance
619	QN_AMI_OTHER_ENDO_METAB_NUTR_DO	267440005	Monoclonal paraproteinemia
619	QN_AMI_OTHER_ENDO_METAB_NUTR_DO	270486005	Localized adiposity
619	QN_AMI_OTHER_ENDO_METAB_NUTR_DO	271949009	Iodine-deficiency-related endemic goiter
619	QN_AMI_OTHER_ENDO_METAB_NUTR_DO	278503003	Congenital hypothyroidism with diffuse goiter
619	QN_AMI_OTHER_ENDO_METAB_NUTR_DO	286909009	Thyrotoxicosis with or without goiter
619	QN_AMI_OTHER_ENDO_METAB_NUTR_DO	302866003	Hypoglycemia
619	QN_AMI_OTHER_ENDO_METAB_NUTR_DO	302872003	Disorder of hyperalimentation
619	QN_AMI_OTHER_ENDO_METAB_NUTR_DO	362969004	Disorder of endocrine system
619	QN_AMI_OTHER_ENDO_METAB_NUTR_DO	373717006	Premature menopause
619	QN_AMI_OTHER_ENDO_METAB_NUTR_DO	386080007	Vitamin B6 deficiency
619	QN_AMI_OTHER_ENDO_METAB_NUTR_DO	399187006	Hemochromatosis
619	QN_AMI_OTHER_ENDO_METAB_NUTR_DO	399357009	Thiamine deficiency
619	QN_AMI_OTHER_ENDO_METAB_NUTR_DO	400003000	Delayed puberty
619	QN_AMI_OTHER_ENDO_METAB_NUTR_DO	400179000	Precocious puberty

© 2021 Matheny ME et al. *JAMA Network Open.*

CONDITIONID	CONDITION_DESCRIPTION	SNOMED_CODE	DESCRIPTION
619	QN_AMI_OTHER_ENDO_METAB_NUTR_DO	418186002	Pellagra
619	QN_AMI_OTHER_ENDO_METAB_NUTR_DO	418279001	Niacin deficiency
619	QN_AMI_OTHER_ENDO_METAB_NUTR_DO	122811000119101	Partial androgen insensitivity syndrome
619	QN_AMI_OTHER_ENDO_METAB_NUTR_DO	147211000119101	Disorder of plasma protein metabolism
619	QN_AMI_OTHER_ENDO_METAB_NUTR_DO	367631000119105	Hypothyroidism caused by drug
619	QN_AMI_OTHER_ENDO_METAB_NUTR_DO	368851000119102	Complete androgen insensitivity syndrome
631	QN_AMI_OTHER_GI	1208004	Gastroptosis
631	QN_AMI_OTHER_GI	1296007	Unilateral recurrent femoral hernia without obstruction AND without gangrene
631	QN_AMI_OTHER_GI	2043009	Alcoholic gastritis
631	QN_AMI_OTHER_GI	2303007	Inguinal hernia with gangrene
631	QN_AMI_OTHER_GI	3004001	Congenital dilatation of esophagus
631	QN_AMI_OTHER_GI	3560000	Bilateral recurrent inguinal hernia
631	QN_AMI_OTHER_GI	3696007	Nonulcer dyspepsia
631	QN_AMI_OTHER_GI	3845008	Congenital duplication of intestine
631	QN_AMI_OTHER_GI	4494009	Diverticulitis of large intestine
631	QN_AMI_OTHER_GI	4556007	Gastritis
631	QN_AMI_OTHER_GI	4661003	Calculus of bile duct with obstruction

© 2021 Matheny ME et al. *JAMA Network Open.*

CONDITIONID	CONDITION_DESCRIPTION	SNOMED_CODE	DESCRIPTION
631	QN_AMI_OTHER_GI	4711003	Congenital anomaly of bile ducts
631	QN_AMI_OTHER_GI	5153001	Ectopic anus
631	QN_AMI_OTHER_GI	5964004	Disorder of rectum
631	QN_AMI_OTHER_GI	7544003	Umbilical hernia without obstruction AND without gangrene
631	QN_AMI_OTHER_GI	7815009	Bilateral inguinal hernia with gangrene
631	QN_AMI_OTHER_GI	8114009	Diverticulosis of small intestine
631	QN_AMI_OTHER_GI	8493009	Chronic gastritis
631	QN_AMI_OTHER_GI	8579004	Projectile vomiting
631	QN_AMI_OTHER_GI	10743008	Irritable bowel syndrome
631	QN_AMI_OTHER_GI	12073000	Bilateral recurrent femoral hernia without obstruction AND without gangrene
631	QN_AMI_OTHER_GI	12776000	Foreign body in colon
631	QN_AMI_OTHER_GI	14066009	Noninfectious colitis
631	QN_AMI_OTHER_GI	14380007	Foreign body in mouth
631	QN_AMI_OTHER_GI	14760008	Constipation
631	QN_AMI_OTHER_GI	16268000	Bowel sounds absent
631	QN_AMI_OTHER_GI	16331000	Heartburn
631	QN_AMI_OTHER_GI	16761005	Esophagitis
631	QN_AMI_OTHER_GI	16932000	Nausea and vomiting
631	QN_AMI_OTHER_GI	17440005	Anal spasm
631	QN_AMI_OTHER_GI	18101008	Bowel sounds hyperactive
631	QN_AMI_OTHER_GI	18253009	Unilateral recurrent inguinal

© 2021 Matheny ME et al. *JAMA Network Open.*

CONDITIONID	CONDITION_DESCRIPTION	SNOMED_CODE	DESCRIPTION
			hernia with obstruction but no gangrene
631	QN_AMI_OTHER_GI	23387001	Perforation of esophagus
631	QN_AMI_OTHER_GI	23913003	External hemorrhoids
631	QN_AMI_OTHER_GI	24813008	Gastrointestinal complication
631	QN_AMI_OTHER_GI	25458004	Acute gastritis
631	QN_AMI_OTHER_GI	26373009	Thrombosed external hemorrhoids
631	QN_AMI_OTHER_GI	29120000	Eosinophilic colitis
631	QN_AMI_OTHER_GI	30037006	Anal fissure
631	QN_AMI_OTHER_GI	30093007	Calculus of bile duct
631	QN_AMI_OTHER_GI	31686000	Congenital anomaly of lower alimentary tract
631	QN_AMI_OTHER_GI	31704005	Residual hemorrhoidal skin tags
631	QN_AMI_OTHER_GI	33334006	Foreign body in digestive tract
631	QN_AMI_OTHER_GI	33995003	Megacolon, not Hirschsprung's
631	QN_AMI_OTHER_GI	35298007	Slow transit constipation
631	QN_AMI_OTHER_GI	35563005	Acquired diverticulum of esophagus
631	QN_AMI_OTHER_GI	36046008	Ischiorectal abscess
631	QN_AMI_OTHER_GI	37373007	Meckel's diverticulum
631	QN_AMI_OTHER_GI	37657006	Disorder of esophagus
631	QN_AMI_OTHER_GI	38996000	External hemorrhoids

© 2021 Matheny ME et al. *JAMA Network Open.*

CONDITIONID	CONDITION_DESCRIPTION	SNOMED_CODE	DESCRIPTION
			without complication
631	QN_AMI_OTHER_GI	39772007	Rectal polyp
631	QN_AMI_OTHER_GI	39839004	Diaphragmatic hernia
631	QN_AMI_OTHER_GI	40315008	Annular pancreas
631	QN_AMI_OTHER_GI	45564002	Achalasia of esophagus
631	QN_AMI_OTHER_GI	45568004	Diverticulosis of large intestine without diverticulitis
631	QN_AMI_OTHER_GI	45979003	Abdominal wind pain
631	QN_AMI_OTHER_GI	47481007	Achlorhydria
631	QN_AMI_OTHER_GI	47609003	Foreign body in esophagus
631	QN_AMI_OTHER_GI	47812002	Functional diarrhea
631	QN_AMI_OTHER_GI	49714001	Congenital anomaly of gallbladder
631	QN_AMI_OTHER_GI	51694003	Foreign body in stomach
631	QN_AMI_OTHER_GI	52232007	Chronic duodenal ileus
631	QN_AMI_OTHER_GI	52515009	Hernia of abdominal cavity
631	QN_AMI_OTHER_GI	52931009	Thrombosed internal hemorrhoids
631	QN_AMI_OTHER_GI	53313006	Hourglass contraction of stomach
631	QN_AMI_OTHER_GI	53462005	Bilateral recurrent inguinal hernia with gangrene
631	QN_AMI_OTHER_GI	53619000	Disorder of digestive system
631	QN_AMI_OTHER_GI	54051005	Cascade stomach
631	QN_AMI_OTHER_GI	54609002	Ulcer of rectum

© 2021 Matheny ME et al. *JAMA Network Open.*

CONDITIONID	CONDITION_DESCRIPTION	SNOMED_CODE	DESCRIPTION
631	QN_AMI_OTHER_GI	56165008	Diverticulitis of small intestine
631	QN_AMI_OTHER_GI	57773001	Rectal prolapse
631	QN_AMI_OTHER_GI	60002000	Hypertrophic gastritis
631	QN_AMI_OTHER_GI	60612008	Abnormal bowel sounds
631	QN_AMI_OTHER_GI	60699003	Congenital anomaly of stomach
631	QN_AMI_OTHER_GI	62120000	Unilateral recurrent inguinal hernia
631	QN_AMI_OTHER_GI	62315008	Diarrhea
631	QN_AMI_OTHER_GI	63305008	Stricture of esophagus
631	QN_AMI_OTHER_GI	63532004	Diverticula of intestine
631	QN_AMI_OTHER_GI	66135001	Bilateral inguinal hernia with obstruction but no gangrene
631	QN_AMI_OTHER_GI	66329006	Eosinophilic gastritis
631	QN_AMI_OTHER_GI	67069009	Bilateral femoral hernia without obstruction AND without gangrene
631	QN_AMI_OTHER_GI	68394001	Calculus of bile duct without obstruction
631	QN_AMI_OTHER_GI	68653001	Anal pain
631	QN_AMI_OTHER_GI	68711006	Eructation
631	QN_AMI_OTHER_GI	69518005	Congenital anomaly of digestive system
631	QN_AMI_OTHER_GI	69771008	Congenital anomaly of esophagus
631	QN_AMI_OTHER_GI	70153002	Hemorrhoids

© 2021 Matheny ME et al. *JAMA Network Open.*

CONDITIONID	CONDITION_DESCRIPTION	SNOMED_CODE	DESCRIPTION
631	QN_AMI_OTHER_GI	70342003	Cholelithiasis without obstruction
631	QN_AMI_OTHER_GI	71419002	Bilious vomiting
631	QN_AMI_OTHER_GI	71583005	Toxic gastroenteritis
631	QN_AMI_OTHER_GI	71820002	Urgent desire for stool
631	QN_AMI_OTHER_GI	72007001	Duodenitis
631	QN_AMI_OTHER_GI	72042002	Incontinence of feces
631	QN_AMI_OTHER_GI	72779005	Anorectal fistula
631	QN_AMI_OTHER_GI	72925005	Congenital cystic disease of liver
631	QN_AMI_OTHER_GI	73052009	Bilateral recurrent inguinal hernia with obstruction but no gangrene
631	QN_AMI_OTHER_GI	74829002	Persistent cloaca
631	QN_AMI_OTHER_GI	75198005	Gastric diverticulum
631	QN_AMI_OTHER_GI	75236001	Anorectal abscess
631	QN_AMI_OTHER_GI	75281002	Femoral hernia without obstruction AND without gangrene
631	QN_AMI_OTHER_GI	75955007	Thrombosed hemorrhoids
631	QN_AMI_OTHER_GI	77528005	Cholelithiasis with obstruction
631	QN_AMI_OTHER_GI	79962008	Diffuse spasm of esophagus
631	QN_AMI_OTHER_GI	80193009	Postgastric surgery syndrome
631	QN_AMI_OTHER_GI	80736008	Rectal fistula
631	QN_AMI_OTHER_GI	81120009	Functional disorder of intestine
631	QN_AMI_OTHER_GI	84231005	Inguinal hernia without obstruction AND without gangrene

© 2021 Matheny ME et al. *JAMA Network Open.*

CONDITIONID	CONDITION_DESCRIPTION	SNOMED_CODE	DESCRIPTION
631	QN_AMI_OTHER_GI	84568007	Atrophic gastritis
631	QN_AMI_OTHER_GI	85502002	Bilateral inguinal hernia
631	QN_AMI_OTHER_GI	85919009	Disorder of intestine
631	QN_AMI_OTHER_GI	85920003	Constipation by outlet obstruction
631	QN_AMI_OTHER_GI	88111009	Altered bowel function
631	QN_AMI_OTHER_GI	88580009	Anal polyp
631	QN_AMI_OTHER_GI	89057003	Leukoplakia of esophagus
631	QN_AMI_OTHER_GI	89166001	Congenital anomaly of liver
631	QN_AMI_OTHER_GI	90458007	Internal hemorrhoids
631	QN_AMI_OTHER_GI	95435007	Tracheoesophageal fistula
631	QN_AMI_OTHER_GI	111359004	Diverticulitis of colon
631	QN_AMI_OTHER_GI	111363006	Proctoptosis
631	QN_AMI_OTHER_GI	119291004	Disorder of upper gastrointestinal tract
631	QN_AMI_OTHER_GI	126764002	Congenital anomaly of intestinal tract
631	QN_AMI_OTHER_GI	128545000	Hernia of abdominal wall
631	QN_AMI_OTHER_GI	196731005	Gastroduodenitis
631	QN_AMI_OTHER_GI	196735001	Chronic superficial gastritis
631	QN_AMI_OTHER_GI	196746003	Persistent vomiting
631	QN_AMI_OTHER_GI	197011006	Radiation gastroenteritis
631	QN_AMI_OTHER_GI	197013009	Radiation enterocolitis
631	QN_AMI_OTHER_GI	197125005	Irritable bowel syndrome with diarrhea

© 2021 Matheny ME et al. *JAMA Network Open.*

CONDITIONID	CONDITION_DESCRIPTION	SNOMED_CODE	DESCRIPTION
631	QN_AMI_OTHER_GI	197130009	Vomiting after gastrointestinal tract surgery
631	QN_AMI_OTHER_GI	197151007	Acute anal fissure
631	QN_AMI_OTHER_GI	197152000	Chronic anal fissure
631	QN_AMI_OTHER_GI	197166005	Rectal abscess
631	QN_AMI_OTHER_GI	197216007	Stenosis of rectum and anus
631	QN_AMI_OTHER_GI	197402000	Calculus of bile duct with cholangitis
631	QN_AMI_OTHER_GI	204667006	Congenital diverticulum of esophagus
631	QN_AMI_OTHER_GI	204808002	Congenital pancreatic cyst
631	QN_AMI_OTHER_GI	211644003	Foreign body in anus and rectum
631	QN_AMI_OTHER_GI	235595009	Gastroesophageal reflux disease
631	QN_AMI_OTHER_GI	235599003	Eosinophilic esophagitis
631	QN_AMI_OTHER_GI	235675006	Gastroparesis syndrome
631	QN_AMI_OTHER_GI	235760009	Radiation proctitis
631	QN_AMI_OTHER_GI	235795007	Intersphincteric abscess
631	QN_AMI_OTHER_GI	235913009	Congenital disorder of gallbladder and biliary tract
631	QN_AMI_OTHER_GI	235919008	Gallstone
631	QN_AMI_OTHER_GI	235923000	Retained bile duct stone
631	QN_AMI_OTHER_GI	235977001	Congenital malformation of pancreas
631	QN_AMI_OTHER_GI	236037000	Incisional hernia
631	QN_AMI_OTHER_GI	236048007	Parastomal hernia
631	QN_AMI_OTHER_GI	249504006	Passing flatus

© 2021 Matheny ME et al. *JAMA Network Open.*

CONDITIONID	CONDITION_DESCRIPTION	SNOMED_CODE	DESCRIPTION
631	QN_AMI_OTHER_GI	249515001	Incomplete passage of stool
631	QN_AMI_OTHER_GI	249562008	Bowel finding
631	QN_AMI_OTHER_GI	249612005	Stool finding
631	QN_AMI_OTHER_GI	249643003	Healed tear of anal sphincter
631	QN_AMI_OTHER_GI	266432008	Esophageal, stomach and duodenal diseases
631	QN_AMI_OTHER_GI	266433003	Gastro-esophageal reflux disease with esophagitis
631	QN_AMI_OTHER_GI	266435005	Gastroesophageal reflux disease without esophagitis
631	QN_AMI_OTHER_GI	266451002	Allergic gastroenteritis and colitis
631	QN_AMI_OTHER_GI	266464001	Hemorrhage of rectum and anus
631	QN_AMI_OTHER_GI	268213006	Congenital abnormality of liver and/or biliary tract
631	QN_AMI_OTHER_GI	269229006	Foreign body in intestine and colon
631	QN_AMI_OTHER_GI	271832001	Flatulence, eructation and gas pain
631	QN_AMI_OTHER_GI	271835004	Abdominal distension, gaseous
631	QN_AMI_OTHER_GI	271837007	Visible peristalsis
631	QN_AMI_OTHER_GI	271837007	Visible peristalsis
631	QN_AMI_OTHER_GI	271840007	Abnormal feces
631	QN_AMI_OTHER_GI	275262008	Congenital malformation of upper alimentary tract
631	QN_AMI_OTHER_GI	282080005	Foreign body in small intestine

© 2021 Matheny ME et al. *JAMA Network Open.*

CONDITIONID	CONDITION_DESCRIPTION	SNOMED_CODE	DESCRIPTION
631	QN_AMI_OTHER_GI	300366003	Vomit contains feces
631	QN_AMI_OTHER_GI	302914006	Barrett's esophagus
631	QN_AMI_OTHER_GI	302952007	Congenital fistula of rectum and anus
631	QN_AMI_OTHER_GI	308925008	Digestive symptom
631	QN_AMI_OTHER_GI	312110005	Gallbladder and bile duct calculi
631	QN_AMI_OTHER_GI	329022007	Inguinal hernia with obstruction but no gangrene
631	QN_AMI_OTHER_GI	359804008	Eosinophilic gastroenteritis
631	QN_AMI_OTHER_GI	386211005	Disorder of function of stomach
631	QN_AMI_OTHER_GI	386617003	Digestive system finding
631	QN_AMI_OTHER_GI	387666006	Acute dilatation of stomach
631	QN_AMI_OTHER_GI	396232000	Inguinal hernia
631	QN_AMI_OTHER_GI	397881000	Diverticular disease
631	QN_AMI_OTHER_GI	398050005	Diverticular disease of colon
631	QN_AMI_OTHER_GI	399563001	Dysplasia of anus
631	QN_AMI_OTHER_GI	405247003	Obstruction of esophagus
631	QN_AMI_OTHER_GI	409656006	Acute esophagitis
631	QN_AMI_OTHER_GI	414399004	Hernia of anterior abdominal wall without obstruction AND without gangrene
631	QN_AMI_OTHER_GI	422400008	Vomiting
631	QN_AMI_OTHER_GI	422587007	Nausea
631	QN_AMI_OTHER_GI	425671009	Neurogenic bowel
631	QN_AMI_OTHER_GI	426867001	Anorectal disorder
631	QN_AMI_OTHER_GI	428722005	Recurrent inguinal hernia with gangrene

© 2021 Matheny ME et al. *JAMA Network Open.*

CONDITIONID	CONDITION_DESCRIPTION	SNOMED_CODE	DESCRIPTION
631	QN_AMI_OTHER_GI	439158007	Fecal fluid leakage
631	QN_AMI_OTHER_GI	440471007	Choledochal cyst
631	QN_AMI_OTHER_GI	442267002	Angiodysplasia of duodenum
631	QN_AMI_OTHER_GI	442405009	Ulcer of anorectal structure
631	QN_AMI_OTHER_GI	1171000119108	Recurrent inguinal hernia
631	QN_AMI_OTHER_GI	3411000119107	Gastroduodenal disorder
631	QN_AMI_OTHER_GI	5211000119107	Abscess of intestine co-occurrent and due to diverticular disease
631	QN_AMI_OTHER_GI	134021000119105	Vomiting fecal matter
631	QN_AMI_OTHER_GI	146291000119108	Vomiting without nausea
631	QN_AMI_OTHER_GI	1082661000119100	Anal abscess
631	QN_AMI_OTHER_GI	1082751000119110	Barretts esophagus with dysplasia
631	QN_AMI_OTHER_GI	1082761000119110	Barretts esophagus with high grade dysplasia
631	QN_AMI_OTHER_GI	1082771000119100	Barretts esophagus with low grade dysplasia
631	QN_AMI_OTHER_GI	1084891000119100	Cholangitis due to bile duct calculus with obstruction
631	QN_AMI_OTHER_GI	1086441000119100	Diverticulitis of small and large intestine with perforation
631	QN_AMI_OTHER_GI	1086491000119110	Perforation of small intestine due to diverticulitis

© 2021 Matheny ME et al. *JAMA Network Open.*

CONDITIONID	CONDITION_DESCRIPTION	SNOMED_CODE	DESCRIPTION
636	QN_AMI_OSTEOPOROSIS_OTHER_BONE	928000	Disorder of musculoskeletal system
636	QN_AMI_OSTEOPOROSIS_OTHER_BONE	2089002	Osteitis deformans
636	QN_AMI_OSTEOPOROSIS_OTHER_BONE	3345002	Idiopathic osteoporosis
636	QN_AMI_OSTEOPOROSIS_OTHER_BONE	4598005	Osteomalacia
636	QN_AMI_OSTEOPOROSIS_OTHER_BONE	12138000	Large head
636	QN_AMI_OSTEOPOROSIS_OTHER_BONE	16726004	Renal osteodystrophy
636	QN_AMI_OSTEOPOROSIS_OTHER_BONE	18040001	Senile osteoporosis
636	QN_AMI_OSTEOPOROSIS_OTHER_BONE	18283000	Acquired deformity of nose
636	QN_AMI_OSTEOPOROSIS_OTHER_BONE	19579005	Juvenile osteochondritis
636	QN_AMI_OSTEOPOROSIS_OTHER_BONE	28072004	Cauliflower ear
636	QN_AMI_OSTEOPOROSIS_OTHER_BONE	30128009	Tietze's disease
636	QN_AMI_OSTEOPOROSIS_OTHER_BONE	31249007	Juvenile osteochondrosis of hand
636	QN_AMI_OSTEOPOROSIS_OTHER_BONE	34713006	Vitamin D deficiency
636	QN_AMI_OSTEOPOROSIS_OTHER_BONE	36964004	Arrest of bone development AND/OR growth
636	QN_AMI_OSTEOPOROSIS_OTHER_BONE	40668007	Acquired musculoskeletal deformity
636	QN_AMI_OSTEOPOROSIS_OTHER_BONE	50642008	Complex regional pain syndrome, type I
636	QN_AMI_OSTEOPOROSIS_OTHER_BONE	50927007	Cartilage disorder
636	QN_AMI_OSTEOPOROSIS_OTHER_BONE	53174001	Disuse osteoporosis
636	QN_AMI_OSTEOPOROSIS_OTHER_BONE	53406005	Juvenile osteochondrosis of spine
636	QN_AMI_OSTEOPOROSIS_OTHER_BONE	55413008	Osteitis condensans

© 2021 Matheny ME et al. *JAMA Network Open.*

CONDITIONID	CONDITION_DESCRIPTION	SNOMED_CODE	DESCRIPTION
636	QN_AMI_OSTEOPOROSIS_OTHER_BONE	62100001	Juvenile osteochondrosis of upper extremity
636	QN_AMI_OSTEOPOROSIS_OTHER_BONE	63198006	Chondromalacia
636	QN_AMI_OSTEOPOROSIS_OTHER_BONE	64859006	Osteoporosis
636	QN_AMI_OSTEOPOROSIS_OTHER_BONE	68421004	Epiphyseal arrest
636	QN_AMI_OSTEOPOROSIS_OTHER_BONE	72082009	Acquired deformity of neck
636	QN_AMI_OSTEOPOROSIS_OTHER_BONE	72275000	Relapsing polychondritis
636	QN_AMI_OSTEOPOROSIS_OTHER_BONE	76069003	Disorder of bone
636	QN_AMI_OSTEOPOROSIS_OTHER_BONE	77881008	Osteochondropathy
636	QN_AMI_OSTEOPOROSIS_OTHER_BONE	79599002	Acquired deformity of pelvis
636	QN_AMI_OSTEOPOROSIS_OTHER_BONE	82562007	Osteochondritis dissecans
636	QN_AMI_OSTEOPOROSIS_OTHER_BONE	84062004	Juvenile osteochondrosis of carpal lunate
636	QN_AMI_OSTEOPOROSIS_OTHER_BONE	89807005	Juvenile osteochondrosis of head of metacarpals
636	QN_AMI_OSTEOPOROSIS_OTHER_BONE	89859004	Monostotic fibrous dysplasia
636	QN_AMI_OSTEOPOROSIS_OTHER_BONE	93303009	Congenital hypoplasia of zygomatic bone
636	QN_AMI_OSTEOPOROSIS_OTHER_BONE	111270009	Acquired deformity of head
636	QN_AMI_OSTEOPOROSIS_OTHER_BONE	118933004	Disorder of hand
636	QN_AMI_OSTEOPOROSIS_OTHER_BONE	118944007	Disorder of shoulder
636	QN_AMI_OSTEOPOROSIS_OTHER_BONE	128131002	Disorder of upper arm
636	QN_AMI_OSTEOPOROSIS_OTHER_BONE	128132009	Disorder of forearm
636	QN_AMI_OSTEOPOROSIS_OTHER_BONE	128135006	Disorder of thigh
636	QN_AMI_OSTEOPOROSIS_OTHER_BONE	128137003	Disorder of lower leg

© 2021 Matheny ME et al. *JAMA Network Open.*

CONDITIONID	CONDITION_DESCRIPTION	SNOMED_CODE	DESCRIPTION
636	QN_AMI_OSTEOPOROSIS_OTHER_BONE	128227001	Disorder of zygomatic bone
636	QN_AMI_OSTEOPOROSIS_OTHER_BONE	190640006	Active rickets
636	QN_AMI_OSTEOPOROSIS_OTHER_BONE	190643008	Puerperal osteomalacia
636	QN_AMI_OSTEOPOROSIS_OTHER_BONE	190644002	Senile osteomalacia
636	QN_AMI_OSTEOPOROSIS_OTHER_BONE	190646000	Adult osteomalacia due to malnutrition
636	QN_AMI_OSTEOPOROSIS_OTHER_BONE	203351003	Paget's disease of skull
636	QN_AMI_OSTEOPOROSIS_OTHER_BONE	203355007	Osteitis deformans associated with another disorder
636	QN_AMI_OSTEOPOROSIS_OTHER_BONE	203356008	Osteitis deformans in neoplastic disease
636	QN_AMI_OSTEOPOROSIS_OTHER_BONE	203357004	Hypertrophic osteoarthropathy
636	QN_AMI_OSTEOPOROSIS_OTHER_BONE	203392007	Juvenile osteochondrosis of foot
636	QN_AMI_OSTEOPOROSIS_OTHER_BONE	203411002	Osteochondritis dissecans of the wrist
636	QN_AMI_OSTEOPOROSIS_OTHER_BONE	203417003	Adult osteochondrosis of spine
636	QN_AMI_OSTEOPOROSIS_OTHER_BONE	203418008	Kienbock's disease of adults
636	QN_AMI_OSTEOPOROSIS_OTHER_BONE	203435007	Localized osteoporosis - Lequesne
636	QN_AMI_OSTEOPOROSIS_OTHER_BONE	203465002	Bone cyst
636	QN_AMI_OSTEOPOROSIS_OTHER_BONE	203467005	Solitary bone cyst
636	QN_AMI_OSTEOPOROSIS_OTHER_BONE	203468000	Aneurysmal bone cyst
636	QN_AMI_OSTEOPOROSIS_OTHER_BONE	203514008	Hypertrophy of bone
636	QN_AMI_OSTEOPOROSIS_OTHER_BONE	203517001	Complete epiphyseal arrest

© 2021 Matheny ME et al. *JAMA Network Open.*

CONDITIONID	CONDITION_DESCRIPTION	SNOMED_CODE	DESCRIPTION
636	QN_AMI_OSTEOPOROSIS_OTHER_BONE	203518006	Partial epiphyseal arrest
636	QN_AMI_OSTEOPOROSIS_OTHER_BONE	203522001	Osteolysis
636	QN_AMI_OSTEOPOROSIS_OTHER_BONE	203676006	Acquired chest and rib deformity
636	QN_AMI_OSTEOPOROSIS_OTHER_BONE	236423003	Renal impairment
636	QN_AMI_OSTEOPOROSIS_OTHER_BONE	236547007	Aluminum bone disease
636	QN_AMI_OSTEOPOROSIS_OTHER_BONE	240134003	Disorder of bone and articular cartilage
636	QN_AMI_OSTEOPOROSIS_OTHER_BONE	240139008	Chondrolysis of articular cartilage
636	QN_AMI_OSTEOPOROSIS_OTHER_BONE	240165007	Osteomalacia secondary to drug
636	QN_AMI_OSTEOPOROSIS_OTHER_BONE	240168009	Skeletal fluorosis
636	QN_AMI_OSTEOPOROSIS_OTHER_BONE	240194000	Disorder of continuity of bone
636	QN_AMI_OSTEOPOROSIS_OTHER_BONE	240226003	Acquired skeletal deformity
636	QN_AMI_OSTEOPOROSIS_OTHER_BONE	302941001	Nonunion of fracture
636	QN_AMI_OSTEOPOROSIS_OTHER_BONE	354223006	Juvenile osteochondrosis of lower extremity, excluding foot
636	QN_AMI_OSTEOPOROSIS_OTHER_BONE	371521007	Disorder of bone development
636	QN_AMI_OSTEOPOROSIS_OTHER_BONE	425852005	Fracture malunion
636	QN_AMI_OSTEOPOROSIS_OTHER_BONE	443165006	Pathological fracture due to osteoporosis
636	QN_AMI_OSTEOPOROSIS_OTHER_BONE	704330001	Pathological fracture of femur due to osteoporosis
636	QN_AMI_OSTEOPOROSIS_OTHER_BONE	704333004	Pathological fracture of hand due to osteoporosis

© 2021 Matheny ME et al. *JAMA Network Open.*

CONDITIONID	CONDITION_DESCRIPTION	SNOMED_CODE	DESCRIPTION
636	QN_AMI_OSTEOPOROSIS_OTHER_BONE	704335006	Pathological fracture of humerus due to osteoporosis
636	QN_AMI_OSTEOPOROSIS_OTHER_BONE	11311601000119100	Pathological fracture of vertebra due to osteoporosis
642	QN_AMI_IRON_DEF_OTHER_ANEMIA	10619002	Megaloblastic anemia due to poor nutrition
642	QN_AMI_IRON_DEF_OTHER_ANEMIA	17342003	Familial erythrocytosis
642	QN_AMI_IRON_DEF_OTHER_ANEMIA	19442009	Heterozygous thalassemia
642	QN_AMI_IRON_DEF_OTHER_ANEMIA	19471005	Lymphadenitis
642	QN_AMI_IRON_DEF_OTHER_ANEMIA	19636003	Monocytosis
642	QN_AMI_IRON_DEF_OTHER_ANEMIA	22996003	Splenic infarction
642	QN_AMI_IRON_DEF_OTHER_ANEMIA	23761004	Hyposplenism
642	QN_AMI_IRON_DEF_OTHER_ANEMIA	31541009	Sarcoidosis
642	QN_AMI_IRON_DEF_OTHER_ANEMIA	32035007	Chronic lymphadenitis
642	QN_AMI_IRON_DEF_OTHER_ANEMIA	37465004	Plasmacytosis
642	QN_AMI_IRON_DEF_OTHER_ANEMIA	38959009	Methemoglobinemia
642	QN_AMI_IRON_DEF_OTHER_ANEMIA	40108008	Thalassemia
642	QN_AMI_IRON_DEF_OTHER_ANEMIA	44865000	Secondary polycythemia
642	QN_AMI_IRON_DEF_OTHER_ANEMIA	48813009	Lymphocytopenia
642	QN_AMI_IRON_DEF_OTHER_ANEMIA	49472006	Megaloblastic anemia due to vitamin B>12< deficiency
642	QN_AMI_IRON_DEF_OTHER_ANEMIA	49708008	Anemia of chronic renal failure
642	QN_AMI_IRON_DEF_OTHER_ANEMIA	51244008	Disorder of spleen
642	QN_AMI_IRON_DEF_OTHER_ANEMIA	52565000	Non megaloblastic anemia associated with nutritional deficiency

© 2021 Matheny ME et al. *JAMA Network Open.*

CONDITIONID	CONDITION_DESCRIPTION	SNOMED_CODE	DESCRIPTION
642	QN_AMI_IRON_DEF_OTHER_ANEMIA	52951008	Congenital dyserythropoietic anemia
642	QN_AMI_IRON_DEF_OTHER_ANEMIA	53165003	Megaloblastic anemia
642	QN_AMI_IRON_DEF_OTHER_ANEMIA	54097007	White blood cell disorder
642	QN_AMI_IRON_DEF_OTHER_ANEMIA	56478004	Leukemoid reaction
642	QN_AMI_IRON_DEF_OTHER_ANEMIA	58381000	Hypersplenism
642	QN_AMI_IRON_DEF_OTHER_ANEMIA	58639003	Doan-Wiseman syndrome
642	QN_AMI_IRON_DEF_OTHER_ANEMIA	64757003	Lymph node sarcoidosis
642	QN_AMI_IRON_DEF_OTHER_ANEMIA	65209002	Basophilia
642	QN_AMI_IRON_DEF_OTHER_ANEMIA	65209002	Basophilia
642	QN_AMI_IRON_DEF_OTHER_ANEMIA	66612000	Nutritional anemia
642	QN_AMI_IRON_DEF_OTHER_ANEMIA	67023009	Lymphocytosis
642	QN_AMI_IRON_DEF_OTHER_ANEMIA	79040006	Splenic cyst
642	QN_AMI_IRON_DEF_OTHER_ANEMIA	80126007	Plummer-Vinson syndrome
642	QN_AMI_IRON_DEF_OTHER_ANEMIA	82053000	Splenic abscess
642	QN_AMI_IRON_DEF_OTHER_ANEMIA	84828003	Leukopenia
642	QN_AMI_IRON_DEF_OTHER_ANEMIA	85649008	Megaloblastic anemia due to folate deficiency
642	QN_AMI_IRON_DEF_OTHER_ANEMIA	85746008	Anemia due to protein deficiency
642	QN_AMI_IRON_DEF_OTHER_ANEMIA	87522002	Iron deficiency anemia
642	QN_AMI_IRON_DEF_OTHER_ANEMIA	111583006	Leukocytosis
642	QN_AMI_IRON_DEF_OTHER_ANEMIA	127388009	Hypergammaglobulinemia
642	QN_AMI_IRON_DEF_OTHER_ANEMIA	191142007	Vitamin B12 deficiency anemia due to malabsorption with proteinuria
642	QN_AMI_IRON_DEF_OTHER_ANEMIA	191148006	Folate deficiency anemia, drug-induced

© 2021 Matheny ME et al. *JAMA Network Open.*

CONDITIONID	CONDITION_DESCRIPTION	SNOMED_CODE	DESCRIPTION
642	QN_AMI_IRON_DEF_OTHER_ANEMIA	191156009	Protein-deficiency anemia
642	QN_AMI_IRON_DEF_OTHER_ANEMIA	191161006	Vitamin C deficiency anemia
642	QN_AMI_IRON_DEF_OTHER_ANEMIA	191265009	Anemia in neoplastic disease
642	QN_AMI_IRON_DEF_OTHER_ANEMIA	191382009	Chronic congestive splenomegaly
642	QN_AMI_IRON_DEF_OTHER_ANEMIA	234347009	Anemia of chronic disease
642	QN_AMI_IRON_DEF_OTHER_ANEMIA	237934001	Transcobalamin II deficiency
642	QN_AMI_IRON_DEF_OTHER_ANEMIA	267513007	Deficiency anemias
642	QN_AMI_IRON_DEF_OTHER_ANEMIA	267517008	Vitamin B12 deficiency anemia due to dietary causes
642	QN_AMI_IRON_DEF_OTHER_ANEMIA	267530009	Acute posthemorrhagic anemia
642	QN_AMI_IRON_DEF_OTHER_ANEMIA	267548000	Nonspecific mesenteric adenitis
642	QN_AMI_IRON_DEF_OTHER_ANEMIA	267550008	Congenital methemoglobinemia
642	QN_AMI_IRON_DEF_OTHER_ANEMIA	271737000	Anemia
642	QN_AMI_IRON_DEF_OTHER_ANEMIA	371315009	Iron deficiency anemia secondary to inadequate dietary iron intake
642	QN_AMI_IRON_DEF_OTHER_ANEMIA	413533008	Anemia due to chronic blood loss
642	QN_AMI_IRON_DEF_OTHER_ANEMIA	414027002	Disorder of hematopoietic structure
642	QN_AMI_IRON_DEF_OTHER_ANEMIA	418010004	Increased blood lymphocyte number

© 2021 Matheny ME et al. *JAMA Network Open.*

CONDITIONID	CONDITION_DESCRIPTION	SNOMED_CODE	DESCRIPTION
642	QN_AMI_IRON_DEF_OTHER_ANEMIA	419455006	Disorder characterized by eosinophilia
642	QN_AMI_IRON_DEF_OTHER_ANEMIA	429564000	Anemia due to chemotherapy
642	QN_AMI_IRON_DEF_OTHER_ANEMIA	442113000	Band neutrophil count above reference range
642	QN_AMI_IRON_DEF_OTHER_ANEMIA	442113000	Band neutrophil count above reference range
642	QN_AMI_IRON_DEF_OTHER_ANEMIA	707323002	Anemia in chronic kidney disease
643	QN_AMI_DELIRIUM_ENCEPH	2776000	Delirium
643	QN_AMI_DELIRIUM_ENCEPH	5510009	Organic delusional disorder
643	QN_AMI_DELIRIUM_ENCEPH	7011001	Hallucinations
643	QN_AMI_DELIRIUM_ENCEPH	17496003	Organic anxiety disorder
643	QN_AMI_DELIRIUM_ENCEPH	18260003	Postpartum psychosis
643	QN_AMI_DELIRIUM_ENCEPH	23645006	Organic mood disorder
643	QN_AMI_DELIRIUM_ENCEPH	25762009	Visual agnosia
643	QN_AMI_DELIRIUM_ENCEPH	28394000	Toxic encephalopathy
643	QN_AMI_DELIRIUM_ENCEPH	42594001	Organic mood disorder of depressed type
643	QN_AMI_DELIRIUM_ENCEPH	45150006	Auditory hallucinations
643	QN_AMI_DELIRIUM_ENCEPH	45912004	Organic hallucinosis
643	QN_AMI_DELIRIUM_ENCEPH	46206005	Mood disorder
643	QN_AMI_DELIRIUM_ENCEPH	50122000	Metabolic encephalopathy
643	QN_AMI_DELIRIUM_ENCEPH	58329000	Organic mood disorder of manic type
643	QN_AMI_DELIRIUM_ENCEPH	64269007	Visual hallucinations

© 2021 Matheny ME et al. *JAMA Network Open.*

CONDITIONID	CONDITION_DESCRIPTION	SNOMED_CODE	DESCRIPTION
643	QN_AMI_DELIRIUM_ENCEPH	70042006	Psychophysical visual disturbance
643	QN_AMI_DELIRIUM_ENCEPH	81308009	Disorder of brain
643	QN_AMI_DELIRIUM_ENCEPH	111479008	Organic mental disorder
643	QN_AMI_DELIRIUM_ENCEPH	191447007	Organic psychotic condition
643	QN_AMI_DELIRIUM_ENCEPH	191507002	Subacute delirium
643	QN_AMI_DELIRIUM_ENCEPH	192730008	Toxic encephalitis
643	QN_AMI_DELIRIUM_ENCEPH	231442003	Organic catatonic disorder
643	QN_AMI_DELIRIUM_ENCEPH	428703001	Transient organic mental disorder
643	QN_AMI_DELIRIUM_ENCEPH	450886002	Posterior reversible encephalopathy syndrome
644	QN_AMI_DEMENTIA_W_COMP	12348006	Presenile dementia
644	QN_AMI_DEMENTIA_W_COMP	30753002	Normal pressure hydrocephalus
644	QN_AMI_DEMENTIA_W_COMP	52522001	Degenerative brain disorder
644	QN_AMI_DEMENTIA_W_COMP	191452002	Presenile dementia with delirium
644	QN_AMI_DEMENTIA_W_COMP	191455000	Presenile dementia with depression
644	QN_AMI_DEMENTIA_W_COMP	191457008	Senile dementia with depressive or paranoid features
644	QN_AMI_DEMENTIA_W_COMP	191459006	Senile dementia with depression
644	QN_AMI_DEMENTIA_W_COMP	191461002	Senile dementia with delirium
644	QN_AMI_DEMENTIA_W_COMP	191464005	Arteriosclerotic dementia with delirium
644	QN_AMI_DEMENTIA_W_COMP	191465006	Arteriosclerotic dementia with paranoia
644	QN_AMI_DEMENTIA_W_COMP	191466007	Arteriosclerotic dementia with depression

CONDITIONID	CONDITION_DESCRIPTION	SNOMED_CODE	DESCRIPTION
644	QN_AMI_DEMENTIA_W_COMP	191519005	Dementia associated with another disease
644	QN_AMI_DEMENTIA_W_COMP	230745008	Hydrocephalus
644	QN_AMI_DEMENTIA_W_COMP	230746009	Obstructive hydrocephalus
644	QN_AMI_DEMENTIA_W_COMP	230751003	Post-traumatic hydrocephalus
644	QN_AMI_DEMENTIA_W_COMP	271569006	Communicating hydrocephalus
644	QN_AMI_DEMENTIA_W_COMP	1591000119103	Dementia with behavioral disturbance
645	QN_AMI_DEMENTIA_WO_COMP	792004	Creutzfeldt-Jakob disease
645	QN_AMI_DEMENTIA_WO_COMP	2584003	Cerebral degeneration in childhood
645	QN_AMI_DEMENTIA_WO_COMP	3298001	Amnestic disorder
645	QN_AMI_DEMENTIA_WO_COMP	12348006	Presenile dementia
645	QN_AMI_DEMENTIA_WO_COMP	13092008	Pick's disease
645	QN_AMI_DEMENTIA_WO_COMP	16517004	Cerebral lipidosis
645	QN_AMI_DEMENTIA_WO_COMP	18842008	Corticobasal degeneration
645	QN_AMI_DEMENTIA_WO_COMP	20415001	Progressive sclerosing poliodystrophy
645	QN_AMI_DEMENTIA_WO_COMP	22255007	Progressive multifocal leukoencephalopathy
645	QN_AMI_DEMENTIA_WO_COMP	23849003	Sandhoff disease
645	QN_AMI_DEMENTIA_WO_COMP	26929004	Alzheimer's disease
645	QN_AMI_DEMENTIA_WO_COMP	29570005	Leigh's disease
645	QN_AMI_DEMENTIA_WO_COMP	33316007	GM 2 gangliosidosis
645	QN_AMI_DEMENTIA_WO_COMP	42012007	Neuronal ceroid lipofuscinosis
645	QN_AMI_DEMENTIA_WO_COMP	45864009	Senile degeneration of brain

© 2021 Matheny ME et al. *JAMA Network Open.*

CONDITIONID	CONDITION_DESCRIPTION	SNOMED_CODE	DESCRIPTION
645	QN_AMI_DEMENTIA_WO_COMP	50967008	Gangliosidosis
645	QN_AMI_DEMENTIA_WO_COMP	52448006	Dementia
645	QN_AMI_DEMENTIA_WO_COMP	52522001	Degenerative brain disorder
645	QN_AMI_DEMENTIA_WO_COMP	67155006	Gerstmann-Straussler-Scheinker syndrome
645	QN_AMI_DEMENTIA_WO_COMP	74351001	Reye's syndrome
645	QN_AMI_DEMENTIA_WO_COMP	80098002	Diffuse Lewy body disease
645	QN_AMI_DEMENTIA_WO_COMP	83157008	Fatal familial insomnia
645	QN_AMI_DEMENTIA_WO_COMP	86188000	Kuru
645	QN_AMI_DEMENTIA_WO_COMP	90099008	Subcortical leukoencephalopathy
645	QN_AMI_DEMENTIA_WO_COMP	111384001	Ganglioside sialidase deficiency
645	QN_AMI_DEMENTIA_WO_COMP	111385000	Tay-Sachs disease
645	QN_AMI_DEMENTIA_WO_COMP	111479008	Organic mental disorder
645	QN_AMI_DEMENTIA_WO_COMP	186482006	Slow viral central nervous system infection
645	QN_AMI_DEMENTIA_WO_COMP	191519005	Dementia associated with another disease
645	QN_AMI_DEMENTIA_WO_COMP	192685000	Subacute sclerosing panencephalitis
645	QN_AMI_DEMENTIA_WO_COMP	192782005	Galactosylceramide beta-galactosidase deficiency
645	QN_AMI_DEMENTIA_WO_COMP	230270009	Frontotemporal dementia
645	QN_AMI_DEMENTIA_WO_COMP	231438001	Presbyophrenic psychosis
645	QN_AMI_DEMENTIA_WO_COMP	238028008	Sphingolipidosis

© 2021 Matheny ME et al. *JAMA Network Open.*

CONDITIONID	CONDITION_DESCRIPTION	SNOMED_CODE	DESCRIPTION
645	QN_AMI_DEMENTIA_WO_COMP	278857002	Dementia of frontal lobe type
645	QN_AMI_DEMENTIA_WO_COMP	279982005	Cerebral degeneration presenting primarily with dementia
645	QN_AMI_DEMENTIA_WO_COMP	304603007	Variant Creutzfeldt-Jakob disease
645	QN_AMI_DEMENTIA_WO_COMP	361273006	Alcoholic cerebellar degeneration
645	QN_AMI_DEMENTIA_WO_COMP	396338004	Metachromatic leukodystrophy
645	QN_AMI_DEMENTIA_WO_COMP	416780008	Primary degenerative dementia of the Alzheimer type, presenile onset
645	QN_AMI_DEMENTIA_WO_COMP	416975007	Primary degenerative dementia of the Alzheimer type, senile onset
645	QN_AMI_DEMENTIA_WO_COMP	420146005	Cerebral degeneration associated with generalized lipidosis
645	QN_AMI_DEMENTIA_WO_COMP	429998004	Vascular dementia
647	QN_AMI_DRUG_ETOH_W_PSYCHOSIS	281004	Dementia associated with alcoholism
647	QN_AMI_DRUG_ETOH_W_PSYCHOSIS	1973000	Sedative, hypnotic AND/OR anxiolytic-induced psychotic disorder with delusions

© 2021 Matheny ME et al. *JAMA Network Open.*

CONDITIONID	CONDITION_DESCRIPTION	SNOMED_CODE	DESCRIPTION
647	QN_AMI_DRUG_ETOH_W_PSYCHOSIS	2403008	Psychoactive substance dependence
647	QN_AMI_DRUG_ETOH_W_PSYCHOSIS	5002000	Inhalant dependence
647	QN_AMI_DRUG_ETOH_W_PSYCHOSIS	5602001	Opioid abuse
647	QN_AMI_DRUG_ETOH_W_PSYCHOSIS	6348008	Cocaine-induced psychotic disorder with hallucinations
647	QN_AMI_DRUG_ETOH_W_PSYCHOSIS	7052005	Alcohol hallucinosis
647	QN_AMI_DRUG_ETOH_W_PSYCHOSIS	8635005	Alcohol withdrawal delirium
647	QN_AMI_DRUG_ETOH_W_PSYCHOSIS	11061003	Psychoactive substance use disorder
647	QN_AMI_DRUG_ETOH_W_PSYCHOSIS	11387009	Psychoactive substance-induced organic mental disorder
647	QN_AMI_DRUG_ETOH_W_PSYCHOSIS	14784000	Opioid-induced organic mental disorder
647	QN_AMI_DRUG_ETOH_W_PSYCHOSIS	19445006	Opioid-induced psychotic disorder with hallucinations
647	QN_AMI_DRUG_ETOH_W_PSYCHOSIS	20385005	Opioid-induced psychotic disorder with delusions
647	QN_AMI_DRUG_ETOH_W_PSYCHOSIS	21000000	Idiosyncratic intoxication
647	QN_AMI_DRUG_ETOH_W_PSYCHOSIS	25508008	Pathological drug intoxication
647	QN_AMI_DRUG_ETOH_W_PSYCHOSIS	26714005	Cannabis-induced psychotic disorder with hallucinations
647	QN_AMI_DRUG_ETOH_W_PSYCHOSIS	29212009	Alcohol-induced organic mental disorder
647	QN_AMI_DRUG_ETOH_W_PSYCHOSIS	30491001	Cocaine delusional disorder

© 2021 Matheny ME et al. *JAMA Network Open.*

CONDITIONID	CONDITION_DESCRIPTION	SNOMED_CODE	DESCRIPTION
647	QN_AMI_DRUG_ETOH_W_PSYCHOSIS	31956009	Cocaine dependence
647	QN_AMI_DRUG_ETOH_W_PSYCHOSIS	32009006	Hallucinogen hallucinosis
647	QN_AMI_DRUG_ETOH_W_PSYCHOSIS	32552001	Psychoactive substance-induced organic delusional disorder
647	QN_AMI_DRUG_ETOH_W_PSYCHOSIS	32875003	Inhalant-induced persisting dementia
647	QN_AMI_DRUG_ETOH_W_PSYCHOSIS	33871004	Phencyclidine-induced psychotic disorder with hallucinations
647	QN_AMI_DRUG_ETOH_W_PSYCHOSIS	37344009	Cannabis abuse
647	QN_AMI_DRUG_ETOH_W_PSYCHOSIS	38247002	Hallucinogen dependence
647	QN_AMI_DRUG_ETOH_W_PSYCHOSIS	41083005	Alcohol-induced sleep disorder
647	QN_AMI_DRUG_ETOH_W_PSYCHOSIS	42344001	Alcohol-induced psychosis
647	QN_AMI_DRUG_ETOH_W_PSYCHOSIS	46975003	Cocaine-induced organic mental disorder
647	QN_AMI_DRUG_ETOH_W_PSYCHOSIS	47664006	Sedative, hypnotic AND/OR anxiolytic-induced psychotic disorder with hallucinations
647	QN_AMI_DRUG_ETOH_W_PSYCHOSIS	50933003	Hallucinogen delusional disorder
647	QN_AMI_DRUG_ETOH_W_PSYCHOSIS	53050002	Hallucinogen-induced organic mental disorder
647	QN_AMI_DRUG_ETOH_W_PSYCHOSIS	53936005	Alcohol-induced mood disorder
647	QN_AMI_DRUG_ETOH_W_PSYCHOSIS	59651006	Sedative, hypnotic AND/OR anxiolytic-induced persisting dementia

© 2021 Matheny ME et al. *JAMA Network Open.*

CONDITIONID	CONDITION_DESCRIPTION	SNOMED_CODE	DESCRIPTION
647	QN_AMI_DRUG_ETOH_W_PSYCHOSIS	61104008	Inhalant-induced organic mental disorder
647	QN_AMI_DRUG_ETOH_W_PSYCHOSIS	61144001	Alcohol-induced psychotic disorder with delusions
647	QN_AMI_DRUG_ETOH_W_PSYCHOSIS	63649001	Cannabis delusional disorder
647	QN_AMI_DRUG_ETOH_W_PSYCHOSIS	63983005	Inhalant-induced psychotic disorder with hallucinations
647	QN_AMI_DRUG_ETOH_W_PSYCHOSIS	70340006	Inhalant abuse
647	QN_AMI_DRUG_ETOH_W_PSYCHOSIS	73097000	Alcohol amnestic disorder
647	QN_AMI_DRUG_ETOH_W_PSYCHOSIS	74851005	Hallucinogen abuse
647	QN_AMI_DRUG_ETOH_W_PSYCHOSIS	74934004	Psychoactive substance-induced withdrawal syndrome
647	QN_AMI_DRUG_ETOH_W_PSYCHOSIS	75122001	Inhalant-induced psychotic disorder with delusions
647	QN_AMI_DRUG_ETOH_W_PSYCHOSIS	75544000	Opioid dependence
647	QN_AMI_DRUG_ETOH_W_PSYCHOSIS	77355000	Cannabis-induced organic mental disorder
647	QN_AMI_DRUG_ETOH_W_PSYCHOSIS	78267003	Cocaine abuse
647	QN_AMI_DRUG_ETOH_W_PSYCHOSIS	83168008	Psychoactive substance-induced organic amnestic disorder
647	QN_AMI_DRUG_ETOH_W_PSYCHOSIS	85005007	Cannabis dependence
647	QN_AMI_DRUG_ETOH_W_PSYCHOSIS	91388009	Psychoactive substance abuse
647	QN_AMI_DRUG_ETOH_W_PSYCHOSIS	111480006	Psychoactive substance-induced organic dementia
647	QN_AMI_DRUG_ETOH_W_PSYCHOSIS	191480000	Alcohol withdrawal syndrome

© 2021 Matheny ME et al. *JAMA Network Open.*

CONDITIONID	CONDITION_DESCRIPTION	SNOMED_CODE	DESCRIPTION
647	QN_AMI_DRUG_ETOH_W_PSYCHOSIS	191483003	Drug-induced psychosis
647	QN_AMI_DRUG_ETOH_W_PSYCHOSIS	191486006	Drug-induced hallucinosis
647	QN_AMI_DRUG_ETOH_W_PSYCHOSIS	191492000	Drug-induced delirium
647	QN_AMI_DRUG_ETOH_W_PSYCHOSIS	191493005	Drug-induced dementia
647	QN_AMI_DRUG_ETOH_W_PSYCHOSIS	191494004	Drug-induced amnestic syndrome
647	QN_AMI_DRUG_ETOH_W_PSYCHOSIS	191496002	Drug-induced personality disorder
647	QN_AMI_DRUG_ETOH_W_PSYCHOSIS	268640002	Hypnotic or anxiolytic dependence
647	QN_AMI_DRUG_ETOH_W_PSYCHOSIS	301643003	Sedative, hypnotic AND/OR anxiolytic-induced persisting amnestic disorder
647	QN_AMI_DRUG_ETOH_W_PSYCHOSIS	312098001	Sedative, hypnotic AND/OR anxiolytic-related disorder
647	QN_AMI_DRUG_ETOH_W_PSYCHOSIS	361150008	Sedative, hypnotic AND/OR anxiolytic withdrawal delirium
647	QN_AMI_DRUG_ETOH_W_PSYCHOSIS	363101005	Drug withdrawal
647	QN_AMI_DRUG_ETOH_W_PSYCHOSIS	418475009	Drug-induced sleep disorder
647	QN_AMI_DRUG_ETOH_W_PSYCHOSIS	427975003	Drug-induced delusional disorder
647	QN_AMI_DRUG_ETOH_W_PSYCHOSIS	429672007	Drug-induced mood disorder
647	QN_AMI_DRUG_ETOH_W_PSYCHOSIS	441527004	Stimulant abuse
647	QN_AMI_DRUG_ETOH_W_PSYCHOSIS	442351006	Mental disorder due to drug
647	QN_AMI_DRUG_ETOH_W_PSYCHOSIS	442406005	Stimulant dependence

© 2021 Matheny ME et al. *JAMA Network Open.*

CONDITIONID	CONDITION_DESCRIPTION	SNOMED_CODE	DESCRIPTION
648	QN_AMI_DRUG_ETOH_DEPEND	1383008	Hallucinogen mood disorder
648	QN_AMI_DRUG_ETOH_DEPEND	1686006	Sedative, hypnotic AND/OR anxiolytic-induced anxiety disorder
648	QN_AMI_DRUG_ETOH_DEPEND	2403008	Psychoactive substance dependence
648	QN_AMI_DRUG_ETOH_DEPEND	5002000	Inhalant dependence
648	QN_AMI_DRUG_ETOH_DEPEND	5444000	Sedative, hypnotic AND/OR anxiolytic intoxication delirium
648	QN_AMI_DRUG_ETOH_DEPEND	5602001	Opioid abuse
648	QN_AMI_DRUG_ETOH_DEPEND	7895008	Poisoning by drug AND/OR medicinal substance
648	QN_AMI_DRUG_ETOH_DEPEND	8686000	Cocaine-induced sexual dysfunction
648	QN_AMI_DRUG_ETOH_DEPEND	10028000	Uncomplicated sedative, hypnotic AND/OR anxiolytic withdrawal
648	QN_AMI_DRUG_ETOH_DEPEND	10327003	Cocaine-induced mood disorder
648	QN_AMI_DRUG_ETOH_DEPEND	11061003	Psychoactive substance use disorder
648	QN_AMI_DRUG_ETOH_DEPEND	11387009	Psychoactive substance-induced organic mental disorder
648	QN_AMI_DRUG_ETOH_DEPEND	12380008	Sedative, hypnotic AND/OR anxiolytic-induced sexual dysfunction
648	QN_AMI_DRUG_ETOH_DEPEND	14784000	Opioid-induced organic mental disorder

© 2021 Matheny ME et al. *JAMA Network Open.*

CONDITIONID	CONDITION_DESCRIPTION	SNOMED_CODE	DESCRIPTION
648	QN_AMI_DRUG_ETOH_DEPEND	15167005	Alcohol abuse
648	QN_AMI_DRUG_ETOH_DEPEND	15277004	Hallucinogen-induced anxiety disorder
648	QN_AMI_DRUG_ETOH_DEPEND	18653004	Alcohol intoxication delirium
648	QN_AMI_DRUG_ETOH_DEPEND	18689007	Inhalant intoxication delirium
648	QN_AMI_DRUG_ETOH_DEPEND	20876004	Inhalant-induced anxiety disorder
648	QN_AMI_DRUG_ETOH_DEPEND	22574000	Cocaine-induced sleep disorder
648	QN_AMI_DRUG_ETOH_DEPEND	23527004	Cannabis intoxication
648	QN_AMI_DRUG_ETOH_DEPEND	25508008	Pathological drug intoxication
648	QN_AMI_DRUG_ETOH_DEPEND	25702006	Alcohol intoxication
648	QN_AMI_DRUG_ETOH_DEPEND	27956007	Cocaine intoxication
648	QN_AMI_DRUG_ETOH_DEPEND	28864000	Sedative, hypnotic AND/OR anxiolytic-induced mood disorder
648	QN_AMI_DRUG_ETOH_DEPEND	29212009	Alcohol-induced organic mental disorder
648	QN_AMI_DRUG_ETOH_DEPEND	29733004	Opioid-induced mood disorder
648	QN_AMI_DRUG_ETOH_DEPEND	31956009	Cocaine dependence
648	QN_AMI_DRUG_ETOH_DEPEND	34938008	Alcohol-induced anxiety disorder
648	QN_AMI_DRUG_ETOH_DEPEND	37331004	Psychoactive substance-induced organic mood disorder
648	QN_AMI_DRUG_ETOH_DEPEND	37344009	Cannabis abuse

© 2021 Matheny ME et al. *JAMA Network Open.*

CONDITIONID	CONDITION_DESCRIPTION	SNOMED_CODE	DESCRIPTION
648	QN_AMI_DRUG_ETOH_DEPEND	38247002	Hallucinogen dependence
648	QN_AMI_DRUG_ETOH_DEPEND	39003006	Psychoactive substance-induced organic delirium
648	QN_AMI_DRUG_ETOH_DEPEND	39807006	Cannabis intoxication delirium
648	QN_AMI_DRUG_ETOH_DEPEND	39951001	Cannabis-induced anxiety disorder
648	QN_AMI_DRUG_ETOH_DEPEND	40571009	Hallucinogen intoxication delirium
648	QN_AMI_DRUG_ETOH_DEPEND	41083005	Alcohol-induced sleep disorder
648	QN_AMI_DRUG_ETOH_DEPEND	46975003	Cocaine-induced organic mental disorder
648	QN_AMI_DRUG_ETOH_DEPEND	50026000	Psychoactive substance-induced organic anxiety disorder
648	QN_AMI_DRUG_ETOH_DEPEND	50320000	Hallucinogen intoxication
648	QN_AMI_DRUG_ETOH_DEPEND	51493001	Cocaine-induced anxiety disorder
648	QN_AMI_DRUG_ETOH_DEPEND	52866005	Opioid intoxication delirium
648	QN_AMI_DRUG_ETOH_DEPEND	53050002	Hallucinogen-induced organic mental disorder
648	QN_AMI_DRUG_ETOH_DEPEND	53936005	Alcohol-induced mood disorder
648	QN_AMI_DRUG_ETOH_DEPEND	57588009	Sedative, hypnotic AND/OR anxiolytic-induced sleep disorder
648	QN_AMI_DRUG_ETOH_DEPEND	60901005	Inhalant intoxication

© 2021 Matheny ME et al. *JAMA Network Open.*

CONDITIONID	CONDITION_DESCRIPTION	SNOMED_CODE	DESCRIPTION
648	QN_AMI_DRUG_ETOH_DEPEND	61104008	Inhalant-induced organic mental disorder
648	QN_AMI_DRUG_ETOH_DEPEND	64386003	Sedative abuse
648	QN_AMI_DRUG_ETOH_DEPEND	66590003	Alcohol dependence
648	QN_AMI_DRUG_ETOH_DEPEND	70328006	Cocaine delirium
648	QN_AMI_DRUG_ETOH_DEPEND	70340006	Inhalant abuse
648	QN_AMI_DRUG_ETOH_DEPEND	71328000	Opioid-induced sexual dysfunction
648	QN_AMI_DRUG_ETOH_DEPEND	74851005	Hallucinogen abuse
648	QN_AMI_DRUG_ETOH_DEPEND	74934004	Psychoactive substance-induced withdrawal syndrome
648	QN_AMI_DRUG_ETOH_DEPEND	75544000	Opioid dependence
648	QN_AMI_DRUG_ETOH_DEPEND	77355000	Cannabis-induced organic mental disorder
648	QN_AMI_DRUG_ETOH_DEPEND	77721001	Opioid intoxication
648	QN_AMI_DRUG_ETOH_DEPEND	78267003	Cocaine abuse
648	QN_AMI_DRUG_ETOH_DEPEND	78524005	Alcohol-induced sexual dysfunction
648	QN_AMI_DRUG_ETOH_DEPEND	80868005	Cocaine withdrawal
648	QN_AMI_DRUG_ETOH_DEPEND	85005007	Cannabis dependence
648	QN_AMI_DRUG_ETOH_DEPEND	87132004	Opioid withdrawal
648	QN_AMI_DRUG_ETOH_DEPEND	88926005	Opioid-induced sleep disorder
648	QN_AMI_DRUG_ETOH_DEPEND	89451009	Inhalant-induced mood disorder
648	QN_AMI_DRUG_ETOH_DEPEND	91388009	Psychoactive substance abuse
648	QN_AMI_DRUG_ETOH_DEPEND	191480000	Alcohol withdrawal syndrome
648	QN_AMI_DRUG_ETOH_DEPEND	191492000	Drug-induced delirium
648	QN_AMI_DRUG_ETOH_DEPEND	191802004	Acute alcoholic intoxication in alcoholism

© 2021 Matheny ME et al. *JAMA Network Open.*

CONDITIONID	CONDITION_DESCRIPTION	SNOMED_CODE	DESCRIPTION
648	QN_AMI_DRUG_ETOH_DEPEND	191804003	Continuous acute alcoholic intoxication in alcoholism
648	QN_AMI_DRUG_ETOH_DEPEND	191805002	Episodic acute alcoholic intoxication in alcoholism
648	QN_AMI_DRUG_ETOH_DEPEND	191806001	Acute alcoholic intoxication in remission, in alcoholism
648	QN_AMI_DRUG_ETOH_DEPEND	191811004	Continuous chronic alcoholism
648	QN_AMI_DRUG_ETOH_DEPEND	191812006	Episodic chronic alcoholism
648	QN_AMI_DRUG_ETOH_DEPEND	191813001	Chronic alcoholism in remission
648	QN_AMI_DRUG_ETOH_DEPEND	191816009	Drug dependence
648	QN_AMI_DRUG_ETOH_DEPEND	191819002	Continuous opioid dependence
648	QN_AMI_DRUG_ETOH_DEPEND	191820008	Episodic opioid dependence
648	QN_AMI_DRUG_ETOH_DEPEND	191821007	Opioid dependence in remission
648	QN_AMI_DRUG_ETOH_DEPEND	191825003	Hypnotic or anxiolytic dependence, continuous
648	QN_AMI_DRUG_ETOH_DEPEND	191826002	Hypnotic or anxiolytic dependence, episodic
648	QN_AMI_DRUG_ETOH_DEPEND	191827006	Hypnotic or anxiolytic dependence in remission
648	QN_AMI_DRUG_ETOH_DEPEND	191831000	Cocaine dependence, continuous

© 2021 Matheny ME et al. *JAMA Network Open.*

CONDITIONID	CONDITION_DESCRIPTION	SNOMED_CODE	DESCRIPTION
648	QN_AMI_DRUG_ETOH_DEPEND	191832007	Cocaine dependence, episodic
648	QN_AMI_DRUG_ETOH_DEPEND	191833002	Cocaine dependence in remission
648	QN_AMI_DRUG_ETOH_DEPEND	191837001	Cannabis dependence, continuous
648	QN_AMI_DRUG_ETOH_DEPEND	191838006	Cannabis dependence, episodic
648	QN_AMI_DRUG_ETOH_DEPEND	191839003	Cannabis dependence in remission
648	QN_AMI_DRUG_ETOH_DEPEND	191843004	Amphetamine or psychostimulant dependence, continuous
648	QN_AMI_DRUG_ETOH_DEPEND	191844005	Amphetamine or psychostimulant dependence, episodic
648	QN_AMI_DRUG_ETOH_DEPEND	191845006	Amphetamine or psychostimulant dependence in remission
648	QN_AMI_DRUG_ETOH_DEPEND	191849000	Hallucinogen dependence, continuous
648	QN_AMI_DRUG_ETOH_DEPEND	191850000	Hallucinogen dependence, episodic
648	QN_AMI_DRUG_ETOH_DEPEND	191851001	Hallucinogen dependence in remission
648	QN_AMI_DRUG_ETOH_DEPEND	191865004	Combined opioid with other drug dependence
648	QN_AMI_DRUG_ETOH_DEPEND	191867007	Combined opioid with other drug

© 2021 Matheny ME et al. *JAMA Network Open.*

CONDITIONID	CONDITION_DESCRIPTION	SNOMED_CODE	DESCRIPTION
			dependence, continuous
648	QN_AMI_DRUG_ETOH_DEPEND	191868002	Combined opioid with other drug dependence, episodic
648	QN_AMI_DRUG_ETOH_DEPEND	191869005	Combined opioid with other drug dependence in remission
648	QN_AMI_DRUG_ETOH_DEPEND	191871005	Combined drug dependence, excluding opioids
648	QN_AMI_DRUG_ETOH_DEPEND	191873008	Combined drug dependence, excluding opioid, continuous
648	QN_AMI_DRUG_ETOH_DEPEND	191874002	Combined drug dependence, excluding opioid, episodic
648	QN_AMI_DRUG_ETOH_DEPEND	191875001	Combined drug dependence, excluding opioid, in remission
648	QN_AMI_DRUG_ETOH_DEPEND	268640002	Hypnotic or anxiolytic dependence
648	QN_AMI_DRUG_ETOH_DEPEND	312098001	Sedative, hypnotic AND/OR anxiolytic-related disorder
648	QN_AMI_DRUG_ETOH_DEPEND	363101005	Drug withdrawal
648	QN_AMI_DRUG_ETOH_DEPEND	396344000	Psychoactive substance-induced organic intoxication
648	QN_AMI_DRUG_ETOH_DEPEND	418475009	Drug-induced sleep disorder
648	QN_AMI_DRUG_ETOH_DEPEND	425528008	Sexual dysfunction due to substance

CONDITIONID	CONDITION_DESCRIPTION	SNOMED_CODE	DESCRIPTION
648	QN_AMI_DRUG_ETOH_DEPEND	429672007	Drug-induced mood disorder
648	QN_AMI_DRUG_ETOH_DEPEND	441527004	Stimulant abuse
648	QN_AMI_DRUG_ETOH_DEPEND	442406005	Stimulant dependence
648	QN_AMI_DRUG_ETOH_DEPEND	1461000119109	Drug dependence in remission
649	QN_AMI_DRUG_ETOH_WO_DEPEND	5602001	Opioid abuse
649	QN_AMI_DRUG_ETOH_WO_DEPEND	11061003	Psychoactive substance use disorder
649	QN_AMI_DRUG_ETOH_WO_DEPEND	11387009	Psychoactive substance-induced organic mental disorder
649	QN_AMI_DRUG_ETOH_WO_DEPEND	15167005	Alcohol abuse
649	QN_AMI_DRUG_ETOH_WO_DEPEND	37344009	Cannabis abuse
649	QN_AMI_DRUG_ETOH_WO_DEPEND	46975003	Cocaine-induced organic mental disorder
649	QN_AMI_DRUG_ETOH_WO_DEPEND	53050002	Hallucinogen-induced organic mental disorder
649	QN_AMI_DRUG_ETOH_WO_DEPEND	56294008	Nicotine dependence
649	QN_AMI_DRUG_ETOH_WO_DEPEND	56294008	Nicotine dependence
649	QN_AMI_DRUG_ETOH_WO_DEPEND	61104008	Inhalant-induced organic mental disorder
649	QN_AMI_DRUG_ETOH_WO_DEPEND	64386003	Sedative abuse
649	QN_AMI_DRUG_ETOH_WO_DEPEND	66214007	Substance abuse
649	QN_AMI_DRUG_ETOH_WO_DEPEND	70340006	Inhalant abuse
649	QN_AMI_DRUG_ETOH_WO_DEPEND	74851005	Hallucinogen abuse
649	QN_AMI_DRUG_ETOH_WO_DEPEND	77355000	Cannabis-induced organic mental disorder
649	QN_AMI_DRUG_ETOH_WO_DEPEND	78267003	Cocaine abuse
649	QN_AMI_DRUG_ETOH_WO_DEPEND	81703003	Chews tobacco
649	QN_AMI_DRUG_ETOH_WO_DEPEND	84758004	Amphetamine abuse

© 2021 Matheny ME et al. *JAMA Network Open.*

CONDITIONID	CONDITION_DESCRIPTION	SNOMED_CODE	DESCRIPTION
649	QN_AMI_DRUG_ETOH_WO_DEPEND	89765005	Tobacco dependence syndrome
649	QN_AMI_DRUG_ETOH_WO_DEPEND	90755006	Nicotine withdrawal
649	QN_AMI_DRUG_ETOH_WO_DEPEND	91388009	Psychoactive substance abuse
649	QN_AMI_DRUG_ETOH_WO_DEPEND	191882002	Nondependent alcohol abuse, continuous
649	QN_AMI_DRUG_ETOH_WO_DEPEND	191883007	Nondependent alcohol abuse, episodic
649	QN_AMI_DRUG_ETOH_WO_DEPEND	191884001	Nondependent alcohol abuse in remission
649	QN_AMI_DRUG_ETOH_WO_DEPEND	191889006	Tobacco dependence in remission
649	QN_AMI_DRUG_ETOH_WO_DEPEND	191893000	Nondependent cannabis abuse, continuous
649	QN_AMI_DRUG_ETOH_WO_DEPEND	191894006	Nondependent cannabis abuse, episodic
649	QN_AMI_DRUG_ETOH_WO_DEPEND	191895007	Nondependent cannabis abuse in remission
649	QN_AMI_DRUG_ETOH_WO_DEPEND	191899001	Nondependent hallucinogen abuse, continuous
649	QN_AMI_DRUG_ETOH_WO_DEPEND	191900006	Nondependent hallucinogen abuse, episodic
649	QN_AMI_DRUG_ETOH_WO_DEPEND	191901005	Nondependent hallucinogen abuse in remission
649	QN_AMI_DRUG_ETOH_WO_DEPEND	191905001	Nondependent hypnotic or anxiolytic abuse, continuous

© 2021 Matheny ME et al. *JAMA Network Open.*

CONDITIONID	CONDITION_DESCRIPTION	SNOMED_CODE	DESCRIPTION
649	QN_AMI_DRUG_ETOH_WO_DEPEND	191906000	Nondependent hypnotic or anxiolytic abuse, episodic
649	QN_AMI_DRUG_ETOH_WO_DEPEND	191907009	Nondependent hypnotic or anxiolytic abuse in remission
649	QN_AMI_DRUG_ETOH_WO_DEPEND	191912005	Nondependent opioid abuse, continuous
649	QN_AMI_DRUG_ETOH_WO_DEPEND	191913000	Nondependent opioid abuse, episodic
649	QN_AMI_DRUG_ETOH_WO_DEPEND	191914006	Nondependent opioid abuse in remission
649	QN_AMI_DRUG_ETOH_WO_DEPEND	191918009	Nondependent cocaine abuse, continuous
649	QN_AMI_DRUG_ETOH_WO_DEPEND	191919001	Nondependent cocaine abuse, episodic
649	QN_AMI_DRUG_ETOH_WO_DEPEND	191920007	Nondependent cocaine abuse in remission
649	QN_AMI_DRUG_ETOH_WO_DEPEND	191928000	Abuse of antidepressant drug
649	QN_AMI_DRUG_ETOH_WO_DEPEND	191930003	Nondependent antidepressant type drug abuse, continuous
649	QN_AMI_DRUG_ETOH_WO_DEPEND	191931004	Nondependent antidepressant type drug abuse, episodic
649	QN_AMI_DRUG_ETOH_WO_DEPEND	191932006	Nondependent antidepressant type drug abuse in remission

© 2021 Matheny ME et al. *JAMA Network Open.*

CONDITIONID	CONDITION_DESCRIPTION	SNOMED_CODE	DESCRIPTION
649	QN_AMI_DRUG_ETOH_WO_DEPEND	191934007	Nondependent mixed drug abuse
649	QN_AMI_DRUG_ETOH_WO_DEPEND	191936009	Nondependent mixed drug abuse, continuous
649	QN_AMI_DRUG_ETOH_WO_DEPEND	191937000	Nondependent mixed drug abuse, episodic
649	QN_AMI_DRUG_ETOH_WO_DEPEND	191938005	Nondependent mixed drug abuse in remission
649	QN_AMI_DRUG_ETOH_WO_DEPEND	231458000	Abuse of steroids
649	QN_AMI_DRUG_ETOH_WO_DEPEND	268727002	Abuse of non-dependence-producing substances
649	QN_AMI_DRUG_ETOH_WO_DEPEND	280982009	Abuse of laxatives
649	QN_AMI_DRUG_ETOH_WO_DEPEND	280983004	Abuse of vitamins
649	QN_AMI_DRUG_ETOH_WO_DEPEND	280986007	Abuse of antacids
649	QN_AMI_DRUG_ETOH_WO_DEPEND	312098001	Sedative, hypnotic AND/OR anxiolytic-related disorder
649	QN_AMI_DRUG_ETOH_WO_DEPEND	427205009	Amphetamine abuse, continuous
649	QN_AMI_DRUG_ETOH_WO_DEPEND	429692000	Amphetamine abuse, episodic
649	QN_AMI_DRUG_ETOH_WO_DEPEND	441527004	Stimulant abuse
649	QN_AMI_DRUG_ETOH_WO_DEPEND	288281000119100	Abuse of herbal medicine or folk remedy
650	QN_AMI_SCHIZOPHREN	4926007	Schizophrenia in remission
650	QN_AMI_SCHIZOPHREN	7025000	Subchronic undifferentiated schizophrenia with acute exacerbations
650	QN_AMI_SCHIZOPHREN	12939007	Chronic disorganized schizophrenia

© 2021 Matheny ME et al. *JAMA Network Open.*

CONDITIONID	CONDITION_DESCRIPTION	SNOMED_CODE	DESCRIPTION
650	QN_AMI_SCHIZOPHREN	14291003	Subchronic disorganized schizophrenia with acute exacerbations
650	QN_AMI_SCHIZOPHREN	16990005	Subchronic schizophrenia
650	QN_AMI_SCHIZOPHREN	26025008	Residual schizophrenia
650	QN_AMI_SCHIZOPHREN	27387000	Subchronic disorganized schizophrenia
650	QN_AMI_SCHIZOPHREN	29599000	Chronic undifferentiated schizophrenia
650	QN_AMI_SCHIZOPHREN	30336007	Chronic residual schizophrenia with acute exacerbations
650	QN_AMI_SCHIZOPHREN	31373002	Disorganized schizophrenia in remission
650	QN_AMI_SCHIZOPHREN	31658008	Chronic paranoid schizophrenia
650	QN_AMI_SCHIZOPHREN	35218008	Chronic disorganized schizophrenia with acute exacerbations
650	QN_AMI_SCHIZOPHREN	35252006	Disorganized schizophrenia
650	QN_AMI_SCHIZOPHREN	38368003	Schizoaffective disorder, bipolar type
650	QN_AMI_SCHIZOPHREN	42868002	Subchronic catatonic schizophrenia
650	QN_AMI_SCHIZOPHREN	51133006	Residual schizophrenia in remission
650	QN_AMI_SCHIZOPHREN	58214004	Schizophrenia

© 2021 Matheny ME et al. *JAMA Network Open.*

CONDITIONID	CONDITION_DESCRIPTION	SNOMED_CODE	DESCRIPTION
650	QN_AMI_SCHIZOPHREN	63181006	Paranoid schizophrenia in remission
650	QN_AMI_SCHIZOPHREN	64905009	Paranoid schizophrenia
650	QN_AMI_SCHIZOPHREN	68890003	Schizoaffective disorder
650	QN_AMI_SCHIZOPHREN	68995007	Chronic catatonic schizophrenia
650	QN_AMI_SCHIZOPHREN	70814008	Subchronic residual schizophrenia with acute exacerbations
650	QN_AMI_SCHIZOPHREN	71103003	Chronic residual schizophrenia
650	QN_AMI_SCHIZOPHREN	76566000	Subchronic residual schizophrenia
650	QN_AMI_SCHIZOPHREN	79204003	Chronic undifferentiated schizophrenia with acute exacerbations
650	QN_AMI_SCHIZOPHREN	79866005	Subchronic paranoid schizophrenia
650	QN_AMI_SCHIZOPHREN	84760002	Schizoaffective disorder, depressive type
650	QN_AMI_SCHIZOPHREN	88975006	Schizophreniform disorder
650	QN_AMI_SCHIZOPHREN	111482003	Subchronic schizophrenia with acute exacerbations
650	QN_AMI_SCHIZOPHREN	111483008	Catatonic schizophrenia in remission
650	QN_AMI_SCHIZOPHREN	111484002	Undifferentiated schizophrenia
650	QN_AMI_SCHIZOPHREN	191526005	Schizophrenic disorders

© 2021 Matheny ME et al. *JAMA Network Open.*

CONDITIONID	CONDITION_DESCRIPTION	SNOMED_CODE	DESCRIPTION
650	QN_AMI_SCHIZOPHREN	191527001	Simple schizophrenia
650	QN_AMI_SCHIZOPHREN	191530008	Acute exacerbation of subchronic schizophrenia
650	QN_AMI_SCHIZOPHREN	191531007	Acute exacerbation of chronic schizophrenia
650	QN_AMI_SCHIZOPHREN	191542003	Catatonic schizophrenia
650	QN_AMI_SCHIZOPHREN	191547009	Acute exacerbation of subchronic catatonic schizophrenia
650	QN_AMI_SCHIZOPHREN	191548004	Acute exacerbation of chronic catatonic schizophrenia
650	QN_AMI_SCHIZOPHREN	191554003	Acute exacerbation of subchronic paranoid schizophrenia
650	QN_AMI_SCHIZOPHREN	191555002	Acute exacerbation of chronic paranoid schizophrenia
650	QN_AMI_SCHIZOPHREN	191559008	Latent schizophrenia
650	QN_AMI_SCHIZOPHREN	191561004	Subchronic latent schizophrenia
650	QN_AMI_SCHIZOPHREN	191562006	Chronic latent schizophrenia
650	QN_AMI_SCHIZOPHREN	191563001	Acute exacerbation of subchronic latent schizophrenia
650	QN_AMI_SCHIZOPHREN	191564007	Acute exacerbation of chronic latent schizophrenia
650	QN_AMI_SCHIZOPHREN	191565008	Latent schizophrenia in remission

© 2021 Matheny ME et al. *JAMA Network Open.*

CONDITIONID	CONDITION_DESCRIPTION	SNOMED_CODE	DESCRIPTION
650	QN_AMI_SCHIZOPHREN	191569002	Subchronic schizoaffective schizophrenia
650	QN_AMI_SCHIZOPHREN	191570001	Chronic schizoaffective schizophrenia
650	QN_AMI_SCHIZOPHREN	191571002	Acute exacerbation of subchronic schizoaffective schizophrenia
650	QN_AMI_SCHIZOPHREN	191572009	Acute exacerbation of chronic schizoaffective schizophrenia
650	QN_AMI_SCHIZOPHREN	191574005	Schizoaffective schizophrenia in remission
651	QN_AMI_MDD_BIPOLAR_PARANOID	162004	Severe manic bipolar I disorder without psychotic features
651	QN_AMI_MDD_BIPOLAR_PARANOID	666000	Poisoning by antivaricose drug AND/OR sclerosing agent
651	QN_AMI_MDD_BIPOLAR_PARANOID	1848004	Poisoning by gaseous anesthetic
651	QN_AMI_MDD_BIPOLAR_PARANOID	2094002	Toxic effect of carbon disulfide
651	QN_AMI_MDD_BIPOLAR_PARANOID	2935001	Poisoning by antiasthmatic
651	QN_AMI_MDD_BIPOLAR_PARANOID	3530005	Bipolar I disorder, single manic episode, in full remission
651	QN_AMI_MDD_BIPOLAR_PARANOID	4771009	Poisoning by cephalosporin group antibiotic
651	QN_AMI_MDD_BIPOLAR_PARANOID	5576007	Toxic effect of nitric acid

© 2021 Matheny ME et al. *JAMA Network Open.*

CONDITIONID	CONDITION_DESCRIPTION	SNOMED_CODE	DESCRIPTION
651	QN_AMI_MDD_BIPOLAR_PARANOID	5578008	Poisoning by fibrinolysis-affecting drug
651	QN_AMI_MDD_BIPOLAR_PARANOID	5703000	Bipolar disorder in partial remission
651	QN_AMI_MDD_BIPOLAR_PARANOID	7201003	Poisoning by anthelmintic
651	QN_AMI_MDD_BIPOLAR_PARANOID	7248001	Poisoning by salicylate
651	QN_AMI_MDD_BIPOLAR_PARANOID	7895008	Poisoning by drug AND/OR medicinal substance
651	QN_AMI_MDD_BIPOLAR_PARANOID	7998009	Poisoning by bee sting
651	QN_AMI_MDD_BIPOLAR_PARANOID	9291000	Poisoning by monoamine oxidase inhibitor
651	QN_AMI_MDD_BIPOLAR_PARANOID	9340000	Bipolar I disorder, single manic episode
651	QN_AMI_MDD_BIPOLAR_PARANOID	9408000	Poisoning by thyroid hormone AND/OR thyroid derivative
651	QN_AMI_MDD_BIPOLAR_PARANOID	9982009	Poisoning by cocaine
651	QN_AMI_MDD_BIPOLAR_PARANOID	10235002	Tetrachloroethylene poisoning
651	QN_AMI_MDD_BIPOLAR_PARANOID	10981006	Severe mixed bipolar I disorder with psychotic features
651	QN_AMI_MDD_BIPOLAR_PARANOID	10984003	Poisoning by skeletal muscle relaxant
651	QN_AMI_MDD_BIPOLAR_PARANOID	11196001	Poisoning by opiate AND/OR related narcotic
651	QN_AMI_MDD_BIPOLAR_PARANOID	13187008	Poisoning by heroin
651	QN_AMI_MDD_BIPOLAR_PARANOID	13746004	Bipolar disorder

© 2021 Matheny ME et al. *JAMA Network Open.*

CONDITIONID	CONDITION_DESCRIPTION	SNOMED_CODE	DESCRIPTION
651	QN_AMI_MDD_BIPOLAR_PARANOID	14546008	Late effect of poisoning due to drug, medicinal AND/OR biological substance
651	QN_AMI_MDD_BIPOLAR_PARANOID	14817008	Late effect of injury
651	QN_AMI_MDD_BIPOLAR_PARANOID	15056007	Poisoning due to arthropod venom
651	QN_AMI_MDD_BIPOLAR_PARANOID	15233006	Poisoning by cannabis derivative
651	QN_AMI_MDD_BIPOLAR_PARANOID	15639000	Moderate major depression, single episode
651	QN_AMI_MDD_BIPOLAR_PARANOID	16464001	Toxic effect of carbon tetrachloride
651	QN_AMI_MDD_BIPOLAR_PARANOID	16506000	Mixed bipolar I disorder
651	QN_AMI_MDD_BIPOLAR_PARANOID	16686005	Toxic effect of hydrocyanic acid
651	QN_AMI_MDD_BIPOLAR_PARANOID	18818009	Moderate recurrent major depression
651	QN_AMI_MDD_BIPOLAR_PARANOID	20260003	Poisoning by psychostimulant
651	QN_AMI_MDD_BIPOLAR_PARANOID	21413004	Poisoning by coronary vasodilator
651	QN_AMI_MDD_BIPOLAR_PARANOID	21520009	Toxic effect of soap AND/OR detergent
651	QN_AMI_MDD_BIPOLAR_PARANOID	22121000	Depressed bipolar I disorder in full remission
651	QN_AMI_MDD_BIPOLAR_PARANOID	22721008	Toxic effect of aflatoxin
651	QN_AMI_MDD_BIPOLAR_PARANOID	22915003	Poisoning by cardiovascular system drug
651	QN_AMI_MDD_BIPOLAR_PARANOID	23833000	Poisoning by antineoplastic AND/OR

© 2021 Matheny ME et al. *JAMA Network Open.*

CONDITIONID	CONDITION_DESCRIPTION	SNOMED_CODE	DESCRIPTION
			immunosuppressive drug
651	QN_AMI_MDD_BIPOLAR_PARANOID	24000001	Poisoning by antiviral drug
651	QN_AMI_MDD_BIPOLAR_PARANOID	24310001	Toxic effect of sulfur dioxide
651	QN_AMI_MDD_BIPOLAR_PARANOID	24984009	Poisoning by antimycobacterial drug
651	QN_AMI_MDD_BIPOLAR_PARANOID	25555003	Chloroform poisoning
651	QN_AMI_MDD_BIPOLAR_PARANOID	26033009	Toxic effect of mycotoxin
651	QN_AMI_MDD_BIPOLAR_PARANOID	26472000	Paraphrenia
651	QN_AMI_MDD_BIPOLAR_PARANOID	27064003	Poisoning by emetic
651	QN_AMI_MDD_BIPOLAR_PARANOID	28008001	Toxic effect of copper salt
651	QN_AMI_MDD_BIPOLAR_PARANOID	28884001	Moderate bipolar I disorder, single manic episode
651	QN_AMI_MDD_BIPOLAR_PARANOID	29290000	Poisoning by sulfonamide
651	QN_AMI_MDD_BIPOLAR_PARANOID	30935000	Manic bipolar I disorder in full remission
651	QN_AMI_MDD_BIPOLAR_PARANOID	31446002	Bipolar I disorder, most recent episode hypomanic
651	QN_AMI_MDD_BIPOLAR_PARANOID	32175001	Suffocation by plastic bag
651	QN_AMI_MDD_BIPOLAR_PARANOID	32449007	Poisoning by bacterial vaccine
651	QN_AMI_MDD_BIPOLAR_PARANOID	32664004	Toxic effect of manganese AND/OR its compounds
651	QN_AMI_MDD_BIPOLAR_PARANOID	32835006	Poisoning by autonomous nervous system drug

© 2021 Matheny ME et al. *JAMA Network Open.*

CONDITIONID	CONDITION_DESCRIPTION	SNOMED_CODE	DESCRIPTION
651	QN_AMI_MDD_BIPOLAR_PARANOID	33135002	Recurrent major depression in partial remission
651	QN_AMI_MDD_BIPOLAR_PARANOID	35149008	Suffocation by bedclothes
651	QN_AMI_MDD_BIPOLAR_PARANOID	35279005	Poisoning by antacid AND/OR antigastric secretion drug
651	QN_AMI_MDD_BIPOLAR_PARANOID	36465006	Toxic effect from eating berries AND/OR other plants
651	QN_AMI_MDD_BIPOLAR_PARANOID	36474008	Severe recurrent major depression without psychotic features
651	QN_AMI_MDD_BIPOLAR_PARANOID	36583000	Mixed bipolar I disorder in partial remission
651	QN_AMI_MDD_BIPOLAR_PARANOID	36923009	Major depression, single episode
651	QN_AMI_MDD_BIPOLAR_PARANOID	38342005	Toxic effect of lead compound
651	QN_AMI_MDD_BIPOLAR_PARANOID	38751004	Poisoning by analgesic AND/OR antipyretic
651	QN_AMI_MDD_BIPOLAR_PARANOID	38918003	Poisoning by caterpillar
651	QN_AMI_MDD_BIPOLAR_PARANOID	39451005	Toxic effect of acid
651	QN_AMI_MDD_BIPOLAR_PARANOID	39994006	Poisoning by antifungal antibiotic
651	QN_AMI_MDD_BIPOLAR_PARANOID	40119006	Poisoning due to brown recluse spider venom
651	QN_AMI_MDD_BIPOLAR_PARANOID	40379007	Mild recurrent major depression
651	QN_AMI_MDD_BIPOLAR_PARANOID	40748005	Poisoning by intravenous anesthetic

© 2021 Matheny ME et al. *JAMA Network Open.*

CONDITIONID	CONDITION_DESCRIPTION	SNOMED_CODE	DESCRIPTION
651	QN_AMI_MDD_BIPOLAR_PARANOID	40926005	Moderate mixed bipolar I disorder
651	QN_AMI_MDD_BIPOLAR_PARANOID	41234002	Poisoning by anesthetic AND/OR muscle-tone depressant
651	QN_AMI_MDD_BIPOLAR_PARANOID	41552001	Mild bipolar I disorder, single manic episode
651	QN_AMI_MDD_BIPOLAR_PARANOID	41832009	Severe bipolar I disorder, single manic episode with psychotic features
651	QN_AMI_MDD_BIPOLAR_PARANOID	41836007	Bipolar disorder in full remission
651	QN_AMI_MDD_BIPOLAR_PARANOID	42377002	Poisoning by anticonvulsant
651	QN_AMI_MDD_BIPOLAR_PARANOID	43124005	Poisoning by skin AND/OR mucous membrane drug
651	QN_AMI_MDD_BIPOLAR_PARANOID	43624006	Poisoning by gastrointestinal system drug
651	QN_AMI_MDD_BIPOLAR_PARANOID	44003006	Poisoning by barbiturate
651	QN_AMI_MDD_BIPOLAR_PARANOID	44400004	Toxic effect of venom
651	QN_AMI_MDD_BIPOLAR_PARANOID	44961008	Poisoning by antidiarrheal drug
651	QN_AMI_MDD_BIPOLAR_PARANOID	45519004	Toxic effect of strychnine
651	QN_AMI_MDD_BIPOLAR_PARANOID	45671002	Toxic effect of cadmium AND/OR its compounds
651	QN_AMI_MDD_BIPOLAR_PARANOID	46193001	Poisoning by androgen AND/OR anabolic congener
651	QN_AMI_MDD_BIPOLAR_PARANOID	46206005	Mood disorder
651	QN_AMI_MDD_BIPOLAR_PARANOID	46229002	Severe mixed bipolar I disorder

© 2021 Matheny ME et al. *JAMA Network Open.*

CONDITIONID	CONDITION_DESCRIPTION	SNOMED_CODE	DESCRIPTION
			without psychotic features
651	QN_AMI_MDD_BIPOLAR_PARANOID	46262005	Poisoning by phenol
651	QN_AMI_MDD_BIPOLAR_PARANOID	46298002	Poisoning by hornet sting
651	QN_AMI_MDD_BIPOLAR_PARANOID	46334001	Toxic effect of nitrogen oxide
651	QN_AMI_MDD_BIPOLAR_PARANOID	48500005	Delusional disorder
651	QN_AMI_MDD_BIPOLAR_PARANOID	49450009	Poisoning by antiprotozoal drug
651	QN_AMI_MDD_BIPOLAR_PARANOID	49468007	Depressed bipolar I disorder
651	QN_AMI_MDD_BIPOLAR_PARANOID	49512000	Depressed bipolar I disorder in partial remission
651	QN_AMI_MDD_BIPOLAR_PARANOID	50578005	Toxic effect of beryllium AND/OR its compounds
651	QN_AMI_MDD_BIPOLAR_PARANOID	51040009	Thallium poisoning
651	QN_AMI_MDD_BIPOLAR_PARANOID	51228004	Poisoning by anterior pituitary hormone
651	QN_AMI_MDD_BIPOLAR_PARANOID	55381001	Poisoning by iron AND/OR its compounds
651	QN_AMI_MDD_BIPOLAR_PARANOID	55847002	Rodenticide poisoning
651	QN_AMI_MDD_BIPOLAR_PARANOID	57005003	Poisoning by caffeine
651	QN_AMI_MDD_BIPOLAR_PARANOID	57335002	Toxic effect of gas, fumes AND/OR vapors
651	QN_AMI_MDD_BIPOLAR_PARANOID	57346004	Toxic effect of fusel oil
651	QN_AMI_MDD_BIPOLAR_PARANOID	57431005	Poisoning due to reptile venom
651	QN_AMI_MDD_BIPOLAR_PARANOID	58104004	Poisoning by expectorant
651	QN_AMI_MDD_BIPOLAR_PARANOID	59617007	Severe depressed bipolar I disorder

© 2021 Matheny ME et al. *JAMA Network Open.*

CONDITIONID	CONDITION_DESCRIPTION	SNOMED_CODE	DESCRIPTION
			with psychotic features
651	QN_AMI_MDD_BIPOLAR_PARANOID	60199004	Poisoning by methadone
651	QN_AMI_MDD_BIPOLAR_PARANOID	61288004	Poisoning by venomous snake
651	QN_AMI_MDD_BIPOLAR_PARANOID	61356009	Poisoning by parasympathomimetic drug
651	QN_AMI_MDD_BIPOLAR_PARANOID	61403008	Severe depressed bipolar I disorder without psychotic features
651	QN_AMI_MDD_BIPOLAR_PARANOID	61438005	Poisoning by psychotropic agent
651	QN_AMI_MDD_BIPOLAR_PARANOID	61831009	Induced psychotic disorder
651	QN_AMI_MDD_BIPOLAR_PARANOID	62208003	Jellyfish poisoning
651	QN_AMI_MDD_BIPOLAR_PARANOID	62276003	Poisoning by succinimide
651	QN_AMI_MDD_BIPOLAR_PARANOID	63249007	Manic bipolar I disorder in partial remission
651	QN_AMI_MDD_BIPOLAR_PARANOID	63978002	Late effect of toxic effects of nonmedical substances
651	QN_AMI_MDD_BIPOLAR_PARANOID	65435003	Poisoning by antiadrenergic drug
651	QN_AMI_MDD_BIPOLAR_PARANOID	66207005	Toxic effect of cyanide
651	QN_AMI_MDD_BIPOLAR_PARANOID	66344007	Recurrent major depression
651	QN_AMI_MDD_BIPOLAR_PARANOID	66409006	Poisoning by oxytocic agent
651	QN_AMI_MDD_BIPOLAR_PARANOID	66413004	Poisoning by penicillin
651	QN_AMI_MDD_BIPOLAR_PARANOID	66466001	Asphyxiation
651	QN_AMI_MDD_BIPOLAR_PARANOID	67002003	Severe bipolar II disorder, most

© 2021 Matheny ME et al. *JAMA Network Open.*

CONDITIONID	CONDITION_DESCRIPTION	SNOMED_CODE	DESCRIPTION
			recent episode major depressive, in partial remission
651	QN_AMI_MDD_BIPOLAR_PARANOID	67181005	Toxic effect of metal
651	QN_AMI_MDD_BIPOLAR_PARANOID	67426006	Toxic effect of alcohol
651	QN_AMI_MDD_BIPOLAR_PARANOID	67438002	Poisoning by anticoagulant
651	QN_AMI_MDD_BIPOLAR_PARANOID	67794001	Poisoning by drug acting on respiratory system
651	QN_AMI_MDD_BIPOLAR_PARANOID	67893003	Poisoning by lysergide
651	QN_AMI_MDD_BIPOLAR_PARANOID	68019004	Recurrent major depression in remission
651	QN_AMI_MDD_BIPOLAR_PARANOID	68563002	Poisoning by carbonic acid anhydrase inhibitor
651	QN_AMI_MDD_BIPOLAR_PARANOID	69434005	Tricyclic antidepressant poisoning
651	QN_AMI_MDD_BIPOLAR_PARANOID	70539005	Poisoning by hormone AND/OR synthetic substitute
651	QN_AMI_MDD_BIPOLAR_PARANOID	70747007	Major depression single episode, in partial remission
651	QN_AMI_MDD_BIPOLAR_PARANOID	71841008	Toxic effect of caustic substance
651	QN_AMI_MDD_BIPOLAR_PARANOID	74264003	Poisoning by opium alkaloid
651	QN_AMI_MDD_BIPOLAR_PARANOID	76441001	Severe major depression, single episode, without psychotic features
651	QN_AMI_MDD_BIPOLAR_PARANOID	79298009	Mild major depression, single episode

© 2021 Matheny ME et al. *JAMA Network Open.*

CONDITIONID	CONDITION_DESCRIPTION	SNOMED_CODE	DESCRIPTION
651	QN_AMI_MDD_BIPOLAR_PARANOID	81034007	Algae poisoning
651	QN_AMI_MDD_BIPOLAR_PARANOID	81844008	Toxic effect of arsenic AND/OR its compounds
651	QN_AMI_MDD_BIPOLAR_PARANOID	81914009	Poisoning by benzodiazepine-based tranquilizer
651	QN_AMI_MDD_BIPOLAR_PARANOID	82276009	Poisoning by antidepressant
651	QN_AMI_MDD_BIPOLAR_PARANOID	82313006	Suicide attempt
651	QN_AMI_MDD_BIPOLAR_PARANOID	82782008	Toxic effect of ethyl alcohol
651	QN_AMI_MDD_BIPOLAR_PARANOID	83225003	Bipolar II disorder
651	QN_AMI_MDD_BIPOLAR_PARANOID	83227006	Scombroid fish poisoning
651	QN_AMI_MDD_BIPOLAR_PARANOID	84419005	Toxic effect of lacrimogenic gas
651	QN_AMI_MDD_BIPOLAR_PARANOID	84997000	Insecticide poisoning
651	QN_AMI_MDD_BIPOLAR_PARANOID	85180002	Toxic effect of mercury AND/OR its compounds
651	QN_AMI_MDD_BIPOLAR_PARANOID	85248005	Bipolar disorder in remission
651	QN_AMI_MDD_BIPOLAR_PARANOID	85337000	Poisoning by sedative AND/OR hypnotic
651	QN_AMI_MDD_BIPOLAR_PARANOID	85975005	Poisoning by psychodysleptic
651	QN_AMI_MDD_BIPOLAR_PARANOID	86068002	Toxic effect of noxious substance eaten as food
651	QN_AMI_MDD_BIPOLAR_PARANOID	86505009	Toxic effect from eating mushrooms
651	QN_AMI_MDD_BIPOLAR_PARANOID	86733006	Poisoning by antitussive
651	QN_AMI_MDD_BIPOLAR_PARANOID	86772005	Toxic effect of petroleum product
651	QN_AMI_MDD_BIPOLAR_PARANOID	87904007	Poisoning by drug acting on smooth

© 2021 Matheny ME et al. *JAMA Network Open.*

CONDITIONID	CONDITION_DESCRIPTION	SNOMED_CODE	DESCRIPTION
			AND/OR skeletal muscle
651	QN_AMI_MDD_BIPOLAR_PARANOID	88627003	Toxic effect of chromium
651	QN_AMI_MDD_BIPOLAR_PARANOID	89878004	Poisoning by halogenated hydrocarbon derivative
651	QN_AMI_MDD_BIPOLAR_PARANOID	90210000	Poisoning by central appetite depressant
651	QN_AMI_MDD_BIPOLAR_PARANOID	90765000	Toxic effect of chlorine gas
651	QN_AMI_MDD_BIPOLAR_PARANOID	95872005	Carbon monoxide poisoning from motor vehicle exhaust
651	QN_AMI_MDD_BIPOLAR_PARANOID	106131003	Mood finding
651	QN_AMI_MDD_BIPOLAR_PARANOID	111485001	Mixed bipolar I disorder in full remission
651	QN_AMI_MDD_BIPOLAR_PARANOID	111757004	Poisoning by aromatic analgesic
651	QN_AMI_MDD_BIPOLAR_PARANOID	111758009	Poisoning by enzyme
651	QN_AMI_MDD_BIPOLAR_PARANOID	111760006	Poisoning by vitamin
651	QN_AMI_MDD_BIPOLAR_PARANOID	111762003	Poisoning by antirheumatic
651	QN_AMI_MDD_BIPOLAR_PARANOID	111764002	Local anesthetic toxicity
651	QN_AMI_MDD_BIPOLAR_PARANOID	111767009	Poisoning by antilipemic AND/OR antiarteriosclerotic drug
651	QN_AMI_MDD_BIPOLAR_PARANOID	111768004	Poisoning by vasodilator
651	QN_AMI_MDD_BIPOLAR_PARANOID	111769007	Poisoning by antihypertensive agent

© 2021 Matheny ME et al. *JAMA Network Open.*

CONDITIONID	CONDITION_DESCRIPTION	SNOMED_CODE	DESCRIPTION
651	QN_AMI_MDD_BIPOLAR_PARANOID	123012009	Poisoning by antidote AND/OR chelating agent
651	QN_AMI_MDD_BIPOLAR_PARANOID	129674006	Asphyxiation by hanging
651	QN_AMI_MDD_BIPOLAR_PARANOID	191583000	Single manic episode, mild
651	QN_AMI_MDD_BIPOLAR_PARANOID	191584006	Single manic episode, moderate
651	QN_AMI_MDD_BIPOLAR_PARANOID	191586008	Single manic episode, severe, with psychosis
651	QN_AMI_MDD_BIPOLAR_PARANOID	191588009	Single manic episode in full remission
651	QN_AMI_MDD_BIPOLAR_PARANOID	191590005	Recurrent manic episodes
651	QN_AMI_MDD_BIPOLAR_PARANOID	191592002	Recurrent manic episodes, mild
651	QN_AMI_MDD_BIPOLAR_PARANOID	191593007	Recurrent manic episodes, moderate
651	QN_AMI_MDD_BIPOLAR_PARANOID	191595000	Recurrent manic episodes, severe, with psychosis
651	QN_AMI_MDD_BIPOLAR_PARANOID	191597008	Recurrent manic episodes, in full remission
651	QN_AMI_MDD_BIPOLAR_PARANOID	191601008	Single major depressive episode, mild
651	QN_AMI_MDD_BIPOLAR_PARANOID	191602001	Single major depressive episode, moderate
651	QN_AMI_MDD_BIPOLAR_PARANOID	191606003	Single major depressive episode, in full remission
651	QN_AMI_MDD_BIPOLAR_PARANOID	191610000	Recurrent major depressive episodes, mild

© 2021 Matheny ME et al. *JAMA Network Open.*

CONDITIONID	CONDITION_DESCRIPTION	SNOMED_CODE	DESCRIPTION
651	QN_AMI_MDD_BIPOLAR_PARANOID	191611001	Recurrent major depressive episodes, moderate
651	QN_AMI_MDD_BIPOLAR_PARANOID	191613003	Recurrent major depressive episodes, severe, with psychosis
651	QN_AMI_MDD_BIPOLAR_PARANOID	191615005	Recurrent major depressive episodes, in full remission
651	QN_AMI_MDD_BIPOLAR_PARANOID	191616006	Recurrent depression
651	QN_AMI_MDD_BIPOLAR_PARANOID	191618007	Bipolar affective disorder, current episode manic
651	QN_AMI_MDD_BIPOLAR_PARANOID	191620005	Bipolar affective disorder, currently manic, mild
651	QN_AMI_MDD_BIPOLAR_PARANOID	191621009	Bipolar affective disorder, currently manic, moderate
651	QN_AMI_MDD_BIPOLAR_PARANOID	191623007	Bipolar affective disorder, currently manic, severe, with psychosis
651	QN_AMI_MDD_BIPOLAR_PARANOID	191625000	Bipolar affective disorder, currently manic, in full remission
651	QN_AMI_MDD_BIPOLAR_PARANOID	191629006	Bipolar affective disorder, currently depressed, mild
651	QN_AMI_MDD_BIPOLAR_PARANOID	191630001	Bipolar affective disorder, currently depressed, moderate
651	QN_AMI_MDD_BIPOLAR_PARANOID	191634005	Bipolar affective disorder, currently

© 2021 Matheny ME et al. *JAMA Network Open.*

CONDITIONID	CONDITION_DESCRIPTION	SNOMED_CODE	DESCRIPTION
			depressed, in full remission
651	QN_AMI_MDD_BIPOLAR_PARANOID	191638008	Mixed bipolar affective disorder, mild
651	QN_AMI_MDD_BIPOLAR_PARANOID	191639000	Mixed bipolar affective disorder, moderate
651	QN_AMI_MDD_BIPOLAR_PARANOID	191641004	Mixed bipolar affective disorder, severe, with psychosis
651	QN_AMI_MDD_BIPOLAR_PARANOID	191643001	Mixed bipolar affective disorder, in full remission
651	QN_AMI_MDD_BIPOLAR_PARANOID	191658009	Atypical manic disorder
651	QN_AMI_MDD_BIPOLAR_PARANOID	191659001	Atypical depressive disorder
651	QN_AMI_MDD_BIPOLAR_PARANOID	192362008	Bipolar affective disorder, current episode mixed
651	QN_AMI_MDD_BIPOLAR_PARANOID	212509008	Poisoning by rifampin
651	QN_AMI_MDD_BIPOLAR_PARANOID	212545006	Poisoning by antidiabetic agent
651	QN_AMI_MDD_BIPOLAR_PARANOID	212553003	Poisoning by mineralocorticoid or mineralocorticoid antagonist
651	QN_AMI_MDD_BIPOLAR_PARANOID	212599001	Pyrazole derivative poisoning
651	QN_AMI_MDD_BIPOLAR_PARANOID	212619008	Hydantoin derivative poisoning
651	QN_AMI_MDD_BIPOLAR_PARANOID	212625007	Poisoning by iminostilbenes
651	QN_AMI_MDD_BIPOLAR_PARANOID	212641008	Poisoning by central nervous

© 2021 Matheny ME et al. *JAMA Network Open.*

CONDITIONID	CONDITION_DESCRIPTION	SNOMED_CODE	DESCRIPTION
			system muscle tone depressant
651	QN_AMI_MDD_BIPOLAR_PARANOID	212694004	Ganglion-blocker poisoning
651	QN_AMI_MDD_BIPOLAR_PARANOID	212718008	Poisoning by histamine H2-receptor antagonists
651	QN_AMI_MDD_BIPOLAR_PARANOID	212723008	Poisoning by saline and osmotic laxatives
651	QN_AMI_MDD_BIPOLAR_PARANOID	212725001	Digestant poisoning
651	QN_AMI_MDD_BIPOLAR_PARANOID	212778002	Topical dental drug poisoning
651	QN_AMI_MDD_BIPOLAR_PARANOID	212809004	Methyl alcohol causing toxic effect
651	QN_AMI_MDD_BIPOLAR_PARANOID	212813006	Toxic effect of isopropyl alcohol
651	QN_AMI_MDD_BIPOLAR_PARANOID	212829003	Toxic effect of dichloromethane
651	QN_AMI_MDD_BIPOLAR_PARANOID	212835003	Toxic effect of caustic alkali
651	QN_AMI_MDD_BIPOLAR_PARANOID	212846000	Toxic effect of tin and its compounds
651	QN_AMI_MDD_BIPOLAR_PARANOID	212847009	Toxic effect of phosphorus and its compounds
651	QN_AMI_MDD_BIPOLAR_PARANOID	212857005	Toxic effect of fluorine gas and hydrogen fluoride
651	QN_AMI_MDD_BIPOLAR_PARANOID	212858000	Toxic effect of hydrogen sulfide
651	QN_AMI_MDD_BIPOLAR_PARANOID	212859008	Toxic effect of carbon dioxide
651	QN_AMI_MDD_BIPOLAR_PARANOID	212877003	Organophosphate and carbamate causing toxic effect
651	QN_AMI_MDD_BIPOLAR_PARANOID	212889001	Toxic effect of herbicides and fungicides

© 2021 Matheny ME et al. *JAMA Network Open.*

CONDITIONID	CONDITION_DESCRIPTION	SNOMED_CODE	DESCRIPTION
651	QN_AMI_MDD_BIPOLAR_PARANOID	212892002	Toxic effect of contact with fish
651	QN_AMI_MDD_BIPOLAR_PARANOID	212894001	Toxic effect of nitroderivatives and aminoderivatives of benzene and its homologues
651	QN_AMI_MDD_BIPOLAR_PARANOID	212898003	Toxic effect of formaldehyde
651	QN_AMI_MDD_BIPOLAR_PARANOID	212899006	Toxic effect of tobacco and nicotine
651	QN_AMI_MDD_BIPOLAR_PARANOID	212979001	Suffocation by mechanical cause
651	QN_AMI_MDD_BIPOLAR_PARANOID	216609004	Accidental poisoning by antibiotic
651	QN_AMI_MDD_BIPOLAR_PARANOID	216612001	Accidental poisoning by hormones and synthetic substitutes
651	QN_AMI_MDD_BIPOLAR_PARANOID	217653004	Poisoning due to coral snake venom
651	QN_AMI_MDD_BIPOLAR_PARANOID	217655006	Poisoning due to gila monster venom
651	QN_AMI_MDD_BIPOLAR_PARANOID	217659000	Poisoning due to rattlesnake venom
651	QN_AMI_MDD_BIPOLAR_PARANOID	217665000	Poisoning due to venomous spider
651	QN_AMI_MDD_BIPOLAR_PARANOID	217666004	Poisoning due to black widow spider venom
651	QN_AMI_MDD_BIPOLAR_PARANOID	217668003	Poisoning due to tarantula spider venom
651	QN_AMI_MDD_BIPOLAR_PARANOID	217670007	Poisoning due to scorpion venom

© 2021 Matheny ME et al. *JAMA Network Open.*

CONDITIONID	CONDITION_DESCRIPTION	SNOMED_CODE	DESCRIPTION
651	QN_AMI_MDD_BIPOLAR_PARANOID	217673009	Toxic reaction to wasp sting
651	QN_AMI_MDD_BIPOLAR_PARANOID	217676001	Poisoning due to centipede and venomous millipede
651	QN_AMI_MDD_BIPOLAR_PARANOID	217681005	Poisoning due to sting of ant
651	QN_AMI_MDD_BIPOLAR_PARANOID	217684002	Poisoning due to venomous marine animals or plants
651	QN_AMI_MDD_BIPOLAR_PARANOID	219122005	Self poisoning by corrosive or caustic substance
651	QN_AMI_MDD_BIPOLAR_PARANOID	219123000	Self poisoning by arsenic or its compounds
651	QN_AMI_MDD_BIPOLAR_PARANOID	219174008	Late effect of self inflicted injury
651	QN_AMI_MDD_BIPOLAR_PARANOID	224946001	Self-electrocution
651	QN_AMI_MDD_BIPOLAR_PARANOID	241748001	Poisoning by analgesic drug
651	QN_AMI_MDD_BIPOLAR_PARANOID	241774007	Ciguatoxin causing toxic effect
651	QN_AMI_MDD_BIPOLAR_PARANOID	241783002	Agrochemical or pesticide causing toxic effect
651	QN_AMI_MDD_BIPOLAR_PARANOID	241801006	Zinc sulfide causing toxic effect
651	QN_AMI_MDD_BIPOLAR_PARANOID	241832007	Poisoning due to Portuguese Man-of-war sting
651	QN_AMI_MDD_BIPOLAR_PARANOID	241835009	Poisoning by sea anemone
651	QN_AMI_MDD_BIPOLAR_PARANOID	241869005	Paint causing toxic effect
651	QN_AMI_MDD_BIPOLAR_PARANOID	242840004	Self poisoning by carbon monoxide
651	QN_AMI_MDD_BIPOLAR_PARANOID	248061004	Self-harm
651	QN_AMI_MDD_BIPOLAR_PARANOID	268619003	Manic disorder, single episode

© 2021 Matheny ME et al. *JAMA Network Open.*

CONDITIONID	CONDITION_DESCRIPTION	SNOMED_CODE	DESCRIPTION
651	QN_AMI_MDD_BIPOLAR_PARANOID	268620009	Single major depressive episode
651	QN_AMI_MDD_BIPOLAR_PARANOID	268621008	Recurrent major depressive episodes
651	QN_AMI_MDD_BIPOLAR_PARANOID	269275003	Seafood causing toxic effect
651	QN_AMI_MDD_BIPOLAR_PARANOID	269280007	Asphyxia by bedclothes or pillow
651	QN_AMI_MDD_BIPOLAR_PARANOID	274207006	Solvents -toxic effects
651	QN_AMI_MDD_BIPOLAR_PARANOID	274912004	Self poisoning by agricultural chemical
651	QN_AMI_MDD_BIPOLAR_PARANOID	275385007	Poisoning caused by biological substance
651	QN_AMI_MDD_BIPOLAR_PARANOID	276853009	Self inflicted injury
651	QN_AMI_MDD_BIPOLAR_PARANOID	282100009	Adverse reaction to substance
651	QN_AMI_MDD_BIPOLAR_PARANOID	287186005	Attempted suicide - jumping from a high place
651	QN_AMI_MDD_BIPOLAR_PARANOID	287194003	Suicide - cut/stab
651	QN_AMI_MDD_BIPOLAR_PARANOID	290145004	Intentional aspirin poisoning
651	QN_AMI_MDD_BIPOLAR_PARANOID	290316006	Diagnostic agent poisoning
651	QN_AMI_MDD_BIPOLAR_PARANOID	290428004	Laxative poisoning
651	QN_AMI_MDD_BIPOLAR_PARANOID	290486002	General anesthetic drug poisoning
651	QN_AMI_MDD_BIPOLAR_PARANOID	290522001	Intentional trichloroethylene poisoning
651	QN_AMI_MDD_BIPOLAR_PARANOID	290888005	Selective serotonin re-uptake inhibitor poisoning
651	QN_AMI_MDD_BIPOLAR_PARANOID	290914002	Tetracyclic antidepressant drug poisoning

© 2021 Matheny ME et al. *JAMA Network Open.*

CONDITIONID	CONDITION_DESCRIPTION	SNOMED_CODE	DESCRIPTION
651	QN_AMI_MDD_BIPOLAR_PARANOID	291121009	Poisoning by anti-psychotic agent
651	QN_AMI_MDD_BIPOLAR_PARANOID	291241005	Intentional amphetamine poisoning
651	QN_AMI_MDD_BIPOLAR_PARANOID	291288009	Intentional poisoning by calcium-channel blocker
651	QN_AMI_MDD_BIPOLAR_PARANOID	291297008	Intentional anticholinesterase poisoning
651	QN_AMI_MDD_BIPOLAR_PARANOID	291299006	Alpha-adrenoceptor agonist poisoning
651	QN_AMI_MDD_BIPOLAR_PARANOID	291303001	Beta-adrenoceptor agonist poisoning
651	QN_AMI_MDD_BIPOLAR_PARANOID	291370004	Intentional anti-common cold drug poisoning
651	QN_AMI_MDD_BIPOLAR_PARANOID	291473001	Aminoglycosides poisoning
651	QN_AMI_MDD_BIPOLAR_PARANOID	291658008	Intentional tetracycline poisoning
651	QN_AMI_MDD_BIPOLAR_PARANOID	291662002	Intentional chloramphenicol poisoning
651	QN_AMI_MDD_BIPOLAR_PARANOID	291713007	Pertussis vaccine poisoning
651	QN_AMI_MDD_BIPOLAR_PARANOID	291766005	Intentional smallpox vaccine poisoning
651	QN_AMI_MDD_BIPOLAR_PARANOID	291825002	Sex hormone poisoning
651	QN_AMI_MDD_BIPOLAR_PARANOID	291830003	Intentional oral contraceptive poisoning
651	QN_AMI_MDD_BIPOLAR_PARANOID	291969008	Loop diuretic poisoning

© 2021 Matheny ME et al. *JAMA Network Open.*

CONDITIONID	CONDITION_DESCRIPTION	SNOMED_CODE	DESCRIPTION
651	QN_AMI_MDD_BIPOLAR_PARANOID	291989009	Intentional poisoning by angiotensin-converting enzyme inhibitor
651	QN_AMI_MDD_BIPOLAR_PARANOID	371596008	Bipolar I disorder
651	QN_AMI_MDD_BIPOLAR_PARANOID	371599001	Severe bipolar I disorder
651	QN_AMI_MDD_BIPOLAR_PARANOID	419639006	Poisoning by anti-infective agent
651	QN_AMI_MDD_BIPOLAR_PARANOID	426487006	Toxic effect from eating shellfish
651	QN_AMI_MDD_BIPOLAR_PARANOID	430852001	Severe major depression, single episode, with psychotic features
651	QN_AMI_MDD_BIPOLAR_PARANOID	439014005	Poisoning by propionic acid derivative
651	QN_AMI_MDD_BIPOLAR_PARANOID	446159001	Poisoning by macrolide
651	QN_AMI_MDD_BIPOLAR_PARANOID	699022007	Poisoning by anticoagulant antagonist AND/OR coagulant
651	QN_AMI_MDD_BIPOLAR_PARANOID	699064007	Poisoning by antimalarial and drug acting on blood protozoa
651	QN_AMI_MDD_BIPOLAR_PARANOID	764731000000103	Single manic episode in partial remission
654	QN_AMI_DEPRESSION	35489007	Depressive disorder
654	QN_AMI_DEPRESSION	76105009	Cyclothymia
654	QN_AMI_DEPRESSION	78667006	Dysthymia
654	QN_AMI_DEPRESSION	192049004	Prolonged depressive adjustment reaction

© 2021 Matheny ME et al. *JAMA Network Open.*

CONDITIONID	CONDITION_DESCRIPTION	SNOMED_CODE	DESCRIPTION
654	QN_AMI_DEPRESSION	268620009	Single major depressive episode
655	QN_AMI_ANXIETY	18193002	Hypochondriasis
655	QN_AMI_ANXIETY	18393005	Undifferentiated somatoform disorder
655	QN_AMI_ANXIETY	19887002	Claustrophobia
655	QN_AMI_ANXIETY	20734000	Psychologic conversion disorder
655	QN_AMI_ANXIETY	21897009	Generalized anxiety disorder
655	QN_AMI_ANXIETY	24315006	Factitious disorder with combined physical AND psychological symptoms
655	QN_AMI_ANXIETY	25501002	Social phobia
655	QN_AMI_ANXIETY	31297008	Somatoform disorder
655	QN_AMI_ANXIETY	35607004	Panic disorder with agoraphobia
655	QN_AMI_ANXIETY	44376007	Dissociative disorder
655	QN_AMI_ANXIETY	47505003	Posttraumatic stress disorder
655	QN_AMI_ANXIETY	50705009	Factitious disorder
655	QN_AMI_ANXIETY	54307006	Zoophobia
655	QN_AMI_ANXIETY	56576003	Panic disorder without agoraphobia
655	QN_AMI_ANXIETY	56882008	Anorexia nervosa
655	QN_AMI_ANXIETY	58963008	Acrophobia
655	QN_AMI_ANXIETY	61569007	Agoraphobia without history of panic disorder
655	QN_AMI_ANXIETY	62351001	Generalized social phobia
655	QN_AMI_ANXIETY	63393005	Anorexia nervosa, binge-eating purging type

© 2021 Matheny ME et al. *JAMA Network Open.*

CONDITIONID	CONDITION_DESCRIPTION	SNOMED_CODE	DESCRIPTION
655	QN_AMI_ANXIETY	70691001	Agoraphobia
655	QN_AMI_ANXIETY	77675002	Anorexia nervosa, restricting type
655	QN_AMI_ANXIETY	78004001	Bulimia nervosa
655	QN_AMI_ANXIETY	82415003	Agoraphobia without history of panic disorder without limited symptom attacks
655	QN_AMI_ANXIETY	83482000	Body dysmorphic disorder
655	QN_AMI_ANXIETY	102916005	Arachnophobia
655	QN_AMI_ANXIETY	102917001	Fear of blood
655	QN_AMI_ANXIETY	102929005	Androphobia
655	QN_AMI_ANXIETY	102930000	Gynephobia
655	QN_AMI_ANXIETY	162246009	Sensory symptoms
655	QN_AMI_ANXIETY	191714002	Dissociative convulsions
655	QN_AMI_ANXIETY	191736004	Obsessive-compulsive disorder
655	QN_AMI_ANXIETY	225633003	Fear of thunderstorm
655	QN_AMI_ANXIETY	247853008	Fear of flying
655	QN_AMI_ANXIETY	268715000	Dissociative motor disorder
655	QN_AMI_ANXIETY	276300008	Dissociative stupor
655	QN_AMI_ANXIETY	313182004	Chronic post-traumatic stress disorder
655	QN_AMI_ANXIETY	386808001	Phobia
655	QN_AMI_ANXIETY	386810004	Phobic disorder
655	QN_AMI_ANXIETY	397923000	Somatization disorder
655	QN_AMI_ANXIETY	430744005	Factitious disorder with predominantly physical signs and symptoms
655	QN_AMI_ANXIETY	430751001	Factitious disorder with

© 2021 Matheny ME et al. *JAMA Network Open.*

CONDITIONID	CONDITION_DESCRIPTION	SNOMED_CODE	DESCRIPTION
			predominantly psychological signs and symptoms
655	QN_AMI_ANXIETY	699389008	Fear of medical treatment
655	QN_AMI_ANXIETY	288241000119105	Fear of bridges
655	QN_AMI_ANXIETY	288251000119107	Fear of injury
678	QN_AMI_CHF	5148006	Hypertensive heart disease with congestive heart failure
678	QN_AMI_CHF	11399002	Pulmonary arterial hypertension
678	QN_AMI_CHF	26117009	Diphtheritic myocarditis
678	QN_AMI_CHF	33258008	Primary eosinophilic endomyocardial restrictive cardiomyopathy
678	QN_AMI_CHF	39785005	Disorder of pulmonary circulation
678	QN_AMI_CHF	42343007	Congestive heart failure
678	QN_AMI_CHF	45227007	Hypertrophic obstructive cardiomyopathy
678	QN_AMI_CHF	45650007	Kyphoscoliotic heart disease
678	QN_AMI_CHF	46113002	Hypertensive heart failure
678	QN_AMI_CHF	49584005	Acute cor pulmonale
678	QN_AMI_CHF	50920009	Myocarditis
678	QN_AMI_CHF	57809008	Myocardial disease
678	QN_AMI_CHF	59282003	Pulmonary embolism

© 2021 Matheny ME et al. *JAMA Network Open.*

CONDITIONID	CONDITION_DESCRIPTION	SNOMED_CODE	DESCRIPTION
678	QN_AMI_CHF	64077000	Myocardial degeneration
678	QN_AMI_CHF	65457005	Endocardial fibroelastosis
678	QN_AMI_CHF	67189007	Acute pulmonary heart disease
678	QN_AMI_CHF	77737007	Benign hypertensive heart disease with congestive heart failure
678	QN_AMI_CHF	79955004	Chronic cor pulmonale
678	QN_AMI_CHF	82523003	Congestive rheumatic heart failure
678	QN_AMI_CHF	83105008	Malignant hypertensive heart disease with congestive heart failure
678	QN_AMI_CHF	83521008	Dilated cardiomyopathy secondary to alcohol
678	QN_AMI_CHF	84114007	Heart failure
678	QN_AMI_CHF	85232009	Left heart failure
678	QN_AMI_CHF	85898001	Cardiomyopathy
678	QN_AMI_CHF	87837008	Chronic pulmonary heart disease
678	QN_AMI_CHF	88223008	Secondary pulmonary hypertension
678	QN_AMI_CHF	91529004	Rheumatic heart valve failure
678	QN_AMI_CHF	111289009	Arteriovenous fistula of pulmonary vessels
678	QN_AMI_CHF	194779001	Hypertensive heart and renal disease

© 2021 Matheny ME et al. *JAMA Network Open.*

CONDITIONID	CONDITION_DESCRIPTION	SNOMED_CODE	DESCRIPTION
			with (congestive) heart failure
678	QN_AMI_CHF	194781004	Hypertensive heart and renal disease with both (congestive) heart failure and renal failure
678	QN_AMI_CHF	194892009	Pulmonary artery aneurysm
678	QN_AMI_CHF	195023001	Nutritional and metabolic cardiomyopathies
678	QN_AMI_CHF	195029002	Cardiomyopathy associated with another disorder
678	QN_AMI_CHF	233873004	Hypertrophic cardiomyopathy
678	QN_AMI_CHF	274096000	Pulmonary heart disease
678	QN_AMI_CHF	398754006	Restrictive cardiomyopathy with endomyocardial fibrosis
678	QN_AMI_CHF	399020009	Dilated cardiomyopathy
678	QN_AMI_CHF	415295002	Restrictive cardiomyopathy
678	QN_AMI_CHF	417996009	Systolic heart failure
678	QN_AMI_CHF	418304008	Diastolic heart failure
678	QN_AMI_CHF	441481004	Chronic systolic heart failure
678	QN_AMI_CHF	441530006	Chronic diastolic heart failure
678	QN_AMI_CHF	441557008	Septic pulmonary embolism

© 2021 Matheny ME et al. *JAMA Network Open.*

CONDITIONID	CONDITION_DESCRIPTION	SNOMED_CODE	DESCRIPTION
678	QN_AMI_CHF	442304009	Combined systolic and diastolic dysfunction
678	QN_AMI_CHF	443253003	Acute on chronic systolic heart failure
678	QN_AMI_CHF	443254009	Acute systolic heart failure
678	QN_AMI_CHF	443343001	Acute diastolic heart failure
678	QN_AMI_CHF	443344007	Acute on chronic diastolic heart failure
678	QN_AMI_CHF	153931000119109	Acute combined systolic and diastolic heart failure
678	QN_AMI_CHF	153941000119100	Chronic combined systolic and diastolic heart failure
678	QN_AMI_CHF	153951000119103	Acute on chronic combined systolic and diastolic heart failure
678	QN_AMI_CHF	204981000119101	Cardiomyopathy due to viral infection
681	QN_AMI_ANGINA	23687008	Coronary artery spasm
681	QN_AMI_ANGINA	87343002	Prinzmetal angina
681	QN_AMI_ANGINA	129573006	Atherosclerotic occlusive disease
681	QN_AMI_ANGINA	194828000	Angina pectoris
681	QN_AMI_ANGINA	429673002	Arteriosclerosis of coronary artery bypass graft
681	QN_AMI_ANGINA	442224005	Arteriosclerosis of autologous vein coronary artery bypass graft

© 2021 Matheny ME et al. *JAMA Network Open.*

CONDITIONID	CONDITION_DESCRIPTION	SNOMED_CODE	DESCRIPTION
681	QN_AMI_ANGINA	442240008	Arteriosclerosis of nonautologous coronary artery bypass graft
681	QN_AMI_ANGINA	444855007	Arteriosclerosis of coronary artery bypass graft of transplanted heart
681	QN_AMI_ANGINA	1641000119107	Coronary arteriosclerosis in native artery
681	QN_AMI_ANGINA	6661000119101	Coronary arteriosclerosis in native artery of transplanted heart
681	QN_AMI_ANGINA	285141000119106	Arteriosclerosis of autologous arterial coronary artery bypass graft
682	QN_AMI_CAD	1755008	Old myocardial infarction
682	QN_AMI_CAD	4557003	Preinfarction syndrome
682	QN_AMI_CAD	28574005	Congenital anomaly of coronary artery
682	QN_AMI_CAD	50570003	Aneurysm of coronary vessels
682	QN_AMI_CAD	53741008	Coronary arteriosclerosis
682	QN_AMI_CAD	63739005	Coronary occlusion
682	QN_AMI_CAD	65340007	Aneurysm of heart
682	QN_AMI_CAD	128599005	Structural disorder of heart
682	QN_AMI_CAD	194828000	Angina pectoris
682	QN_AMI_CAD	194849004	Generalized ischemic myocardial dysfunction
682	QN_AMI_CAD	233823002	Silent myocardial ischemia

© 2021 Matheny ME et al. *JAMA Network Open.*

CONDITIONID	CONDITION_DESCRIPTION	SNOMED_CODE	DESCRIPTION
682	QN_AMI_CAD	413838009	Chronic ischemic heart disease
682	QN_AMI_CAD	414795007	Myocardial ischemia
682	QN_AMI_CAD	429673002	Arteriosclerosis of coronary artery bypass graft
682	QN_AMI_CAD	442224005	Arteriosclerosis of autologous vein coronary artery bypass graft
682	QN_AMI_CAD	442240008	Arteriosclerosis of nonautologous coronary artery bypass graft
682	QN_AMI_CAD	442421004	Arteriosclerosis of arterial coronary artery bypass graft
682	QN_AMI_CAD	444855007	Arteriosclerosis of coronary artery bypass graft of transplanted heart
682	QN_AMI_CAD	1641000119107	Coronary arteriosclerosis in native artery
682	QN_AMI_CAD	6661000119101	Coronary arteriosclerosis in native artery of transplanted heart
682	QN_AMI_CAD	285141000119106	Arteriosclerosis of autologous arterial coronary artery bypass graft
683	QN_AMI_HEART_INFECT	3238004	Pericarditis
683	QN_AMI_HEART_INFECT	4834000	Typhoid fever
683	QN_AMI_HEART_INFECT	9241004	Gonococcal heart disease
683	QN_AMI_HEART_INFECT	12232008	Syphilitic aneurysm of aorta
683	QN_AMI_HEART_INFECT	15555002	Acute pericarditis
683	QN_AMI_HEART_INFECT	20735004	Syphilitic aortitis

© 2021 Matheny ME et al. *JAMA Network Open.*

CONDITIONID	CONDITION_DESCRIPTION	SNOMED_CODE	DESCRIPTION
683	QN_AMI_HEART_INFECT	22653005	Myocarditis due to infectious agent
683	QN_AMI_HEART_INFECT	23412002	Hemopericardium
683	QN_AMI_HEART_INFECT	30242009	Scarlet fever
683	QN_AMI_HEART_INFECT	31993003	Toxic myocarditis
683	QN_AMI_HEART_INFECT	33618002	Meningococcal carditis
683	QN_AMI_HEART_INFECT	34845009	Meningococcal endocarditis
683	QN_AMI_HEART_INFECT	35304003	Cardiac tamponade
683	QN_AMI_HEART_INFECT	37715009	Adhesive pericarditis
683	QN_AMI_HEART_INFECT	41739008	Infectious pericarditis
683	QN_AMI_HEART_INFECT	42999000	Chronic adhesive pericarditis
683	QN_AMI_HEART_INFECT	46701001	Acute myocarditis
683	QN_AMI_HEART_INFECT	52535005	Chronic constrictive pericarditis
683	QN_AMI_HEART_INFECT	55855009	Disorder of pericardium
683	QN_AMI_HEART_INFECT	63462008	Mumps myocarditis
683	QN_AMI_HEART_INFECT	63553008	Candidal endocarditis
683	QN_AMI_HEART_INFECT	64043005	Bacterial myocarditis
683	QN_AMI_HEART_INFECT	66704002	Meningococcal pericarditis
683	QN_AMI_HEART_INFECT	67391006	Syphilitic endocarditis
683	QN_AMI_HEART_INFECT	70189005	Viral pericarditis
683	QN_AMI_HEART_INFECT	76534005	Myocarditis due to acquired toxoplasmosis
683	QN_AMI_HEART_INFECT	82323002	Late congenital syphilis
683	QN_AMI_HEART_INFECT	83883001	Cardiovascular syphilis

© 2021 Matheny ME et al. *JAMA Network Open.*

CONDITIONID	CONDITION_DESCRIPTION	SNOMED_CODE	DESCRIPTION
683	QN_AMI_HEART_INFECT	85598007	Constrictive pericarditis
683	QN_AMI_HEART_INFECT	89141000	Viral myocarditis
683	QN_AMI_HEART_INFECT	91025000	Idiopathic myocarditis
683	QN_AMI_HEART_INFECT	91357005	Acute endocarditis
683	QN_AMI_HEART_INFECT	91468009	Meningococcal myocarditis
683	QN_AMI_HEART_INFECT	186318004	Listerial endocarditis
683	QN_AMI_HEART_INFECT	186755002	Viral carditis
683	QN_AMI_HEART_INFECT	187040006	Histoplasma capsulatum with pericarditis
683	QN_AMI_HEART_INFECT	187041005	Histoplasma capsulatum with endocarditis
683	QN_AMI_HEART_INFECT	187050007	Histoplasma duboisii with pericarditis
683	QN_AMI_HEART_INFECT	187051006	Histoplasma duboisii with endocarditis
683	QN_AMI_HEART_INFECT	187059008	Histoplasmosis with pericarditis
683	QN_AMI_HEART_INFECT	187195003	Toxoplasma myocarditis
683	QN_AMI_HEART_INFECT	194921005	Acute and subacute endocarditis
683	QN_AMI_HEART_INFECT	194926000	Acute and subacute infective endocarditis associated with another disorder
683	QN_AMI_HEART_INFECT	194942007	Acute myocarditis associated with another disorder
683	QN_AMI_HEART_INFECT	194970009	Pericardial effusion - noninflammatory

© 2021 Matheny ME et al. *JAMA Network Open.*

CONDITIONID	CONDITION_DESCRIPTION	SNOMED_CODE	DESCRIPTION
683	QN_AMI_HEART_INFECT	195033009	Sarcoid heart muscle disease
683	QN_AMI_HEART_INFECT	266235007	Acute idiopathic pericarditis
683	QN_AMI_HEART_INFECT	266237004	Acute and subacute bacterial endocarditis
683	QN_AMI_HEART_INFECT	266238009	Isolated (Fiedler's) myocarditis
683	QN_AMI_HEART_INFECT	309762007	Systemic lupus erythematosus with pericarditis
683	QN_AMI_HEART_INFECT	399617002	Carditis
683	QN_AMI_HEART_INFECT	459057007	Viral endocarditis
684	QN_AMI_VALVE_RHEUM_HEART_DZ	368009	Heart valve disorder
684	QN_AMI_VALVE_RHEUM_HEART_DZ	787001	Rheumatic mitral stenosis with regurgitation
684	QN_AMI_VALVE_RHEUM_HEART_DZ	4374004	Congenital anomaly of tricuspid valve
684	QN_AMI_VALVE_RHEUM_HEART_DZ	7305005	Coarctation of aorta
684	QN_AMI_VALVE_RHEUM_HEART_DZ	7438000	Congenital atresia of aorta
684	QN_AMI_VALVE_RHEUM_HEART_DZ	8722008	Aortic valve disorder
684	QN_AMI_VALVE_RHEUM_HEART_DZ	11851006	Mitral valve disorder
684	QN_AMI_VALVE_RHEUM_HEART_DZ	12023003	Rheumatic disease of aortic valve
684	QN_AMI_VALVE_RHEUM_HEART_DZ	13213009	Congenital heart disease
684	QN_AMI_VALVE_RHEUM_HEART_DZ	15096009	Congenital insufficiency of pulmonary valve
684	QN_AMI_VALVE_RHEUM_HEART_DZ	15676002	Acute rheumatic pericarditis
684	QN_AMI_VALVE_RHEUM_HEART_DZ	16063004	Rheumatic disease of heart valve

© 2021 Matheny ME et al. *JAMA Network Open.*

CONDITIONID	CONDITION_DESCRIPTION	SNOMED_CODE	DESCRIPTION
684	QN_AMI_VALVE_RHEUM_HEART_DZ	16440002	Rheumatic disease of mitral AND aortic valves
684	QN_AMI_VALVE_RHEUM_HEART_DZ	17759006	Rheumatic aortic stenosis with regurgitation
684	QN_AMI_VALVE_RHEUM_HEART_DZ	18192007	Acute rheumatic endocarditis
684	QN_AMI_VALVE_RHEUM_HEART_DZ	18546004	Congenital stenosis of aortic valve
684	QN_AMI_VALVE_RHEUM_HEART_DZ	18687009	Rheumatic disease of pulmonary valve
684	QN_AMI_VALVE_RHEUM_HEART_DZ	23685000	Rheumatic heart disease
684	QN_AMI_VALVE_RHEUM_HEART_DZ	24363009	Rheumatic fever without heart involvement
684	QN_AMI_VALVE_RHEUM_HEART_DZ	28656008	Congenital insufficiency of aortic valve
684	QN_AMI_VALVE_RHEUM_HEART_DZ	29928006	Congenital insufficiency of mitral valve
684	QN_AMI_VALVE_RHEUM_HEART_DZ	31085000	Rheumatic mitral regurgitation
684	QN_AMI_VALVE_RHEUM_HEART_DZ	36233006	Congenital stenosis of tricuspid valve
684	QN_AMI_VALVE_RHEUM_HEART_DZ	46826000	Rheumatic chorea
684	QN_AMI_VALVE_RHEUM_HEART_DZ	48872007	Rheumatic endocarditis
684	QN_AMI_VALVE_RHEUM_HEART_DZ	49699002	Rheumatic disease of tricuspid valve
684	QN_AMI_VALVE_RHEUM_HEART_DZ	56819008	Endocarditis
684	QN_AMI_VALVE_RHEUM_HEART_DZ	57803009	Rheumatic chorea with heart involvement
684	QN_AMI_VALVE_RHEUM_HEART_DZ	59877000	Congenital anomaly of aorta
684	QN_AMI_VALVE_RHEUM_HEART_DZ	67278007	Congenital stenosis of pulmonary valve

© 2021 Matheny ME et al. *JAMA Network Open.*

CONDITIONID	CONDITION_DESCRIPTION	SNOMED_CODE	DESCRIPTION
684	QN_AMI_VALVE_RHEUM_HEART_DZ	67696008	Rheumatic tricuspid valve regurgitation
684	QN_AMI_VALVE_RHEUM_HEART_DZ	72011007	Rheumatic aortic stenosis
684	QN_AMI_VALVE_RHEUM_HEART_DZ	76267008	Pulmonary valve disorder
684	QN_AMI_VALVE_RHEUM_HEART_DZ	78031003	Rheumatic aortic regurgitation
684	QN_AMI_VALVE_RHEUM_HEART_DZ	78069008	Chronic rheumatic pericarditis
684	QN_AMI_VALVE_RHEUM_HEART_DZ	79439001	Congenital anomaly of aortic arch
684	QN_AMI_VALVE_RHEUM_HEART_DZ	82458004	Congenital stenosis of mitral valve
684	QN_AMI_VALVE_RHEUM_HEART_DZ	83898004	Rheumatic disease of mitral valve
684	QN_AMI_VALVE_RHEUM_HEART_DZ	86466006	Rheumatic mitral stenosis
684	QN_AMI_VALVE_RHEUM_HEART_DZ	88318005	Rheumatic tricuspid valve stenosis
684	QN_AMI_VALVE_RHEUM_HEART_DZ	89736004	Valvular endocarditis
684	QN_AMI_VALVE_RHEUM_HEART_DZ	123950001	Rheumatic chorea without heart involvement
684	QN_AMI_VALVE_RHEUM_HEART_DZ	194708008	Rheumatic fever with heart involvement
684	QN_AMI_VALVE_RHEUM_HEART_DZ	194709000	Acute rheumatic myocarditis
684	QN_AMI_VALVE_RHEUM_HEART_DZ	194726006	Mitral stenosis with insufficiency
684	QN_AMI_VALVE_RHEUM_HEART_DZ	194727002	Non-rheumatic mitral valve stenosis
684	QN_AMI_VALVE_RHEUM_HEART_DZ	194732001	Diseases of mitral and aortic valves

© 2021 Matheny ME et al. *JAMA Network Open.*

CONDITIONID	CONDITION_DESCRIPTION	SNOMED_CODE	DESCRIPTION
684	QN_AMI_VALVE_RHEUM_HEART_DZ	194734000	Mitral stenosis and aortic insufficiency
684	QN_AMI_VALVE_RHEUM_HEART_DZ	194735004	Mitral insufficiency and aortic stenosis
684	QN_AMI_VALVE_RHEUM_HEART_DZ	194736003	Mitral and aortic incompetence
684	QN_AMI_VALVE_RHEUM_HEART_DZ	194737007	Multiple mitral and aortic valve involvement
684	QN_AMI_VALVE_RHEUM_HEART_DZ	194741006	Rheumatic tricuspid stenosis and insufficiency
684	QN_AMI_VALVE_RHEUM_HEART_DZ	194750008	Rheumatic myocarditis
684	QN_AMI_VALVE_RHEUM_HEART_DZ	194983005	Aortic incompetence, non-rheumatic
684	QN_AMI_VALVE_RHEUM_HEART_DZ	194987006	Aortic valve stenosis with insufficiency
684	QN_AMI_VALVE_RHEUM_HEART_DZ	194989009	Tricuspid valve disorder, non-rheumatic
684	QN_AMI_VALVE_RHEUM_HEART_DZ	194990000	Tricuspid incompetence, non-rheumatic
684	QN_AMI_VALVE_RHEUM_HEART_DZ	194991001	Tricuspid stenosis, non-rheumatic
684	QN_AMI_VALVE_RHEUM_HEART_DZ	194992008	Non-rheumatic tricuspid valve stenosis with insufficiency
684	QN_AMI_VALVE_RHEUM_HEART_DZ	194995005	Pulmonary incompetence, non-rheumatic
684	QN_AMI_VALVE_RHEUM_HEART_DZ	194997002	Pulmonary stenosis, non-rheumatic
684	QN_AMI_VALVE_RHEUM_HEART_DZ	195002007	Multiple valve disease

© 2021 Matheny ME et al. *JAMA Network Open.*

CONDITIONID	CONDITION_DESCRIPTION	SNOMED_CODE	DESCRIPTION
684	QN_AMI_VALVE_RHEUM_HEART_DZ	195003002	Disorders of both aortic and tricuspid valves
684	QN_AMI_VALVE_RHEUM_HEART_DZ	195004008	Disorders of both mitral and tricuspid valves
684	QN_AMI_VALVE_RHEUM_HEART_DZ	195005009	Combined disorders of mitral, aortic and tricuspid valves
684	QN_AMI_VALVE_RHEUM_HEART_DZ	195012000	Endocarditis associated with another disorder
684	QN_AMI_VALVE_RHEUM_HEART_DZ	204339005	Congenital pulmonary valve abnormality
684	QN_AMI_VALVE_RHEUM_HEART_DZ	204354004	Congenital tricuspid atresia and stenosis
684	QN_AMI_VALVE_RHEUM_HEART_DZ	204357006	Ebstein's anomaly
684	QN_AMI_VALVE_RHEUM_HEART_DZ	204431007	Atresia and stenosis of aorta
684	QN_AMI_VALVE_RHEUM_HEART_DZ	218728005	Interrupted aortic arch
684	QN_AMI_VALVE_RHEUM_HEART_DZ	233848004	Disorder of endocardium and heart valve
684	QN_AMI_VALVE_RHEUM_HEART_DZ	234119001	Arterial malformation
684	QN_AMI_VALVE_RHEUM_HEART_DZ	268180007	Right hypoplastic heart syndrome
684	QN_AMI_VALVE_RHEUM_HEART_DZ	268185002	Supravalvar aortic stenosis
684	QN_AMI_VALVE_RHEUM_HEART_DZ	274097009	Non-rheumatic heart valve disorder
684	QN_AMI_VALVE_RHEUM_HEART_DZ	301105002	Pulmonary valve lesion
684	QN_AMI_VALVE_RHEUM_HEART_DZ	312591002	Acute rheumatic heart disease

© 2021 Matheny ME et al. *JAMA Network Open.*

CONDITIONID	CONDITION_DESCRIPTION	SNOMED_CODE	DESCRIPTION
684	QN_AMI_VALVE_RHEUM_HEART_DZ	315615007	Non-rheumatic aortic sclerosis
684	QN_AMI_VALVE_RHEUM_HEART_DZ	409712001	Mitral valve prolapse
684	QN_AMI_VALVE_RHEUM_HEART_DZ	703178004	Non-rheumatic pulmonary valve stenosis with regurgitation
684	QN_AMI_VALVE_RHEUM_HEART_DZ	708121009	Non-rheumatic mitral valve disease
684	QN_AMI_VALVE_RHEUM_HEART_DZ	72181000119109	Endocarditis due to systemic lupus erythematosus
685	QN_AMI_MAJOR_CONGEN_HEART	7368005	Double outlet left ventricle
685	QN_AMI_MAJOR_CONGEN_HEART	7484005	Double outlet right ventricle
685	QN_AMI_MAJOR_CONGEN_HEART	9904008	Congenital anomaly of cardiovascular system
685	QN_AMI_MAJOR_CONGEN_HEART	13213009	Congenital heart disease
685	QN_AMI_MAJOR_CONGEN_HEART	26780008	Coarctation of pulmonary artery
685	QN_AMI_MAJOR_CONGEN_HEART	45503006	Common ventricle
685	QN_AMI_MAJOR_CONGEN_HEART	59631007	Anomalous pulmonary venous drainage
685	QN_AMI_MAJOR_CONGEN_HEART	61959006	Common arterial trunk
685	QN_AMI_MAJOR_CONGEN_HEART	62067003	Hypoplastic left heart syndrome
685	QN_AMI_MAJOR_CONGEN_HEART	68237008	Partial anomalous pulmonary venous connection
685	QN_AMI_MAJOR_CONGEN_HEART	77978002	Persistent left superior vena cava
685	QN_AMI_MAJOR_CONGEN_HEART	81990004	Cor biloculare

© 2021 Matheny ME et al. *JAMA Network Open.*

CONDITIONID	CONDITION_DESCRIPTION	SNOMED_CODE	DESCRIPTION
685	QN_AMI_MAJOR_CONGEN_HEART	83330001	Patent ductus arteriosus
685	QN_AMI_MAJOR_CONGEN_HEART	83799000	Corrected transposition of great vessels
685	QN_AMI_MAJOR_CONGEN_HEART	86299006	Tetralogy of Fallot
685	QN_AMI_MAJOR_CONGEN_HEART	88244008	Congenital stenosis of vena cava
685	QN_AMI_MAJOR_CONGEN_HEART	111323005	Total anomalous pulmonary venous return
685	QN_AMI_MAJOR_CONGEN_HEART	204296002	Discordant ventriculoarterial connection
685	QN_AMI_MAJOR_CONGEN_HEART	204342004	Congenital atresia of pulmonary valve
685	QN_AMI_MAJOR_CONGEN_HEART	204443008	Pulmonary artery atresia
685	QN_AMI_MAJOR_CONGEN_HEART	204451006	Anomalies of great veins
685	QN_AMI_MAJOR_CONGEN_HEART	253272009	Congenital abnormality of cardiac connection
685	QN_AMI_MAJOR_CONGEN_HEART	253277003	Discordant atrioventricular connection
685	QN_AMI_MAJOR_CONGEN_HEART	253281003	Double inlet ventricle
686	QN_AMI_OTHER_CONGEN_HEART	1519002	Congenital phlebectasia
686	QN_AMI_OTHER_CONGEN_HEART	9904008	Congenital anomaly of cardiovascular system
686	QN_AMI_OTHER_CONGEN_HEART	10818008	Congenital malposition of heart
686	QN_AMI_OTHER_CONGEN_HEART	13213009	Congenital heart disease
686	QN_AMI_OTHER_CONGEN_HEART	15459006	Endocardial cushion defect

© 2021 Matheny ME et al. *JAMA Network Open.*

CONDITIONID	CONDITION_DESCRIPTION	SNOMED_CODE	DESCRIPTION
686	QN_AMI_OTHER_CONGEN_HEART	17024001	Aortopulmonary window
686	QN_AMI_OTHER_CONGEN_HEART	17718000	Ostium primum defect
686	QN_AMI_OTHER_CONGEN_HEART	27637000	Dextrocardia
686	QN_AMI_OTHER_CONGEN_HEART	30288003	Ventricular septal defect
686	QN_AMI_OTHER_CONGEN_HEART	36110001	Congenital anomaly of pulmonary artery
686	QN_AMI_OTHER_CONGEN_HEART	43876007	Situs inversus viscerum
686	QN_AMI_OTHER_CONGEN_HEART	55510008	Cor triatriatum
686	QN_AMI_OTHER_CONGEN_HEART	59494005	Congenital septal defect of heart
686	QN_AMI_OTHER_CONGEN_HEART	70142008	Atrial septal defect
686	QN_AMI_OTHER_CONGEN_HEART	73660006	Congenital subaortic stenosis
686	QN_AMI_OTHER_CONGEN_HEART	74877002	Congenital anomaly of spine
686	QN_AMI_OTHER_CONGEN_HEART	84773003	Congenital anomaly of lower limb
686	QN_AMI_OTHER_CONGEN_HEART	95441000	Pulmonary artery stenosis
686	QN_AMI_OTHER_CONGEN_HEART	95474000	Arteriovenous malformation of kidney
686	QN_AMI_OTHER_CONGEN_HEART	111322000	Congenital anomaly of pulmonary veins
686	QN_AMI_OTHER_CONGEN_HEART	128347007	Congenital anomaly of gastrointestinal tract
686	QN_AMI_OTHER_CONGEN_HEART	204315000	Ostium secundum type atrial septal defect
686	QN_AMI_OTHER_CONGEN_HEART	204370002	Infundibular pulmonic stenosis

© 2021 Matheny ME et al. *JAMA Network Open.*

CONDITIONID	CONDITION_DESCRIPTION	SNOMED_CODE	DESCRIPTION
686	QN_AMI_OTHER_CONGEN_HEART	204461004	Portal vein-hepatic artery fistula
686	QN_AMI_OTHER_CONGEN_HEART	204469002	Absence or hypoplasia of the umbilical artery
686	QN_AMI_OTHER_CONGEN_HEART	204493007	Arteriovenous malformation of precerebral vessels
686	QN_AMI_OTHER_CONGEN_HEART	205769006	Situs inversus with levocardia
686	QN_AMI_OTHER_CONGEN_HEART	206597007	Persistent fetal circulation
686	QN_AMI_OTHER_CONGEN_HEART	234141001	Congenital arteriovenous malformation
686	QN_AMI_OTHER_CONGEN_HEART	253273004	Cardiac septal defects
686	QN_AMI_OTHER_CONGEN_HEART	253335001	Isomerism of atrial appendages
686	QN_AMI_OTHER_CONGEN_HEART	253414002	Atrioventricular septal defect and common atrioventricular junction
686	QN_AMI_OTHER_CONGEN_HEART	268174004	Bulbus cordis and cardiac septal closure anomalies
686	QN_AMI_OTHER_CONGEN_HEART	271432005	Congenital renal artery stenosis
686	QN_AMI_OTHER_CONGEN_HEART	275519006	Peripheral arteriovenous malformation
686	QN_AMI_OTHER_CONGEN_HEART	303070000	Pulmonary arteriovenous malformation
686	QN_AMI_OTHER_CONGEN_HEART	430166008	Congenital anomaly of peripheral blood vessel
686	QN_AMI_OTHER_CONGEN_HEART	432293006	Congenital anomaly of blood

© 2021 Matheny ME et al. *JAMA Network Open.*

CONDITIONID	CONDITION_DESCRIPTION	SNOMED_CODE	DESCRIPTION
			vessel of upper limb
686	QN_AMI_OTHER_CONGEN_HEART	432461000	Congenital anomaly of renal blood vessel
686	QN_AMI_OTHER_CONGEN_HEART	703331008	Arteriovenous malformation of limb
686	QN_AMI_OTHER_CONGEN_HEART	83031000119105	Congenital malformation of nasal septum
687	QN_AMI_HTN_CARDIAC	36221001	Benign hypertensive heart disease
687	QN_AMI_HTN_CARDIAC	60899001	Hypertensive heart disease without congestive heart failure
687	QN_AMI_HTN_CARDIAC	64715009	Hypertensive heart disease
687	QN_AMI_HTN_CARDIAC	66052004	Benign hypertensive heart AND renal disease
687	QN_AMI_HTN_CARDIAC	86234004	Hypertensive heart AND renal disease
687	QN_AMI_HTN_CARDIAC	194780003	Hypertensive heart and renal disease with renal failure
687	QN_AMI_HTN_CARDIAC	8501000119104	Hypertensive heart and chronic kidney disease
688	QN_AMI_HTN	1201005	Benign essential hypertension
688	QN_AMI_HTN	31992008	Secondary hypertension
688	QN_AMI_HTN	59621000	Essential hypertension
688	QN_AMI_HTN	73410007	Benign secondary renovascular hypertension

© 2021 Matheny ME et al. *JAMA Network Open.*

CONDITIONID	CONDITION_DESCRIPTION	SNOMED_CODE	DESCRIPTION
688	QN_AMI_HTN	89242004	Malignant secondary hypertension
688	QN_AMI_HTN	123799005	Renovascular hypertension
688	QN_AMI_HTN	194783001	Malignant secondary renovascular hypertension
688	QN_AMI_HTN	194785008	Benign secondary hypertension
688	QN_AMI_HTN	194788005	Hypertension secondary to endocrine disorder
688	QN_AMI_HTN	367821000119106	Page kidney
694	QN_AMI_TIA_PRECEREBRAL_OCCUS	2929001	Occlusion of artery
694	QN_AMI_TIA_PRECEREBRAL_OCCUS	15258001	Subclavian steal syndrome
694	QN_AMI_TIA_PRECEREBRAL_OCCUS	20059004	Cerebral artery occlusion
694	QN_AMI_TIA_PRECEREBRAL_OCCUS	34781003	Vertebral artery syndrome
694	QN_AMI_TIA_PRECEREBRAL_OCCUS	43658003	Vertebral artery obstruction
694	QN_AMI_TIA_PRECEREBRAL_OCCUS	64009001	Basilar artery syndrome
694	QN_AMI_TIA_PRECEREBRAL_OCCUS	69798007	Carotid artery obstruction
694	QN_AMI_TIA_PRECEREBRAL_OCCUS	75543006	Cerebral embolism
694	QN_AMI_TIA_PRECEREBRAL_OCCUS	195180004	Basilar artery occlusion
694	QN_AMI_TIA_PRECEREBRAL_OCCUS	195182007	Vertebral artery occlusion
694	QN_AMI_TIA_PRECEREBRAL_OCCUS	195183002	Multiple and bilateral precerebral arterial occlusion
694	QN_AMI_TIA_PRECEREBRAL_OCCUS	195199008	Vertebrobasilar artery syndrome

© 2021 Matheny ME et al. *JAMA Network Open.*

CONDITIONID	CONDITION_DESCRIPTION	SNOMED_CODE	DESCRIPTION
694	QN_AMI_TIA_PRECEREBRAL_OCCUS	195200006	Carotid artery syndrome hemispheric
694	QN_AMI_TIA_PRECEREBRAL_OCCUS	195201005	Multiple and bilateral precerebral artery syndromes
694	QN_AMI_TIA_PRECEREBRAL_OCCUS	195209007	Middle cerebral artery syndrome
694	QN_AMI_TIA_PRECEREBRAL_OCCUS	195210002	Anterior cerebral artery syndrome
694	QN_AMI_TIA_PRECEREBRAL_OCCUS	195211003	Posterior cerebral artery syndrome
694	QN_AMI_TIA_PRECEREBRAL_OCCUS	195232006	Occlusion and stenosis of middle cerebral artery
694	QN_AMI_TIA_PRECEREBRAL_OCCUS	195233001	Occlusion and stenosis of anterior cerebral artery
694	QN_AMI_TIA_PRECEREBRAL_OCCUS	195234007	Occlusion and stenosis of posterior cerebral artery
694	QN_AMI_TIA_PRECEREBRAL_OCCUS	195235008	Occlusion and stenosis of cerebellar arteries
694	QN_AMI_TIA_PRECEREBRAL_OCCUS	266253001	Precerebral arterial occlusion
694	QN_AMI_TIA_PRECEREBRAL_OCCUS	266254007	Carotid artery occlusion
694	QN_AMI_TIA_PRECEREBRAL_OCCUS	266257000	Transient cerebral ischemia
694	QN_AMI_TIA_PRECEREBRAL_OCCUS	426651005	Bilateral carotid artery occlusion
694	QN_AMI_TIA_PRECEREBRAL_OCCUS	700467001	Reversible cerebral vasoconstriction syndrome
694	QN_AMI_TIA_PRECEREBRAL_OCCUS	703218000	Cerebral vasoconstriction syndrome

© 2021 Matheny ME et al. *JAMA Network Open.*

CONDITIONID	CONDITION_DESCRIPTION	SNOMED_CODE	DESCRIPTION
694	QN_AMI_TIA_PRECEREBRAL_OCCUS	285161000119105	Left carotid artery occlusion
694	QN_AMI_TIA_PRECEREBRAL_OCCUS	285171000119104	Right carotid artery occlusion
699	QN_AMI_PVD_W_COMP	80466000	Gas gangrene
699	QN_AMI_PVD_W_COMP	95344007	Ulcer of lower extremity
699	QN_AMI_PVD_W_COMP	95345008	Ulcer of foot
699	QN_AMI_PVD_W_COMP	195295006	Raynaud's disease
699	QN_AMI_PVD_W_COMP	301022003	Ulcer of heel
699	QN_AMI_PVD_W_COMP	372070002	Gangrenous disorder
699	QN_AMI_PVD_W_COMP	428194005	Ulcer of thigh
699	QN_AMI_PVD_W_COMP	442439008	Atherosclerosis of bypass graft of limb
699	QN_AMI_PVD_W_COMP	442693003	Atherosclerosis of autologous vein bypass graft of limb
699	QN_AMI_PVD_W_COMP	442701004	Atherosclerosis of nonautologous biological bypass graft of limb
699	QN_AMI_PVD_W_COMP	709585003	Atherosclerosis of nonautologous biological bypass graft of lower limb
699	QN_AMI_PVD_W_COMP	709587006	Atherosclerosis of autologous bypass graft of lower limb
699	QN_AMI_PVD_W_COMP	145891000119104	Atherosclerosis of native arteries of the extremities
699	QN_AMI_PVD_W_COMP	284891000119106	Ulcer of ankle due to atherosclerosis of native artery of limb
699	QN_AMI_PVD_W_COMP	284901000119105	Ulcer of calf due to atherosclerosis of

© 2021 Matheny ME et al. *JAMA Network Open.*

CONDITIONID	CONDITION_DESCRIPTION	SNOMED_CODE	DESCRIPTION
			native artery of limb
700	QN_AMI_VASC_DZ_W_COMP	9406001	Dissecting aneurysm of artery
700	QN_AMI_VASC_DZ_W_COMP	14336007	Ruptured abdominal aortic aneurysm
700	QN_AMI_VASC_DZ_W_COMP	16934004	Renal vascular disorder
700	QN_AMI_VASC_DZ_W_COMP	20427003	Postthrombotic syndrome
700	QN_AMI_VASC_DZ_W_COMP	31529002	Thrombosis of arteries of upper extremity
700	QN_AMI_VASC_DZ_W_COMP	45456005	Renal infarction
700	QN_AMI_VASC_DZ_W_COMP	51677000	Atheroembolism of renal arteries
700	QN_AMI_VASC_DZ_W_COMP	59282003	Pulmonary embolism
700	QN_AMI_VASC_DZ_W_COMP	67362008	Aortic aneurysm
700	QN_AMI_VASC_DZ_W_COMP	69352009	Varicose veins of lower extremity with ulcer AND inflammation
700	QN_AMI_VASC_DZ_W_COMP	72866009	Varicose veins of lower extremity
700	QN_AMI_VASC_DZ_W_COMP	73067008	Ruptured aortic aneurysm
700	QN_AMI_VASC_DZ_W_COMP	82196007	Vascular insufficiency of intestine
700	QN_AMI_VASC_DZ_W_COMP	82453008	Thrombosis of iliac artery
700	QN_AMI_VASC_DZ_W_COMP	90958004	Thrombosis of arteries of lower extremity
700	QN_AMI_VASC_DZ_W_COMP	91489000	Acute vascular insufficiency of intestine
700	QN_AMI_VASC_DZ_W_COMP	95344007	Ulcer of lower extremity

© 2021 Matheny ME et al. *JAMA Network Open.*

CONDITIONID	CONDITION_DESCRIPTION	SNOMED_CODE	DESCRIPTION
700	QN_AMI_VASC_DZ_W_COMP	195258006	Thoracic aortic aneurysm which has ruptured
700	QN_AMI_VASC_DZ_W_COMP	195265003	Thoracoabdominal aortic aneurysm, ruptured
700	QN_AMI_VASC_DZ_W_COMP	195317001	Embolism and thrombosis of the thoracic aorta
700	QN_AMI_VASC_DZ_W_COMP	195318006	Embolism and thrombosis of an arm or leg artery
700	QN_AMI_VASC_DZ_W_COMP	195335005	Embolism and/or thrombosis of the common iliac artery
700	QN_AMI_VASC_DZ_W_COMP	230730001	Dissection of vertebral artery
700	QN_AMI_VASC_DZ_W_COMP	233972005	Aortic bifurcation syndrome
700	QN_AMI_VASC_DZ_W_COMP	233994002	Dissection of thoracic aorta
700	QN_AMI_VASC_DZ_W_COMP	233999007	Dissection of iliac artery
700	QN_AMI_VASC_DZ_W_COMP	236489002	Cholesterol embolus syndrome
700	QN_AMI_VASC_DZ_W_COMP	266262004	Arterial embolus and thrombosis
700	QN_AMI_VASC_DZ_W_COMP	266263009	Embolism and thrombosis of the abdominal aorta
700	QN_AMI_VASC_DZ_W_COMP	274101000	Aortic thromboembolism
700	QN_AMI_VASC_DZ_W_COMP	304930004	Varicose veins of lower extremity with ulcer
700	QN_AMI_VASC_DZ_W_COMP	307816004	Leriche's syndrome
700	QN_AMI_VASC_DZ_W_COMP	308546005	Dissection of aorta
700	QN_AMI_VASC_DZ_W_COMP	408666009	Dissection of abdominal aorta

© 2021 Matheny ME et al. *JAMA Network Open.*

CONDITIONID	CONDITION_DESCRIPTION	SNOMED_CODE	DESCRIPTION
700	QN_AMI_VASC_DZ_W_COMP	423674003	Chronic peripheral venous hypertension
700	QN_AMI_VASC_DZ_W_COMP	425527003	Atheromatous embolus of lower limb
700	QN_AMI_VASC_DZ_W_COMP	427567003	Atheromatous embolus of upper limb
700	QN_AMI_VASC_DZ_W_COMP	441557008	Septic pulmonary embolism
700	QN_AMI_VASC_DZ_W_COMP	441746000	Infarction of lung due to iatrogenic pulmonary embolism
700	QN_AMI_VASC_DZ_W_COMP	442105001	Septic embolus of artery
700	QN_AMI_VASC_DZ_W_COMP	705066004	Dissection of internal carotid artery
700	QN_AMI_VASC_DZ_W_COMP	713029000	Dissection of thoracoabdominal aorta
700	QN_AMI_VASC_DZ_W_COMP	713036004	Dissection of renal artery
700	QN_AMI_VASC_DZ_W_COMP	713082007	Nonruptured cerebral aneurysm due to dissection of cerebral artery
700	QN_AMI_VASC_DZ_W_COMP	133971000119108	Chronic pulmonary embolism
700	QN_AMI_VASC_DZ_W_COMP	153811000119105	Chronic peripheral venous hypertension with lower extremity complication
701	QN_AMI_VASC_DZ	2900003	Hyperplasia of renal artery
701	QN_AMI_VASC_DZ	3827008	Aneurysm of artery of neck
701	QN_AMI_VASC_DZ	7930004	Necrosis of artery

© 2021 Matheny ME et al. *JAMA Network Open.*

CONDITIONID	CONDITION_DESCRIPTION	SNOMED_CODE	DESCRIPTION
701	QN_AMI_VASC_DZ	9250002	Celiac artery compression syndrome
701	QN_AMI_VASC_DZ	11791001	Necrotizing vasculitis
701	QN_AMI_VASC_DZ	13290008	Aneurysm of iliac artery
701	QN_AMI_VASC_DZ	20981007	Aneurysm of artery of lower extremity
701	QN_AMI_VASC_DZ	21877004	Osler hemorrhagic telangiectasia syndrome
701	QN_AMI_VASC_DZ	26660001	Dilatation of aorta
701	QN_AMI_VASC_DZ	29495008	Aneurysm of artery of upper extremity
701	QN_AMI_VASC_DZ	35239001	Aneurysm of visceral artery
701	QN_AMI_VASC_DZ	36184004	Aneurysm of renal artery
701	QN_AMI_VASC_DZ	37151006	Erythromelalgia
701	QN_AMI_VASC_DZ	40136003	Aneurysm of subclavian artery
701	QN_AMI_VASC_DZ	42861008	Thrombophlebitis of iliac vein
701	QN_AMI_VASC_DZ	45281005	Atherosclerosis of renal artery
701	QN_AMI_VASC_DZ	46126003	Rupture of artery
701	QN_AMI_VASC_DZ	52089001	Arteritis
701	QN_AMI_VASC_DZ	52403007	Thromboangiitis obliterans
701	QN_AMI_VASC_DZ	58729003	Disorder of capillaries
701	QN_AMI_VASC_DZ	63491006	Intermittent claudication
701	QN_AMI_VASC_DZ	67362008	Aortic aneurysm
701	QN_AMI_VASC_DZ	68109007	Stricture of artery
701	QN_AMI_VASC_DZ	70405009	Aneurysm of splenic artery
701	QN_AMI_VASC_DZ	70933002	Aortitis

© 2021 Matheny ME et al. *JAMA Network Open.*

CONDITIONID	CONDITION_DESCRIPTION	SNOMED_CODE	DESCRIPTION
701	QN_AMI_VASC_DZ	74883004	Thoracic aortic aneurysm without rupture
701	QN_AMI_VASC_DZ	75878002	Abdominal aortic aneurysm without rupture
701	QN_AMI_VASC_DZ	81817003	Atherosclerosis of aorta
701	QN_AMI_VASC_DZ	82196007	Vascular insufficiency of intestine
701	QN_AMI_VASC_DZ	82385007	Budd-Chiari syndrome
701	QN_AMI_VASC_DZ	83938003	Thrombosis of vena cava
701	QN_AMI_VASC_DZ	90507008	Disorder of vein
701	QN_AMI_VASC_DZ	95452006	Thrombophlebitis of deep veins of upper extremities
701	QN_AMI_VASC_DZ	111354009	Chronic vascular insufficiency of intestine
701	QN_AMI_VASC_DZ	128054009	Deep venous thrombosis of upper extremity
701	QN_AMI_VASC_DZ	128321003	Vascular disorder of intestine
701	QN_AMI_VASC_DZ	195410000	Thrombophlebitis of the femoral vein
701	QN_AMI_VASC_DZ	195437003	Embolism and thrombosis of the vena cava
701	QN_AMI_VASC_DZ	195438008	Embolism and thrombosis of the renal vein
701	QN_AMI_VASC_DZ	233981004	Arterial aneurysm
701	QN_AMI_VASC_DZ	233984007	Thoracoabdominal aortic aneurysm
701	QN_AMI_VASC_DZ	233985008	Abdominal aortic aneurysm

© 2021 Matheny ME et al. *JAMA Network Open.*

CONDITIONID	CONDITION_DESCRIPTION	SNOMED_CODE	DESCRIPTION
701	QN_AMI_VASC_DZ	233988005	Carotid artery aneurysm
701	QN_AMI_VASC_DZ	234061005	Internal jugular vein stenosis
701	QN_AMI_VASC_DZ	251036003	Aortic root dilatation
701	QN_AMI_VASC_DZ	266259002	Arterial, arteriole and capillary disease
701	QN_AMI_VASC_DZ	266267005	Deep vein phlebitis and thrombophlebitis of the leg
701	QN_AMI_VASC_DZ	276494008	Phlebitis and/or thrombophlebitis of iliac vein
701	QN_AMI_VASC_DZ	281596000	Thrombosis of superior vena cava
701	QN_AMI_VASC_DZ	297156001	Axillary vein thrombosis
701	QN_AMI_VASC_DZ	309735004	Thrombosis of vein of lower limb
701	QN_AMI_VASC_DZ	312584000	Thrombosis of vein of trunk
701	QN_AMI_VASC_DZ	359553002	Fibromuscular hyperplasia of artery
701	QN_AMI_VASC_DZ	359557001	Disorder of artery
701	QN_AMI_VASC_DZ	399957001	Peripheral arterial occlusive disease
701	QN_AMI_VASC_DZ	400047006	Peripheral vascular disease
701	QN_AMI_VASC_DZ	404223003	Deep venous thrombosis of lower extremity
701	QN_AMI_VASC_DZ	428171009	Pain at rest due to peripheral vascular disease
701	QN_AMI_VASC_DZ	432119003	Aneurysm
701	QN_AMI_VASC_DZ	433068007	Aneurysm of thoracic aorta

© 2021 Matheny ME et al. *JAMA Network Open.*

CONDITIONID	CONDITION_DESCRIPTION	SNOMED_CODE	DESCRIPTION
701	QN_AMI_VASC_DZ	438647008	Thrombosis of subclavian vein
701	QN_AMI_VASC_DZ	438785004	Deep venous thrombosis of tibial vein
701	QN_AMI_VASC_DZ	439470001	Arteriovenous fistula
701	QN_AMI_VASC_DZ	441574008	Atherosclerosis of artery
701	QN_AMI_VASC_DZ	442439008	Atherosclerosis of bypass graft of limb
701	QN_AMI_VASC_DZ	442693003	Atherosclerosis of autologous vein bypass graft of limb
701	QN_AMI_VASC_DZ	442701004	Atherosclerosis of nonautologous biological bypass graft of limb
701	QN_AMI_VASC_DZ	443210003	Deep venous thrombosis of peroneal vein
701	QN_AMI_VASC_DZ	443971004	Arteriosclerosis of artery of extremity
701	QN_AMI_VASC_DZ	449184004	Dilatation of descending aorta
701	QN_AMI_VASC_DZ	698816006	Chronic occlusion of artery of extremity
701	QN_AMI_VASC_DZ	651000119108	Acute deep vein thrombosis of lower limb
701	QN_AMI_VASC_DZ	132151000119108	Chronic deep venous thrombosis of popliteal vein
701	QN_AMI_VASC_DZ	132191000119103	Chronic deep venous thrombosis of femoral vein

© 2021 Matheny ME et al. *JAMA Network Open.*

CONDITIONID	CONDITION_DESCRIPTION	SNOMED_CODE	DESCRIPTION
701	QN_AMI_VASC_DZ	132251000119101	Acute deep venous thrombosis of popliteal vein
701	QN_AMI_VASC_DZ	132261000119104	Acute deep venous thrombosis of tibial vein
701	QN_AMI_VASC_DZ	132291000119106	Acute deep venous thrombosis of femoral vein
701	QN_AMI_VASC_DZ	132551000119104	Chronic thrombosis of subclavian vein
701	QN_AMI_VASC_DZ	132611000119104	Acute thrombosis of subclavian vein
701	QN_AMI_VASC_DZ	145891000119104	Atherosclerosis of native arteries of the extremities
701	QN_AMI_VASC_DZ	864191000000104	Thrombosis of internal jugular vein
711	QN_AMI_OTHER_RESP	1694004	Accessory lobe of lung
711	QN_AMI_OTHER_RESP	4386001	Bronchospasm
711	QN_AMI_OTHER_RESP	5505005	Acute bronchiolitis
711	QN_AMI_OTHER_RESP	10509002	Acute bronchitis
711	QN_AMI_OTHER_RESP	11356007	Foreign body in main bronchus
711	QN_AMI_OTHER_RESP	11356007	Foreign body in main bronchus
711	QN_AMI_OTHER_RESP	14532008	Congenital anomaly of trachea
711	QN_AMI_OTHER_RESP	19829001	Disorder of lung
711	QN_AMI_OTHER_RESP	24385003	Foreign body in lung
711	QN_AMI_OTHER_RESP	27405005	Central sleep apnea syndrome
711	QN_AMI_OTHER_RESP	27405005	Central sleep apnea syndrome
711	QN_AMI_OTHER_RESP	32398004	Bronchitis
711	QN_AMI_OTHER_RESP	41427001	Disorder of bronchus

© 2021 Matheny ME et al. *JAMA Network Open.*

CONDITIONID	CONDITION_DESCRIPTION	SNOMED_CODE	DESCRIPTION
711	QN_AMI_OTHER_RESP	41975002	Insomnia with sleep apnea
711	QN_AMI_OTHER_RESP	45865005	Foreign body in tracheobronchial tree
711	QN_AMI_OTHER_RESP	46621007	Atelectasis
711	QN_AMI_OTHER_RESP	47070001	Congenital web of larynx
711	QN_AMI_OTHER_RESP	47147007	Congenital anomaly of lung
711	QN_AMI_OTHER_RESP	47597000	Mediastinitis
711	QN_AMI_OTHER_RESP	48475001	Disorder of diaphragm
711	QN_AMI_OTHER_RESP	49483002	Disorder of mediastinum
711	QN_AMI_OTHER_RESP	50043002	Disorder of respiratory system
711	QN_AMI_OTHER_RESP	50943000	Congenital anomaly of bronchus
711	QN_AMI_OTHER_RESP	51523009	Congenital laryngocele
711	QN_AMI_OTHER_RESP	54421009	Foreign body in trachea
711	QN_AMI_OTHER_RESP	54421009	Foreign body in trachea
711	QN_AMI_OTHER_RESP	55604004	Avian influenza
711	QN_AMI_OTHER_RESP	60652005	Congenital cyst of mediastinum
711	QN_AMI_OTHER_RESP	61700007	Influenza with non-respiratory manifestation
711	QN_AMI_OTHER_RESP	63039003	Influenza with respiratory manifestation other than pneumonia
711	QN_AMI_OTHER_RESP	66466001	Asphyxiation
711	QN_AMI_OTHER_RESP	66466001	Asphyxiation
711	QN_AMI_OTHER_RESP	72292009	Congenital anomaly of larynx

© 2021 Matheny ME et al. *JAMA Network Open.*

CONDITIONID	CONDITION_DESCRIPTION	SNOMED_CODE	DESCRIPTION
711	QN_AMI_OTHER_RESP	73430006	Sleep apnea
711	QN_AMI_OTHER_RESP	73430006	Sleep apnea
711	QN_AMI_OTHER_RESP	74644004	Influenza with encephalopathy
711	QN_AMI_OTHER_RESP	77593006	Congenital bronchiectasis
711	QN_AMI_OTHER_RESP	78275009	Obstructive sleep apnea syndrome
711	QN_AMI_OTHER_RESP	78275009	Obstructive sleep apnea syndrome
711	QN_AMI_OTHER_RESP	79280005	Hypersomnia with sleep apnea
711	QN_AMI_OTHER_RESP	87119009	Congenital cystic lung
711	QN_AMI_OTHER_RESP	95467005	Congenital tracheomalacia
711	QN_AMI_OTHER_RESP	95468000	Congenital bronchomalacia
711	QN_AMI_OTHER_RESP	195720006	Acute streptococcal bronchitis
711	QN_AMI_OTHER_RESP	195721005	Acute haemophilus influenzae bronchitis
711	QN_AMI_OTHER_RESP	195725001	Acute coxsackievirus bronchitis
711	QN_AMI_OTHER_RESP	195726000	Acute parainfluenza virus bronchitis
711	QN_AMI_OTHER_RESP	195727009	Acute respiratory syncytial virus bronchitis
711	QN_AMI_OTHER_RESP	195728004	Acute bronchitis due to rhinovirus
711	QN_AMI_OTHER_RESP	195729007	Acute echovirus bronchitis
711	QN_AMI_OTHER_RESP	195739001	Acute bronchiolitis due to respiratory syncytial virus

© 2021 Matheny ME et al. *JAMA Network Open.*

CONDITIONID	CONDITION_DESCRIPTION	SNOMED_CODE	DESCRIPTION
711	QN_AMI_OTHER_RESP	195742007	Acute lower respiratory tract infection
711	QN_AMI_OTHER_RESP	204551008	Congenital bronchial stenosis
711	QN_AMI_OTHER_RESP	204552001	Congenital subglottic stenosis
711	QN_AMI_OTHER_RESP	230494007	Alveolar sleep apnea
711	QN_AMI_OTHER_RESP	233599001	Acute mycoplasmal bronchitis
711	QN_AMI_OTHER_RESP	253736003	Laryngeal hypoplasia
711	QN_AMI_OTHER_RESP	253737007	Congenital laryngomalacia
711	QN_AMI_OTHER_RESP	253748000	Anomaly of pleura
711	QN_AMI_OTHER_RESP	262599003	Foreign body in respiratory tract
711	QN_AMI_OTHER_RESP	268195009	Ectopic tissue in lung
711	QN_AMI_OTHER_RESP	275260000	Congenital malformation of the respiratory system
711	QN_AMI_OTHER_RESP	282461001	Foreign body in bronchus
711	QN_AMI_OTHER_RESP	297159008	Laryngeal web
711	QN_AMI_OTHER_RESP	389078002	Transfusion related acute lung injury
711	QN_AMI_OTHER_RESP	399040002	Congenital central hypoventilation
711	QN_AMI_OTHER_RESP	399040002	Congenital central hypoventilation
711	QN_AMI_OTHER_RESP	441910000	Idiopathic sleep related non-obstructive alveolar hypoventilation
711	QN_AMI_OTHER_RESP	441910000	Idiopathic sleep related non-obstructive

© 2021 Matheny ME et al. *JAMA Network Open.*

CONDITIONID	CONDITION_DESCRIPTION	SNOMED_CODE	DESCRIPTION
			alveolar hypoventilation
711	QN_AMI_OTHER_RESP	442164004	Organic sleep apnea
711	QN_AMI_OTHER_RESP	442438000	Influenza due to Influenza A virus
711	QN_AMI_OTHER_RESP	442549008	High altitude periodic breathing
711	QN_AMI_OTHER_RESP	442696006	Influenza due to Influenza A virus subtype H1N1
711	QN_AMI_OTHER_RESP	443760008	Sleep hypoventilation
711	QN_AMI_OTHER_RESP	445102008	Bronchiolitis due to Human metapneumovirus
711	QN_AMI_OTHER_RESP	9741000119101	Primary central sleep apnea
711	QN_AMI_OTHER_RESP	9741000119101	Primary central sleep apnea
712	QN_AMI_BLIND	1909000	Impairment level: better eye: severe impairment: lesser eye: near-total impairment
712	QN_AMI_BLIND	7469001	Impairment level: better eye: near-total impairment: lesser eye: total impairment
712	QN_AMI_BLIND	7758003	Impairment level: better eye: profound impairment: lesser eye: near-total impairment
712	QN_AMI_BLIND	13734003	Impairment level: better eye: profound impairment: lesser

© 2021 Matheny ME et al. *JAMA Network Open.*

CONDITIONID	CONDITION_DESCRIPTION	SNOMED_CODE	DESCRIPTION
			eye: total impairment
712	QN_AMI_BLIND	15010004	Impairment level: better eye: near-total impairment: lesser-eye: not further specified
712	QN_AMI_BLIND	30272003	Impairment level: better eye: severe impairment: lesser eye: profound impairment
712	QN_AMI_BLIND	36676002	Impairment level: profound impairment of both eyes
712	QN_AMI_BLIND	38465001	Impairment level: near-total impairment of both eyes
712	QN_AMI_BLIND	39914007	Impairment level: better eye: severe impairment: lesser eye: not further specified
712	QN_AMI_BLIND	58937003	Impairment level: better eye: severe impairment: lesser eye: total impairment
712	QN_AMI_BLIND	63511007	Impairment level: better eye: profound impairment: lesser eye: not further specified
712	QN_AMI_BLIND	86643000	Impairment level: severe impairment of both eyes

© 2021 Matheny ME et al. *JAMA Network Open.*

CONDITIONID	CONDITION_DESCRIPTION	SNOMED_CODE	DESCRIPTION
712	QN_AMI_BLIND	88768004	Impairment level: total impairment of both eyes
712	QN_AMI_BLIND	105597003	Blindness AND/OR vision impairment level
712	QN_AMI_BLIND	111518009	Impairment level: better eye: severe impairment: lesser eye: blind, not further specified
712	QN_AMI_BLIND	193699007	Blindness - both eyes
712	QN_AMI_BLIND	193731001	Legal blindness USA
715	QN_AMI_DM_PROLIF_RETIN	31341008	Vitreous hemorrhage
716	QN_AMI_DM_OTHER_RETIN	776009	Partial retinal artery occlusion
716	QN_AMI_DM_OTHER_RETIN	4855003	Retinopathy due to diabetes mellitus
716	QN_AMI_DM_OTHER_RETIN	6141006	Retinal edema
716	QN_AMI_DM_OTHER_RETIN	6962006	Hypertensive retinopathy
716	QN_AMI_DM_OTHER_RETIN	24596005	Venous retinal branch occlusion
716	QN_AMI_DM_OTHER_RETIN	25506007	Exudative retinopathy
716	QN_AMI_DM_OTHER_RETIN	28998008	Retinal hemorrhage
716	QN_AMI_DM_OTHER_RETIN	29555009	Retinal disorder
716	QN_AMI_DM_OTHER_RETIN	34037000	Microaneurysm of retinal artery
716	QN_AMI_DM_OTHER_RETIN	38742007	Central retinal artery occlusion
716	QN_AMI_DM_OTHER_RETIN	46674002	Nondiabetic proliferative retinopathy
716	QN_AMI_DM_OTHER_RETIN	50821009	Arterial retinal branch occlusion

© 2021 Matheny ME et al. *JAMA Network Open.*

CONDITIONID	CONDITION_DESCRIPTION	SNOMED_CODE	DESCRIPTION
716	QN_AMI_DM_OTHER_RETIN	57534004	Retinal vascular disorder
716	QN_AMI_DM_OTHER_RETIN	61267008	Retinal neovascularization
716	QN_AMI_DM_OTHER_RETIN	68478007	Central retinal vein occlusion
716	QN_AMI_DM_OTHER_RETIN	73757007	Retinal vascular occlusion
716	QN_AMI_DM_OTHER_RETIN	77628002	Retinal vasculitis
716	QN_AMI_DM_OTHER_RETIN	84884003	Retinal telangiectasia
716	QN_AMI_DM_OTHER_RETIN	87224000	Transient arterial retinal occlusion
716	QN_AMI_DM_OTHER_RETIN	88032003	Amaurosis fugax
716	QN_AMI_DM_OTHER_RETIN	193379006	Retinal venous engorgement
716	QN_AMI_DM_OTHER_RETIN	247136003	Intraretinal microvascular abnormality
716	QN_AMI_DM_OTHER_RETIN	408847006	Retinopathy of prematurity stage 1 - demarcation line
716	QN_AMI_DM_OTHER_RETIN	408848001	Retinopathy of prematurity stage 2 - intraretinal ridge
716	QN_AMI_DM_OTHER_RETIN	408849009	Retinopathy of prematurity stage 3 - ridge with extraretinal fibrovascular proliferation
716	QN_AMI_DM_OTHER_RETIN	408850009	Retinopathy of prematurity stage 4 - subtotal retinal detachment
716	QN_AMI_DM_OTHER_RETIN	408851008	Retinopathy of prematurity stage 5 - total retinal detachment

© 2021 Matheny ME et al. *JAMA Network Open.*

CONDITIONID	CONDITION_DESCRIPTION	SNOMED_CODE	DESCRIPTION
716	QN_AMI_DM_OTHER_RETIN	415297005	Retinopathy of prematurity
716	QN_AMI_DM_OTHER_RETIN	124111000119102	Retinopathy of prematurity stage 0
727	QN_AMI_DIALYSIS	615005	Obstruction due to foreign body accidentally left in operative wound AND/OR body cavity during a procedure
727	QN_AMI_DIALYSIS	7058009	Noncompliance with treatment
727	QN_AMI_DIALYSIS	17778006	Mechanical complication of dialysis catheter
727	QN_AMI_DIALYSIS	19220005	Complication of implant
727	QN_AMI_DIALYSIS	31871009	Infection AND/OR inflammatory reaction due to internal prosthetic device, implant AND/OR graft
727	QN_AMI_DIALYSIS	32825000	Perforation due to foreign body accidentally left in operative wound AND/OR body cavity during a procedure
727	QN_AMI_DIALYSIS	33603003	Complication of renal dialysis
727	QN_AMI_DIALYSIS	61625009	Adhesions due to foreign body accidentally left in operative wound AND/OR body cavity during a procedure

© 2021 Matheny ME et al. *JAMA Network Open.*

CONDITIONID	CONDITION_DESCRIPTION	SNOMED_CODE	DESCRIPTION
727	QN_AMI_DIALYSIS	73257006	Peritoneal dialysis catheter maintenance
727	QN_AMI_DIALYSIS	73257006	Peritoneal dialysis catheter maintenance
727	QN_AMI_DIALYSIS	105502003	Dependence on renal dialysis
727	QN_AMI_DIALYSIS	108241001	Dialysis procedure
727	QN_AMI_DIALYSIS	108241001	Dialysis procedure
727	QN_AMI_DIALYSIS	216904007	Foreign object left in body during kidney dialysis
727	QN_AMI_DIALYSIS	216926008	Failure of sterile precautions during procedure
727	QN_AMI_DIALYSIS	216933008	Failure of sterile precautions during kidney dialysis
727	QN_AMI_DIALYSIS	251859005	Dialysis finding
727	QN_AMI_DIALYSIS	269191009	Late effect of medical and surgical care complication
727	QN_AMI_DIALYSIS	431028002	Mechanical complication of peritoneal dialysis catheter
765	QN_AMI_INT_TRAUMA	171008	Injury of ascending right colon without open wound into abdominal cavity
765	QN_AMI_INT_TRAUMA	188001	Injury of intercostal artery
765	QN_AMI_INT_TRAUMA	658009	Injury of colon without open wound into abdominal cavity
765	QN_AMI_INT_TRAUMA	845006	Injury of inferior mesenteric artery

© 2021 Matheny ME et al. *JAMA Network Open.*

CONDITIONID	CONDITION_DESCRIPTION	SNOMED_CODE	DESCRIPTION
765	QN_AMI_INT_TRAUMA	1264004	Injury of descending left colon without open wound into abdominal cavity
765	QN_AMI_INT_TRAUMA	1351002	Injury of iliac artery
765	QN_AMI_INT_TRAUMA	1367008	Injury of superior mesenteric artery
765	QN_AMI_INT_TRAUMA	2973002	Pelvic organ injury without open wound into abdominal cavity
765	QN_AMI_INT_TRAUMA	3759007	Injury of heart with open wound into thorax
765	QN_AMI_INT_TRAUMA	3913002	Injury of gastrointestinal tract with open wound into abdominal cavity
765	QN_AMI_INT_TRAUMA	5256002	Injury of spleen with open wound into abdominal cavity
765	QN_AMI_INT_TRAUMA	5753006	Injury of renal vein
765	QN_AMI_INT_TRAUMA	7346000	Injury of inferior mesenteric vein
765	QN_AMI_INT_TRAUMA	8281003	Injury of uterine artery
765	QN_AMI_INT_TRAUMA	9298006	Hematoma of kidney without rupture of capsule AND without open wound into abdominal cavity
765	QN_AMI_INT_TRAUMA	10392004	Injury of abdominal aorta
765	QN_AMI_INT_TRAUMA	11229008	Laceration of heart without penetration of heart chambers

© 2021 Matheny ME et al. *JAMA Network Open.*

CONDITIONID	CONDITION_DESCRIPTION	SNOMED_CODE	DESCRIPTION
765	QN_AMI_INT_TRAUMA	12227007	Capsular tear without major disruption of parenchyma of spleen AND without open wound into abdominal cavity
765	QN_AMI_INT_TRAUMA	13891000	Major laceration of liver without open wound into abdominal cavity
765	QN_AMI_INT_TRAUMA	14180000	Injury of splenic vein
765	QN_AMI_INT_TRAUMA	15151004	Injury of liver with open wound into abdominal cavity
765	QN_AMI_INT_TRAUMA	17414004	Contusion to heart
765	QN_AMI_INT_TRAUMA	17567001	Injury of hepatic artery
765	QN_AMI_INT_TRAUMA	18147000	Injury of colon with open wound into abdominal cavity
765	QN_AMI_INT_TRAUMA	18296004	Injury of ovarian artery
765	QN_AMI_INT_TRAUMA	18796000	Injury of renal artery
765	QN_AMI_INT_TRAUMA	20213006	Injury of spleen without open wound into abdominal cavity
765	QN_AMI_INT_TRAUMA	20474007	Injury of multiple sites of pancreas without open wound into abdominal cavity
765	QN_AMI_INT_TRAUMA	20580001	Injury of bronchus with open wound into thoracic cavity
765	QN_AMI_INT_TRAUMA	20784007	Internal injury of abdominal organs

© 2021 Matheny ME et al. *JAMA Network Open.*

CONDITIONID	CONDITION_DESCRIPTION	SNOMED_CODE	DESCRIPTION
			with open wound into cavity
765	QN_AMI_INT_TRAUMA	22095002	Injury of adrenal gland without open wound into abdominal cavity
765	QN_AMI_INT_TRAUMA	22724000	Injury of urethra
765	QN_AMI_INT_TRAUMA	22897006	Traumatic pneumothorax with open wound into thorax
765	QN_AMI_INT_TRAUMA	23368007	Injury of uterus without open wound into abdominal cavity
765	QN_AMI_INT_TRAUMA	23589004	Injury of spleen
765	QN_AMI_INT_TRAUMA	24179004	Hematoma AND contusion of liver without open wound into abdominal cavity
765	QN_AMI_INT_TRAUMA	24350003	Injury of lung without open wound into thorax
765	QN_AMI_INT_TRAUMA	24850009	Injury of ureter
765	QN_AMI_INT_TRAUMA	25110002	Injury of multiple sites in colon AND/OR rectum with open wound into abdominal cavity
765	QN_AMI_INT_TRAUMA	25420003	Injury of multiple sites of pancreas with open wound into abdominal cavity
765	QN_AMI_INT_TRAUMA	25554004	Major laceration of liver with open wound into abdominal cavity

© 2021 Matheny ME et al. *JAMA Network Open.*

CONDITIONID	CONDITION_DESCRIPTION	SNOMED_CODE	DESCRIPTION
765	QN_AMI_INT_TRAUMA	27817002	Internal injury of chest
765	QN_AMI_INT_TRAUMA	28545001	Injury of renal vessels
765	QN_AMI_INT_TRAUMA	29691006	Injury of multiple sites in colon AND/OR rectum without open wound into abdominal cavity
765	QN_AMI_INT_TRAUMA	29880001	Injury of rectum with open wound into abdominal cavity
765	QN_AMI_INT_TRAUMA	31110000	Injury of ovarian vein
765	QN_AMI_INT_TRAUMA	33072005	Traumatic hemothorax with open wound into thorax
765	QN_AMI_INT_TRAUMA	33908005	Injury of peritoneum without open wound into abdominal cavity
765	QN_AMI_INT_TRAUMA	34047002	Injury of multiple intrathoracic organs without open wound into cavity
765	QN_AMI_INT_TRAUMA	34704009	Injury of portal vein
765	QN_AMI_INT_TRAUMA	35191005	Injury of blood vessels of abdomen AND/OR pelvis
765	QN_AMI_INT_TRAUMA	35790004	Injury of retroperitoneum without open wound into abdominal cavity

© 2021 Matheny ME et al. *JAMA Network Open.*

CONDITIONID	CONDITION_DESCRIPTION	SNOMED_CODE	DESCRIPTION
765	QN_AMI_INT_TRAUMA	36787001	Contusion of trunk
765	QN_AMI_INT_TRAUMA	38261007	Injury of superior vena cava
765	QN_AMI_INT_TRAUMA	38972005	Injury of sigmoid colon without open wound into abdominal cavity
765	QN_AMI_INT_TRAUMA	39400004	Injury of liver
765	QN_AMI_INT_TRAUMA	40095003	Injury of kidney
765	QN_AMI_INT_TRAUMA	40521006	Contusion to heart with open wound into thorax
765	QN_AMI_INT_TRAUMA	41547006	Injury of bronchus without open wound into thoracic cavity
765	QN_AMI_INT_TRAUMA	42019003	Contusion of lung with open wound into thorax
765	QN_AMI_INT_TRAUMA	42434002	Traumatic pneumohemothorax
765	QN_AMI_INT_TRAUMA	42458003	Traumatic hemothorax
765	QN_AMI_INT_TRAUMA	43841005	Moderate laceration of liver without open wound into abdominal cavity
765	QN_AMI_INT_TRAUMA	43842003	Injury of lung with open wound into thorax
765	QN_AMI_INT_TRAUMA	44599007	Traumatic hemothorax without open wound into thorax
765	QN_AMI_INT_TRAUMA	46328008	Injury of ureter without open wound into abdominal cavity

© 2021 Matheny ME et al. *JAMA Network Open.*

CONDITIONID	CONDITION_DESCRIPTION	SNOMED_CODE	DESCRIPTION
765	QN_AMI_INT_TRAUMA	47468000	Injury of gastric artery
765	QN_AMI_INT_TRAUMA	47533003	Injury of tail of pancreas without open wound into abdominal cavity
765	QN_AMI_INT_TRAUMA	47771009	Injury of gastrointestinal tract
765	QN_AMI_INT_TRAUMA	48125009	Injury of trunk
765	QN_AMI_INT_TRAUMA	48424004	Contusion of lung without open wound into thorax
765	QN_AMI_INT_TRAUMA	48653005	Injury of appendix without open wound into abdominal cavity
765	QN_AMI_INT_TRAUMA	49011004	Internal injury of abdominal organs
765	QN_AMI_INT_TRAUMA	49280002	Injury of multiple intra-abdominal organs with open wound into abdominal cavity
765	QN_AMI_INT_TRAUMA	50088001	Hematoma of spleen without rupture of capsule AND without open wound into abdominal cavity
765	QN_AMI_INT_TRAUMA	51000003	Injury of multiple intra-abdominal organs without open wound into abdominal cavity
765	QN_AMI_INT_TRAUMA	51536009	Injury of hypogastric artery
765	QN_AMI_INT_TRAUMA	52087004	Injury of rectum without open wound into abdominal cavity

© 2021 Matheny ME et al. *JAMA Network Open.*

CONDITIONID	CONDITION_DESCRIPTION	SNOMED_CODE	DESCRIPTION
765	QN_AMI_INT_TRAUMA	56472003	Injury of pleura
765	QN_AMI_INT_TRAUMA	56515006	Injury of inferior vena cava
765	QN_AMI_INT_TRAUMA	57662003	Injury of blood vessel
765	QN_AMI_INT_TRAUMA	58565006	Injury of head of pancreas without open wound into cavity
765	QN_AMI_INT_TRAUMA	59190009	Traumatic pneumohemothorax without open wound into thorax
765	QN_AMI_INT_TRAUMA	59568004	Injury of thoracic aorta
765	QN_AMI_INT_TRAUMA	60190000	Injury of blood vessels of thorax
765	QN_AMI_INT_TRAUMA	60366008	Injury of pulmonary vein
765	QN_AMI_INT_TRAUMA	61014001	Laceration extending into parenchyma of spleen without open wound into abdominal cavity
765	QN_AMI_INT_TRAUMA	61474001	Injury of kidney with open wound into abdominal cavity
765	QN_AMI_INT_TRAUMA	61812003	Injury of multiple blood vessels of abdomen AND/OR pelvis
765	QN_AMI_INT_TRAUMA	61823004	Injury of pancreas
765	QN_AMI_INT_TRAUMA	64042000	Injury of uterine vein
765	QN_AMI_INT_TRAUMA	64323009	Complete disruption of kidney parenchyma without open wound into cavity

© 2021 Matheny ME et al. *JAMA Network Open.*

CONDITIONID	CONDITION_DESCRIPTION	SNOMED_CODE	DESCRIPTION
765	QN_AMI_INT_TRAUMA	64638002	Injury of hepatic vein
765	QN_AMI_INT_TRAUMA	64672005	Injury of innominate artery
765	QN_AMI_INT_TRAUMA	65071008	Injury of gastrointestinal tract without open wound into abdominal cavity
765	QN_AMI_INT_TRAUMA	65324009	Minor laceration of liver without open wound into abdominal cavity
765	QN_AMI_INT_TRAUMA	65785006	Injury of superior mesenteric vein AND/OR primary subdivisions
765	QN_AMI_INT_TRAUMA	66829004	Injury of splenic artery
765	QN_AMI_INT_TRAUMA	67447005	Injury of body of pancreas without open wound into abdominal cavity
765	QN_AMI_INT_TRAUMA	68708005	Injury of celiac AND/OR mesenteric arteries
765	QN_AMI_INT_TRAUMA	68734002	Injury of small intestine without open wound into abdominal cavity
765	QN_AMI_INT_TRAUMA	70092007	Contusion of kidney
765	QN_AMI_INT_TRAUMA	73477001	Traumatic pneumohemothorax with open wound into thorax
765	QN_AMI_INT_TRAUMA	73557005	Injury of internal mammary vein
765	QN_AMI_INT_TRAUMA	74324004	Laceration of kidney without

© 2021 Matheny ME et al. *JAMA Network Open.*

CONDITIONID	CONDITION_DESCRIPTION	SNOMED_CODE	DESCRIPTION
			open wound into abdominal cavity
765	QN_AMI_INT_TRAUMA	76211000	Injury of iliac vein
765	QN_AMI_INT_TRAUMA	76877000	Injury of diaphragm with open wound into cavity
765	QN_AMI_INT_TRAUMA	77165001	Injury of bladder
765	QN_AMI_INT_TRAUMA	77940004	Hematoma of spleen without rupture of capsule AND with open wound into abdominal cavity
765	QN_AMI_INT_TRAUMA	80192004	Massive parenchymal disruption of spleen with open wound into abdominal cavity
765	QN_AMI_INT_TRAUMA	81193001	Pelvic organ injury with open wound into abdominal cavity
765	QN_AMI_INT_TRAUMA	83209008	Injury of transverse colon without open wound into abdominal cavity
765	QN_AMI_INT_TRAUMA	83276000	Injury of uterus with open wound into abdominal cavity
765	QN_AMI_INT_TRAUMA	84338002	Laceration of heart with penetration of heart chambers
765	QN_AMI_INT_TRAUMA	84621006	Injury of esophagus with open wound into thoracic cavity
765	QN_AMI_INT_TRAUMA	85719003	Injury of subclavian vein

© 2021 Matheny ME et al. *JAMA Network Open.*

CONDITIONID	CONDITION_DESCRIPTION	SNOMED_CODE	DESCRIPTION
765	QN_AMI_INT_TRAUMA	86006001	Massive parenchymal disruption of spleen without open wound into abdominal cavity
765	QN_AMI_INT_TRAUMA	86175003	Injury of heart
765	QN_AMI_INT_TRAUMA	87438005	Injury of multiple blood vessels of thorax
765	QN_AMI_INT_TRAUMA	88837005	Injury of pulmonary artery
765	QN_AMI_INT_TRAUMA	88898002	Injury of primary branch of superior mesenteric artery
765	QN_AMI_INT_TRAUMA	90070003	Traumatic pneumothorax
765	QN_AMI_INT_TRAUMA	105612003	Injury of internal organ
765	QN_AMI_INT_TRAUMA	105615001	Open wound of trunk
765	QN_AMI_INT_TRAUMA	111683002	Injury of duodenum without open wound into abdominal cavity
765	QN_AMI_INT_TRAUMA	111694003	Injury of branch of celiac axis
765	QN_AMI_INT_TRAUMA	125626004	Injury of stomach
765	QN_AMI_INT_TRAUMA	125627008	Injury of small intestine
765	QN_AMI_INT_TRAUMA	125628003	Injury of duodenum
765	QN_AMI_INT_TRAUMA	125629006	Injury of colon
765	QN_AMI_INT_TRAUMA	125631002	Injury of ascending colon
765	QN_AMI_INT_TRAUMA	125632009	Injury of transverse colon
765	QN_AMI_INT_TRAUMA	125633004	Injury of descending colon
765	QN_AMI_INT_TRAUMA	125634005	Injury of sigmoid colon

© 2021 Matheny ME et al. *JAMA Network Open.*

CONDITIONID	CONDITION_DESCRIPTION	SNOMED_CODE	DESCRIPTION
765	QN_AMI_INT_TRAUMA	125635006	Injury of rectum
765	QN_AMI_INT_TRAUMA	125637003	Injury of uterus
765	QN_AMI_INT_TRAUMA	125638008	Injury of Fallopian tube
765	QN_AMI_INT_TRAUMA	125639000	Injury of ovary
765	QN_AMI_INT_TRAUMA	125640003	Injury of prostate
765	QN_AMI_INT_TRAUMA	125643001	Open wound
765	QN_AMI_INT_TRAUMA	125670008	Foreign body
765	QN_AMI_INT_TRAUMA	127313006	Injury of bile duct
765	QN_AMI_INT_TRAUMA	127314000	Open wound of chest wall
765	QN_AMI_INT_TRAUMA	128069005	Injury of abdomen
765	QN_AMI_INT_TRAUMA	128125000	Injury of bronchus
765	QN_AMI_INT_TRAUMA	210063008	Heart laceration with open wound into thorax, without penetration of heart chambers
765	QN_AMI_INT_TRAUMA	210064002	Heart laceration with open wound into thorax, with penetration of heart chambers
765	QN_AMI_INT_TRAUMA	210076002	Lung laceration with open wound into thorax
765	QN_AMI_INT_TRAUMA	210078001	Injury of heart with hemopericardium
765	QN_AMI_INT_TRAUMA	210083009	Closed injury of diaphragm
765	QN_AMI_INT_TRAUMA	210100006	Injury of thoracic trachea
765	QN_AMI_INT_TRAUMA	210106000	Multiple intrathoracic organ injury with open wound into cavity
765	QN_AMI_INT_TRAUMA	210109007	Stomach injury with open wound into cavity

© 2021 Matheny ME et al. *JAMA Network Open.*

CONDITIONID	CONDITION_DESCRIPTION	SNOMED_CODE	DESCRIPTION
765	QN_AMI_INT_TRAUMA	210114006	Injury of small intestine with open wound into abdominal cavity
765	QN_AMI_INT_TRAUMA	210116008	Injury of duodenum with open wound into abdominal cavity
765	QN_AMI_INT_TRAUMA	210128000	Colon or rectum injury with open wound into cavity
765	QN_AMI_INT_TRAUMA	210130003	Injury of ascending right colon with open wound into abdominal cavity
765	QN_AMI_INT_TRAUMA	210131004	Injury of transverse colon with open wound into abdominal cavity
765	QN_AMI_INT_TRAUMA	210132006	Injury of descending left colon with open wound into abdominal cavity
765	QN_AMI_INT_TRAUMA	210133001	Sigmoid colon injury with open wound into cavity
765	QN_AMI_INT_TRAUMA	210155009	Injury of head of pancreas with open wound into abdominal cavity
765	QN_AMI_INT_TRAUMA	210156005	Pancreas body injury with open wound into cavity
765	QN_AMI_INT_TRAUMA	210157001	Injury of tail of pancreas with open wound into abdominal cavity
765	QN_AMI_INT_TRAUMA	210159003	Injury of appendix with open wound

© 2021 Matheny ME et al. *JAMA Network Open.*

CONDITIONID	CONDITION_DESCRIPTION	SNOMED_CODE	DESCRIPTION
			into abdominal cavity
765	QN_AMI_INT_TRAUMA	210173007	Liver hematoma and contusion with open wound into cavity
765	QN_AMI_INT_TRAUMA	210174001	Liver minor laceration with open wound into cavity
765	QN_AMI_INT_TRAUMA	210175000	Liver moderate laceration with open wound into cavity
765	QN_AMI_INT_TRAUMA	210191002	Spleen capsular tear without major disruption of parenchyma, with open wound into cavity
765	QN_AMI_INT_TRAUMA	210192009	Spleen laceration extending into parenchyma with open wound into cavity
765	QN_AMI_INT_TRAUMA	210205007	Kidney hematoma without rupture of capsule, with open wound into cavity
765	QN_AMI_INT_TRAUMA	210206008	Kidney laceration with open wound into cavity
765	QN_AMI_INT_TRAUMA	210207004	Complete disruption of kidney parenchyma with open wound into cavity
765	QN_AMI_INT_TRAUMA	210215001	Bladder and urethra injury with open wound into cavity

© 2021 Matheny ME et al. *JAMA Network Open.*

CONDITIONID	CONDITION_DESCRIPTION	SNOMED_CODE	DESCRIPTION
765	QN_AMI_INT_TRAUMA	210220001	Injury of ureter with open wound into abdominal cavity
765	QN_AMI_INT_TRAUMA	210260000	Injury of adrenal gland with open wound into abdominal cavity
765	QN_AMI_INT_TRAUMA	210262008	Peritoneum injury with open wound into cavity
765	QN_AMI_INT_TRAUMA	210263003	Retroperitoneum injury with open wound into cavity
765	QN_AMI_INT_TRAUMA	210410008	Open wound of front wall of thorax
765	QN_AMI_INT_TRAUMA	210412000	Open wound of back wall of thorax
765	QN_AMI_INT_TRAUMA	210456000	Open wound of anterior abdominal wall
765	QN_AMI_INT_TRAUMA	210457009	Open wound of epigastric region
765	QN_AMI_INT_TRAUMA	210458004	Open wound umbilical region
765	QN_AMI_INT_TRAUMA	210470000	Open wound of hypochondrium
765	QN_AMI_INT_TRAUMA	210472008	Open wound of iliac region
765	QN_AMI_INT_TRAUMA	210790008	Pulmonary blood vessel injury
765	QN_AMI_INT_TRAUMA	210809009	Superior mesenteric vein injury
765	QN_AMI_INT_TRAUMA	210814008	Iliac blood vessel injury
765	QN_AMI_INT_TRAUMA	210816005	Injury of internal iliac vein
765	QN_AMI_INT_TRAUMA	242006000	Blast lung
765	QN_AMI_INT_TRAUMA	242007009	Blast injury to intestines

© 2021 Matheny ME et al. *JAMA Network Open.*

CONDITIONID	CONDITION_DESCRIPTION	SNOMED_CODE	DESCRIPTION
765	QN_AMI_INT_TRAUMA	262681003	Contusion of trachea
765	QN_AMI_INT_TRAUMA	262782002	Laceration of heart
765	QN_AMI_INT_TRAUMA	262784001	Contusion of lung
765	QN_AMI_INT_TRAUMA	262785000	Laceration of lung
765	QN_AMI_INT_TRAUMA	262787008	Injury of diaphragm
765	QN_AMI_INT_TRAUMA	262791003	Contusion of esophagus
765	QN_AMI_INT_TRAUMA	262793000	Laceration of esophagus
765	QN_AMI_INT_TRAUMA	262799001	Contusion of liver
765	QN_AMI_INT_TRAUMA	262802005	Laceration of liver
765	QN_AMI_INT_TRAUMA	262805007	Injury of gallbladder
765	QN_AMI_INT_TRAUMA	262809001	Contusion of gallbladder
765	QN_AMI_INT_TRAUMA	262810006	Laceration of gallbladder
765	QN_AMI_INT_TRAUMA	262812003	Bile duct injury without open wound into cavity
765	QN_AMI_INT_TRAUMA	262820001	Contusion of spleen
765	QN_AMI_INT_TRAUMA	262822009	Laceration of spleen
765	QN_AMI_INT_TRAUMA	262823004	Capsular tear of spleen
765	QN_AMI_INT_TRAUMA	262827003	Contusion of pancreas
765	QN_AMI_INT_TRAUMA	262829000	Laceration of pancreas parenchyma
765	QN_AMI_INT_TRAUMA	262831009	Injury of adrenal gland
765	QN_AMI_INT_TRAUMA	262833007	Contusion of adrenal gland
765	QN_AMI_INT_TRAUMA	262834001	Laceration of adrenal gland
765	QN_AMI_INT_TRAUMA	262839006	Contusion of stomach

© 2021 Matheny ME et al. *JAMA Network Open.*

CONDITIONID	CONDITION_DESCRIPTION	SNOMED_CODE	DESCRIPTION
765	QN_AMI_INT_TRAUMA	262841007	Laceration of stomach
765	QN_AMI_INT_TRAUMA	262845003	Contusion of duodenum
765	QN_AMI_INT_TRAUMA	262850009	Laceration of duodenum
765	QN_AMI_INT_TRAUMA	262853006	Contusion of small intestine
765	QN_AMI_INT_TRAUMA	262855004	Laceration of small intestine
765	QN_AMI_INT_TRAUMA	262871004	Contusion of colon
765	QN_AMI_INT_TRAUMA	262873001	Laceration of colon
765	QN_AMI_INT_TRAUMA	262877000	Contusion of rectum
765	QN_AMI_INT_TRAUMA	262879002	Laceration of rectum
765	QN_AMI_INT_TRAUMA	262893009	Laceration of kidney
765	QN_AMI_INT_TRAUMA	262926008	Laceration of ovary
765	QN_AMI_INT_TRAUMA	269159008	Injury of pelvic organs
765	QN_AMI_INT_TRAUMA	269162006	Bile duct/gallbladder injury with open wound into cavity
765	QN_AMI_INT_TRAUMA	274167004	Laceration of chest wall
765	QN_AMI_INT_TRAUMA	274170000	Open wound of abdominal wall
765	QN_AMI_INT_TRAUMA	282459005	Laceration of trachea
765	QN_AMI_INT_TRAUMA	282463003	Laceration of bronchus
765	QN_AMI_INT_TRAUMA	282723003	Contusion of bronchus
765	QN_AMI_INT_TRAUMA	283375001	Laceration of trunk
765	QN_AMI_INT_TRAUMA	283378004	Laceration of abdomen
765	QN_AMI_INT_TRAUMA	283522004	Puncture wound of trunk
765	QN_AMI_INT_TRAUMA	283906006	Contusion of ureter

© 2021 Matheny ME et al. *JAMA Network Open.*

CONDITIONID	CONDITION_DESCRIPTION	SNOMED_CODE	DESCRIPTION
765	QN_AMI_INT_TRAUMA	283908007	Laceration of ureter
765	QN_AMI_INT_TRAUMA	283915004	Contusion of bladder
765	QN_AMI_INT_TRAUMA	283916003	Laceration of bladder
765	QN_AMI_INT_TRAUMA	283918002	Contusion of urethra
765	QN_AMI_INT_TRAUMA	283922007	Laceration of urethra
765	QN_AMI_INT_TRAUMA	283928006	Contusion of prostate
765	QN_AMI_INT_TRAUMA	283930008	Laceration of prostate
765	QN_AMI_INT_TRAUMA	283956001	Contusion of ovary
765	QN_AMI_INT_TRAUMA	283957005	Contusion of fallopian tube
765	QN_AMI_INT_TRAUMA	283959008	Laceration of fallopian tube
765	QN_AMI_INT_TRAUMA	283961004	Contusion of uterus
765	QN_AMI_INT_TRAUMA	283963001	Laceration of uterus
765	QN_AMI_INT_TRAUMA	284006002	Injury of thoracic cavity
765	QN_AMI_INT_TRAUMA	285708004	Injury of systemic artery
765	QN_AMI_INT_TRAUMA	285751000	Injury of vein of trunk
765	QN_AMI_INT_TRAUMA	285808008	Injury of peritoneum
765	QN_AMI_INT_TRAUMA	286603008	Foreign body of body cavity and wall
765	QN_AMI_INT_TRAUMA	308890008	Intra-abdominal foreign body
765	QN_AMI_INT_TRAUMA	316358009	Injury of lung
765	QN_AMI_INT_TRAUMA	320934008	Esophageal injury
765	QN_AMI_INT_TRAUMA	446390008	Traumatic injury of body of pancreas

© 2021 Matheny ME et al. *JAMA Network Open.*

CONDITIONID	CONDITION_DESCRIPTION	SNOMED_CODE	DESCRIPTION
765	QN_AMI_INT_TRAUMA	446391007	Traumatic injury of head of pancreas
765	QN_AMI_INT_TRAUMA	446392000	Traumatic injury of tail of pancreas
765	QN_AMI_INT_TRAUMA	447260005	Traumatic injury of celiac artery
765	QN_AMI_INT_TRAUMA	285821000119105	Major laceration of heart with hemopericardium
765	QN_AMI_INT_TRAUMA	286001000119104	Minor laceration of heart with hemopericardium
765	QN_AMI_INT_TRAUMA	286041000119102	Moderate laceration of heart with hemopericardium
1001	AMI	54329005	Acute myocardial infarction of anterior wall
1001	AMI	57054005	Acute myocardial infarction
1001	AMI	58612006	Acute myocardial infarction of lateral wall
1001	AMI	65547006	Acute myocardial infarction of inferolateral wall
1001	AMI	70211005	Acute myocardial infarction of anterolateral wall
1001	AMI	70422006	Acute subendocardial infarction
1001	AMI	73795002	Acute myocardial infarction of inferior wall
1001	AMI	76593002	Acute myocardial infarction of inferoposterior wall

© 2021 Matheny ME et al. *JAMA Network Open.*

CONDITIONID	CONDITION_DESCRIPTION	SNOMED_CODE	DESCRIPTION
1001	AMI	194802003	True posterior myocardial infarction
1001	AMI	401303003	Acute ST segment elevation myocardial infarction
1001	AMI	401314000	Acute non-ST segment elevation myocardial infarction
1001	AMI	15713121000119100	Acute ST segment elevation myocardial infarction due to right coronary artery occlusion
1002	Cardiac Surgery	116360008	Arterial bypass graft
1002	Cardiac Surgery	232717009	Coronary artery bypass graft
1002	Cardiac Surgery	232720001	Coronary artery bypass grafts x 2
1002	Cardiac Surgery	232721002	Coronary artery bypass grafts x 3
1002	Cardiac Surgery	232722009	Coronary artery bypass grafts x 4
1002	Cardiac Surgery	232723004	Coronary artery bypass grafts x 5
1002	Cardiac Surgery	232724005	Coronary artery bypass grafts greater than 5
1002	Cardiac Surgery	287347004	Arterial bypass using vein graft
1003	Chest Pain	29857009	Chest pain
1004	Coronary Artery Disease	4557003	Preinfarction syndrome
1004	Coronary Artery Disease	23687008	Coronary artery spasm
1004	Coronary Artery Disease	194828000	Angina pectoris

© 2021 Matheny ME et al. *JAMA Network Open.*

CONDITIONID	CONDITION_DESCRIPTION	SNOMED_CODE	DESCRIPTION
1004	Coronary Artery Disease	1641000119107	Coronary arteriosclerosis in native artery
1005	Disorders of magnesium metabolism	60853003	Disorder of magnesium metabolism
1005	Disorders of magnesium metabolism	66978005	Hypermagnesemia
1005	Disorders of magnesium metabolism	190855004	Hypomagnesemia
1006	Hemorrhagic Stroke	21454007	Subarachnoid hemorrhage
1006	Hemorrhagic Stroke	274100004	Cerebral hemorrhage
1007	Hypercholesterolemia	267432004	Pure hypercholesterolemia
1008	Hypervolemia	21639008	Hypervolemia
1008	Hypervolemia	35633007	Transfusion reaction due to excess volume
1009	Hypokalemia	43339004	Hypokalemia
1010	Ischemic Stroke	43658003	Vertebral artery obstruction
1010	Ischemic Stroke	62914000	Cerebrovascular disease
1010	Ischemic Stroke	64775002	Vertebral artery thrombosis
1010	Ischemic Stroke	65084004	Vertebral artery embolism
1010	Ischemic Stroke	69798007	Carotid artery obstruction
1010	Ischemic Stroke	71444005	Cerebral thrombosis
1010	Ischemic Stroke	80606009	Carotid artery embolism
1010	Ischemic Stroke	86003009	Carotid artery thrombosis
1010	Ischemic Stroke	88174006	Basilar artery thrombosis
1010	Ischemic Stroke	111296006	Basilar artery embolism

© 2021 Matheny ME et al. *JAMA Network Open.*

CONDITIONID	CONDITION_DESCRIPTION	SNOMED_CODE	DESCRIPTION
1010	Ischemic Stroke	195183002	Multiple and bilateral precerebral arterial occlusion
1010	Ischemic Stroke	195185009	Cerebral infarct due to thrombosis of precerebral arteries
1010	Ischemic Stroke	195186005	Cerebral infarction due to embolism of precerebral arteries
1010	Ischemic Stroke	195189003	Cerebral infarction due to thrombosis of cerebral arteries
1010	Ischemic Stroke	195190007	Cerebral infarction due to embolism of cerebral arteries
1010	Ischemic Stroke	195230003	Cerebral infarction due to cerebral venous thrombosis, non-pyogenic
1010	Ischemic Stroke	195232006	Occlusion and stenosis of middle cerebral artery
1010	Ischemic Stroke	195233001	Occlusion and stenosis of anterior cerebral artery
1010	Ischemic Stroke	195234007	Occlusion and stenosis of posterior cerebral artery
1010	Ischemic Stroke	195235008	Occlusion and stenosis of cerebellar arteries
1010	Ischemic Stroke	230692004	Infarction - precerebral
1010	Ischemic Stroke	266253001	Precerebral arterial occlusion

© 2021 Matheny ME et al. *JAMA Network Open.*

CONDITIONID	CONDITION_DESCRIPTION	SNOMED_CODE	DESCRIPTION
1010	Ischemic Stroke	266254007	Carotid artery occlusion
1010	Ischemic Stroke	288723005	Acute ill-defined cerebrovascular disease
1010	Ischemic Stroke	432504007	Cerebral infarction
1010	Ischemic Stroke	705128004	Cerebral infarction due to embolism of middle cerebral artery
1010	Ischemic Stroke	705130002	Cerebral infarction due to thrombosis of middle cerebral artery
1010	Ischemic Stroke	34181000119102	Cerebral infarction due to occlusion of basilar artery
1010	Ischemic Stroke	125081000119106	Cerebral infarction due to occlusion of precerebral artery
1010	Ischemic Stroke	285161000119105	Left carotid artery occlusion
1010	Ischemic Stroke	285171000119104	Right carotid artery occlusion
1010	Ischemic Stroke	285191000119103	Left carotid artery stenosis
1010	Ischemic Stroke	285201000119100	Right carotid artery stenosis
1011	JVD	49601007	Disorder of cardiovascular system
1011	JVD	90507008	Disorder of vein
1012	Long-term anticoagulants	182764009	Anticoagulant therapy
1012	Long-term anticoagulants	243872007	High risk drug monitoring status
1012	Long-term anticoagulants	711150003	Long-term current use of anticoagulant
1013	LVEF	85232009	Left heart failure

© 2021 Matheny ME et al. *JAMA Network Open.*

CONDITIONID	CONDITION_DESCRIPTION	SNOMED_CODE	DESCRIPTION
1014	Presence of cardiac device	416940007	Past history of procedure
1014	Presence of cardiac device	429074009	History of cardiovascular surgery
1015	Pulmonary Edema	40541001	Acute pulmonary edema
1016	Rales	362965005	Disorder of body system
1016	Rales	449264008	Auscultation of lower respiratory tract
1017	Rehabilitation	5154007	Speech therapy
1017	Rehabilitation	12895002	Physiotherapeutic breathing exercise
1017	Rehabilitation	40505001	Orthoptic training
1017	Rehabilitation	52052004	Rehabilitation therapy
1017	Rehabilitation	70082004	Vocational rehabilitation
1017	Rehabilitation	74914000	Gait training procedure
1017	Rehabilitation	84478008	Occupational therapy
1017	Rehabilitation	108305003	Physical rehabilitation therapy procedure
1017	Rehabilitation	217897003	Accidental collision with stationary object
1017	Rehabilitation	308335008	Patient encounter procedure
1017	Rehabilitation	413467001	Aftercare
1018	Revascularization	10190003	Aortocoronary bypass of four or more coronary arteries
1018	Revascularization	14323007	Aortocoronary bypass of three coronary arteries

© 2021 Matheny ME et al. *JAMA Network Open.*

CONDITIONID	CONDITION_DESCRIPTION	SNOMED_CODE	DESCRIPTION
1018	Revascularization	15256002	Transmyocardial revascularization by laser technique
1018	Revascularization	29819009	Aortocoronary bypass of one coronary artery
1018	Revascularization	39724006	Anastomosis of internal mammary artery to coronary artery, double vessel
1018	Revascularization	63077009	Implantation of blood vessels into myocardium
1018	Revascularization	67166004	Aortocoronary artery bypass graft
1018	Revascularization	81266008	Heart revascularization
1018	Revascularization	90487008	Aortocoronary bypass of two coronary arteries
1018	Revascularization	232717009	Coronary artery bypass graft
1018	Revascularization	359597003	Single internal mammary-coronary artery bypass
1019	Urinary tract infection	68566005	Urinary tract infectious disease

© 2021 Matheny ME et al. *JAMA Network Open.*
